# Force-controlled release of small molecules with a rotaxane actuator

**DOI:** 10.1038/s41586-024-07154-0

**Published:** 2024-04-10

**Authors:** Lei Chen, Robert Nixon, Guillaume De Bo

**Affiliations:** Department of Chemistry, University of Manchester, Manchester, UK

**Keywords:** Interlocked molecules, Mechanical properties

## Abstract

Force-controlled release of small molecules offers great promise for the delivery of drugs and the release of healing or reporting agents in a medical or materials context^1–3^. In polymer mechanochemistry, polymers are used as actuators to stretch mechanosensitive molecules (mechanophores)^4^. This technique has enabled the release of molecular cargo by rearrangement, as a direct^5,6^ or indirect^7–10^ consequence of bond scission in a mechanophore, or by dissociation of cage^11^, supramolecular^12^ or metal complexes^13,14^, and even by ‘flex activation’^15,16^. However, the systems described so far are limited in the diversity and/or quantity of the molecules released per stretching event^1,2^. This is due to the difficulty in iteratively activating scissile mechanophores, as the actuating polymers will dissociate after the first activation. Physical encapsulation strategies can be used to deliver a larger cargo load, but these are often subject to non-specific (that is, non-mechanical) release^3^. Here we show that a rotaxane (an interlocked molecule in which a macrocycle is trapped on a stoppered axle) acts as an efficient actuator to trigger the release of cargo molecules appended to its axle. The release of up to five cargo molecules per rotaxane actuator was demonstrated in solution, by ultrasonication, and in bulk, by compression, achieving a release efficiency of up to 71% and 30%, respectively, which places this rotaxane device among the most efficient release systems achieved so far^1^. We also demonstrate the release of three representative functional molecules (a drug, a fluorescent tag and an organocatalyst), and we anticipate that a large variety of cargo molecules could be released with this device. This rotaxane actuator provides a versatile platform for various force-controlled release applications.

## Abstract (web summary)

A rotaxane-based mechanochemical system enables force-controlled release of multiple cargo molecules that are appended to its molecular axle.

## Main

**Fig. 1:**
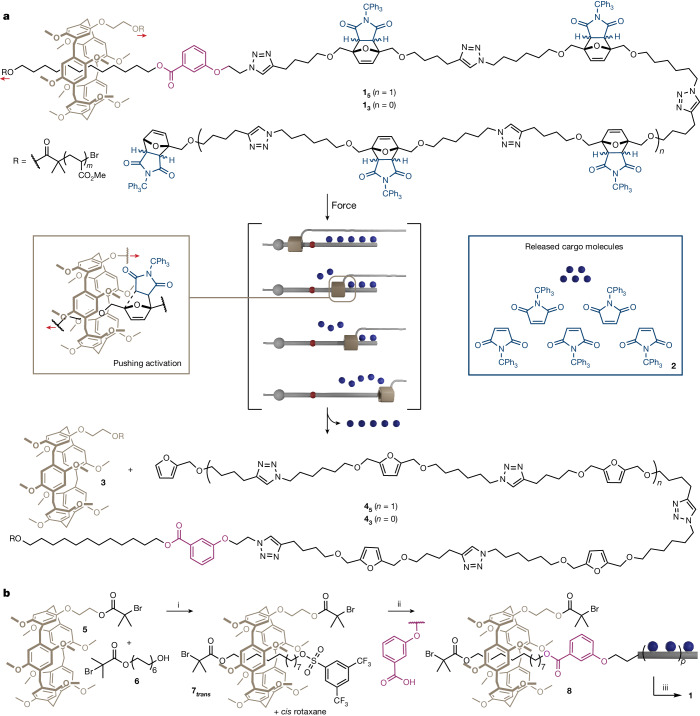
Elongation of the rotaxane actuator leads to the sequential release of the cargo units placed on the axle as they are pushed by the macrocycle. **a**, Design of rotaxane actuator **1**, able to release up to five cargo units per chain. The rotaxane is built around a pillar[5]arene macrocycle, which can trigger the release of *N*-triphenylmethyl maleimide (**2**) by promoting a mechanical retrocycloaddition when entering into contact with the furan/maleimide Diels–Alder adduct. **b**, Synthesis of cargo-bearing rotaxanes via a stopper exchange mechanism. Conditions: (i) BTBSCl, Et_3_N, CHCl_3_, −15 °C, 2 h, yield: 11%; (ii) K_2_CO_3_, 18-crown-6, acetone, room temperature, 16 h, yield: see Supplementary Information; (iii) methyl acrylate, Cu wire, CuBr_2_, Me_6_TREN, DMSO. Red arrows indicate the direction of the force.

**Extended Data Fig. 1:**
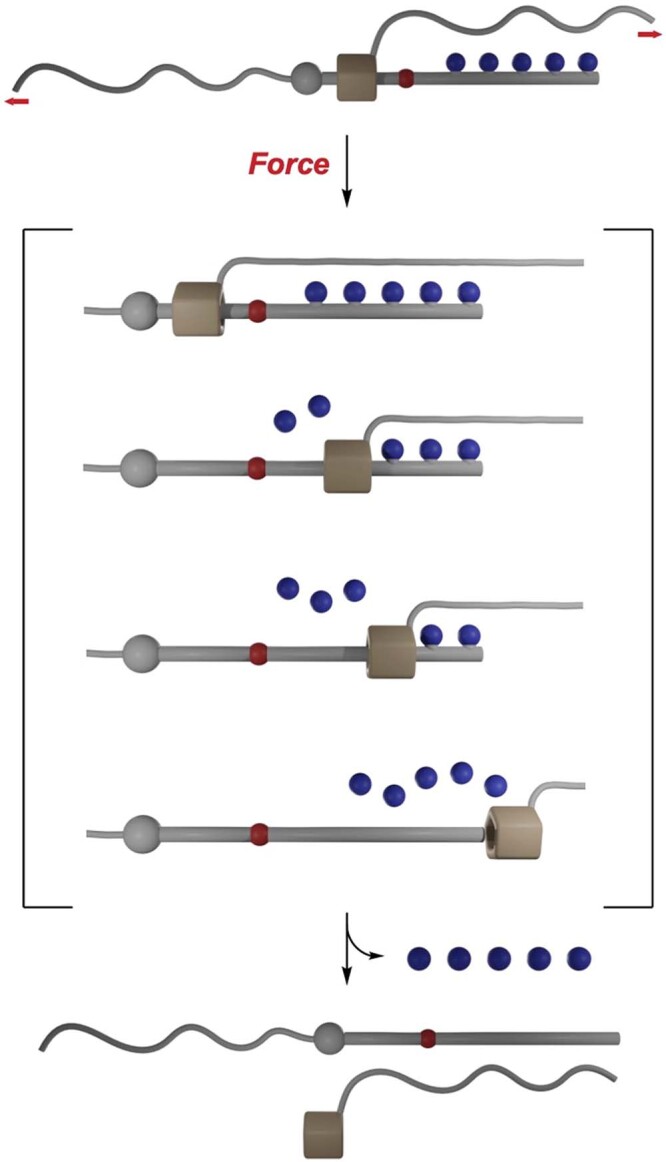
Cartoon depiction of the force-controlled release of cargo molecules by a rotaxane actuator. Upon elongation of the rotaxane, by the intermediary of the polymer chains (thin grey strands), the macrocycle (light brown) is pulled along the cargo compartment until it reaches the first cargo molecule (blue ball), which acts as a barrier as the macrocycle is unable to pass this steric obstacle without its detachment. Pulling the macrocycle further eventually triggers the release of the first cargo unit. This process is repeated as the macrocycle is pulled along the cargo compartment, leading to the release of all the cargo molecules and the escape of the macrocycle.

Interlocked molecules, such as rotaxanes, are well suited to act as force actuators due to their capability for large amplitude movements^17^. This property has been exploited to create non-scissile rotaxane-based force sensors^18–21^. We have recently demonstrated the ability of a rotaxane to influence the mechanochemical reactivity of a mechanophore embedded in its axle^22^, and shown how a rotaxane actuator can promote unstoppering reactions by enhancing the mechanical lability of covalent bonds in the axle^23^. At the same time, stimuli-responsive rotaxanes have been interfaced with polymers to perform various chemical tasks^24–27^. Building on these concepts, here we propose the use of a rotaxane architecture to release several small-molecule cargoes at once (Fig. 1a, Extended Data Fig. 1 and Supplementary Video 1). The rotaxane is mechanically activated by the intermediary of two polymer chains attached to the axle and the macrocycle, respectively (see “Design”). Elongational force will pull the macrocycle towards the cargo compartment, in which cargo molecules (blue balls; Fig. 1a) are dispersed along the axle. Forceful contact between the macrocycle and these steric obstacles leads to the sequential release of these cargo molecules via mechanochemical scission of the covalent bonds linking them to the axle.

## Design

Our design is based on a rotaxane architecture (**1**, Fig. 1a) built around a pillar[5]arene^28^ (P5) macrocycle threaded onto a C12 alkyl chain, extended on one side with a poly(methyl acrylate) (PMA) polymer and on the other side with the cargo-bearing oligomer (the cargo compartment). Another PMA chain is connected to the P5 macrocycle to enable the mechanical activation of this assembly^22,23^. This macrocycle was chosen for its rigid and tubular nature, as a more flexible macrocycle would favour the scission of the axle (unstoppering)^23^. The cargo units (*N*-triphenylmethyl maleimide, **2**, chosen for its steric bulk) were loaded onto the furan moieties of the cargo oligomer via a Diels–Alder reaction. On stretching, the macrocycle is pulled along the cargo compartment until it reaches the first Diels–Alder adduct, which acts as a barrier, as this small macrocycle is physically unable to pass this steric obstacle without the scission of a covalent bond (Fig. 1a). Pulling the macrocycle further against the adduct eventually triggers the retro-Diels–Alder reaction (Fig. 1a) that releases the cargo unit^29^. This geometry of activation, in which the mechanophore (the Diels–Alder adduct) is pushed rather than pulled (Fig. 1a, left box), is unique to the rotaxane architecture. This process is repeated as the macrocycle is pulled along the cargo compartment until it escapes (Fig. 1a). Up to five cargo units are released with rotaxane, **1**_**5**_.

## Synthesis

Chain-centred macromolecular rotaxanes were obtained by single-electron transfer living radical polymerization^30^ of methyl acrylate initiated from both the macrocycle and the axle of rotaxane **8** (Fig. 1b). This ensured that the rotaxane is placed in the central region of the chain, which experiences the largest intensity of force during sonication (see below)^22,23^. The rotaxane initiators were assembled following a stopper exchange strategy in which an activated rotaxane (**7**) is first produced by capping the inclusion complex formed between axle **6** and the P5 derivative **5** with a 3,5-bis(trifluoromethyl) benzenesulfonyl moiety (BTBS), which is also an excellent leaving group^31^. Substitution of BTBS by the carboxylic acid terminating the cargo compartment, assembled by iterative click chemistry (three- and five-cargo compartments present a small fraction (typically less than 6%) of unloaded furan units after assembly (Supplementary Information)), affords rotaxane initiator **8**.

## Model study

**Fig. 2:**
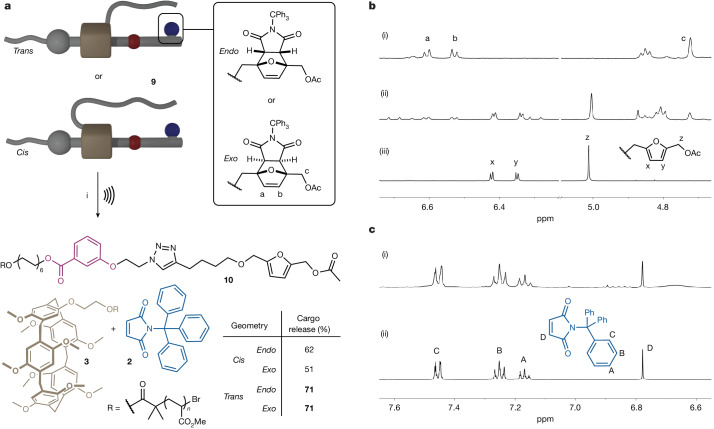
Mechanical activation of various geometrical isomers of model rotaxane 9. **a**, Mechanical activation of *cis* and *trans* isomers of model rotaxane **9** bearing *endo* or *exo* mechanophores. Conditions: (i) ultrasound (20 kHz, 13 W cm^−2^, 1 s on/1 s off), CH_3_CN, 5−10 °C, 300 min. **b**, Partial ^1^H NMR (400 MHz, acetone-d_6_) spectra of rotaxane **9**_*trans/exo*_ before (i) and after (ii) sonication, along with reference compound **S3** (iii), indicate activation of the Diels–Alder adduct and release of the maleimide cargo. **c**, Partial ^1^H NMR (400 MHz, acetone-d_6_) spectra of the post-sonication MeOH extract (i), along with a reference compound **2** (ii).

As the P5 macrocycle is cylindrical in shape^28^, the rotaxane formation results in two isomers in which the polymers are either on the same or opposite sides of the rotaxane, labelled as *cis* and *trans*, respectively. The identity was confirmed by rotating-frame nuclear Overhauser effect spectroscopy (Supplementary Information section 4.3.) Similarly, the Diels–Alder reaction linking the cargo to the axle can produce both *endo* and *exo* isomers of the adduct (Supplementary Information). As the geometry of these mechanophores can greatly affect their mechanochemical reactivity^29,32^, we decided to explore the influence of these two sources of isomerism before assembling a multicargo rotaxane device (Fig. 2). The four possible isomers of rotaxane **9** (number average molecular weight *M*_*n*_ = 92–114 kDa, dispersity *Đ* = 1.12–1.17; see Supplementary Information section 5.11 for details), which contains a single cargo unit, were mechanically activated by ultrasonication (Fig. 2a), a technique in which elongational flows are generated in the vicinity of collapsing cavitation bubbles^33^. The progress of the reaction was monitored by size-exclusion chromatography (Supplementary Section 6.3) and the efficiency of the cargo release was determined by ^1^H NMR spectroscopy, by comparing the integration of diagnostic peaks of the Diels–Alder adduct (peaks a, b, c; Fig. 2b) and those of the furan unit revealed after the release of the maleimide cargo (peaks x, y, z; Fig. 2b). The *trans* isomer of rotaxane **9** proved to be the better actuator, activating both the *endo* and *exo* adducts with the same efficiency (71% conversion; Fig. 2a), so we decided to proceed with this isomer for the assembly of larger structures. The cargo molecules can be recovered by extracting the post-sonication polymer residue with MeOH (Supplementary Information section 6). Analysis of this extract by ^1^H NMR spectroscopy confirms the release of the cargo, notably with the presence of the maleimide olefinic peak (peak D; Fig. 2c). The liberation of the macrocycle from the axle after the release of the cargo unit was also confirmed by ^1^H NMR spectroscopy (Supplementary Information section 6.5).

## Scope of cargo release

**Fig. 3:**
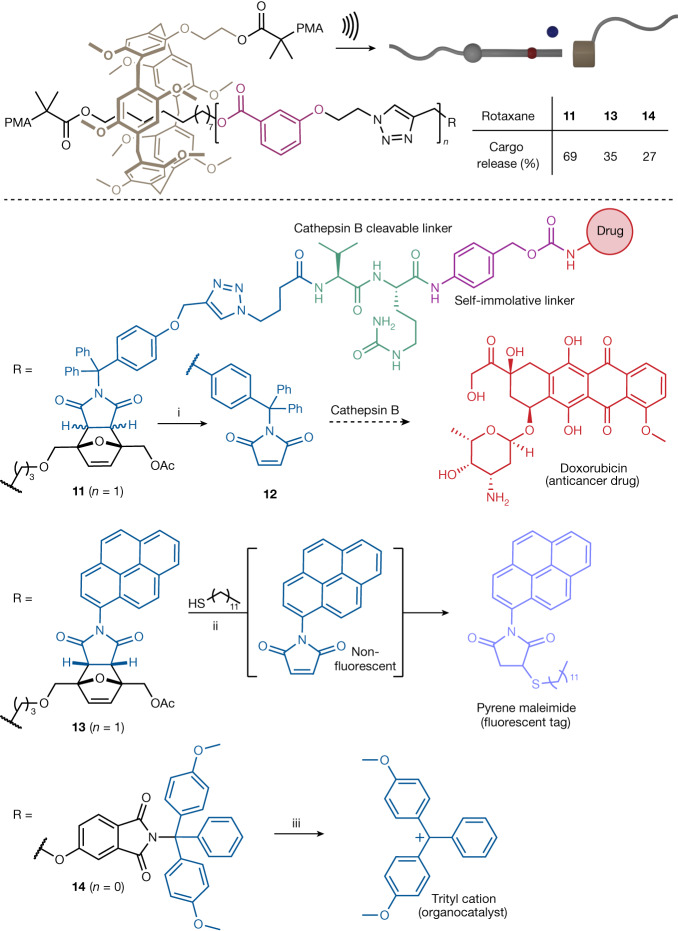
Force-controlled release of functional cargo molecules via retrocycloaddition and heterolytic cleavage. Conditions: ultrasound (20 kHz, 13 W cm^−2^, 1 s on/1 s off), 5−10 °C and (i) CH_3_CN, 90 min or (ii) 1-dodecane thiol (50 equiv.), CH_3_CN/H_2_O: 9/1, 90 min or (iii) THF/H_2_O: 75/1, 120 min.

We then sought to explore the ability of our rotaxane actuator to release functional molecules (Fig. 3). We chose three model cargo molecules (a drug, a fluorescent tag and an organocatalyst), which are representative of the potential biomedical (drug release, molecular tagging) and materials (damage reporting, self-healing) applications for such a force-controlled release device. In the first case, we appended our bulky maleimide cargo unit with a valine–citrulline peptide linker, which connects to the drug via a self-immolative *para*-aminobenzyloxycarbonyl spacer. This architecture is commonly used in antibody–drug conjugates as it relies on the overexpression of cathepsin B in cancerous cells, a lysosomal cysteine protease, to trigger the release cascade^34^. As a proof-of-concept we chose to load the cargo with doxorubicin, a potent wide-spectrum chemotherapeutic agent^35^, which is released with an efficiency of 65% in solution. We selected *N*-(1-pyrenyl)maleimide, a popular fluorescent probe^36^, as a molecular tag to demonstrate that the rotaxane actuator can accommodate cargoes of different size and shape. A model thiol (dodecane thiol) was tagged in situ on mechanical release of non-fluorescent *N*-(1-pyrenyl)maleimide to form the corresponding fluorescent adduct. Finally, the release of the trityl cation, a mild Lewis acid organocatalyst known to catalyse cycloadditions and rearrangements^37^, further illustrates the versatility of the rotaxane actuation to deliver varied cargo molecules through diverse dissociation mechanisms.

## Activation of multicargo rotaxanes in solution

**Fig. 4:**
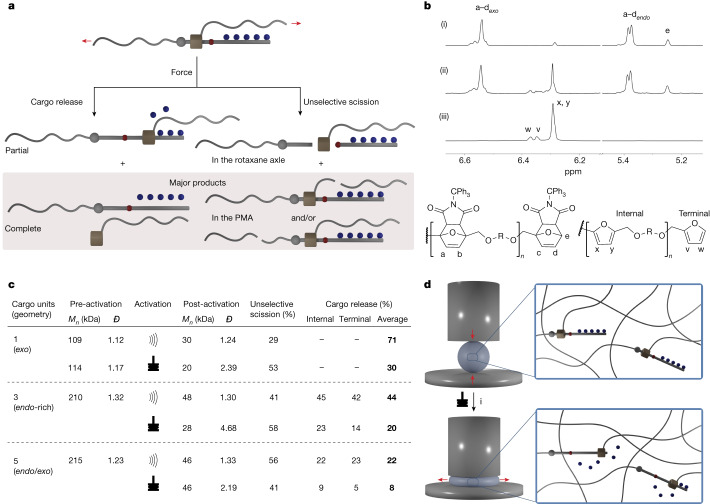
Activation of multicargo rotaxanes in solution and bulk. **a**, Mechanical activation of multicargo rotaxanes can lead to partial or complete release of the cargo load. Unselective scission can occur if the rotaxane breaks in the axle or in one of the PMA chains. **b**, Partial ^1^H NMR (400 MHz, acetone-d_6_) spectra of rotaxane **1**_**5-215**_ before (i) and after (ii) sonication, along with reference compound **S22** (iii), indicate the activation of internal and terminal Diels–Alder adducts. **c**, Structural and activation parameters for representative one-, three- and five-cargo rotaxanes. Percentage of unselective scission combines PMA and axle scissions. Relative error of unselective scission and cargo release, 17–51%, see Supplementary Section 8.7 for full data and calculation details. **d**, Activation by compression in bulk leads to cargo release in an entangled network. Condition: (i) manual press (0.74 GPa, less than 60 min per cycle, 10–45 cycles).

We performed the mechanical activation of rotaxanes **1**_**5**_ and **1**_**3**_ (Fig. 1b) loaded with five and three cargo units, respectively, in solution by ultrasonication (Fig. 4a–c). The mechanical behaviour of these longer systems is more complex, as the macrocycle can reach the end of the cargo compartment or potentially stop somewhere along the way (for example, due to competing bond scission; see below), leading to complete or partial release of the cargo load, respectively (Fig. 4a). Competing bond scission in one of the PMA chains, or in the rotaxane axle (unstoppering)^23^, could also occur at any stage; in both cases, no more cargo molecules can be released as the rotaxane is no longer in the central region of the polymer, or the interlocked architecture is lost altogether (Fig. 4a). Dilute solutions of these polymers were sonicated until at least one bond (covalent or mechanical) scission had occurred in the main chain (that is, until the observed *M*_*n*_ was below half of the initial *M*_*n*_; Supplementary Information section 6). Unselective cleavage in the PMA chain, determined from the amount of intact rotaxane left after sonication (Supplementary Information section 8), proved to be the major pathway for these longer rotaxanes (typically less than 40% and less than 50% for three- and five-cargo rotaxanes, respectively; Fig. 4c and Supplementary Information section 8.7), whereas cleavage of the axle is a very minor pathway (typically less than 5%; Supplementary Information section 8.7). The rest of the rotaxanes engage in the cargo-release process. As it is possible to distinguish between the internal and terminal Diels–Alder adducts/furan groups by ^1^H NMR spectroscopy (Fig. 4b), we were able to determine the extent of cargo release at these positions separately. Their relative integration (Fig. 4c) shows that most macrocycles engaging in cargo release (that is, not experiencing unselective scission; left path in Fig. 4a) are able to reach the end of the cargo compartment and deliver the entire load, with a release efficiency of up to 44% and 22% for three- and five-cargo rotaxanes, respectively (Fig. 4c). The release efficiency shows an apparent decrease as the length of the cargo compartment increases, but, as the macrocycle is not observed to stop midway (as discussed above), it is likely that the decrease in efficiency is not an inherent limitation of the rotaxane actuator; this is possibly due to the formation of less defined polymers when the polymerization is initiated from such large molecules (Fig. 4c). In this case, the rotaxane is less likely to be located at the centre of the chain, which experiences the highest intensity of force during sonication, and the unselective scission of a PMA chain becomes predominant^38^. We also explored the effect of the polymer length (*M*_*n*_ = 60–215 kDa) on the activation of the five-cargo rotaxane and the influence of the *exo*/*endo* content (*exo*-rich to *endo*-rich) on the three-cargo rotaxane (Supplementary Information section 8.7). We found no influence of the polymer length on the efficiency of cargo release, but the *endo*-rich rotaxane shows a better conversion (44%) than its *exo*-rich counterpart (29%).

## Activation in bulk

The bulk activation of one-, three- or five-cargo rotaxanes was performed by compressing a small sample (approximately 30 mg) of polymers **9**, **1**_**3**_ and **1**_**5**_, respectively, using a manual press (0.74 GPa, less than 60 min per cycle, 10–45 cycles; Fig. 4c,d). Despite the lack of covalent crosslinks, which would lead to a greater activation^39^, a substantial amount of cargo release was observed in these entangled networks (a non-interlocked control polymer confirmed the mechanical nature of the activation; Supplementary Information section 7.2), although to a lower extent than in solution at similar post-activation *M*_*n*_ (Fig. 4c). Here again, the cargo molecules could be recovered by extracting the post-compression polymer residue with MeOH (Supplementary Information section 7). The bulk activation contrasts with the solution experiments by the lower proportion of rotaxanes releasing their entire cargo load; that is to say, fewer macrocycles can reach the end of their cargo compartment. This can be explained by the ability of the network to distribute tensional stress, and the fact that the rotaxanes could be located in a low stress region of the network. Nevertheless, the possibility of releasing up to 30% of the cargo load across the sample (with the one-cargo system) or up to five cargo molecules in the same location (with the five-cargo system), places our systems among the best for covalent force-controlled release molecular mechanisms^1^ and offers great promise for the release of active compounds in a variety of contexts.

## Conclusions

We have demonstrated the force-controlled release of small molecules using a rotaxane actuator in which the force-induced movement of the macrocycle along the axle leads to the activation and subsequent release of small-molecule cargo in series. The rotaxane architecture enables the iterative actuation of scissile mechanophores because the actuating polymers are not directly attached to the mechanophore. This architecture is also unique in the way it activates mechanophores, with a pushing rather than a pulling geometry. We have shown that such a molecular device can release up to five cargo units per chain both in solution and in bulk. An activation efficiency of up to 30% has been achieved in bulk, which places our device among the best covalent force-controlled release systems achieved so far. Furthermore, we have demonstrated the versatility of this actuating device by releasing three representative functional molecules (a drug, a fluorescent tag and an organocatalyst) and we anticipate that a larger diversity of cargo could be released. Additionally, such a system offers the possibility to release different cargo units in a defined sequence. The versatility and efficiency of the rotaxane actuator should pave the way to more sophisticated force-controlled release systems.

## Methods

See Supplementary Information for detailed methods and protocols.

### Mechanical activation in solution by ultrasonication

The appropriate polymer (20 mg) was added to a Suslick cell and dissolved in dry MeCN (15 ml). The solution was degassed by bubbling N_2_ through it for a minimum of 10 min before the start of sonication and throughout the experiment. The Suslick cell was cooled with an ice bath throughout the duration of the sonication to maintain a temperature of approximately 5–10 °C inside the cell. Pulsed ultrasound was applied to the system (1 s on/1 s off, 25% amplitude (13.0 W cm^−2^), 20 kHz) for the desired period of time. After sonication, the solvent was evaporated and the polymer was analysed by size-exclusion chromatography and NMR. The post-sonication polymer was recovered and washed with MeOH to extract any small molecules not attached to polymer chains. The remaining MeOH-washed polymer and the concentrated MeOH washings were then analysed by NMR.

### Mechanical activation in bulk by compression

The appropriate polymer (25–30 mg) was formed into a rough spherical shape by hand. The material was placed in between the anvils of a standard 13 mm KBr pellet die. A compressive force of 10 tonnes was then applied; as the material was compressed, the pressure was relieved gradually by rearrangement of the material so, over the course of an hour, it was ensured that 10 tonnes of force was being continuously applied. The pressure was then released and the flattened material refolded into a new sphere. This cyclical process of folding followed by compression over an hour was repeated until size-exclusion chromatography analysis of the material showed adequate reduction in the *M*_*n*_. At this point, the material was dissolved in dichloromethane and carefully filtered (0.45 μm polytetrafluoroethylene membrane) to remove any metal particulates before being condensed in vacuo. The crude polymer material was analysed by ^1^H NMR before being thoroughly dried and the polymer film being directly washed over with MeOH. The MeOH washings were collected and analysed by ^1^H NMR along with the washed polymer material itself.

## Online content

Any methods, additional references, Nature Portfolio reporting summaries, source data, extended data, supplementary information, acknowledgements, peer review information; details of author contributions and competing interests; and statements of data and code availability are available at 10.1038/s41586-024-07154-0.

### Supplementary information

Supplementary Information Experimental procedures, methods and characterization data.

Supplementary Video 1 Animation of the force-controlled release of cargo molecules by a rotaxane actuator. On elongation of the rotaxane, by the intermediary of the polymer chains (thin grey strands), the macrocycle (light brown) is pulled along the cargo compartment until it reaches the first cargo molecule (blue ball), which acts as a barrier as the macrocycle is unable to pass this steric obstacle without its detachment. Pulling the macrocycle further eventually triggers release of the first cargo unit. This process is repeated as the macrocycle is pulled along the cargo compartment, leading to release of all the cargo molecules and escape of the macrocycle.

## Data Availability

The data that support the finding of this study are available within the paper and its Supplementary Information, or are available from the figshare data repository (https://figshare.com) under 10.6084/m9.figshare.25053494.
