# Supplementary material for: Force-controlled release of small molecules with a rotaxane actuator
Source: Nature. 2024 Apr 10;628(8007):320–5. doi: 10.1038/s41586-024-07154-0 (PMC11006608; doi:10.1038/s41586-024-07154-0)
Supplement: Supplementary file 1 — Experimental procedures, methods and characterization data. [file 41586_2024_7154_MOESM1_ESM.pdf]

nature portfolio

https://doi.org/10.1038/s41586-024-07154-0

Supplementary information

Force-controlled release of small molecules with a rotaxane actuator

In the format provided by the authors and unedited

Force-controlled release of small molecules with a rotaxane actuator

*Lei Chen, Robert Nixon, and Guillaume De Bo**

Department of Chemistry, University of Manchester, Oxford Road, Manchester, M13 9PL, UK

*Email: guillaume.debo@manchester.ac.uk

1 Table of Contents and Indexes

1.1 Table of Contents

1 Table of Contents and Indexes 2
1.1 Table of Contents 2
2 General Experimental Details 9
3 Nomenclature of Multi-Cargo-Containing Compounds and Polymers 10
4 Synthesis of Rotaxanes and Reference compounds 12
4.1 Synthesis of 1-, 3-, and 5-Cargo Diels-Alder Cargo Compartments 12
4.1.1 Synthetic Route to *Endo* and *Exo* 1-Cargo Diels-Alder Cargo Compartments 12
4.1.2 Synthesis of 2 12
4.1.3 Synthesis of S1 12
4.1.4 Synthesis of S2 13
4.1.5 Synthesis of S3 13
4.1.6 Synthesis of S4 14
4.1.7 Synthetic Route to Attachment of Carboxylic Acid Linker to 1-Cargo Diels-Alder Cargo Compartments 15
4.1.8 Synthesis of S5 15
4.1.9 Synthesis of S6 15
4.1.10 Synthesis of S7 16
4.1.11 Synthesis of $S8_{exo}$ 16
4.1.12 Synthesis of $S8_{endo}$ 17
4.1.13 Synthetic Route to 3- and 5-Cargo Unloaded Cargo Compartments 18
4.1.14 Synthesis of S9 18
4.1.15 Synthesis of S10 19
4.1.16 Synthesis of S11 19
4.1.17 Synthesis of S12 20
4.1.18 Synthesis of S13 20
4.1.19 Synthesis of S14 21
4.1.20 Synthesis of S15 22
4.1.21 Synthesis of S16 23
4.1.22 Synthetic Routes to 3-Cargo Diels-Alder Cargo Compartments 24
4.1.23 Synthesis of S17 24
4.1.24 Synthesis of S18a, S18b, and S18c 27
4.1.25 Synthesis of S19 29
4.1.26 Synthesis of S18d 30
4.1.27 Synthetic Route to 5-Cargo Diels-Alder Cargo Compartments 31
4.1.28 Synthesis of S20 32
4.1.29 Synthesis of S21 33
4.1.30 Synthesis of Reference Five-Cargo Unloaded Cargo Compartment, S22 35
4.2 Synthesis of Rotaxane Precursors 36
4.2.30 Synthetic Route to *cis*- and *trans*-Rotaxane Precursors 36
4.2.31 Synthesis of 5 36
4.2.32 Synthesis of 6 37
4.2.33 Synthesis of 7 and S24 37
4.3 Determination of the *cis/trans* Isomerism of Rotaxane Precursors 7 39
4.3.1 Synthesis of $S25_{trans}$ 40
4.3.2 Synthesis of $S25_{cis}$ 41
4.4 Synthesis of Control Compounds, S26 42
4.4.1 Synthesis of $S26_{exo}$ 42
4.4.1 Synthesis of $S26_{endo}$ 42

4.5 Synthesis of 1-Cargo Rotaxanes ... 44
4.5.1 Synthesis of S27$_{trans/exo}$ ... 44
4.5.2 Synthesis of S27$_{cis/exo}$ ... 45
4.5.3 Synthesis of S27$_{trans/endo}$ ... 45
4.5.4 Synthesis of S27$_{cis/endo}$ ... 46
4.6 Synthesis of 3-Cargo Rotaxanes ... 47
4.6.1 Synthesis of 8_3 ... 47
4.7 Synthesis of 5-Cargo Rotaxane ... 51
4.7.1 Synthesis of 8_5 ... 51
4.8 Synthesis of Alternative-Cargo Compounds – Drug-Containing Cargo Release ... 53
4.8.1 Synthetic Route to Rotaxane With Linker-Containing Cargo ... 53
4.8.2 Synthesis of S29 ... 53
4.8.3 Synthesis of S30 ... 54
4.8.4 Synthesis of S31 ... 54
4.8.5 Synthesis of S32 ... 55
4.8.6 Synthesis of S33 ... 56
4.8.7 Synthetic Route to Val-Cit Dipeptide Linker, S36 ... 57
4.8.8 Synthesis of S35 ... 57
4.8.9 Synthesis of S36 ... 58
4.8.10 Synthetic Route to Reference Compound, 12 ... 59
4.8.11 Synthesis of S37 ... 59
4.8.12 Synthesis of S38 ... 60
4.8.13 Synthesis of 12 ... 61
4.9 Synthesis of Alternative-Cargo Compounds – N-(1-pyrenyl)maleimide Cargo Release ... 62
4.9.1 Synthetic Route to N-(1-pyrenyl)maleimide-Cargo-Containing Rotaxane ... 62
4.9.2 Synthesis of S39 ... 62
4.9.3 Synthesis of S40 ... 63
4.9.4 Synthesis of S41 ... 64
4.9.5 Synthesis of Control Compound, S42 ... 65
4.9.6 Synthesis of Reference Compound, S43 ... 66
4.9.7 Synthetic Route to Reference Compound, S45 ... 66
4.9.8 Synthesis of S44 ... 67
4.9.9 Synthesis of S45 ... 67
4.10 Synthesis of Alternative-Cargo Compounds – Trityl Cargo Release ... 69
4.10.1 Synthetic Route to Trityl-Cargo-Containing Rotaxane ... 69
4.10.2 Synthesis of S46 ... 69
4.10.3 Synthesis of S47 ... 70
4.10.4 Synthesis of S48 ... 70
4.10.5 Synthesis of S49 ... 71
4.10.6 Synthetic Route to Trityl-Cargo Control Compound ... 72
4.10.7 Synthesis of S50 ... 72
4.10.8 Synthesis of S51 ... 72
4.10.9 Synthesis of S52 ... 73
4.10.10 Synthesis of Reference Compound, S54 ... 73
5 Synthesis of Polymers ... 75
5.1 Representative Procedure for SET-LRP of Methyl Acrylate Using Mechanophore Initiators ... 75
5.2 Synthesis of Control Polymers ... 75
5.2.1 Synthesis of Polymer S55$_{exo\text{-}112}$ ... 75
5.2.2 Synthesis of Polymer S55$_{endo\text{-}65}$ ... 75
5.3 Synthesis of 1-Cargo Polymers ... 76
5.3.1 Synthesis of Polymer $9_{trans/exo\text{-}109}$ ... 76
5.3.2 Synthesis of Polymer $9_{trans/exo\text{-}114}$ ... 76
5.3.3 Synthesis of Polymer $9_{cis/exo\text{-}114}$ ... 76
5.3.4 Synthesis of Polymer $9_{trans/endo\text{-}90}$ ... 77

5.3.5 Synthesis of Polymer $9_{cis/endo\text{-}92}$ 77
5.4 Synthesis of 3-Cargo Polymers 78
5.4.1 Synthesis of Polymer 1_3a_{-210} 78
5.4.2 Synthesis of Polymers 1_3b 78
5.4.3 Synthesis of Polymer 1_3c_{-174} 78
5.4.4 Synthesis of Polymer 1_3d_{-89} 79
5.5 Synthesis of 5-Cargo Polymers 79
5.5.1 Synthesis of Polymers 1_5 79
5.6 Synthesis of Reference Polymers 80
5.6.1 Synthesis of Polymer 3_{ref} 80
5.7 Synthesis of Matrix Polymer, $S56_{-153}$ 80
5.8 Synthesis of Alternative Cargo Polymers – Drug-Containing-Cargo Release 81
5.8.1 Synthetic Route to Drug-Containing-Cargo Polymer, 11_{-127} 81
5.8.2 Synthesis of Polymer $S57_{-108}$ 82
5.8.3 Synthesis of Polymer $S58_{-122}$ 82
5.8.4 Synthesis of Polymer 11_{-127} 83
5.9 Synthesis of Alternative-Cargo Polymers – N-(1-pyrenyl)maleimide Cargo Release 83
5.9.1 Synthesis of N-(1-pyrenyl)maleimide-Cargo Polymer, 13_{-119} 83
5.9.2 Synthesis of N-(1-pyrenyl)maleimide-Cargo Control Polymer, $S59_{-77}$ 84
5.9.3 Synthesis of N-(1-pyrenyl)maleimide-Cargo Reference Polymer, 10_{-80} 84
5.10 Synthesis of Alternative-Cargo Polymers – Trityl Cargo Release 85
5.10.1 Synthesis of Trityl-Cargo Polymer, 14_{-124} 85
5.10.2 Synthesis of Trityl-Cargo Control Polymer, $S60_{-95}$ 85
5.10.3 Synthesis of Trityl-Cargo Reference Polymer, $S61_{-72}$ 85
5.11 SEC Data for Synthesised Polymers 86
5.12 SEC Traces for Control Polymers, S55 87
5.13 SEC Traces for 1-Cargo Polymers, 9 88
5.14 SEC Traces for 3-Cargo Polymers, 1_3 89
5.15 SEC Traces for 5-Cargo Polymers, 1_5 90
5.16 SEC Traces for Reference Polymer, 3_{ref} 90
5.17 SEC Trace of Matrix Polymer, S56 91
5.18 SEC Traces of Alternative Cargo Polymers – Drug-Containing-Cargo Release 91
5.19 SEC Traces of Alternative Cargo Polymers – N-(1-pyrenyl)maleimide-Cargo Release 92
5.20 SEC Traces of Alternative Cargo Polymers – Trityl-Cargo Release 93
6 Mechanophore Activation via Ultrasound 94
6.1 General Procedure for Sonication Experiments 94
6.2 Sonication of Control Polymers, S55 94
6.3 Sonication of 1-Cargo Polymers, 9 97
6.4 Sonication of 3-Cargo Polymers, 1_3, and 5-Cargo Polymers, 1_5 102
6.5 Dethreading of the Macrocycle after Complete Cargo Release 105
6.6 Sonication of Alternative-Cargo Polymers – Drug-Containing-Cargo Release 106
6.7 Sonication of Alternative-Cargo Polymers – N-(1-pyrenyl)maleimide-Cargo Release 108
6.7.1 Sonication of Polymer 13_{-119} in the Presence of 1-Dodecanethiol 111
6.7.2 Sonication of Control Polymer $S59_{-77}$ 112
6.8 Sonication of Alternative-Cargo Polymers – Trityl-Cargo Release 113
6.8.1 Sonication of Control Polymer, $S60_{-95}$ 114
7 Activation in Bulk by Compression 115
7.1 General Procedure for Compression Experiments 115
7.2 Control Experiment for Activation in Bulk 115
7.3 Bulk Activation of 1-, 3-, and 5-Cargo Polymers 117
7.4 Bulk Activation Using PMA Matrix 121
8 Calculation of Extent of Mechanophore Activation 122
8.1 Co-conformational Isomerism in Cargo-loaded Rotaxanes 122
8.2 Calculations for 1-Cargo Mechanophores with No Unstoppering 124

8.3 Calculations for 1-Cargo Mechanophores with Unstoppering 126
8.4 Calculations for 3- and 5-Cargo Mechanophores 128
8.5 Calculations for N-(1-pyrenyl)maleimide-Cargo Polymer, $13_{\text{-}119}$ 130
8.6 Calculations for Trityl-Cargo Polymer, $14_{\text{-}124}$ 131
8.7 Summary of Mechanophores Activated by Sonication 133
8.8 Summary of Alternative-Cargo Mechanophores Activated by Sonication 134
8.9 Summary of Mechanophores Activated in the Solid-State 135
9 NMR Spectra 136
9.1 Small Molecule NMR Spectra 136
9.1.1 Spectra of 2 136
9.1.2 Spectra of S1 137
9.1.3 Spectra of S2 138
9.1.4 Spectra of S3 139
9.1.5 Spectra of $S4_{exo}$ 140
9.1.6 Spectra of $S4_{endo}$ 141
9.1.7 Spectra of S5 142
9.1.8 Spectra of S6 143
9.1.9 Spectra of S7 144
9.1.10 Spectra of $S8_{exo}$ 145
9.1.11 Spectra of $S8_{endo}$ 146
9.1.12 Spectra of S9 147
9.1.13 Spectra of S10 148
9.1.14 Spectra of S11 149
9.1.15 Spectra of S12 150
9.1.16 Spectra of S13 151
9.1.17 Spectra of S14 152
9.1.18 Spectra of S15 153
9.1.19 Spectra of S16 154
9.1.20 Spectra of S17a 155
9.1.21 Spectra of S17b 156
9.1.22 Spectra of S17c 157
9.1.23 Spectra of S18a 158
9.1.24 Spectra of S18b 159
9.1.25 Spectra of S18c 160
9.1.26 Spectra of S18d 161
9.1.27 Spectra of S19 162
9.1.28 Spectra of S20 163
9.1.29 Spectra of S21 164
9.1.30 Spectra of S22 165
9.1.31 Spectra of 5 166
9.1.32 Spectra of 6 167
9.1.33 Spectra of 7_{trans} 168
9.1.34 Spectra of 7_{cis} 170
9.1.35 Spectra of S24 172
9.1.36 Spectra of $S25_{trans}$ 174
9.1.37 Spectra of $S25_{cis}$ 177
9.1.38 Spectra of $S26_{exo}$ 180
9.1.39 Spectra of $S26_{endo}$ 181
9.1.40 Spectra of $S27_{trans/exo}$ 182
9.1.41 Spectra of $S27_{cis/exo}$ 185
9.1.42 Spectra of $S27_{trans/endo}$ 186
9.1.43 Spectra of $S27_{cis/endo}$ 187
9.1.44 Spectra of 8_3a 188
9.1.45 Spectra of 8_3b 189

9.1.46 Spectra of 8_3c ... 190
9.1.47 Spectra of 8_3d ... 191
9.1.48 Spectra of 8_5 ... 192
9.1.49 Spectra of S29 ... 193
9.1.50 Spectra of S30 ... 194
9.1.51 Spectra of S31 ... 195
9.1.52 Spectra of S32 ... 196
9.1.53 Spectra of S33 ... 197
9.1.54 Spectra of S35 ... 198
9.1.55 Spectra of S36 ... 199
9.1.56 Spectra of S37 ... 200
9.1.57 Spectra of S38 ... 201
9.1.58 Spectra of 12 ... 202
9.1.59 Spectra of S39 ... 203
9.1.60 Spectra of S40 ... 204
9.1.61 Spectra of S41 ... 205
9.1.62 Spectra of S42 ... 206
9.1.63 Spectra of S43 ... 207
9.1.64 Spectra of S44 ... 208
9.1.65 Spectra of S45 ... 209
9.1.66 Spectra of S46 ... 210
9.1.67 Spectra of S47 ... 211
9.1.68 Spectra of S48 ... 212
9.1.69 Spectra of S49 ... 213
9.1.70 Spectra of S50 ... 214
9.1.71 Spectra of S51 ... 215
9.1.72 Spectra of S52 ... 216
9.1.73 Spectra of S54 ... 217
9.2 Polymer NMR Spectra ... 218
9.2.1 Spectra of polymer $S55_{\textit{exo}\text{-}112}$ ... 218
9.2.2 Spectra of polymer $S55_{\textit{endo}\text{-}65}$ ... 219
9.2.3 Spectra of polymer $9_{\textit{trans/exo}\text{-}109}$ ... 220
9.2.4 Spectra of polymer $9_{\textit{trans/exo}\text{-}114}$ ... 221
9.2.5 Spectra of polymer $9_{\textit{cis/exo}\text{-}114}$ ... 222
9.2.6 Spectra of polymer $9_{\textit{trans/endo}\text{-}90}$ ... 223
9.2.7 Spectra of polymer $9_{\textit{cis/endo}\text{-}92}$ ... 224
9.2.8 Spectra of polymer 1_3a_{-210} ... 225
9.2.9 Spectra of polymer 1_3b_{-142} ... 226
9.2.10 Spectra of polymer 1_3b_{-171} ... 227
9.2.11 Spectra of polymer 1_3b_{-178} ... 228
9.2.12 Spectra of polymer 1_3c_{-174} ... 229
9.2.13 Spectra of polymer 1_3d_{-89} ... 230
9.2.14 Spectra of polymer $1_{5\text{-}60}$ ... 231
9.2.15 Spectra of polymer $1_{5\text{-}165}$ ... 232
9.2.16 Spectra of polymer $1_{5\text{-}215}$ ... 233
9.2.17 Spectra of polymer 3_{ref} ... 234
9.2.18 Spectra of polymer $S57_{-108}$ ... 235
9.2.19 Spectra of polymer $S58_{-122}$ ... 236
9.2.20 Spectra of polymer 11_{-127} ... 237
9.2.21 Spectra of polymer 13_{-119} ... 238
9.2.22 Spectra of polymer $S59_{-77}$ ... 239
9.2.23 Spectra of polymer 10_{-80} ... 240
9.2.24 Spectra of polymer 14_{-124} ... 241
9.2.25 Spectra of polymer $S60_{-95}$ ... 242

9.2.26 Spectra of polymer S61$_{-72}$ 243
9.3 Post-Sonication NMR Spectra 244
9.3.1 Post-Sonication ^{1}H NMR Spectra of Polymer S55$_{exo\text{-}112}$ 244
9.3.2 Post-Sonication ^{1}H NMR Spectra of Polymer S55$_{endo\text{-}65}$ 246
9.3.3 Post-Sonication ^{1}H NMR Spectra of Polymer 9$_{trans/exo\text{-}109}$ 248
9.3.4 Post-Sonication ^{1}H NMR Spectra of Polymer 9$_{cis/exo\text{-}114}$ 250
9.3.5 Post-Sonication ^{1}H NMR Spectra of Polymer 9$_{trans/endo\text{-}90}$ 252
9.3.6 Post-Sonication ^{1}H NMR Spectra of Polymer 9$_{cis/endo\text{-}92}$ 254
9.3.7 Post-Sonication ^{1}H NMR Spectra of Polymer 1$_3$d$_{-89}$ 256
9.3.8 Post-Sonication ^{1}H NMR Spectra of Polymer 1$_3$a$_{-210}$ 258
9.3.9 Post-Sonication ^{1}H NMR Spectra of Polymer 1$_3$b$_{-142}$ 260
9.3.10 Post-Sonication ^{1}H NMR Spectra of Polymer 1$_3$b$_{-171}$ 262
9.3.11 Post-Sonication ^{1}H NMR Spectra of Polymer 1$_3$b$_{-178}$ 264
9.3.12 Post-Sonication ^{1}H NMR Spectra of Polymer 1$_3$c$_{-174}$ 266
9.3.13 Post-Sonication ^{1}H NMR Spectra of Polymer 1$_{5\text{-}60}$ 268
9.3.14 Post-Sonication ^{1}H NMR Spectra of Polymer 1$_{5\text{-}165}$ 270
9.3.15 Post-Sonication ^{1}H NMR Spectra of Polymer 1$_{5\text{-}215}$ 272
9.3.16 Post-Sonication ^{1}H NMR Spectra of Polymer 11$_{-127}$ 274
9.3.17 Post-Sonication ^{1}H NMR Spectra of Polymer 13$_{-119}$ 276
9.3.18 Post-Sonication ^{1}H NMR Spectra of Polymer S59$_{-77}$ 280
9.3.19 Post-Sonication ^{1}H NMR Spectra of Polymer 14$_{-124}$ 282
9.3.20 Post-Sonication ^{1}H NMR Spectra of Polymer S60$_{-95}$ 284
9.4 Post-Solid-State-Activation NMR Spectra 286
9.4.1 Post-Solid-State-Activation ^{1}H NMR of Control Polymer S55$_{exo\text{-}112}$ 286
9.4.2 Post-Solid-State-Activation ^{1}H NMR of Polymer 9$_{trans/exo\text{-}114}$ 287
9.4.3 Post-Solid-State-Activation ^{1}H NMR of 1$_3$a$_{-210}$ 289
9.4.4 Post-Solid-State-Activation ^{1}H NMR of Polymer 1$_{5\text{-}165}$ 291
9.4.5 Post-Solid-State-Activation ^{1}H NMR of Polymer 1$_{5\text{-}215}$ 293
9.4.6 Post-Solid-State-Activation ^{1}H NMR of Polymer blend S30 295
10 Mass Spectrometry Isotopic Patterns 297
10.1 Isotopic distribution of S15 297
10.2 Isotopic distribution of S16 297
10.3 Isotopic distribution of S17a 298
10.4 Isotopic distribution of S17b 298
10.5 Isotopic distribution of S17c 299
10.6 Isotopic distribution of S18a 299
10.7 Isotopic distribution of S18b 300
10.8 Isotopic distribution of S18c 300
10.9 Isotopic distribution of S18d 301
10.10 Isotopic distribution of S19 301
10.11 Isotopic distribution of S20 302
10.12 Isotopic distribution of S21 302
10.13 Isotopic distribution of S22 303
10.14 Isotopic distribution of 5 303
10.15 Isotopic distribution of 7$_{trans}$ 304
10.16 Isotopic distribution of 7$_{cis}$ 304
10.17 Isotopic distribution of S25$_{trans}$ 305
10.18 Isotopic distribution of S25$_{cis}$ 305
10.19 Isotopic distribution of S26$_{exo}$ 306
10.20 Isotopic distribution of S26$_{endo}$ 306
10.21 Isotopic distribution of S27$_{trans/exo}$ 307
10.22 Isotopic distribution of S27$_{cis/exo}$ 307
10.23 Isotopic distribution of S27$_{trans/endo}$ 308
10.24 Isotopic distribution of S27$_{cis/endo}$ 308

10.25 Isotopic distribution of 8_3a ... 309
10.26 Isotopic distribution of 8_3b ... 309
10.27 Isotopic distribution of 8_3c ... 310
10.28 Isotopic distribution of 8_3d ... 310
10.29 Isotopic distribution of 8_5 ... 311
10.30 Isotopic Distribution of S33 ... 311
10.31 Isotopic Distribution of 12 ... 312
10.32 Isotopic Distribution of S41 ... 312
10.33 Isotopic Distribution of S42 ... 313
10.34 Isotopic Distribution of S49 ... 313
11 Rotaxane Modelling ... 314
11.1 General Procedure ... 314
11.2 Stoppering Ability of the Diels-Alder Adduct ... 314
12 References ... 316

2 General Experimental Details

Unless otherwise stated, all reagents and solvents were purchased from commercial suppliers and used without further purification. Dry solvents were obtained by passing through an activated alumina column on a Phoenix SDS solvent drying system (JC Meyer Solvent Systems, CA, USA). Compounds **S23**[(1)], **S28**[(2)], **S34**[(3)], and **S53**[(4)] were prepared according to literature procedures.

Size exclusion chromatography (SEC) analyses were performed in THF solution (1.0 mg mL^{-1}) at 40 °C using a GPC/SEC Agilent 1260 Infinity II with 2 × PL gel 10 µm mixed-B and a PL gel 500 Å column, and equipped with a differential refractive index (DRI) detector employing narrow polydispersity polystyrene standards (Agilent Technologies) as a calibration reference. Samples were filtered through a Whatman Puradisc 4 mm syringe filter with 0.45 µm PTFE membrane before injection to equipment, and experiments were carried out with injection volume of 50 µL, flow rate of 1 mL min^{-1}. Results were analyzed using *n*-dodecane as internal marker using Agilent GPC/SEC Software Version 2.2.

Ultrasound experiments were performed using a Sonics VCX 500 ultrasonic processor equipped with a 13 mm diameter solid or replaceable-tip probe. The distance between the titanium tip and the bottom of the Suslick cell was 2 cm. The ultrasonic intensity was calibrated using the method outlined by Hickenboth *et al*.[2] The Suslick cells were fabricated by the Department of Chemistry glass workshop at the University of Manchester.

Solid-state compression experiments were carried out by placing the appropriate polymer material within an International Crystal Laboratories 13mm KBr pellet die and force was applied using a Specac Atlas 15T manual hydraulic press.

Analytical TLC was performed on precoated silica gel plates (0.25 mm thick, 60 F254, Merck, Germany) and observed under UV light or stained with a potassium permanganate base solution. Preparative TLC was performed on precoated silica gel plates: 500 µm or 2000 µm, UNIPLATE GF, Analtech Inc., DE, USA. Flash column chromatography was performed with silica gel 60 (230-400 mesh) from Sigma-Aldrich. ^{1}H and ^{13}C NMR spectra were recorded on a Bruker Avance III 600 MHz Prodigy instrument, a Bruker Avance III 500 MHz Prodigy instrument or a Bruker Avance III 400 MHz Prodigy instrument. Chemical shifts are reported in parts per million (ppm) from high to low frequency and referenced to the residual solvent resonance. Coupling constants (*J*) are reported in Hertz (Hz) and splitting patterns are designated as follows: b = broad, s = singlet, d = doublet, t = triplet, q = quartet, p = pentet and m = multiplet. ^{1}H and ^{13}C assignments were made using 1D or 2D NMR methods (HSQC, HMBC, COSY). Mass spectra were obtained through the Mass Spectrometry services in the Department of Chemistry at the University of Manchester.

Abbreviations: BiBB: bromoisobutyryl bromide; BTBSCl: 3,5-bis(trifluoromethyl)benzenesulfonyl chloride; DCM: dichloromethane; DDQ: 2,3-dichloro-5,6-dicyano-1,4-benzoquinone; DIPEA: N,N-diisopropylethylamine; DMF: dimethylformamide; DMSO: dimethylsulfoxide; ESI: electrospray ionization; EtOAc: ethyl acetate; Et_2O: diethyl ether; HRMS: high resolution mass spectrometry; MeCN: acetonitrile; MeOH: methanol; MS: mass spectrometry; Me_6TREN: tris[2-(dimethylamino)ethyl]amine; PE: petroleum ether; PMDETA: N,N,N′,N′′,N′′-Pentamethyldiethylenetriamine; TBAI: tetrabutylammonium iodide; THF: tetrahydrofuran; TLC: thin layer chromatography.

3 Nomenclature of Multi-Cargo-Containing Compounds and Polymers

In this work, we discuss the synthesis and mechanical activation of complex rotaxane systems that are capable of releasing up to five cargo units *via* retro-Diels-Alder reactions. Owing to the numerous isomers that such systems can adopt, in the case of the three- and five-cargo systems, we had to synthesise multiple batches of material, each of which contained different ratios of *endo* to *exo* structures; on top of this, in some cases a single batch was used to form multiple different polymer samples. Here, for the sake of clarity, we discuss our chosen nomenclature for differentiating between these batches and their respective polymers.

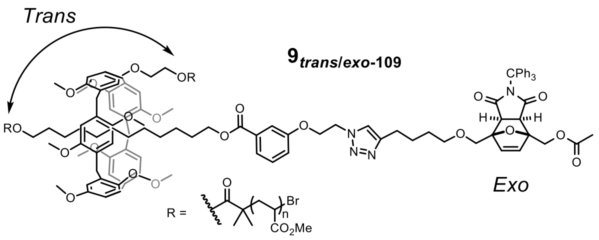

Figure S1. Example of nomenclature for 1-cargo systems using polymer $\mathbf{9}_{\boldsymbol{trans/exo}\text{-}\mathbf{109}}$.

1-cargo mechanophores have *cis* and *trans* isomers with respect to the asymmetrical pillararene macrocycle's direction relative to the rotaxane's axle (see *Section 4.3*) along with *endo* and *exo* isomers with respect to the Diels-Alder cargo unit; therefore, any such species has these designations declared in its assigned name explicitly. Such an example can be seen in *Figure S1*.

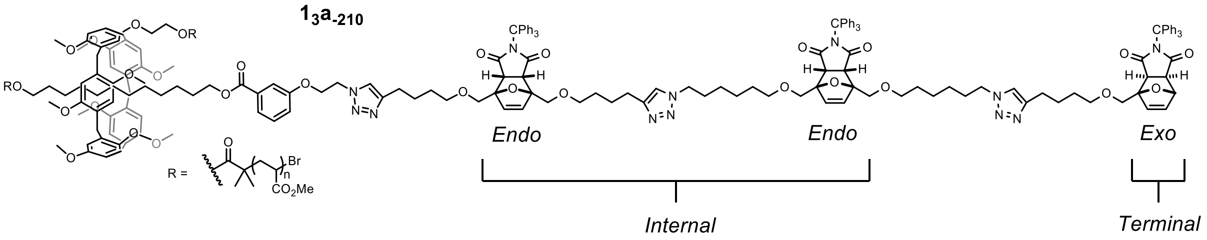

Figure S2. Example of nomenclature for 3- and 5-cargo systems using an isomer found in 3-cargo polymer $\mathbf{1_3a_{\text{-}210}}$.

Any multi-cargo mechanophore that has multiple batches with differing ratios of *endo* to *exo* isomers has a lettered suffix to declare its batch; this designation is consistent with any polymers that are synthesised from these specific batches. For example, 3-cargo small molecule compounds are named **S18** (see *Section 4.1*) and, depending on their batch, have a suffix from **a** to **d** (**S18a**, **S18b**, **S18c**, and **S18d**); polymers grown from these batches are respectively named $\mathbf{1_3a\text{-}d}$.

One of the most complex examples, $\mathbf{1_3a_{\text{-}210}}$ (see *Figure S2*), can have its nomenclature explained as follows: **1** is the compound designation, $_{\mathbf{3}}$ is the number of cargo units, **a** is the batch assignment, and $_{\mathbf{210}}$ is the M_n of the polymer in kDa.

Figure S2 also demonstrates how we refer to the ordering of the Diels-Alder adduct isomers within a cargo compartment; we determined that polymer $\mathbf{1_3a_{-210}}$ contains an isomer of order *endo*-*endo*-*exo* where *exo* is the terminal adduct.

4 Synthesis of Rotaxanes and Reference compounds

4.1 Synthesis of 1-, 3-, and 5-Cargo Diels-Alder Cargo Compartments

4.1.1 Synthetic Route to *Endo* and *Exo* 1-Cargo Diels-Alder Cargo Compartments

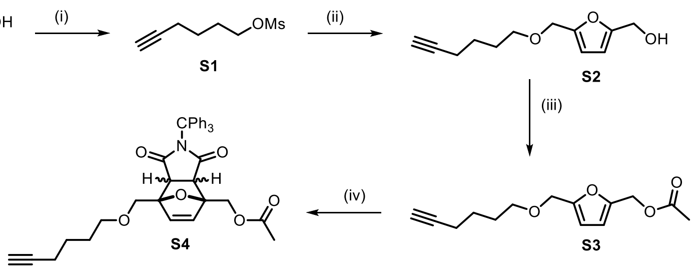

Figure S3. Synthetic route to **S4**. Conditions: (i) MsCl, Et_3N, DCM, r.t., 4 h, 91% yield; (ii) 2,5-Bis(hydroxymethyl)furan, NaH, THF, 50 °C, 8 h, 36% yield; (iii) Acetyl bromide, Et_3N, DCM, r.t., 3 h, 79% yield; (iv) **2**, DMF, 80 °C, 25% yield for both **S4**$_{exo}$ and **S4**$_{endo}$.

4.1.2 Synthesis of 2

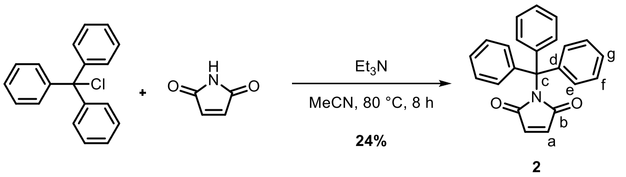

Trityl chloride (3.00 g, 10.8 mmol, 1.0 eq.) and maleimide (4.18 g, 43.0 mmol, 4.0 eq.) were added to acetonitrile (54 mL). The mixture was heated to 80 °C until all the starting materials had dissolved. At this point, Et_3N (1.65 mL, 11.8 mmol, 1.1 eq.) was added slowly. The mixture was stirred at 80 °C for 8 h before being condensed. The crude material was purified via column chromatography (SiO_2, PE/EtOAc, 2/1) to give the pure product as a beige powder (0.87 g, 2.56 mmol, 24% yield).

^{1}H NMR: (400 MHz, CD_3CN, 298 K) δ = 7.43 – 7.38 (m, 6H, H_e), 7.29 – 7.23 (m, 6H, H_f), 7.23 – 7.16 (m, 3H, H_g), 6.67 (s, 2H, H_a).
^{13}C NMR: (101 MHz, CD_3CN, 298 K) δ = 171.91 (C_b), 143.96 (C_d), 135.88 (C_a), 129.52 (C_e), 128.51 (C_f), 127.58 (C_g), 73.80 (C_c).
HRMS-ESI(+): 362.1135 $[M+Na]^+$, calculated for $C_{23}H_{17}O_2NNa$: 362.1152.

4.1.3 Synthesis of S1

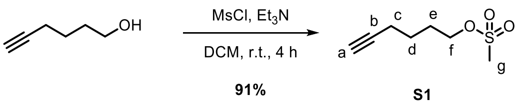

5-Hexyn-1-ol (2.00 g, 20.4 mmol, 1.0 eq.) and Et_3N (4.30 mL, 30.6 mmol, 1.5 eq.) were dissolved in DCM (41 mL). The mixture was cooled in an ice bath and placed under N_2. Mesyl chloride (2.10 mL, 26.5 mmol, 1.3 eq.) was added dropwise. The mixture was left to stir in the ice bath for 1 h. The mixture was then left to stir at r.t. for 3 h. The mixture was diluted with DCM and thoroughly washed with water followed by brine. The organic phase was dried over magnesium sulfate, filtered and condensed. The crude product required no further purification and was a light yellow liquid (3.30 g, 18.6 mmol, 91% yield).

CAS: 79496-61-0

^{1}H NMR: (400 MHz, $CDCl_3$, 298 K) δ = 4.26 (t, J = 6.3 Hz, 2H, H_f), 3.01 (s, 3H, H_g), 2.26 (td, J = 6.9, 2.7 Hz, 2H, H_c), 1.97 (t, J = 2.7 Hz, 1H, H_a), 1.94 – 1.84 (m, 2H, H_e), 1.71 – 1.61 (m, 2H, H_d).
^{13}C NMR: (101 MHz, $CDCl_3$, 298 K) δ = 83.49 (C_b), 69.50 (C_f), 69.24 (C_a), 37.54 (C_g), 28.18 (C_e), 24.40 (C_d), 17.95 (C_c).
HRMS-ESI(+): 199.0394 $[M+Na]^+$, calculated for $C_7H_{12}O_3SNa$: 199.0399.

4.1.4 Synthesis of S2

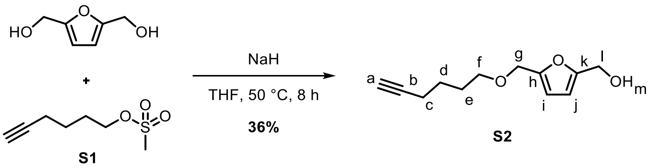

2,5-Bis(hydroxymethyl)furan (1.00 g, 7.81 mmol, 1.0 eq.) was added to dry THF (39 ml) and the mixture cooled in an ice bath. NaH (60% in mineral oil, 0.34 g, 8.59 mmol, 1.1 eq.) was added and the mixture stirred for 5 min under N_2. **S1** (1.51 g, 8.59 mmol, 1.1 eq.) was added. The reaction mixture was stirred at 50 °C for 8 h. Water was added carefully to quench the reaction. The reaction mixture was condensed to remove as much THF as possible. DCM was added to the remaining aqueous residue and the mixture thoroughly washed with water followed by brine. The organic phase was dried over magnesium sulfate, filtered and condensed. The crude material was purified via column chromatography (SiO_2, PE/EtOAc, 1/1) to give the pure product as a colourless viscous liquid (0.59 g, 2.83 mmol, 36% yield).

^{1}H NMR: (500 MHz, $CDCl_3$, 298 K) δ = 6.25 (d, J = 3.3 Hz, 1H, H_i), 6.24 (d, J = 3.3 Hz, 1H, H_j), 4.59 (d, J = 3.5 Hz, 2H, H_l), 4.41 (s, 2H, H_g), 3.49 (t, J = 6.4 Hz, 2H, H_f), 2.20 (td, J = 7.1, 2.7 Hz, 2H, H_c), 1.94 (t, J = 2.7 Hz, 1H, H_a), 1.80 (t, J = 5.6 Hz, 1H, H_m), 1.75 – 1.67 (m, 2H, H_e), 1.64 – 1.56 (m, 2H, H_d).
^{13}C NMR: (126 MHz, $CDCl_3$, 298 K) δ = 154.37 (C_k), 152.17 (C_h), 110.03 (C_i), 108.58 (C_j), 84.45 (C_b), 69.82 (C_f), 68.57 (C_a), 64.97 (C_g), 57.75 (C_l), 28.72 (C_e), 25.23 (C_d), 18.28 (C_c).
HRMS-ESI(+): 231.0983 $[M+Na]^+$, calculated for $C_{12}H_{16}O_3Na$: 231.0992.

4.1.5 Synthesis of S3

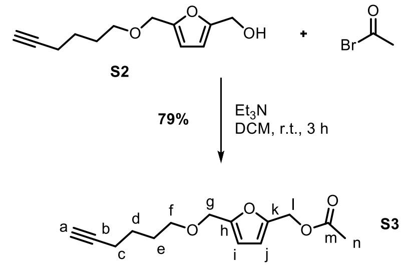

S2 (0.40 g, 1.92 mmol, 1.0 eq.) was dissolved in DCM (9.6 mL) along with Et_3N (0.40 mL, 2.88 mmol, 1.5 eq.). The mixture was cooled in an ice bath and placed under N_2. Acetyl bromide (0.17 mL, 2.31 mmol, 1.2 eq.) was added dropwise. The mixture was stirred at r.t. for 3 h. The mixture was diluted with DCM and washed thoroughly with water followed by brine. The organic phase was dried over magnesium sulfate, filtered and condensed to give the crude product. Purification was carried out via column chromatography (SiO_2, PE/EtOAc, 3/1) to give the pure product as a colourless liquid (0.38 g, 1.52 mmol, 79% yield).

^{1}H NMR: (500 MHz, CD_3CN, 298 K) δ = 6.39 (d, J = 3.1 Hz, 1H, H_j), 6.32 (d, J = 3.2 Hz, 1H, H_i), 5.00 (s, 2H, H_l), 4.37 (s, 2H, H_g), 3.53 (t, J = 7.1 Hz, 2H, H_f), 2.18 (td, J = 7.1, 2.7 Hz, 2H, H_c), 2.15 (t, J = 2.4 Hz, 1H, H_a), 2.02 (s, 3H, H_n), 1.67 – 1.59 (m, 2H, H_e), 1.56 – 1.48 (m, 2H, H_d).
^{13}C NMR: (126 MHz, CD_3CN, 298 K) δ = 171.24 (C_m), 154.12 (C_h), 150.88 (C_k), 112.21 (C_j), 110.98 (C_i), 85.26 (C_b), 70.26 (C_f), 69.79 (C_a), 65.15 (C_g), 58.79 (C_l), 29.36 (C_e), 26.01 (C_d), 20.98 (C_n), 18.50 (C_c).
HRMS-ESI(+): 273.1085 $[M+Na]^+$, calculated for $C_{14}H_{18}O_4Na$: 273.1097.

4.1.6 Synthesis of S4

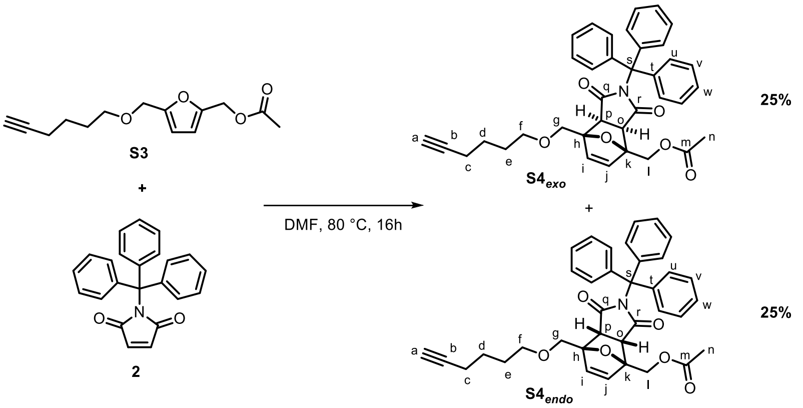

A solution of **S3** (50 mg, 0.20 mmol, 1.0 eq.) and **2** (136 mg, 0.40 mmol, 2.0 eq.) in DMF (1 mL) was stirred at 80 °C for 16 h. The reaction mixture was then diluted with DCM (5 mL) and the resulting solution washed by water (2 x 5 mL) and brine (1 x 5 mL). The organic phase was separated and dried with magnesium sulfate before being filtered and concentrated under vacuum. The crude material was purified by flash column chromatography (SiO_2, PE/EtOAc, 3/1) to yield **S4*exo*** (30 mg, 0.05 mmol, 25% yield) and **S4*endo*** (30 mg, 0.05 mmol, 25% yield) as faint yellow powders.

Compound **S4*exo***:

^{1}H NMR (400 MHz, Acetone-*d*$_6$, 298 K) δ = 7.52 – 7.45 (m, 6H, H_u), 7.29 – 7.23 (m, 6H, H_v), 7.19 – 7.13 (m, 3H, H_w), 6.62 (d, *J* = 5.6 Hz, 1H, H_j), 6.54 (d, *J* = 5.6 Hz, 1H, H_i), 4.73 (s, 2H, H_l), 4.18 (d, *J* = 11.7 Hz, 1H, H_g), 3.91 (d, *J* = 11.7 Hz, 1H, H_g), 3.68 (dt, *J* = 9.5, 6.2 Hz, 1H, H_f), 3.57 (dt, *J* = 9.5, 6.1 Hz, 1H, H_f), 3.00 – 2.93 (d, *J* = 6.6 Hz, d, *J* = 6.6 Hz, 2H, $H_{o,p}$), 2.33 (t, *J* = 2.7 Hz, 1H, H_a), 2.23 (td, *J* = 7.0, 2.7 Hz, 2H, H_c), 2.08 (s, 3H, H_n), 1.76 – 1.67 (m, 2H, H_e), 1.66 – 1.57 (m, 2H, H_d).
^{13}C NMR (101 MHz, Acetone-*d*$_6$, 298 K) δ = 173.91 (C_r), 173.87 (C_q), 170.71 (C_m), 143.38 (C_t), 139.45 (C_j), 137.94 (C_i), 129.35 (C_u), 128.26 (C_v), 127.25 (C_w), 92.34 (C_h), 90.76 (C_k), 84.92 (C_b), 74.26 (C_s), 71.82 (C_f), 69.99 (C_a), 69.01 (C_g), 62.24 (C_l), 51.39, 51.37 ($C_{o,p}$), 29.38 (C_e), 26.06 (C_d), 20.70 (C_n), 18.51 (C_c).
HRMS-ESI(+): 612.2361 $[M+Na]^+$, calculated for $C_{37}H_{35}NO_6Na^+$: 612.2357.

Compound **S4*endo***:

^{1}H NMR (400 MHz, Acetone-*d*$_6$, 298 K) δ = 7.46 – 7.42 (m, 6H, H_u), 7.26 – 7.19 (m, 6H, H_v), 7.18 – 7.11 (m, 3H, H_w), 5.47 – 5.42 (m, 2H, $H_{i,j}$), 4.59 (d, *J* = 12.8 Hz, 1H, H_l), 4.38 (d, *J* = 12.8 Hz, 1H, H_l), 3.91 (d, *J* = 12.0 Hz, 1H, H_g), 3.83 (d, *J* = 12.1 Hz, 1H, H_g), 3.72 (d, *J* = 8.3 Hz, 1H, H_p), 3.66 (d, *J* = 8.2 Hz, 1H, H_o), 3.50 (td, *J* = 6.2, 1.2 Hz, 2H, H_f), 2.32 (t, *J* = 2.7 Hz, 1H, H_a), 2.18 (td, *J* = 7.0, 2.7 Hz, 2H, H_c), 1.99 (s, 3H, H_n), 1.70 – 1.60 (m, 2H, H_e), 1.59 – 1.50 (m, 2H, H_d).
^{13}C NMR (101 MHz, Acetone-*d*$_6$, 298 K) δ = 175.28 (C_r), 175.12 (C_q), 170.57 (C_m), 143.37 (C_t), 136.82 (C_i), 135.77 (C_j), 129.33 (C_u), 128.06 (C_v), 127.08 (C_w), 92.30 (C_h), 90.24 (C_k), 84.84 (C_b), 74.59 (C_s), 71.60 (C_f), 69.99 (C_a), 69.38 (C_g), 62.82 (C_l), 48.81 (C_o), 48.00 (C_p), 29.38 (C_e), 26.00 (C_d), 20.57 (C_n), 18.45 (C_c).
HRMS-ESI(+): 612.2350 $[M+Na]^+$, calculated for $C_{37}H_{35}NO_6Na^+$: 612.2357.

4.1.7 Synthetic Route to Attachment of Carboxylic Acid Linker to 1-Cargo Diels-Alder Cargo Compartments

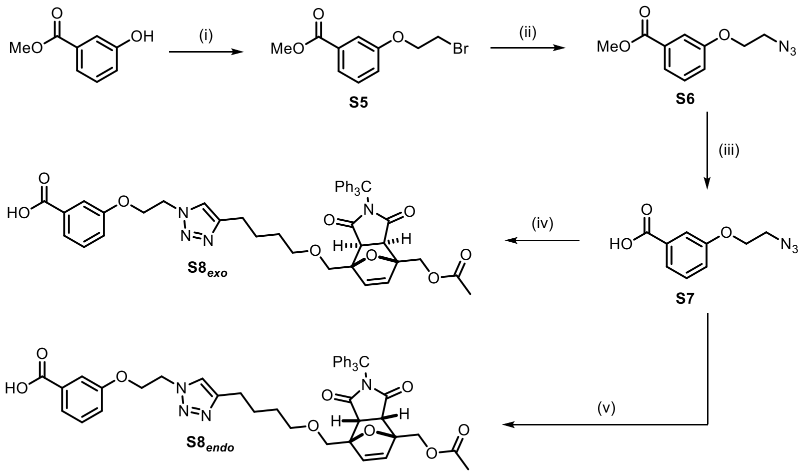

Figure S4. Synthetic route to **S8**. Conditions: (i) 1,2-dibromoethane, K_2CO_3, MeCN, 60 °C, 16 h, 72% yield; (ii) NaN_3, acetone/H_2O, 55 °C, 16 h, 93% yield; (iii) LiOH, THF/H_2O, 60 °C, 16 h, 94% yield; (iv) **S4*exo***, CuBr, PMDETA, DCM, r.t., 16 h, 82% yield; (v) **S4*endo***, CuBr, PMDETA, DCM, r.t., 16 h, 86% yield.

4.1.8 Synthesis of S5

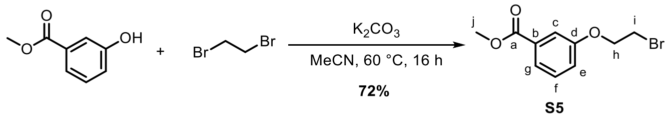

To a solution of methyl 3-hydroxybenzoate (1.00 g, 6.6 mmol, 1.0 eq.) and 1,2-dibromoethane (1.96 g, 52.6 mmol, 8.0 eq.) in acetonitrile (40 mL) was added K_2CO_3 (3.63 g, 26.3 mmol, 4.0 eq.). The mixture was heated to 60 °C for 16 h. The solution was cooled down to room temperature. The mixture was filtered, and the filtrate concentrated under vacuum. The residue was purified by flash column chromatography (SiO_2, PE/EtOAc, 15/1) to yield **S5** as a white powder (1.22 g, 4.7 mmol, 72% yield).

CAS: 59516-96-0
^{1}H NMR (500 MHz, $CDCl_3$, 298 K) δ = 7.65 (dt, *J* = 7.7, 1.2 Hz, 1H, H_g), 7.54 (dd, *J* = 2.7, 1.5 Hz, 1H, H_c), 7.33 (t, *J* = 8.0 Hz, 1H, H_f), 7.10 (ddd, *J* = 8.2, 2.6, 1.0 Hz, 1H, H_e), 4.31 (t, *J* = 6.2 Hz, 2H, H_h), 3.89 (s, 3H, H_j), 3.63 (t, *J* = 6.2 Hz, 2H, H_i).
^{13}C NMR (126 MHz, $CDCl_3$, 298 K) δ = 166.77 (C_a), 158.12 (C_d), 131.61 (C_b), 129.64 (C_f), 122.73 (C_g), 120.18 (C_e), 114.89 (C_c), 68.05 (C_h), 52.27 (C_j), 29.04 (C_i).
HRMS-ESI(+): 258.9965 $[M+H]^+$, calculated for $C_{10}H_{12}BrO_3^+$: 258.9964.

4.1.9 Synthesis of S6

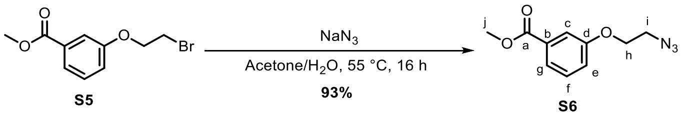

To a solution of **S5** (1088 mg, 4.2 mmol, 1.0 eq.) in a mixture of acetone (20 mL) and water (10 mL) was added sodium azide (411 mg, 6.3 mmol, 1.5 eq.). The mixture was heated to 55 °C for 16 h. The solution was cooled down to room temperature. The mixture was concentrated under vacuum to remove the organic solvent. The residual aqueous mixture was extracted with EtOAc (3 x 50 mL). The organic phases were combined and dried with magnesium sulfate. The mixture was filtered and concentrated under vacuum and the residue purified by flash column chromatography (SiO_2, PE/EtOAc, 15/1) to yield **S6** as a white powder (877 mg, 4.0 mmol, 93% yield).

^{1}**H NMR** (500 MHz, $CDCl_3$, 298 K) δ = 7.66 (dt, J = 7.7, 1.2 Hz, 1H, H_g), 7.56 (dd, J = 2.7, 1.5 Hz, 1H, H_c), 7.35 (t, J = 7.9 Hz, 1H, H_f), 7.13 (ddd, J = 8.2, 2.7, 1.0 Hz, 1H, H_e), 4.19 (t, J = 5.0 Hz, 2H, H_h), 3.91 (s, 3H, H_j), 3.61 (t, J = 5.0 Hz, 2H, H_i).
^{13}**C NMR** (126 MHz, $CDCl_3$) δ = 166.89 (C_a), 158.30 (C_d), 131.64 (C_b), 129.68 (C_f), 122.77 (C_g), 120.23 (C_e), 114.64 (C_c), 67.25 (C_h), 52.33 (C_j), 50.20 (C_i).
HRMS-ESI(+): 222.0869 $[M+H]^+$, calculated for $C_{10}H_{12}N_3O_3^+$: 222.0873.

4.1.10 Synthesis of S7

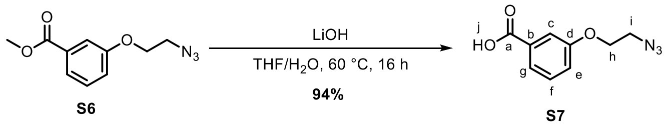

To a solution of **S6** (877 mg, 4.0 mmol, 1.0 eq.) in a mixture of THF (8 mL) and water (8 mL) was added LiOH (1900 mg, 79.3 mmol, 20.0 eq.). The mixture was heated to 60 °C for 16 h. After the solution was cooled down to room temperature, the pH was adjusted to ~2 by addition of aqueous 1 M HCl. The resulting solution was extracted with EtOAc (3 x 25 mL). The organic phases were combined and dried with magnesium sulfate. The mixture was filtered and concentrated under vacuum. The residual material was dried under high vacuum to yield **S7** as a white powder (770 mg, 3.7 mmol, 94% yield).

CAS: 2024604-49-5
^{1}**H NMR** (500 MHz, $CDCl_3$, 298 K) δ = 13.3 – 8.5 (b, 1H, H_j), 7.76 (dt, J = 7.6, 1.3 Hz, 1H, H_g), 7.64 (dd, J = 2.7, 1.5 Hz, 1H, H_c), 7.40 (t, J = 7.9 Hz, 1H, H_f), 7.20 (ddd, J = 8.2, 2.7, 1.0 Hz, 1H, H_e), 4.21 (t, J = 4.8 Hz, 2H, H_h), 3.63 (t, J = 4.8 Hz, 2H, H_i).
^{13}**C NMR** (126 MHz, $CDCl_3$, 298 K) δ = 172.15 (C_a), 158.38 (C_d), 130.78 (C_b), 129.85 (C_f), 123.48 (C_g), 121.25 (C_e), 115.07 (C_c), 67.31 (C_h), 50.21 (C_i).
HRMS-ESI(+): 206.0571 $[M-H]^-$, calculated for $C_9H_8N_3O_3^-$: 206.0571.

4.1.11 Synthesis of S8*exo*

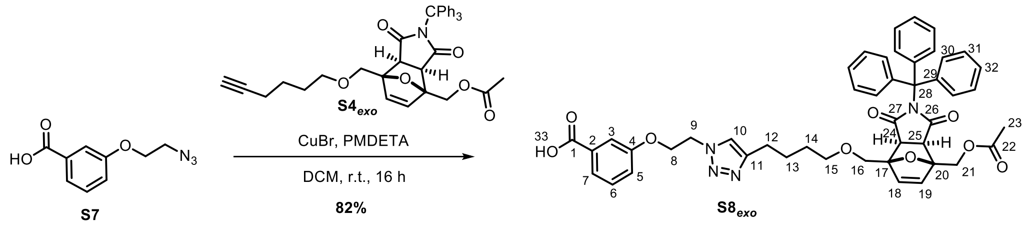

Three sealed 5 mL microwave vials (*A*, *B*, and *C*) were prepared as follows: *A* contained PMDETA (5 mg, 29 µmol, 1.5 eq.) in DCM (3 mL), *B* contained CuBr (4.5 mg, 31 µmol, 1.6 eq.), and *C* contained **S4*exo*** (11 mg, 19 µmol, 1.0 eq.) and **S7** (4 mg, 19 µmol, 1.0 eq.). Vials *B* and *C* were subjected to three N_2/vacuum cycles before use. The solution in vial *A* was degassed by bubbling with N_2 until ~1 mL DCM left before being transferred to vial *B via* cannula; the resulting mixture was stirred until all CuBr had dissolved. The CuBr/PMDETA solution in vial *B* was then transferred to vial *C via* cannula; the resulting reaction mixture was stirred for 16 h at room temperature. The reaction mixture was washed by aqueous EDTA solution (0.25 M, pH 7, 2 x 1 mL) and brine (2 x 1 mL). The organic layer was collected and dried with magnesium sulfate. The mixture was filtered before being concentrated under vacuum. The residue was purified by preparative TLC (500 µm, DCM/MeOH, 20/1, eluted twice) to yield **S8*exo*** as a white powder (12 mg, 15 µmol, 82% yield).

^{1}**H NMR** (500 MHz, Acetone-d_6, 298 K) δ = 7.82 (s, 1H, H_{10}), 7.64 (dt, J = 7.8, 1.2 Hz, 1H, H_7), 7.57 – 7.54 (m, 1H, H_3), 7.48 (d, J = 8.7 Hz, 6H, H_{30}), 7.41 (t, J = 7.9 Hz, 1H, H_6), 7.26 (t, J = 7.7 Hz, 6H, H_{31}), 7.19 (dd, J = 8.2, 2.6 Hz, 1H, H_5), 7.18 – 7.14 (m, 3H, H_{32}), 6.61 (d, J = 5.6 Hz, 1H, H_{19}), 6.52 (d, J = 5.5 Hz, 1H, H_{18}), 4.80 (t, J = 5.1 Hz, 2H, H_9), 4.76 – 4.68 (m, 2H, H_{21}), 4.51 (t, J = 5.1 Hz, 2H, H_8), 4.16 (d, J = 11.6 Hz, 1H, H_{16}), 3.91 (d, J = 11.6 Hz, 1H, H_{16}), 3.67 (dt, J = 9.4, 6.3 Hz, 1H, H_{15}), 3.56 (dt, J = 9.4, 6.3 Hz, 1H, H_{15}),

2.99 – 2.93 (d, J = 6.5 Hz, d, J = 6.5 Hz, 2H, $H_{24,25}$), 2.71 (t, J = 7.5 Hz, 2H, H_{12}), 2.05 (s, 3H, H_{23}), 1.75 (m, 2H, H_{13}), 1.69 – 1.62 (m, 2H, H_{14}).
^{13}C NMR (126 MHz, Acetone-d_6, 298 K) δ = 173.89, 173.88 ($C_{26,27}$), 170.72 (C_{22}), 167.28 (C_1), 159.30 (C_4), 148.23 (C_{11}), 143.35 (C_{29}), 139.49 (C_{19}), 137.90 (C_{18}), 132.91 (C_2), 130.57 (C_6), 129.32 (C_{30}), 128.26 (C_{31}), 127.25 (C_{32}), 123.29 (C_7), 122.88 (C_{10}), 120.22 (C_5), 116.12 (C_3), 92.30 (C_{17}), 90.71 (C_{20}), 74.21 (C_{28}), 72.10 (C_{15}), 68.96 (C_{16}), 67.70 (C_8), 62.22 (C_{21}), 51.36, 51.35 ($C_{24,25}$), 49.97 (C_9), 29.74 (C_{14}), 26.86 (C_{13}), 25.93 (C_{12}), 20.69 (C_{23}).
HRMS-ESI(-): 795.3044 $[M-H]^-$, calculated for $C_{46}H_{43}N_4O_9^-$: 795.3036.

4.1.12 Synthesis of S8*endo*

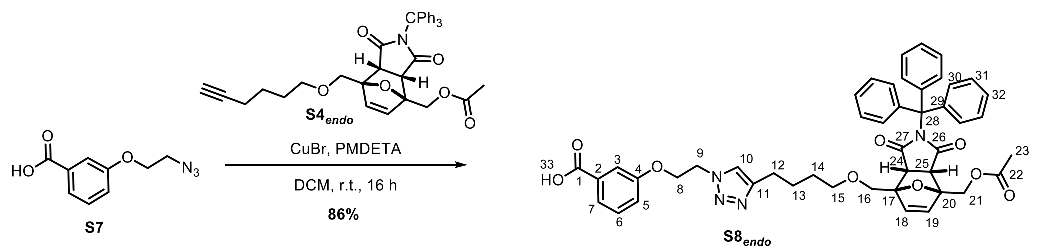

Three sealed 5 mL microwave vials (*A*, *B*, and *C*) were prepared as follows: *A* contained PMDETA (5 mg, 29 µmol, 1.5 eq.) in DCM (3 mL), *B* contained CuBr (4.5 mg, 31 µmol, 1.6 eq.), and *C* contained **S4*endo*** (11 mg, 19 µmol, 1.0 eq.) and **S7** (4 mg, 19 µmol, 1.0 eq.). Vials *B* and *C* were subjected to three N_2/vacuum cycles before use. The solution in vial *A* was degassed by bubbling with N_2 until ~1 mL DCM left before being transferred to vial *B via* cannula; the resulting mixture was stirred until all CuBr had dissolved. The CuBr/PMDETA solution in vial *B* was then transferred to vial *C via* cannula; the resulting reaction mixture was stirred for 16 h at room temperature. The reaction mixture was washed by aqueous EDTA solution (0.25 M, pH 7, 2 x 1 mL) and brine (2 x 1 mL). The organic layer was collected and dried with magnesium sulfate. The mixture was filtered before being concentrated under vacuum. The residue was purified by preparative TLC (500 µm, DCM/MeOH, 20/1, eluted twice) to yield **S8*endo*** as a white powder (13 mg, 16 µmol, 86% yield).

^{1}H NMR (500 MHz, Acetone-d_6, 298 K) δ = 7.81 (s, 1H, H_{10}), 7.64 (dd, J = 7.7, 1.3 Hz, 1H, H_7), 7.57 – 7.54 (m, 1H, H_3), 7.44 (d, J = 7.8 Hz, 6H, H_{30}), 7.41 (t, J = 7.9 Hz, 1H, H_6) 7.24 – 7.18 (m, 7H, $H_{5,31}$), 7.16 – 7.10 (m, 3H, H_{32}), 5.45 – 5.41 (m, 2H, $H_{18,19}$), 4.79 (t, J = 5.1 Hz, 2H, H_9), 4.58 (d, J = 12.8 Hz, 1H, H_{21}), 4.51 (t, J = 5.1 Hz, 2H, H_8), 4.38 (d, J = 12.8 Hz, 1H, H_{21}), 3.88 (d, J = 12.0 Hz, 1H, H_{16}), 3.81 (d, J = 12.0 Hz, 1H, H_{16}), 3.72 (d, J = 8.3 Hz, 1H, H_{24}), 3.65 (d, J = 8.2 Hz, 1H, H_{25}), 3.49 (t, J = 6.3 Hz, 2H, H_{15}), 2.67 (t, J = 7.5 Hz, 2H, H_{12}), 1.97 (s, 3H, H_{23}), 1.74 – 1.65 (m, 2H, H_{13}), 1.62 – 1.53 (m, 2H, H_{14}).
^{13}C NMR (126 MHz, Acetone-d_6, 298 K) δ = 175.33 (C_{26}), 175.13 (C_{27}), 170.59 (C_{22}), 167.29 (C_1), 159.31 (C_4), 148.15 (C_{11}), 143.36 (C_{29}), 136.84 (C_{18}), 135.72 (C_{19}), 132.91 (C_2), 130.58 (C_6), 129.31 (C_{30}), 128.06 (C_{31}), 127.07 (C_{32}), 123.29 (C_7), 122.88 (C_{10}), 120.28 (C_5), 116.10 (C_3), 92.26 (C_{17}), 90.21 (C_{20}), 74.56 (C_{28}), 71.87 (C_{15}), 69.29 (C_{16}), 67.70 (C_8), 62.81 (C_{21}), 49.97 (C_9), 48.79 (C_{25}), 47.94 (C_{24}), 29.94 (C_{14}), 26.80 (C_{13}), 25.88 (C_{12}), 20.56 (C_{23}).
HRMS-ESI(-): 795.3023 $[M-H]^+$, calculated for $C_{46}H_{43}N_4O_9^-$: 795.3036.

4.1.13 Synthetic Route to 3- and 5-Cargo Unloaded Cargo Compartments

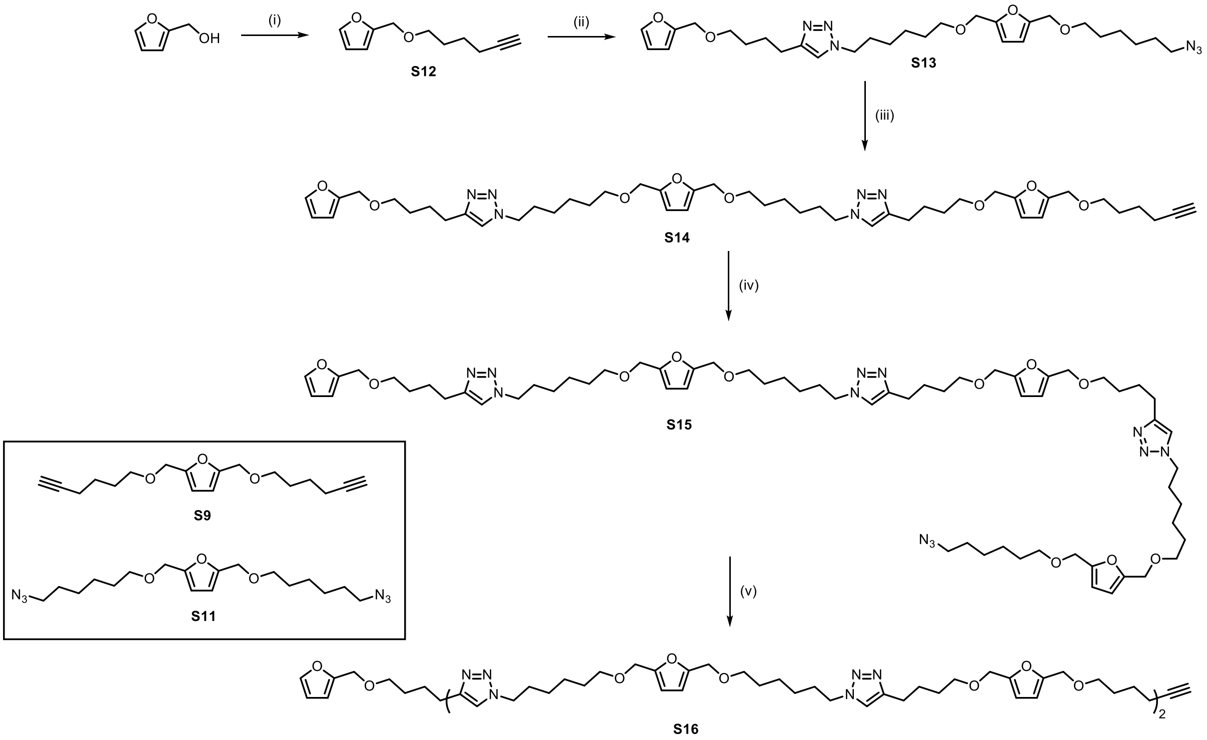

Figure S5. Synthetic route to **S16**. Conditions: (i) **S1**, NaH, THF, 60 °C, 8 h, 80% yield; (ii) **S11**, CuBr, PMDETA, DCM, r.t., 16 h, 74% yield; (iii) **S9**, CuBr, PMDETA, DCM, r.t., 16 h, 73% yield; (iv) **S11**, CuBr, PMDETA, DCM, r.t., 16 h, 62% yield; (v) **S9**, CuBr, PMDETA, DCM, r.t., 16 h, 45% yield.

4.1.14 Synthesis of S9

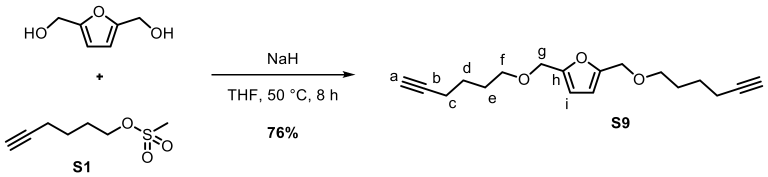

2,5-Bis(hydroxymethyl)furan (1.00 g, 7.81 mmol, 1.0 eq.) was added to dry THF (39 ml) and the mixture cooled in an ice bath. NaH (60% in mineral oil, 0.72 g, 18.0 mmol, 2.3 eq.) was added and the mixture stirred for 5 min under N_2. **S1** (3.16 g, 18.0 mmol, 2.3 eq.) was added. The reaction mixture was stirred at 50 °C for 8 h. Water was added carefully to quench the reaction. The reaction mixture was condensed to remove as much THF as possible. DCM was added to the remaining aqueous residue and the mixture thoroughly washed with water followed by brine. The organic phase was dried over magnesium sulfate, filtered and condensed. The crude material was purified via column chromatography (SiO_2, PE/EtOAc, 5/1) to give the pure product as a pale yellow liquid (1.72 g, 6.00 mmol, 76% yield).

^{1}H NMR: (500 MHz, CD_3CN, 298 K) δ = 6.29 (s, 2H, H_i), 4.37 (s, 4H, H_g), 3.45 (t, J = 6.3 Hz, 4H, H_f), 2.18 (td, J = 7.0, 2.6 Hz, 4H, H_c), 2.14 (t, J = 2.6 Hz, 2H, H_a), 1.66 – 1.59 (m, 4H, H_e), 1.56 – 1.49 (m, 4H, H_d).
^{13}C NMR: (126 MHz, CD_3CN, 298 K) δ = 153.42 (C_h), 110.69 (C_i), 85.25 (C_b), 70.13 (C_f), 69.77 (C_a), 65.20 (C_g), 29.38 (C_e), 26.03 (C_d), 18.51 (C_c).
HRMS-ESI(+): 311.1603 $[M+Na]^+$, calculated for $C_{18}H_{24}O_3Na$: 311.1618.

4.1.15 Synthesis of S10

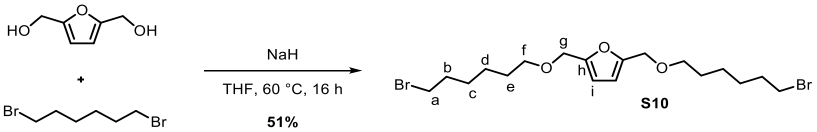

2,5-Bis(hydroxymethyl)furan (2.00 g, 15.6 mmol, 1.0 eq.) was dissolved in dry THF (78 mL) and the mixture cooled in an ice bath. NaH (60% in mineral oil, 1.56 g, 39.0 mmol, 2.5 eq.) was added and the mixture stirred for 5 min under N_2. 1,6-Dibromohexane (16.8 mL, 109.3 mmol, 7.0 eq.) was added and the mixture stirred at 60 °C overnight. Water was added to quench the reaction. The mixture was condensed to remove as much THF as possible. DCM was added to the remaining aqueous residue which was then washed thoroughly with water followed by brine. The organic phase was dried over magnesium sulfate, filtered, and condensed. The crude material was purified via column chromatography (SiO_2, PE/EtOAc, 10/1) to give the pure product as a very pale yellow liquid (3.61 g, 7.93 mmol, 51% yield).

^{1}H NMR: (500 MHz, CD_3CN, 298 K) δ = 6.28 (s, 2H, H_i), 4.36 (s, 4H, H_g), 3.46 (t, J = 7.0 Hz, 4H, H_a), 3.43 (t, J = 6.6 Hz, 4H, H_f), 1.83 (p, J = 7.0 Hz, 4H, H_b), 1.54 (p, J = 6.8 Hz, 4H, H_e), 1.45 – 1.38 (m, 4H, H_c), 1.37 – 1.29 (m, 4H, H_d).
^{13}C NMR: (126 MHz, CD_3CN, 298 K) δ = 153.43 (C_h), 110.64 (C_i), 70.64 (C_f), 65.18 (C_g), 35.27 (C_a), 33.50 (C_b), 30.17 (C_e), 28.57 (C_c), 26.01 (C_d).
HRMS-APCI(+): 452.0545 $[M]^+$, calculated for $C_{18}H_{30}O_3Br_2$: 452.0556.

4.1.16 Synthesis of S11

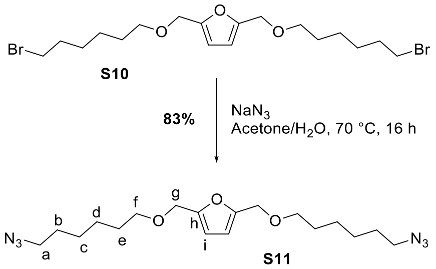

S10 (1.50 g, 3.30 mmol, 1.0 eq.) was dissolved in acetone (6.6 mL). Water was added just until the mixture was no longer miscible; minimal acetone was added in order to make the mixture miscible once more. NaN_3 (0.64 g, 9.91 mmol, 3.0 eq.) was added and the mixture stirred at 70 °C overnight. The reaction was condensed to remove as much acetone as possible. The remaining aqueous residue was diluted with EtOAc before being washed with water followed by brine. The organic phase was dried over magnesium sulfate, filtered and condensed. The crude material was purified via column chromatography (SiO_2, PE/EtOAc, 1/1) to give the pure product as a pale yellow liquid (1.04 g, 2.75 mmol, 83% yield).

^{1}H NMR: (500 MHz, CD_3CN, 298 K) δ = 6.28 (s, 2H, H_i), 4.36 (s, 4H, H_g), 3.43 (t, J = 6.5 Hz, 4H, H_f), 3.27 (t, J = 6.9 Hz, 4H, H_a), 1.60 – 1.50 (m, 8H, $H_{b,e}$), 1.39 – 1.30 (m, 8H, $H_{c,d}$).
^{13}C NMR : (126 MHz, CD_3CN, 298 K) δ = 153.48 (C_h), 110.65 (C_i), 70.69 (C_f), 65.21 (C_g), 52.08 (C_a), 30.24 (C_e), 29.41 (C_b), 27.19 (C_c), 26.44 (C_d).
HRMS-ESI(+): 401.2257 $[M+Na]^+$, calculated for $C_{18}H_{30}O_3N_6Na$: 401.2272.

4.1.17 Synthesis of S12

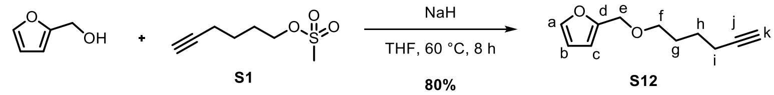

Furfuryl alcohol (200 mg, 2.04 mmol, 1.0 eq.) was added to dry THF (10 ml) and the mixture cooled in an ice bath. NaH (60% in mineral oil, 98 mg, 2.45 mmol, 1.2 eq.) was added and the mixture stirred for 5 min under N_2. **S1** (430 mg, 2.45 mmol, 1.2 eq.) was added. The reaction mixture was stirred at 60 °C for 8 h. Water was added carefully to quench the reaction. The reaction mixture was condensed to remove as much THF as possible. DCM was added to the remaining aqueous residue and the mixture thoroughly washed with water followed by brine. The organic phase was dried over magnesium sulfate, filtered, and condensed. The crude material was purified via column chromatography (SiO_2, PE/EtOAc, 5/1) to give the pure product as a colourless viscous liquid (290 mg, 1.63 mmol, 80% yield).

^{1}H NMR: (500 MHz, CD_3CN, 298 K) δ = 7.47 (dd, *J* = 1.8, 0.8 Hz, 1H, H_a), 6.38 (dd, *J* = 3.2, 1.9 Hz, 1H, H_b), 6.34 (d, *J* = 3.2 Hz, 1H, H_c), 4.39 (s, 2H, H_e), 3.44 (t, *J* = 6.3 Hz, 2H, H_f), 2.17 (td, *J* = 7.0, 2.7 Hz, 2H, H_i), 2.15 (t, *J* = 2.6 Hz, 1H, H_k), 1.66 – 1.59 (m, 2H, H_g), 1.56 – 1.49 (m, 2H, H_h).
^{13}C NMR: (126 MHz, CD_3CN, 298 K) δ = 153.42 (C_d), 143.80 (C_a), 111.30 (C_b), 109.99 (C_c), 85.27 (C_j), 70.11 (C_f), 69.76 (C_k), 65.08 (C_e), 29.39 (C_g), 26.04 (C_h), 18.51 (C_i).
HRMS-ESI(+): 201.0879 $[M+Na]^+$, calculated for $C_{11}H_{14}O_2Na$: 201.0886.

4.1.18 Synthesis of S13

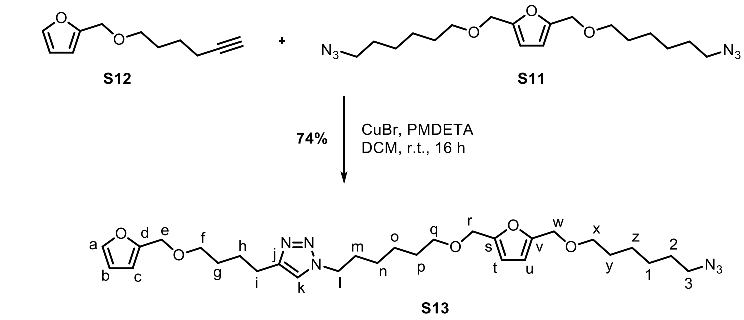

S12 (100 mg, 0.56 mmol, 1.0 eq.) and **S11** (1060 mg, 2.81 mmol, 5.0 eq.) were dissolved in DCM (5.6 mL) in a microwave vial. Separately, PMDETA (59 µL, 0.28 mmol, 0.5 eq.) was dissolved in DCM (1 mL) in a microwave vial. The two mixtures were degassed via three freeze-thaw-pump cycles. In a third microwave vial was added CuBr (16 mg, 0.11 mmol, 0.2 eq.) under N_2. The PMDETA mixture was transferred via cannula onto the CuBr; the resulting mixture was stirred until all solids had dissolved. The mixture was then degassed via a single freeze-thaw-pump cycle before being transferred via cannula onto the reaction mixture. The reaction mixture was stirred at r.t. overnight before being condensed. The crude material was purified via column chromatography (SiO_2, PE/EtOAc, 1/1) to give the pure product as a colourless viscous oil (230 mg, 0.41 mmol, 74% yield).

^{1}H NMR: (500 MHz, CD_3CN, 298 K) δ = 7.48 – 7.45 (m, 2H, $H_{a,k}$), 6.37 (dd, *J* = 3.2, 1.9 Hz, 1H, H_b), 6.33 (d, *J* = 3.2 Hz, 1H, H_c), 6.29 – 6.26 (m, 2H, $H_{t,u}$), 4.38 (s, 2H, H_e), 4.35 – 4.34 (s, s, 4H, $H_{r,w}$), 4.26 (t, *J* = 7.1 Hz, 2H, H_l), 3.47 – 3.38 (t, *J* = 6.4 Hz, t, *J* = 6.6 Hz, t, *J* = 6.5 Hz, 6H, $H_{f,q,x}$), 3.26 (t, *J* = 6.9 Hz, 2H, H_3), 2.64 (t, *J* = 7.4 Hz, 2H, H_i), 1.82 (p, *J* = 7.2 Hz, 2H, H_m), 1.68 – 1.61 (m, 2H, H_h), 1.60 – 1.48 (m, 8H, $H_{g,p,y,2}$), 1.33 (m, 6H, $H_{o,z,1}$), 1.29 – 1.22 (m, 2H, H_n).
^{13}C NMR: (126 MHz, CD_3CN, 298 K) δ = 153.47, 153.46 ($C_{d,s,v}$), 148.41 (C_j), 143.77 (C_a), 122.10 (C_k), 111.30 (C_b), 110.65 ($C_{t,u}$), 109.96 (C_c), 70.68, 70.63 ($C_{q,w}$), 70.43 (C_f), 65.20 ($C_{r,w}$), 65.07 (C_e), 52.07 (C_3), 50.53 (C_l), 30.89 (C_m) 30.23, 30.14, 29.83, 29.40 ($C_{g,p,y,2}$), 27.18, 26.43, 26.26 ($C_{o,z,1}$), 26.92, 26.89 ($C_{h,n}$), 25.93 (C_i).
HRMS-ESI(+): 579.3249 $[M+Na]^+$, calculated for $C_{29}H_{44}O_5N_6Na$: 579.3265.

4.1.19 Synthesis of S14

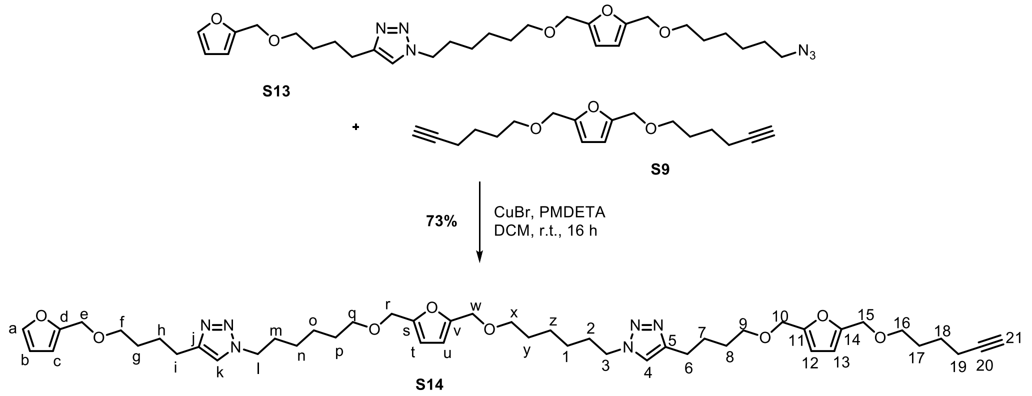

S13 (200 mg, 0.36 mmol, 1.0 eq.) and **S9** (508 mg, 1.80 mmol, 5.0 eq.) were dissolved in DCM (3.6 mL) in a microwave vial. Separately, PMDETA (38 µL, 0.18 mmol, 0.5 eq.) was dissolved in DCM (1 mL) in a microwave vial. The two mixtures were degassed via three freeze-thaw-pump cycles. In a third microwave vial was added CuBr (10 mg, 0.072 mmol, 0.2 eq.) under N_2. The PMDETA mixture was transferred via cannula onto the CuBr; the resulting mixture was stirred until all solids had dissolved. The mixture was then degassed via a single freeze-thaw-pump cycle before being transferred via cannula onto the reaction mixture. The reaction mixture was stirred at r.t. overnight before being condensed. The crude material was purified via column chromatography (SiO_2, PE/EtOAc, 1/2) to give the pure product as a colourless viscous oil (220 mg, 0.26 mmol, 73% yield).

^{1}H NMR: (500 MHz, CD_3CN, 298 K) δ = 7.47 – 7.44 (m, 3H, $H_{a,k,4}$), 6.37 (dd, *J* = 3.3, 1.8 Hz, 1H, H_b), 6.33 (d, *J* = 3.2 Hz, 1H, H_c), 6.27 (d, *J* = 5.0 Hz, 4H, $H_{t,u,12,13}$), 4.37 (s, 2H, H_e), 4.36 – 4.34 (s, s, 8H, $H_{r,w,10,15}$), 4.26 (t, *J* = 7.1 Hz, 4H, $H_{l,3}$), 3.47 – 3.42 (m, 6H, $H_{f,9,16}$), 3.40 (t, *J* = 6.5 Hz, 4H, $H_{q,x}$), 2.64 (td, *J* = 7.5, 1.6 Hz, 4H, $H_{i,6}$), 2.19 – 2.14 (m, 3H, $H_{19,21}$), 1.81 (p, *J* = 7.3 Hz, 4H, $H_{m,2}$), 1.68 – 1.46 (m, 16H, $H_{g,h,p,y,7,8,17,18}$), 1.36 – 1.29 (m, 4H, $H_{o,z}$), 1.28 – 1.21 (m, 4H, $H_{n,1}$).
^{13}C NMR: (126 MHz, CD_3CN, 298 K) δ = 153.48, 153.45, 153.40 ($C_{d,s,v,11,14}$), 148.40 ($C_{j,5}$), 143.76 (C_a), 122.10 ($C_{k,4}$), 111.29 (C_b), 110.70, 110.66, 110.65 ($C_{t,u,12,13}$), 109.95 (C_c), 85.26 (C_{20}), 70.63, 70.47, 70.42, 70.14 ($C_{f,q,x,9,16}$), 69.80 (C_{21}), 65.20 ($C_{r,w,10,15}$), 65.07 (C_e), 50.53 ($C_{l,3}$), 30.89 ($C_{m,2}$), 30.13, 29.82, 29.38, 26.92, 26.04 ($C_{g,h,p,y,7,8,17,18}$), 26.88 ($C_{n,1}$), 26.25 ($C_{o,z}$), 25.93 ($C_{i,6}$), 18.51 (C_{19}).
HRMS-ESI(+): 867.4952 $[M+Na]^+$, calculated for $C_{47}H_{68}O_8N_6Na$: 867.4991.

4.1.20 Synthesis of S15

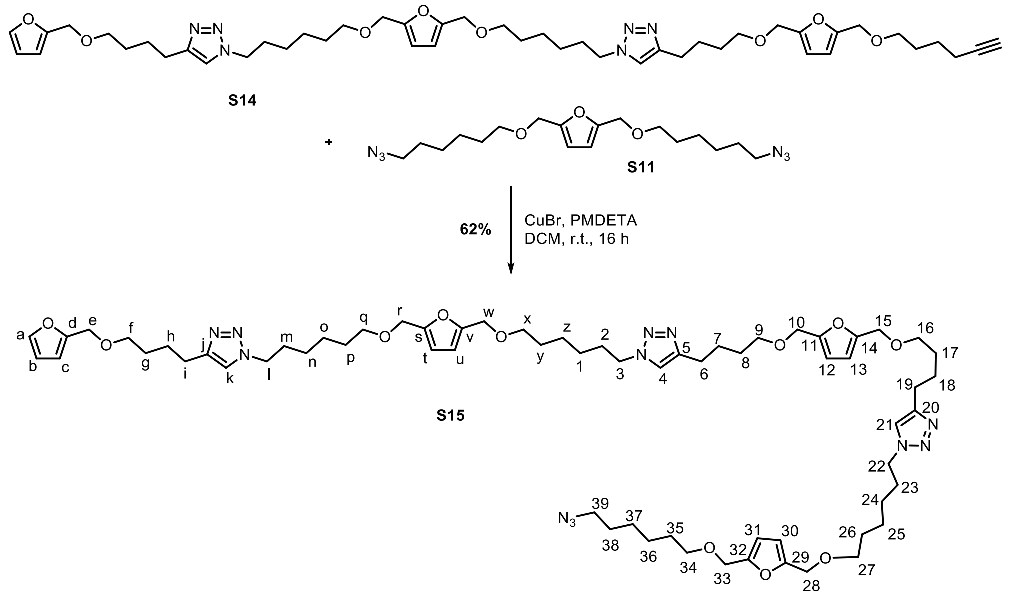

S14 (200 mg, 0.24 mmol, 1.0 eq.) and **S11** (450 mg, 1.18 mmol, 5.0 eq.) were dissolved in DCM (2.4 mL) in a microwave vial. Separately, PMDETA (25 µL, 0.12 mmol, 0.5 eq.) was dissolved in DCM (1 mL) in a microwave vial. The two mixtures were degassed via three freeze-thaw-pump cycles. In a third microwave vial was added CuBr (7 mg, 0.047 mmol, 0.2 eq.) under N_2. The PMDETA mixture was transferred via cannula onto the CuBr; the resulting mixture was stirred until all solids had dissolved. The mixture was then degassed via a single freeze-thaw-pump cycle before being transferred via cannula onto the reaction mixture. The reaction mixture was stirred at r.t. overnight before being condensed. The crude material was purified via column chromatography (SiO_2, DCM/MeOH, 100/4) to give the pure product as an off-white solid (180 mg, 0.15 mmol, 62% yield).

^{1}H NMR: (500 MHz, CD_3CN, 298 K) δ = 7.47 – 7.44 (m, 4H, $H_{a,k,4,21}$), 6.37 (dd, J = 3.3, 1.9 Hz, 1H, H_b), 6.33 (d, J = 3.2 Hz, 1H, H_c), 6.27 (d, J = 2.6 Hz, 6H, $H_{t,u,12,13,30,31}$), 4.37 (s, 2H, H_e), 4.35 – 4.34 (m, 12H, $H_{r,w,10,15,28,33}$), 4.25 (t, J = 7.1 Hz, 6H, $H_{l,3,22}$), 3.46 – 3.37 (m, 14H, $H_{f,q,x,9,16,27,34}$), 3.26 (t, J = 7.0 Hz, 2H, H_{39}), 2.63 (t, J = 7.5 Hz, 6H, $H_{i,6,19}$), 1.85 – 1.77 (m, 6H, $H_{m,2,23}$), 1.67 – 1.61 (m, 6H, $H_{h,7,18}$), 1.59 – 1.46 (m, 16H, $H_{g,p,y,8,17,26,35,38}$), 1.35 – 1.29 (m, 10H, $H_{o,z,25,36,37}$), 1.27 – 1.22 (m, 6H, $H_{n,1,24}$).
^{13}C NMR: (126 MHz, CD_3CN, 298 K) δ = 153.46 ($C_{d,s,v,11,14,29,32}$), 148.40 ($C_{j,5,20}$), 143.77 (C_a), 122.11 ($C_{k,4,21}$), 111.30 (C_b), 110.67, 110.65 ($C_{t,u,12,13,30,31}$), 109.96 (C_c), 70.69, 70.63, 70.46, 70.43 ($C_{f,q,x,9,16,27,34}$), 65.20 ($C_{r,w,10,15,28,33}$), 65.08 (C_e), 52.07 (C_{39}), 50.53 ($C_{l,3,22}$), 30.90 ($C_{m,2,23}$), 30.23, 30.14, 29.83, 29.40 ($C_{g,p,y,8,17,26,35,38}$), 27.19, 26.43, 26.26 ($C_{o,z,25,36,37}$), 26.93, 26.89 ($C_{h,7,18,n,1,24}$), 25.94 ($C_{i,6,19}$).
HRMS-ESI(+): 1245.7328 $[M+H]^+$, calculated for $C_{65}H_{98}O_{11}N_{12}Na$: 1245.7370.

4.1.21 Synthesis of S16

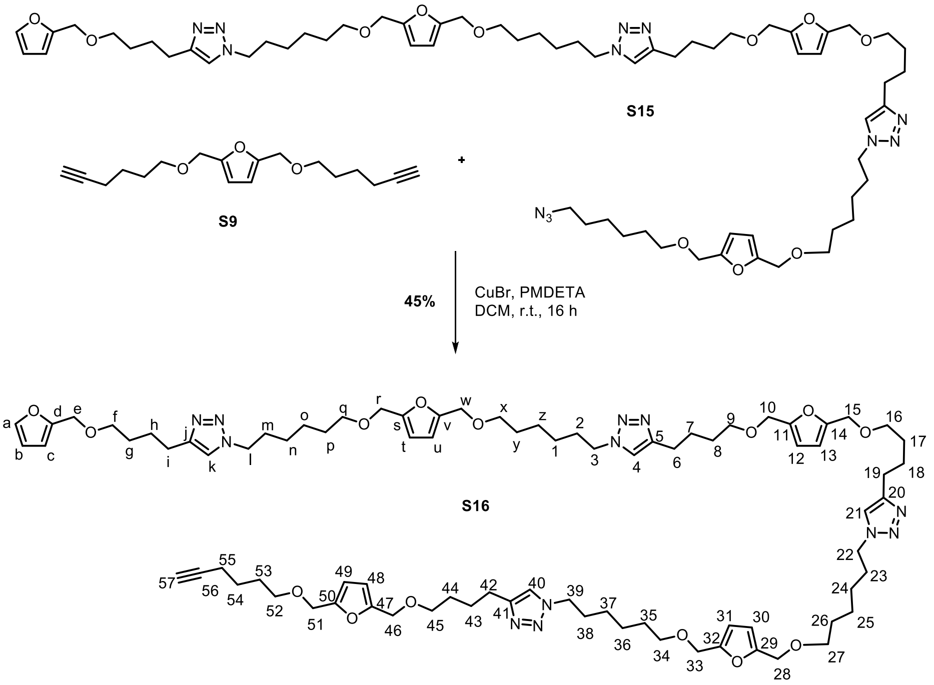

S15 (150 mg, 0.123 mmol, 1.0 eq.) and **S9** (177 mg, 0.61 mmol, 5.0 eq.) were dissolved in DCM (1.2 mL) in a microwave vial. Separately, PMDETA (13 µL, 0.061 mmol, 0.5 eq.) was dissolved in DCM (1 mL) in a microwave vial. The two mixtures were degassed via three freeze-thaw-pump cycles. In a third microwave vial was added CuBr (4 mg, 0.025 mmol, 0.2 eq.) under N_2. The PMDETA mixture was transferred via cannula onto the CuBr; the resulting mixture was stirred until all solids had dissolved. The mixture was then degassed via a single freeze-thaw-pump cycle before being transferred via cannula onto the reaction mixture. The reaction mixture was stirred at r.t. overnight before being condensed. Purification of the crude material was attempted via column chromatography (SiO_2, DCM/MeOH, 100/5) but the desired product could not be separated from the large excess of **S9**. The partially purified material was dissolved in minimal DCM before being massively diluted with Et_2O resulting in a large amount of precipitate forming. The mixture was filtered; the filtered solid was thoroughly washed with Et_2O and collected as the pure product as a beige solid (83 mg, 0.055 mmol, 45% yield).

^{1}H NMR: (500 MHz, $CDCl_3$, 298 K) δ = 7.39 – 7.37 (m, 1H, H_a), 7.25 – 7.22 (s, s, 4H, $H_{k,4,21,40}$), 6.32 (dd, J = 3.3, 1.9 Hz, 1H, H_b), 6.28 (d, J = 3.2 Hz, 1H, H_c), 6.26 – 6.22 (m, 8H, $H_{t,u,12,13,30,31,48,49}$), 4.41 (s, 2H, H_e), 4.40 – 4.37 (s, s, s, 16H, $H_{r,w,10,15,28,33,46,51}$), 4.27 (t, J = 7.2 Hz, 8H, $H_{l,3,22,39}$), 3.50 – 3.46 (m, 10H, $H_{f,9,16,45,52}$), 3.43 (t, J = 6.5 Hz, 8H, $H_{q,x,27,34}$), 2.70 (td, J = 7.5, 3.4 Hz, 8H, $H_{i,6,19,42}$), 2.18 (td, J = 7.0, 2.7 Hz, 2H, H_{55}), 1.92 (t, J = 2.6 Hz, 1H, H_{57}), 1.85 (p, J = 7.3 Hz, 8H, $H_{m,2,23,38}$), 1.76 – 1.52 (m, 28H, $H_{g,h,p,y,7,8,17,18,26,35,43,44,53,54}$), 1.41 – 1.26 (m, 16H, $H_{n,o,z,1,24,25,36,37}$).

^{13}C NMR: (126 MHz, $CDCl_3$, 298 K) δ = 152.30, 152.28, 152.27, 152.10 ($C_{d,s,v,11,14,29,32,47,50}$), 148.08, 148.06 ($C_{j,5,20,42}$), 142.78 ($C_a$), 120.63, 120.60 ($C_{k,4,21,40}$), 110.33 ($C_b$), 109.86 ($C_{t,u,12,13,30,31,48,49}$), 109.15 ($C_c$), 84.41 ($C_{56}$), 70.19, 70.10, 70.04, 69.73 ($C_{f,q,x,9,16,27,34,45,52}$), 68.55 ($C_{57}$), 65.00, 64.97 ($C_{r,w,10,15,28,33,46,51}$), 64.87 ($C_e$), 50.14 ($C_{l,3,22,39}$), 30.39 ($C_{m,2,23,38}$), 29.47, 29.26, 28.70, 26.41, 26.14, 25.68, 25.20 ($C_{g,h,o,p,y,z,7,8,17,18,24,25,26,35,36,37,43,44,53,54}$), 25.53 ($C_{i,6,19,42}$), 18.26 ($C_{55}$).

HRMS-ESI(+): 1533.9056 $[M+Na]^+$, calculated for $C_{83}H_{122}O_{14}N_{12}Na$: 1533.9096.

4.1.22 Synthetic Routes to 3-Cargo Diels-Alder Cargo Compartments

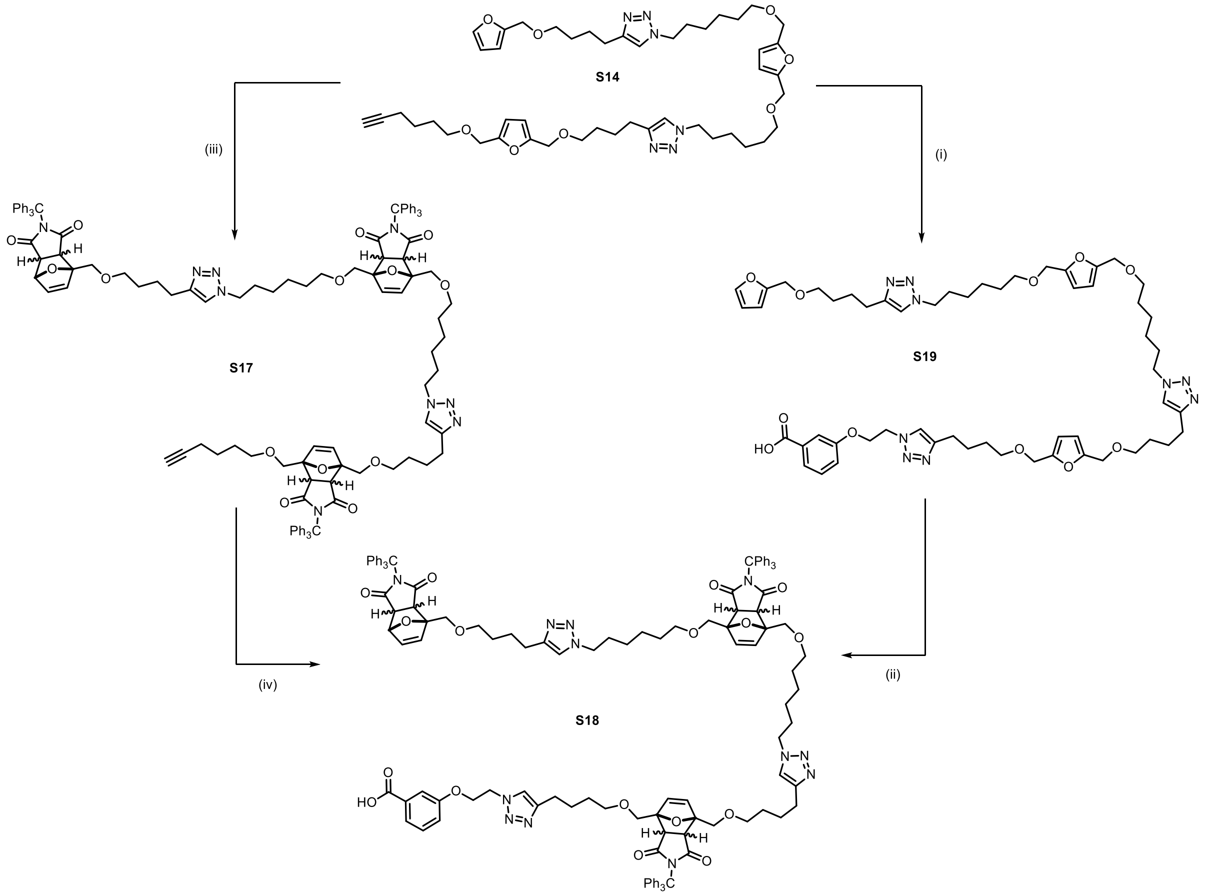

Figure S6. Synthetic routes to **S18**. Conditions: (i) **S7**, CuBr, PMDETA, DCM, r.t., 16 h, 74% yield; (ii) **2**, MeCN/DCM, 50 °C, 72 h, 69% yield (**S18d**); (iii) **2**, MeCN/DCM, 50 °C, 72 h, 18 %, 36% and 18% yields for **S17a**, **S17b** and **S17c** respectively; (iv) **S7**, $CuSO_4$, sodium ascorbate, H_2O/THF, r.t., 16 h, 45%, 60% and 41% yields for **S18a**, **S18b** and **S18c** respectively.

4.1.23 Synthesis of S17

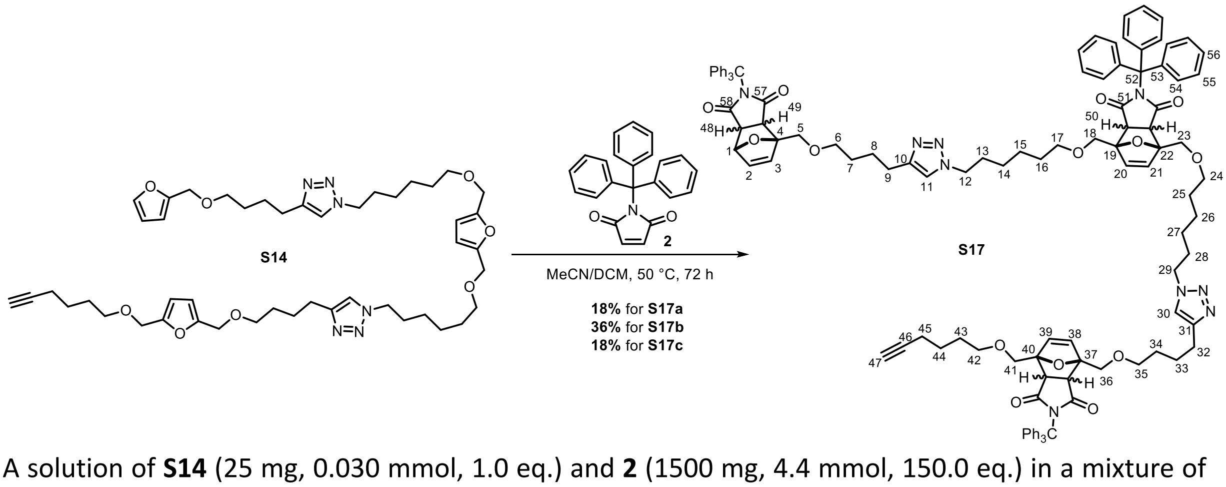

A solution of **S14** (25 mg, 0.030 mmol, 1.0 eq.) and **2** (1500 mg, 4.4 mmol, 150.0 eq.) in a mixture of acetonitrile (4 mL) and DCM (4 mL) was stirred at 50 °C for 72 h. The solvent was removed under vacuum and the residue purified by flash column chromatography (SiO_2, DCM/Et_2O, 2/1) to yield **S17a** (10 mg, 0.005 mmol, 18% yield, 96% furan conversion), **S17b** (20 mg, 0.011 mmol, 36% yield, 96% furan conversion) and **S17c** (10 mg, 0.005 mmol, 18% yield, 91% furan conversion) as faint yellow powders.

S17 was isolated as three separate mixtures of stereoisomers with unreacted furan units (compounds **S17a**, **S17b**, and **S17c**). When distinguishable, *exo* and *endo* adducts are denoted as X_x and X_x' respectively. Unreacted furan units are denoted as X_x''.

Compound **S17a**:

The product is a mixture of stereoisomers including *endo-endo-exo* and *endo-endo-endo* with respect to the furan-maleimide Diels-Alder moieties; 4% of the furan units remain unreacted. Other minor stereoisomers are present in minute amount and can't be individually quantified.

^{1}H NMR (500 MHz, Acetone-d_6, 298 K) δ = 7.67 – 7.61 (m, 2H, $H_{11,30}$), 7.52 – 7.40 (m, 18H, H_{54}), 7.28 – 7.18 (m, 18H, H_{55}), 7.18 – 7.08 (m, 9H, H_{56}), 6.61 – 6.51 (m, 1.67H, $H_{2,3,20,21,38,39}$), 6.39 – 6.36 (m, *J* = 2.8 Hz, 0.10H, H_{2}''), 6.35 (d, *J* = 3.3 Hz, 0.10H, H_{3}''), 6.31 – 6.28 (m, 0.05H, $H_{20,21,38,39}''$), 5.50 (d, *J* = 5.9 Hz, 0.12H, H_{2}'), 5.42 (d, *J* = 5.7 Hz, 0.16H, H_{3}'), 5.38 (d, *J* = 6.2 Hz, 3.85H, $H_{20,21,38,39}'$), 5.26 (d, *J* = 1.7 Hz, 0.73H, H_{1}), 5.00 (dd, *J* = 5.5, 1.6 Hz, 0.14H, H_{1}'), 4.41 – 4.36 (m, 0.35H, $H_{5,18,23,36,41}''$), 4.36 – 4.26 (m, 4H, $H_{12,29}$), 4.15 (d, *J* = 11.6 Hz, 0.96H, $H_{5,18,23,36,41}$), 3.95 – 3.77 (m, 9.06H, $H_{5,18,23,36,41}$ *and* $H_{5,18,23,36,41}'$), 3.74 – 3.71 (m, 0.19H, H_{48}'), 3.69 – 3.60 (m, 4.82H, H_{6} *and* H_{50}'), 3.59 – 3.40 (m, 9.72H, $H_{6,17,24,35,42}$ *and* H_{49}'), 2.90 – 2.78 (m, 14.88H, $H_{48,49,50}$, overlapped with water peak), 2.70 (t, *J* = 7.5 Hz, 2H, H_{9}), 2.66 (t, *J* = 7.5 Hz, 2H, H_{32}), 2.32 (t, *J* = 2.7 Hz, 1H, H_{47}), 2.16 (td, *J* = 7.0, 2.7 Hz, 2H, H_{45}'), 1.85 (p, *J* = 7.1 Hz, 4H, $H_{13,28}$), 1.78 – 1.47 (m, 16H, $H_{7,8,33,44,34,43,16,25}$), 1.41 – 1.31 (m, 8H, $H_{14,27,15,26}$, overlapped with grease peak).
^{13}C NMR (126 MHz, Acetone-d_6, 298 K) δ = 175.61, 175.57, 175.53 (C_{51}'), 175.44 (C_{58}), 174.31 (C_{57}), 174.02 (C_{51}), 148.10, 148.05 ($C_{10,31}$), 143.55, 143.45, 143.43 (C_{53}), 138.75 (C_{3}), 137.46 (C_{2}), 136.39, 136.36, 136.34 ($C_{20,21,38,39}'$), 135.86, 135.79 ($C_{2,3}'$), 129.51 – 129.17 (C_{54}), 128.20, 128.04 (C_{55}), 127.17, 127.05 (C_{56}), 121.92 ($C_{11,30}$), 111.06 (C_{2}''), 110.38 ($C_{20,21,38,39}''$), 109.70 (C_{3}''), 92.25, 92.23, 92.19 ($C_{19,22,37,40}'$), 92.07 (C_{4}), 84.87 (C_{46}), 82.29 (C_{1}), 80.10 (C_{1}'), 74.46 (C_{52}'), 74.09 (C_{52}), 72.13 (C_{6}), 71.93, 71.91, 71.58 ($C_{17,24,35,42}$), 70.05 (C_{47}), 69.57, 69.51, 69.45 ($C_{18,23,36,41}'$), 69.01 (C_{5}), 65.06 ($C_{5,18,23,36,41}''$), 51.30 (C_{50}), 50.41 (C_{48}), 50.26 ($C_{12,29}$), 48.93 (C_{49}), 48.37 (C_{48}'), 48.04, 48.01, 47.95 (C_{50}'), 46.25 (C_{49}'), 31.00 ($C_{13,28}$), 30.03 – 29.70 ($C_{16,25,34}$, overlapped with solvent peak), 26.93, 26.89, 26.85 ($C_{8,33,14,27,7,43}$), 26.26 ($C_{15,26}$), 26.04, 26.01, 25.99 ($C_{44,9,32}$), 18.48 (C_{45}).
HRMS-ESI (+): 1884.8827 $[M+Na]^+$, calculated for $C_{116}H_{119}N_9O_{14}Na^+$: 1884.8769.

Compound **S17b**:

The product is a mixture of stereoisomers including *exo-endo-exo*, *endo-exo-exo*, *exo-endo-endo* and *endo-exo-endo* with respect to the furan-maleimide Diels-Alder moieties; 4% of the furan units remain unreacted. Other minor stereoisomers are present in minute amount and can't be individually quantified.

^{1}H NMR (500 MHz, Acetone-d_6, 298 K) δ = 7.68 – 7.60 (m, 2H, $H_{11,30}$), 7.52 – 7.36 (m, 18H, H_{54}), 7.29 – 7.18 (m, 18H, H_{55}), 7.18 – 7.09 (m, 9H, H_{56}), 6.61 – 6.50 (m, 3.31H, $H_{2,3,20,21,38,39}$), 6.39 – 6.36 (m, 0.05H, H_{2}''), 6.36 – 6.34 (m, 0.04H, H_{3}''), 6.30 (d, *J* = 4.1 Hz, 0.30H, $H_{20,21,38,39}''$), 5.50 (d, *J* = 5.3 Hz, 0.16H, H_{2}'), 5.41 (d, *J* = 5.8 Hz, 0.19H, H_{3}'), 5.38 (d, *J* = 6.6 Hz, 2.22H, $H_{20,21,38,39}'$), 5.25 (d, *J* = 1.8 Hz, 0.82H, H_{1}), 5.00 (dd, *J* = 5.6, 1.7 Hz, 0.17H, H_{1}'), 4.41 – 4.35 (m, 0.77H, $H_{5,18,23,36,41}''$), 4.35 – 4.27 (m, 4H, $H_{12,29}$), 4.19 – 4.11 (m, 2.55H, $H_{5,18,23,36,41}$), 3.96 – 3.77 (m, 7.44H, $H_{5,18,23,36,41}$ *and* $H_{5,18,23,36,41}'$), 3.74 – 3.60 (m, 5.05H, H_{6} *and* $H_{48,50}'$), 3.60 – 3.38 (m, 8.71H, $H_{6,17,24,35,42}$ *and* H_{49}'), 2.90 – 2.77 (m, 13.46H, $H_{48,49,50}$, overlapped with water peak), 2.73 – 2.62 (m, 4H, $H_{9,32}$), 2.35 – 2.31 (m, 1H, H_{47}), 2.21 (td, *J* = 7.1, 2.7 Hz, 0.94H, H_{45}), 2.16 (td, *J* = 6.9, 2.5 Hz, 1.28H, H_{45}'), 1.90 – 1.80 (m, 4H, $H_{13,28}$), 1.80 – 1.40 (m, 16H, $H_{7,8,33,44,34,43,16,25}$), 1.39 – 1.29 (m, 8H, $H_{14,27,15,26}$, overlapped with grease peak).
^{13}C NMR (126 MHz, Acetone-d_6, 298 K) δ = 175.61, 175.56, 175.54 – 175.49 (C_{51}'), 175.43 (C_{58}), 174.31 (C_{57}), 174.05 – 173.97 (C_{51}), 153.38 ($C_{19,22,37,40}''$), 148.09, 148.07, 148.02 ($C_{10,31}$), 143.55, 143.51 –

143.37 (C_{53}), 138.85 – 138.64 ($C_{3,20,21,38,39}$), 137.46 (C_2), 136.38, 136.36, 136.33 ($C_{20,21,38,39}'$), 135.86, 135.78 ($C_{2,3}'$), 129.51 – 129.16 (C_{54}), 128.22, 128.19, 128.04 (C_{55}), 127.26, 127.16, 127.04 (C_{56}), 121.92, 121.88 ($C_{11,30}$), 110.43, 110.39 ($C_{20,21,38,39}''$), 92.25, 92.22, 92.19 ($C_{19,22,37,40}'$), 92.13, 92.11, 92.06 ($C_{4,19,22,37,40}$), 84.94 (C_{46}), 84.86 (C_{46}'), 82.28 (C_1), 80.09 (C_1'), 74.46, 74.44 – 74.41 (C_{52}'), 74.13 – 74.05 (C_{52}), 72.22 – 72.15, 72.13, 71.96 – 71.86, 71.82, 71.57 ($C_{6,17,24,35,42}$), 70.05 (C_{47}), 69.56, 69.51, 69.45 ($C_{18,23,36,41}'$), 69.19 – 69.12, 69.08 ($C_{18,23,36,41}$), 69.01 (C_5), 65.19 ($C_{5,18,23,36,41}''$), 51.30 (C_{50}), 50.41 (C_{48}), 50.30 – 50.22 ($C_{12,29}$), 48.93 (C_{49}), 48.37 (C_{48}'), 48.03, 48.00, 47.95 (C_{50}'), 46.25 (C_{49}'), 31.05, 31.00 ($C_{13,28}$), 30.04 – 29.74 ($C_{16,25,34}$, overlapped with solvent peak), 26.95, 26.92, 26.88, 26.85 ($C_{8,33,14,27,7,43}$), 26.25 ($C_{15,26}$), 26.06, 26.04, 26.01, 25.98 ($C_{44,9,32}$), 18.54 (C_{45}), 18.48 (C_{45}').
HRMS-ESI (+): 1884.8904 $[M+Na]^+$, calculated for $C_{116}H_{119}N_9O_{14}Na^+$: 1884.8769.

Compound **S17c**:

The product is a mixture of stereoisomers including *exo-exo-exo* and *exo-exo-endo* with respect to the furan-maleimide Diels-Alder moieties; 9% of the furan units remain unreacted. Other minor stereoisomers are present in minute amount and can't be individually quantified.

^{1}H NMR (500 MHz, Acetone-d_6, 298 K) δ = 7.67 – 7.63 (m, 2H, $H_{11,30}$), 7.51 – 7.40 (m, 18H, H_{54}), 7.32 – 7.18 (m, 18H, H_{55}), 7.18 – 7.07 (m, 9H, H_{56}), 6.61 – 6.50 (m, 4.72H, $H_{2,3,20,21,38,39}$), 6.39 – 6.36 (m, 0.02H, H_2''), 6.36 – 6.34 (m, 0.01H, H_3''), 6.32 – 6.28 (m, 0.53H, $H_{20,21,38,39}''$), 5.50 (d, J = 5.7 Hz, 0.18H, H_2'), 5.41 (d, J = 5.9 Hz, 0.19H, H_3'), 5.38 (d, J = 5.8 Hz, 0.33H, $H_{20,21,38,39}'$), 5.28 – 5.22 (m, 0.82H, H_1), 4.99 (d, J = 5.2 Hz, 0.19H, H_1'), 4.40 – 4.35 (m, 1.19H, $H_{5,18,23,36,41}''$), 4.35 – 4.26 (m, 4H, $H_{12,29}$), 4.20 – 4.09 (m, 4.04H, $H_{5,18,23,36,41}$), 3.97 – 3.78 (m, 5.18H, $H_{5,18,23,36,41}$ *and* $H_{5,18,23,36,41}'$), 3.73 – 3.59 (m, 4.62H, H_6 *and* $H_{48,50}'$), 3.59 – 3.36 (m, 7.17H, $H_{6,17,24,35,42}$ *and* H_{49}'), 2.94 – 2.75 (m, 19.24H, $H_{48,49,50}$, overlapped with water peak), 2.74 – 2.62 (m, 4H, $H_{9,32}$), 2.35 – 2.30 (m, 1H, H_{47}), 2.21 (td, J = 7.1, 2.6 Hz, 1.55H, H_{45}), 2.17 (td, J = 7.0, 2.2 Hz, 0.64H, H_{45}'), 1.92 – 1.81 (m, 4H, $H_{13,28}$), 1.79 – 1.39 (m, 16H, $H_{7,8,33,44,34,43,16,25}$), 1.39 – 1.30 (m, 8H, $H_{14,27,15,26}$, overlapped with grease peak).
^{13}C NMR (126 MHz, Acetone-d_6, 298 K) δ = 175.61, 175.56, 175.52 (C_{51}'), 175.42 (C_{58}), 174.30 (C_{57}), 174.06 – 173.98 (C_{51}), 153.41, 153.37, 153.31 ($C_{19,22,37,40}''$), 148.18 – 147.92 ($C_{10,31}$), 143.53, 143.45 (C_{53}), 138.76, 138.74, 138.68 ($C_{3,20,21,38,39}$), 137.45 (C_2), 136.38, 136.35, 136.33 (, $C_{20,21,38,39}'$), 135.85, 135.77 ($C_{2,3}'$), 129.73 – 129.07 (C_{54}), 128.21, 128.19, 128.06 – 128.00 (C_{55}), 127.26, 127.16, 127.07 – 126.99 (C_{56}), 121.90 (, $C_{11,30}$), 110.43, 110.39 ($C_{20,21,38,39}''$), 92.30, 92.24, 92.22, 92.18 ($C_{19,22,37,40}'$), 92.12, 92.10, 92.08 – 92.02 ($C_{4,19,22,37,40}$), 84.93 (C_{46}), 82.27 (C_1), 80.09 (C_1'), 74.46, 74.42 (C_{52}'), 74.10, 74.09, 74.07 (C_{52}), 72.18, 72.15, 72.11, 71.92, 71.90, 71.81 ($C_{6,17,24,35,42}$), 70.05 (C_{47}), 69.67 – 69.27 ($C_{18,23,36,41}'$), 69.22 – 69.03 ($C_{18,23,36,41}$), 69.00 (C_5), 65.18 ($C_{5,18,23,36,41}''$), 51.30 (C_{50}), 50.40 (C_{48}), 50.28 (, $C_{12,29}$), 48.92 (C_{49}), 48.36 (C_{48}'), 48.03, 48.00, 47.94 (C_{50}'), 46.24 (C_{49}'), 31.04 ($C_{13,28}$), 30.05 – 29.64 ($C_{16,25,34}$, overlapped with solvent peak), 26.92 ($C_{8,33,14,27,7,43}$), 26.29, 26.23 ($C_{15,26}$), 26.05, 26.02 ($C_{44,9,32}$), 18.54 (C_{45}).
HRMS-ESI (+): 1884.8914 $[M+Na]^+$, calculated for $C_{116}H_{119}N_9O_{14}Na^+$: 1884.8769.

4.1.24 Synthesis of S18a, S18b, and S18c

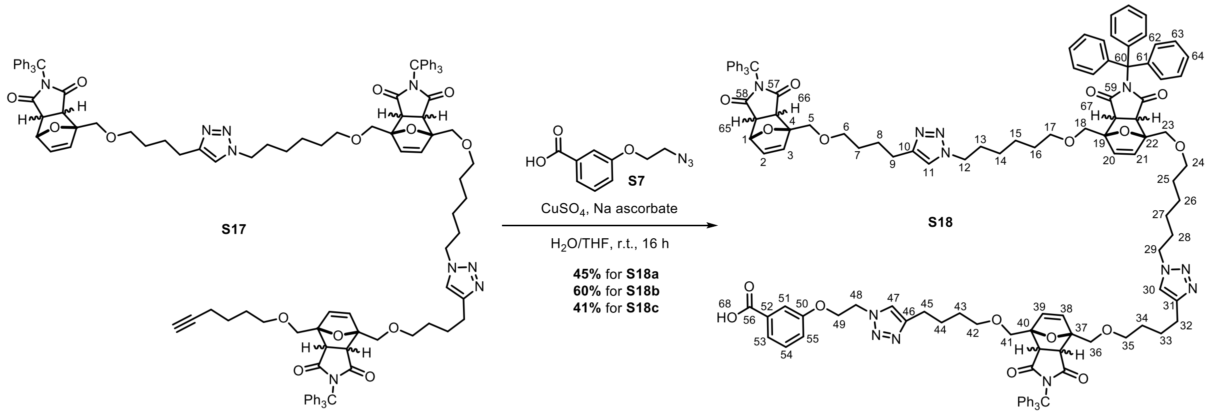

General Experimental Procedure:

A solution of $CuSO_4$, **S7** and either **S17a**, **S17b** or **S17c** in a mixture of THF (1.5 mL) and water (0.5 mL) was degassed by bubbling N_2 through it for 3 min; sodium ascorbate was then added. The mixture was stirred for 16 h at room temperature before aqueous HCl solution (0.1 N) was added to adjust the pH of the mixture to ~3. DCM (10 mL) was added and the mixture washed by aqueous EDTA solution (0.25 M, pH 7, 2 x 10 mL) and brine (2 x 10 mL). The organic solvent was separated and dried with magnesium sulfate. The mixture was filtered before being concentrated under vacuum. The residue was purified by preparative TLC (500 µm, DCM/MeOH, 20/1, eluted twice) to give either **S18a**, **S18b** or **S18c** as white powders.

S18a, **S18b** and **S18c** were each isolated as mixtures of stereoisomers. When distinguishable, *exo* and *endo* adducts are denoted as X_x and X_x' respectively.

Compound **S18a**:

Synthesis followed the general experimental procedure. **S17a** (10 mg, 5.4 µmol, 1.0 eq.), **S7** (1.3 mg, 6.4 µmol, 1.2 eq.), $CuSO_4$ (8.6 mg, 54 µmol, 10.0 eq.) and sodium ascorbate (53 mg, 268 µmol, 50.0 eq.) were used in the reaction to yield **S18a** (5 mg, 2.4 µmol, 45% yield).

The product is a mixture of stereoisomers including *endo-endo-exo* and *endo-endo-endo* with respect to the furan-maleimide Diels-Alder moieties; 4% of the furan units remain unreacted. Other minor stereoisomers are present in minute amount and can't be individually quantified.

^{1}H NMR (500 MHz, Acetone-d_6, 298 K) δ = 7.80 (s, 1H, H_{47}), 7.69 – 7.62 (m, 3H, $H_{11,30,53}$), 7.58 – 7.54 (m, H_{51}), 7.49 – 7.37 (m, 19H, $H_{62,54}$), 7.27 – 7.18 (m, 18H, H_{63}), 7.18 – 7.09 (m, 10H, $H_{64,55}$), 6.58 (dd, J = 5.7, 1.7 Hz, 0.83H, H_2), 6.54 (d, J = 5.6 Hz, 0.89H, $H_{3,20,21,38,39}$), 5.50 (d, 0.13H, H_2'), 5.41 (d, J = 5.7 Hz, 0.16H, H_3'), 5.40 – 5.36 (m, 3.95H, $H_{20,21,38,39}'$), 5.25 (d, J = 1.7 Hz, 0.85H, H_1), 5.00 (dd, J = 5.4, 1.4 Hz, 0.14H, H_1'), 4.77 (t, J = 5.1 Hz, 2H, H_{48}), 4.49 (t, J = 5.1 Hz, 2H, H_{49}), 4.35 – 4.25 (m, 4H, $H_{12,29}$), 4.14 (d, J = 11.6 Hz, 0.91H, $H_{5,18,23,36,41}$), 3.91 – 3.76 (m, 9.15H, $H_{5,18,23,36,41}$ *and* $H_{5,18,23,36,41}'$), 3.68 – 3.59 (m, 4.94H, H_6 *and* $H_{65,67}'$), 3.59 – 3.38 (m, 10.12H, $H_{6,35,42,17,24}$ *and* H_{66}'), 2.96 – 2.73 (m, 28.94H, $H_{65,66,67}$, overlapped with water peak), 2.70 (t, J = 7.5 Hz, 2H, H_9), 2.65 (t, J = 7.5 Hz, 4H, $H_{32,45}$), 1.89 – 1.80 (m, 4H, $H_{13,28}$), 1.78 – 1.45 (m, 16H, $H_{7,8,33,44,34,43,16,25}$), 1.39 – 1.33 (m, 8H, $H_{14,27,15,26}$).
^{13}C NMR (126 MHz, Acetone-d_6, 298 K) δ = 175.63, 175.57 (C_{59}'), 175.45 (C_{58}), 174.32 (C_{57}), 167.42 (C_{56}), 159.31 (C_{50}), 148.16, 148.10, 148.06 ($C_{10,31,46}$), 143.55, 143.46, 143.43 (C_{61}), 138.75 ($C_{3,20,21,38,39}$), 137.47 (C_2), 136.37 ($C_{20,21,38,39}'$), 133.10 (C_{52}), 130.57 (C_{54}), 129.47 – 129.23 (C_{62}), 128.20, 128.05 (C_{63}), 127.17, 127.05 (C_{64}), 123.30 (C_{53}), 122.90 (C_{47}), 122.02, 121.96 ($C_{11,30}$), 120.19 (C_{55}), 116.16 (C_{51}), 92.24, 92.19 ($C_{19,22,37,40}'$), 92.07 ($C_{4,19,22,37,40}$), 82.29 (C_1), 74.47 (C_{60}'), 74.09 (C_{60}), 72.13, 71.94, 71.90, 71.88 ($C_{6,17,24,35,42}$), 69.50, 69.49, 69.45 ($C_{18,23,36,41}'$), 69.01 (C_5), 67.71 (C_{49}), 50.41 (C_{65}), 50.31, 50.28 ($C_{12,29}$),

49.98 (C_{48}), 48.93 (C_{66}), 48.02, 47.95 (C_{67}′), 30.99 ($C_{13,28}$), 30.06 – 29.75 ($C_{16,25,34}$, overlapped with solvent peak), 26.93, 26.88, 26.84, 26.80 ($C_{44,8,33,14,27,7,43}$), 26.25 ($C_{15,26}$), 26.02, 25.92 ($C_{45,9,32}$).
HRMS-ESI (-): 2067.9439 $[M-H]^-$, calculated for $C_{125}H_{127}N_{12}O_{17}^-$: 2067.9448

Compound **S18b**:

Synthesis followed the general experimental procedure. **S17b** (15 mg, 8.1 µmol, 1.0 eq.), **S7** (2.0 mg, 9.7 µmol, 1.2 eq.), $CuSO_4$ (13 mg, 81 µmol, 10.0 eq.) and sodium ascorbate (80 mg, 402 µmol, 50.0 eq.) were used in the reaction to yield **S18b** (10 mg, 4.8 µmol, 60% yield).

The product is a mixture of stereoisomers including *exo-endo-exo*, *endo-exo-exo*, *exo-endo-endo* and *endo-exo-endo* with respect to the furan-maleimide Diels-Alder moieties; 4% of the furan units remain unreacted. Other minor stereoisomers are present in minute amount and can't be individually quantified.

^{1}H NMR (500 MHz, Acetone-d_6 , 298 K) δ = 7.81 (s, 0.47H, H_{47}), 7.80 (s, 0.51H, H_{47}′), 7.67 – 7.62 (m, 3H, $H_{11,30,53}$), 7.58 – 7.54 (m, 1H, H_{51}), 7.49 – 7.36 (m, 19H, $H_{62,54}$), 7.29 – 7.17 (m, 18H, H_{63}), 7.17 – 7.07 (m, 10H, $H_{64,55}$), 6.57 (dd, *J* = 6.2, 1.4 Hz, 0.84H, H_2), 6.55 – 6.51 (m, 2.68H, $H_{3,20,21,38,39}$), 5.50 (d, *J* = 5.9 Hz, 0.16H, H_2′), 5.41 (d, *J* = 5.8 Hz, 0.18H, H_3′), 5.37 (d, *J* = 2.0 Hz, 2.28H, $H_{20,21,38,39}$′), 5.25 (d, *J* = 1.7 Hz, 0.86H, H_1), 4.99 (d, *J* = 5.5 Hz, 0.17H, H_1′), 4.81 – 4.74 (m, 2H, H_{48}), 4.53 – 4.45 (m, 2H, H_{49}), 4.35 – 4.26 (m, 4H, $H_{12,29}$), 4.20 – 4.10 (m, 2.83H, $H_{5,18,23,36,41}$), 3.94 – 3.76 (m, 7.70H, $H_{5,18,23,36,41}$ *and* $H_{5,18,23,36,41}$′), 3.73 – 3.60 (m, 5.48H, H_6 *and* $H_{65,67}$′), 3.59 – 3.38 (m, 8.68H, $H_{6,35,42,17,24}$ *and* H_{66}′), 2.91 – 2.76 (m, 16.00H, $H_{65,66,67}$, overlapped with water peak), 2.74 – 2.61 (m, 6H, $H_{9,32,45}$), 1.91 – 1.79 (m, 4H, $H_{13,28}$), 1.79 – 1.46 (m, 16H, $H_{7,8,33,44,34,43,16,25}$), 1.46 – 1.33 (m, 8H, $H_{14,27,15,26}$).
^{13}C NMR (126 MHz, Acetone-d_6, 298 K) δ = 175.62, 175.57 (C_{59}′), 175.44 (C_{58}), 174.32 (C_{57}), 174.11 – 173.96 (C_{59}), 167.34 (C_{56}), 159.31 (C_{50}), 148.28 – 148.22, 148.15, 148.11, 148.07, 148.03 ($C_{10,31,46}$), 143.55, 143.48, 143.45, 143.43 (C_{61}), 138.75 ($C_{3,20,21,38,39}$), 137.46 (C_2), 136.36 ($C_{20,21,38,39}$′), 132.99 (C_{52}), 130.59 (C_{54}), 129.55 – 129.18 (C_{62}), 128.23, 128.20, 128.04 (C_{63}), 127.27, 127.17, 127.04 (C_{64}), 123.29 (C_{53}), 122.90 (C_{47}), 122.04, 121.99, 121.97, 121.93 ($C_{11,30}$), 120.22, 120.19 (C_{55}), 116.16 (C_{51}), 92.23, 92.23, 92.19, 92.16 ($C_{19,22,37,40}$′), 92.14, 92.07 ($C_{4,19,22,37,40}$), 82.28 (C_1), 74.47 (C_{60}′), 74.11, 74.09 (C_{60}), 72.18, 72.16, 72.13, 71.93, 71.89, 71.88 ($C_{6,17,24,35,42}$), 69.50, 69.48, 69.44 ($C_{18,23,36,41}$′), 69.15, 69.09, 69.01 ($C_{5,18,23,36,41}$), 67.71 (C_{49}), 51.30 (C_{67}), 50.41 (C_{65}), 50.34, 50.31, 50.28 ($C_{12,29}$), 49.98 (C_{48}), 48.93 (C_{66}), 48.01, 47.95 (C_{67}′), 31.03, 30.98 ($C_{13,28}$), 30.12 – 29.64 ($C_{16,25,34}$, overlapped with solvent peak), 26.97, 26.92, 26.89, 26.87, 26.84, 26.80 ($C_{44,8,33,14,27,7,43}$), 26.24 ($C_{15,26}$), 26.01, 25.99, 25.98, 25.92 ($C_{45,9,32}$).
HRMS-ESI (-): 2067.9460 $[M-H]^-$, calculated for $C_{125}H_{127}N_{12}O_{17}^-$: 2067.9448

Compound **S18c**:

Synthesis followed the general experimental procedure. **S17c** (11 mg, 5.9 µmol, 1.0 eq.), **S7** (1.5 mg, 7.1 µmol, 1.2 eq.), $CuSO_4$ (9.4 mg, 59 µmol, 10.0 eq.) and sodium ascorbate (58 mg, 295 µmol, 50.0 eq.) were used in the reaction to yield **S18c** (5 mg, 2.4 µmol, 41% yield).

The product is a mixture of stereoisomers including *exo-exo-exo* and *exo-exo-endo* with respect to the furan-maleimide Diels-Alder moieties; 9% of the furan units remain unreacted. Other minor stereoisomers are present in minute amount and can't be individually quantified.

^{1}H NMR (500 MHz, Acetone-d_6, 298 K) δ = 7.81 (s, 0.82H, H_{47}), 7.80 (s, 0.13H, H_{47}′), 7.68 – 7.61 (m, 3H, $H_{11,30,53}$), 7.58 – 7.53 (m, 1H, H_{51}), 7.49 – 7.41 (m, 18H, H_{62}), 7.39 (t, *J* = 7.9 Hz, 1H, H_{54}), 7.28 – 7.18 (m, 18H, H_{63}), 7.18 – 7.10 (m, 10H, $H_{64,55}$), 6.59 – 6.56 (m, 0.85H, H_2), 6.56 – 6.52 (m, 4.49H, $H_{3,20,21,38,39}$), 5.49 (d, *J* = 5.7 Hz, 0.17H, H_2′), 5.40 (d, *J* = 5.7 Hz, 0.19H, H_3′), 5.38 (s, 0.31H, $H_{20,21,38,39}$′), 5.25 (d, *J* = 1.8

Hz, 0.84H, H_1), 4.99 (d, J = 5.5 Hz, 0.18H, $H_{1'}$), 4.81 – 4.75 (m, 2H, H_{48}), 4.49 (t, J = 5.1 Hz, 2H, H_{49}), 4.31 (t, J = 7.1 Hz, 4H, $H_{12,29}$), 4.19 – 4.11 (m, 4.61H, $H_{5,18,23,36,41}$), 3.94 – 3.76 (m, 5.59H, $H_{5,18,23,36,41}$ *and* $H_{5,18,23,36,41}'$), 3.71 – 3.58 (m, 5.18H, H_6 *and* $H_{65,67}'$), 3.58 – 3.36 (m, 7.32H, $H_{6,35,42,17,24}$ *and* H_{66}'), 2.97 – 2.73 (m, 21.92H, $H_{65,66,67}$, overlapped with water peak), 2.73 – 2.62 (m, 6H, $H_{9,32,45}$), 1.86 (p, J = 7.2 Hz, 4H, $H_{13,28}$), 1.78 – 1.48 (m, 16H, $H_{7,8,33,44,34,43,16,25}$), 1.47 – 1.37 (m, 4H, $H_{15,26}$), 1.37 – 1.32 (m, 4H, $H_{14,27}$).
^{13}C NMR (126 MHz, Acetone-d_6, 298 K) δ = 175.62 (C_{59}'), 175.43 (C_{58}), 174.31 (C_{57}), 174.04 (C_{59}), 167.39 (C_{56}), 159.30 (C_{50}), 148.24, 148.09, 148.07 ($C_{10,31,46}$), 143.54, 143.45 (C_{61}), 138.75 ($C_{3,20,21,38,39}$), 137.45 (C_2), 133.09 (C_{52}), 130.57 (C_{54}), 129.35 (C_{62}), 128.22, 128.19, 128.05, 128.03 (C_{63}), 127.26, 127.16, 127.05, 127.03 (C_{64}), 123.29 (C_{53}), 122.90 (C_{47}), 122.00 , 121.92 ($C_{11,30}$), 120.13 (C_{55}), 116.17 (C_{51}), 92.15, 92.13, 92.07 ($C_{4,19,22,37,40}$), 82.28 (C_1), 74.11, 74.08 (C_{60}), 72.18, 72.15, 72.12 ($C_{6,17,24,35,42}$), 69.15, 69.09, 69.01 ($C_{5,18,23,36,41}$), 67.71 (C_{49}), 51.31 (C_{67}), 50.41 (C_{65}), 50.33, 50.30 ($C_{12,29}$), 49.98 (C_{48}), 48.93 (C_{66}), 31.04 ($C_{13,28}$), 30.07 – 29.67 ($C_{16,25,34}$, overlapped with solvent peak), 26.95, 26.92, 26.89 ($C_{44,8,33,14,27,7,43}$), 26.23 ($C_{15,26}$), 26.06 – 25.92 ($C_{45,9,32}$).
HRMS-ESI (-): 2067.9421 [M-H]$^-$, calculated for $C_{125}H_{127}N_{12}O_{17}^-$: 2067.9448

4.1.25 Synthesis of S19

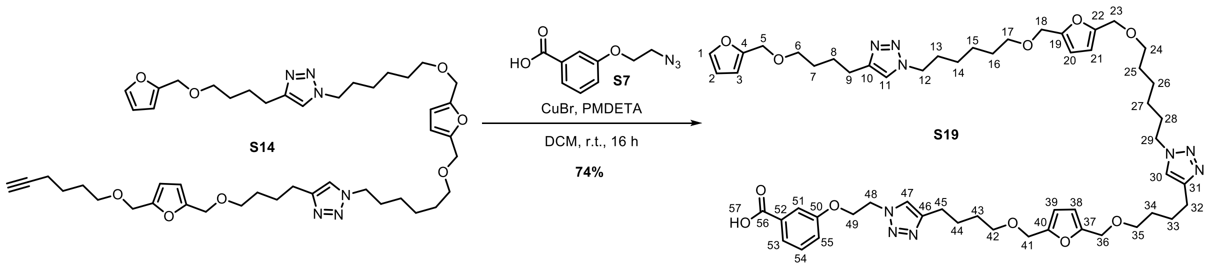

Three sealed 5 mL microwave vials (*A*, *B*, and *C*) were prepared as follows: *A* contained PMDETA (20 mg, 0.12 mmol, 1.5 eq.) in DCM (3 mL), *B* contained CuBr (18 mg, 0.12 mmol, 1.6 eq.), and *C* contained **S14** (59 mg, 0.070 mmol, 0.9 eq.) and **S7** (16 mg, 0.077 mmol, 1.0 eq.). Vials *B* and *C* were subjected to three N_2/vacuum cycles before use. The solution in vial *A* was degassed by bubbling with N_2 until ~1 mL DCM left before being transferred to vial *B via* cannula; the resulting mixture was stirred until all CuBr had dissolved. The CuBr/PMDETA solution in vial *B* was then transferred to vial *C via* cannula; the resulting reaction mixture was stirred for 16 h at room temperature. The reaction mixture was washed by aqueous EDTA solution (0.25 M, pH 7, 2 x 1 mL) and brine (2 x 1 mL). The organic layer was collected and dried with magnesium sulfate. The mixture was filtered before being concentrated under vacuum. The residue was purified by preparative TLC (2000 µm, DCM/MeOH, 20/1, eluted twice) to yield **S19** as white powder (60 mg, 0.057 mmol, 74% yield).

^{1}H NMR (500 MHz, Acetone-d_6, 298 K) δ = 7.81 (s, 1H, H_{47}), 7.69 – 7.61 (m, 3H, $H_{11,30,53}$), 7.56 (dd, J = 2.7, 1.5 Hz, 1H, H_{51}), 7.50 (dd, J = 1.8, 0.9 Hz, 1H, H_1), 7.40 (t, J = 8.0 Hz, 1H, H_{54}), 7.18 (dd, J = 8.2, 2.8 Hz, 1H, H_{55}), 6.37 (dd, J = 3.2, 1.8 Hz, 1H, H_2), 6.35 (d, J = 3.2 Hz, 1H, H_3), 6.29 (d, J = 4.1 Hz, 4H, $H_{20,21,38,39}$), 4.80 (t, J = 5.1 Hz, 2H, H_{48}), 4.51 (t, J = 5.1 Hz, 2H, H_{49}), 4.39 (s, 2H, H_5), 4.38 – 4.35 (m, 8H, $H_{18,23,36,41}$), 4.32 (t, J = 7.1 Hz, 4H, $H_{12,29}$), 3.48 – 3.43 (m, 6H, $H_{6,35,42}$), 3.43 – 3.39 (m, 4H, $H_{17,24}$), 2.66 (td, J = 7.5, 1.8 H, 6H, $H_{9,32,45}$), 1.85 (p, J = 7.2 Hz, 4H, $H_{13,28}$), 1.74 – 1.63 (m, 6H, $H_{8,33,44}$), 1.63 – 1.54 (m, 6H, $H_{7,34,43}$), 1.54 – 1.47 (m, 4H, $H_{16,25}$), 1.41 – 1.33 (m, 5H, $H_{15,26}$), 1.33 – 1.24 (m, 10H, $H_{14,27}$).
^{13}C NMR (126 MHz, Acetone-d_6, 298 K) δ = 167.45 (C_{56}), 159.29 (C_{50}), 153.44, 153.39, 153.37 ($C_{4,19,22,37,40}$), 148.20, 148.05, 148.04 ($C_{10,31,46}$), 143.48 (C_1), 133.14 (C_{52}), 130.52 (C_{54}), 123.30 (C_{53}), 122.83 (C_{47}), 121.90, 121.84 ($C_{11,30}$), 120.14 (C_{55}), 116.16 (C_{51}), 111.05 (C_2), 110.37 ($C_{20,21,38,39}$), 109.67 (C_3), 70.37 ($C_{17,24}$), 70.25 ($C_{6,35,42}$), 67.69 (C_{49}), 65.19 ($C_{18,23,36,41}$), 65.06 (C_5), 50.32, 50.29 ($C_{12,29}$), 50.00 (C_{48}), 30.98 ($C_{13,28}$), 29.92 – 29.80 ($C_{16,25,34}$, overlapped with solvent peak), 26.94, 26.91, 26.89 ($C_{8,33,44,14,27,7,43}$), 26.27 ($C_{15,26}$), 25.98, 25.94, 25.93 ($C_{9,32,45}$).
HRMS-ESI (-): 1050.5648 [M-H]$^-$, calculated for $C_{56}H_{76}N_9O_{11}^-$: 1050.5670.

4.1.26 Synthesis of S18d

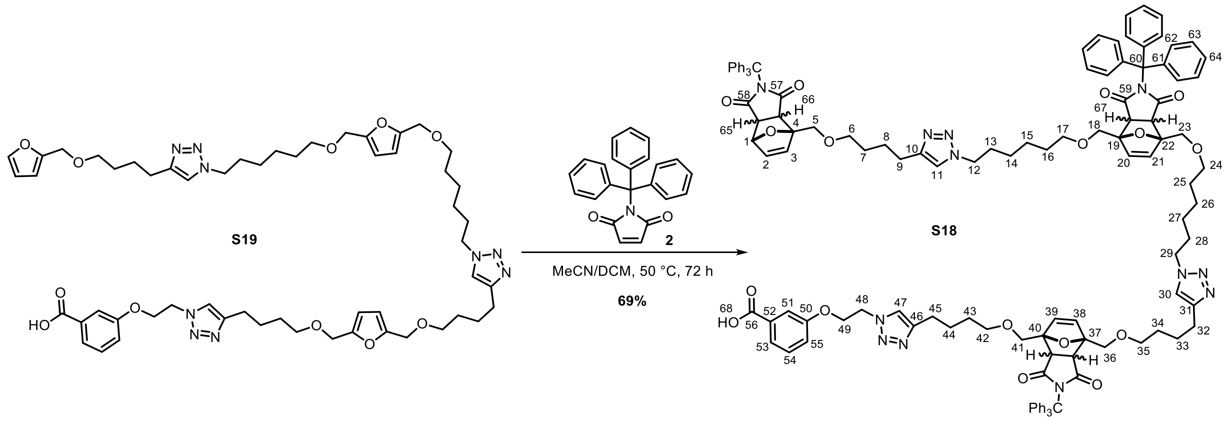

A solution of **S19** (25 mg, 0.024 mmol, 1.0 eq.) and **2** (1600 mg, 4.8 mmol, 200.0 eq.) in a mixture of acetonitrile (6 mL) and DCM (6 mL) was stirred at 50 °C for 72 h. The solvent was removed under vacuum and the residue purified by flash column chromatography (SiO_2, DCM/MeOH, 20/1, eluted twice) to yield **S18d** (34 mg, 0.016 mmol, 69% yield) as a faint yellow powder.

S18d was isolated as a mixture of stereoisomers including *endo-endo-exo*, *endo-endo-endo*, *exo-endo-exo*, *endo-exo-exo*, *exo-endo-endo*, *endo-exo-endo*, *exo-exo-exo*, and *exo-exo-endo* with respect to the furan-maleimide Diels-Alder moieties; 6% of the furan units remain unreacted.

When distinguishable, *exo* and *endo* adducts are denoted as X_x and X_x' respectively. Unreacted furan units are denoted as X_x''.

^{1}H NMR (500 MHz, Acetone-d$_6$, 298 K) δ = 7.80 (s, 0.43H, H_{47}), 7.79 (s, 0.49H, H_{47}'), 7.69 – 7.60 (m, 3H, $H_{11,30,53}$), 7.57 (s, 1H, H_{51}), 7.51 – 7.34 (m, 19H, $H_{62,54}$), 7.28 – 7.17 (m, 18H, H_{63}), 7.17 – 7.08 (m, 10H, $H_{64,55}$), 6.56 (d, J = 5.1 Hz, 0.76H, H_2), 6.55 – 6.51 (m, 2.50H, $H_{3,20,21,38,39}$), 6.38 – 6.36 (m, 0.07H, H_2''), 6.36 – 6.33 (m, 0.08H, H_3''), 6.30 – 6.26 (m, 0.22H, $H_{20,21,38,39}''$), 5.50 (dd, J = 5.8, 1.8 Hz, 0.17H, H_2'), 5.42 (dd, J = 5.7, 2.0 Hz, 0.19H, H_3'), 5.40 – 5.35 (m, 2.06H, $H_{20,21,38,39}'$), 5.29 – 5.20 (m, 0.77H, H_1), 5.02 – 4.96 (m, 0.18H, H_1'), 4.84 – 4.70 (m, 2H, H_{48}), 4.54 – 4.43 (m, 2H, H_{49}), 4.40 – 4.35 (m, 0.63H, $H_{5,18,23,36,41}''$), 4.34 – 4.24 (m, 4H, $H_{12,29}$), 4.19 – 4.09 (m, 2.60H, $H_{5,18,23,36,41}$), 3.95 – 3.74 (m, 7.11H, $H_{5,18,23,36,41}$ *and* $H_{5,18,23,36,41}'$), 3.73 – 3.38 (m, 13.07H, $H_{6,35,42,17,24}$ *and* $H_{65,66,67}'$), 2.88 – 2.81 (m, 4.41H, $H_{65,67}$, overlapped with water peak), 2.81 – 2.75 (m, 1.61H, H_{66}, overlapped with water peak), 2.74 – 2.62 (m, 6H, $H_{9,32,45}$), 1.91 – 1.79 (m, 4H, $H_{13,28}$), 1.78 – 1.45 (m, 16H, $H_{7,8,33,44,34,43,16,25}$), 1.45 – 1.30 (m, 8H, $H_{14,27,15,26}$).

^{13}C NMR (126 MHz, Acetone-d$_6$, 298 K) δ = 175.61, 175.56 (C_{59}'), 175.45 (C_{58}), 174.32 (C_{57}), 174.04 (C_{59}), 167.43 (C_{56}), 159.31 (C_{50}), 153.65 – 153.22 ($C_{4,19,22,37,40}''$), 148.25, 148.17, 148.14 – 147.97 ($C_{10,31,46}$), 143.55, 143.46, 143.43 (C_{61}), 138.76 ($C_{3,20,21,38,39}$), 137.45 (C_2), 136.38 ($C_{20,21,38,39}'$), 135.88, 135.80 ($C_{2,3}'$), 133.08 (C_{52}), 130.57 (C_{54}), 129.50 – 129.24 (C_{62}), 128.23, 128.20, 128.05 (C_{63}), 127.27, 127.17, 127.05 (C_{64}), 123.32 (C_{53}), 122.90 (C_{47}), 122.03, 121.98, 121.94 ($C_{11,30}$), 120.22 (C_{55}), 116.19 (C_{51}), 111.06 (C_2''), 110.39 ($C_{20,21,38,39}''$), 109.69 (C_3''), 92.33, 92.26, 92.21, 92.16 ($C_{19,22,37,40}'$), 92.15, 92.08 ($C_{4,19,22,37,40}$), 82.29 (C_1), 80.11 (C_1'), 74.49 (C_{60}'), 74.14, 74.12 (C_{60}), 72.20, 72.18, 72.14, 71.96, 71.92, 71.91 ($C_{6,17,24,35,42}$), 70.49 – 70.15 ($C_{6,17,24,35,42}''$), 69.64 – 69.39 ($C_{18,23,36,41}'$), 69.26 – 68.86 ($C_{5,18,23,36,41}$), 67.72 (C_{49}), 65.20, 65.07 ($C_{5,18,23,36,41}''$), 51.31 (C_{67}), 50.42 (C_{65}), 50.35, 50.33, 50.30 ($C_{12,29}$), 50.00 (C_{48}), 48.93 (C_{66}), 48.38 (C_{65}'), 48.03, 47.97 (C_{67}'), 46.27 (C_{66}'), 31.02, 30.98 ($C_{13,28}$), 30.10 – 29.76 ($C_{16,25,34}$, overlapped with solvent peak), 26.96, 26.93, 26.88, 26.85, 26.80 ($C_{44,8,33,14,27,7,43}$), 26.29, 26.24, ($C_{15,26}$), 26.02, 25.98, 25.93 ($C_{45,9,32}$).

HRMS-ESI (+): 1035.4817 $[M+2H]^{2+}$, calculated for $C_{125}H_{128}N_{12}O_{17}H_2^{2+}$: 1035.4833.

4.1.27 Synthetic Route to 5-Cargo Diels-Alder Cargo Compartments

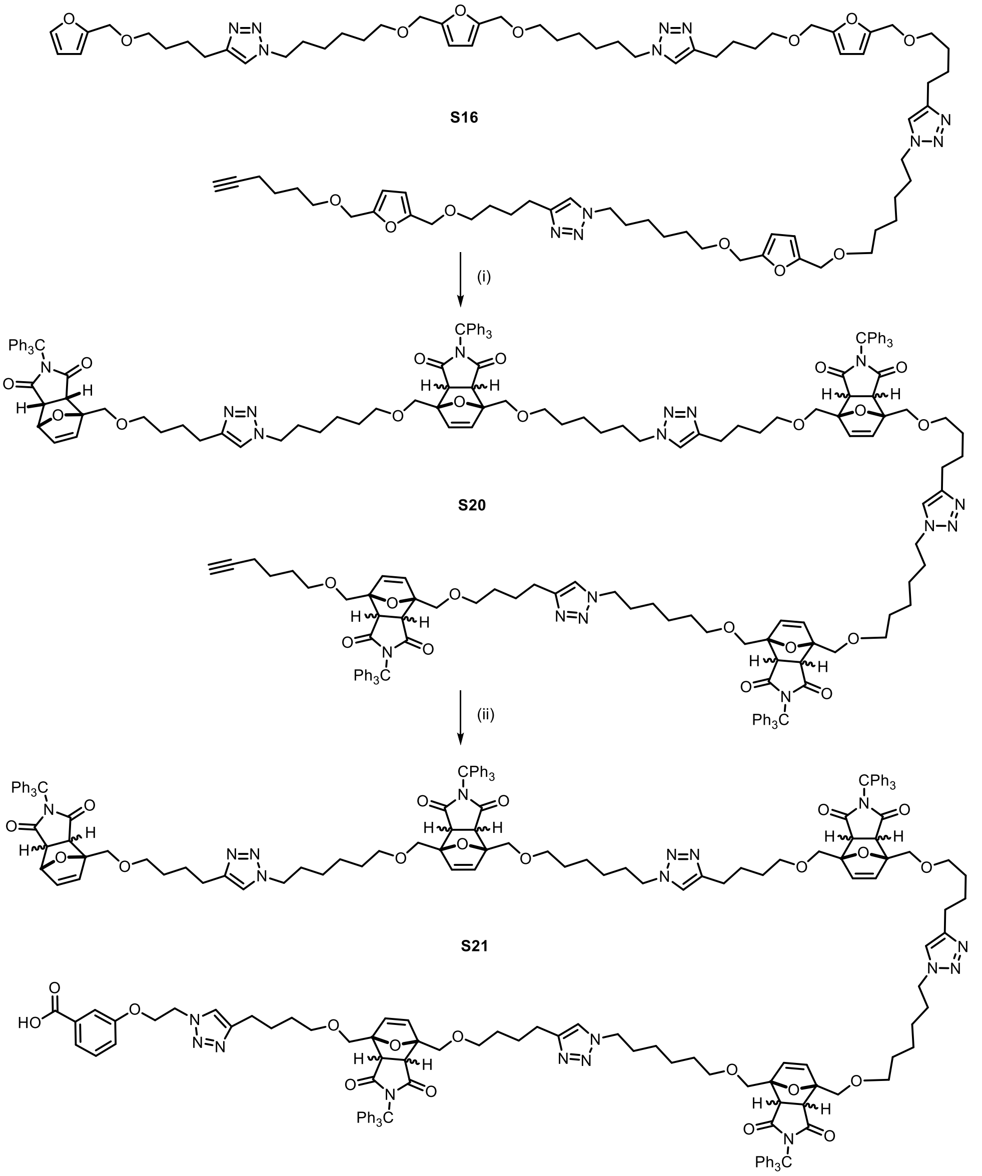

Figure S7. (i) **2**, MeCN/DCM, 50 °C, 72 h, 89% yield; (ii) **S7**, $CuSO_4$, sodium ascorbate, H_2O/THF, r.t., 16 h, 89% yield.

4.1.28 Synthesis of S20

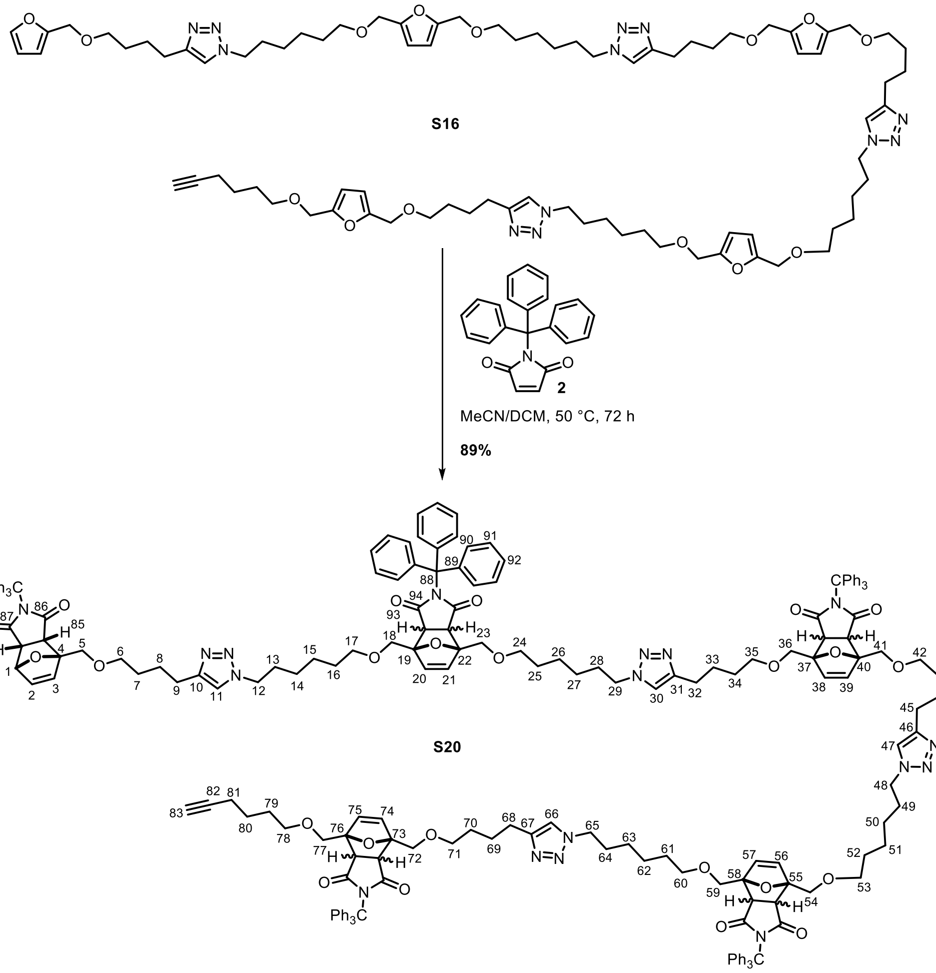

A solution of **S16** (100 mg, 0.066 mmol, 1.0 eq.) and **2** (5600 mg, 16 mmol, 250.0 eq.) in acetonitrile (15 mL) and DCM (15 mL) was stirred at 50 °C for 72 h. The solvent was removed and the residue purified by flash column chromatography (SiO_2, DCM/MeOH, 30/1) to yield **S20** (188 mg, 0.059 mmol, 89% yield) as faint yellow powder.

S20 was isolated as a mixture of stereoisomers; 5% of the furan units remain unreacted.

When distinguishable, *exo* and *endo* adducts are denoted as X_x and X_x' respectively. Unreacted furan units are denoted as X_x''.

^{1}H NMR (500 MHz, Acetone-*d*$_6$, 298 K) δ = 7.69 – 7.62 (m, 4H, $H_{11,30,47,66}$), 7.55 – 7.39 (m, 30H, H_{90}), 7.29 – 7.17 (m, 30H, H_{91}), 7.17 – 7.05 (m, 15H, H_{92}), 6.61 – 6.48 (m, 5.20H, $H_{2,3,20,21,38,39,56,57,74,75}$), 6.39 – 6.34 (m, 0.16H $H_{2,3}''$), 6.34 – 6.28 (m, 0.31H, $H_{20,21,38,39,56,57,74,75}''$), 5.47 (d, *J* = 4.5 Hz, 0.19H, H_2'), 5.42 – 5.32 (m, 4.25H, $H_{3,20,21,38,39,56,57,74,75}'$), 5.25 (s, 0.82H, H_1), 5.00 (d, *J* = 4.2 Hz, 0.21H, H_1'), 4.41 – 4.34 (m, 0.98H, $H_{5,18,23,36,41,54,59,72,77}''$), 4.34 – 4.25 (m, 8H, $H_{12,29,48,65}$), 4.21 – 4.09 (m, 4.56H, $H_{5,18,23,36,41,72,77}$), 3.97 – 3.75 (m, 13.37H, $H_{5,18,23,36,41,54,59,72,77}$ and $H_{5,18,23,36,41,54,59,72,77}'$), 3.75 – 3.59 (m, 8.52H, H_6 *and* $H_{84,93}'$), 3.59 – 3.35 (m, 15.81H, $H_{6,17,24,35,42,53,60,71,78}$ and H_{85}'), 2.90 – 2.73 (m, 5.19H, $H_{84,85,93}$), 2.73 – 2.58 (m, 8H, $H_{9,32,45,68}$), 2.44 – 2.34 (m, 1H, H_{83}), 2.24 – 2.15 (m, 2H, H_{81}), 1.92 – 1.79 (m, 8H, $H_{13,28,49,64}$), 1.79 – 1.38 (m, 28H, $H_{8,33,44,69,80,7,34,43,70,79,16,25,52,61}$), 1.37 – 1.21 (m, 16H, $H_{14,27,15,26,51,62,50,63}$, overlapped with grease peak).

^{13}C NMR (126 MHz, Acetone-*d*$_6$, 298 K) δ = 175.58, 175.54, 175.50 (C_{94}'), 175.42 (C_{87}), 174.30 (C_{86}), 174.11 – 173.92 (C_{94}), 153.37 ($C_{19,22,37,40,55,58,73,76}''$), 148.08, 148.06, 148.03, 148.01 ($C_{10,31,46,67}$), 143.52, 143.48 – 143.36 (C_{89}), 138.86 – 138.61 ($C_{3,20,21,38,39,56,57,74,75}$), 137.45 ($C_2$), 136.47 – 136.27

($C_{20,21,38,39,56,57,74,75}'$), 135.86, 135.79 ($C_{2,3}'$), 129.57, 129.50 – 129.16 (C_{90}), 128.22, 128.19, 128.04 (C_{91}), 127.27, 127.16, 127.04 (C_{92}), 122.06 – 121.69 ($C_{11,30,47,66}$), 110.38 ($C_{20,21,38,39,56,57,74,75}''$), 92.32, 92.26, 92.23, 92.20 ($C_{19,22,37,40,55,58,73,76}'$), 92.14, 92.10, 92.06 ($C_{4,19,22,37,40,55,58,73,76}$), 84.95 ($C_{82}$), 84.88 ($C_{82}'$), 82.27 ($C_{1}$), 80.09 ($C_{1}'$), 74.48 ($C_{88}'$), 74.22 – 74.04 ($C_{88}$), 72.26 – 72.15, 72.13, 71.96, 71.92, 71.82, 71.59 ($C_{6,17,24,35,42,53,60,71,78}$), 70.05 ($C_{83}$), 69.57, 69.53, 69.46 ($C_{18,23,36,41,54,59,72,77}'$), 69.15, 69.08, 69.00 ($C_{5,18,23,36,41,54,59,72,77}$), 65.20 ($C_{5,\ 18,23,36,41,54,59,72,77}''$), 51.29 ($C_{93}$), 50.39 ($C_{84}$), 50.35 – 50.13 ($C_{12,29,48,65}$), 48.91 ($C_{85}$), 48.36 ($C_{84}'$), 48.04, 48.01, 47.96 ($C_{93}'$), 46.26 ($C_{85}'$), 31.03, 30.98 ($C_{13,28,49,64}$), 30.08 – 29.48 ($C_{16,25,52,61,34,43,70}$, overlapped with solvent peak), 26.92, 26.85 ($C_{7,79,14,27,50,63,8,33,44,69}$), 26.24 ($C_{15,26,51,62}$), 26.05, 25.99 ($C_{9,32,45,68,80}$), 18.55 ($C_{81}$), 18.49 ($C_{81}'$).

HRMS-ESI (+): 1604.2889 $[M+2H]^{2+}$, calculated for $C_{198}H_{207}N_{17}O_{24}H_2^{2+}$: 1604.2823.

4.1.29 Synthesis of S21

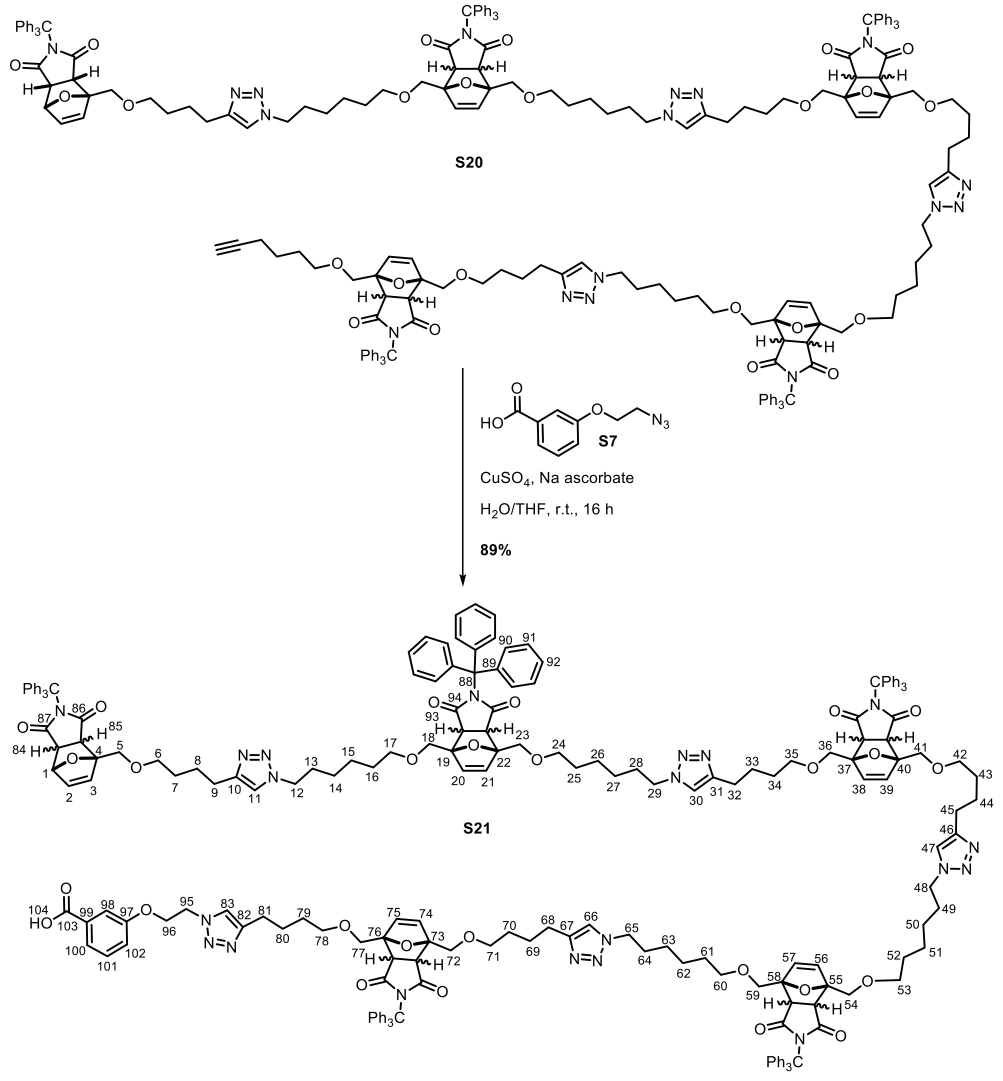

A solution of $CuSO_4$ (18 mg, 115 µmol, 5.0 eq.), **S7** (5.7 mg, 28 µmol, 1.2 eq.) and **S20** (74 mg, 23 µmol, 1.0 eq.) in a mixture of THF (3 mL) and water (1 mL) was degassed by bubbling N_2 through it for 3 min; sodium ascorbate (114 mg, 577 µmol, 25.0 eq.) was then added. The mixture was stirred for 16 h at room temperature before aqueous HCl solution (0.1 N) was added to adjust the pH of the mixture to ~3. DCM (20 mL) was added, and the mixture washed by aqueous EDTA solution (0.25 M, pH 7, 2 x 20 mL) and brine (2 x 20 mL). The organic solvent was separated and dried with magnesium sulfate. The mixture was filtered before being concentrated under vacuum. The residue was purified by preparative TLC (2000 µm, DCM/MeOH, 20/1, eluted twice) to give **S21** (70 mg, 21 µmol, 89% yield) as a white powder.

S21 was isolated as a mixture of stereoisomers; 1% of the furan units remained unreacted.

When distinguishable, *exo* and *endo* adducts are denoted as X_x and X_x′ respectively. Unreacted furan units are denoted as X_x′′.

^{1}H NMR (500 MHz, Acetone-d_6, 298 K) δ = 7.79 (s, 0.38H, H_{83}), 7.78 (s, 0.48H, H_{83}′), 7.69 – 7.59 (m, 5H, $H_{11,30,47,66,100}$), 7.57 (s, 1H, H_{98}), 7.54 – 7.34 (m, 31H, $H_{90,101}$), 7.33 – 7.17 (m, 30H, H_{91}), 7.17 – 7.07 (m, 16H, $H_{92,102}$), 6.61 – 6.49 (m, 4.85H, $H_{2,3,20,21,38,39,56,57,74,75}$), 6.29 (s, 0.10H, $H_{20,21,38,39,56,57,74,75}$′′), 5.51 (d, *J* = 5.8 Hz, 0.15H, H_2′), 5.46 – 5.35 (m, 4.59H, $H_{3,20,21,38,39,56,57,74,75}$′), 5.25 (s, 0.76H, H_1), 4.99 (d, *J* = 5.5 Hz, 0.16H, H_1′), 4.80 – 4.73 (m, 2H, H_{95}), 4.47 (t, *J* = 4.4 Hz, 2H, H_{96}), 4.38 – 4.24 (m, 9.91H, $H_{5,18,23,36,41,54,59,72,77}$′′ and $H_{12,29,48,65}$), 4.21 – 4.10 (m, 3.98H, $H_{5,18,23,36,41,72,77}$), 3.99 – 3.76 (m, 13.66H, $H_{5,18,23,36,41,54,59,72,77}$ and $H_{5,18,23,36,41,54,59,72,77}$′), 3.74 – 3.59 (m, 10.27H, H_6 *and* $H_{84,93}$′), 3.59 – 3.39 (m, 15.86H, $H_{6,17,24,35,42,53,60,71,78}$ and H_{85}′), 2.90 – 2.76 (m, 4.83H, $H_{84,85,93}$), 2.76 – 2.58 (m, 10.55H, $H_{9,32,45,68,81}$), 1.91 – 1.79 (m, 8H, $H_{13,28,49,64}$), 1.78 – 1.38 (m, 28H, $H_{8,33,44,69,80,7,34,43,70,79,16,25,52,61}$), 1.37 – 1.23 (m, 16H, $H_{14,27,15,26,51,62,50,63}$, overlapped with grease peak).
^{13}C NMR (126 MHz, Acetone-d_6, 298 K) δ = 175.62, 175.57 (C_{94}′), 175.45 (C_{84}), 174.33 (C_{85}), 174.05 (C_{94}), 159.42, 159.27 (C_{97}), 148.25, 148.17, 148.11, 148.09, 148.07, 148.04 ($C_{10,31,46,67}$), 143.55, 143.46, 143.43 (C_{89}), 138.76 ($C_{3,20,21,38,39,56,57,74,75}$), 137.47 ($C_2$), 136.40 ($C_{20,21,38,39,56,57,74,75}$′), 130.46 ($C_{101}$), 129.55 – 129.24 ($C_{90}$), 128.24, 128.21, 128.06 (C_{91}), 127.29, 127.18, 127.06 (C_{92}), 123.34, 123.24 (C_{100}), 122.90 (C_{83}), 122.09 – 121.82 ($C_{11,30,47,66}$), 119.99, 119.93, 119.87 (C_{102}), 116.20, 115.96 (C_{98}), 92.28, 92.22 ($C_{19,22,37,40,55,58,73,76}$′), 92.17, 92.09 ($C_{4,19,22,37,40,55,58,73,76}$), 82.30 ($C_1$), 80.12 ($C_1$′), 74.50 ($C_{88}$′), 74.15, 74.13 ($C_{88}$), 72.21, 72.15, 71.98, 71.94, 71.91 ($C_{6,17,24,35,42,53,60,71,78}$), 69.54, 69.48 ($C_{18,23,36,41,54,59,72,77}$′), 69.17, 69.10, 69.02 ($C_{5,18,23,36,41,54,59,72,77}$), 67.70 ($C_{96}$), 51.32 ($C_{93}$), 50.42 ($C_{84}$), 50.38 – 50.21 ($C_{12,29,48,65}$), 50.01 ($C_{95}$), 48.94 ($C_{85}$), 48.39 ($C_{84}$′), 48.11 – 48.02, 47.99 (C_{93}′), 46.28 (C_{85}′), 31.04, 30.99 ($C_{13,28,49,64}$), 30.13 – 29.60 ($C_{16,25,52,61,34,43,70}$, overlapped with solvent peak), 26.94, 26.87, 26.80 ($C_{7,79,14,27,50,63,8,33,44,69,80}$), 26.26 ($C_{15,26,51,62}$), 26.05, 25.99, 25.96, 25.94 ($C_{9,32,45,68,81}$).
HRMS-ESI (+): 1138.8776 $[M+3H]^{3+}$, calculated for $C_{207}H_{216}N_{20}O_{27}H_3^{3+}$: 1138.8787

4.1.30 Synthesis of Reference Five-Cargo Unloaded Cargo Compartment, S22

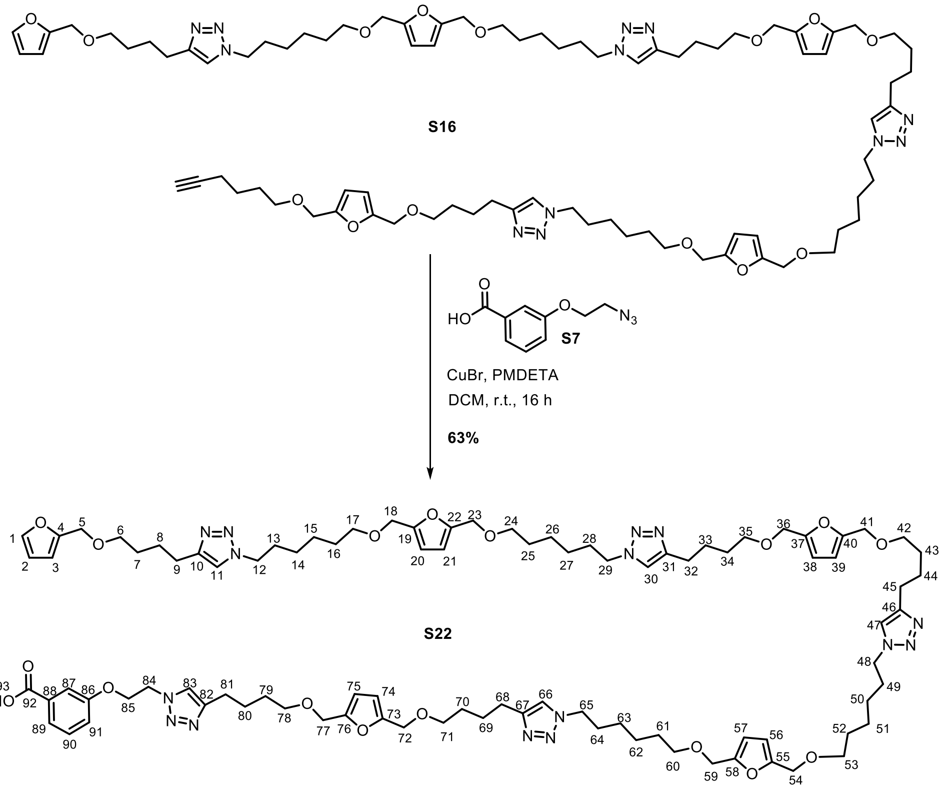

Three sealed 5 mL microwave vials (*A*, *B*, and *C*) were prepared as follows: *A* contained PMDETA (6.3 mg, 36 µmol, 1.5 eq.) in DCM (3 mL), *B* contained CuBr (5.6 mg, 39 µmol, 1.6 eq.), and *C* contained **S16** (37 mg, 24 µmol, 1.0 eq.) and **S7** (5.0 mg, 24 µmol, 1.0 eq.). Vials *B* and *C* were subjected to three N_2/vacuum cycles before use. The solution in vial *A* was degassed by bubbling with N_2 until ~1 mL DCM left before being transferred to vial *B via* cannula; the resulting mixture was stirred until all CuBr had dissolved. The CuBr/PMDETA solution in vial *B* was then transferred to vial *C via* cannula; the resulting reaction mixture was stirred for 16 h at room temperature. The reaction mixture was washed by aqueous EDTA solution (0.25 M, pH 7, 2 x 1 mL) and brine (2 x 1 mL). The organic layer was collected and dried with magnesium sulfate. The mixture was filtered before being concentrated under vacuum. The residue was purified by preparative TLC (2000 µm, DCM/MeOH, 20/1, eluted twice) to yield **S22** as white powder (26 mg, 15 µmol, 63% yield).

^{1}H NMR (500 MHz, Methanol-*d*$_4$, 298 K) δ = 7.81 (s, 1H, H_{83}), 7.73 – 7.65 (m, 4H, $H_{11,30,47,66}$), 7.61 (d, *J* = 7.6 Hz, 1H, H_{89}), 7.54 – 7.49 (m, 1H, H_{87}), 7.48 – 7.43 (m, 1H, H_1), 7.34 (t, *J* = 7.9 Hz, 1H, H_{90}), 7.11 (dd, *J* = 8.3, 2.7 Hz, 1H, H_{91}), 6.40 – 6.32 (m, 2H, $H_{2,3}$), 6.32 – 6.22 (m, 8H, $H_{20,21,38,39,56,57,74,75}$), 4.77 (t, *J* = 5.0 Hz, 2H, H_{84}, overlapped with H_2O signal), 4.46 – 4.27 (m, 28H, $H_{85,5,18,23,36,41,54,59,72,77,12,29,48,65}$), 3.51 – 3.39 (m, 18H, $H_{6,35,42,71,78,17,24,53,60}$), 2.73 – 2.61 (m, 10H, $H_{9,32,45,68,81}$), 1.93 – 1.78 (m, 8H, $H_{13,28,49,64}$), 1.74 – 1.64 (m, 10H, $H_{8,33,44,69,80}$), 1.63 – 1.46 (m, 18H, $H_{7,34,43,70,79,16,25,52,61}$), 1.42 – 1.20 (m, 16H, $H_{15,26,51,62,14,27,50,63}$, overlapped with grease peak).

^{13}C NMR (126 MHz, Methanol-*d*$_4$, 298 K) δ = 159.65 (C_{86}), 153.64 ($C_{19,22,37,40,55,58,73,76}$), 153.40 ($C_4$), 149.04 ($C_{82}$), 148.95 ($C_{10,31,46,67}$), 143.99 ($C_1$), 130.72 ($C_{90}$), 124.08 ($C_{83}$), 123.79 ($C_{89}$), 123.16 ($C_{11,30,47,66}$), 120.51 ($C_{91}$), 116.37 ($C_{87}$), 111.30 ($C_3$), 111.01 ($C_{20,21,38,39,56,57,74,75}$), 110.27 ($C_2$), 70.91 ($C_{17,24,53,60}$), 70.77, 70.72, 70.68 ($C_{6,35,42,71,78}$), 67.86 ($C_{85}$), 65.60, 65.56 ($C_{18,23,36,41,54,59,72,77}$), 65.49 ($C_5$), 51.15 ($C_{12,29,48,65}$), 50.86 ($C_{84}$), 31.19 ($C_{13,28,49,64}$), 30.37 ($C_{16,25,52,61}$), 30.13 – 29.88 (m, $C_{7,34,43,70,79}$), 27.18, 27.16, 27.09 ($C_{14,27,50,63}$), 26.54 ($C_{15,26,51,62}$), 25.98, 25.91 ($C_{9,32,45,68,81}$).

HRMS-ESI (-): 1716.9736 [M-H]$^-$, calculated for $C_{92}H_{130}N_{15}O_{17}^-$: 1716.9775.

4.2 Synthesis of Rotaxane Precursors

4.2.30 Synthetic Route to *cis*- and *trans*-Rotaxane Precursors

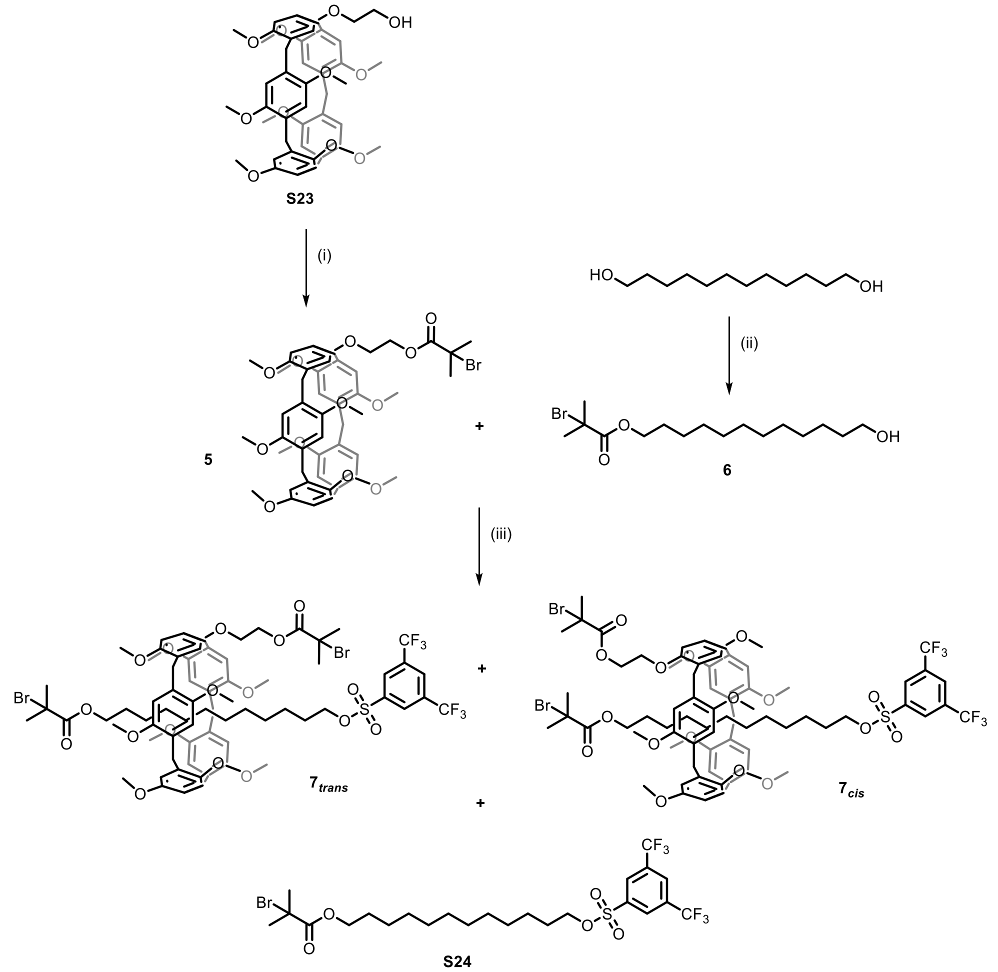

Figure S8. Synthetic route to **S24**. Conditions: (i) BiBB, Et_3N, DCM, r.t., 2 h, 86% yield; (ii) BiBB, Et_3N, DCM, r.t., 16 h, 30% yield; (iii) BTBSCl, Et_3N, $CHCl_3$, -15 °C, 2 h, 11%, 13%, and 69% yields for $\mathbf{7}_{trans}$, $\mathbf{7}_{cis}$, and **S24** respectively.

4.2.31 Synthesis of 5

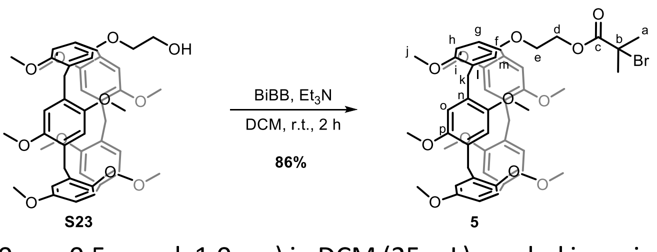

To a solution of **S23** (380 mg, 0.5 mmol, 1.0 eq.) in DCM (25 mL), cooled in an ice bath, was added BiBB (333 mg, 1.5 mmol, 3.0 eq.) and Et_3N (148 mg, 1.5 mmol, 3.0 eq.). The mixture was stirred for 2 h at room temperature before being concentrated under vacuum. The residue was purified by flash column chromatography (SiO_2, PE/EtOAc, 4/1) to yield **5** as a slightly orange powder (390 mg, 0.4 mmol, 86% yield).

^{1}H NMR (400 MHz, $CDCl_3$, 298 K) δ = 6.80 – 6.72 (m, 10H, $H_{h,m,o}$), 4.48 (t, J = 4.8 Hz, 2H, H_d), 4.05 (t, J = 4.9 Hz, 2H, H_e), 3.80 – 3.74 (m, 10H, H_k), 3.68 – 3.61 (m, 27H, H_j), 1.96 (s, 6H, H_a).

^{13}C NMR (101 MHz, $CDCl_3$, 298 K) δ = 171.88 (C_c), 151.53, 151.00, 150.97, 150.95, 150.90 ($C_{i,p}$), 149.58 (C_f), 129.17, 129.12, 128.57, 128.47, 128.42, 128.36, 128.32, 128.25, 128.21, 128.12 ($C_{g,l,n}$), 116.05, 114.34, 114.32, 114.29, 114.25, 114.20, 114.12 ($C_{h,m,o}$), 66.91 (C_e), 64.73 (C_d), 56.08, 56.02, 55.98, 55.95, 55.90, 55.84, 55.80 (C_j), 55.66 (C_b), 30.89 (C_a), 29.94, 29.87, 29.73, 29.70 (C_k).
HRMS-ESI (+): 953.2941 $[M+Na]^+$, calculated for $C_{50}H_{57}BrO_{12}Na^+$: 953.2926.

4.2.32 Synthesis of 6

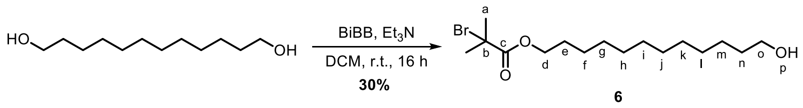

To a solution of dodecane-1,12-diol (4.0 g, 19.8 mmol, 1.0 eq.) in THF (60 mL), cooled in an ice bath, was added BiBB (2.3 g, 9.9 mmol, 0.5 eq.) and Et_3N (1.2 g, 11.9 mmol, 0.6 eq.). The mixture was stirred for 16 h at room temperature. The solution was concentrated under vacuum. The residue was purified by flash column chromatography (SiO_2, PE/EtOAc, 5/1) to yield **6** as colorless oil (2.1 g, 6.0 mmol, 30% yield).

^{1}H NMR (500 MHz, $CDCl_3$, 298 K) δ = 4.16 (t, J = 6.5 Hz, 2H, H_d), 3.64 (t, J = 6.6 Hz, 2H, H_o), 1.93 (s, 6H, H_a), 1.73 – 1.63 (m, 2H, H_e), 1.61 – 1.52 (m, 2H, H_n), 1.44 – 1.23 (m, 16H, $H_{f\text{-}m}$).
^{13}C NMR (126 MHz, $CDCl_3$, 298 K) δ = 171.91 (C_c), 66.31 (C_d), 63.23 (C_o), 56.16 (C_b), 32.94 (C_n), 30.94 (C_a), 29.72, 29.66, 29.64, 29.59, 29.56, 29.29 ($C_{g\text{-}l}$), 28.48 (C_e), 25.92, 25.87 ($C_{f,m}$).
HRMS-ESI (+): 373.1340 $[M+Na]^+$, calculated for $C_{16}H_{31}BrO_3Na^+$: 373.1349.

4.2.33 Synthesis of 7 and S24

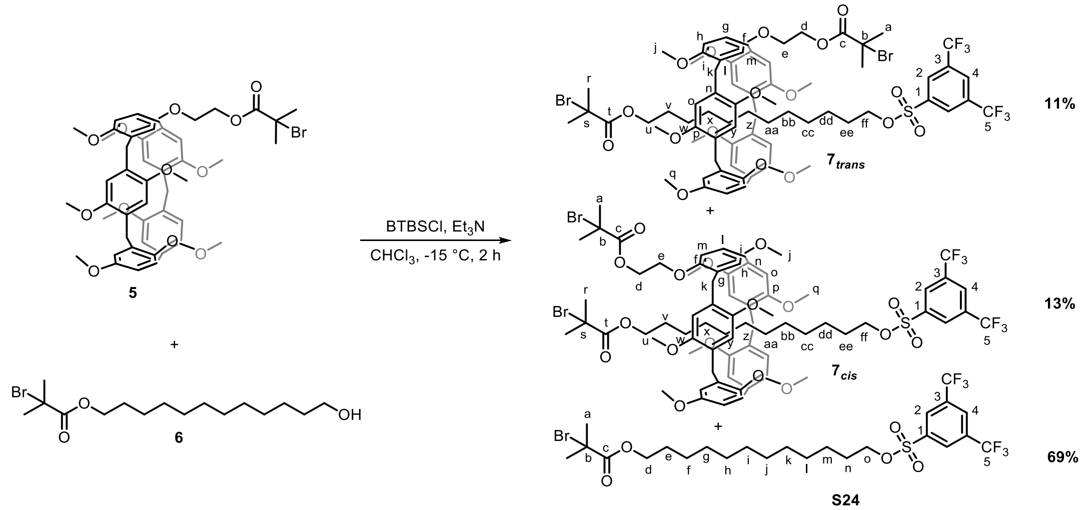

A solution of **5** (258 mg, 0.3 mmol, 1.0 eq.) and **6** (650 mg, 1.9 mmol, 6.7 eq.) in dry $CHCl_3$ (0.8 mL) was stirred at room temperature for 5 h. The reaction mixture was cooled in an ice bath and a solution of BTBSCl (695 mg, 2.2 mmol, 8.0 eq.) and Et_3N (244 mg, 2.4 mmol, 8.7 eq.) in dry $CHCl_3$ (0.5 mL) was added to the reaction mixture dropwise; the mixture was then stirred for a further 2 h. The reaction mixture was neutralized by addition of aqueous 0.2 M HCl solution before being extracted with DCM (3 x 10 mL). The organic phases were combined and dried with magnesium sulfate. The solvent was removed under vacuum and the resulting residue purified by flash column chromatography (SiO_2, PE/Et_2O, 6/1 to 1/2) to yield **7*trans*** (65 mg, 0.04 mmol, 15% yield based on **5**) and **7*cis*** (75 mg, 0.05 mmol, 17% yield based on **5**) as slightly orange powders along with **S24** (800 mg, 1.3 mmol, 69% yield based on **6**) as a yellow oil.

Compound **7**$_{\textbf{\textit{trans}}}$:

^{1}H NMR (500 MHz, $CDCl_3$, 298 K) δ = 8.39 (s, 2H, H_2), 8.23 (s, 1H, H_4), 6.98 – 6.78 (m, 10H, $H_{h,m,o}$), 4.83 – 4.72 (m, 1H, H_d), 4.48 – 4.38 (m, 1H, H_d), 4.34 – 4.25 (m, 1H, H_e), 4.15 – 4.04 (m, 1H, H_e), 3.94 (t, J = 7.3 Hz, 2H, H_u), 3.85 – 3.58 (m, 37H, $H_{j,q,k}$), 3.57 – 3.49 (m, 2H, H_{ff}), 1.99 – 1.94 (s, s, 12H, $H_{a,r}$), 1.26 – 1.16 (m, 2H, H_v), 0.80 – 0.70 (m, 2H, H_z), 0.70 – 0.60 (m, 2H, H_y), 0.60 – 0.49 (m, 2H, H_{aa}), 0.46 – 0.36 (m, 2H, H_w), 0.37 – 0.27 (m, 2H, H_x), 0.22 (p, J = 8.3 Hz, 2H, H_{ee}), 0.03 – -0.10 (m, 2H, H_{bb}), -0.94 – -1.06 (m, 2H, H_{cc}), -1.19 – -1.32 (m, 2H, H_{dd}).
^{13}C NMR (126 MHz, $CDCl_3$, 298 K) δ = 171.86 (C_c), 171.74 (C_t), 150.94, 150.44, 150.41, 150.40, 150.35, 150.31, 150.28 ($C_{i,p}$), 149.12 (C_f), 140.06 (C_1), 133.38 (q, J = 34.7 Hz, C_3), 129.27, 128.49, 128.45, 128.38, 128.32, 128.28, 128.26, 128.07 – 128.89 ($C_{g,l,n,2,6,17}$), 127.19 (p, J = 3.7 Hz, C_4), 122.47 (q, J = 273.5 Hz, C_5), 115.24, 113.67, 113.49, 113.43, 113.35, 113.27, 113.11 ($C_{h,m,o}$), 72.52 (C_{ff}), 66.64 (C_u), 66.50 (C_e), 64.61 (C_d), 56.07, 55.76, 55.68, 55.62, 55.47, 55.43, 55.40, 55.36, 55.30 ($C_{b,s,j}$) 30.95, 30.91, 30.89, 30.62, 30.39, 30.03, 29.83, 29.38, 29.25, 29.12, 28.98, 28.96 ($C_{a,r,k,x\text{-}bb}$), 28.76 (C_{ee}), 28.48 (C_{cc}), 28.32 (C_v), 24.78 (C_w), 22.91 (C_{dd}).
^{19}F NMR (471 MHz, $CDCl_3$, 298 K) δ = -62.88 (s, 6F, F_5).
HRMS-ESI (+): 1579.4040 $[M+Na]^+$, calculated for $C_{74}H_{90}Br_2F_6O_{17}SNa^+$: 1579.4062.

Compound **7**$_{\textbf{\textit{cis}}}$:

^{1}H NMR (500 MHz, $CDCl_3$, 298 K) δ = 8.40 (s, 2H, H_2), 8.23 (s, 1H, H_4), 6.95 – 6.79 (m, 10H, $H_{h,m,o}$), 4.78 – 4.68 (m, 1H, H_d), 4.47 – 4.37 (m, 1H, H_d), 4.28 – 4.21 (m, 1H, H_e), 4.15 – 4.05 (m, 1H, H_e), 3.86 (t, J = 7.5 Hz, 2H, H_u), 3.83 – 3.67 (m, 37H, $H_{j,q,k}$), 3.65 – 3.55 (m, 2H, H_{ff}), 2.01 – 1.94 (s, s, 12H, $H_{a,r}$), 1.07 (p, J = 7.4 Hz, 2H, H_v), 0.78 - 0.46 (m, 6H, $H_{y,z,aa}$), 0.38 (p, J = 8.0 Hz, 2H, H_{ee}), 0.19 – 0.01 (m, 6H, $H_{w,x,bb}$), -0.72 – -0.82 (m, 2H, H_{cc}), -0.93 – -1.04 (m, 2H, H_{dd}).
^{13}C NMR (126 MHz, $CDCl_3$, 298 K) δ = 171.86 (C_c), 171.74 (C_t), 150.91, 150.44, 150.39, 150.36, 150.34, 150.33, 150.30, 150.26 ($C_{i,p}$), 149.12 (C_f), 140.06 (C_1), 133.38 (q, J = 34.7 Hz, C_3), 129.13, 128.54, 128.44, 128.42, 128.39, 128.24, 128.15, 128.09, 128.07 – 127.94 ($C_{g,l,n,2}$), 127.19 (p, J = 3.7 Hz, C_4), 122.47 (q, J = 273.4 Hz, C_5), 115.11, 113.55, 113.51, 113.43, 113.30, 113.27, 113.23, 113.12, 113.09 ($C_{h,m,o}$), 72.52 (C_{ff}), 66.64 (C_u), 66.50 (C_e), 64.61 (C_d), 56.10, 55.63, 55.55, 55.49, 55.47, 55.39, 55.37 ($C_{b,s,j}$), 30.95, 30.90, 30.88, 30.70, 30.68, 30.41, 30.04, 29.82, 29.32, 29.26, 29.13, 28.97, 28.93 ($C_{a,r,k,x\text{-}bb}$), 28.76 (C_{ee}), 28.48 (C_{cc}), 28.32 (C_v), 24.78 (C_w), 22.91 (C_{dd}).
^{19}F NMR (471 MHz, $CDCl_3$, 298 K) δ = -62.92 (s, 6F, F_5).
HRMS-ESI (+): 1579.4041 $[M+Na]^+$, calculated for $C_{74}H_{90}Br_2F_6O_{17}SNa^+$: 1579.4062.

Compound **S24**:

^{1}H NMR (500 MHz, $CDCl_3$, 298 K) δ = 8.35 (s, 2H, H_2), 8.15 (s, 1H, H_4), 4.18 (t, J = 6.5 Hz, 2H, H_o), 4.16 (t, J = 6.7 Hz, 2H , H_d), 1.93 (s, 6H, H_a), 1.75 – 1.63 (m, 4H, $H_{e,n}$), 1.44 – 1.19 (m, 16H, $H_{f\text{-}m}$).
^{13}C NMR (126 MHz, $CDCl_3$, 298 K) δ = 171.89 (C_t), 139.44 (C_1), 133.37 (q, J = 34.8 Hz, C_3), 128.21 (q, J = 3.6 Hz, C_2), 127.38 (p, J = 3.7 Hz, C_4), 122.42 (q, J = 273.6 Hz, C_5), 72.48 (C_o), 66.28 (C_d), 56.16 (C_b), 30.92 (C_a), 29.56, 29.55, 29.53, 29.45, 29.27, 29.02, 29.00 ($C_{g\text{-}l,n}$), 28.47 (C_e), 25.90 (C_f), 25.43 (C_m).
^{19}F NMR (471 MHz, $CDCl_3$, 298 K) δ = -62.96 (s, 6F, F_5).
HRMS-ESI(+): 627.1201 $[M+H]^+$, calculated for $C_{24}H_{33}BrF_6O_5SH^+$: 627.1209.

4.3 Determination of the *cis/trans* Isomerism of Rotaxane Precursors 7

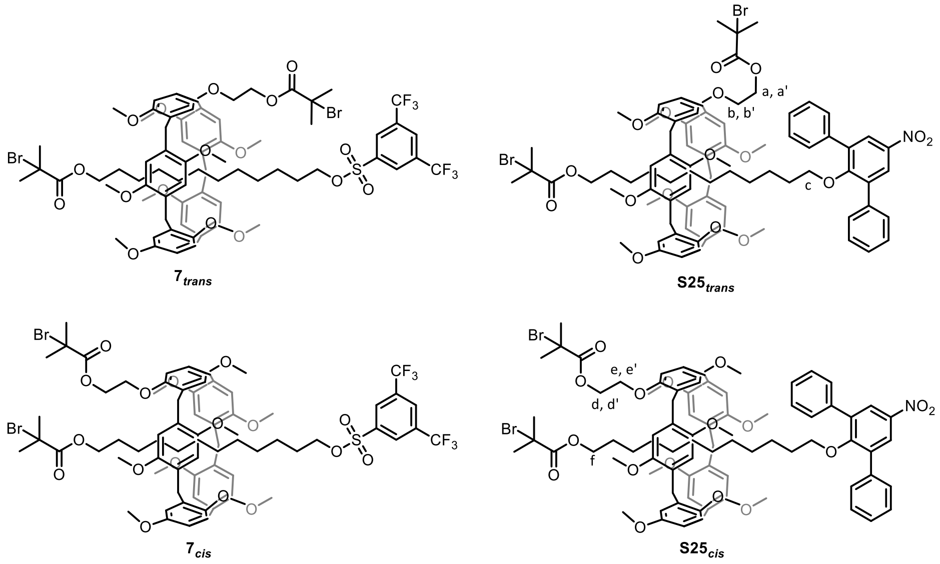

Figure S9. *cis*- and *trans*-structures of both rotaxane precursor **7** and rotaxane **S25**.

As we couldn't determine the relative orientation of the macrocycle in rotaxanes **7**, we prepared rotaxanes **S25** (made from **7**, see *Sections 4.3.1* and *4.3.2*) where the bulkier nitro-2,6-diphenylphenol stopper reduces the mobility of the macrocycle. 1D selective ROESY was used to assign the isomerism of **S25*trans*** and **S25*cis*** and, by extension, **7*trans*** and **7*cis***. For **S25*trans***, selective inversion of proton H_a found on the macrocycle linker demonstrates a clear correlation with proton H_c found on the rotaxane axle (*Figure S10*), hence demonstrating the *trans* isomerism. Likewise, for **S25*cis***, selective inversion of protons H_d, $H_{d'}$, or H_e (all found on the macrocycle linker) demonstrates a correlation with proton H_f found on the rotaxane axle (*Figure S11*), hence confirming the *cis* isomerism. By relation, the precursor compounds to the two **S25** isomers, **7**, can have their isomerisms assigned respectively.

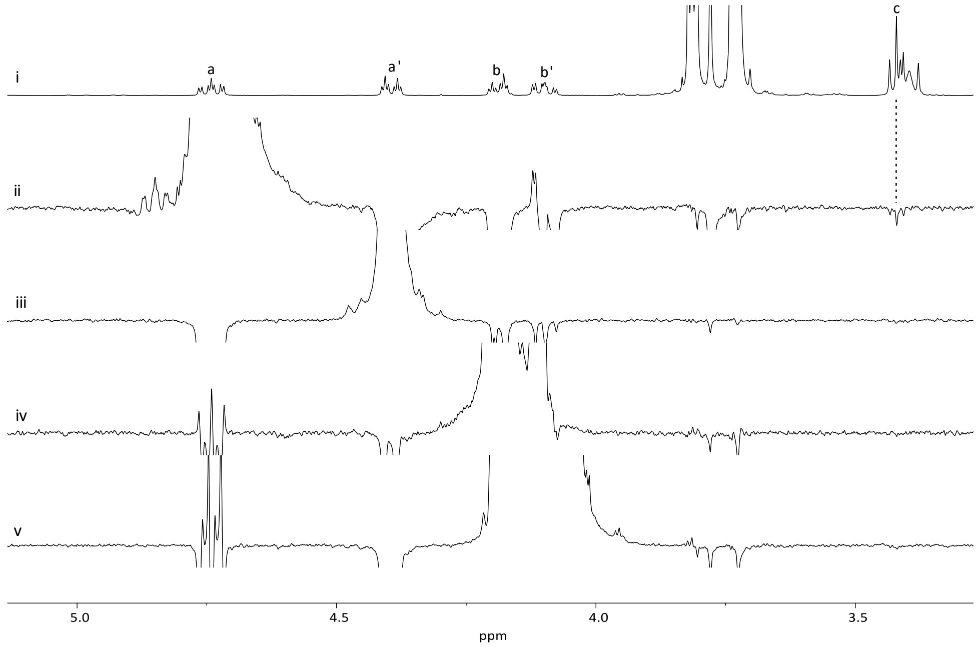

Figure S10. Partial ^{1}H NMR (500 MHz, Acetone-d_6, 298 K) spectrum of rotaxane **S25*trans*** (i) and partial 1D selective ROESY ^{1}H NMR (500 MHz, Acetone-d_6, 298 K) spectra of rotaxane **S25*trans*** with selectively inverted protons H_a (ii), $H_{a'}$ (iii), H_b (iv) and $H_{b'}$ (v).

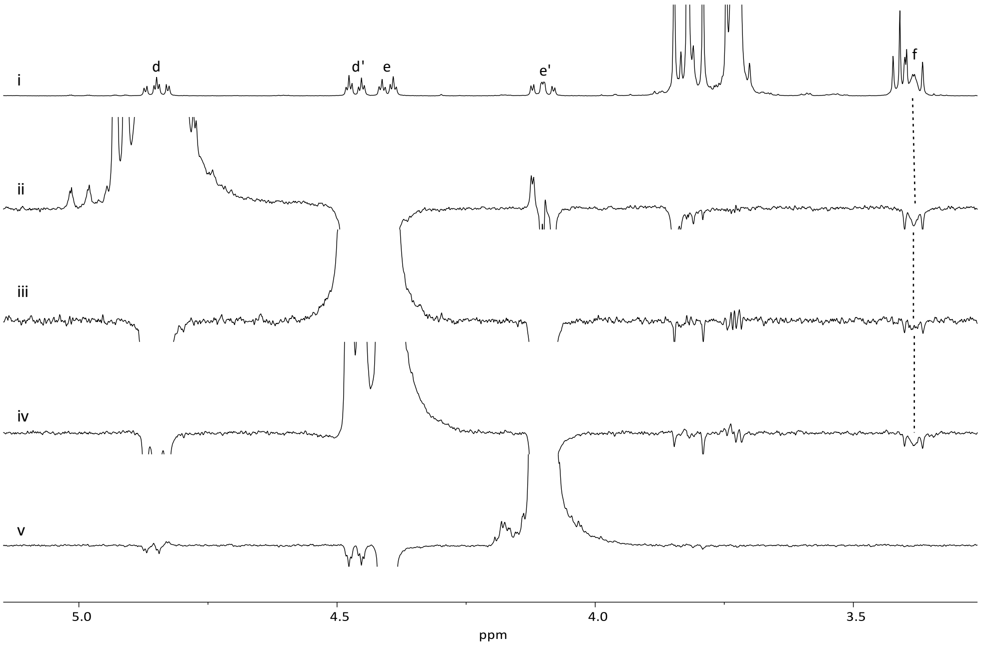

Figure S11. Partial ^{1}H NMR (500 MHz, Acetone-d_6, 298 K) spectrum of rotaxane **S25$_{cis}$** (i) and partial 1D selective ROESY ^{1}H NMR (500 MHz, Acetone-d_6, 298 K) spectra of rotaxane **S25$_{cis}$** with selectively inverted protons H_d (ii), $H_{d'}$ (iii), H_e (iv) and $H_{e'}$ (v).

4.3.1 Synthesis of S25$_{trans}$

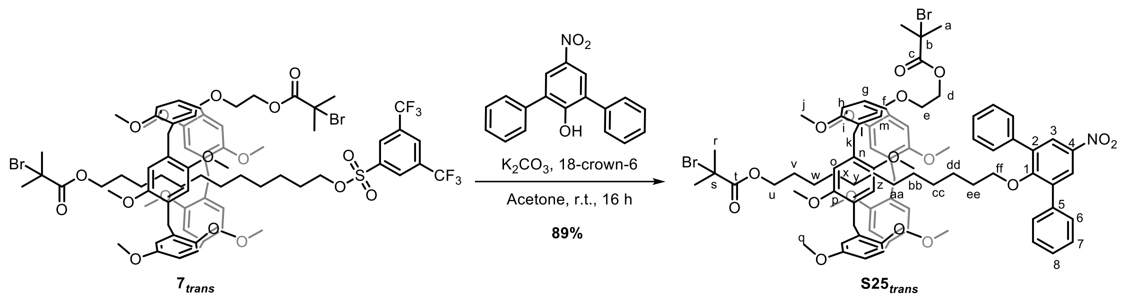

7$_{trans}$ **S25$_{trans}$**

To a solution of 4-nitro-2,6-diphenylphenol (1.7 mg, 5.8 µmol, 1.0 eq.) in acetone (1 mL) was added K_2CO_3 (0.8 mg, 5.8 µmol, 1.0 eq.) and 18-crown-6 (1.5 mg, 5.8 µmol, 1.0 eq.). The mixture was then stirred for 2 h at room temperature. **7$_{trans}$** (9 mg, 5.8 µmol, 1.0 eq.) was added and the mixture stirred for a further 16 h at room temperature. The solution was filtered, and the filtrate concentrated under vacuum. The residue was purified by preparative TLC (500 µm, DCM/acetone, 8/1) to yield **S25$_{trans}$** as a white powder (8 mg, 5.1 µmol, 89% yield).

^{1}H NMR (500 MHz, Acetone-d_6, 298 K) δ = 8.24 (s, 2H, H_3), 7.78 – 7.74 (m, 4H, H_6), 7.63 – 7.58 (m, 4H, H_7), 7.56 – 7.51 (m, 2H, H_8), 6.97 – 6.86 (m, 10H, $H_{h,m,o}$), 4.73 (ddd, J = 12.1, 9.1, 3.1 Hz, 1H, H_d), 4.38 (dt, J = 11.8, 3.2 Hz, 1H, H_d), 4.17 (dt, J = 11.0, 3.3 Hz, 1H, H_e), 4.08 (ddd, J = 10.9, 9.1, 3.0 Hz, 1H, H_e), 3.82 – 3.70 (m, 37H, $H_{j,k,q}$), 3.41 (t, J = 6.6 Hz, 2H, H_{ff}), 3.40 – 3.35 (m, 2H, H_u), 1.98 – 1.96 (m, 12H, $H_{a,r}$), 1.32 – 1.25 (m, 2H, H_{ee}), 1.00 – 0.84 (m, 4H, $H_{cc,dd}$), 0.83 – 0.72 (m, 2H, H_{bb}), 0.57 – 0.48 (m, 2H, H_{aa}), 0.20 – 0.06 (m, 4H, $H_{v,z}$), -0.61 – -0.69 (m, 2H, H_y), -1.73 – -1.87 (m, 4H, $H_{w,x}$).

^{13}C NMR (126 MHz, Acetone-d_6, 298 K) δ = 172.07 (C_c), 171.90 (C_t), 160.54 (C_1), 151.62, 151.15, 151.10, 151.05, 151.02 ($C_{i,p}$), 149.94 (C_f), 144.76 (C_4), 138.12 (C_2), 137.91 (C_5), 130.30 (C_6), 129.42 (C_7), 129.15 (C_8), 128.85, 128.84, 128.81, 128.71, 128.69, 128.68, 128.63, 128.61, 128.49 ($C_{g,l,n}$), 125.90 (C_3), 115.72, 114.18, 113.83, 113.70, 133.67, 113.55, 113.53, 113.50, 113.40, 113.37 ($C_{h,m,o}$), 74.74 (C_{ff}), 67.93 (C_u),

67.25 (C_e), 65.22 (C_d), 57.43, 57.06 ($C_{s,b}$), 55.83, 55.81, 55.79, 55.75, 55.69, 55.64, 55.61 ($C_{j,q}$), 31.43 (C_{aa}), 31.21, 31.18, 31.04 ($C_{a,r}$), 30.96 (C_{bb}), 30.80, 30.77, 30.74 ($C_{y,z,cc}$), 30.51 (C_{ee}), 30.10 – 29.35 (C_k, overlapped with residual solvent signal), 28.83 (C_v), 28.40 (C_x), 26.63 (C_{dd}), 23.57 (C_w).
HRMS-ESI(+): 1574.5213 $[M+Na]^+$, calculated for $C_{84}H_{99}Br_2NO_{17}Na^+$: 1574.5172.

4.3.2 Synthesis of S25$_{cis}$

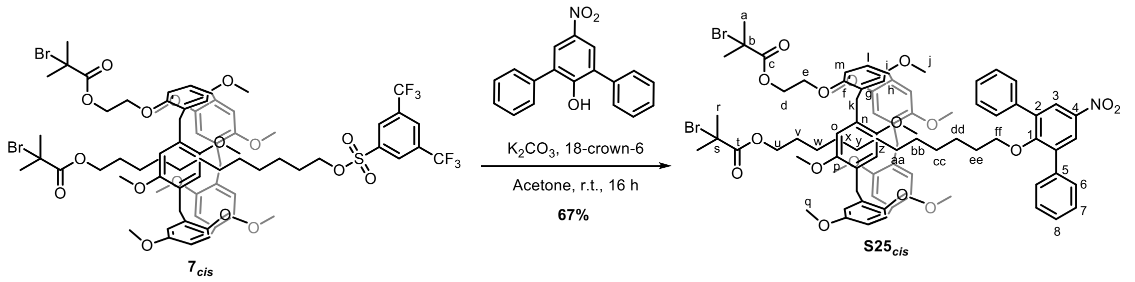

To a solution of 4-nitro-2,6-diphenylphenol (1.7 mg, 5.8 µmol, 1.0 eq.) in acetone (1 mL) was added K_2CO_3 (0.8 mg, 5.8 µmol, 1.0 eq.) and 18-crown-6 (1.5 mg, 5.8 µmol, 1.0 eq.). The mixture was then stirred for 2 h at room temperature. **7**$_{cis}$ (9 mg, 5.8 µmol, 1.0 eq.) was added and the mixture stirred for a further 16 h at room temperature. The solution was filtered, and the filtrate concentrated under vacuum. The residue was purified by preparative TLC (500 µm, DCM/acetone, 8/1) to yield **S25**$_{cis}$ as a white powder (6 mg, 3.9 µmol, 67% yield).

^{1}H NMR (500 MHz, Acetone-d_6, 298 K) δ = 8.24 (s, 2H, H_3), 7.78 – 7.74 (m, 4H, H_6), 7.63 – 7.57 (m, 4H, H_7), 7.56 – 7.52 (m, 2H, H_8), 7.01 – 6.83 (m, 10H, $H_{h,m,o}$), 4.83 (ddd, J = 12.2, 9.5, 2.8 Hz, 1H, H_d), 4.45 (dt, J = 12.0, 2.9 Hz, 1H, H_d), 4.39 (dt, J = 10.8, 3.0 Hz, 1H, H_e), 4.09 (ddd, J = 10.8, 9.6, 2.7 Hz, 1H, H_e), 3.85 – 3.69 (m, 37H, $H_{j,k,q}$), 3.40 (t, J = 6.6 Hz, 2H, H_{ff}), 3.38 – 3.34 (m, 2H, H_u), 1.99 (s, 6H, H_r), 1.98 – 1.96 (s, s, 6H, H_a), 1.34 – 1.25 (m, 2H, H_{ee}), 0.99 – 0.83 (m, 4H, $H_{cc,dd}$), 0.83 – 0.76 (m, 2H, H_{bb}), 0.61 – 0.49 (m, 2H, H_{aa}), 0.20 – 0.05 (m, 4H, $H_{v,z}$), -0.56 – -0.67 (m, 2H, H_y), -1.78 – -1.89 (m, 4H, $H_{w,x}$).
^{13}C NMR (126 MHz, Acetone-d_6, 298 K) δ = 172.10, 172.07 ($C_{c,t}$), 160.53 (C_1), 151.59, 151.14, 151.11, 151.10, 151.06, 151.01, 151.00 ($C_{i,p}$), 149.95 (C_f), 144.77 (C_4), 138.12 (C_2), 137.91 (C_5), 130.29 (C_6), 129.53, 128.90, 128.81, 128.72, 128.67, 128.62, 128.55, 128.52 ($C_{g,l,n}$), 129.40 (C_7), 129.13 (C_8), 125.90 (C_3), 115.30, 113.95, 113.92, 113.83, 113.69, 113.66, 113.62, 113.48, 113.45, 113.36 ($C_{h,m,o}$), 74.71 (C_{ff}), 68.04 (C_u), 67.22 (C_e), 65.54 (C_d), 57.45 (C_s), 57.15 (C_b), 55.95, 55.77, 55.73, 55.66, 55.59, 55.56 ($C_{j,q}$), 31.40 (C_{aa}), 31.23, 31.20, 31.07, 31.06 ($C_{a,r}$), 30.88 ($C_{y,bb}$), 30.75, 30.73 ($C_{z,cc}$), 30.47 – 30.35 (C_{ee}, overlapped with residual solvent signal), 30.17 – 29.44 (C_k, overlapped with residual solvent signal), 28.76 (C_v), 28.33 (C_x), 26.56 (C_{dd}), 23.49 (C_w).
HRMS-ESI(+): 1574.5260 $[M+Na]^+$, calculated for $C_{84}H_{99}Br_2NO_{17}Na^+$: 1574.5172.

4.4 Synthesis of Control Compounds, S26

4.4.1 Synthesis of S26*exo*

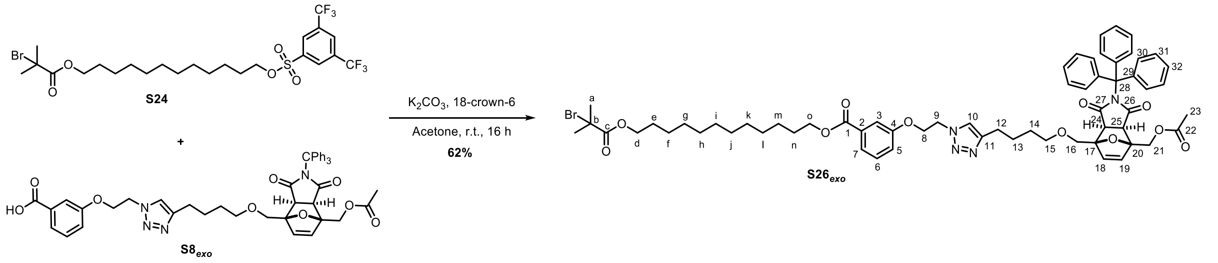

To a solution of **S8*exo*** (4.6 mg, 5.8 µmol, 1.0 eq.) in acetone (1 mL) was added K_2CO_3 (0.8 mg, 5.8 µmol, 1.0 eq.) and 18-crown-6 (1.5 mg, 5.8 µmol, 1.0 eq.). The mixture was stirred for 2 h at room temperature. **S24** (3.6 mg, 5.8 µmol, 1.0 eq.) was added and the mixture stirred for a further 16 h at room temperature. The solution was filtered, and the filtrate concentrated under vacuum. The residue was purified by preparative TLC (500 µm, DCM/MeOH, 30/1) to yield **S26*exo*** as a white powder (4 mg, 3.5 µmol, 62% yield).

^{1}H NMR (500 MHz, Acetone-d_6, 298 K) δ = 7.80 (s, 1H, H_{10}), 7.61 (dt, J = 7.6, 1.2 Hz, 1H, H_7), 7.53 (dd, J = 2.7, 1.5 Hz, 1H, H_3), 7.50 – 7.45 (m, 6H, H_{30}), 7.41 (t, J = 8.0 Hz, 1H, H_6), 7.29 – 7.23 (m, 6H, H_{31}), 7.19 (ddd, J = 8.2, 2.7, 0.9 Hz, 1H, H_5), 7.18 – 7.13 (m, 3H, H_{32}), 6.61 (d, J = 5.6 Hz, 1H, H_{19}), 6.52 (d, J = 5.6 Hz, 1H, H_{18}), 4.79 (t, J = 5.1 Hz, 2H, H_x), 4.72 (d, J = 1.3 Hz, 2H, H_{21}), 4.51 (t, J = 5.1 Hz, 2H, H_w), 4.29 (t, J = 6.6 Hz, 2H, H_o), 4.15 (t, J = 6.6 Hz, 2H, H_d), 4.15 (d, J = 11.5 Hz, 2H, H_{16}), 3.90 (d, J = 11.6, 1H, H_{16}), 3.67 (dt, J = 9.4, 6.2 Hz, 1H, H_{15}), 3.56 (dt, J = 9.4, 6.3 Hz, 1H, H_{15}), 2.99 – 2.94 (d, J = 6.6 Hz, d, J = 6.5 Hz, 2H, $H_{24,25}$), 2.71 (t, J = 7.5 Hz, 2H, H_{12}), 2.05 (s, 3H, H_{23}), 1.92 (s, 6H, H_a), 1.79 – 1.72 (m, 4H, $H_{n,13}$), 1.70 – 1.62 (m, 4H, $H_{e,14}$), 1.49 – 1.25 (m, 16H, $H_{f\text{-}l}$).
^{13}C NMR (126 MHz, Acetone-d_6, 298 K) δ = 173.90 (C_{27}), 173.89 (C_{26}), 171.83 (C_c), 170.72 (C_{22}), 166.46 (C_1), 159.36 (C_4), 148.25 (C_{11}), 143.39 (C_{29}), 139.52 (C_{19}), 137.93 (C_{18}), 132.93 (C_2), 130.65 (C_6), 129.36 (C_{30}), 128.27 (C_{31}), 127.27 (C_{32}), 122.98 (C_7), 122.85 (C_{10}), 120.21 (C_5), 115.96 (C_3), 92.32 (C_{17}), 90.75 (C_{20}), 74.27 (C_{28}), 72.14 (C_{15}), 68.98 (C_{16}), 67.77 (C_8), 66.51 (C_d), 65.70 (C_o), 62.24 (C_{21}), 57.51 (C_b), 51.39 ($C_{24,25}$), 49.98 (C_9), 30.98 (C_a), 30.49 – 29.34 ($C_{g\text{-}l,n,14}$, overlapped with residual solvent signal), 29.09 (C_e), 26.88 (C_{13}), 26.72 (C_m), 26.51 (C_f), 25.96 (C_{12}), 20.70 (C_{23}).
HRMS-ESI(+): 1151.4331 $[M+Na]^+$, calculated for $C_{62}H_{73}BrN_4O_{11}Na^+$: 1151.4351.

4.4.1 Synthesis of S26*endo*

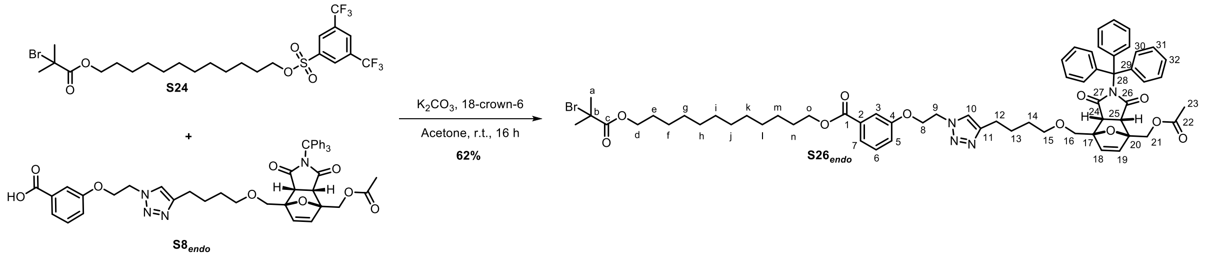

To a solution of **S8*endo*** (4.6 mg, 5.8 µmol, 1.0 eq.) in acetone (1 mL) was added K_2CO_3 (0.8 mg, 5.8 µmol, 1.0 eq.) and 18-crown-6 (1.5 mg, 5.8 µmol, 1.0 eq.). The mixture was stirred for 2 h at room temperature. **S24** (3.6 mg, 5.8 µmol, 1.0 eq.) was added and the mixture stirred for a further 16 h at room temperature. The solution was filtered, and the filtrate concentrated under vacuum. The residue was purified by preparative TLC (500 µm, DCM/MeOH, 30/1) to yield **S26*endo*** as a white powder (4 mg, 3.5 µmol, 62% yield).

^{1}H NMR (400 MHz, Acetone-d_6, 298 K) δ = 7.79 (s, 1H, H_{10}), 7.62 (dt, J = 7.7, 1.2 Hz, 1H, H_7), 7.53 (dd, J = 2.8, 1.5 Hz, 1H, H_3), 7.47 – 7.42 (m, 7H, H_{30}), 7.42 (t, J = 7.9 Hz, 1H, H_6), 7.25 – 7.18 (m, 7H, $H_{5,32}$), 7.16

– 7.11 (m, 3H, H_{32}), 5.46 – 5.41 (m, 2H, $H_{18,19}$), 4.78 (t, J = 4.9 Hz, 2H, H_9), 4.58 (d, J = 12.8 Hz, 1H, H_{21}), 4.51 (t, J = 5.1 Hz, 2H, H_8), 4.38 (d, J = 12.7 Hz, 1H, H_{21}), 4.29 (t, J = 6.6 Hz, 2H, H_o), 4.16 (t, J = 6.5 Hz, 2H, H_d), 3.88 (d, J = 12.0 Hz, 1H, H_{16}), 3.81 (d, J = 12.0 Hz, 1H, H_{16}), 3.72 (d, J = 8.3 Hz, 1H, H_{24}), 3.65 (d, J = 8.3 Hz, 1H, H_{25}), 3.49 (t, J = 6.3 Hz, 2H, H_{15}), 2.67 (t, J = 7.4 Hz, 2H, H_{12}), 1.97 (s, 3H, H_{23}), 1.92 (s, 6H, H_a), 1.80 – 1.73 (m, 2H, H_n), 1.72 – 1.63 (m, 4H, $H_{e,13}$), 1.60 – 1.55 (m, 2H, H_{14}), 1.48 – 1.24 (m, 16H, H_{f-l}).
^{13}C NMR (126 MHz, Acetone-d_6, 298 K) δ = 175.33 (C_{26}), 175.13 (C_{27}), 171.84 (C_c), 170.58 (C_{22}), 166.47 (C_1), 159.37 (C_4), 148.18 (C_{11}), 143.39 (C_{29}), 136.87 (C_{18}), 135.76 (C_{19}), 132.94 (C_2), 130.66 (C_6), 129.35 (C_{30}), 128.07 (C_{31}), 127.09 (C_{32}), 122.99 (C_7), 122.85 (C_{10}), 120.24 (C_5), 115.96 (C_3), 92.31 (C_{17}), 90.25 (C_{20}), 74.60 (C_{28}), 71.90 (C_{15}), 69.33 (C_{16}), 67.77 (C_8), 66.51 (C_d), 65.71 (C_o), 62.83 (C_{21}), 57.52 (C_b), 49.98 (C_9), 48.83 (C_{25}), 47.99 (C_{24}), 30.98 (C_a), 30.48 – 29.31 ($C_{g-l,n,14}$, overlapped with residual solvent peak), 29.09 (C_e), 26.82 (C_{13}), 26.73 (C_m), 26.51 (C_f), 25.91 (C_{aa}), 20.57 (C_{23}).
HRMS-ESI(+): 1151.4331 $[M+Na]^+$, calculated for $C_{62}H_{73}BrN_4O_{11}Na^+$: 1151.4351.

4.5 Synthesis of 1-Cargo Rotaxanes

4.5.1 Synthesis of S27*trans/exo*

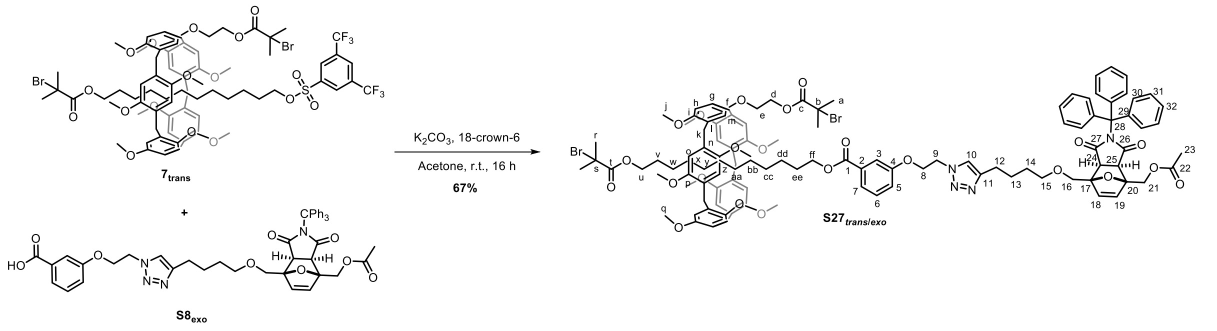

To a solution of **S8*exo*** (4.6 mg, 5.8 µmol, 1.0 eq.) in acetone (1 mL) was added K_2CO_3 (0.8 mg, 5.8 µmol, 1.0 eq.) and 18-crown-6 (1.5 mg, 5.8 µmol, 1.0 eq.). The mixture was stirred for 2 h at room temperature. **7*trans*** (9 mg, 5.8 µmol, 1.0 eq.) was added and the mixture stirred for a further 16 h at room temperature. The solution was filtered, and the filtrate concentrated under vacuum. The residue was purified by preparative TLC (500 µm, DCM/MeOH, 30/1) to yield **S27*trans/exo*** as a white powder (8 mg, 3.9 µmol, 67% yield).

^{1}H NMR (500 MHz, DMSO-d_6, 298 K) δ = 7.93 (s, 1H, H_{10}), 7.58 (dt, J = 7.7, 1.2 Hz, 1H, H_7), 7.49 (t, J = 8.0 Hz, 1H, H_6), 7.44 (dd, J = 2.7, 1.5 Hz, 1H, H_3), 7.38 – 7.32 (m, 6H, H_{30}), 7.27 – 7.22 (m, 7H, $H_{5,31}$), 7.19 – 7.12 (m, 3H, H_{32}), 6.87 – 6.76 (m, 10H, $H_{h,m,o}$), 6.51 (d, J = 5.5 Hz, 1H, H_{19}), 6.46 (d, J = 5.6 Hz, 1H, H_{18}), 4.74 (t, J = 5.0 Hz, 2H, H_9), 4.65 (d, J = 12.8 Hz, 1H, H_{21}), 4.62 – 4.58 (m, 1H, H_d), 4.53 – 4.48 (m, 1H, H_{21}), 4.48 – 4.44 (m, 2H, H_8), 4.31 (dt, J = 11.8, 3.1 Hz, 1H, H_d), 4.13 (dt, J = 11.0, 3.3 Hz, 1H, H_e), 4.04 (dd, J = 11.8, 1.9 Hz, 1H, H_{16}), 3.99 (ddd, J = 11.6, 9.1, 2.9 Hz, 1H, H_e), 3.78 – 3.60 (m, 42H, $H_{j,k,q,u,ff,16}$), 3.58 – 3.43 (m, 2H, H_{15}), 2.98 (d, J = 6.5 Hz, 1H, H_{25}), 2.92 (dd, J = 6.5, 2.9 Hz, 1H, H_{24}), 2.63 (t, J = 7.4 Hz, 2H, H_{12}), 2.04 (s, 3H, H_{23}), 1.92 (s, 6H, H_r), 1.90 – 1.87 (s, s, 6H, H_a), 1.68 – 1.61 (m, 2H, H_{13}), 1.59 – 1.53 (m, 2H, H_{14}), 0.87 – 0.82 (m, 2H, H_v), 0.71 – 0.67 (m, 2H, H_{ee}), 0.43 – 0.34 (m, 4H, $H_{z,aa}$), 0.19 – 0.15 (m, 2H, H_y), 0.12 – 0.09 (m, 2H, H_{bb}), -0.22 – -0.28 (m, 2H, H_w), -0.32 – -0.41 (m, 6H, $H_{x,cc,dd}$).
^{13}C NMR (126 MHz, DMSO-d_6, 298 K) δ = 173.15 (C_{26}), 173.06 (C_{27}), 170.89 (C_c), 170.77 (C_t), 170.06 (C_{22}), 165.42 (C_1), 158.01 (C_4), 150.16, 149.70, 149.67, 149.64, 149.60, 149.58, 149.56 ($C_{i,p}$), 148.59 (C_f), 146.82 (C_{11}), 141.97 (C_{29}), 138.37 (C_{19}), 137.05 (C_{18}), 131.34 (C_2), 130.09 (C_6), 128.00 (C_{30}), 127.47 (C_{31}), 127.32, 127.29, 127.16, 127.10, 127.07, 127.06, 126.00 ($C_{g,l,n}$), 126.42 (C_{32}), 122.32 (C_{10}), 121.65 (C_7), 119.25 (C_5), 115.02 (C_3), 114.44, 112.82 – 112.12 ($C_{h,m,o}$), 90.96 (C_{17}), 89.22 (C_{20}), 72.83 (C_{28}), 70.83 (C_{15}), 67.74 (C_{16}), 66.50 (C_8), 66.05 ($C_{e,u}$), 65.20 (C_{ff}), 64.21 (C_d), 61.27 (C_{21}), 57.30 (C_s), 56.92 (C_b), 54.93, 54.89, 54.85, 54.77, 54.71 (C_j), 50.06 ($C_{24,25}$), 48.81 (C_9), 30.30, 30.26, 30.23, 30.19 ($C_{a,r}$), 29.83, 29.67, 29.51, 29.44 ($C_{z,y,aa,bb}$), 29.06 – 28.40 ($C_{k,14}$), 28.23 ($C_{x,cc}$), 27.98 (C_{ee}), 27.70 (C_v), 25.60 (C_{13}), 24.75 (C_{12}), 23.82 ($C_{w,dd}$), 20.58 (C_{23}).
HRMS-ESI(+): 2079.7319 [M+Na]$^+$, calculated for $C_{112}H_{130}Br_2N_4O_{23}Na^+$: 2079.7385.

4.5.2 Synthesis of S27*cis/exo*

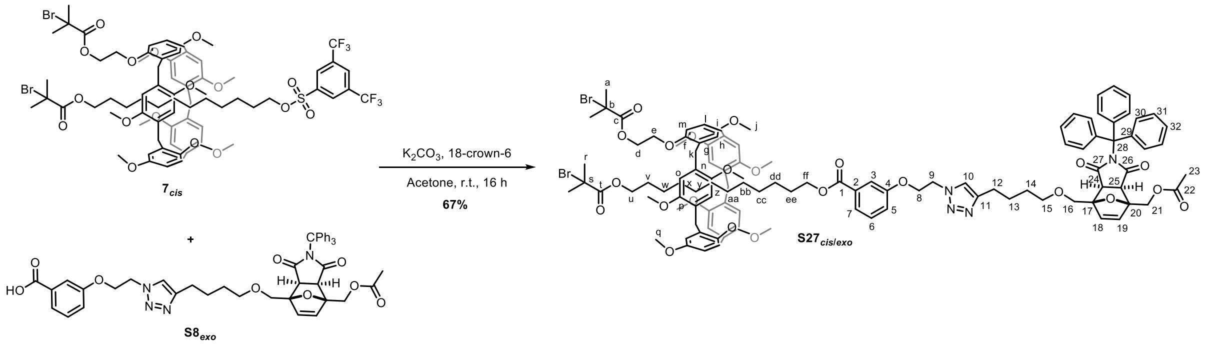

To a solution of **S8*exo*** (4.6 mg, 5.8 µmol, 1.0 eq.) in acetone (1 mL) was added K_2CO_3 (0.8 mg, 5.8 µmol, 1.0 eq.) and 18-crown-6 (1.5 mg, 5.8 µmol, 1.0 eq.). The mixture was stirred for 2 h at room temperature. **7*cis*** (9 mg, 5.8 µmol, 1.0 eq.) was added and the mixture stirred for a further 16 h at room temperature. The solution was filtered, and the filtrate concentrated under vacuum. The residue was purified by preparative TLC (500 µm, DCM/MeOH, 30/1) to yield **S27*cis/exo*** as a white powder (8 mg, 3.9 µmol, 67% yield).

^{1}H NMR (500 MHz, DMSO-d_6, 298 K) δ = 7.93 (s, 1H, H_{10}), 7.58 (dt, J = 7.8, 1.2 Hz, 1H, H_7), 7.48 (t, J = 7.9 Hz, 1H, H_6), 7.44 (dd, J = 2.7, 1.5 Hz, 1H, H_3), 7.37 – 7.33 (m, 6H, H_{30}), 7.25 (m, 7H, $H_{5,31}$), 7.18 – 7.14 (m, 3H, H_{32}), 6.88 – 6.77 (m, 10H, $H_{h,m,o}$), 6.51 (d, J = 5.6 Hz, 1H, H_{19}), 6.46 (d, J = 5.6 Hz, 1H, H_{18}), 4.73 (t, J = 5.1 Hz, 2H, H_9), 4.71 – 4.66 (m, 1H, H_d), 4.65 (d, J = 12.8 Hz, 1H, H_{21}), 4.50 (d, J = 12.8 Hz, 1H, H_{21}), 4.47 (t, J = 5.1 Hz, 2H, H_8), 4.39 (dt, J = 11.9, 3.1 Hz, 1H, H_d), 4.19 – 4.14 (m, 1H, H_e), 4.05 – 4.00 (m, 2H, $H_{e,16}$), 3.86 – 3.79 (m, 2H, H_{ff}), 3.76 – 3.61 (m, 40H, $H_{j,k,q,u,16}$), 3.58 – 3.43 (m, 2H, H_{15}), 2.98 (d, J = 6.5 Hz, 1H, H_{25}), 2.92 (dd, J = 6.5, 2.1 Hz, 1H, H_{24}), 2.63 (t, J = 7.4 Hz, 2H, H_{12}), 2.03 (s, 3H, H_{23}), 1.93 – 1.90 (m, 12H, $H_{a,r}$), 1.68 – 1.61 (m, 2H, H_{13}), 1.59 – 1.52 (m, 2H, H_{14}), 0.88 – 0.81 (m, 2H, H_{ee}), 0.77 – 0.70 (m, 2H, H_v), 0.45 – 0.30 (m, 4H, $H_{z,aa}$), 0.24 – 0.16 (m, 2H, H_{bb}), 0.09 – 0.00 (m, 2H, H_y), -0.14 – -0.24 (m, 4H, $H_{cc,dd}$), -0.45 – -0.50 (m, 2H, H_w), -0.52 – -0.58 (m, 2H, H_x).

^{13}C NMR (126 MHz, DMSO-d_6, 298 K) δ = 173.14, 173.05 ($C_{26,27}$), 170.93, 170.83 ($C_{c,t}$), 170.05 (C_{22}), 165.38 (C_1), 158.02 (C_4), 150.18, 149.72, 149.65, 149.61, 149.56, 149.53 ($C_{i,p}$), 148.55 (C_f), 146.82 (C_{11}), 141.97 (C_{29}), 138.37 (C_{19}), 137.04 (C_{18}), 131.33 (C_2), 130.09 (C_6), 128.00 (C_{30}), 127.46 (C_{31}), 127.36 – 126.96 ($C_{g,l,n}$), 126.41 (C_{32}), 122.33 (C_{10}), 121.66 (C_7), 119.29 (C_5), 114.99 (C_3), 114.36, 112.62 – 112.06 ($C_{h,m,o}$), 90.96 (C_{17}), 89.22 (C_{20}), 72.82 (C_{28}), 70.82 (C_{15}), 67.73 (C_{16}), 66.50 (C_8), 66.13 (C_e), 65.14 (C_{ff}), 64.28 (C_d), 61.26 (C_{21}), 57.24 (C_s), 56.96 (C_b), 54.94, 54.88, 54.86, 54.82, 54.80, 54.75, 54.71 (C_j), 50.06 ($C_{25,24}$), 48.80 (C_9), 30.30, 30.29, 30.27, 30.25, 30.08, 29.92, 29.83, 29.77, 29.63, 29.54, 29.42, 29.01, 28.49, 28.30, 27.96, 27.84, 27.65 ($C_{a,k,r,x\text{-}z,aa\text{-}cc,ee,14}$), 25.59 ($C_{13}$), 24.73 ($C_{12}$), 24.04 ($C_{dd}$), 23.62 ($C_w$), 20.57 ($C_{23}$).

HRMS-ESI(+): 2079.7315 $[M+Na]^+$, calculated for $C_{112}H_{130}Br_2N_4O_{23}Na^+$: 2079.7385.

4.5.3 Synthesis of S27*trans/endo*

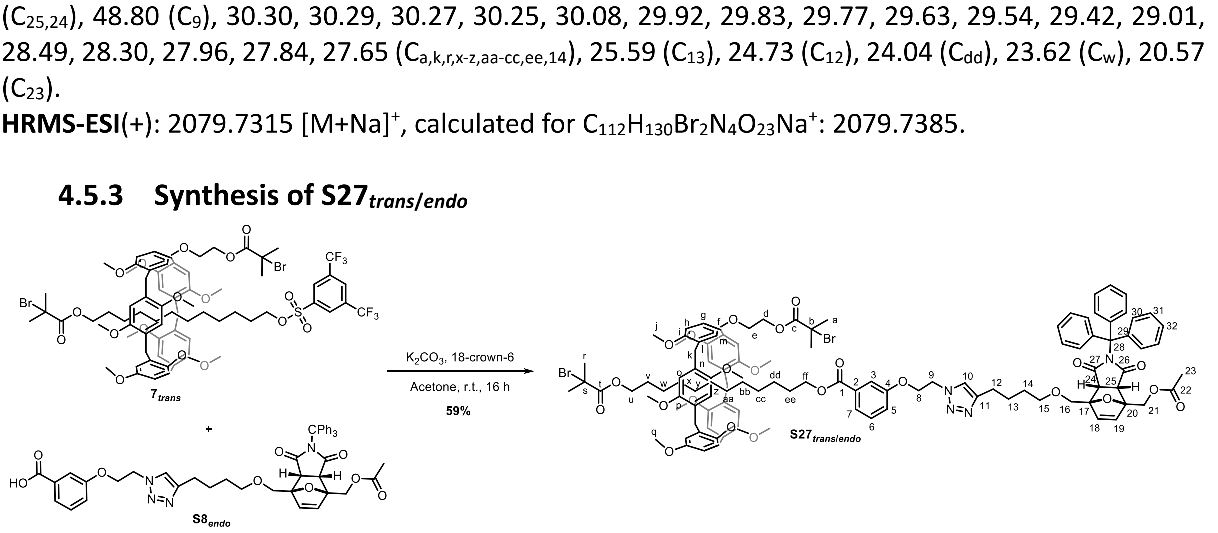

To a solution of **S8*endo*** (4.6 mg, 5.8 µmol, 1.0 eq.) in acetone (1 mL) was added K_2CO_3 (0.8 mg, 5.8 µmol, 1.0 eq.) and 18-crown-6 (1.5 mg, 5.8 µmol, 1.0 eq.). The mixture was stirred for 2 h at room temperature. **7*trans*** (9 mg, 5.8 µmol, 1.0 eq.) was added and the mixture stirred for a further 16 h at

room temperature. The solution was filtered, and the filtrate concentrated under vacuum. The residue was purified by preparative TLC (500 µm, DCM/MeOH, 30/1) to yield **S27*trans/endo*** as a white powder (7 mg, 3.4 µmol, 59% yield).

^{1}H NMR (500 MHz, DMSO-d_6, 298 K) δ = 7.92 (s, 1H, H_{10}), 7.58 (dt, *J* = 7.7, 1.2 Hz, 1H, H_7), 7.49 (t, *J* = 8.0 Hz, 1H, H_6), 7.43 (dd, *J* = 2.7, 1.5 Hz, 1H, H_3), 7.37 – 7.32 (m, 6H, H_{30}), 7.29 – 7.24 (m, 1H, H_5), 7.23 – 7.19 (m, 6H, H_{31}), 7.15 – 7.11 (m, 3H, H_{32}), 6.87 – 6.78 (m, 10H, $H_{\mathrm{h,m,o}}$), 5.33 – 5.28 (d, *J* = 5.7 Hz, d, *J* = 5.7 Hz, 2H, $H_{18,19}$), 4.73 (t, *J* = 5.1 Hz, 2H, H_9), 4.61 (td, *J* = 9.1, 4.6 Hz, 1H, H_{d}), 4.50 (d, *J* = 12.8 Hz, 1H, H_{21}), 4.47 (t, *J* = 5.1 Hz, 2H, H_8), 4.35 – 4.28 (m, 1H, H_{d}), 4.34 (d, *J* = 12.9 Hz, 1H, H_{21}), 4.17 – 4.07 (m, 1H, H_{e}), 4.02 – 3.95 (m, 1H, H_{e}), 3.81 – 3.57 (m, 47H, $H_{\mathrm{j,k,q,u,ff},16,24,25}$), 3.42 – 3.37 (m, 2H, H_{15}), 2.59 (t, *J* = 7.4 Hz, 2H, H_{12}), 1.98 (s, 3H, H_{23}), 1.92 (s, 6H, H_{r}), 1.89 – 1.86 (m, 6H, H_{a}), 1.60 – 1.55 (m, 2H, H_{13}), 1.53 – 1.46 (m, 2H, H_{14}), 0.87 – 0.82 (m, 2H, H_{v}), 0.74 – 0.64 (m, 2H, H_{ee}), 0.45 – 0.34 (m, 4H, $H_{\mathrm{z,aa}}$), 0.20 – 0.14 (m, 2H, H_{y}), 0.14 – 0.08 (m, 2H, H_{bb}), -0.21 – -0.28 (m, 2H, H_{w}), -0.30 – -0.36 (m, 2H, H_{x}), -0.37 – -0.41 (m, 4H, $H_{\mathrm{cc,dd}}$).
^{13}C NMR (126 MHz, DMSO-d_6, 298 K) δ = 174.36 (C_{26}), 174.24 (C_{27}), 170.89 (C_{c}), 170.77 (C_{t}), 169.95 (C_{22}), 165.41 (C_1), 158.01 (C_4), 150.16, 149.80 – 149.51 ($C_{\mathrm{i,p}}$), 148.59 (C_{f}), 146.76 (C_{11}), 141.99 (C_{29}), 135.58 (C_{19}), 134.58 (C_{18}), 131.33 (C_2), 130.08 (C_6), 128.07 (C_{30}), 127.35 – 126.94 ($C_{\mathrm{g,l,n}}$), 127.21 (C_{31}), 126.16 (C_{32}), 122.31 (C_{10}), 121.65 (C_7), 119.25 (C_5), 115.03 (C_3), 114.46, 112.52 – 112.02 ($C_{\mathrm{h,m,o}}$), 90.75 (C_{17}), 88.87 (C_{20}), 73.14 (C_{28}), 70.62 (C_{15}), 68.09 (C_{16}), 66.50 (C_8), 66.05 ($C_{\mathrm{e,u}}$), 65.20 (C_{ff}), 64.22 (C_{d}), 61.70 (C_{21}), 57.30 (C_{s}), 56.91 (C_{b}), 54.93, 54.89, 54.84, 54.82, 54.78, 54.71 (C_{j}), 48.81 (C_9), 47.44 (C_{25}), 46.76 (C_{24}), 30.70, 30.30, 30.26, 30.23, 30.19, 29.83, 29.67, 29.52, 29.44, 29.01 – 28.50, 28.23, 27.98, 27.86, 27.71 ($C_{\mathrm{a,k,r,v,x\text{-}z,aa\text{-}cc,ee},14}$), 25.56 ($C_{13}$), 24.70 ($C_{12}$), 23.83 ($C_{\mathrm{w,dd}}$), 20.48 ($C_{23}$).
HRMS-ESI(+): 2079.7410 $[M+Na]^+$, calculated for $C_{112}H_{130}Br_2N_4O_{23}Na^+$: 2079.7385.

4.5.4 Synthesis of S27*cis/endo*

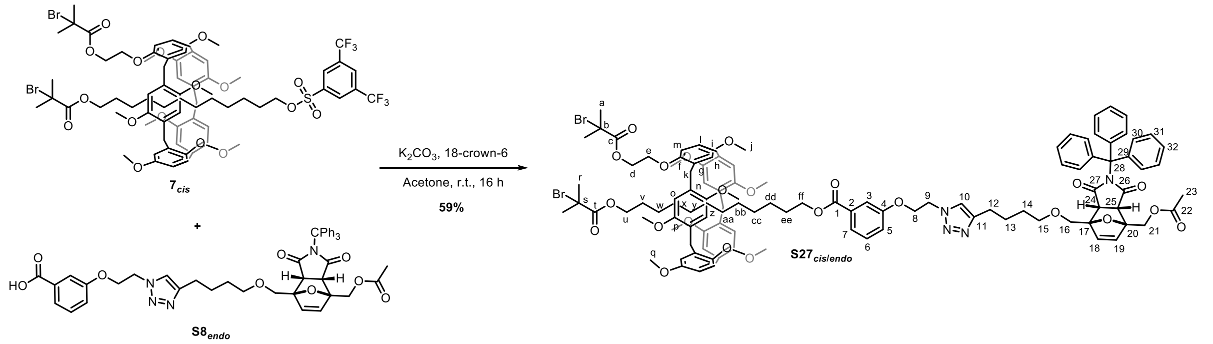

To a solution of **S8*endo*** (4.6 mg, 5.8 µmol, 1.0 eq.) in acetone (1 mL) was added K_2CO_3 (0.8 mg, 5.8 µmol, 1.0 eq.) and 18-crown-6 (1.5 mg, 5.8 µmol, 1.0 eq.). The mixture was stirred for 2 h at room temperature. **7*cis*** (9 mg, 5.8 µmol, 1.0 eq.) was added and the mixture stirred for a further 16 h at room temperature. The solution was filtered and the filtrate concentrated under vacuum. The residue was purified by preparative TLC (500 µm, DCM/MeOH, 30/1) to yield **S27*cis/endo*** as a white powder (7 mg, 3.4 µmol, 59% yield).

^{1}H NMR (500 MHz, DMSO-d_6, 298 K) δ = 7.91 (s, 1H, H_{10}), 7.58 (dt, *J* = 7.7, 1.2 Hz, 1H, H_7), 7.48 (t, *J* = 8.0 Hz, 1H, H_6), 7.43 (dd, *J* = 2.7, 1.5 Hz, 1H, H_3), 7.36 – 7.32 (m, 6H, H_{30}), 7.24 – 7.18 (m, 7H, $H_{5,31}$), 7.16 – 7.09 (m, 3H, H_{32}), 6.87 – 6.77 (m, 10H, $H_{\mathrm{h,m,o}}$), 5.33 – 5.28 (d, *J* = 5.7 Hz, d, *J* = 5.7 Hz, 2H, $H_{18,19}$), 4.73 (t, *J* = 5.1 Hz, 2H, H_9), 4.68 (ddd, *J* = 12.0, 9.1, 2.8 Hz, 1H, H_{d}), 4.50 (d, *J* = 12.8 Hz, 1H, H_{21}), 4.46 (t, *J* = 5.1 Hz, 2H, H_8), 4.40 (dt, *J* = 11.7, 3.0 Hz, 1H, H_{d}), 4.34 (d, *J* = 12.9 Hz, 1H, H_{21}), 4.17 (dt, *J* = 11.0, 3.2 Hz, 1H, H_{e}), 4.02 (ddd, *J* = 11.4, 9.2, 2.9 Hz, 1H, H_{e}), 3.86 – 3.76 (m, 4H, $H_{\mathrm{ff},16}$), 3.75 – 3.58 (m, 43H, $H_{\mathrm{j,k,q,u},24,25}$), 3.42 – 3.38 (m, 2H, H_{15}), 2.59 (t, *J* = 7.5 Hz, 2H, H_{12}), 1.97 (s, 3H, H_{23}), 1.93 – 1.91 (m, 12H, $H_{\mathrm{a,r}}$), 1.63 – 1.53 (m, 2H, H_{13}), 1.51 – 1.46 (m, 2H, H_{14}), 0.87 – 0.80 (m, 2H, H_{ee}), 0.79 – 0.70 (m, 2H, H_{v}), 0.44 – 0.32 (m, 4H, $H_{\mathrm{z,aa}}$), 0.23 – 0.15 (m, 2H, H_{bb}), 0.09 – 0.01 (m, 2H, H_{y}), -0.12 – -0.25 (m, 4H, $H_{\mathrm{cc,dd}}$), -0.42 – -0.50 (m, 2H, H_{w}), -0.50 – -0.57 (m, 2H, H_{x}).

^{13}C NMR (126 MHz, DMSO-d_6, 298 K) δ = 174.36 (C_{26}), 174.24 (C_{27}), 170.94, 170.83 ($C_{c,t}$), 169.95 (C_{22}), 165.38 (C_1), 158.02 (C_4), 150.18, 149.76 – 149.42 ($C_{i,p}$), 148.56 (C_f), 146.75 (C_{11}), 141.99 (C_{29}), 138.13 (C_{19}), 136.96 (C_{18}), 131.33 (C_2), 130.09 (C_6), 128.07 (C_{30}), 127.38 – 126.97 ($C_{g,l,n}$), 127.21 (C_{31}), 126.16 (C_{32}), 122.32 (C_{10}), 121.67 (C_7), 119.29 (C_5), 115.01 (C_3), 112.76 – 112.15 ($C_{h,m,o}$), 90.75 (C_{17}), 88.87 (C_{20}), 73.14 (C_{28}), 70.61 (C_{15}), 68.06 (C_{16}), 66.50 (C_8), 66.14 (C_e), 65.14 (C_{ff}), 64.29 (C_d), 61.70 (C_{21}), 57.25 (C_s), 56.96 (C_b), 54.94, 54.89, 54.87, 54.83, 54.80, 54.75, 54.72 (C_j), 48.80 (C_9), 47.44 (C_{25}), 46.75 (C_{24}), 30.40 – 30.18, 29.83, 29.63, 29.54, 29.42, 29.13 – 28.88, 28.50, 28.31, 27.96, 27.85, 27.65 ($C_{a,k,r,v,x\text{-}z,aa\text{-}cc,ee,14}$), 25.56 ($C_{13}$), 24.69 ($C_{12}$), 24.03 ($C_{dd}$), 23.62 ($C_w$), 20.47 ($C_{23}$).
HRMS-ESI(+): 2079.7370 [M+Na]$^+$, calculated for $C_{112}H_{130}Br_2N_4O_{23}Na^+$: 2079.7385.

4.6 Synthesis of 3-Cargo Rotaxanes

4.6.1 Synthesis of 8_3

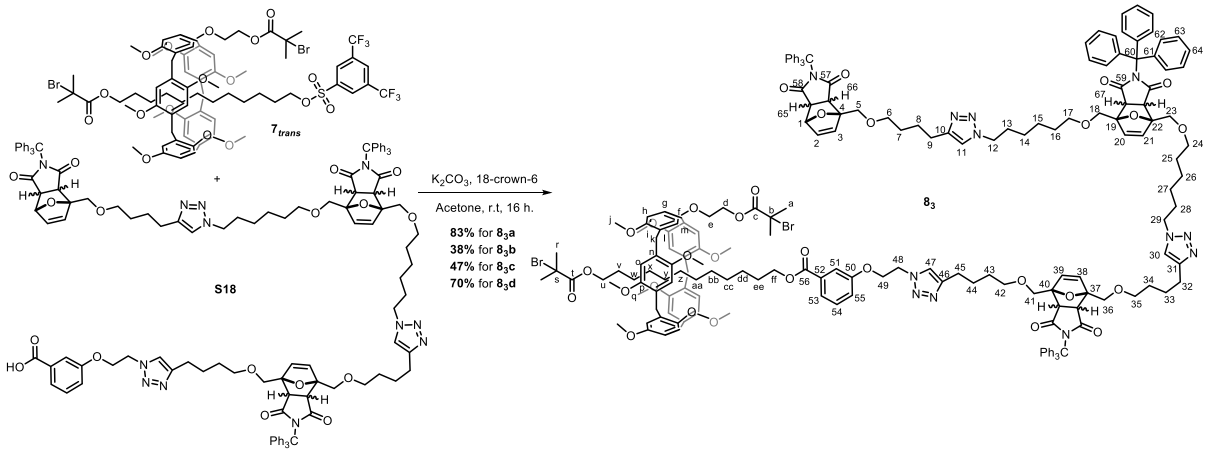

General Experimental Procedure:

To a solution of either **S18a, S18b, S18c** or **S18d** in acetone (1 mL) was added K_2CO_3 and 18-crown-6; the mixture was then stirred for 2 h at room temperature. **7_{trans}** was then added and the mixture stirred for a further 16 h at room temperature. The solution was filtered and the filtrate concentrated under vacuum. The residue was purified by preparative TLC (500 µm, DCM/MeOH, 25/1) to give the appropriate compound (either **8_3a**, **8_3b**, **8_3c** or **8_3d**) as a white powder.

8_3a, **8_3b**, **8_3c** and **8_3d** were isolated as mixtures of stereoisomers. When distinguishable, *exo* and *endo* adducts are denoted as X_x and X_x' respectively. Furan units are denoted as X_x''.

Compound **8_3a**:

Synthesis followed the general experimental procedure. **S18a** (6.0 mg, 2.9 µmol, 1.0 eq.), K_2CO_3 (0.4 mg, 2.9 µmol, 1.0 eq.), 18-crown-6 (0.8 mg, 2.9 µmol, 1.0 eq.) and **7_{trans}** (4.5 mg, 2.9 µmol, 1.0 eq.) were used in the reaction to yield **8_3a** (8 mg, 2.4 mmol, 83% yield).

The product is a mixture of stereoisomers including *endo-endo-exo* and *endo-endo-endo* with respect to the furan-maleimide Diels-Alder moieties; 5% of the furan moieties remain unfunctionalised with a maleimide cargo unit. Other minor stereoisomers are present in minute amount and can't be individually quantified.

^{1}H NMR (600 MHz, DMSO-d_6, 298 K) δ = 7.90 (s, 1H, H_{47}'), 7.79 – 7.75 (s, s, 2H, $H_{11,30}$), 7.57 (dt, J = 7.8, 1.2 Hz, 1H, H_{53}), 7.48 (t, J = 7.9 Hz, 1H, H_{54}), 7.43 (dd, J = 2.8, 1.5 Hz, 1H, H_{51}), 7.38 – 7.30 (m, 18H, H_{62}), 7.29 – 7.17 (m, 19H, $H_{55,63}$), 7.17 – 7.08 (m, 9H, H_{64}), 6.87 – 6.76 (m, 10H, $H_{h,m,o}$), 6.54 (dd, J = 5.6, 1.8

Hz, 0.88H, H_{2}), 6.46 (d, J = 5.6 Hz, 0.92H, $H_{3,20,21,38,39}$), 6.41 – 6.39 (m, 0.07H, H_{2}''), 6.38 (d, J = 3.0 Hz, 0.07H, H_{3}''), 6.33 – 6.30 (m, 0.17H, $H_{20,21,38,39}''$), 5.41 (d, J = 5.7 Hz, 0.13H, H_{2}'), 5.33 (d, J = 5.6 Hz, 0.14H, H_{3}'), 5.29 – 5.23 (m, 3.90H, $H_{20,21,38,39}'$), 5.20 (d, J = 1.8 Hz, 0.93H, H_{1}), 5.01 (dd, J = 5.7, 1.3 Hz, 0.15H, H_{1}'), 4.72 (t, J = 5.1 Hz, 2H, H_{48}), 4.61 (ddd, J = 11.9, 9.4, 2.9 Hz, 1H, H_{d}), 4.46 (t, J = 5.2 Hz, 2H, H_{49}), 4.33 – 4.27 (m, 1.41H, H_{d} and $H_{5,18,23,36,41}''$), 4.27 – 4.19 (m, 4H, $H_{12,29}$), 4.15 – 4.11 (m, 1H, H_{e}), 4.03 (d, J = 11.8 Hz, 0.91H, $H_{5,18,23,36,41}$), 4.02 – 3.95 (m, 1H, H_{e}), 3.81 – 3.58 (m, 54.08H, $H_{5,18,23,36,41}$, $H_{5,18,23,36,41,65}'$ and $H_{j,q,k,ff,u}$), 3.56 (d, J = 3.4 Hz, 4.87H, H_{67}'), 3.53 – 3.36 (m, 12.20H, $H_{6,17,24,35,42}$ and H_{66}', overlapped with water peak), 2.92 (d, J = 6.6 Hz, 0.87H, H_{65}), 2.80 (d, J = 6.6 Hz, 0.86H, H_{66}), 2.63 – 2.54 (m, 6H, $H_{9,32,45}$), 1.91 (s, 6H, H_{r}), 1.89 – 1.86 (s, s, 6H, H_{a}), 1.78 – 1.70 (m, 4H, $H_{13,28}$), 1.65 – 1.38 (m, 16H, $H_{7,8,33,44,34,43,16,25}$), 1.31 – 1.15 (m, 8H, $H_{14,27,15,26}$, overlapped with grease peak), 0.83 – 0.77 (m, 2H, H_{v}), 0.73 – 0.64 (m, 2H, H_{ee}), 0.45 – 0.33 (m, 4H, $H_{aa,z}$), 0.20 – 0.14 (m, 2H, H_{y}), 0.14 – 0.07 (m, 2H, H_{bb}), -0.21 – -0.30 (m, 2H, H_{w}), -0.30 – -0.36 (m, 2H, H_{x}), -0.36 – -0.43 (m, 4H, $H_{cc,dd}$).

^{13}C NMR (151 MHz, DMSO-d_6, 298 K) δ = 174.80 (C_{58}), 174.59, 174.57, 174.56, 174.50 (C_{59}'), 173.51(C_{57}), 170.88 (C_{c}), 170.77 (C_{t}), 165.41 (C_{56}), 158.01 (C_{50}), 150.16, 149.70, 149.67, 149.64, 149.60, 149.58, 149.56 ($C_{i,p}$), 148.59 (C_{f}), 146.76, 146.67, 146.61 ($C_{10,31,46}$), 142.67, 142.16, 142.12, 142.05 (C_{61}), 137.64 ($C_{3,20,21,38,39}$), 136.68 (C_{2}), 135.13 ($C_{20,21,38,39}'$), 131.33 (C_{52}), 130.08 (C_{54}), 128.28, 128.20, 128.08, 128.04 (C_{62}), 127.48, 127.41, 127.32, 127.29, 127.19, 127.11, 127.07, 127.06, 127.00 ($C_{63,g,l,n}$), 126.34, 126.30, 126.12 (C_{64}), 122.29 (C_{47}), 121.64 (C_{53}), 121.58, 121.54 ($C_{10,30}$), 119.24 (C_{55}), 115.04 (C_{51}), 114.43, 114.37, 112.82, 112.45, 112.41, 112.37, 112.32, 112.29, 112.23 ($C_{h,m,o}$), 110.37 (C_{2}''), 109.18 (C_{3}''), 90.81 (C_{4}), 90.70 ($C_{19,22,37,40}'$), 80.86 (C_{1}), 73.05 (C_{60}'), 72.72 (C_{60}), 70.81, 70.71, 70.62, 70.60 ($C_{6,17,24,35,42}$), 68.25, 68.20 ($C_{18,23,36,41}'$), 67.74 (C_{5}), 66.50 (C_{49}), 66.05 ($C_{e,u}$), 65.19 (C_{ff}), 64.21 (C_{d}), 57.30 (C_{s}), 56.91 (C_{b}), 54.93, 54.89, 54.84, 54.82, 54.76, 54.71 ($C_{j,q}$), 49.10 (C_{65}), 49.04 ($C_{12,29}$), 48.80 (C_{48}), 47.75 (C_{66}), 46.81, 46.75(C_{67}'), 30.30, 30.26, 30.23, 30.18 ($C_{a,r}$), 29.83, 29.66, 29.60, 29.51, 29.44 ($C_{13,28,aa,z,y,bb}$), 29.08 – 28.93, 28.90, 28.84, 28.73, 28.70 ($C_{16,25,34,k}$), 28.23, 27.98($C_{x,cc}$), 27.86 (C_{ee}), 27.70 (C_{v}), 25.74 – 25.52 ($C_{44,8,33,14,27,7,43}$), 24.93 ($C_{15,26}$), 24.78, 24.74, 24.71 ($C_{9,32,45}$), 23.81 ($C_{w,dd}$).

HRMS-ESI (+): 1665.7086 $[M+2H]^{2+}$, calculated for $C_{191}H_{214}Br_2N_{12}O_{31}H_2^{2+}$: 1665.7025.

Compound **8_3b**:

Synthesis followed the general experimental procedure. **S18b** (15 mg, 7.1 µmol, 1.0 eq.), K_2CO_3 (1.0 mg, 7.1 µmol, 1.0 eq.), 18-crown-6 (1.9 mg, 7.1 µmol, 1.0 eq.) and **7_{trans}** (11 mg, 7.1 µmol, 1.0 eq.) were used in the reaction to yield **8_3b** (9 mg, 2.7 mmol, 38% yield).

The product is a mixture of stereoisomers including *exo-endo-exo*, *endo-exo-exo*, *exo-endo-endo* and *endo-exo-endo* with respect to the furan-maleimide Diels-Alder moieties; 4% of the furan moieties remain unfunctionalised with a maleimide cargo unit. Other minor stereoisomers are present in minute amount and can't be individually quantified.

^{1}H NMR (500 MHz, DMSO-d_6, 298 K) δ = 7.91 (s, 0.46H, H_{47}), 7.90 (s, 0.47H, H_{47}'), 7.80 – 7.72 (m, 2H, $H_{11,30}$), 7.58 (d, J = 7.7 Hz, 1H, H_{53}), 7.51 – 7.45 (m, 1H, H_{54}), 7.45 – 7.42 (m, 1H, H_{51}), 7.37 – 7.28 (m, 18H, H_{62}), 7.28 – 7.17 (m, 19H, $H_{55,63}$), 7.17 – 7.07 (m, 9H, H_{64}), 6.87 – 6.77 (m, 10H, $H_{h,m,o}$), 6.54 (dd, J = 5.6, 1.8 Hz, 0.86H, H_{2}), 6.48 – 6.43 (m, 2.59H, $H_{3,20,21,38,39}$), 6.41 – 6.39 (m, 0.05H, H_{2}''), 6.38 (d, J = 2.8 Hz, 0.05H, H_{3}''), 6.33 – 6.30 (m, 0.14H, $H_{20,21,38,39}''$), 5.41 (d, J = 5.8 Hz, 0.15H, H_{2}'), 5.34 (d, J = 5.7 Hz, 0.18H, H_{3}'), 5.26 (s, 2.10H, $H_{20,21,38,39}'$), 5.20 (d, J = 1.7 Hz, 0.90H, H_{1}), 5.01 (d, J = 5.4 Hz, 0.16H, H_{1}'), 4.78 – 4.67 (m, 2H, H_{48}), 4.61 (ddd, J = 12.0, 9.1, 3.0 Hz, 1H, H_{d}), 4.51 – 4.39 (m, 2.8 Hz, 2H, H_{49}), 4.36 – 4.27 (m, 1.42H, H_{d} and $H_{5,18,23,36,41}''$), 4.27 – 4.19 (m, 4H, $H_{12,29}$), 4.13 (dt, J = 11.1, 3.3 Hz, 1H, H_{e}), 4.08 – 3.95 (m, 3.78H, $H_{5,18,23,36,41}$ and H_{e}), 3.86 – 3.58 (m, 51.04H, $H_{5,18,23,36,41}$, $H_{5,18,23,36,41,65}'$ and $H_{j,q,k,ff,u}$), 3.56 (d, J = 2.8 Hz, 3.88H, H_{67}'), 3.53 – 3.37 (m, 9.63H, $H_{6,17,24,35,42}$ and H_{66}', overlapped with water peak), 2.91 (d, J = 6.5 Hz, 0.81H, H_{65}), 2.88 – 2.83 (m, 1.72H, H_{67}), 2.81 – 2.77 (m, 0.81H, H_{66}), 2.65 – 2.53 (m, 6H, $H_{9,32,45}$), 1.91 (s, 6H, H_{r}), 1.89 – 1.87 (s, s, 6H, H_{a}), 1.76 – 1.70 (m, 4H, $H_{13,28}$), 1.66 – 1.37 (m, 16H, $H_{7,8,33,44,34,43,16,25}$), 1.34 – 1.15 (m, 8H, $H_{14,27,15,26}$, overlapped with grease peak), 0.84 – 0.80 (m, 2H, H_{v}),

0.75 – 0.64 (m, 2H, H_{ee}), 0.44 – 0.34 (m, 2H, $H_{aa,z}$), 0.21 – 0.13 (m, 2H, H_{y}), 0.13 – 0.07 (m, 2H, H_{bb}), -0.21 – -0.30 (m, 2H, H_{w}), -0.30 – -0.42 (m, 6H, $H_{cc,dd,x}$).
^{13}C NMR (126 MHz, DMSO-d_6, 298 K) δ = 174.78 (C_{58}), 174.58, 174.56 (C_{59}'), 173.50 (C_{57}), 173.17 (C_{59}), 170.88 (C_{c}), 170.76 (C_{t}), 165.40 (C_{56}), 158.01 (C_{50}), 150.15, 149.70, 149.67, 149.64, 149.59, 149.58, 149.56 ($C_{i,p}$), 148.59 (C_{f}), 146.81, 146.75, 146.66, 146.60 ($C_{10,31,46}$), 142.16, 142.04 (C_{61}), 137.72, 137.64 ($C_{3,20,21,38,39}$), 136.67 (C_{2}), 135.13 ($C_{20,21,38,39}'$), 131.33 (C_{52}), 130.06 (C_{54}), 128.28 – 127.87 (C_{62}), 127.57 – 126.97 ($C_{63,g,l,n}$), 126.40, 126.29, 126.1 (C_{64}), 122.28 (C_{47}), 121.63 (C_{53}), 121.56 ($C_{10,30}$), 119.24 (C_{55}), 115.03 (C_{51}), 14.43, 112.81, 112.45, 112.41, 112.36, 112.31, 112.29, 112.21 ($C_{h,m,o}$), 90.80, 90.75, 90.73, 90.71 ($C_{4,19,22,37,40}$), 90.69 ($C_{19,22,37,40}'$), 80.86 (C_{1}), 73.05 (C_{60}'), 72.72 (C_{60}), 70.95, 70.85, 70.80, 70.71, 70.61 ($C_{6,17,24,35,42}$), 68.32 – 68.12 ($C_{18,23,36,41}'$), 67.94 – 67.66 ($C_{5,18,23,36,41}$), 66.49 (C_{49}), 66.05 ($C_{e,u}$), 65.18 (C_{ff}), 64.20 (C_{d}), 57.29 (C_{s}), 56.89 (C_{b}), 54.92, 54.88, 54.84, 54.81, 54.75, 54.70 ($C_{j,q}$), 49.98 (C_{67}), 49.15 – 48.97 ($C_{65,12,29}$), 48.79 (C_{48}), 47.74 (C_{66}), 46.80, 46.75 (C_{67}'), 30.29, 30.26, 30.22, 30.18 ($C_{a,r}$), 29.83, 29.71 – 29.62, 29.60, 29.51, 29.43 ($C_{13,28,aa,z,y,bb}$), 29.08 – 28.62 ($C_{16,25,34,k}$), 28.23, 27.97 ($C_{x,cc}$), 27.86 (C_{ee}), 27.70 (C_{v}), 25.71 – 25.48 ($C_{44,8,33,14,27,7,43}$), 24.93 ($C_{15,26}$), 24.84 – 24.67 ($C_{9,32,45}$), 23.81 ($C_{w,dd}$).
HRMS-ESI (+): 1687.6820 $[M+2Na]^{2+}$, calculated for $C_{191}H_{214}Br_2N_{12}O_{31}Na_2^{2+}$: 1687.6845.

Compound **8_3c**:

Synthesis followed the general experimental procedure. **S18c** (9.3 mg, 4.5 µmol, 1.0 eq.), K_2CO_3 (0.6 mg, 4.5 µmol, 1.0 eq.), 18-crown-6 (1.2 mg, 4.5 µmol, 1.0 eq.) and **7_{trans}** (7.0 mg, 4.5 µmol, 1.0 eq.) were used in the reaction to yield **8_3c** (7 mg, 2.1 mmol, 47% yield).

The product is a mixture of stereoisomers including *exo-exo-exo* and *exo-exo-endo* with respect to the furan-maleimide Diels-Alder moieties; 4% of the furan moieties remain unfunctionalised with a maleimide cargo unit. Other minor stereoisomers are present in minute amount and can't be individually quantified.

^{1}H NMR (600 MHz, DMSO-d_6, 298 K) δ = 7.92 (s, 1H, H_{47}), 7.81 – 7.75 (m, 2H, $H_{11,30}$), 7.57 (d, J = 7.6 Hz, 1H, H_{53}), 7.48 (t, J = 8.0 Hz, 1H, H_{54}), 7.45 – 7.42 (m, 1H, H_{51}), 7.42 – 7.30 (m, 18H, H_{62}), 7.27 – 7.17 (m, 19H, $H_{55,63}$), 7.17 – 7.08 (m, 9H, H_{64}), 6.87 – 6.76 (m, 10H, $H_{h,m,o}$), 6.53 (d, J = 5.1 Hz, 0.83H, H_{2}), 6.49 – 6.42 (m, 4.44H, $H_{3,20,21,38,39}$), 6.41 – 6.39 (m, 0.06H, H_{2}''), 6.38 – 6.37 (m, 0.06H, H_{3}''), 6.32 – 6.30 (m, 0.16H, $H_{20,21,38,39}''$), 5.40 (d, J = 5.7 Hz, 0.15H, H_{2}'), 5.33 (d, J = 5.4 Hz, 0.33H, H_{3}'), 5.25 (s, 0.26H, $H_{20,21,38,39}''$), 5.20 (s, 0.85H, H_{1}), 5.01 (d, J = 5.6 Hz, 0.15H, H_{1}'), 4.73 (t, J = 5.1 Hz, 2H, H_{48}), 4.64 – 4.58 (m, 1H, H_{d}), 4.46 (t, J = 5.0 Hz, 2H, H_{49}), 4.35 – 4.28 (m, 1.41H, H_{d} and $H_{5,18,23,36,41}''$), 4.28 – 4.18 (m, 4H, $H_{12,29}$), 4.16 – 4.08 (m, 1H, H_{e}), 4.07 – 4.01 (m, 4.56H, $H_{5,18,23,36,41}$), 4.01 – 3.95 (m, 1H, H_{e}), 3.80 – 3.58 (m, 48.67H, $H_{5,18,23,36,41}$, $H_{5,18,23,36,41,65}'$ and $H_{j,q,k,ff,u}$), 3.58 – 3.39 (m, 12.91H, $H_{6,17,24,35,42}$ and H_{66}', overlapped with water peak), 2.91 (d, J = 6.5 Hz, 0.78H, H_{65}), 2.88 – 2.83 (m, 3.51H, H_{67}), 2.79 (dd, J = 6.5, 2.0 Hz, 0.78H, H_{66}), 2.64 – 2.55 (m, 6H, $H_{9,32,45}$), 1.91 (s, 6H, H_{r}), 1.89 – 1.86 (s, s, 6H, H_{a}), 1.79 – 1.72 (m, 4H, $H_{13,28}$), 1.66 – 1.42 (m, 16H, $H_{7,8,33,44,34,43,16,25}$), 1.36 – 1.17 (m, 8H, $H_{14,27,15,26}$, overlapped with grease peak), 0.83 – 0.78 (m, 2H, H_{v}), 0.73 – 0.65 (m, 2H, H_{ee}), 0.45 – 0.35 (m, 4H, $H_{aa,z}$), 0.19 – 0.14 (m, 2H, H_{y}), 0.14 – 0.08 (m, 2H, H_{bb}), -0.23 – -0.31 (m, 2H, H_{w}), -0.32 – -0.45 (m, 6H, $H_{cc,dd,x}$).
^{13}C NMR (151 MHz, DMSO-d_6, 298 K) δ = 174.81 (C_{58}), 173.53 (C_{57}), 173.19 (C_{59}), 170.90 (C_{c}), 170.78 (C_{t}), 165.42 (C_{56}), 158.02 (C_{50}), 150.15, 149.72 – 149.51 ($C_{i,p}$), 148.59 (C_{f}), 146.83, 146.68 ($C_{10,31,46}$), 142.18, 142.13, 142.05 (C_{61}), 137.74, 137.66 ($C_{3,20,21,38,39}$), 136.70 (C_{2}), 131.33 (C_{52}), 130.10 (C_{54}), 128.28 – 127.88 (C_{62}), 127.59 – 126.94 ($C_{63,g,l,n}$), 126.43, 126.32, 126.13 (C_{64}), 122.33 (C_{47}), 121.65 (C_{53}), 121.60, 121.59 (m $C_{10,30}$), 119.26 (C_{55}), 115.02 (C_{51}), 114.40, 112.81, 112.44, 112.40, 112.35, 112.30, 112.21 ($C_{h,m,o}$), 90.82, 90.78 – 90.70 ($C_{4,19,22,37,40}$), 80.87 (C_{1}), 72.72 (C_{60}), 70.97, 70.86, 70.81 ($C_{6,17,24,35,42}$), 67.90, 67.84, 67.76 ($C_{5,18,23,36,41}$), 66.50 (C_{49}), 66.06 ($C_{e,u}$), 65.21 (C_{ff}), 64.21 (C_{d}), 57.31 (C_{s}), 56.92 (C_{b}), 54.93, 54.89, 54.84, 54.82, 54.76, 54.71 ($C_{j,q}$), 50.00 (C_{67}), 49.18 – 49.00 ($C_{65,12,29}$), 48.81 (C_{48}), 47.76 (C_{66}), 30.30, 30.27, 30.23, 30.19 ($C_{a,r}$), 29.86, 29.75 – 29.66, 29.62, 29.54, 29.46 ($C_{13,28,aa,z,y,bb}$), 29.17 – 28.69 ($C_{16,25,34,k}$), 28.25, 27.99 ($C_{x,cc}$), 27.88 (C_{ee}), 27.71 (C_{v}), 25.75 – 25.56 ($C_{44,8,33,14,27,7,43}$), 24.96 ($C_{15,26}$), 24.80

($C_{9,32,45}$), 23.82 ($C_{w,dd}$).
HRMS-ESI (+): 1687.6860 [M+2Na]$^{2+}$, calculated for $C_{191}H_{214}Br_2N_{12}O_{31}Na_2{}^{2+}$: 1687.6845.

Compound **8₃d**:

Synthesis followed the general experimental procedure. **S18d** (19 mg, 9.0 µmol, 1.0 eq.), K_2CO_3 (1.2 mg, 9.0 µmol, 1.0 eq.), 18-crown-6 (2.4 mg, 9.0 µmol, 1.0 eq.) and **7*trans*** (14 mg, 9.0 µmol, 1.0 eq.) were used in the reaction to yield **8₃d** (21 mg, 6.3 mmol, 70% yield).

The product is a mixture of stereoisomers including *endo-endo-exo*, *endo-endo-endo*, *exo-endo-exo*, *endo-exo-exo*, *exo-endo-endo*, *endo-exo-endo*, *exo-exo-exo*, and *exo-exo-endo* with respect to the furan-maleimide Diels-Alder moieties; 7% of the furan moieties remain unfunctionalised with a maleimide cargo unit. Other minor stereoisomers are present in minute amount and can't be individually quantified.

^{1}H NMR (500 MHz, DMSO-d$_6$, 298 K) δ = 7.92 (s, 0.44H, H_{47}), 7.91 (s, 0.50H, H_{47}'), 7.81 – 7.73 (m, 2H, $H_{11,30}$), 7.58 (d, *J* = 7.7 Hz, 1H, H_{53}), 7.48 (t, *J* = 8.1 Hz, 1H, H_{54}), 7.45 – 7.42 (m, 1H, H_{51}), 7.41 – 7.29 (m, 18H, H_{62}), 7.28 – 7.17 (m, 19H, $H_{55,63}$), 7.17 – 7.08 (m, 9H, H_{64}), 6.89 – 6.74 (m, 10H, $H_{h,m,o}$), 6.53 (d, *J* = 5.6 Hz, 0.80H, H_2), 6.49 – 6.42 (m, 2.61H, $H_{3,20,21,38,39}$), 6.40 (s, 0.09H, H_2''), 6.38 (s, 0.09H, H_3''), 6.32 (s, 0.25H, $H_{20,21,38,39}''$), 5.40 (d, *J* = 5.7 Hz, 0.17H, H_2'), 5.33 (d, *J* = 5.7 Hz, 0.24H, H_3'), 5.25 (s, 1.98H, $H_{20,21,38,39}'$), 5.20 (s, 0.84H, H_1), 5.01 (d, *J* = 5.5 Hz, 0.17H, H_1'), 4.78 – 4.67 (m, 2H, H_{48}), 4.62 (t, *J* = 9.9 Hz, 1H, H_d), 4.53 – 4.40 (m, 2H, H_{49}), 4.36 – 4.27 (m, 1.66H, H_d and $H_{5,18,23,36,41}''$), 4.27 – 4.18 (m, 4H, $H_{12,29}$), 4.16 – 4.07 (m, 1H, H_e), 4.07 – 4.01 (m, 2.59H, $H_{5,18,23,36,41}$), 4.01 – 3.95 (m, 1H, H_e), 3.87 – 3.58 (m, 48.79H, $H_{5,18,23,36,41}$, $H_{5,18,23,36,41,65}'$ and $H_{j,q,k,ff,u}$), 3.56 (d, *J* = 2.8 Hz, 3.77H, H_{67}'), 3.53 – 3.36 (m, 9.30H, $H_{6,17,24,35,42}$ and H_{66}', overlapped with water peak), 2.91 (d, *J* = 6.4 Hz, 0.77H, H_{65}), 2.88 – 2.83 (m, 1.76H, H_{67}), 2.81 – 2.76 (m, 0.77H, H_{66}), 2.66 – 2.52 (m, 6H, $H_{9,32,45}$), 1.91 (s, 6H, H_r), 1.90 – 1.85 (s, s, 6H, H_a), 1.80 – 1.69 (m, 4H, $H_{13,28}$), 1.68 – 1.36 (m, 16H, $H_{7,8,33,44,34,43,16,25}$), 1.36 – 1.20 (m, 8H, $H_{14,27,15,26}$), 0.79 – 0.73 (m, 2H, H_v), 0.73 – 0.64 (m, 2H, H_{ee}), 0.46 – 0.34 (m, 4H, $H_{aa,z}$), 0.21 – 0.14 (m, 2H, H_y), 0.14 – 0.07 (m, 2H, H_{bb}), -0.22 – -0.32 (m, 2H, H_w), -0.32 – -0.43 (m, 6H, $H_{cc,dd,x}$).
^{13}C NMR (126 MHz, DMSO-*d*$_6$, 298 K) δ = 174.82 (C_{58}), 174.68 – 174.57 (m, C_{59}'), 173.54 (C_{57}), 173.20 (C_{59}), 170.91 (C_c), 170.79 (C_t), 165.43 (C_{56}), 158.03 (C_{50}), 151.93 ($C_{4,19,22,37,40}''$), 150.16, 149.71, 149.68, 149.65, 149.60, 149.59, 149.56 (m, $C_{i,p}$), 148.60 (C_f), 146.84, 146.78, 146.69, 146.64 (m, $C_{10,31,46}$), 142.19, 142.14, 142.07 (m, C_{61}), 137.75, 137.66 (m, $C_{3,20,21,38,39}$), 136.71 (C_2), 135.15 ($C_{20,21,38,39}'$), 131.34 (C_{52}), 130.11 (C_{54}), 128.21 – 127.93 (m, C_{62}), 127.45, 127.32, 127.29, 127.22, 127.16, 127.11, 127.06, 127.00 (m, $C_{63,g,l,n}$), 126.44, 126.33, 126.14 (m, C_{64}), 122.33 (C_{47}), 121.66 (C_{53}), 121.63 – 121.51 (m, $C_{10,30}$), 119.25 (C_{55}), 115.04 (C_{51}), 114.41, 112.81, 112.51 – 112.14 (m, $C_{h,m,o}$), 110.40 (C_2''), 109.84 ($C_{20,21,38,39}''$), 109.23 (C_3''), 90.90, 90.83, 90.79 – 90.73 (m, $C_{4,19,22,37,40}$), 90.71 ($C_{19,22,37,40}'$), 80.89 (C_1), 73.06 (C_{60}'), 72.73 (C_{60}), 70.98, 70.86, 70.83, 70.73, 70.64, 70.62 (m, $C_{6,17,24,35,42}$), 68.34 – 68.12 (m, $C_{18,23,36,41}'$), 67.99 – 67.69 (m, $C_{5,18,23,36,41}$), 66.51 (C_{49}), 66.07 ($C_{e,u}$), 65.22 (C_{ff}), 64.22 (C_d), 57.31 (C_s), 56.92 (C_b), 54.93, 54.89, 54.85, 54.82, 54.76, 54.71 (m, $C_{j,q}$), 50.01 (C_{67}), 49.17 – 49.00 (m, $C_{65,12,29}$), 48.82 (C_{48}), 47.78 (C_{66}), 46.82, 46.77 (m, C_{67}'), 30.31, 30.27, 30.24, 30.19 (m, $C_{a,r}$), 29.88, 29.79 – 29.65, 29.63, 29.56, 29.48 (m, $C_{13,28,aa,z,y,bb}$), 29.12 – 28.99, 28.94, 28.88, 28.85 – 28.70 (m, $C_{16,25,34,k}$), 28.27, 28.00 ($C_{x,cc}$), 27.89 (C_{ee}), 27.73 (C_v), 25.79 – 25.55 (m, $C_{44,8,33,14,27,7,43}$), 24.96 ($C_{15,26}$), 24.85 – 24.72 (m, $C_{9,32,45}$), 23.83 ($C_{w,dd}$).
HRMS-ESI (+): 1687.6807 [M+2Na]$^{2+}$, calculated for $C_{191}H_{214}Br_2N_{12}O_{31}Na_2{}^{2+}$: 1687.6845.

4.7 Synthesis of 5-Cargo Rotaxane

4.7.1 Synthesis of 8_5

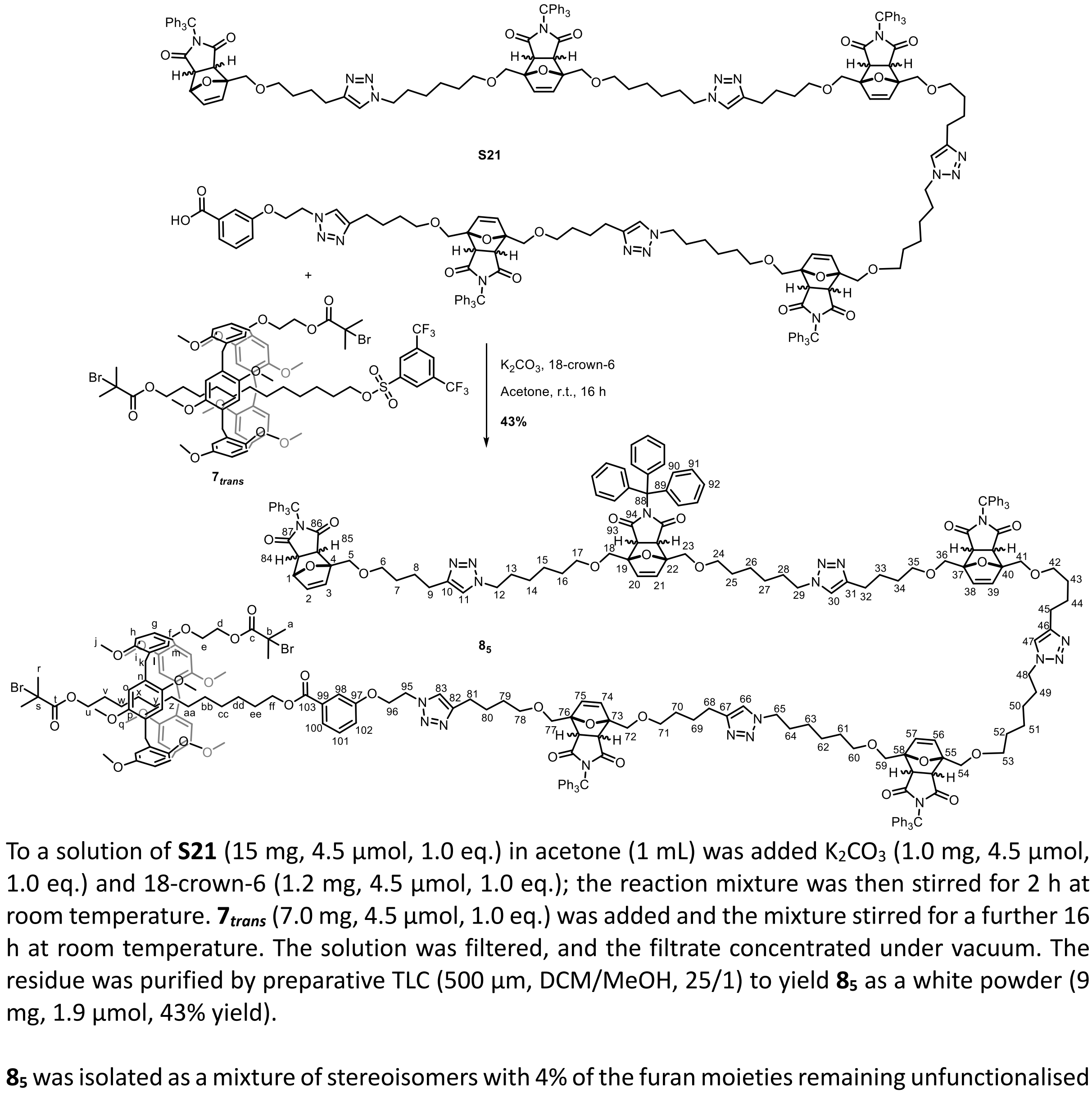

To a solution of **S21** (15 mg, 4.5 µmol, 1.0 eq.) in acetone (1 mL) was added K_2CO_3 (1.0 mg, 4.5 µmol, 1.0 eq.) and 18-crown-6 (1.2 mg, 4.5 µmol, 1.0 eq.); the reaction mixture was then stirred for 2 h at room temperature. **7**$_{\textbf{\textit{trans}}}$ (7.0 mg, 4.5 µmol, 1.0 eq.) was added and the mixture stirred for a further 16 h at room temperature. The solution was filtered, and the filtrate concentrated under vacuum. The residue was purified by preparative TLC (500 µm, DCM/MeOH, 25/1) to yield **8**$_{\mathbf{5}}$ as a white powder (9 mg, 1.9 µmol, 43% yield).

8$_{\mathbf{5}}$ was isolated as a mixture of stereoisomers with 4% of the furan moieties remaining unfunctionalised with a maleimide cargo unit.

When distinguishable, *exo* and *endo* adducts are denoted as X_x and X_x' respectively. Furan units are denoted as X_x''.

^{1}H NMR (500 MHz, DMSO-d_6, 298 K) δ = 7.91 (s, 0.38H, H_{83}), 7.90 (s, 0.53H, H_{83}'), 7.80 – 7.73 (m, 4H, $H_{11,30,47,66}$), 7.57 (d, *J* = 7.6 Hz, 1H, H_{100}), 7.50 – 7.45 (m, 1H, H_{101}), 7.45 – 7.42 (m, 1H, H_{98}), 7.42 – 7.29 (m, 30H, H_{90}), 7.28 – 7.16 (m, 31H, $H_{91,102}$), 7.16 – 7.06 (m, 15H, H_{92}), 6.87 – 6.74 (m, 10H, $H_{h,m,o}$), 6.53 (d, *J* = 5.7 Hz, 0.83H, H_2), 6.48 – 6.42 (m, 4.27H, $H_{3,20,21,38,39,56,57,74,75}$), 6.41 – 6.39 (m, 0.05H, H_2''), 6.39 – 6.37 (m, 0.06H, H_3''), 6.33 – 6.30 (m, 0.33H, $H_{20,21,38,39,56,57,74,75}''$), 5.41 (d, *J* = 5.2 Hz, 0.17H, H_2'), 5.34 (d, *J* = 5.4 Hz, 0.22H, H_3'), 5.26 (s, 4.91H, $H_{20,21,38,39,56,57,74,75}'$), 5.20 (s, 0.93H, H_1), 5.01 (d, *J* = 5.6 Hz, 0.17H, H_1'), 4.75 – 4.67 (m, 2H, H_{95}), 4.61 (ddd, *J* = 12.0, 8.6, 2.7 Hz, 1H, H_d), 4.49 – 4.39 (m, 2H, H_{96}), 4.36 – 4.28 (m, 1.92H, H_d and $H_{5,18,23,36,41,54,59,72,77}''$), 4.27 – 4.18 (m, 8H, $H_{12,29,48,65}$), 4.16 – 4.08 (m, 1H, H_e), 4.07 – 3.96 (m, 5.25H, $H_{5,18,23,36,41,72,77}$ and H_e), 3.88 – 3.58 (m, 58.04H, $H_{5,18,23,36,41,54,59,72,77}$,

$H_{5,18,23,36,41,54,59,72,77,84}'$ and $H_{j,q,k,ff,u}$), 3.56 (s, 7.77H, H_{67}'), 3.53 – 3.36 (m, 15.84H, $H_{6,17,24,35,42,53,60,71,78}$ and H_{85}', overlapped with water peak), 2.91 (d, J = 6.5 Hz, 0.79H, H_{84}), 2.88 – 2.82 (m, 3.34H, H_{93}), 2.79 (d, J = 6.6 Hz, 0.80H, H_{85}), 2.68 – 2.53 (m, 10H, $H_{9,32,45,68,81}$), 1.91 (s, 6H, H_r), 1.89 – 1.86 (s, s, 6H, H_a), 1.76 – 1.69 (m, 8H, $H_{13,28,49,64}$), 1.66 – 1.37 (m, 28H, $H_{8,33,44,69,80,7,34,43,70,79,16,25,52,61}$), 1.34 – 1.15 (m, 16H, $H_{14,27,15,26,51,62,50,63}$, overlapped with grease peak), 0.83 – 0.78 (m, 2H, H_v), 0.73 – 0.62 (m, 2H, H_{ee}), 0.47 – 0.33 (m, 4H, $H_{aa,z}$), 0.22 – 0.14 (m, 2H, H_y), 0.14 – 0.07 (m, 2H, H_{bb}), -0.21 – -0.30 (m, 2H, H_w), -0.30 – -0.48 (m, 6H, $H_{cc,dd,x}$).
^{13}C NMR (126 MHz, DMSO-d_6, 298 K) δ = 174.78 (C_{87}), 174.57 (C_{94}'), 173.49 (C_{86}), 173.16 (C_{94}), 170.87 (C_c), 170.75 (C_t), 165.40 (C_{103}), 158.00 (C_{97}), 150.15, 149.70, 149.66, 149.64, 149.59, 149.58, 149.55 ($C_{i,p}$), 148.59 (C_f), 146.81, 146.75, 146.66, 146.60 ($C_{10,31,46,67,82}$), 142.15, 142.11, 142.03 (C_{89}), 137.71, 137.63 ($C_{3,20,21,38,39,56,57,74,75}$), 136.67 ($C_2$), 135.13 ($C_{20,21,38,39,56,57,74,75}'$), 131.33 ($C_{99}$), 130.06 ($C_{101}$), 128.20, 128.07, 128.03, 128.00 (C_{90}), 127.61 – 126.92 ($C_{91,g,l,n}$), 126.40, 126.29, 126.10 (C_{92}), 122.28 (C_{83}), 121.63, 121.55 ($C_{100,11,30,47,66}$), 119.23 (C_{102}), 115.01 (C_{98}), 114.42, 112.49 – 112.17 ($C_{h,m,o}$), 90.80, 90.75, 90.69 ($C_{4,19,22,37,40,55,58,73,76}$), 80.86 ($C_1$), 73.05 ($C_{88}'$), 72.72 ($C_{88}$), 70.95, 70.85, 70.80, 70.71, 70.62($C_{6,17,24,35,42,53,60,71,78}$), 68.26, 68.20 ($C_{18,23,36,41,54,59,72,77}'$), 67.88, 67.81, 67.74 ($C_{5,18,23,36,41,54,59,72,77}$), 66.49 ($C_{96}$), 66.07, 66.04 ($C_{e,u}$), 65.18 ($C_{ff}$), 64.20 ($C_d$), 57.28 ($C_s$), 56.89 ($C_b$), 54.92, 54.88, 54.83, 54.81, 54.75, 54.70 ($C_{j,q}$), 49.98 (C_{93}), 49.19 – 48.94 ($C_{84,12,29,48,65}$), 48.79 (C_{95}), 47.74 (C_{85}), 46.81, 46.76 (C_{93}), 30.29, 30.25, 30.22, 30.18 ($C_{a,r}$), 29.82, 29.68, 29.65, 29.60, 29.51, 29.43 ($C_{13,28,49,64,aa,z,y,bb}$), 29.07 – 28.62 ($C_{16,25,52,61,34,43,70,k}$), 28.22, 27.97 ($C_{x,cc}$), 27.86 ($C_{ee}$), 27.69 ($C_v$), 25.66, 25.60($C_{7,79,14,27,50,63,8,33,44,69,80}$), 24.93 ($C_{15,26,51,62}$), 24.78, 24.73 ($C_{9,32,45,68,81}$), 23.81.
HRMS-ESI (+): 2338.0224 $[M+2H]^{2+}$, calculated for $C_{273}H_{302}Br_2N_{20}O_{41}H_2^{2+}$: 2338.0337.

4.8 Synthesis of Alternative-Cargo Compounds – Drug-Containing Cargo Release

4.8.1 Synthetic Route to Rotaxane With Linker-Containing Cargo

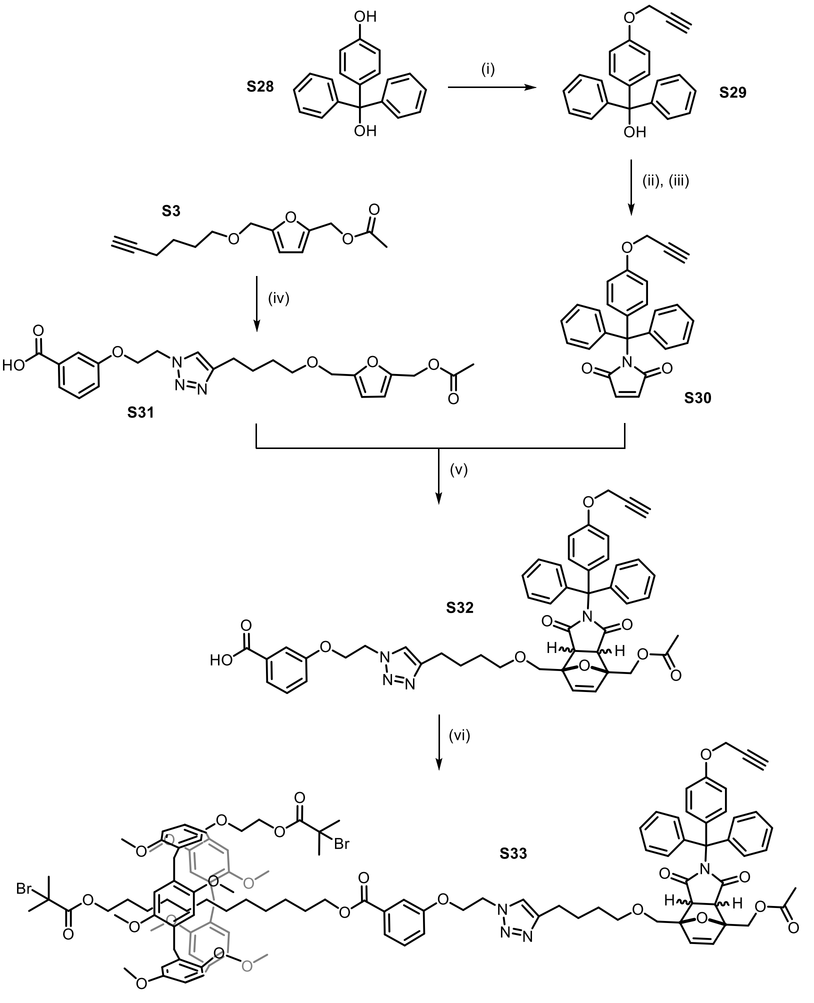

Figure S12. Synthetic route to **S33**. Conditions: (i) Propargyl bromide, K_2CO_3, 18-crown-6, acetone, 55 °C, 16 h, 59% yield; (ii) Acetyl chloride, r.t., 16 h; (iii) Maleimide, Et_3N, DCM, DCM, r.t., 1 h, 50% yield; (iv) **S7**, CuBr, PMDETA, DCM, r.t., 16 h, 91% yield; (v) DCM/MeCN, 60 °C, 48 h, 94% yield; (vi) **7*trans***, K_2CO_3, 18-crown-6, acetone, r.t., 16 h, 43% yield.

4.8.2 Synthesis of S29

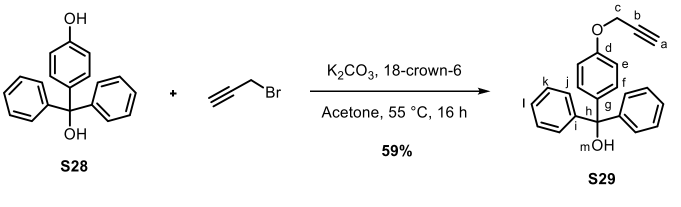

To a solution of **S28** (150 mg, 543 µmol, 1.0 eq.) in acetone (3 mL) was added K_2CO_3 (112 mg, 815 µmol, 1.5 eq.) and 18-crown-6 (14 mg, 54 µmol, 0.1 eq.). The mixture was stirred for 2 h at room temperature. Propargyl bromide (80 % in toluene, 90 µL, 815 µmol, 1.5 eq.) was added and the mixture stirred for a further 16 h at 55 °C. The solution was filtered, and the filtrate concentrated under vacuum. The

residue was purified by preparative TLC (2000 µm, PE/Acetone, 3/1, eluted twice) to yield **S29** as a yellow powder (100 mg, 318 µmol, 59% yield).

^{1}H NMR (400 MHz, Acetone-d_6, 298 K) δ = 7.34 – 7.23 (m, 10H, $H_{j,k,l}$), 7.23 – 7.19 (m, 2H, H_f), 6.96 – 6.90 (m, 2H, H_e), 5.29 (s, 1H, H_m), 4.78 (d, J = 2.4 Hz, 2H, H_c), 3.09 (t, J = 2.4 Hz, 1H, H_a).
^{13}C NMR (101 MHz, Acetone-d_6, 298 K) δ = 157.43 (C_d), 148.92, 148.89 (C_i), 141.70, 141.66 (C_g), 130.08 (C_f), 128.78 (C_k), 128.35 (C_j), 127.54 (C_l), 114.54 (C_e), 81.70, 81.60 (C_h), 79.79 (C_b), 76.96 (C_a), 56.14 (C_c).
HRMS-ESI (+): 337.1199 [M+Na]$^+$, calculated for $C_{22}H_{18}O_2Na^+$: 337.1199.

4.8.3 Synthesis of S30

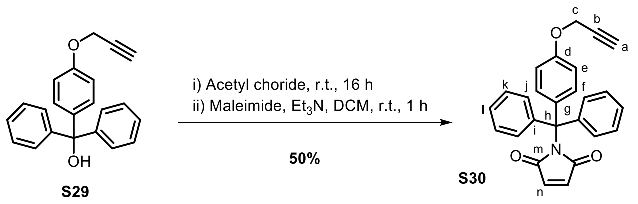

S29 (80 mg, 255 µmol, 1.0 eq.) was dissolved in acetyl chloride (0.5 mL) and the mixture stirred for 16 h at room temperature. The solvent was evaporated and the residue dried under vacuum for 1 h before being added to a solution of maleimide (37 mg, 382 µmol, 1.5 eq.) in DCM (1.0 mL). Et_3N (71 µL, 510 µmol, 2.0 eq.) was added and the mixture stirred for 1 h at room temperature. The reaction mixture was concentrated under vacuum and the residue purified by preparative TLC (2000 µm, PE/Et_2O, 2/1, eluted twice) to yield **S30** as a white powder (50 mg, 127 µmol, 50% yield).

^{1}H NMR (400 MHz, Acetone-d_6, 298 K) δ = 7.47 – 7.42 (m, 4H, H_j), 7.42 – 7.38 (m, 2H, H_f), 7.28 – 7.21 (m, 4H, H_k), 7.18 – 7.12 (m, 2H, H_l), 6.92 – 6.86 (m, 2H, H_e), 6.77 (s, 2H, H_n), 4.76 (d, J = 2.4 Hz, 2H, H_c), 3.09 (t, J = 2.4 Hz, 1H, H_a).
^{13}C NMR (101 MHz, Acetone-d_6, 298 K) δ = 171.50 (C_m), 157.11 (C_d), 144.28 (C_i), 136.48 (C_g), 135.71 (C_n), 130.96 (C_f), 129.19 (C_j), 128.23 (C_k), 127.04 (C_l), 114.30 (C_e), 79.75 (C_b), 77.01 (C_a), 73.25 (C_h), 56.10 (C_c).
HRMS-ESI (+): 416.1251 [M+Na]$^+$, calculated for $C_{26}H_{19}NO_3Na^+$: 416.1257.

4.8.4 Synthesis of S31

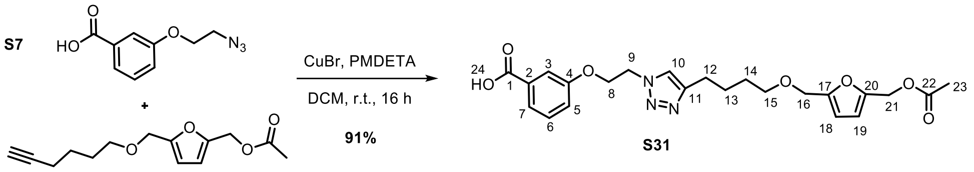

Three sealed 5 mL microwave vials (*A*, *B*, and *C*) were prepared as follows: *A* contained PMDETA (17 mg, 97 µmol, 0.5 eq.) in DCM (3 mL), *B* contained CuBr (17 mg, 116 µmol, 0.6 eq.), and *C* contained **S3** (48 mg, 193 µmol, 1.0 eq.) and **S7** (40 mg, 193 µmol, 1.0 eq.). Vials *B* and *C* were subjected to three N_2/vacuum cycles before use. The solution in vial *A* was degassed by bubbling with N_2 until ~1 mL DCM was left before being transferred to vial *B via* cannula; the resulting mixture was stirred until all the CuBr had dissolved. The CuBr/PMDETA solution in vial *B* was then transferred to vial *C via* cannula; the resulting reaction mixture was stirred for 16 h at room temperature. The reaction mixture was washed with aqueous EDTA solution (0.25 M, pH 7, 2 x 1 mL) and brine (2 x 1 mL). The organic layer was collected and dried over magnesium sulfate. The mixture was filtered before being concentrated under vacuum. The residue was purified by preparative TLC (500 µm, DCM/MeOH, 20/1, eluted twice) to yield **S31** as a white powder (80 mg, 175 µmol, 91% yield).

^{1}H NMR (500 MHz, Acetone-d_6, 298 K) δ = 7.83 (s, 1H, H_{10}), 7.64 (dt, J = 7.6, 1.3 Hz, 1H, H_7), 7.56 (dd, J

= 2.7, 1.5 Hz, 1H, *H*$_3$), 7.41 (t, *J* = 8.0 Hz, 1H, *H*$_6$), 7.19 (ddd, *J* = 8.2, 2.6, 1.0 Hz, 1H, *H*$_5$), 6.41 (d, *J* = 3.1 Hz, 1H, *H*$_{19}$), 6.33 (d, *J* = 3.2 Hz, 1H, *H*$_{18}$), 5.00 (s, 2H, *H*$_{21}$), 4.81 (t, *J* = 5.2 Hz, 2H, *H*$_9$), 4.52 (t, *J* = 5.2 Hz, 2H, *H*$_8$), 4.38 (s, 2H, *H*$_{16}$), 3.47 (t, *J* = 6.3 Hz, 2H, *H*$_{15}$), 2.68 (t, *J* = 7.5 Hz, 2H, *H*$_{12}$), 1.99 (s, 3H, *H*$_{23}$), 1.76 – 1.64 (m, 2H, *H*$_{13}$), 1.63 – 1.55 (m, 2H, *H*$_{14}$).
^{13}C NMR (126 MHz, Acetone-*d*$_6$, 298 K) δ = 170.61 (C$_{22}$), 167.32 (C$_1$), 159.29 (C$_4$), 154.11 (C$_{17}$), 150.72 (C$_{20}$), 148.19 (C$_{11}$), 132.95 (C$_2$), 130.54 (C$_6$), 123.30 (C$_7$), 122.87 (C$_{10}$), 120.23 (C$_5$), 116.11 (C$_3$), 111.97 (C$_{19}$), 110.60 (C$_{18}$), 70.39 (C$_{15}$), 67.69 (C$_8$), 65.13 (C$_{16}$), 58.46 (C$_{21}$), 50.01 (C$_9$), 26.86 (C$_{13}$), 25.87 (C$_{12}$), 20.67 (C$_{23}$).
HRMS-ESI (+): 408.1744 [M+Na]$^+$, calculated for $C_{23}H_{27}N_3O_7Na^+$: 408.1741.

4.8.5 Synthesis of S32

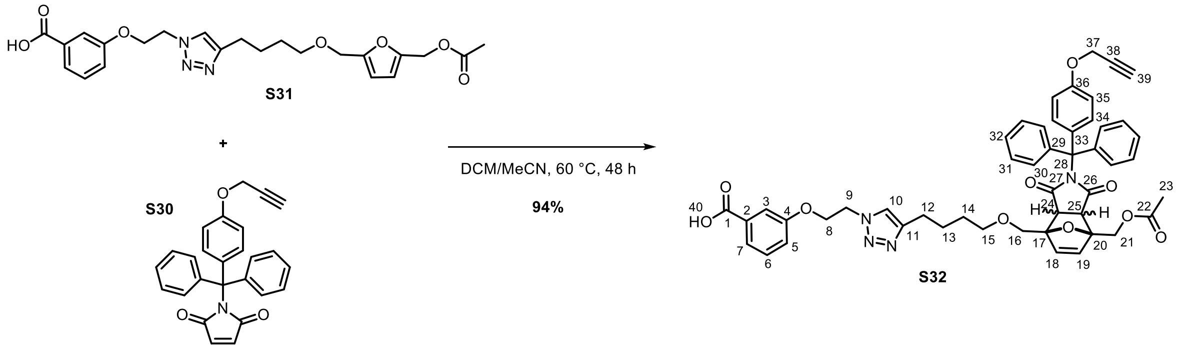

A solution of **S31** (20 mg, 44 µmol, 1.0 eq.) and **S30** (275 mg, 700 µmol, 16.0 eq.) in DCM/MeCN (2:1, 1 mL) was stirred for 48 h at 60 °C. After the mixture was cooled down, it was concentrated under vacuum. The crude material was purified by preparative TLC (2000 µm, DCM/MeOH, 20/1, eluted twice) to yield **S32** (35 mg, 41 µmol, 94% yield) as a pale-yellow powder.

The product is a mixture of stereoisomers including *exo* and *endo* with respect to the furan-maleimide Diels-Alder moiety. When distinguishable, *exo* and *endo* adducts are denoted as X$_x$ and X$_x$’ respectively.

^{1}H NMR (400 MHz, Acetone-*d*$_6$, 298 K) δ = 7.81 (s, 1H, *H*$_{10}$), 7.81 (s, 1H, *H*$_{10'}$), 7.67 – 7.62 (m, 1H, *H*$_7$), 7.56 (dd, *J* = 2.7, 1.5 Hz, 1H, *H*$_3$), 7.48 – 7.38 (m, 7H, *H*$_{34,30,6}$), 7.27 – 7.17 (m, 5H, *H*$_{31,5}$), 7.16 – 7.08 (m, 2H, *H*$_{32}$), 6.94 – 6.85 (m, 2H, *H*$_{35}$), 6.61 (d, *J* = 5.6 Hz, 0.43H, *H*$_{19}$), 6.52 (d, *J* = 5.6 Hz, 0.43H, *H*$_{18}$), 5.48 (s, 1.14H, *H*$_{18',19'}$), 4.82 – 4.77 (m, 2H, *H*$_9$), 4.76 (t, *J* = 2.4 Hz, 2H, *H*$_{37}$), 4.72 (d, *J* = 1.9 Hz, 0.93H, *H*$_{21}$), 4.58 (d, *J* = 12.8 Hz, 0.59H, *H*$_{21'}$), 4.51 (t, *J* = 5.1 Hz, 2H, *H*$_8$), 4.38 (d, *J* = 12.8 Hz, 0.56H, *H*$_{21'}$), 4.16 (d, *J* = 11.6 Hz, 0.45H, *H*$_{16}$), 3.94 – 3.85 (m, 1.05H, *H*$_{16,16'}$), 3.81 (d, *J* = 12.0 Hz, 0.74H, *H*$_{16'}$), 3.72 (d, *J* = 8.3 Hz, 0.63H, *H*$_{24'}$), 3.69 – 3.62 (m, 1.04H, *H*$_{25',15}$), 3.60 – 3.52 (m, 0.60H, *H*$_{15}$), 3.49 (t, *J* = 6.3 Hz, 1.15H, *H*$_{15'}$), 3.06 (t, *J* = 2.4 Hz, 0.51H, *H*$_{39}$), 3.04 (t, *J* = 2.4 Hz, 0.61H, *H*$_{39'}$), 2.99 – 2.92 (m, 1.57H, *H*$_{24,25}$, overlapped with water peak), 2.74 – 2.65 (m, 2H, *H*$_{12}$), 2.04 (s, 1.66H, *H*$_{23}$, overlapped with solvent peak), 1.97 (s, 1.66H, *H*$_{23'}$), 1.81 – 1.70 (m, 1.39H, *H*$_{13}$), 1.70 – 1.62 (m, 2.02H, *H*$_{13',14}$), 1.62 – 1.55 (m, 1.73H, *H*$_{14'}$).
^{13}C NMR (101 MHz, Acetone-*d*$_6$, 298 K) δ = 175.37 (C$_{26'}$), 175.17 (C$_{27'}$), 173.91 (C$_{26,27}$), 170.73 (C$_{22}$), 170.60 (C$_{22'}$), 167.29 (C$_1$), 159.34 (C$_4$), 157.28 (C$_{36}$), 157.15 (C$_{36'}$), 148.26 (C$_{11}$), 148.18 (C$_{11'}$), 143.88, 143.81 (C$_{29}$), 139.52 (C$_{19}$), 137.91 (C$_{18}$), 136.94 (C$_{18'}$), 135.91 (C$_{33'}$), 135.80 (C$_{19'}$), 135.73 (C$_{33}$), 132.94 (C$_2$), 131.16 (C$_{34}$), 131.07 (C$_{34'}$), 130.59 (C$_6$), 129.01, 128.98 (C$_{30}$), 128.30, 128.28 (C$_{31}$), 128.09 (C$_{31'}$), 127.07 (C$_{32}$), 126.87 (C$_{32'}$), 123.32 (C$_7$), 122.88 (C$_{10}$), 120.31 (C$_{5'}$), 120.26 (C$_5$), 116.16 (C$_3$), 114.33 (C$_{35}$), 114.27 (C$_{35'}$), 92.32 (C$_{17}$), 92.27 (C$_{17'}$), 90.74 (C$_{20}$), 90.21 (C$_{20'}$), 79.82 (C$_{38'}$), 79.80 (C$_{38}$), 77.02 (C$_{39}$), 76.97 (C$_{39'}$), 74.25 (C$_{28'}$), 73.90 (C$_{28}$), 72.13 (C$_{15}$), 71.90 (C$_{15'}$), 69.33 (C$_{16'}$), 68.98 (C$_{16}$), 67.74 (C$_8$), 62.84 (C$_{21'}$), 62.24 (C$_{21}$), 56.20 (C$_{37}$), 51.38 (C$_{24,25}$), 49.99 (C$_9$), 48.81 (C$_{25'}$), 47.98 (C$_{24'}$), 30.54 – 29.17 (C$_{14}$, overlapped with solvent peak), 26.88 (C$_{13}$), 26.82 (C$_{13'}$), 25.95 (C$_{12}$), 25.90 (C$_{12'}$), 20.70 (C$_{23}$), 20.57 (C$_{23'}$).
HRMS-ESI (+): 873.3104 [M+Na]$^+$, calculated for $C_{49}H_{46}N_4O_{10}Na^+$: 873.3106.

4.8.6 Synthesis of S33

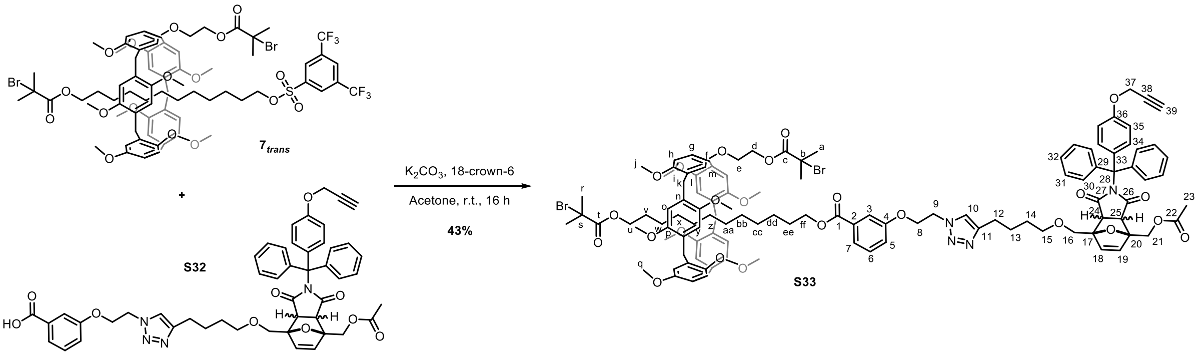

To a solution of **S32** (7 mg, 7.7 µmol, 1.0 eq.) in acetone (1 mL) was added K_2CO_3 (1.1 mg, 7.7 µmol, 1.0 eq.) and 18-crown-6 (2.0 mg, 7.7 µmol, 1.0 eq.). The mixture was stirred for 2 h at room temperature. $\mathbf{7}_{\mathit{trans}}$ (12 mg, 7.7 µmol, 1.0 eq.) was added and the mixture stirred for a further 16 h at room temperature. The solution was filtered, and the filtrate concentrated under vacuum. The residue was purified by preparative TLC (500 µm, DCM/MeOH, 40/1, eluted twice) to yield **S33** as a white powder (7 mg, 3.3 µmol, 43% yield).

The product is a mixture of stereoisomers including *exo* and *endo* with respect to the furan-maleimide Diels-Alder moiety. When distinguishable, *exo* and *endo* adducts are denoted as X_x and X_x' respectively.

^{1}H NMR (600 MHz, DMSO-d_6, 298 K) δ = 7.93 (s, 0.44H, H_{10}), 7.92 (s, 0.50H, $H_{10'}$), 7.58 (d, *J* = 7.9 Hz, 1H, H_7), 7.49 (t, *J* = 7.9 Hz, 1H, H_6), 7.45 – 7.42 (m, 1H, H_3), 7.34 – 7.17 (m, 11H, $H_{34,30,31,5}$), 7.15 – 7.09 (m, 2H, H_{32}), 6.90 – 6.86 (m, 1.21H, H_{35}), 6.86 – 6.77 (m, 11.63H, $H_{35',h,m,o}$), 6.51 (d, *J* = 5.5 Hz, 0.46H, H_{19}), 6.46 (d, *J* = 5.6 Hz, 0.46H, H_{18}), 5.37 – 5.31 (m, 1.20H, $H_{18',19'}$), 4.76 – 4.71 (m, 4H, $H_{37,9}$), 4.67 – 4.58 (m, 1.48H, $H_{21,d}$), 4.53 – 4.45 (m, 3.08H, $H_{21,21',8}$), 4.35 (d, *J* = 12.9 Hz, 0.55H, $H_{21'}$), 4.33 – 4.29 (m, 1H, H_d), 4.16 – 4.11 (m, 1H, H_e), 4.04 (dd, *J* = 11.7, 1.7 Hz, 0.49H, H_{16}), 4.02 – 3.96 (m, 1H, H_e), 3.79 – 3.58 (m, 47.34H, $H_{j,q,k,ff,u,16,16',24',25'}$), 3.57 – 3.37 (m, 3H, $H_{39,15}$), 2.97 (d, *J* = 6.5 Hz, 0.44H, H_{25}), 2.92 (dd, *J* = 6.5, 2.5 Hz, 0.44H, H_{24}), 2.63 (t, *J* = 7.6 Hz, 1.00H, H_{12}), 2.60 (t, *J* = 7.6 Hz, 1.09H, $H_{12'}$), 2.04 (s, 1.35H, H_{23}), 1.98 (s, 1.83H, $H_{23'}$), 1.92 (s, 6H, H_r), 1.90 – 1.87 (s, s, 6H, H_a), 1.67 – 1.61 (m, 1.25H, H_{13}), 1.60 – 1.54 (m, 2.22H, $H_{13',14}$), 1.52 – 1.47 (m, 1.52H, $H_{14'}$), 0.84 – 0.78 (m, 2H, H_v), 0.74 – 0.65 (m, 2H, H_{ee}), 0.45 – 0.34 (m, 4H, $H_{z,aa}$), 0.21 – 0.14 (m, 2H, H_y), 0.14 – 0.06 (m, 2H, H_{bb}), -0.21 – -0.29 (m, 2H, H_w), -0.31 – -0.44 (m, 6H, $H_{x,cc,dd}$).

^{13}C NMR (151 MHz, DMSO-d_6, 298 K) δ = 174.39 ($C_{26'}$), 174.28 ($C_{27'}$), 173.13 (C_{26}), 173.05 (C_{27}), 170.89 (C_c), 170.77 (C_t), 170.06 (C_{22}), 169.96 ($C_{22'}$), 165.41 (C_1), 158.01 (C_4), 155.76 (C_{36}), 155.54 ($C_{36'}$), 150.16, 149.70, 149.67, 149.64, 149.60, 149.59, 149.56 ($C_{i,p}$), 148.59 (C_f), 146.83 (C_{11}), 146.76 ($C_{11'}$), 142.49, 142.43, 142.41, 142.39 (C_{29}), 138.36 (C_{19}), 137.03 (C_{18}), 135.64 ($C_{19'}$), 134.64 ($C_{18'}$), 134.51 ($C_{33'}$), 134.18 (C_{33}), 131.34 (C_2), 130.09 (C_6), 129.82 (C_{34}), 129.66 ($C_{34'}$), 128.03, 127.79, 127.76, 127.63, 127.60, 127.48, 127.47 ($C_{30,31}$), 127.31, 127.29, 127.23, 127.16, 127.11, 127.07, 127.00 ($C_{g,l,n}$), 126.20 (C_{32}), 125.97 ($C_{32'}$), 122.32 (C_{10}), 121.65 (C_7), 119.26 (C_5), 115.01 (C_3), 114.43, 113.50, 113.38, 112.82, 112.46, 112.42, 112.37, 112.32, 112.29, 112.22 ($C_{35,h,m,o}$), 90.96 (C_{17}), 90.73 ($C_{17'}$), 89.22 (C_{20}), 88.83 ($C_{20'}$), 79.28 ($C_{38'}$), 79.25 (C_{38}), 78.27 (C_{39}), 78.15 ($C_{39'}$), 72.78 ($C_{28'}$), 72.44 (C_{28}), 70.83 (C_{15}), 70.63 ($C_{15'}$), 68.09 ($C_{16'}$), 67.75 (C_{16}), 66.50 (C_8), 66.07, 66.05 ($C_{e,u}$), 65.20 (C_{ff}), 64.21 (C_d), 61.71 ($C_{21'}$), 61.25 (C_{21}), 57.30 (C_s), 56.92 (C_b), 55.29, 55.28 (C_{37}), 54.93, 54.89, 54.84, 54.82, 54.76, 54.71 (C_j), 50.06 ($C_{24,25}$), 48.80 (C_9), 47.42 ($C_{25'}$), 46.73 ($C_{24'}$), 30.30, 30.26, 30.23, 30.19, 29.83, 29.67, 29.60, 29.51, 29.44, 29.12, 29.09, 29.01, 28.98, 28.89, 28.84, 28.82, 28.77, 28.73, 28.72, 28.69, 28.61, 28.58, 28.56, 28.52, 28.47, 28.45, 28.23, 27.98, 27.87, 27.70 ($C_{a,r,k,v,ee,x\text{-}cc,14}$), 25.60 ($C_{13}$), 25.56 ($C_{13'}$), 24.75 ($C_{12}$), 24.70 ($C_{12'}$), 23.82 ($C_{w,dd}$), 20.58 ($C_{23}$), 20.48 ($C_{23'}$), 13.96 ($C_1$).

HRMS-ESI (+): 2133.7574 $[M+Na]^+$, calculated for $C_{115}H_{132}Br_2N_4O_{24}Na^+$: 2133.7490.

4.8.7 Synthetic Route to Val-Cit Dipeptide Linker, S36

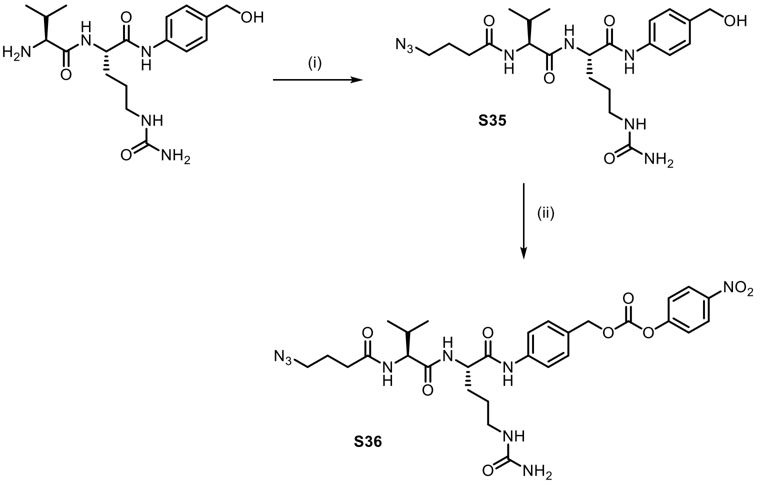

Figure S13. Synthetic route to **S36**. Conditions: (i) **S34**, DMF, r.t., 1 h, 91% yield; (ii) Bis(4-nitrophenyl)carbonate, Et_3N, DCM/DMF, 0 °C – r.t., 24 h, 68% yield.

4.8.8 Synthesis of S35

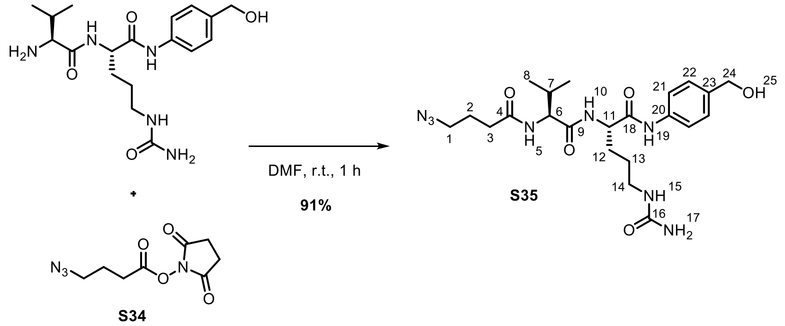

Val-Cit-PAB-OH (100 mg, 0.264 mmol, 1 eq.) and **S34** (66 mg, 0.290 mmol, 1.1 eq.) were dissolved in dry DMF (2.6 mL) and the mixture stirred at room temperature for 1 h. The mixture was condensed under vacuum. The crude material was washed thoroughly with Et_2O followed by DCM. The washed solid was collected to yield the pure product **S35** as a white powder (118 mg, 0.240 mmol, 91% yield).

^{1}H NMR (500 MHz, DMSO-d_6, 298 K) δ = 9.89 (s, 1H, H_{19}), 8.08 (d, J = 7.6 Hz, 1H, H_{10}), 7.92 (d, J = 8.6 Hz, 1H, H_5), 7.58 – 7.52 (m, 2H, H_{21}), 7.25 – 7.20 (m, 2H, H_{22}), 5.97 (t, J = 5.9 Hz, 1H, H_{15}), 5.41 (s, 2H, H_{17}), 5.09 (t, J = 5.7 Hz, 1H, H_{25}), 4.42 (d, J = 5.7 Hz, 2H, H_{24}), 4.38 (td, J = 7.9, 5.3 Hz, 1H, H_{11}), 4.19 (dd, J = 8.6, 6.7 Hz, 1H, H_6), 3.31 (t, J = 6.9 Hz, 1H, H_1), 3.06 – 2.90 (m, 2H, H_{14}), 2.32 – 2.21 (m, 2H, H_3), 2.04 – 1.93 (m, 1H, H_7), 1.79 – 1.71 (m, 2H, H_2), 1.71 – 1.66 (m, 1H, H_{12}), 1.63 – 1.53 (m, 1H, H_{12}), 1.49 – 1.31 (m, 2H, H_{13}), 0.87 (d, J = 6.7 Hz, 3H, H_8), 0.84 (d, J = 6.7 Hz, 3H, H_8).
^{13}C NMR (126 MHz, DMSO-d_6, 298 K) δ = 171.60 (C_4), 171.20 (C_9), 170.40 (C_{18}), 158.87 (C_{16}), 137.53 (C_{20}), 137.41 (C_{23}), 126.93 (C_{22}), 118.83 (C_{21}), 62.59 (C_{24}), 57.74 (C_6), 53.07 (C_{11}), 50.29 (C_1), 38.59 (br, C_{14}), 32.05 (C_3), 30.34 (C_7), 29.36 (C_{12}), 26.84 (C_{13}), 24.65 (C_2), 19.24, 18.19 (C_8).
HRMS-ESI (+): 513.2536 $[M+Na]^+$, calculated for $C_{22}H_{34}N_8O_5Na^+$: 513.2544.

4.8.9 Synthesis of S36

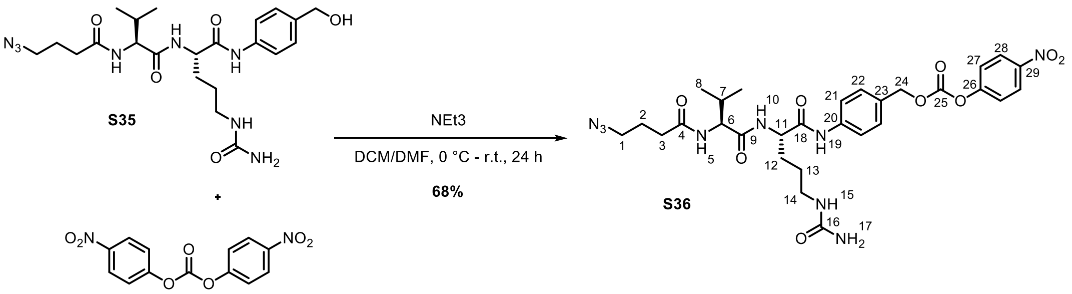

S35 (100 mg, 0.204 mmol, 1 eq.) and bis(4-nitrophenyl)carbonate (93 mg, 0.306 mmol, 1.5 eq.) were added to dry DCM (2 mL) along with triethylamine (62 mg, 85 µL, 0.612 mmol, 3 eq.). The mixture was placed under a nitrogen atmosphere and cooled in an ice bath. DMF (8 mL) was added dropwise to the mixture. The mixture was stirred for 1 h before being allowed to warm to room temperature. The mixture was stirred for a further 23 h before being condensed under vacuum. The crude material was washed thoroughly with Et_2O followed by DCM. The washed solid was collected to yield the pure product **S36** as a light yellow powder (91 mg, 0.139 mmol, 68% yield).

^{1}H NMR (500 MHz, DMSO-d_6, 298 K) δ = 10.06 (s, 1H, H_{19}), 8.33 – 8.29 (m, 2H, H_{28}), 8.12 (d, J = 7.4 Hz, 1H, H_{10}), 7.92 (d, J = 8.5 Hz, 1H, H_5), 7.68 – 7.64 (m, 2H, H_{21}), 7.59 – 7.54 (m, 2H, H_{22}), 7.43 – 7.39 (m, 2H, H_{27}), 5.98 (t, J = 5.8 Hz, 1H, H_{15}), 5.42 (s, 2H, H_{17}), 5.24 (s, 2H, H_{24}), 4.39 (td, J = 8.3, 5.2 Hz, 1H, H_{11}), 4.20 (dd, J = 8.6, 6.7 Hz, 1H, H_6), 3.32 (t, J = 6.9 Hz, 2H, H_1), 3.08 – 2.90 (m, 2H, H_{14}), 2.32 – 2.21 (m, 2H, H_3), 2.03 – 1.93 (m, 1H, H_7), 1.80 – 1.74 (m, 2H, H_2), 1.72 – 1.66 (m, 1H, H_{12}), 1.65 – 1.55 (m, 1H, H_{12}), 1.50 – 1.32 (m, 2H, H_{13}), 0.87 (d, J = 6.8 Hz, 3H, H_8), 0.84 (d, J = 6.8 Hz, 3H, H_8).
^{13}C NMR (126 MHz, DMSO-d_6, 298 K) δ = 171.60 (C_4), 171.27 (C_9), 170.77 (C_{18}), 158.91 (C_{16}), 155.30 (C_{26}), 151.97 (C_{25}), 145.18 (C_{29}), 139.41 (C_{20}), 129.51 (C_{22}), 129.29 (C_{23}), 125.42 (C_{28}), 122.64 (C_{27}), 119.02 (C_{21}), 70.27 (C_{24}), 57.72 (C_6), 53.15 (C_{11}), 50.29 (C_1), 38.55 (C_{14}), 32.05 (C_3), 30.37 (C_7), 29.24 (C_{12}), 26.86 (C_{13}), 24.65 (C_2), 19.23, 18.21 (C_8).
HRMS-ESI (+): 678.2594 $[M+Na]^+$, calculated for $C_{29}H_{37}N_9O_9Na^+$: 678.2606.

4.8.10 Synthetic Route to Reference Compound, 12

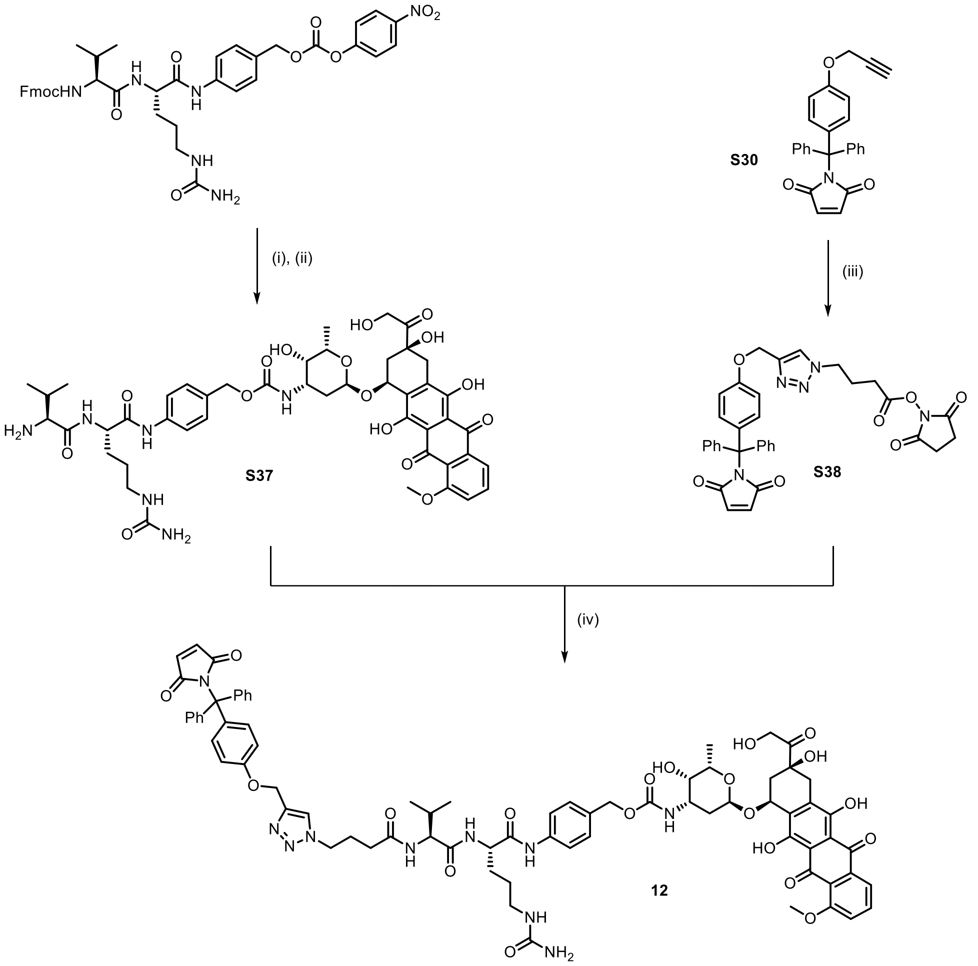

Figure S14. Synthetic route to **12**. Conditions: (i) Doxorubicin hydrochloride, DIPEA, DMF, r.t., 24 h; (ii) Piperidine, DMF, r.t., 2 min, 83% yield; (iii) **S34**, CuBr, PMDETA, DCM, r.t., 2 h, 96% yield; (iv) DMF, -15 °C – r.t., 1 h, 77% yield.

4.8.11 Synthesis of S37

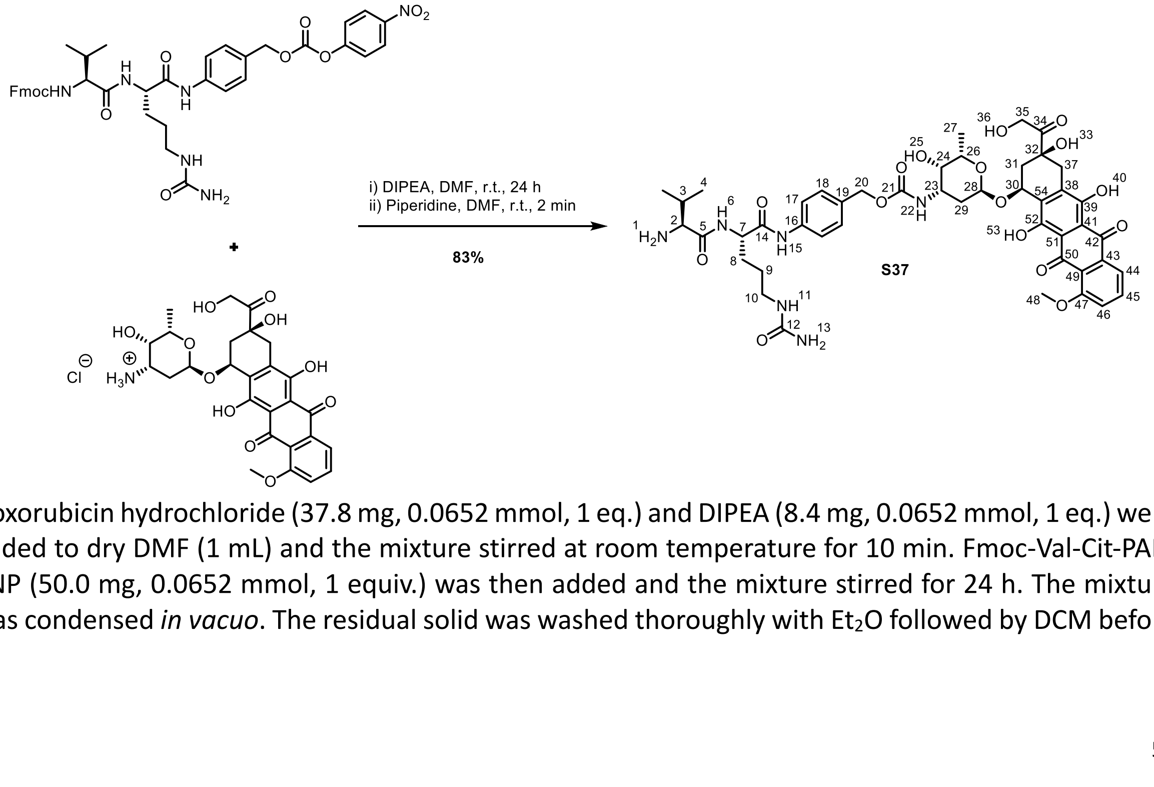

Doxorubicin hydrochloride (37.8 mg, 0.0652 mmol, 1 eq.) and DIPEA (8.4 mg, 0.0652 mmol, 1 eq.) were added to dry DMF (1 mL) and the mixture stirred at room temperature for 10 min. Fmoc-Val-Cit-PAB-PNP (50.0 mg, 0.0652 mmol, 1 equiv.) was then added and the mixture stirred for 24 h. The mixture was condensed *in vacuo*. The residual solid was washed thoroughly with Et_2O followed by DCM before

being redissolved in dry DMF (1 mL) and placed under a nitrogen atmosphere. Piperidine (0.3 mL) was added and the mixture stirred for 2 min at room temperature. The reaction was rapidly diluted with a mixture of diethyl ether (100 mL) and DCM (50 mL). The mixture was filtered with the collected solid being the product **S37** as a dark red powder (51.4 mg, 0.0542 mmol, 83% yield).

^{1}H NMR (500 MHz, DMSO-d_6, 298 K) δ = 10.09 (s, 1H, H_{15}), 8.13 (d, J = 8.3 Hz, 1H, H_6), 7.97 – 7.85 (m, 2H, $H_{44,45}$), 7.68 – 7.62 (m, 1H, H_{46}), 7.56 – 7.52 (m, 2H, H_{17}), 7.26 – 7.22 (m, 2H, H_{18}), 6.84 (d, J = 8.0 Hz, 1H, H_{22}), 5.97 (t, J = 6.0 Hz, 1H, H_{11}), 5.46 (s, 1H, H_{33}), 5.39 (s, 2H, H_{13}), 5.23 – 5.19 (m, 1H, H_{28}), 4.96 – 4.92 (m, 1H, H_{30}), 4.91 – 4.82 (m, 3H, $H_{20,36}$), 4.70 (d, J = 5.7 Hz, 1H, H_{25}), 4.57 (s, 2H, H_{35}), 4.49 – 4.41 (m, 1H, H_7), 4.18 – 4.12 (m, 1H, H_{26}), 3.98 (s, 3H, H_{48}), 3.76 – 3.67 (m, 1H, H_{23}), 3.47 – 3.41 (m, 1H, H_{24}), 3.06 – 3.01 (m, 1H, H_2), 3.01 – 2.90 (m, 4H, $H_{10,37}$), 2.25 – 2.07 (m, 2H, H_{31}), 1.96 – 1.88 (m, 1H, H_3), 1.87 – 1.79 (m, 1H, H_{29}), 1.70 – 1.51 (m, 2H, H_8), 1.50 – 1.44 (m, 1H, H_{29}), 1.43 – 1.28 (m, 2H, H_9), 1.12 (d, J = 6.4 Hz, 3H, H_{27}), 0.87 (d, J = 6.8 Hz, 3H, H_4), 0.77 (d, J = 6.8 Hz, 3H, H_4).
^{13}C NMR (126 MHz, DMSO-d_6, 298 K) δ = 213.80 (C_{34}), 186.53 ($C_{42,50}$), 174.23 (C_5), 170.66 (C_{14}), 160.81 (C_{47}), 158.86 (C_{12}), 156.13 ($C_{39,52}$), 155.32 (C_{21}), 154.58 ($C_{39,52}$), 138.43 (C_{16}), 136.23 (C_{45}), 135.56 (C_{54}), 134.75 (C_{43}), 134.16 (C_{38}), 131.92 (C_{19}), 128.54 (C_{18}), 120.06 (C_{49}), 119.73 (C_{46}) 118.99 ($C_{17,44}$), 110.83, 10.68 ($C_{41,51}$), 100.28 (C_{28}), 74.98 (C_{32}), 69.89 (C_{30}), 67.99 (C_{24}), 66.68 (C_{26}), 64.92 (C_{20}), 63.68 (C_{35}), 59.56 (C_2), 56.60 (C_{48}), 52.52 (C_7), 47.14 (C_{23}), 38.59 (C_{10}), 36.67 (C_{31}), 32.11 (C_{37}), 31.30 (C_3), 30.05 (C_{29}), 29.84 (C_8), 26.69 (C_9), 19.50 (C_4), 17.03 (C_{27}), 16.95 (C_4).
HRMS-ESI (-): 947.3658 $[M-H]^-$, calculated for $C_{46}H_{55}N_6O_{16}^-$: 642.3680.

4.8.12 Synthesis of S38

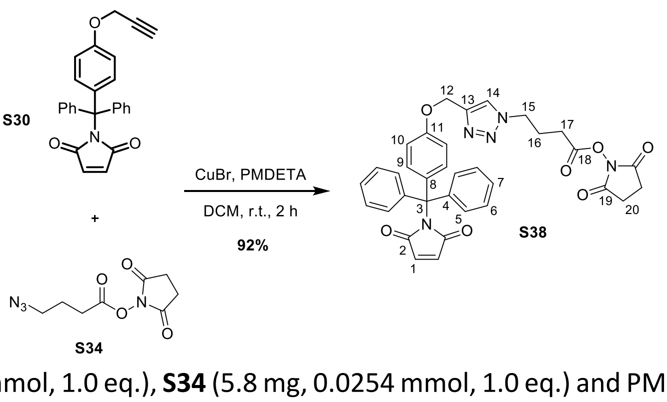

S30 (10.0 mg, 0.0254 mmol, 1.0 eq.), **S34** (5.8 mg, 0.0254 mmol, 1.0 eq.) and PMDETA (0.9 mg, 0.00509 mmol, 0.2 eq.) were dissolved in DCM (0.25 mL) in a sealed 5 mL microwave vial. The mixture was degassed *via* three freeze-thaw-pump cycles. In a separate sealed 5 mL microwave vial was added CuBr (0.4 mg, 0.00254 mmol, 0.1 eq.) which was placed under a nitrogen atmosphere. The reaction mixture was transferred *via* cannula onto the CuBr. The mixture was stirred for 2 h at room temperature. The mixture was washed with aqueous EDTA solution (0.25 M, pH 7, 10 mL) and brine (10 mL). The organic phase was dried over magnesium sulfate before being filtered and concentrated under vacuum to yield the product **S38** as a white powder (14.5 mg, 0.0234 mmol, 92% yield).

^{1}H NMR (400 MHz, Acetone-d_6, 298 K) δ = 8.09 (s, 1H, H_{14}), 7.46 – 7.41 (m, 4H, H_5), 7.40 – 7.37 (m, 2H, H_9), 7.27 – 7.20 (m, 4H, H_6), 7.18 – 7.12 (m, 2H, H_7), 6.95 – 6.90 (m, 2H, H_{10}), 6.77 (s, 2H, H_1), 5.15 (s, 2H, H_{12}), 4.58 (t, J = 7.0 Hz, 2H, H_{15}), 2.88 (s, 4H, H_{20}), 2.75 (t, J = 7.4 Hz, 2H, H_{17}), 2.39 – 2.31 (m, 2H, H_{16}).
^{13}C NMR (101 MHz, Acetone-d_6, 298 K) δ = 171.55 (C_2), 170.48 (C_{19}), 169.07 (C_{18}), 158.07 (C_{11}), 144.39 (C_{13}), 144.37 (C_4), 136.12 (C_9), 135.74 (C_1), 131.07 (C_9), 129.26 (C_5), 128.23 (C_6), 127.04 (C_7), 124.86 (C_{14}), 114.38 (C_{10}), 73.38 (C_3), 62.40 (C_{12}), 49.25 (C_{15}), 28.44 (C_{17}), 26.28 (C_{20}), 26.24 (C_{16}).
HRMS-ESI (+): 642.1949 $[M+Na]^+$, calculated for $C_{34}H_{29}N_5O_7Na^+$: 642.1959.

4.8.13 Synthesis of 12

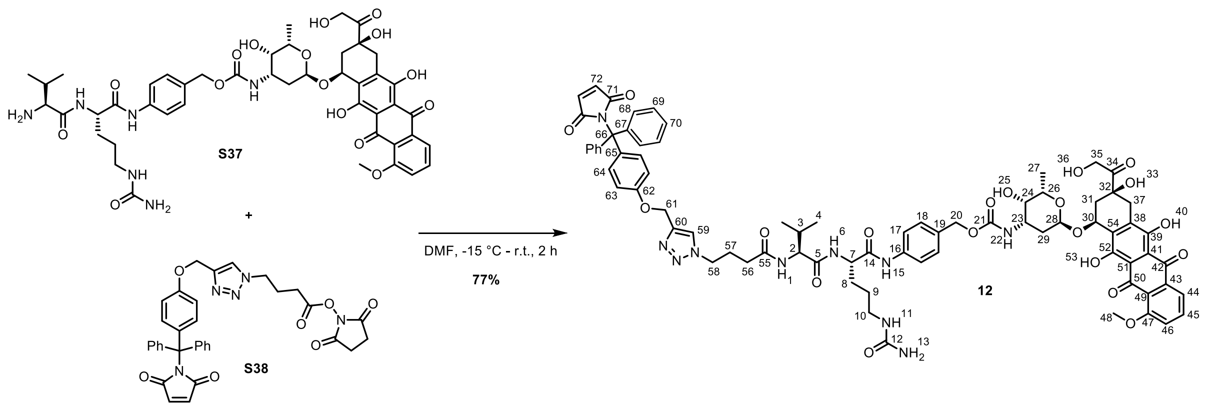

S38 (10.0 mg, 0.0162 mmol, 1.2 eq.) was dissolved in DMF (65 µL). The reaction vessel was placed under a nitrogen atmosphere and the mixture cooled in a salt-ice bath to -15 °C. A solution of **S37** (12.8 mg, 0.0135 mmol, 1.0 eq.) in DMF (65 µL) was added slowly over the course of 5 min to the reaction mixture. The reaction mixture was stirred and allowed to warm to room temperature over the course of 2 h. The mixture was concentrated under vacuum. The crude material was washed thoroughly with Et_2O followed by DCM. The washed solid was collected to yield the product **12** as a bright red powder (15.1 mg, 0.0104 mmol, 77% yield).

^{1}H NMR (500 MHz, DMSO-d_6, 298 K) δ = 14.01 (s, 1H, $H_{40,53}$), 13.25 (s, 1H, $H_{40,53}$), 9.95 (s, 1H, H_{15}), 8.21 (s, 1H, H_{59}), 8.10 (d, J = 7.7 Hz, 1H, H_6), 7.93 (d, J = 8.6 Hz, 1H, H_1), 7.92 – 7.85 (m, 2H, $H_{44,45}$), 7.67 – 7.61 (m, 1H, H_{46}), 7.57 – 7.50 (m, 2H, H_{17}), 7.37 – 7.32 (m, 4H, H_{68}), 7.31 – 7.27 (m, 2H, H_{64}), 7.26 – 7.22 (m, 6H, $H_{18,69}$), 7.17 – 7.12 (m, 2H, H_{70}), 6.96 – 6.92 (m, 2H, H_{63}), 6.91 (s, 2H, H_{72}), 6.83 (d, J = 8.0 Hz, 1H, H_{22}), 5.97 (t, J = 5.9 Hz, 1H, H_{11}), 5.45 (s, 1H, H_{33}), 5.40 (s, 2H, H_{13}), 5.23 – 5.19 (m, 1H, H_{28}), 5.08 (s, 2H, H_{61}), 4.95 – 4.91 (m, 1H, H_{30}), 4.90 – 4.83 (m, 3H, $H_{20,36}$), 4.71 (d, J = 5.7 Hz, 1H, H_{25}), 4.57 (d, J = 4.9 Hz, 2H, H_{35}), 4.39 – 4.32 (m, 3H, $H_{7,58}$), 4.23 – 4.12 (m, 2H, $H_{2,26}$), 3.97 (s, 3H, H_{48}), 3.75 – 3.67 (m, 1H, H_{23}), 3.46 – 3.43 (m, 1H, H_{24}), 3.05 – 2.87 (m, 4H, $H_{10,37}$), 2.24 – 2.15 (m, 3H, $H_{31,56}$), 2.13 – 2.08 (m, 1H, H_{31}), 2.07 – 2.00 (m, 2H, H_{57}), 1.99 – 1.93 (m, 1H, H_3), 1.89 – 1.79 (m, 1H, H_{29}), 1.73 – 1.52 (m, 2H, H_8), 1.51 – 1.27 (m, 3H, $H_{9,29}$), 1.12 (d, J = 6.3 Hz, 3H, H_{27}), 0.85 (d, J = 6.7 Hz, 3H, H_4), 0.82 (d, J = 6.7 Hz, 3H, H_4).

^{13}C NMR (126 MHz, DMSO-d_6, 298 K) δ = 214.28 (C_{34}), 187.02, 186.93 ($C_{50,53}$), 171.87 (C_{55}), 171.67 (C_5), 171.26 (C_{71}), 171.04 (C_{14}), 161.25 (C_{47}), 159.34 (C_{12}), 157.00 (C_{62}), 156.57 ($C_{39,52}$), 155.78 (C_{21}), 154.97 ($C_{39,52}$), 143.54 (C_{67}), 143.08 (C_{60}), 138.97 (C_{16}), 136.69 (C_{45}), 136.01 (C_{54}), 135.54 (C_{72}), 135.22 (C_{65}), 135.14 (C_{43}), 134.56 (C_{38}), 132.26 (C_{19}), 130.28 (C_{64}), 128.99 (C_{18}), 128.43 (C_{68}), 127.92 (C_{69}), 126.62 (C_{70}), 124.95 (C_{59}), 120.48 (C_{49}), 120.19 (C_{46}), 119.35 ($C_{17,44}$), 113.95 (C_{63}), 111.25, 111.11 ($C_{41,51}$), 100.76 (C_{28}), 75.42 (C_{32}), 72.33 (C_{65}), 70.33 (C_{30}), 68.45 (C_{26}), 67.13 (C_{24}), 65.38 (C_{20}), 64.15 (C_{35}), 61.55 (C_{61}), 58.19 (C_2), 57.05 (C_{48}), 53.55 (C_7), 49.47 (C_{58}), 47.59 (C_{23}), 39.00 (C_{10}), 37.09 (C_{31}), 32.55 (C_{37}), 32.28 (C_{56}), 30.80 (C_3), 30.30 (C_{29}), 29.71 (C_8), 27.28 (C_9), 26.53 (C_{57}), 19.69, 18.64 (C_4), 17.49 (C_{27}).

HRMS-ESI (+): 1475.5403 $[M+Na]^+$, calculated for $C_{76}H_{80}N_{10}O_{20}Na^+$: 1475.5443.

4.9 Synthesis of Alternative-Cargo Compounds – N-(1-pyrenyl)maleimide Cargo Release

4.9.1 Synthetic Route to N-(1-pyrenyl)maleimide-Cargo-Containing Rotaxane

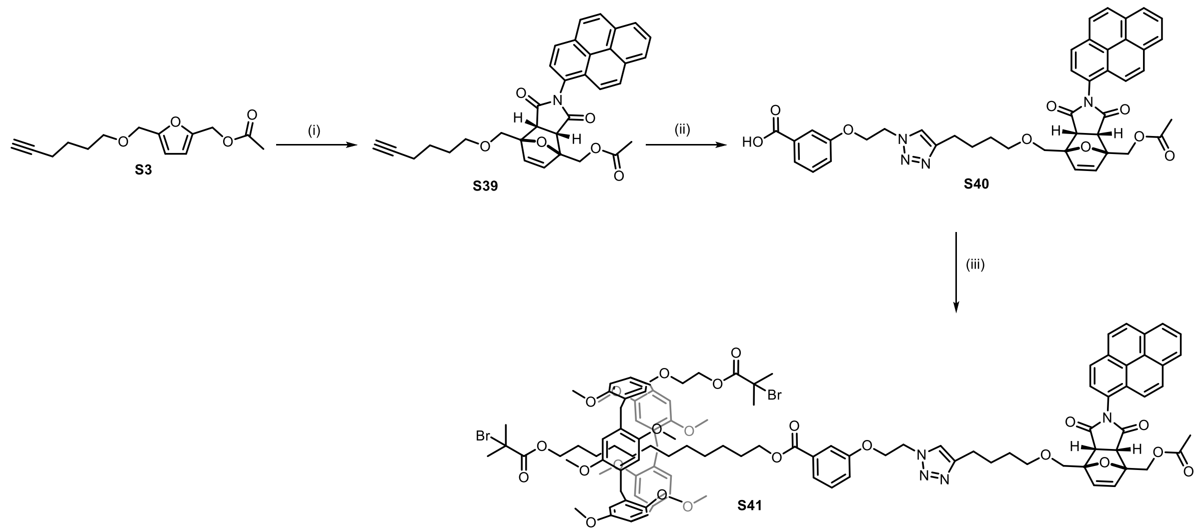

Figure S15. Synthetic route to **S41**. Conditions: (i) DMF, 80 °C, 16 h, 15% yield; (ii) **S7**, CuBr, PMDETA, DCM, r.t., 16 h, 61% yield; (iii) **7***trans*, K_2CO_3, 18-crown-6, acetone, r.t., 16 h, 86% yield.

4.9.2 Synthesis of S39

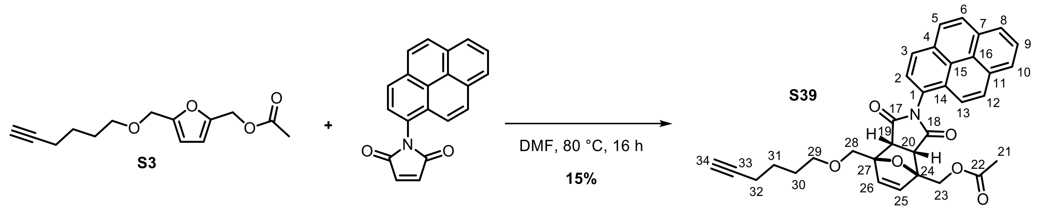

A solution of **S3** (90 mg, 0.36 mmol, 1.0 eq.) and N-(1-pyrenyl)maleimide (107 mg, 0.36 mmol, 1.0 eq.) in DMF (1 mL) was stirred at 80 °C for 16 h. The reaction mixture was then diluted with DCM (5 mL) and the resulting solution washed by water (2 x 5 mL) and brine (1 x 5 mL). The organic phase was separated and dried with magnesium sulfate before being filtered and concentrated under vacuum. The crude material was purified by preparative TLC (2000 µm, PE/Et_2O, 1/2, eluted twice) to yield **S39** (30 mg, 0.11 mmol, 15% yield) as a faint yellow powder.

S39 is observable as two interconverting isomers by NMR spectroscopy because of the slow rotation of the C_1-N bond. When distinguishable, these two isomers are denoted as X_x and X_x’.

^{1}H NMR (400 MHz, Acetone-d_6, 298 K) δ = 8.38 – 8.30 (m, 3H, $H_{3,8,10}$), 8.29 – 8.16 (m, 3H, $H_{12,5,6}$), 8.15 – 8.07 (m, 1H, H_9), 8.04 (d, *J* = 9.2 Hz, 0.64H, H_{13}), 7.91 (d, *J* = 9.2 Hz, 0.35H, $H_{13'}$), 7.87 (d, *J* = 8.1 Hz, 0.34H, $H_{2'}$), 7.74 (d, *J* = 8.1 Hz, 0.63H, H_2), 7.03 (s, 0.69H, $H_{25',26'}$), 6.82 (s, 1.27H, $H_{25,26}$), 4.95 – 4.85 (m, 1H, H_{23}), 4.66 – 4.57 (m, 1H, H_{23}), 4.22 – 3.94 (m, 4H, $H_{28,19,20}$), 3.71 – 3.58 (m, 2H, H_{29}), 2.34 (t, *J* = 2.7 Hz, 1H, H_{34}), 2.24 (td, *J* = 7.0, 2.6 Hz, 2H, H_{32}), 2.11 – 2.09 (s, s, 3H, H_{21}), 1.79 – 1.69 (m, 2H, H_{30}), 1.68 – 1.59 (m, 2H, H_{31}).

^{13}C NMR (101 MHz, Acetone-d_6, 298 K) δ = 175.54, 175.37 ($C_{17,18}$), 175.02, 174.87 ($C_{17',18'}$), 170.82, 170.81 (C_{22}), 138.49 ($C_{26'}$), 137.61 (C_{26}), 137.50 ($C_{25'}$), 136.61 (C_{25}), 132.70 (C_4), 132.66 ($C_{4'}$), 131.94, 131.92, 131.65, 131.55 ($C_{7,11}$), 129.54 (C_{12}), 129.36, 129.31, 129.29 ($C_{12',5}$), 128.92 (C_{14}), 128.90 ($C_{14'}$), 127.97 (C_6), 127.51 (C_9), 127.48 ($C_{2'}$), 127.41 ($C_{1'}$), 127.32 (C_1), 126.93, 126.90, 126.74, 126.68 ($C_{8,10,2}$),

125.90 ($C_{3'}$), 125.81 (C_3), 125.69 (C_{15}), 125.61 ($C_{15'}$), 124.94 ($C_{16'}$), 124.92 (C_{16}), 123.00 (C_{13}), 122.90 ($C_{13'}$), 92.91 (C_{27}), 92.77 ($C_{27'}$), 90.85 (C_{24}), 90.73 ($C_{24'}$), 84.88 (C_{33}), 71.79 (C_{29}), 70.04 (C_{34}), 69.56 ($C_{28'}$), 69.46 (C_{28}), 62.98 ($C_{23'}$), 62.89 (C_{23}), 50.73 ($C_{20'}$), 49.86 (C_{20}), 49.78 ($C_{19'}$), 48.93 (C_{19}), 30.53 – 29.18 (C_{30}, overlapped with solvent peak), 26.05 (C_{31}), 20.69 (C_{21}), 18.51 (C_{32}).
HRMS-ESI (+): 570.1887 $[M+Na]^+$, calculated for $C_{34}H_{29}NO_6Na^+$: 570.1898.

4.9.3 Synthesis of S40

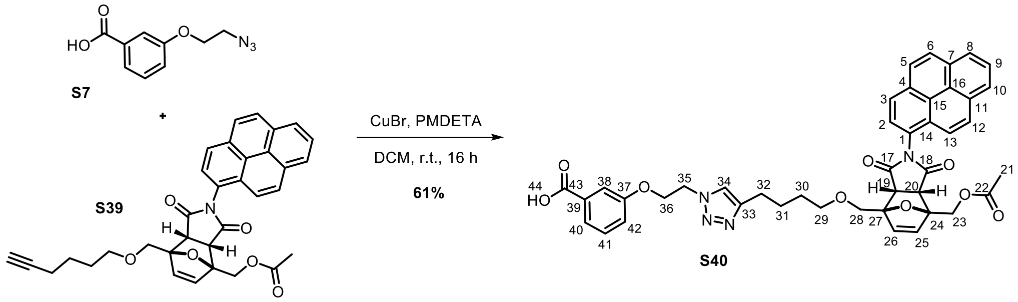

Three sealed 5 mL microwave vials (*A*, *B*, and *C*) were prepared as follows: *A* contained PMDETA (11 mg, 65 µmol, 1.5 eq.) in DCM (3 mL), *B* contained CuBr (10 mg, 70 µmol, 1.6 eq.), and *C* contained **S39** (24 mg, 44 µmol, 1.0 eq.) and **S7** (9 mg, 44 µmol, 1.0 eq.). Vials *B* and *C* were subjected to three N_2/vacuum cycles before use. The solution in vial *A* was degassed by bubbling with N_2 until ~1 mL DCM was left before being transferred to vial *B via* cannula; the resulting mixture was stirred until all the CuBr had dissolved. The CuBr/PMDETA solution in vial *B* was then transferred to vial *C via* cannula; the resulting reaction mixture was stirred for 16 h at room temperature. The reaction mixture was washed with aqueous EDTA solution (0.25 M, pH 7, 2 x 1 mL) and brine (2 x 1 mL). The organic layer was collected and dried over magnesium sulfate. The mixture was filtered before being concentrated under vacuum. The residue was purified by preparative TLC (500 µm, DCM/MeOH, 20/1, eluted twice) to yield **S40** as a faint yellow powder (20 mg, 27 µmol, 61% yield).

S40 is observable as two interconverting isomers by NMR spectroscopy because of the slow rotation of the C_1-N bond. When distinguishable, these two isomers are denoted as X_x and X_x'. Additionally, 3% of the mixture is not loaded with the N-(1-pyrenyl)maleimide unit and exists as the furan derivative, which is denoted as X_x'' if distinguishable.

^{1}H NMR (400 MHz, Acetone-d_6, 298 K) δ = 8.37 – 8.29 (m, 3H, $H_{3,8,10}$), 8.29 – 8.16 (m, 3H, $H_{12,5,6}$), 8.13 – 8.07 (m, 1H, H_9), 8.04 (d, *J* = 9.2 Hz, 0.62H, H_{13}), 7.91 (d, *J* = 9.2 Hz, 0.34H, $H_{13'}$), 7.87 (d, *J* = 8.2 Hz, 0.33H, $H_{2'}$), 7.83 (s, 1H, H_{34}), 7.74 (d, *J* = 8.1 Hz, 0.62H, H_2), 7.63 (dt, *J* = 7.7, 1.2 Hz, 1H, H_{40}), 7.57 – 7.51 (m, 1H, H_{38}), 7.38 (t, *J* = 8.0 Hz, 1H, H_{41}), 7.19 – 7.12 (m, 1H, H_{42}), 7.02 (s, 0.67H, $H_{25',26'}$), 6.81 (s, 1.26H, $H_{25,26}$), 6.41 (d, *J* = 3.1 Hz, 0.03H, $H_{25''}$), 6.33 (d, *J* = 3.3 Hz, 0.03H, $H_{26''}$), 5.00 (s, 0.06H, $H_{23''}$), 4.93 – 4.85 (m, 1H, H_{23}), 4.77 (t, *J* = 5.2 Hz, 2H, H_{35}), 4.65 – 4.58 (m, 1H, H_{23}), 4.54 – 4.43 (m, 2H, H_{36}), 4.38 (s, 0.06H, $H_{28''}$), 4.20 – 3.94 (m, 4H, $H_{28,19,20}$), 3.68 – 3.60 (m, 2H, H_{29}), 2.72 (t, *J* = 7.4 Hz, 2H, H_{32}), 2.08 – 2.07 (s, s, 3H, H_{21}), 1.83 – 1.73 (m, 2H, H_{31}), 1.72 – 1.63 (m, 2H, H_{30}).
^{13}C NMR (101 MHz, Acetone-d_6, 298 K) δ = 175.58, 175.38 ($C_{17,18}$), 175.07, 174.88 ($C_{17',18'}$), 170.83, 170.82 (C_{22}), 167.30 (C_{43}), 159.28 (C_{37}), 148.22 (C_{33}), 138.52 ($C_{26'}$), 137.65 (C_{26}), 137.48 ($C_{25'}$), 136.59 (C_{25}), 132.91 (C_{39}), 132.69 (C_4), 132.65 ($C_{4'}$), 131.93, 131.91, 131.64, 131.54 ($C_{7,11}$), 130.55 (C_{41}), 129.55 (C_{12}), 129.37, 129.30, 129.28 ($C_{12',5}$), 128.92 (C_{14}), 128.89 ($C_{14'}$), 127.96 (C_6), 127.50 (C_9), 127.48 ($C_{2'}$), 127.40 ($C_{1'}$), 127.31 (C_1), 126.93, 126.89, 126.74, 126.68 ($C_{8,10,2}$), 125.90 ($C_{3'}$), 125.81 (C_3), 125.68 (C_{15}), 125.60 ($C_{15'}$), 124.93 ($C_{16'}$), 124.91 (C_{16}), 123.29 (C_{40}), 123.00 (C_{13}), 122.92 (C_{34}), 122.89 ($C_{13'}$), 120.23 (C_{42}), 116.12 (C_{38}), 92.90 (C_{27}), 92.76 ($C_{27'}$), 90.85 (C_{24}), 90.73 ($C_{24'}$), 72.09, 72.06 (C_{29}), 69.52 ($C_{28'}$), 69.42 (C_{28}), 67.66 (C_{36}), 62.97 ($C_{23'}$), 62.88 (C_{23}), 50.74 ($C_{20'}$), 49.98 (C_{35}), 49.87 (C_{20}), 49.77 ($C_{19'}$), 48.93 (C_{19}), 30.76 – 29.08 (C_{30}, overlapped with solvent peak), 26.87 (C_{31}), 25.92 (C_{32}), 20.69 (C_{21}).
HRMS-ESI (+): 777.2527 $[M+Na]^+$, calculated for $C_{43}H_{38}N_4O_9Na^+$: 777.2531.

4.9.4 Synthesis of S41

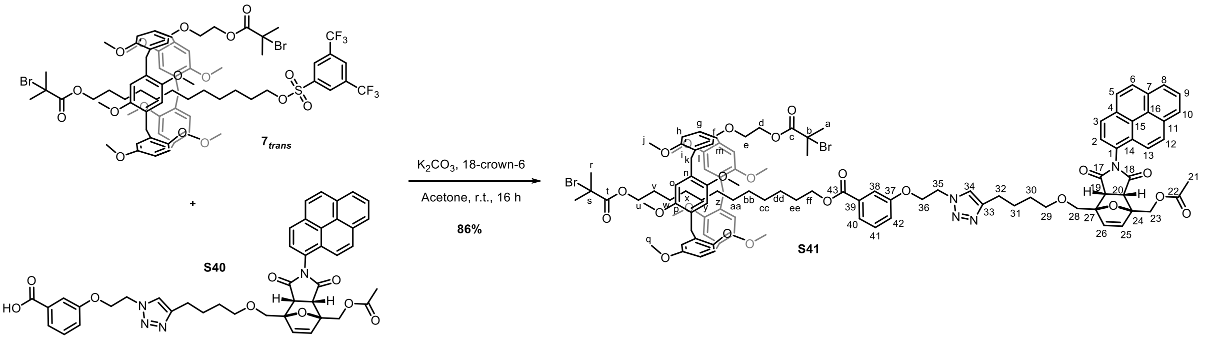

To a solution of **S40** (4 mg, 5.8 µmol, 1.0 eq.) in acetone (1 mL) was added K_2CO_3 (0.8 mg, 5.8 µmol, 1.0 eq.) and 18-crown-6 (1.5 mg, 5.8 µmol, 1.0 eq.). The mixture was stirred for 2 h at room temperature. **7*trans*** (9 mg, 5.8 µmol, 1.0 eq.) was added and the mixture stirred for a further 16 h at room temperature. The solution was filtered, and the filtrate concentrated under vacuum. The residue was purified by preparative TLC (500 µm, DCM/MeOH, 40/1, eluted twice) to yield **S41** as a white powder (10 mg, 5.0 µmol, 86% yield).

S41 is observable as two interconverting isomers by NMR spectroscopy because of the slow rotation of the C_1-N bond. When distinguishable, these two isomers are denoted as X_x and X_x'. Additionally, 6% of the mixture is not loaded with the N-(1-pyrenyl)maleimide unit and exists as the furan derivative, which is denoted as X_x'' if distinguishable.

^{1}H NMR (500 MHz, DMSO-*d*$_6$, 298 K) δ = 8.44 – 8.33 (m, 3H, $H_{3,8,10}$), 8.31 – 8.20 (m, 3H, $H_{12,5,6}$), 8.14 (t, *J* = 7.6 Hz, 1H, H_9), 8.11 (d, *J* = 9.2 Hz, 0.61H, H_{13}), 7.95 (s, 1H, H_{34}), 7.91 (d, *J* = 8.1 Hz, 0.38H, $H_{2'}$), 7.73 (d, *J* = 9.2 Hz, 0.32H, $H_{13'}$), 7.69 (d, *J* = 8.1 Hz, 0.62H, H_2), 7.56 (dt, *J* = 7.8, 1.2 Hz, 1H, H_{40}), 7.47 (t, *J* = 7.9 Hz, 1H, H_{38}), 7.44 – 7.41 (m, 1H, H_{38}), 7.25 – 7.21 (m, 1H, H_{42}), 7.00 – 6.96 (m, 0.71H, $H_{25',26'}$), 6.85 – 6.78 (m, 10H, $H_{h,m,o}$), 6.77 (s, 1.35H, $H_{25,26}$), 6.45 (d, *J* = 2.9 Hz, 0.06H, $H_{25''}$), 6.37 (d, *J* = 3.3 Hz, 0.06H, $H_{26''}$), 4.99 (s, 0.12H, $H_{23''}$), 4.83 – 4.69 (m, 3H, $H_{23,25}$), 4.64 – 4.56 (m, 2H, $H_{d,23}$), 4.49 – 4.41 (m, 2H, H_{36}), 4.34 (s, 0.13H, $H_{28''}$), 4.34 – 4.27 (m, 1H, H_d), 4.15 – 4.10 (m, 1H, H_e), 4.09 – 3.92 (m, 5H, $H_{e,28,19,20}$), 3.80 – 3.58 (m, 41H, $H_{j,q,k,ff,u}$), 3.57 – 3.52 (m, 2H, H_{29}), 2.65 (t, *J* = 7.4 Hz, 2H, H_{32}), 2.08 (s, 2.84H, H_{21}), 2.01 (s, 0.19H, $H_{21'}$), 1.91 (s, 6H, H_r), 1.89 – 1.86 (s, s, 6H, H_a), 1.72 – 1.64 (m, 2H, H_{31}), 1.63 – 1.57 (m, 2H, H_{30}), 0.84 – 0.80 (m, 2H, H_v), 0.73 – 0.64 (m, 2H, H_{ee}), 0.44 – 0.33 (m, 4H, $H_{z,aa}$), 0.22 – 0.13 (m, 2H, H_y), 0.13 – 0.06 (m, 2H, H_{bb}), -0.22 – -0.31 (m, 2H, H_w), -0.32 – -0.43 (m, 6H, $H_{x,cc,dd}$).
^{13}C NMR (126 MHz, DMSO-*d*$_6$, 298 K) δ = 174.87, 174.73 ($C_{17,18}$), 174.35, 174.23 ($C_{17',18'}$), 170.89 (C_c), 170.77 (C_t), 170.15 (C_{22}), 165.41 (C_{43}), 158.00 (C_{37}), 150.16, 149.70, 149.67, 149.64, 149.60, 149.59, 149.55 ($C_{i,p}$), 148.59 (C_f), 146.81 (C_{33}), 136.64 (C_{26}), 135.62 (C_{25}), 131.33, 131.23 ($C_{39,4}$), 130.47, 130.21 ($C_{7,11}$), 130.08, 130.03 (C_{41}), 128.44, 128.03, 127.56, 127.32, 127.29, 127.16, 127.11, 127.06, 127.03, 127.00, 126.82 ($C_{12,5,6,9,14,g,l,n}$), 126.11, 125.94, 125.78 ($C_{1,2,8,10}$), 125.11 ($C_{3'}$), 124.99 (C_3), 124.10 (C_{15}), 123.33 (C_{16}), 122.36 (C_{34}), 122.28 (C_{13}), 121.64 (C_{40}), 119.24 (C_{42}), 115.01 (C_{38}), 114.43, 112.82, 112.45, 112.37, 112.32, 112.29, 112.22 ($C_{h,m,o}$), 91.42 (C_{27}), 91.28 ($C_{27'}$), 89.48 (C_{24}), 89.38 ($C_{24'}$), 70.79 (C_{29}), 68.52 (C_{28}), 68.17 ($C_{28'}$), 66.49 (C_{36}), 66.06 ($C_{e,u}$), 65.20 (C_{ff}), 64.21 (C_d), 61.89 ($C_{23'}$), 61.79 (C_{23}), 57.30 (C_s), 56.92 (C_b), 54.93, 54.89, 54.85, 54.82, 54.77, 54.71 ($C_{j,q}$), 48.82 (C_{35}), 48.59 (C_{20}), 47.81 (C_{19}), 30.30, 30.26, 30.23, 30.19, 29.83, 29.66, 29.61, 29.51, 29.44, 29.02, 28.89, 28.84, 28.77, 28.63, 28.48, 28.24, 27.97, 27.87, 27.70 ($C_{a,r,k,v,ee,x\text{-}cc,30}$), 25.64 ($C_{31}$), 24.76 ($C_{32}$), 23.82 ($C_{w,dd}$), 20.60 ($C_{21}$).
HRMS-ESI (+): 2015.7107 $[M+H]^+$, calculated for $C_{109}H_{124}Br_2N_4O_{23}H^+$: 2015.7096.

4.9.5 Synthesis of Control Compound, S42

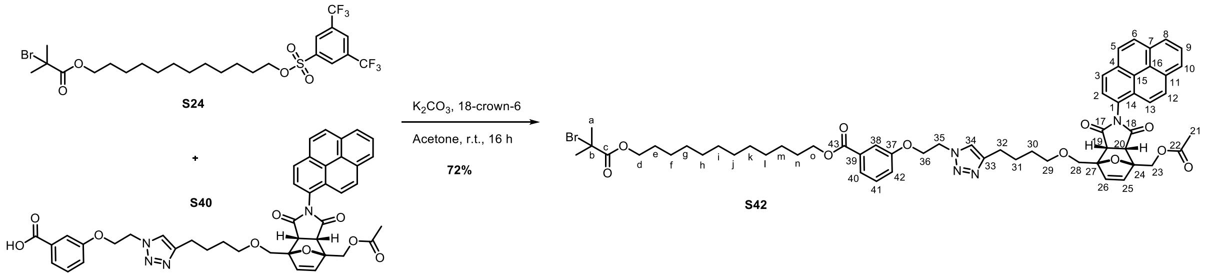

To a solution of **S40** (5 mg, 6.4 µmol, 1.0 eq.) in acetone (1 mL) was added K_2CO_3 (0.9 mg, 6.4 µmol, 1.0 eq.) and 18-crown-6 (1.7 mg, 6.4 µmol, 1.0 eq.). The mixture was stirred for 2 h at room temperature. **S24** (4 mg, 6.4 µmol, 1.0 eq.) was added and the mixture stirred for a further 16 h at room temperature. The solution was filtered, and the filtrate concentrated under vacuum. The residue was purified by preparative TLC (500 µm, DCM/MeOH, 40/1, eluted twice) to yield **S42** as a white powder (5 mg, 4.6 µmol, 72% yield).

S42 is observable as two interconverting isomers by NMR spectroscopy because of the slow rotation of the C_1-N bond. When distinguishable, these two isomers are denoted as X_x and X_x'. Additionally, 4% of the mixture is not loaded with the N-(1-pyrenyl)maleimide unit and exists as the furan derivative, which is denoted as X_x'' if distinguishable.

^{1}H NMR (400 MHz, Acetone-d_6, 298 K) δ = 8.38 – 8.30 (m, 3H, $H_{3,8,10}$), 8.29 – 8.17 (m, 3H, $H_{12,5,6}$), 8.11 (m, 1H, H_9), 8.04 (d, J = 9.2 Hz, 0.63H, H_{13}), 7.91 (d, J = 9.3 Hz, 0.34H, $H_{13'}$), 7.87 (d, J = 8.1 Hz, 0.33H, $H_{2'}$), 7.83 (s, 1H, H_{34}), 7.74 (d, J = 8.1 Hz, 0.63H, H_2), 7.60 (dt, J = 7.7, 1.2 Hz, 1H, H_{40}), 7.53 – 7.49 (m, 1H, H_{38}), 7.38 (t, J = 8.0 Hz, 1H, H_{41}), 7.19 – 7.12 (m, 1H, H_{42}), 7.02 (s, 0.69H, $H_{25',26'}$), 6.80 (s, 1.28H, $H_{25,26}$), 6.41 (d, J = 3.2 Hz, 0.04H, $H_{25''}$), 6.33 (d, J = 3.2 Hz, 0.04H, $H_{26''}$), 5.00 (s, 0.09H, $H_{23''}$), 4.93 – 4.85 (m, 1H, H_{23}), 4.78 (t, J = 5.1 Hz, 2H, H_{35}), 4.65 – 4.57 (m, 1H, H_{23}), 4.53 – 4.44 (m, 2H, H_{36}), 4.39 (s, 0.09H, $H_{28''}$), 4.31 – 4.25 (m, 2H, H_o), 4.20 – 3.94 (m, 6H, $H_{d,28,19,20}$), 3.68 – 3.61 (m, 2H, H_{29}), 2.72 (t, J = 7.4 Hz, 2H, H_{32}), 2.08 – 2.07 (s, s, 3H, H_{21}), 1.91 (s, 6H, H_a), 1.83 – 1.71 (m, 4H, $H_{31,n}$), 1.71 – 1.61 (m, 4H, $H_{30,e}$), 1.48 – 1.25 (m, 16H, $H_{f\text{-}m}$).

^{13}C NMR (101 MHz, Acetone-d_6, 298 K) δ = 175.56, 175.37 ($C_{17,18}$), 175.05, 174.86 ($C_{17',18'}$), 171.83 (C_c), 170.80 (C_{22}), 166.46 (C_{43}), 159.32 (C_{37}), 148.22 (C_{33}), 138.53 ($C_{26'}$), 137.65 (C_{26}), 137.50 ($C_{25'}$), 136.60 (C_{25}), 132.90 (C_{39}), 132.71 (C_4), 132.67 ($C_{4'}$), 131.96, 131.94, 131.67, 131.56 ($C_{7,11}$), 130.61 (C_{41}), 129.55 (C_{12}), 129.37, 129.32, 129.30 ($C_{12',5}$), 128.93 (C_{14}), 128.91 ($C_{14'}$), 127.99 (C_6), 127.51 (C_9), 127.50 ($C_{2'}$), 127.43 ($C_{1'}$), 127.33 (C_1), 126.94, 126.90, 126.75, 126.70 ($C_{8,10,2}$), 125.90 ($C_{3'}$), 125.81 (C_3), 125.70 (C_{15}), 125.63 ($C_{15'}$), 124.96 ($C_{16'}$), 124.94 (C_{16}), 123.02, 122.95, 122.89 ($C_{40,34,13}$), 120.15 (C_{42}), 115.94 (C_{38}), 92.91 (C_{27}), 92.77 ($C_{27'}$), 90.86 (C_{24}), 90.74 ($C_{24'}$), 72.09, 72.07 (C_{29}), 69.54 ($C_{28'}$), 69.43 (C_{28}), 67.71 (C_{36}), 66.51 (C_d), 65.70 (C_o), 62.98 ($C_{23'}$), 62.89 (C_{23}), 57.51 (C_b), 50.75 ($C_{20'}$), 49.96 (C_{35}), 49.89 (C_{20}), 49.78 ($C_{19'}$), 48.93 (C_{19}), 30.97 (C_a), 30.67 – 29.17 ($C_{g\text{-}l,n,30}$, overlapped with solvent peak), 29.08 (C_e), 26.90 (C_{31}), 26.71 (C_m), 26.50 (C_f), 25.95 (C_{32}), 20.70 (C_{21}).

HRMS-ESI (+): 1109.3888 $[M+Na]^+$, calculated for $C_{59}H_{67}BrN_4O_{11}Na^+$: 1109.3882.

4.9.6 Synthesis of Reference Compound, S43

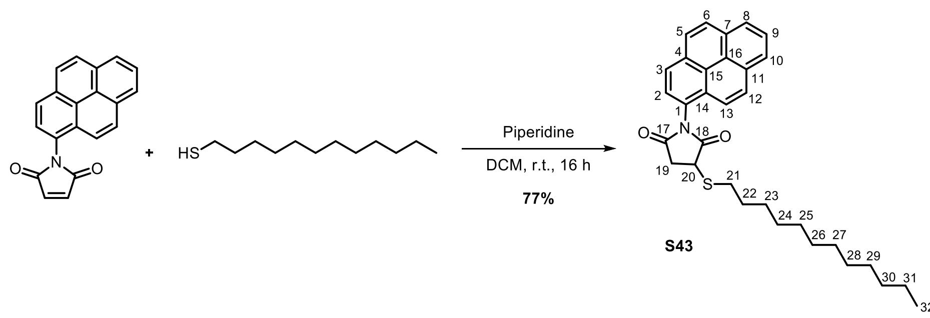

To a solution of N-(1-pyrenyl)maleimide (50 mg, 0.17 mmol, 1.0 eq.) and 1-dodecanethiol (102 mg, 0.50 mmol, 3.0 eq.) in DCM (1 mL) was added piperidine (57 mg, 0.67 mmol, 4.0 eq.). The mixture was stirred for 16 h at room temperature before being concentrated under vacuum. The crude material was purified by preparative TLC (2000 µm, PE/DCM/acetone, 14/2/1, eluted twice) to yield **S43** (65 mg, 0.13 mmol, 77% yield) as a yellow powder.

S43 is observable as two interconverting isomers by NMR spectroscopy because of the slow rotation of the C_1-N bond. When distinguishable, these two isomers are denoted as X_x and X_x'.

^{1}H NMR (400 MHz, Acetone-d_6, 298 K) δ = 8.43 – 8.30 (m, 3H, $H_{3,8,10}$), 8.30 – 8.17 (m, 3H, $H_{12,5,6}$), 8.16 – 8.08 (m, 1H, H_9), 8.08 – 8.00 (m, 1H, H_{13}), 7.97 – 7.88 (m, 1H, H_2), 4.43 (dd, J = 9.1, 3.9 Hz, 0.41H, $H_{20'}$), 4.27 (dd, J = 8.9, 3.2 Hz, 0.60H, H_{20}), 3.77 (dd, J = 18.4, 9.1 Hz, 0.43H, $H_{19'}$), 3.65 (dd, J = 18.5, 8.9 Hz, 0.62H, H_{19}), 3.14 – 2.86 (m, 3H, $H_{21,19}$), 1.85 – 1.64 (m, 2H, H_{22}), 1.55 – 1.42 (m, 2H, H_{23}), 1.40 – 1.19 (m, 16H, $H_{24\text{-}31}$), 0.91 – 0.81 (m, 3H, H_{32}).

^{13}C NMR (101 MHz, Acetone-d_6, 298 K) δ = 177.27 ($C_{17'}$), 177.13 (C_{17}), 175.41 (C_{18}), 175.37 ($C_{18'}$), 132.75 ($C_{4'}$), 132.70 (C_4), 131.96, 131.66, 131.62 ($C_{7,11}$), 129.61 (C_{12}), 129.35, 129.31, 129.28 ($C_{12',5}$), 128.89 ($C_{14'}$), 128.81 (C_{14}), 128.03 (C_6), 128.02 ($C_{6'}$), 127.68 ($C_{1'}$), 127.52 (C_9), 127.35 (C_1), 127.33 (C_2), 127.09 ($C_{2'}$), 126.99, 126.91, 126.81, 126.74 ($C_{8,10}$), 125.95 ($C_{3'}$), 125.90 (C_3), 125.81 ($C_{15'}$), 125.79 (C_{15}), 125.01 (C_{16}), 124.98 ($C_{16'}$), 123.07 ($C_{13'}$), 122.27 (C_{13}), 41.10 ($C_{20'}$), 40.82 (C_{20}), 37.63 ($C_{19'}$), 37.44 (C_{19}), 32.35 (C_{21}), 32.12 ($C_{21'}$), 30.69 – 29.01 ($C_{24\text{-}30}$, overlapped with solvent peak), 23.32 (C_{31}), 14.35 (C_{32}).

HRMS-ESI (+): 522.2442 $[M+Na]^+$, calculated for $C_{32}H_{37}NO_2SNa^+$: 522.2437.

4.9.7 Synthetic Route to Reference Compound, S45

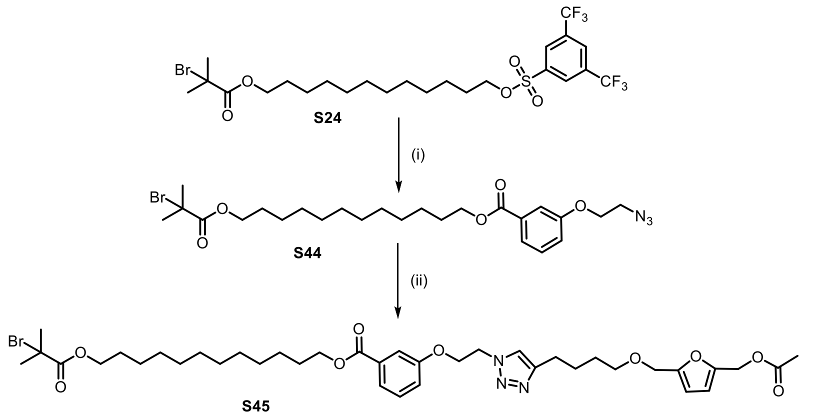

Figure S16. Synthetic route to **S45**. Conditions: (i) **S7**, K_2CO_3, 18-crown-6, acetone, r.t., 16 h, 73% yield; (ii) **S3**, CuBr, PMDETA, DCM, r.t., 16 h, 48% yield.

4.9.8 Synthesis of S44

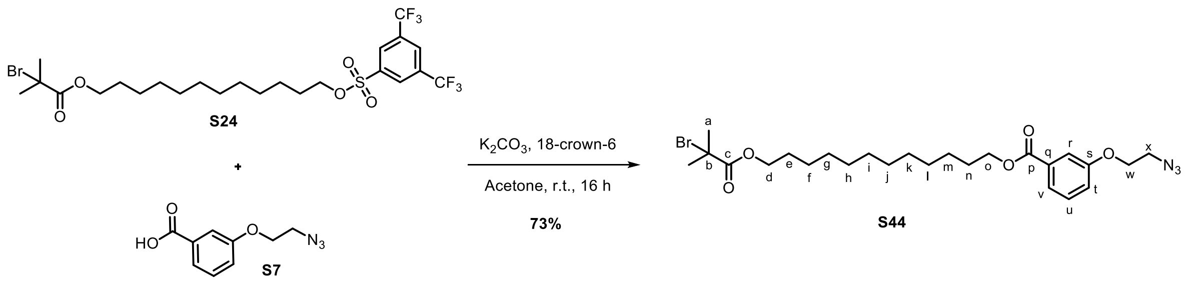

To a solution of **S7** (26 mg, 128 µmol, 1.0 eq.) in acetone (2 mL) was added K_2CO_3 (18 mg, 128 µmol, 1.0 eq.) and 18-crown-6 (34 mg, 128 µmol, 1.0 eq.). The mixture was stirred for 2 h at room temperature before **S24** (80 mg, 128 µmol, 1.0 eq.) was added. The mixture was stirred for 16 h at room temperature. The solution was filtered and the filtrate concentrated under vacuum. The residue was purified by preparative TLC (2000 µm, PE/Et_2O = 8/1) to yield **S44** as colorless oil (50 mg, 93 µmol, 73 % yield).

^{1}H NMR (500 MHz, Acetone-d$_6$, 298 K) δ = 7.64 (dt, *J* = 7.6, 1.3 Hz, 1H, H_v), 7.57 (dd, *J* = 2.7, 1.5 Hz, 1H, H_r), 7.45 (t, *J* = 8.0 Hz, 1H, H_u), 7.25 (ddd, *J* = 8.3, 2.7, 1.0 Hz, 1H, H_t), 4.33 – 4.27 (m, 4H, $H_{w,o}$), 4.16 (t, *J* = 6.5 Hz, 2H, H_d), 3.71 (t, *J* = 4.8 Hz, 2H, H_x), 1.92 (s, 6H, H_a), 1.82 – 1.72 (m, 2H, H_n), 1.71 – 1.64 (m, 2H, H_e), 1.50 – 1.44 (m, 2H, H_m), 1.44 – 1.26 (m, 14H, H_{f-l}).
^{13}C NMR (126 MHz, Acetone-d$_6$, 298 K) δ = 171.82 (C_c), 166.47 (C_p), 159.49 (C_s), 132.96 (C_q), 130.65 (C_u), 122.90 (C_v), 120.20 (C_t), 115.84 (C_r), 68.26 (C_w), 66.50 (C_d), 65.68 (C_o), 57.49 (C_b), 50.88 (C_x), 30.97 (C_a), 30.25, 30.22, 30.21, 30.06-29.60 (overlapped with solvent peaks, C_{g-l}), 29.43 (C_n), 29.09 (C_e), 26.74 (C_m), 26.51 (C_f).
HRMS-ESI (+): 562.1882 $[M+Na]^+$, calculated for $C_{25}H_{38}BrN_3O_5Na^+$: 562.1887.

4.9.9 Synthesis of S45

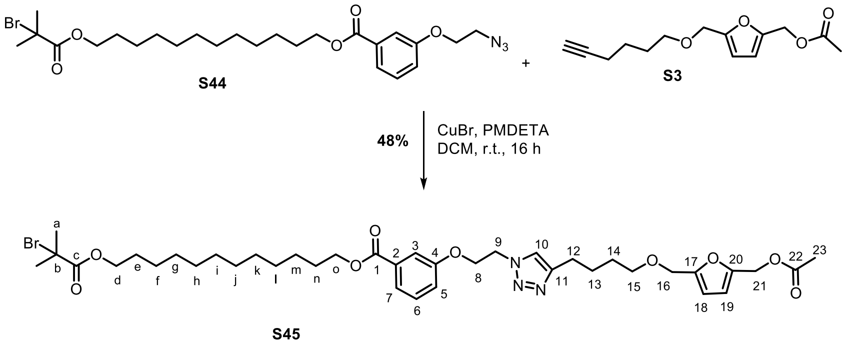

Three sealed 5 mL microwave vials (*A*, *B*, and *C*) were prepared as follows: *A* contained PMDETA (5 mg, 28 µmol, 1.5 eq.) in DCM (3 mL), *B* contained CuBr (4 mg, 30 µmol, 1.6 eq.), and *C* contained **S44** (10 mg, 19 µmol, 1.0 eq.) and **S3** (9 mg, 37 µmol, 2.0 eq.). Vials *B* and *C* were subjected to three N_2/vacuum cycles before use. The solution in vial *A* was degassed by bubbling with N_2 until ~1 mL DCM was left before being transferred to vial *B via* cannula; the resulting mixture was stirred until all the CuBr had dissolved. The CuBr/PMDETA solution in vial *B* was then transferred to vial *C via* cannula; the resulting reaction mixture was stirred for 16 h at room temperature. The reaction mixture was washed with aqueous EDTA solution (0.25 M, pH 7, 2 x 1 mL) and brine (2 x 1 mL). The organic layer was collected and dried over magnesium sulfate. The mixture was filtered before being concentrated under vacuum. The residue was purified by preparative TLC (500 µm, PE/acetone, 2/1, eluted twice) to yield **S45** as a white solid (7 mg, 9 µmol, 48% yield).

^{1}H NMR (500 MHz, DMSO-d_6, 298 K) δ = 7.92 (s, 1H, H_{10}), 7.54 (dt, *J* = 7.8, 1.3 Hz, 1H, H_7), 7.45 – 7.38 (m, 2H, $H_{3,6}$), 7.20 (ddd, *J* = 8.3, 2.7, 1.0 Hz, 1H, H_5), 6.45 (d, *J* = 3.2 Hz, 1H, H_{19}), 6.37 (d, *J* = 3.2 Hz, 1H, H_{18}), 4.99 (s, 2H, H_{21}), 4.71 (t, *J* = 5.1 Hz, 2H, H_9), 4.44 (t, *J* = 5.1 Hz, 2H, H_8), 4.34 (s, 2H, H_{16}), 4.24 (t, *J* = 6.6 Hz, 2H, H_o), 4.11 (t, *J* = 6.4 Hz, 2H, H_d), 3.41 (t, *J* = 6.4 Hz, 2H, H_{15}), 2.59 (t, *J* = 7.4 Hz, 2H, H_{12}), 2.01 (s, 3H, H_{23}), 1.88 (s, 6H, H_a), 1.71 – 1.65 (m, 2H, H_n), 1.63 – 1.56 (m, 4H, $H_{13,e}$), 1.55 – 1.48 (m, 2H, H_{14}), 1.41 – 1.34 (m, 2H, H_m), 1.34 – 1.29 (m, 4H, $H_{f,l}$), 1.24 (d, *J* = 5.1 Hz, 10H, $H_{g\text{-}k}$).
^{13}C NMR (126 MHz, DMSO-d_6, 298 K) δ = 170.77 (C_c), 169.94 (C_{22}), 165.42 (C_1), 157.98 (C_4), 152.64 (C_{17}), 149.28 (C_{20}), 146.75 (C_{11}), 131.27 (C_2), 130.02 (C_6), 122.35 (C_{10}), 121.81 (C_7), 119.59 (C_5), 114.76 (C_3), 111.44 (C_{19}), 110.04 (C_{18}), 69.15 (C_{15}), 66.52 (C_8), 65.54 (C_d), 64.79 (C_o), 63.86 (C_{16}), 57.58 (C_{21}), 57.43 (C_b), 48.78 (C_9), 30.22 (C_a), 28.89, 28.84, 28.61 ($C_{h\text{-}l}$), 28.51, 28.48 ($C_{14,g}$), 28.09 (C_n), 27.77 (C_e), 25.62 (C_{13}), 25.42 (C_m), 25.18 (C_f), 24.68 (C_{12}), 20.57 (C_{23}).
HRMS-ESI (+): 812.3130 $[M+Na]^+$, calculated for $C_{39}H_{56}BrN_3O_9Na^+$: 812.3092.

4.10 Synthesis of Alternative-Cargo Compounds – Trityl Cargo Release

4.10.1 Synthetic Route to Trityl-Cargo-Containing Rotaxane

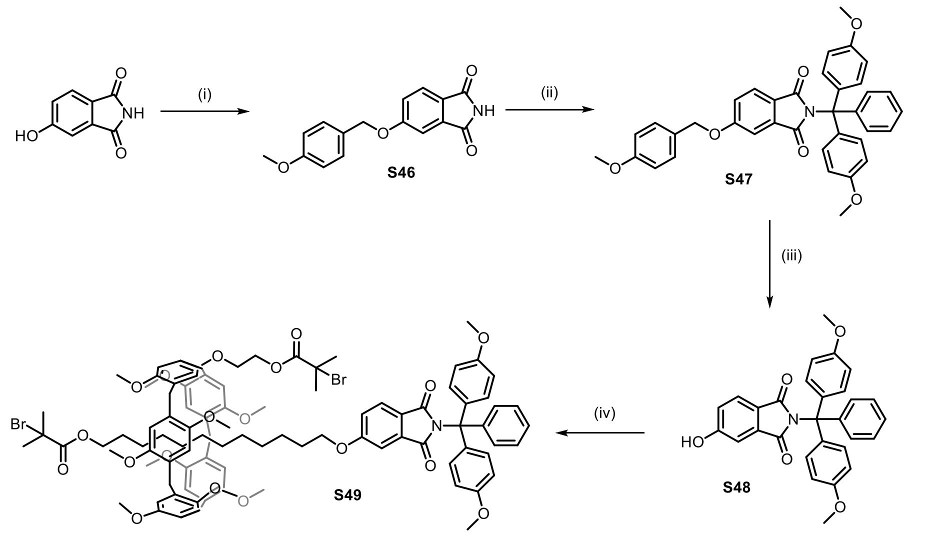

Figure S17. Synthetic route to **S49**. Conditions: (i) 4-Methoxybenzyl chloride, K_2CO_3, TBAI, acetone, 55 °C, 16 h, 63% yield; (ii) 4,4'-Dimethoxytrityl chloride, Et_3N, DCM, r.t., 2 h, 97% yield; (iii) DDQ, DCM, H_2O, r.t., 16 h, 38% yield; (iv) **7***trans*, K_2CO_3, 18-crown-6, acetone, r.t., 16 h, 60% yield.

4.10.2 Synthesis of S46

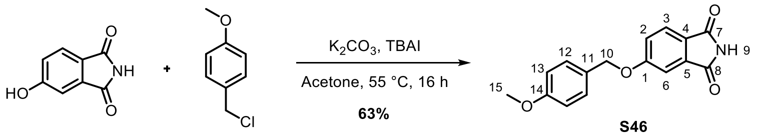

To a solution of 5-hydroxyisoindoline-1,3-dione (100 mg, 0.6 mmol, 1.0 eq.) in acetone (2 mL) was added K_2CO_3 (85 mg, 0.6 mmol, 1.0 eq.). The mixture was stirred for 2 h at room temperature. 4-Methoxybenzyl chloride (96 mg, 0.6 mmol, 1.0 eq.) and TBAI (5 mg, 0.01 mmol, 0.02 eq.) were added and the mixture stirred for 16 h at 55 °C. The solution was allowed to cool to room temperature before being filtered. The filtrate was concentrated under vacuum. The residue was purified by column chromatography (SiO_2, PE/acetone, 8/1) to yield **S46** as a white powder (110 mg, 0.4 mmol, 63% yield).

^{1}H NMR (400 MHz, Acetone-d_6, 298 K) δ = 9.91 (b, 1H, H_9), 7.75 (dd, J = 7.8, 1.1 Hz, 1H, H_3), 7.49 – 7.43 (m, 2H, H_{12}), 7.41 – 7.35 (m, 2H, $H_{6,2}$), 7.01 – 6.94 (m, 2H, H_{13}), 5.25 (s, 2H, H_{10}), 3.81 (s, 3H, H_{15}).
^{13}C NMR (101 MHz, Acetone-d_6, 298 K) δ = 168.95 ($C_{7,8}$), 164.88 (C_1), 160.77 (C_{14}), 136.54 (C_5), 130.48 (C_{12}), 129.12 (C_{11}), 125.94 (C_4), 125.55 (C_3), 121.57 (C_2), 114.81 (C_{13}), 109.36 (C_6), 71.24 (C_{10}), 55.57 (C_{15}).
HRMS-ESI (+): 306.0738 $[M+Na]^+$, calculated for $C_{16}H_{13}NO_4Na^+$: 306.0737.

4.10.3 Synthesis of S47

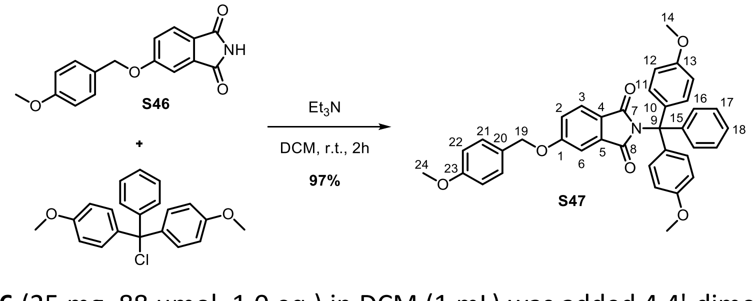

To a solution of **S46** (25 mg, 88 µmol, 1.0 eq.) in DCM (1 mL) was added 4,4'-dimethoxytrityl chloride (45 mg, 132 µmol, 1.5 eq.) and Et_3N (37 µL, 265 µmol, 3.0 eq.). The mixture was stirred at room temperature for 2 h before being concentrated under vacuum. The residue was purified by preparative TLC (2000 µm, PE/EtOAc, 3/1) to yield **S47** as a faint yellow solid (50 mg, 85 µmol, 97% yield).

^{1}H NMR (400 MHz, Acetone-d_6, 298 K) δ = 7.64 (d, J = 8.2 Hz, 1H, H_3), 7.51 – 7.46 (m, 2H, H_{16}), 7.46 – 7.39 (m, 6H, $H_{11,21}$), 7.31 (dd, J = 8.3, 2.3 Hz, 1H, H_2), 7.27 (d, J = 2.2 Hz, 1H, H_6), 7.25 – 7.19 (m, 2H, H_{17}), 7.15 – 7.08 (m, 1H, H_{18}), 6.98 – 6.93 (m, 2H, H_{22}), 6.84 – 6.76 (m, 4H, H_{12}), 5.21 (s, 2H, H_{19}), 3.79 (s, 3H, H_{24}), 3.74 (s, 6H, H_{14}).
^{13}C NMR (101 MHz, Acetone-d_6, 298 K) δ = 168.48 (C_8), 168.37 (C_7), 164.89 (C_1), 160.72 (C_{23}), 158.95 (C_{13}), 145.19 (C_{15}), 136.40 (C_{10}), 135.67 (C_5), 130.90 (C_{11}), 130.36 (C_{21}), 129.06 (C_{20}), 129.02 (C_{16}), 128.14 (C_{17}), 126.67 (C_{18}), 125.31 (C_3), 125.20 (C_4), 122.05 (C_2), 114.80 (C_{22}), 113.47 (C_{12}), 108.75 (C_6), 73.47 (C_9), 71.15 (C_{19}), 55.56 (C_{24}), 55.39 (C_{14}).
HRMS-ESI (+): 608.2045 $[M+Na]^+$, calculated for $C_{37}H_{31}NO_6Na^+$: 608.2044.

4.10.4 Synthesis of S48

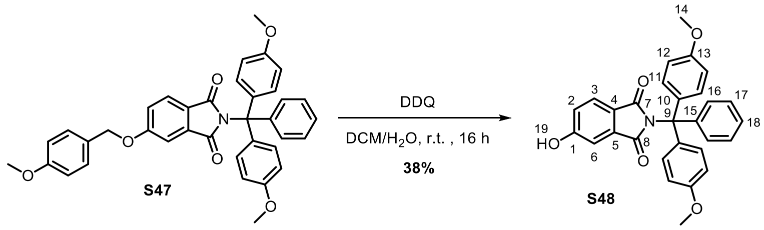

To a solution of **S47** (30 mg, 51 µmol, 1.0 eq.) in DCM (2 mL) and H_2O (0.1 mL) was added DDQ (58 mg, 256 µmol, 5.0 eq.). The mixture was stirred at room temperature for 16 h before being dried over magnesium sulfate and concentrated under vacuum. The residue was purified by preparative TLC (2000 µm, PE/EtOAc, 3/1, eluted twice) to yield **S48** as a faint yellow solid (9 mg, 19 µmol, 38% yield).

^{1}H NMR (400 MHz, Acetone-d_6, 298 K) δ = 7.59 (dd, J = 8.2, 0.5 Hz, 1H, H_3), 7.49 – 7.46 (m, 2H, H_{16}), 7.46 – 7.41 (m, 4H, H_{11}), 7.26 – 7.20 (m, 2H, H_{17}), 7.17 (dd, J = 8.2, 2.2 Hz, 1H, H_2), 7.15 – 7.09 (m, 2H, $H_{18,6}$), 6.83 – 6.77 (m, 4H, H_{12}), 3.75 (s, 6H, H_{14}).
^{13}C NMR (101 MHz, Acetone-d_6, 298 K) δ = 168.55 (C_8), 168.49 (C_7), 163.91 (C_1), 158.95 (C_{13}), 145.26 (C_{15}), 136.47 (C_{10}), 135.95 (C_5), 130.92 (C_{11}), 129.04 (C_{16}), 128.13 (C_{17}), 126.65 (C_{18}), 125.65 (C_3), 124.21 (C_4), 121.55 (C_2), 113.46 (C_{12}), 109.86 (C_6), 73.37 (C_9), 55.39 (C_{14}).
HRMS-ESI (+): 488.1472 $[M+Na]^+$, calculated for $C_{29}H_{23}NO_5Na^+$: 488.1468.

4.10.5 Synthesis of S49

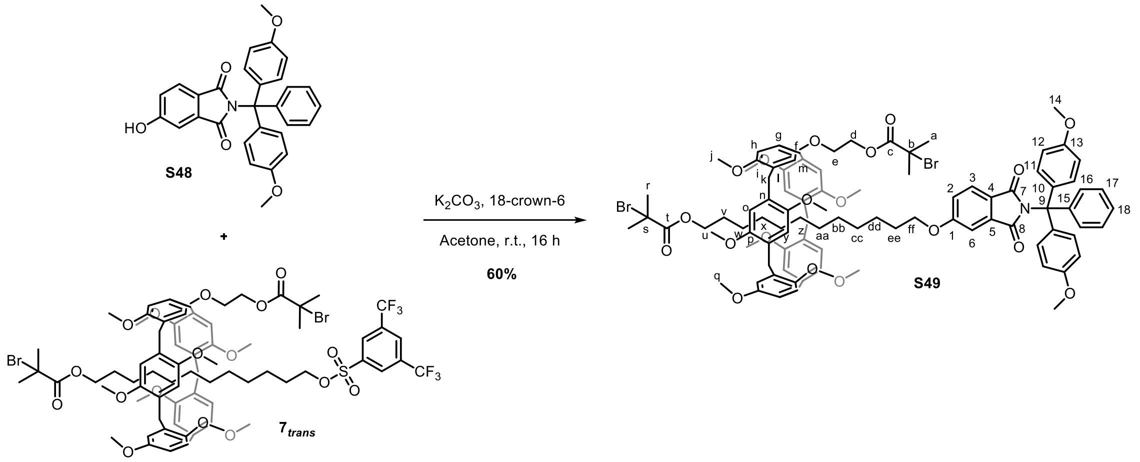

To a solution of **S48** (2.7 mg, 5.8 µmol, 1.0 eq.) in acetone (1 mL) was added K_2CO_3 (0.8 mg, 5.8 µmol, 1.0 eq.) and 18-crown-6 (1.5 mg, 5.8 µmol, 1.0 eq.). The mixture was stirred for 2 h at room temperature. **7*trans*** (9 mg, 5.8 µmol, 1.0 eq.) was added and the mixture stirred for a further 16 h at room temperature. The solution was filtered, and the filtrate concentrated under vacuum. The residue was purified by preparative TLC (500 µm, PE/Acetone, 4/1, eluted twice) to yield **S49** as a white powder (6 mg, 3.5 µmol, 60% yield).

^{1}H NMR (500 MHz, DMSO-d_6, 298 K) δ = 7.71 (d, *J* = 8.2 Hz, 1H, H_3), 7.55 – 7.52 (m, 2H, H_{16}), 7.51 – 7.47 (m, 4H, H_{11}), 7.29 – 7.24 (m, 3H, $H_{17,6}$), 7.22 (dd, *J* = 8.2, 2.3 Hz, 1H, H_2), 7.18 – 7.12 (m, 1H, H_{18}), 7.06 – 6.89 (m, 10H, $H_{h,m,o}$), 6.87 – 6.82 (m, 4H, H_{12}), 4.41 (ddd, *J* = 11.9, 9.1, 2.5 Hz, 1H, H_d), 4.22 (dt, *J* = 10.8, 2.9 Hz, 1H, H_e), 4.17 (ddd, *J* = 11.9, 3.6, 2.4 Hz, 1H, H_d), 4.06 – 4.00 (m, 3H, $H_{e,u}$), 3.83 – 3.64 (m, 43H, $H_{j,k,q,14}$), 2.96 – 2.84 (m, 2H, H_{ff}), 1.97 (s, 7H, H_r), 1.94 – 1.90 (s, s, 6H, H_a), 1.40 – 1.33 (m, 3H, H_v), 1.02 – 0.95 (m, 2H, H_z), 0.93 – 0.80 (m, 4H, $H_{y,aa}$), 0.74 – 0.67 (m, 2H, H_w), 0.67 – 0.59 (m, 2H, H_x), 0.40 – 0.30 (m, 2H, H_{bb}), -0.15 – -0.27 (m, 2H, H_{ee}), -0.52 – -0.64 (m, 2H, H_{cc}), -1.21 – -1.32 (m, 2H, H_{dd}).

^{13}C NMR (126 MHz, DMSO-d_6, 298 K) δ = 172.04 (C_c), 171.89 (C_t), 168.97 (C_8), 168.51 (C_7), 165.51 (C_1), 159.02 (C_{13}), 151.97, 151.32, 151.28, 151.26, 151.20, 151.18, 151.14, 151.12 ($C_{i,p}$), 150.06 (C_f), 145.29 (C_{15}), 136.49 (C_{10}), 135.81 (C_5), 130.95 (C_{11}), 130.94 ($C_{11'}$), 130.20 , 129.16, 129.13, 129.05, 128.94, 128.93, 128.86, 128.83, 128.80, 128.72, 128.64 ($C_{g,l,n,16}$), 128.21 (C_{17}), 126.76 (C_{18}), 125.36 (C_3), 124.73 (C_4), 121.93 (C_2), 116.85, 114.56, 114.35, 114.13, 113.90, 113.76, 113.70, 113.62, 113.59 ($C_{h,m,o}$), 113.53 (C_{12}), 107.82 (C_6), 73.50 (C_9), 70.44 (C_{ff}), 68.19 (C_e), 66.91 (C_u), 65.44 (C_d), 57.55 (C_s), 57.09 (C_b), 56.23, 56.18, 56.00, 55.80, 55.79, 55.76, 55.70, 55.44 ($C_{j,q,14}$), 31.55 (C_{aa}), 31.29 (C_z), 31.20 (C_{bb}), 31.06, 31.05, 31.02, 31.00 ($C_{a,r}$), 30.91 (C_y), 30.48 – 29.31 ($C_{k,x,cc}$, overlapped with residual solvent peak), 29.15 (C_v), 28.60 (C_{ee}), 25.97 (C_w), 23.98 (C_{dd}).

HRMS-ESI (+): 1748.5906 $[M+Na]^+$, calculated for $C_{95}H_{109}Br_2NO_{19}Na^+$: 1748.5853.

4.10.6 Synthetic Route to Trityl-Cargo Control Compound

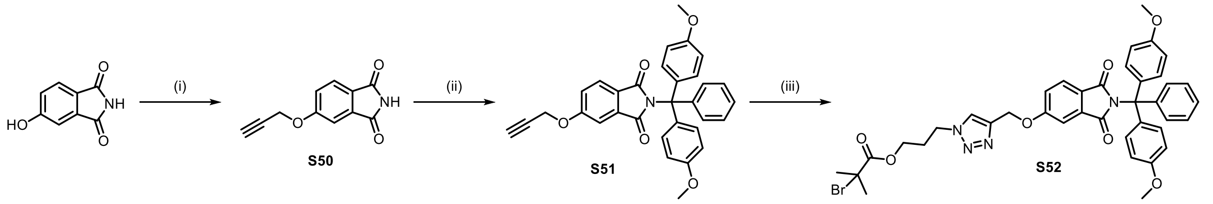

Figure 18. Synthetic route to **S52**. Conditions: (i) Propargyl bromide, K_2CO_3, MeCN, 60 °C, 16 h, 18% yield; (ii) 4,4'-Dimethoxytrityl chloride, Et_3N, DCM, r.t., 2 h, 72% yield; (iii) **S53**, CuBr, PMDETA, DCM, r.t., 2 h, 67% yield.

4.10.7 Synthesis of S50

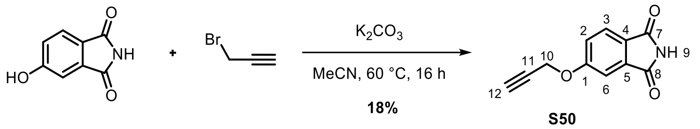

To a solution of 5-hydroxyisoindoline-1,3-dione (400 mg, 2.4 mmol, 1.0 eq.) and propargyl bromide (80 % in toluene, 325 µL, 2.9 mmol, 1.2 eq.) in acetonitrile (5 mL) was added K_2CO_3 (406 g, 2.9 mmol, 1.2 eq.). The mixture was heated to 60 °C for 16 h. The mixture was allowed to cool to room temperature before being filtered. The filtrate was concentrated under vacuum. The residue was purified by flash column chromatography (SiO_2, PE/acetone, 5/1) to yield **S50** as a white powder (90 mg, 0.4 mmol, 18% yield).

^{1}H NMR (400 MHz, Acetone-d_6, 298 K) δ 7.79 (d, J = 8.3 Hz, 1H, H_{10}), 7.42 (d, J = 2.2 Hz, 1H, H_5), 7.39 (dd, J = 8.2, 2.4 Hz, 1H, H_{11}), 5.03 (d, J = 2.4 Hz, 2H, H_3), 3.20 (t, J = 2.4 Hz, 1H, H_1).
^{13}C NMR (101 MHz, Acetone-d_6, 298 K) δ 168.86 ($C_{7,8}$), 163.52 (C_4), 136.46 (C_6), 126.64 (C_9), 125.56 (C_{10}), 121.70 (C_{11}), 109.41 (C_5), 78.72 (C_2), 78.10 (C_1), 57.15 (C_3).
HRMS-ESI (+): 202.0493 $[M+H]^+$, calculated for $C_{11}H_7NO_3H^+$: 202.0499.

4.10.8 Synthesis of S51

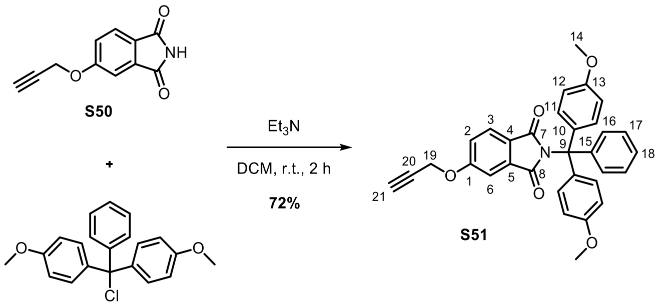

To a solution of **S50** (5 mg, 25 µmol, 1.0 eq.) in DCM (1 mL) was added 4,4'-dimethoxytrityl chloride (10 mg, 30 µmol, 1.2 eq.) and Et_3N (7 µL, 50 µmol, 2.0 eq.). The mixture was stirred at room temperature for 2 h before being concentrated under vacuum. The residue was purified by preparative TLC (500 µm, PE/Et_2O, 1/1) to yield **S51** as a faint yellow solid (9 mg, 18 µmol, 72% yield).

^{1}H NMR (500 MHz, Acetone-d_6, 298 K) δ = 7.69 (dd, J = 8.0, 0.8 Hz, 1H, H_3), 7.51 – 7.47 (m, 2H, H_{16}), 7.47 – 7.42 (m, 4H, H_{11}), 7.35 – 7.30 (m, 2H, $H_{2,6}$), 7.26 – 7.20 (m, 2H, H_{17}), 7.15 – 7.10 (m, 1H, H_{18}), 6.83 – 6.78 (m, 4H, H_{12}), 4.99 (d, J = 2.4 Hz, 2H, H_{19}), 3.75 (s, 6H, H_{14}), 3.19 (t, J = 2.4 Hz, 1H, H_{21}).
^{13}C NMR (126 MHz, Acetone-d_6, 298 K) δ = 168.35 (C_8), 168.29 (C_7), 163.58 (C_1), 158.98 (C_{13}), 145.16 (C_{15}), 136.37 (C_{10}), 135.61 (C_5), 130.91 (C_{11}), 129.03 (C_{16}), 128.16 (C_{17}), 126.70 (C_{18}), 125.92 (C_4), 125.34 (C_3), 122.17 (C_2), 113.48 (C_{12}), 108.75 (C_6), 78.71 (C_{20}), 78.13 (C_{21}), 73.53 (C_9), 57.11 (C_{19}), 55.39 (C_{14}).
HRMS-ESI (+): 526.1620 $[M+Na]^+$, calculated for $C_{32}H_{25}NO_5Na^+$: 526.1625.

4.10.9 Synthesis of S52

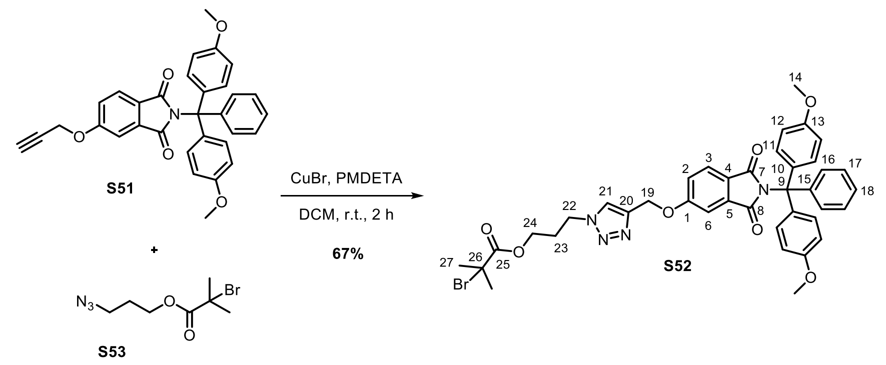

Three sealed 5 mL microwave vials (*A*, *B*, and *C*) were prepared as follows: *A* contained PMDETA (3 mg, 18 µmol, 1.5 eq.) in DCM (3 mL), *B* contained CuBr (3 mg, 19 µmol, 1.6 eq.), and *C* contained **S51** (6 mg, 12 µmol, 1.0 eq.) and **S53** (3 mg, 12 µmol, 1.0 eq.). Vials *B* and *C* were subjected to three N_2/vacuum cycles before use. The solution in vial *A* was degassed by bubbling with N_2 until ~1 mL DCM was left before being transferred to vial *B via* cannula; the resulting mixture was stirred until all the CuBr had dissolved. The CuBr/PMDETA solution in vial *B* was then transferred to vial *C via* cannula; the resulting reaction mixture was stirred for 2 h at room temperature. The reaction mixture was washed with aqueous EDTA solution (0.25 M, pH 7, 2 x 1 mL) and brine (2 x 1 mL). The organic layer was collected and dried over magnesium sulfate. The mixture was filtered before being concentrated under vacuum. The residue was purified by preparative TLC (500 µm, PE/acetone, 3/1, eluted twice) to yield **S52** as a white powder (6 mg, 8 µmol, 67% yield).

^{1}H NMR (400 MHz, Acetone-d_6, 298 K) δ = 8.15 (s, 1H, H_{21}), 7.66 (d, *J* = 8.4 Hz, 1H, H_3), 7.51 – 7.46 (m, 2H, H_{16}), 7.46 – 7.41 (m, 4H, H_{11}), 7.39 (d, *J* = 2.1 Hz, 1H, H_6), 7.35 (dd, *J* = 8.2, 2.3 Hz, 1H, H_2), 7.26 – 7.20 (m, 2H, H_{17}), 7.16 – 7.09 (m, 1H, H_{18}), 6.84 – 6.77 (m, 4H, H_{12}), 5.37 (s, 2H, H_{19}), 4.60 (t, *J* = 6.9 Hz, 2H, H_{22}), 4.21 (t, *J* = 6.1 Hz, 2H, H_{24}), 3.75 (s, 6H, H_{14}), 2.38 – 2.30 (m, 2H, H_{23}), 1.92 (s, 6H, H_{27}).
^{13}C NMR (101 MHz, Acetone-d_6, 298 K) δ = 171.74 (C_{25}), 168.41 (C_8), 168.35 (C_7), 164.54 (C_1), 158.98 (C_{13}), 145.20 (C_{15}), 143.38 (C_{20}), 136.41 (C_{10}), 135.71 (C_5), 130.92 (C_{11}), 129.04 (C_{16}), 128.15 (C_{17}), 126.69 (C_{18}), 125.48 (C_4), 125.34 (C_3), 125.20 (C_{21}), 121.93 (C_2), 113.49 (C_{12}), 108.93 (C_6), 73.50 (C_9), 63.47 (C_{24}), 63.20 (C_{19}), 57.37 (C_{26}), 55.40 (C_{14}), 47.61 (C_{22}), 30.89 (C_{27}), 30.53 – 29.18 (C_{23}, overlapped with residual solvent peak).
HRMS-ESI (+): 775.1729 $[M+Na]^-$, calculated for $C_{39}H_{37}BrN_4O_7Na^+$: 775.1738.

4.10.10 Synthesis of Reference Compound, S54

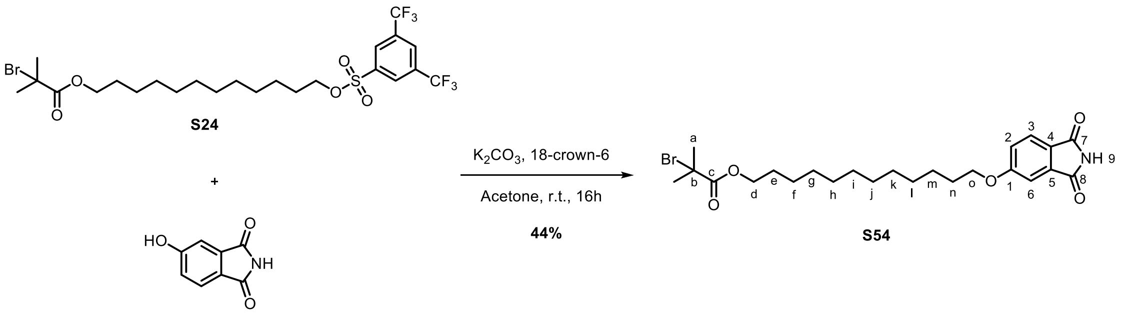

To a solution of 5-hydroxyisoindoline-1,3-dione (21 mg, 128 µmol, 2.0 eq.) in acetone (1 mL) was added K_2CO_3 (11 mg, 77 µmol, 1.2 eq.) and 18-crown-6 (17 mg, 64 µmol, 1.0 eq.). The mixture was stirred for 2 h at room temperature. **S24** (40 mg, 64 µmol, 1.0 eq.) was added and the mixture stirred for a further 16 h at room temperature. The solution was filtered, and the filtrate concentrated under vacuum. The residue was purified by preparative TLC (2000 µm, PE/Et_2O, 2/1, eluted twice) to yield **S54** as a white powder (14 mg, 28 µmol, 44% yield).

^{1}H NMR (500 MHz, Acetone-d_6, 298 K) δ = 9.91 (s, 1H, H_9), 7.75 – 7.72 (m, 1H, H_3), 7.32 – 7.29 (m, 2H, $H_{2,6}$), 4.20 (t, J = 6.5 Hz, 2H, H_o), 4.16 (t, J = 6.5 Hz, 2H, H_d), 1.92 (s, 6H, H_a), 1.88 – 1.80 (m, 2H, H_n), 1.72 – 1.63 (m, 2H, H_e), 1.55 – 1.47 (m, 2H, H_m), 1.45 – 1.30 (m, 14H, $H_{f\text{-}l}$).
^{13}C NMR (126 MHz, Acetone-d_6, 298 K) δ = 171.82 (C_c), 169.06, 169.02 ($C_{7,8}$), 165.23 (C_1), 136.59 (C_5), 125.68 (C_4), 125.53 (C_3), 121.12 (C_2), 108.90 (C_6), 69.73 (C_o), 66.50 (C_d), 57.49 (C_b), 30.97 (C_a), 30.39 – 30.03 ($C_{g\text{-}l}$, overlapped with solvent peak), 29.72 (C_n), 29.09 (C_e), 26.62 (C_m), 26.51 (C_f).
HRMS-ESI (+): 496.1686 $[M+H]^+$, calculated for $C_{24}H_{34}BrNO_5H^+$: 496.1693.

5 Synthesis of Polymers

5.1 Representative Procedure for SET-LRP of Methyl Acrylate Using Mechanophore Initiators

Methyl acrylate was filtered through basic alumina to remove the inhibitor prior to use. A stock catalytic solution of Me_6TREN (16 µL, 0.060 mmol) and $CuBr_2$ (5.6 mg, 0.025 mmol) in dry DMSO (1 mL) was prepared. To a 5 mL microwave vial was added the appropriate initiator compound along with catalytic solution, methyl acylate and dry DMSO. This solution was degassed by bubbling with N_2 for 10 min. A Cu(0) wire wrapped around a stirrer bar, having been cleaned in 12 N HCl for 10 min, was added to the reaction mixture. The reaction mixture was degassed for a further 2 min before being allowed to stir for 15 - 40 min (until the extent of polymerization, as determined approximately by the increasing viscosity of the solution, was deemed acceptable). The solution was added dropwise to a solution of vigorously stirred methanol; the precipitated polymer was recovered and dried under vacuum for two days to yield a white material. Molecular weight and polydispersity indices were determined using an analytical SEC that had been calibrated with polystyrene standards.

5.2 Synthesis of Control Polymers

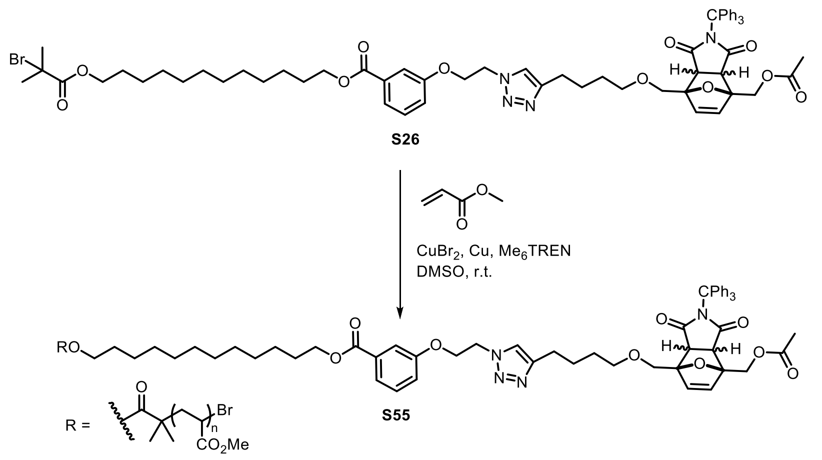

5.2.1 Synthesis of Polymer $S55_{exo\text{-}112}$

Synthesis followed the representative procedure. **S26*exo*** (1.1 mg, 1.0 µmol, 1.0 eq.), 8 µL of catalytic solution (Me_6TREN: 0.47 µmol, 0.5 eq.; $CuBr_2$: 0.19 µmol, 0.2 eq.), methyl acrylate (170 µL, 2.0 mmol, 2000.0 eq.), Cu (0) wire (~3 cm, ~30 mg, 0.5 mmol, ~500.0 eq.) and dry DMSO (170 µL) were used in the reaction to yield polymer **S55*exo*-112** (62 mg, M_n = 112 kDa; $Đ$ = 1.25).

5.2.2 Synthesis of Polymer $S55_{endo\text{-}65}$

Synthesis followed the representative procedure. **S26*endo*** (1.1 mg, 1.0 µmol, 1.0 eq.), 8 µL of catalytic solution (Me_6TREN: 0.47 µmol, 0.5 eq.; $CuBr_2$: 0.19 µmol, 0.2 eq.), methyl acrylate (170 µL, 2.0 mmol, 2000.0 eq.), Cu (0) wire (~3 cm, ~30 mg, 0.5 mmol, ~500.0 eq.) and dry DMSO (170 µL) were used in the reaction to yield polymer **S55*endo*-65** (35 mg, M_n = 65 kDa; $Đ$ = 1.20).

5.3 Synthesis of 1-Cargo Polymers

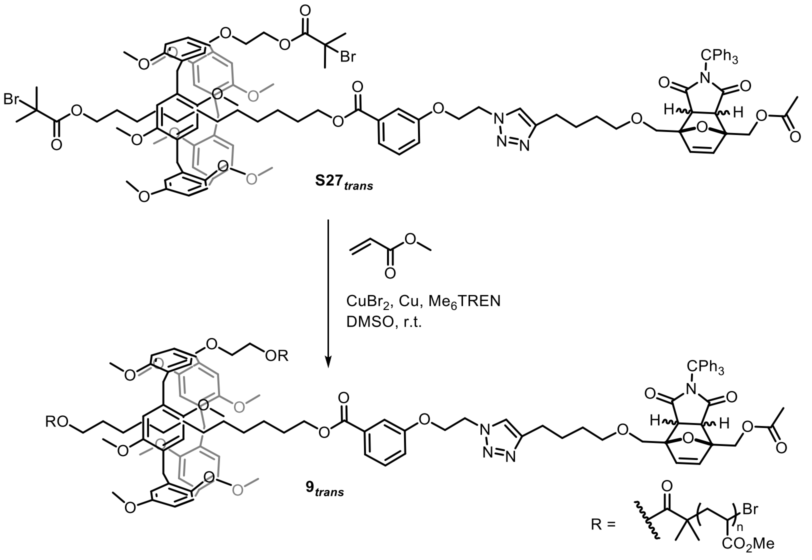

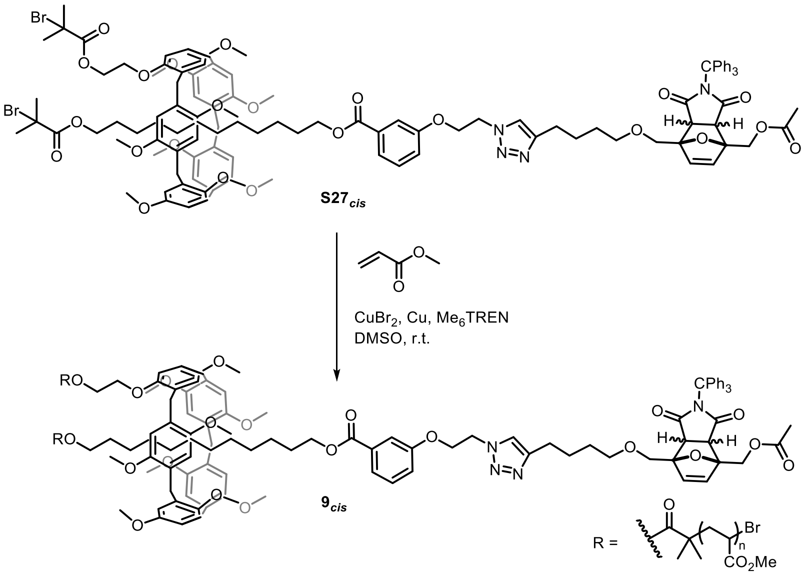

5.3.1 Synthesis of Polymer **9**$_{trans/exo\text{-}109}$

Synthesis followed the representative procedure. **S27**$_{trans/exo}$ (2.0 mg, 1.0 µmol, 1.0 eq.), 8 µL of catalytic solution (Me_6TREN: 0.47 µmol, 0.5 eq.; $CuBr_2$: 0.19 µmol, 0.2 eq.), methyl acrylate (170 µL, 2.0 mmol, 2000.0 eq.), Cu (0) wire (~3 cm, ~30 mg, 0.5 mmol, ~500.0 eq.) and dry DMSO (170 µL) were used in the reaction to yield polymer **9**$_{trans/exo\text{-}109}$ (52 mg, M_n = 109 kDa; $Đ$ = 1.12).

5.3.2 Synthesis of Polymer **9**$_{trans/exo\text{-}114}$

Synthesis followed the representative procedure. **S27**$_{trans/exo}$ (2.0 mg, 1.0 µmol, 1.0 eq.), 8 µL of catalytic solution (Me_6TREN: 0.47 µmol, 0.5 eq.; $CuBr_2$: 0.19 µmol, 0.2 eq.), methyl acrylate (170 µL, 2.0 mmol, 2000.0 eq.), Cu (0) wire (~3 cm, ~30 mg, 0.5 mmol, ~500.0 eq.) and dry DMSO (170 µL) were used in the reaction to yield polymer **9**$_{trans/exo\text{-}114}$ (60 mg, M_n = 114 kDa; $Đ$ = 1.17).

5.3.3 Synthesis of Polymer **9**$_{cis/exo\text{-}114}$

Synthesis followed the representative procedure. **S27**$_{cis/exo}$ (2.0 mg, 1.0 µmol, 1.0 eq.), 8 µL of catalytic

solution (Me_6TREN: 0.47 µmol, 0.5 eq.; $CuBr_2$: 0.19 µmol, 0.2 eq.), methyl acrylate (170 µL, 2.0 mmol, 2000.0 eq.), Cu (0) wire (~3 cm, ~30 mg, 0.5 mmol, ~500.0 eq.) and dry DMSO (170 µL) were used in the reaction to yield polymer $\mathbf{9}_{\textit{cis/exo}\text{-}114}$ (58 mg, M_n = 114 kDa; $Đ$ = 1.12).

5.3.4 Synthesis of Polymer $\mathbf{9}_{\textit{trans/endo}\text{-}90}$

Synthesis followed the representative procedure. $\mathbf{S27}_{\textit{trans/endo}}$ (2.0 mg, 1.0 µmol, 1.0 eq.), 8 µL of catalytic solution (Me_6TREN: 0.47 µmol, 0.5 eq.; $CuBr_2$: 0.19 µmol, 0.2 eq.), methyl acrylate (170 µL, 2.0 mmol, 2000.0 eq.), Cu (0) wire (~3 cm, ~30 mg, 0.5 mmol, ~500.0 eq.) and dry DMSO (170 µL) were used in the reaction to yield polymer $\mathbf{9}_{\textit{trans/endo}\text{-}90}$ (46 mg, M_n = 90 kDa; $Đ$ = 1.16).

5.3.5 Synthesis of Polymer $\mathbf{9}_{\textit{cis/endo}\text{-}92}$

Synthesis followed the representative procedure. $\mathbf{S27}_{\textit{cis/endo}}$ (2.0 mg, 1.0 µmol, 1.0 eq.), 8 µL of catalytic solution (Me_6TREN: 0.47 µmol, 0.5 eq.; $CuBr_2$: 0.19 µmol, 0.2 eq.), methyl acrylate (170 µL, 2.0 mmol, 2000.0 eq.), Cu (0) wire (~3 cm, ~30 mg, 0.5 mmol, ~500.0 eq.) and dry DMSO (170 µL) were used in the reaction to yield polymer $\mathbf{9}_{\textit{cis/endo}\text{-}92}$ (50 mg, M_n = 92 kDa; $Đ$ = 1.17).

5.4 Synthesis of 3-Cargo Polymers

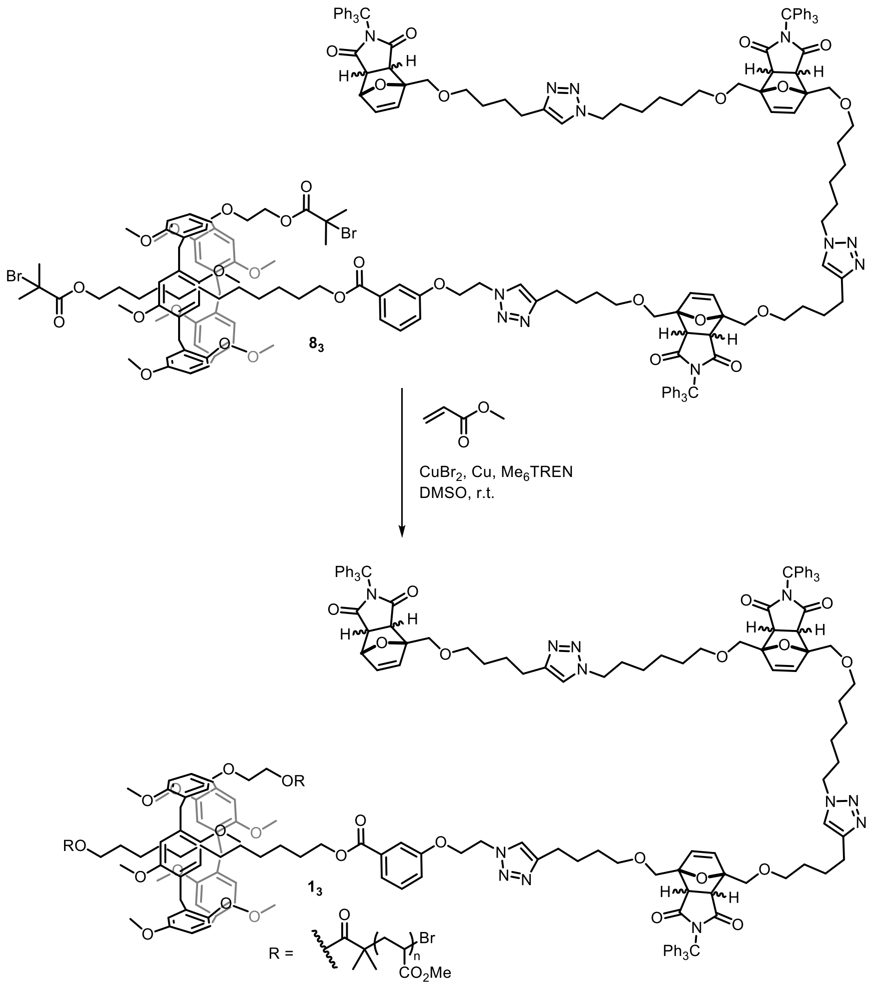

5.4.1 Synthesis of Polymer 1$_3$a$_{-210}$

Synthesis followed the representative procedure. **8$_3$a** (3 mg, 0.9 µmol, 1.0 eq.), 7 µL of catalytic solution (Me$_6$TREN: 0.43 µmol, 0.5 eq.; CuBr$_2$: 0.18 µmol, 0.2 eq.), methyl acrylate (320 µL, 3.6 mmol, 4000.0 eq.), Cu (0) wire (~3 cm, ~30 mg, 0.5 mmol, ~500.0 eq.) and dry DMSO (320 µL) were used in the reaction to yield polymer **1$_3$a$_{-210}$** (112 mg, M_n = 210 kDa; $Đ$ = 1.32).

5.4.2 Synthesis of Polymers 1$_3$b

Synthesis followed the representative procedure. **8$_3$b** (3 mg, 0.9 µmol, 1.0 eq.), 7 µL of catalytic solution (Me$_6$TREN: 0.43 µmol, 0.5 eq.; CuBr$_2$: 0.18 µmol, 0.2 eq.), methyl acrylate (320 µL, 3.6 mmol, 4000.0 eq.), Cu (0) wire (~3 cm, ~30 mg, 0.5 mmol, ~500.0 eq) and dry DMSO (320 µL) were used in the reaction to yield polymer **1$_3$b**$_{-142}$ (72 mg, M_n = 142 kDa; $Đ$ = 1.32), **1$_3$b$_{-171}$** (68 mg, M_n = 171 kDa; $Đ$ = 1.27) and **1$_3$b$_{-178}$** (73 mg, M_n = 178 kDa; $Đ$ = 1.33) from three batches with different reaction time.

5.4.3 Synthesis of Polymer 1$_3$c$_{-174}$

Synthesis followed the representative procedure. **8$_3$c** (3 mg, 0.9 µmol, 1.0 eq.), 7 µL of catalytic solution (Me$_6$TREN: 0.43 µmol, 0.5 eq.; CuBr$_2$: 0.18 µmol, 0.2 eq.), methyl acrylate (320 µL, 3.6 mmol, 4000.0 eq.), Cu (0) wire (~3 cm, ~30 mg, 0.5 mmol, ~500.0 eq.) and dry DMSO (320 µL) were used in the reaction to yield polymer **1$_3$c$_{-174}$** (70 mg, M_n = 174 kDa; $Đ$ = 1.25).

5.4.4 Synthesis of Polymer **1_3d$_{-89}$**

Synthesis followed the representative procedure. **8_3d** (5 mg, 1.5 µmol, 1.0 eq.), 12 µL of catalytic solution (Me_6TREN: 0.72 µmol, 0.5 eq.; $CuBr_2$: 0.30 µmol, 0.2 eq.), methyl acrylate (270 µL, 3.0 mmol, 2000.0 eq.), Cu (0) wire (~3 cm, ~30 mg, 0.5 mmol, ~300.0 eq.) and dry DMSO (270 µL) were used in the reaction to yield polymer **1_3d$_{-89}$** (85 mg, M_n = 89 kDa; $Đ$ = 1.15).

5.5 Synthesis of 5-Cargo Polymers

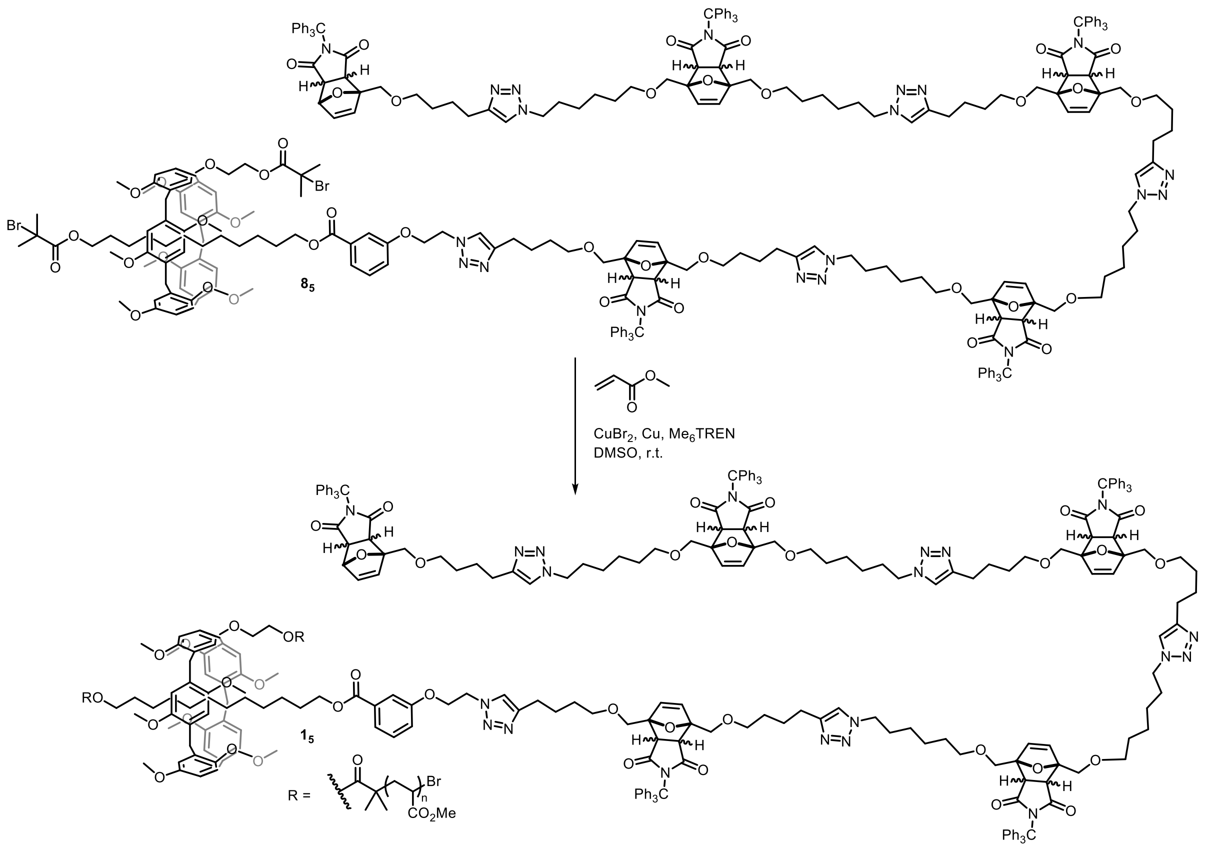

5.5.1 Synthesis of Polymers **1_5**

Synthesis followed the representative procedure. **8_5** (3 mg, 0.6 µmol, 1.0 eq.), 5 µL of catalytic solution (Me_6TREN: 0.31 µmol, 0.5 eq.; $CuBr_2$: 0.13 µmol, 0.2 eq.), methyl acrylate (230 µL, 2.6 mmol, 4000.0 eq.), Cu (0) wire (~3 cm, ~30 mg, 0.5 mmol, ~700.0 eq.) and dry DMSO (230 µL) were used in the reaction to yield polymer **$1_{5\text{-}60}$** (22 mg, M_n = 60 kDa; $Đ$ = 1.20), **$1_{5\text{-}165}$** (52 mg, M_n = 165 kDa; $Đ$ = 1.37) and **$1_{5\text{-}215}$** (88 mg, M_n = 215 kDa; $Đ$ = 1.23) from three batches with different reaction time.

5.6 Synthesis of Reference Polymers

5.6.1 Synthesis of Polymer 3_{ref}

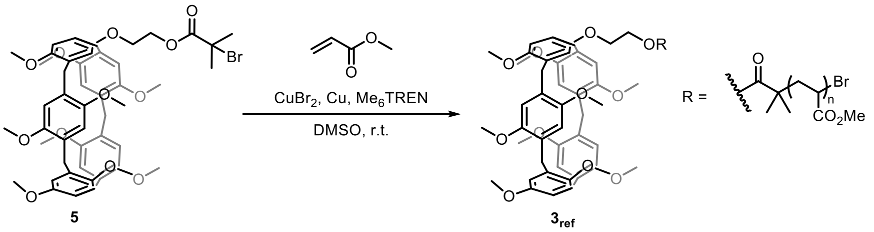

5 3_{ref}

Synthesis followed the representative procedure. **5** (2.0 mg, 2.2 µmol, 1.0 eq.), 31 µL of catalytic solution (Me_6TREN: 1.03 µmol, 0.5 eq.; $CuBr_2$: 0.43 µmol, 0.2 eq.), methyl acrylate (380 µL, 4.3 mmol, 2000.0 eq.), Cu (0) wire (~3 cm, ~30 mg, 0.5 mmol, ~250.0 eq.) and dry DMSO (380 µL) were used in the reaction to yield polymer $\mathbf{3_{ref}}$ (90 mg, M_n = 76 kDa; $Đ$ = 1.14).

5.7 Synthesis of Matrix Polymer, $S56_{-153}$

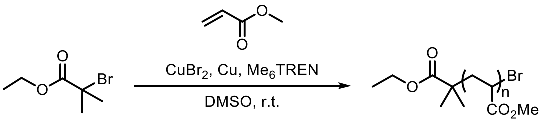

S56

Synthesis followed the representative procedure. Ethyl α-bromoisobutyrate (2.5 mg, 12.8 µmol, 1.0 eq.), 103 µL of catalytic solution (Me_6TREN: 6.41 µmol, 0.5 eq.; $CuBr_2$: 2.56 µmol, 0.2 eq.), methyl acrylate (2.3 mL, 25.6 mmol, 2000.0 eq.), Cu (0) wire (~3 cm, ~30 mg, 0.5 mmol, ~250.0 eq.) and dry DMSO (2.3 mL) were used in the reaction to yield polymer $\mathbf{S56_{-153}}$ (1.3 g, M_n = 153 kDa; $Đ$ = 1.07).

5.8 Synthesis of Alternative Cargo Polymers – Drug-Containing-Cargo Release

5.8.1 Synthetic Route to Drug-Containing-Cargo Polymer, $11_{\text{-}127}$

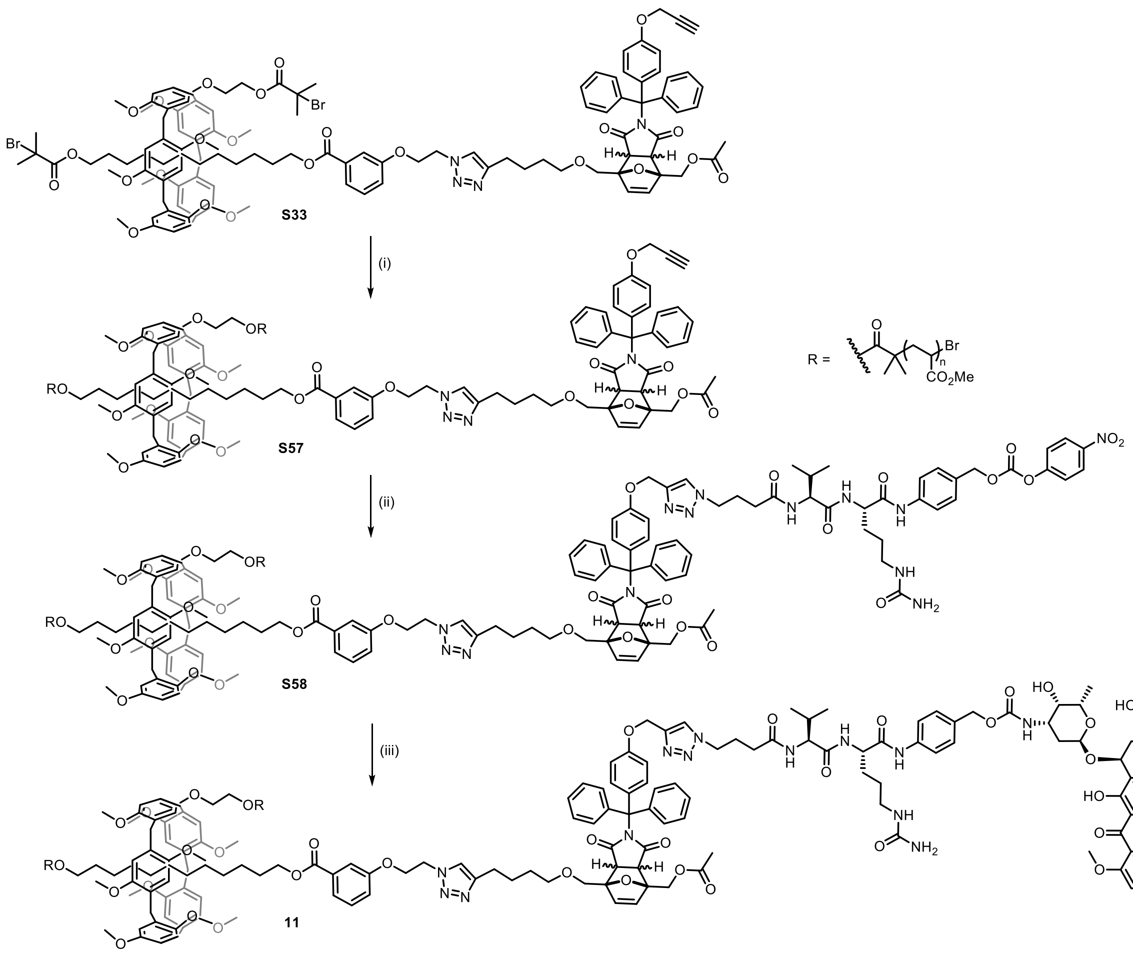

Figure S19. Synthesis of drug-containing-cargo polymer **11**. Conditions: (i) Methyl acrylate, $CuBr_2$, Cu, Me_6TREN, DMSO, r.t.; (ii) **S36**, CuBr, PMDETA, DMF, r.t., 2 h; (iii) Doxorubicin hydrochloride, DIPEA, DMF, r.t., 16 h.

5.8.2 Synthesis of Polymer **S57$_{-108}$**

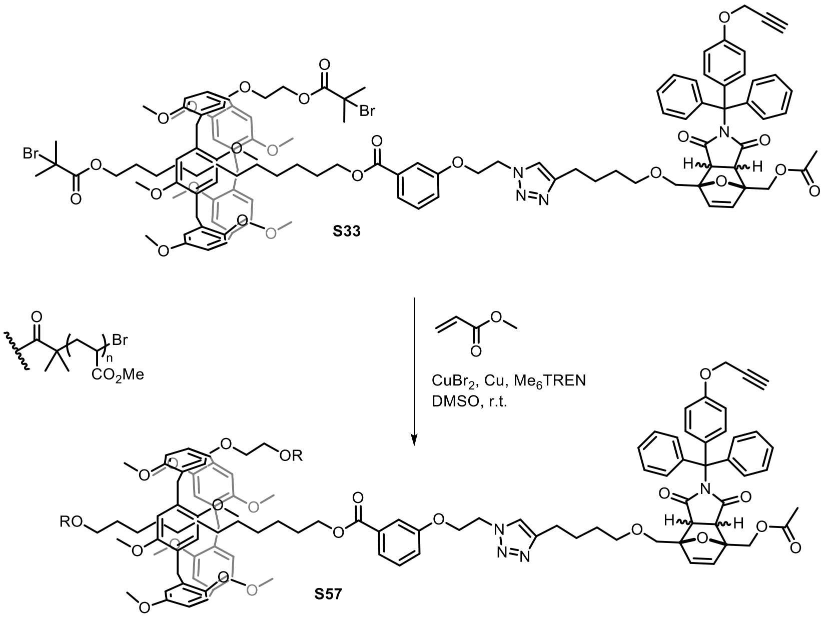

Synthesis followed the representative procedure. **S33** (6.0 mg, 2.8 µmol, 1.0 eq.), 23 µL of catalytic solution ($CuBr_2$: 0.6 µmol, 0.2 eq.; Me_6TREN: 1.4 µmol, 0.5 eq.), methyl acylate (506 µL, 5.7 mmol, 2000.0 eq.), Cu (0) wire (~3 cm, ~30 mg, 0.5 mmol, ~166.0 eq.) and dry DMSO (506 µL) were used in the reaction to yield polymer **S57$_{-108}$** (150 mg, M_n = 108 kDa; $Đ$ = 1.21).

5.8.3 Synthesis of Polymer **S58$_{-122}$**

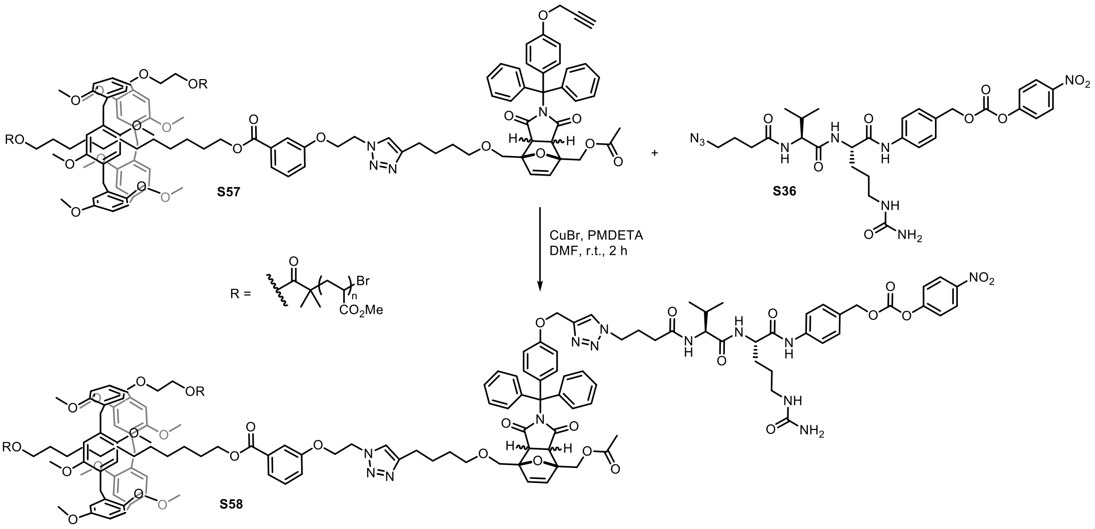

Three sealed 5 mL microwave vials (*A*, *B*, and *C*) were prepared as follows: *A* contained PMDETA (0.6 mg, 3.7 µmol, 10.0 eq.) in dry DMF (1 mL), *B* contained CuBr (0.6 mg, 4.4 µmol, 12.0 eq.), and *C* contained **S57$_{-108}$** (40 mg, 0.4 µmol, 1.0 eq.) and **S36** (1.2 mg, 1.9 µmol, 5.0 eq.). Vials *B* and *C* were subjected to three N_2/vacuum cycles before use. The solution in vial *A* was degassed by bubbling with N_2 for 10 min before being transferred to vial *B via* cannula; the resulting mixture was stirred until all the CuBr had dissolved. The CuBr/PMDETA solution in vial *B* was then transferred to vial *C via* cannula; the resulting reaction mixture was stirred for 2 h at room temperature. DCM (25 mL) was added in the reaction mixture before washed with aqueous EDTA solution (0.25 M, pH 7, 10 mL) and brine (10 mL). The organic layer was collected and dried with magnesium sulfate. The mixture was filtered and evaporated by rotavap in a round bottom flask to make a polymer film. This polymer film was washed with MeOH (3 x 25 mL) and dried under vacuum for 16 h to to give polymer **S58$_{-122}$** (30 mg, M_n = 122 kDa; $Đ$ = 1.31).

5.8.4 Synthesis of Polymer 11$_{-127}$

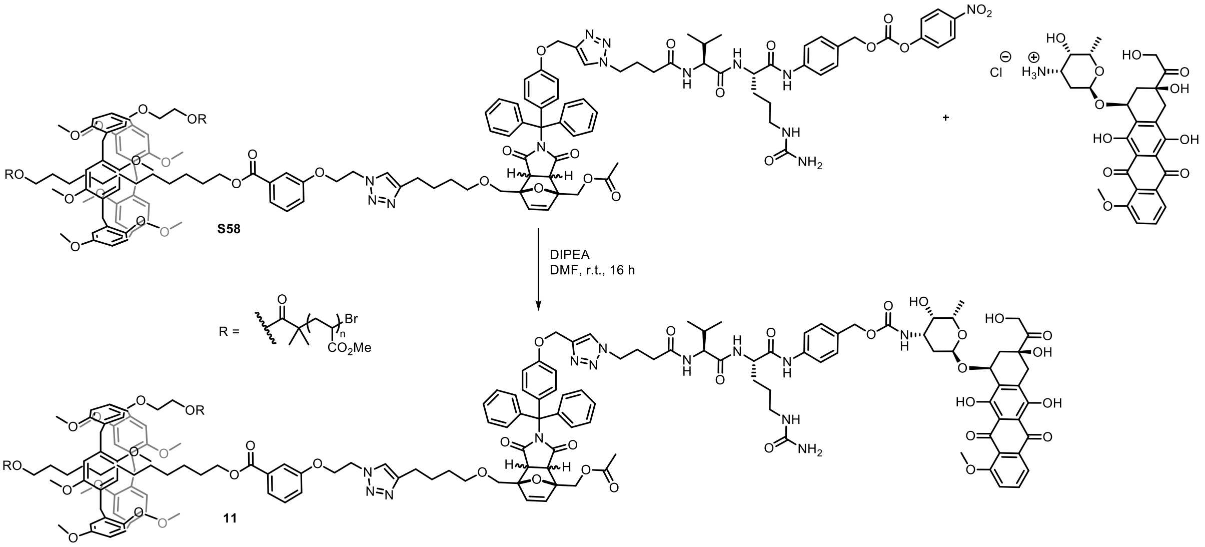

Three sealed 5 mL microwave vials (*A*, *B*, and *C*) were prepared as follows: *A* contained DIPEA (0.3 mg, 2.0 µmol, 8.0 eq.) in dry DMF (1 mL), *B* contained doxorubicin hydrochloride (1.5 mg, 2.5 µmol, 10.0 eq.), and *C* contained **S58**$_{-122}$ (30 mg, 0.3 µmol, 1.0 eq.). Vials *B* and *C* were subjected to three N_2/vacuum cycles before use. The solution in vial *A* was degassed by bubbling with N_2 for 10 min before being transferred to vial *B* *via* cannula and stirred for 5min. The solution in vial *B* was then transferred to vial *C* *via* cannula; the resulting reaction mixture was stirred for 16 h at room temperature. The solvent was evaporated by rotavap in a round bottom flask to make a polymer film. This polymer film was washed with MeOH (3 x 25 mL) and dried under vacuum for 16 h to to give polymer **11**$_{-127}$ (28 mg, M_n = 127 kDa; $Đ$ = 1.31).

5.9 Synthesis of Alternative-Cargo Polymers – N-(1-pyrenyl)maleimide Cargo Release

5.9.1 Synthesis of N-(1-pyrenyl)maleimide-Cargo Polymer, 13$_{-119}$

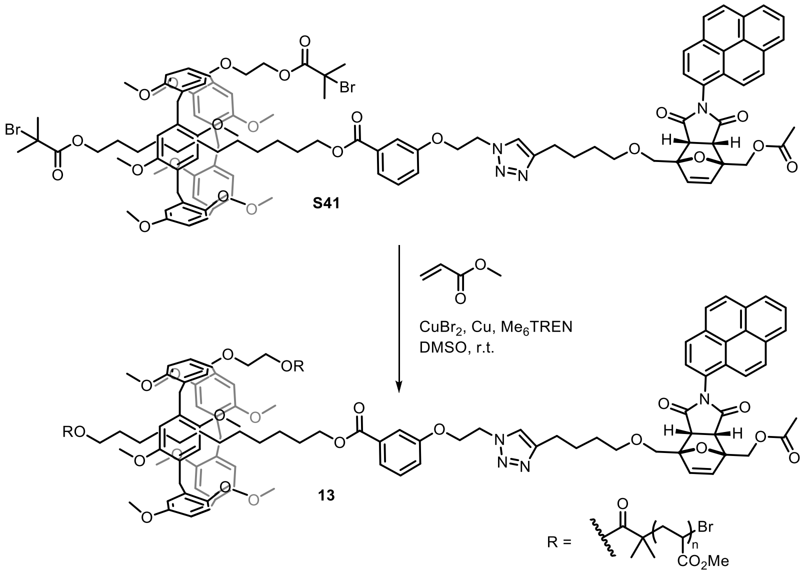

Synthesis followed the representative procedure. **S41** (4.0 mg, 2.0 µmol, 1.0 eq.), 16 µL of catalytic solution ($CuBr_2$: 0.4 µmol, 0.2 eq.; Me_6TREN: 1.0 µmol, 0.5 eq.), methyl acylate (354 µL, 4.0 mmol, 2000.0 eq.), Cu (0) wire (~3 cm, ~30 mg, 0.5 mmol, ~238.0 eq.) and dry DMSO (354 µL) were used in the reaction to yield polymer **13**$_{-119}$ (84 mg, M_n = 119 kDa; $Đ$ = 1.20).

5.9.2 Synthesis of N-(1-pyrenyl)maleimide-Cargo Control Polymer, S59$_{-77}$

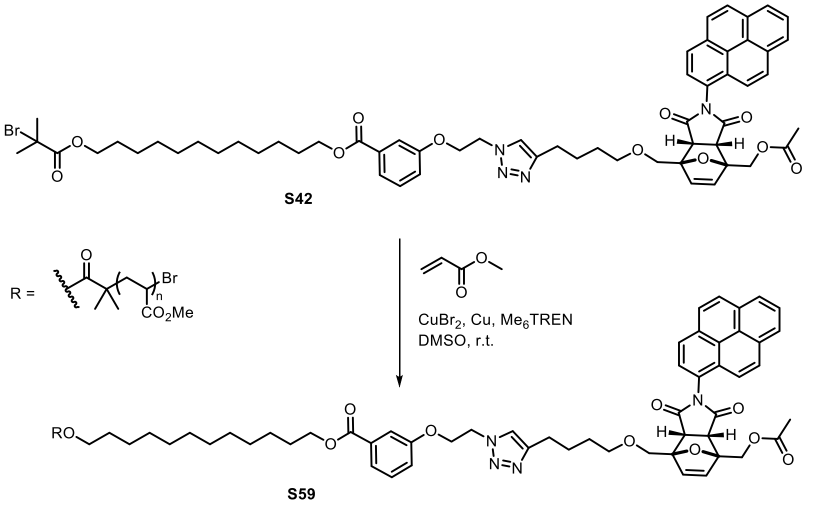

Synthesis followed the representative procedure. **S42** (2.0 mg, 1.8 µmol, 1.0 eq.), 15 µL of catalytic solution ($CuBr_2$: 0.3 µmol, 0.2 eq.; Me_6TREN: 0.9 µmol, 0.5 eq.), methyl acylate (328 µL, 3.7 mmol, 2000.0 eq.), Cu (0) wire (~3 cm, ~30 mg, 0.5 mmol, ~256.0 eq.) and dry DMSO (328 µL) were used in the reaction to yield polymer **S59$_{-77}$** (50 mg, M_n = 77 kDa; $Đ$ = 1.29).

5.9.3 Synthesis of N-(1-pyrenyl)maleimide-Cargo Reference Polymer, 10$_{-80}$

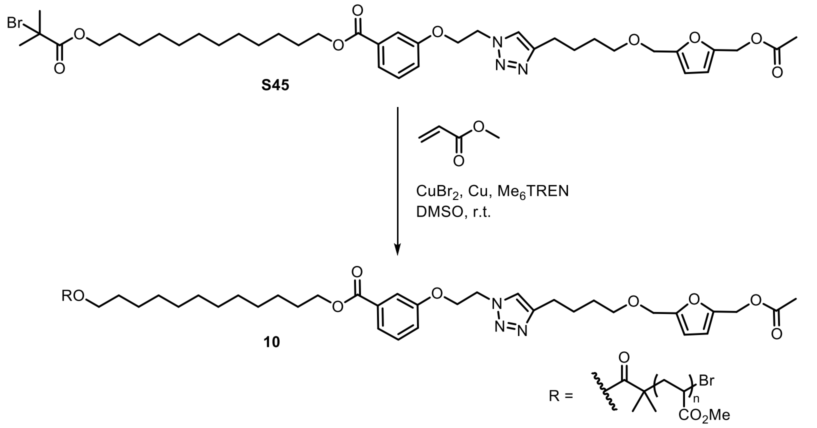

Synthesis followed the representative procedure. **S45** (1.0 mg, 1.3 µmol, 1.0 eq.), 10 µL of catalytic solution ($CuBr_2$: 0.3 µmol, 0.2 eq.; Me_6TREN: 0.6 µmol, 0.5 eq.), methyl acylate (226 µL, 2.5 mmol, 2000.0 eq.), Cu (0) wire (~3 cm, ~30 mg, 0.5 mmol, ~400.0 eq.) and dry DMSO (226 µL) were used in the reaction to yield polymer **10$_{-80}$** (36 mg, M_n = 80 kDa; $Đ$ = 1.27).

5.10 Synthesis of Alternative-Cargo Polymers – Trityl Cargo Release

5.10.1 Synthesis of Trityl-Cargo Polymer, $14_{\text{-}124}$

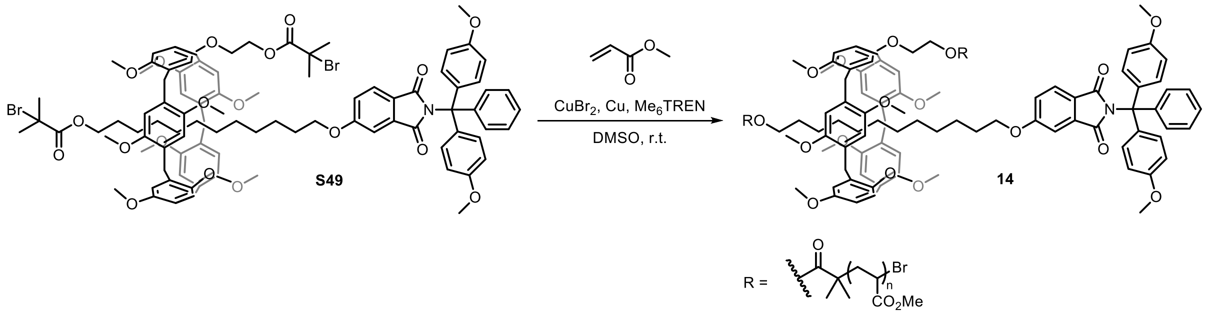

Synthesis followed the representative procedure. **S49** (3.0 mg, 1.7 µmol, 1.0 eq.), 14 µL of catalytic solution ($CuBr_2$: 0.3 µmol, 0.2 eq.; Me_6TREN: 0.8 µmol, 0.5 eq.), methyl acylate (310 µL, 3.5 mmol, 2000.0 eq.), Cu (0) wire (~3 cm, ~30 mg, 0.5 mmol, ~272.0 eq.) and dry DMSO (310 µL) were used in the reaction to yield polymer **14**$_{\text{-}124}$ (85 mg, M_n = 124 kDa; $Đ$ = 1.19).

5.10.2 Synthesis of Trityl-Cargo Control Polymer, $S60_{\text{-}95}$

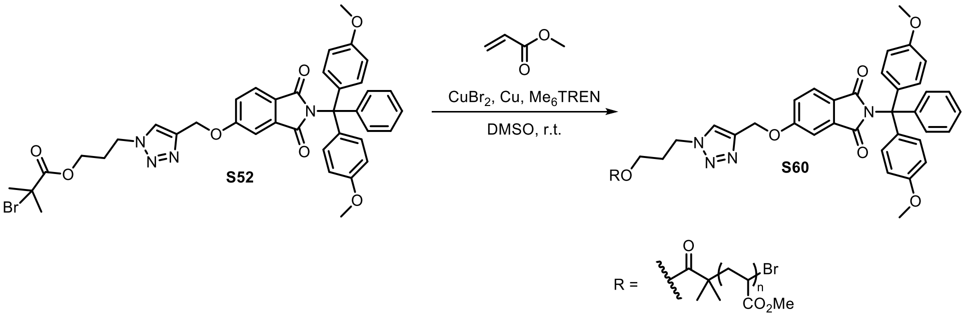

Synthesis followed the representative procedure. **S52** (3.0 mg, 4.0 µmol, 1.0 eq.), 32 µL of catalytic solution ($CuBr_2$: 0.8 µmol, 0.2 eq.; Me_6TREN: 1.9 µmol, 0.5 eq.), methyl acylate (712 µL, 8.0 mmol, 2000.0 eq.), Cu (0) wire (~3 cm, ~30 mg, 0.5 mmol, ~118.0 eq.) and dry DMSO (712 µL) were used in the reaction to yield polymer **S60**$_{\text{-}95}$ (120 mg, M_n = 95 kDa; $Đ$ = 1.15).

5.10.3 Synthesis of Trityl-Cargo Reference Polymer, $S61_{\text{-}72}$

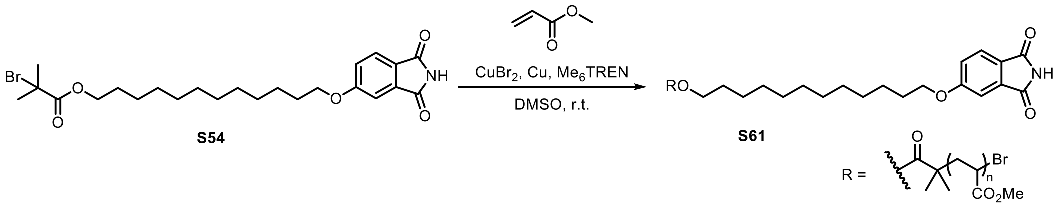

Synthesis followed the representative procedure. **S54** (3.0 mg, 6.1 µmol, 1.0 eq.), 48 µL of catalytic solution ($CuBr_2$: 1.2 µmol, 0.2 eq.; Me_6TREN: 2.9 µmol, 0.5 eq.), methyl acylate (1081 µL, 12.1 mmol, 2000.0 eq.), Cu (0) wire (~3 cm, ~30 mg, 0.5 mmol, ~78.0 eq.) and dry DMSO (1081 µL) were used in the reaction to yield polymer **S61**$_{\text{-}72}$ (220 mg, M_n = 72 kDa; $Đ$ = 1.16).

5.11 SEC Data for Synthesised Polymers

Table S1. M_n and *Đ* values for all synthesised polymers.

Polymer	M_n / kDa	*Đ*
$\mathbf{S55}_{exo\text{-}112}$	112	1.25
$\mathbf{S55}_{endo\text{-}65}$	65	1.20
$\mathbf{9}_{trans/exo\text{-}109}$	109	1.12
$\mathbf{9}_{trans/exo\text{-}114}$	114	1.17
$\mathbf{9}_{cis/exo\text{-}114}$	114	1.12
$\mathbf{9}_{trans/endo\text{-}90}$	90	1.16
$\mathbf{9}_{cis/endo\text{-}92}$	92	1.17
$\mathbf{1_3a}_{\text{-}210}$	210	1.32
$\mathbf{1_3b}_{\text{-}142}$	142	1.32
$\mathbf{1_3b}_{\text{-}171}$	171	1.27
$\mathbf{1_3b}_{\text{-}178}$	178	1.33
$\mathbf{1_3c}_{\text{-}174}$	174	1.25
$\mathbf{1_3d}_{\text{-}89}$	89	1.15
$\mathbf{1}_{5\text{-}60}$	60	1.20
$\mathbf{1}_{5\text{-}165}$	165	1.37
$\mathbf{1}_{5\text{-}215}$	215	1.23
$\mathbf{3}_{ref}$	76	1.14
$\mathbf{S56}_{\text{-}153}$	153	1.07
$\mathbf{S57}_{\text{-}108}$	108	1.21
$\mathbf{S58}_{\text{-}122}$	122	1.31
$\mathbf{11}_{\text{-}127}$	127	1.31
$\mathbf{13}_{\text{-}119}$	119	1.20
$\mathbf{S59}_{\text{-}77}$	77	1.29
$\mathbf{10}_{\text{-}80}$	80	1.27
$\mathbf{14}_{\text{-}124}$	124	1.19
$\mathbf{S60}_{\text{-}95}$	95	1.15
$\mathbf{S61}_{\text{-}72}$	72	1.16

5.12 SEC Traces for Control Polymers, S55

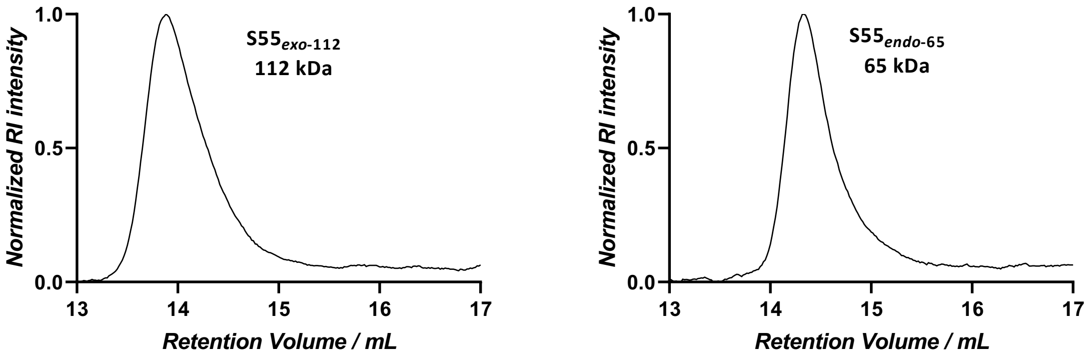

Figure S20. SEC traces for control polymers **S55**.

5.13 SEC Traces for 1-Cargo Polymers, 9

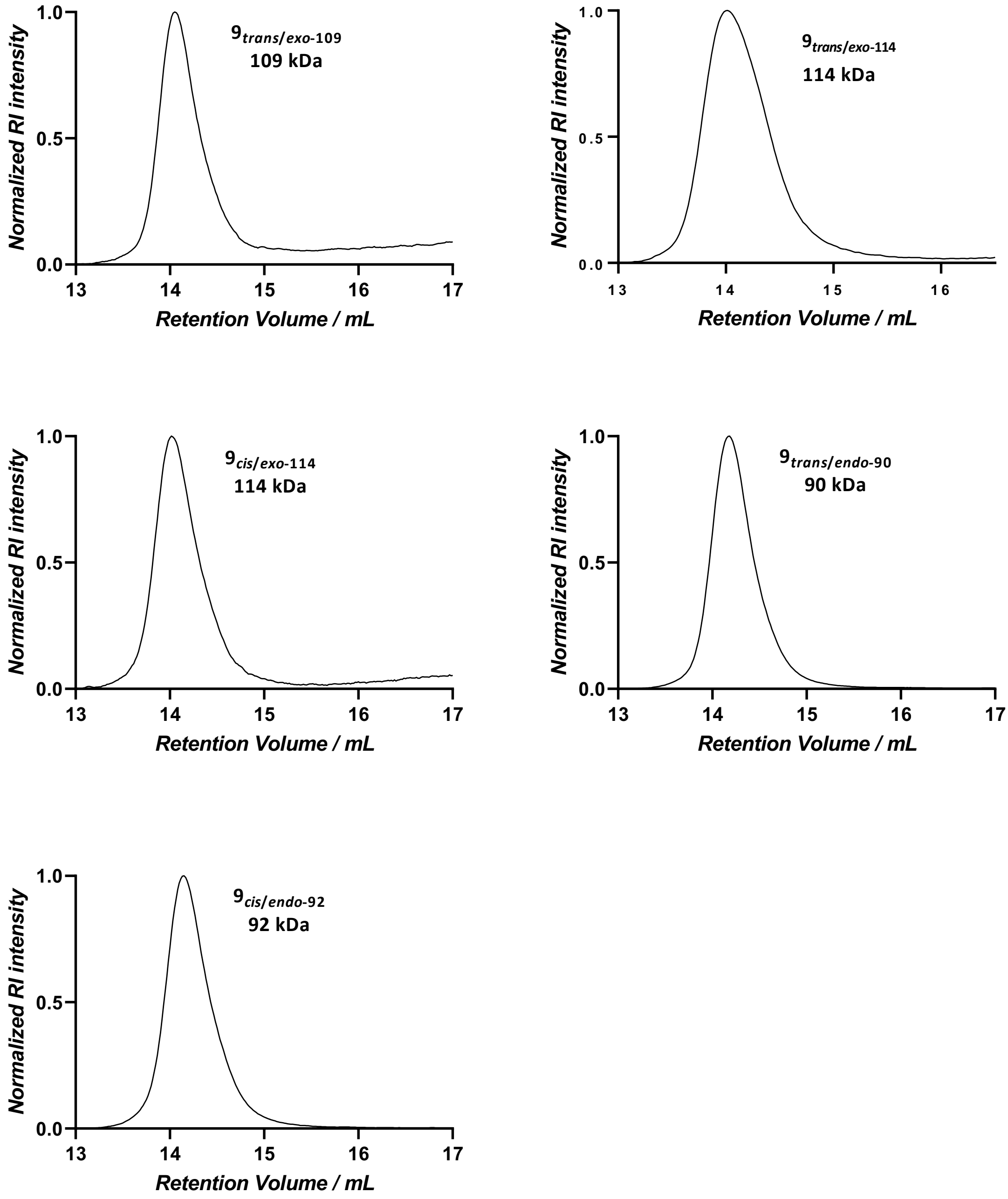

Figure S21. SEC traces for 1-cargo polymers **9**.

5.14 SEC Traces for 3-Cargo Polymers, 1_3

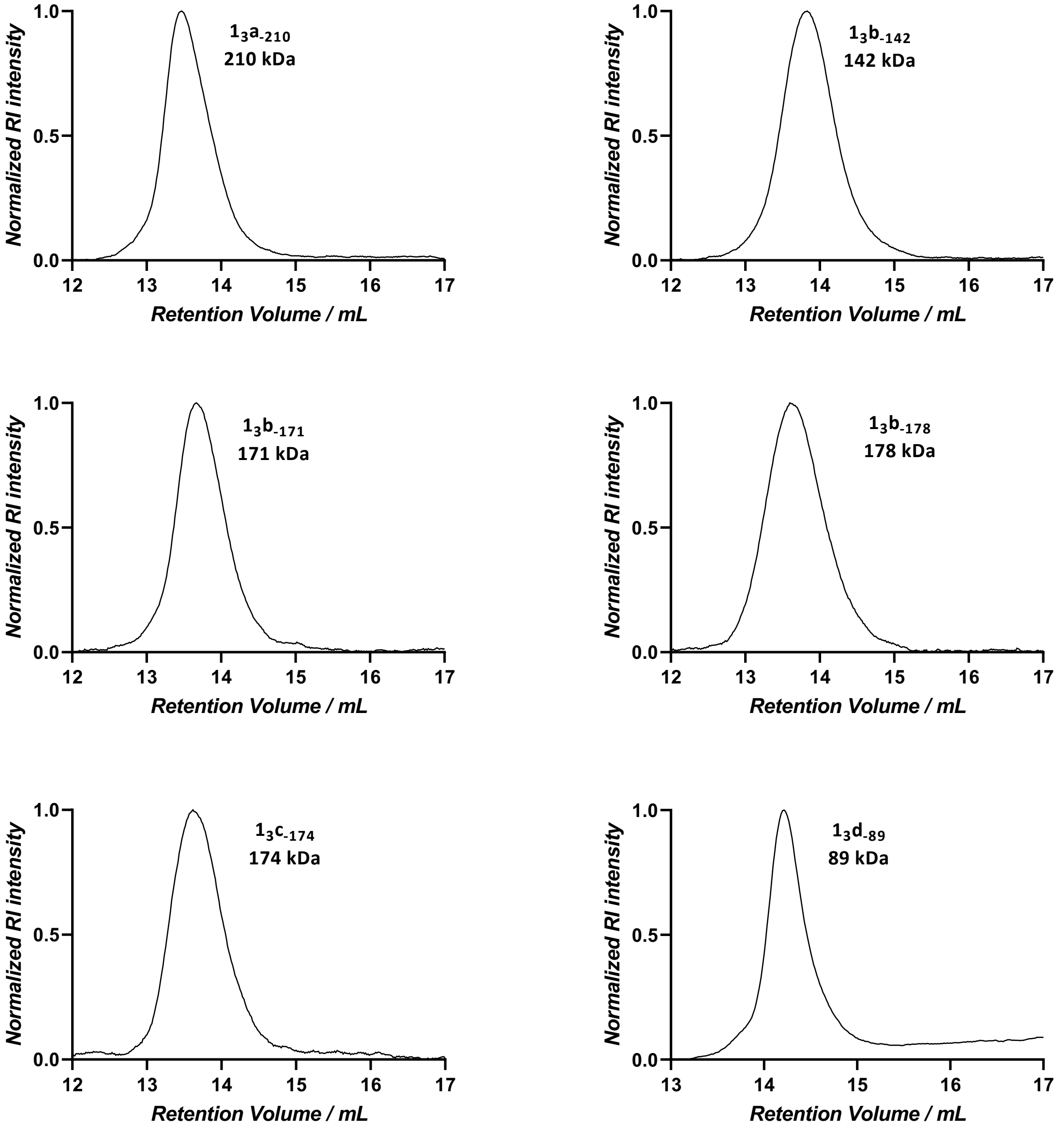

Figure S22. SEC traces for 3-cargo polymers **1_3**.

5.15 SEC Traces for 5-Cargo Polymers, 1_5

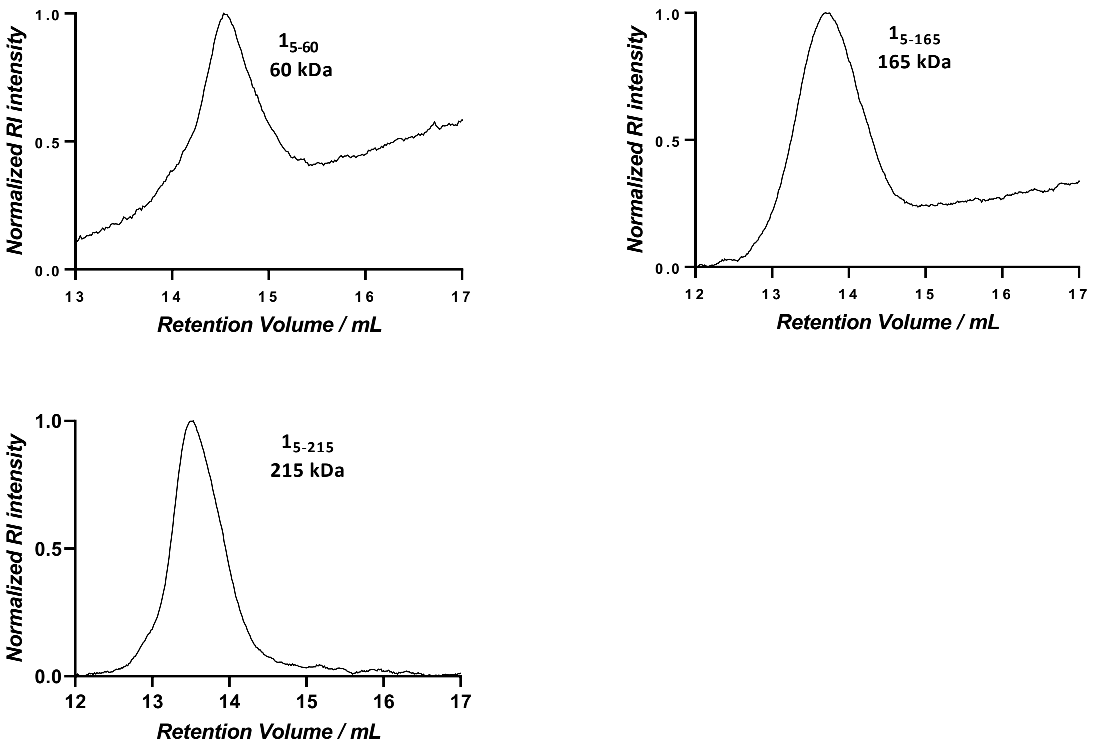

Figure S23. SEC traces for 5-cargo polymers $\mathbf{1_5}$.

5.16 SEC Traces for Reference Polymer, 3_{ref}

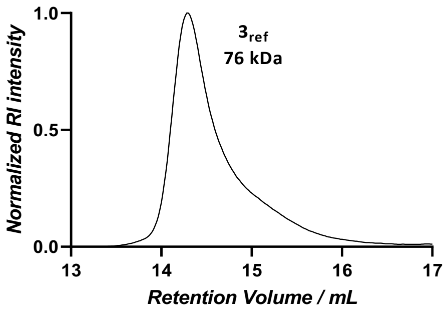

Figure S24. SEC trace of reference polymer $\mathbf{3_{ref}}$.

5.17 SEC Trace of Matrix Polymer, S56

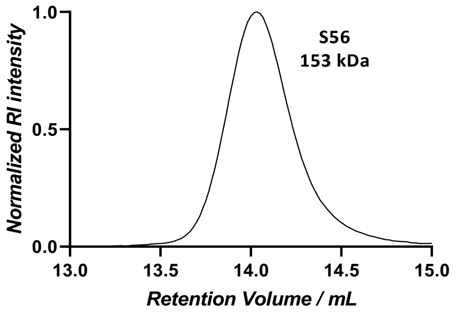

Figure S25. SEC trace of polymer **S56**$_{-153}$.

5.18 SEC Traces of Alternative Cargo Polymers – Drug-Containing-Cargo Release

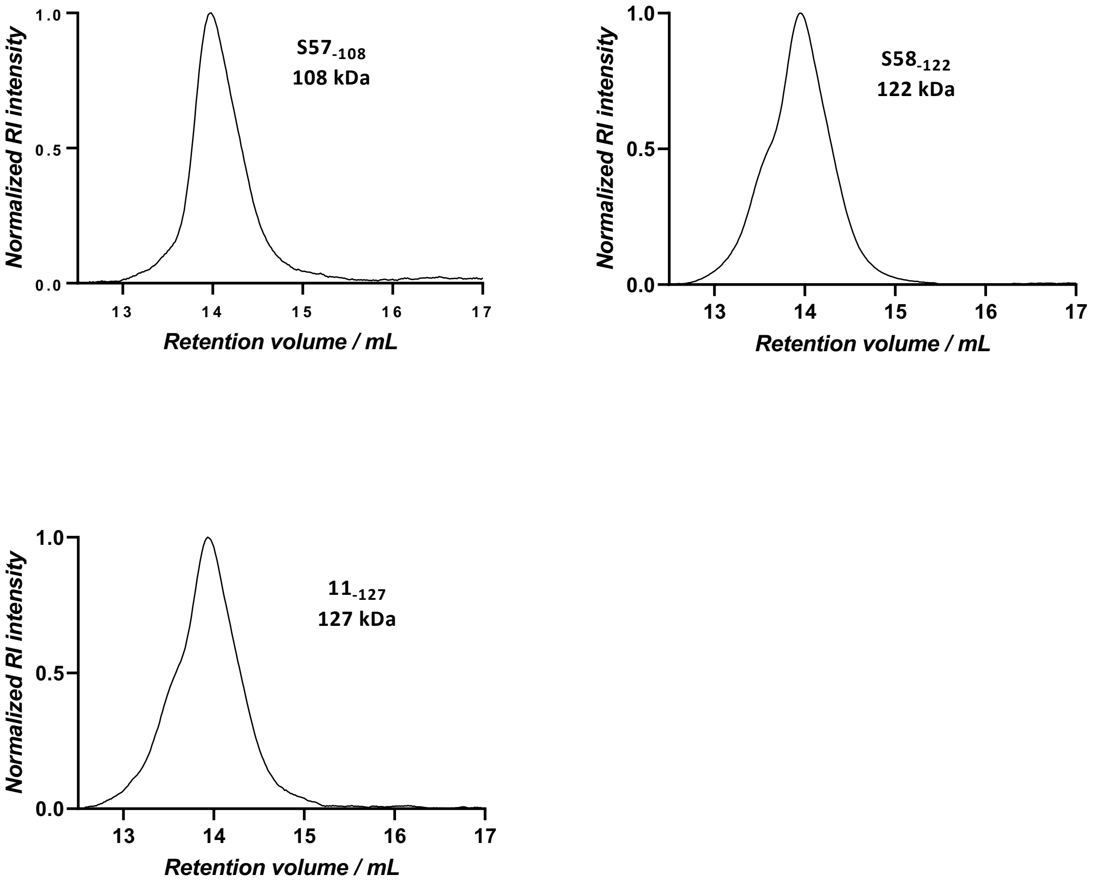

Figure S26. SEC traces for polymers **S57**$_{-108}$, **S58**$_{-122}$, and **11**$_{-127}$.

5.19 SEC Traces of Alternative Cargo Polymers – N-(1-pyrenyl)maleimide-Cargo Release

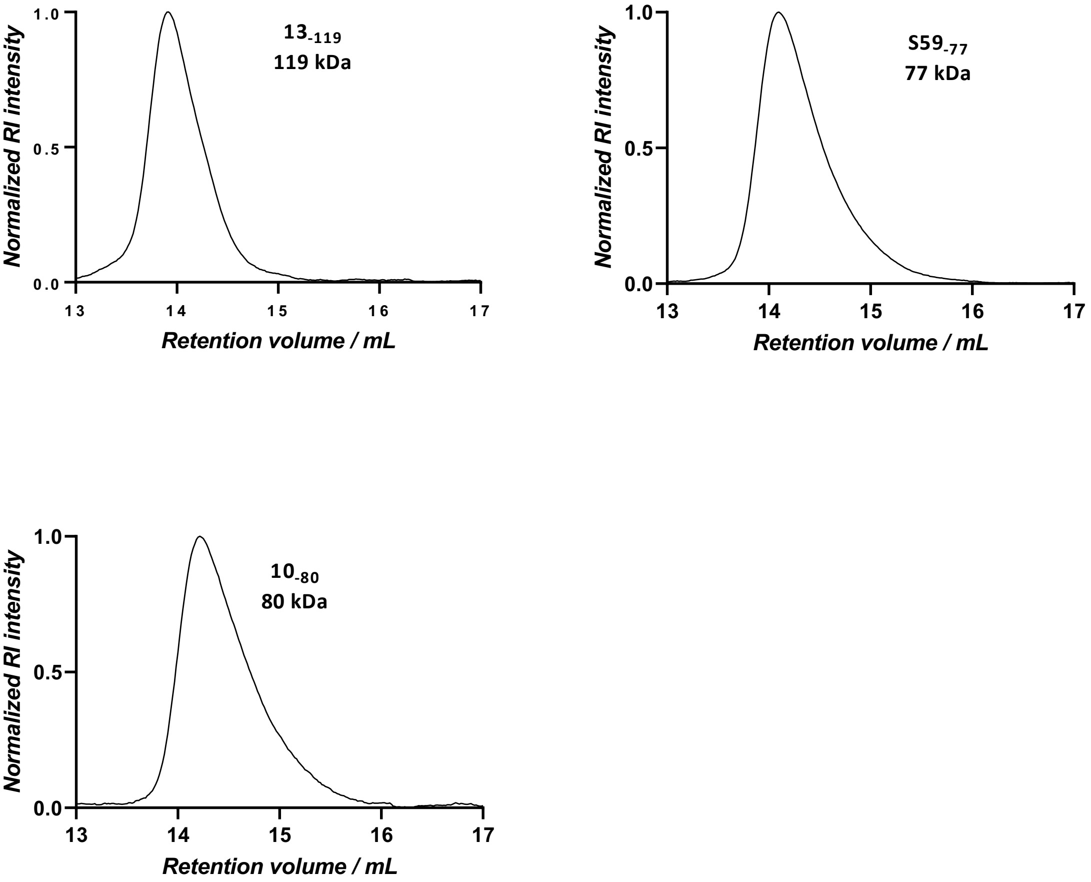

Figure S27. SEC traces for polymers **13**$_{-119}$, **S59**$_{-77}$, and **10**$_{-80}$.

5.20 SEC Traces of Alternative Cargo Polymers – Trityl-Cargo Release

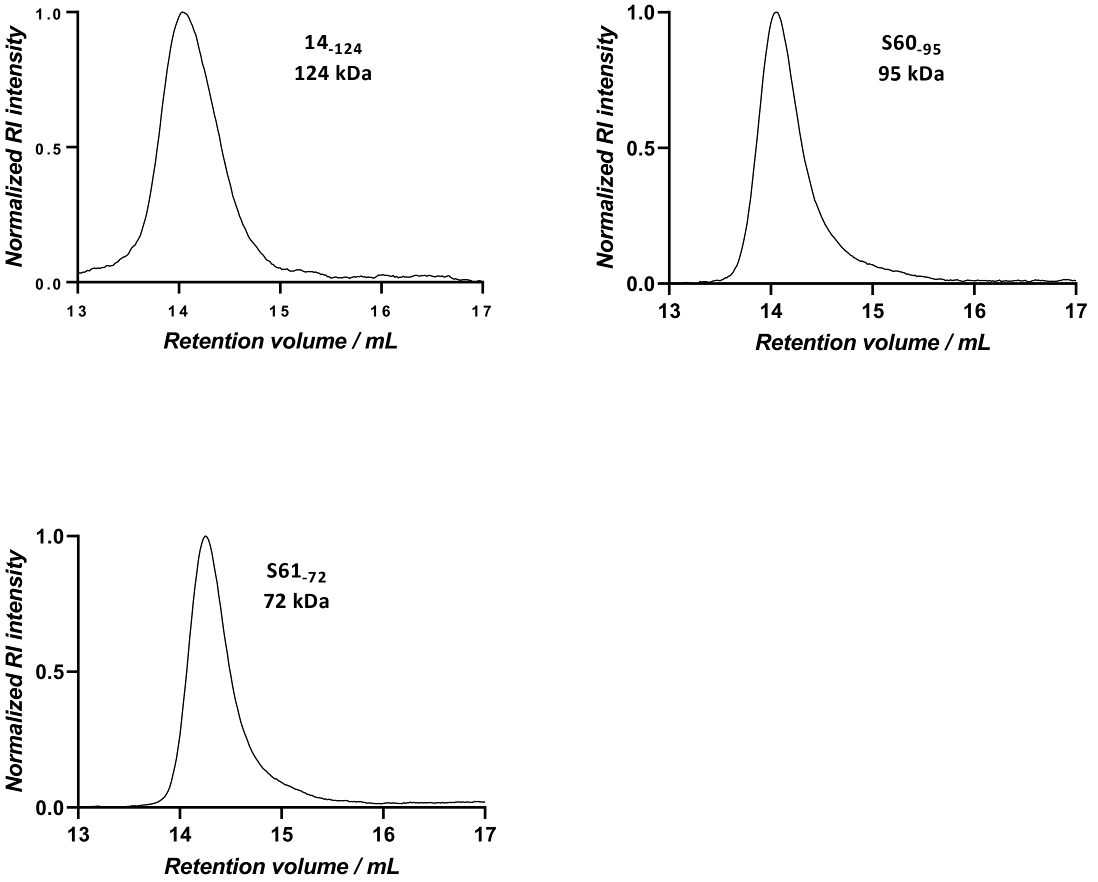

Figure S28. SEC traces for polymers **14**$_{-124}$, **S60**$_{-95}$, and **S61**$_{-72}$.

6 Mechanophore Activation via Ultrasound

6.1 General Procedure for Sonication Experiments

The appropriate polymer (20 mg) was added to a Suslick cell and dissolved in the appropriate solvent (15 mL). The solution was degassed by bubbling N_2 through it for a minimum of 10 min prior to the start of sonication; bubbling of N_2 was also maintained throughout the experiment. The Suslick cell was cooled with an ice bath throughout the duration of the sonication to maintain a temperature of ~ 5-10 °C inside the cell. Pulsed ultrasound was applied to the system (1 s ON / 1 s OFF, 25% amplitude (13.0 W cm^{-2}), 20 kHz) for the desired period of time. After sonication, the solvent was evaporated and the polymer was analysed by SEC and NMR spectroscopy. The post-sonication polymer was recovered and washed with MeOH to extract any small molecules not attached to polymer chains. The remaining MeOH-washed polymer and the concentrated MeOH washings were then analysed by NMR spectroscopy.

6.2 Sonication of Control Polymers, S55

Sonication of polymers **S55**$_{\textit{exo}\text{-}112}$ and **S55**$_{\textit{endo}\text{-}65}$, using the methodology described in the general procedure (*Section 6.1*) and with the solvent used being acetonitrile, was carried out in order to show that activation of the mechanophore under sonication conditions was due to mechanical force and not other effects. SEC analysis of the sonicated polymers showed complete cleavage (M_n of the post-sonication material was less than half of that of the pre-sonication polymer). Comparison of ^{1}H NMR spectra of the pre- and post-sonication polymers show only a small inconsequential amount of furan species (< 4%) having formed during the sonication procedure along with a respective quantity of maleimide; this shows that the species cannot undergo the desired retro Diels-Alder reaction to any meaningful extent without the polymer-attached rotaxane actuator used in polymers **9**, $\mathbf{1_3}$, and $\mathbf{1_5}$.

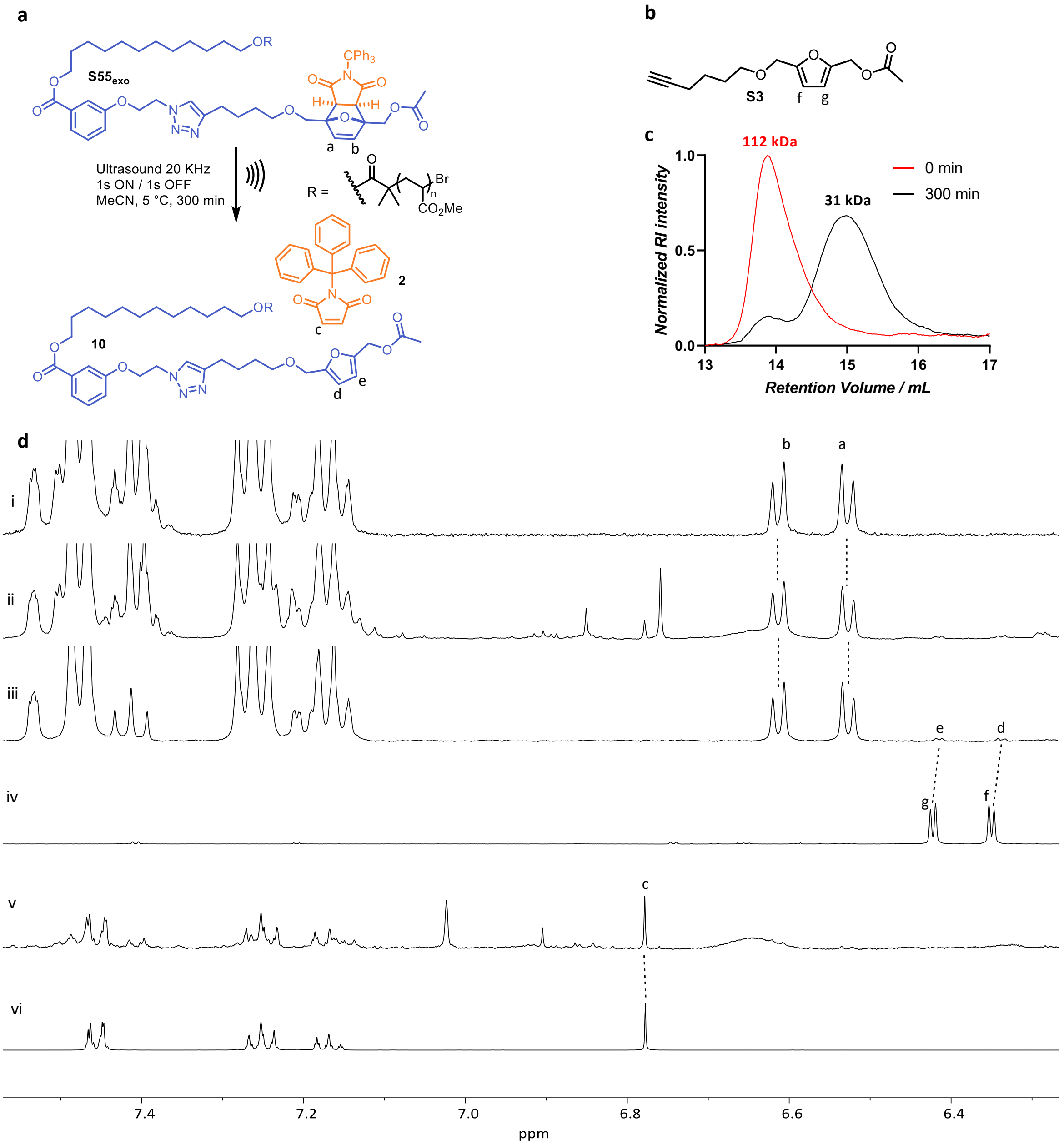

Figure S29. Sonication of polymer **S55*exo*-112** in MeCN. Sonication of polymer **S55*exo*** affords polymer fragments **10** and small molecule **2** (a). Reference compound **S3** (b). SEC traces of polymer **S55*exo*-112** (c) with M_n values before (red) and after (black) sonication. Partial NMR (400 MHz, Acetone-d_6, 298 K) spectra comparison (d) of the pre-sonication polymer **S55*exo*-112** (i), post-sonication polymer before being washed with methanol (ii), post-sonication mixture after being washed with methanol (iii), reference compound **S3** (iv), concentrated methanol washings (v), and reference compound **2** (vi).

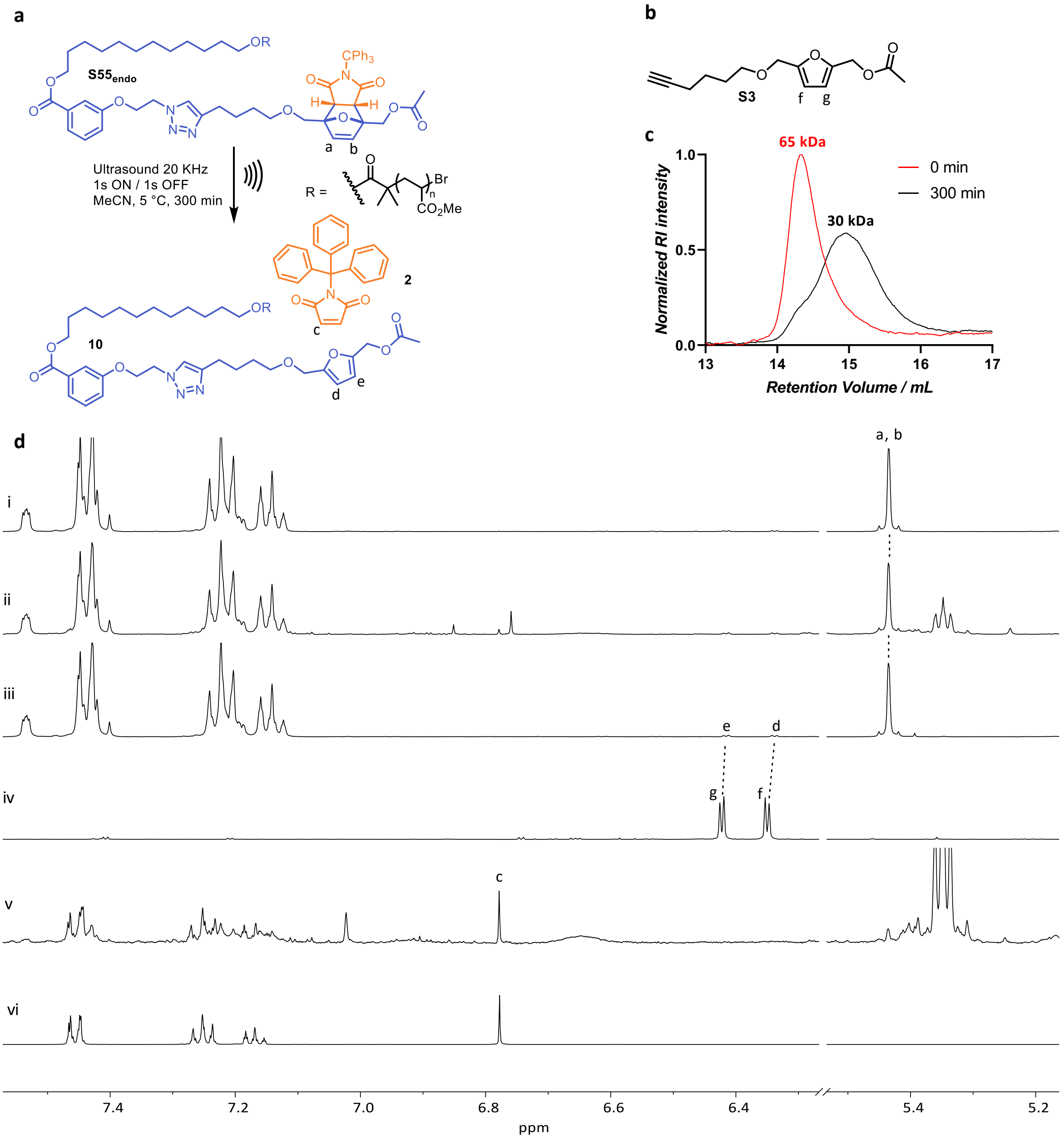

Figure S30. Sonication of polymer **S55**$_{endo\text{-}65}$ in MeCN. Sonication of polymer **S55**$_{endo}$ affords polymer fragments **10** and small molecule **2** (a). Reference compound **S3** (b). SEC traces of polymer **S55**$_{endo\text{-}65}$ (c) with M_n values before (red) and after (black) sonication. Partial NMR (400 MHz, Acetone-d_6, 298 K) spectra comparison (d) of the pre-sonication polymer **S55**$_{endo\text{-}65}$ (i), post-sonication polymer before being washed with methanol (ii), post-sonication mixture after being washed with methanol (iii), reference compound **S3** (iv), concentrated methanol washings (v), and reference compound **2** (vi).

6.3 Sonication of 1-Cargo Polymers, 9

Sonication of polymers $\mathbf{9}_{\textit{trans/exo}\text{-}109}$, $\mathbf{9}_{\textit{cis/exo}\text{-}114}$, $\mathbf{9}_{\textit{trans/endo}\text{-}90}$ and $\mathbf{9}_{\textit{cis/endo}\text{-}92}$, using the methodology described in the general procedure (*Section 6.1*) and with the solvent used being acetonitrile, was carried out to determine the extent of activation of the Diels-Alder structure for both *trans* and *cis* forms of the rotaxane structure along with the *exo* and *endo* stereoisomers of the Diels-Alder unit itself. SEC analysis of the sonicated polymers showed complete cleavage (M_n of the post-sonication material was less than half of that of the pre-sonication polymer).

Comparison of the ^{1}H NMR spectra of pre- and post-sonication polymers showed that all four isomers demonstrated the desired retro Diels-Alder reaction upon mechanical force application; this is evidenced by clear formation of furan species along with the respective maleimide group while the intact Diels-Alder structure clearly decreased in relative intensity. Comparison to reference species also demonstrated that the post-sonication material had some intact rotaxane species remaining. Additionally, the ^{1}H NMR spectrum of the concentrated MeOH washings of polymer $\mathbf{9}_{\textit{cis/exo}\text{-}114}$ showed a small quantity of an intact Diels-Alder structure indicating the occurrence of unstoppering; this phenomenon was observed for this polymer only.

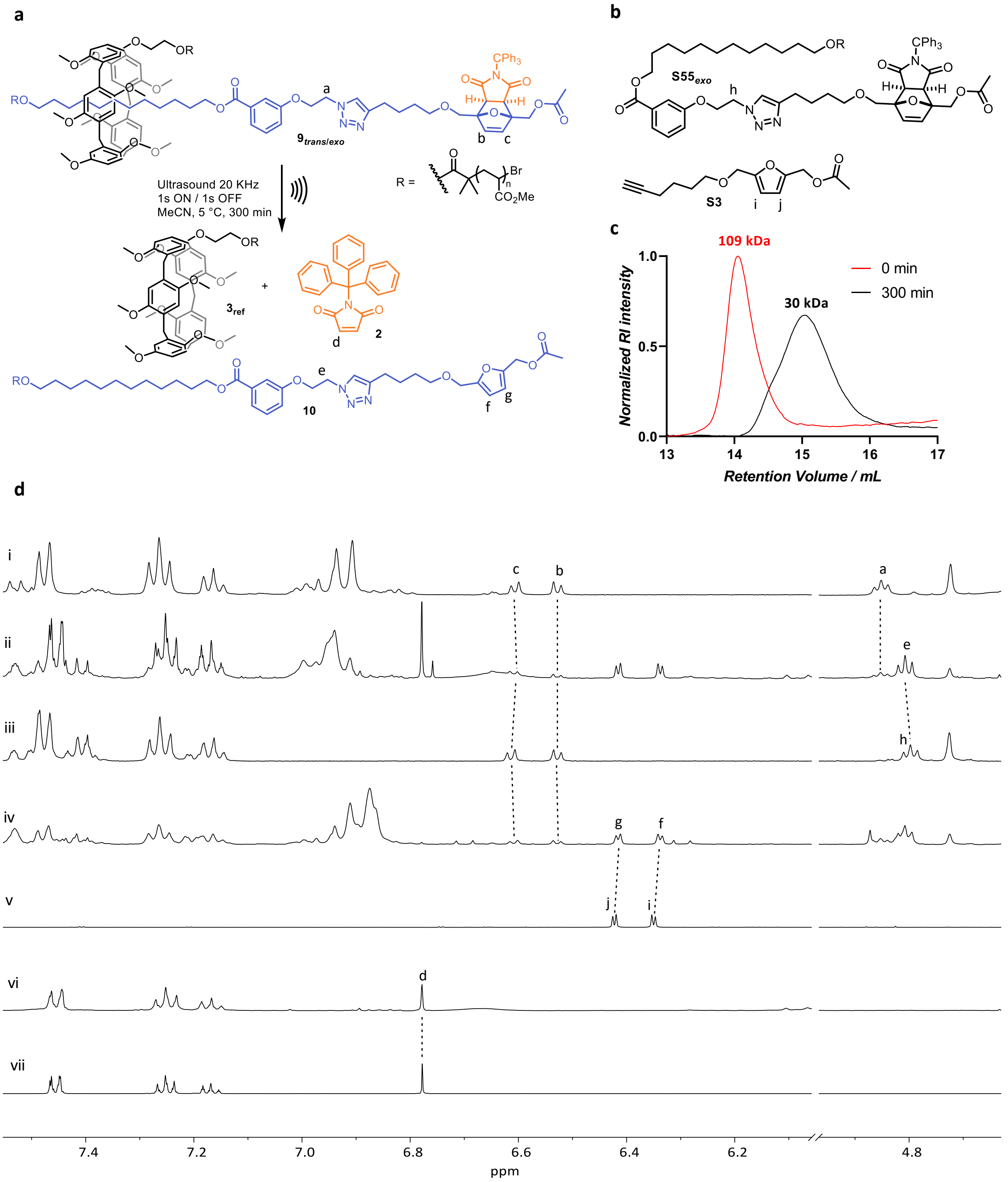

Figure S31. Sonication of polymer **9**$_{trans/exo\text{-}109}$ in MeCN. Sonication of polymer **9**$_{trans/exo}$ affords polymer fragments **3**$_{ref}$ and **10** along with small molecule **2** (a). Reference species **S55**$_{exo}$ and **S3** (b). SEC traces of polymer **9**$_{trans/exo\text{-}109}$ (c) with M_n values before (red) and after (black) sonication. Partial NMR (400 MHz, Acetone-d_6, 298 K) spectra comparison (d) of the pre-sonication polymer **9**$_{trans/exo\text{-}109}$ (i), post-sonication polymer before being washed with methanol (ii), reference polymer **S55**$_{exo\text{-}112}$ (iii), post-sonication polymer after being washed with methanol (iv), reference compound **S3** (v), concentrated methanol washings (vi), and reference compound **2** (vii).

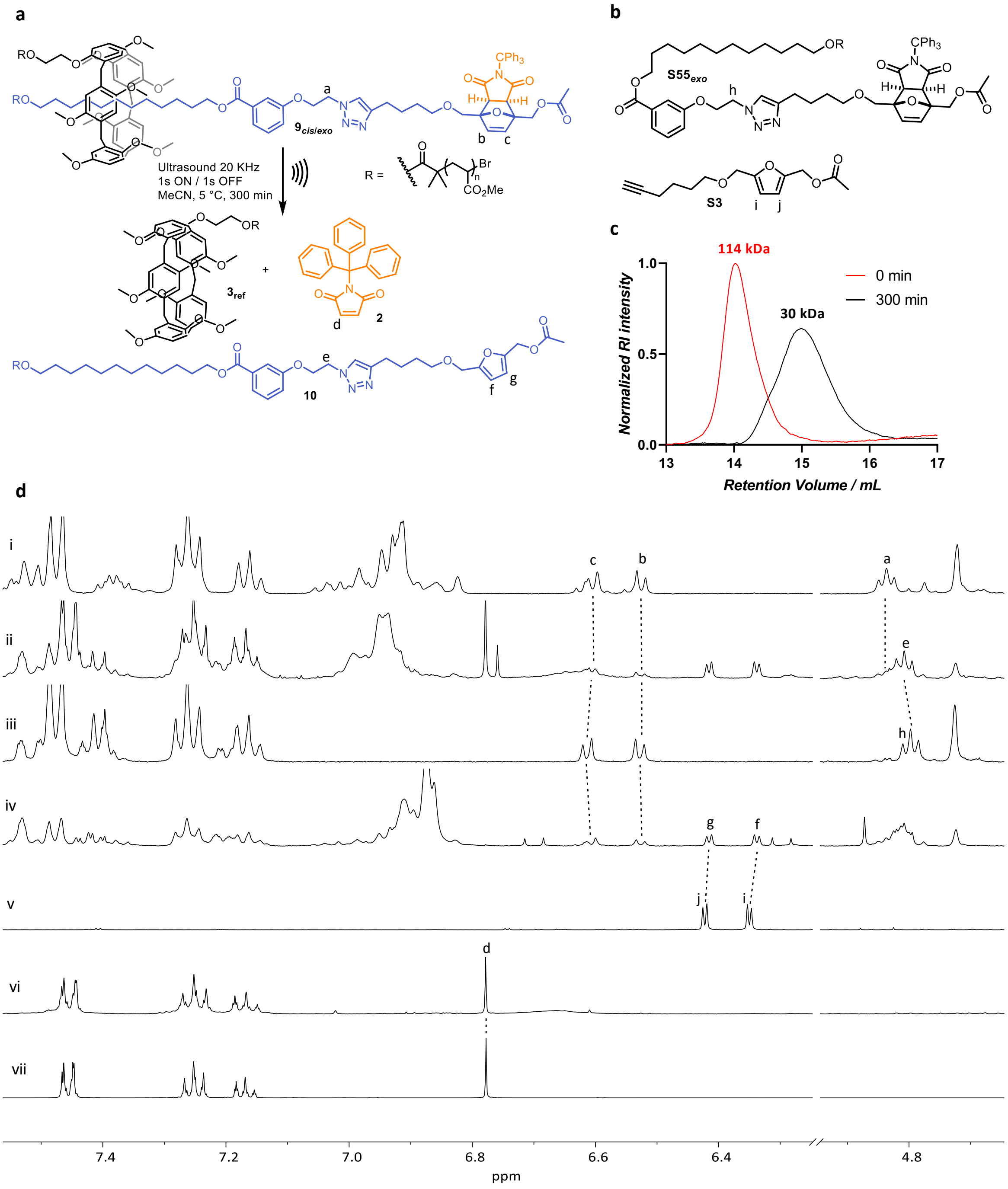

Figure S32. Sonication of polymer $\mathbf{9}_{cis/exo\text{-}114}$ in MeCN. Sonication of polymer $\mathbf{9}_{cis/exo}$ affords polymer fragments $\mathbf{3}_{ref}$ and **10** along with small molecule **2** (a). Reference species $\mathbf{S55}_{exo}$ and **S3** (b). SEC traces of polymer $\mathbf{9}_{cis/exo\text{-}114}$ (c) with M_n values before (red) and after (black) sonication. Partial NMR (400 MHz, Acetone-d_6, 298 K) spectra comparison (d) of the pre-sonication polymer $\mathbf{9}_{cis/exo\text{-}114}$ (i), post-sonication polymer before being washed with methanol (ii), reference polymer $\mathbf{S55}_{exo\text{-}112}$ (iii), post-sonication polymer after being washed with methanol (iv), reference compound **S3** (v), concentrated methanol washings (vi), and reference compound **2** (vii).

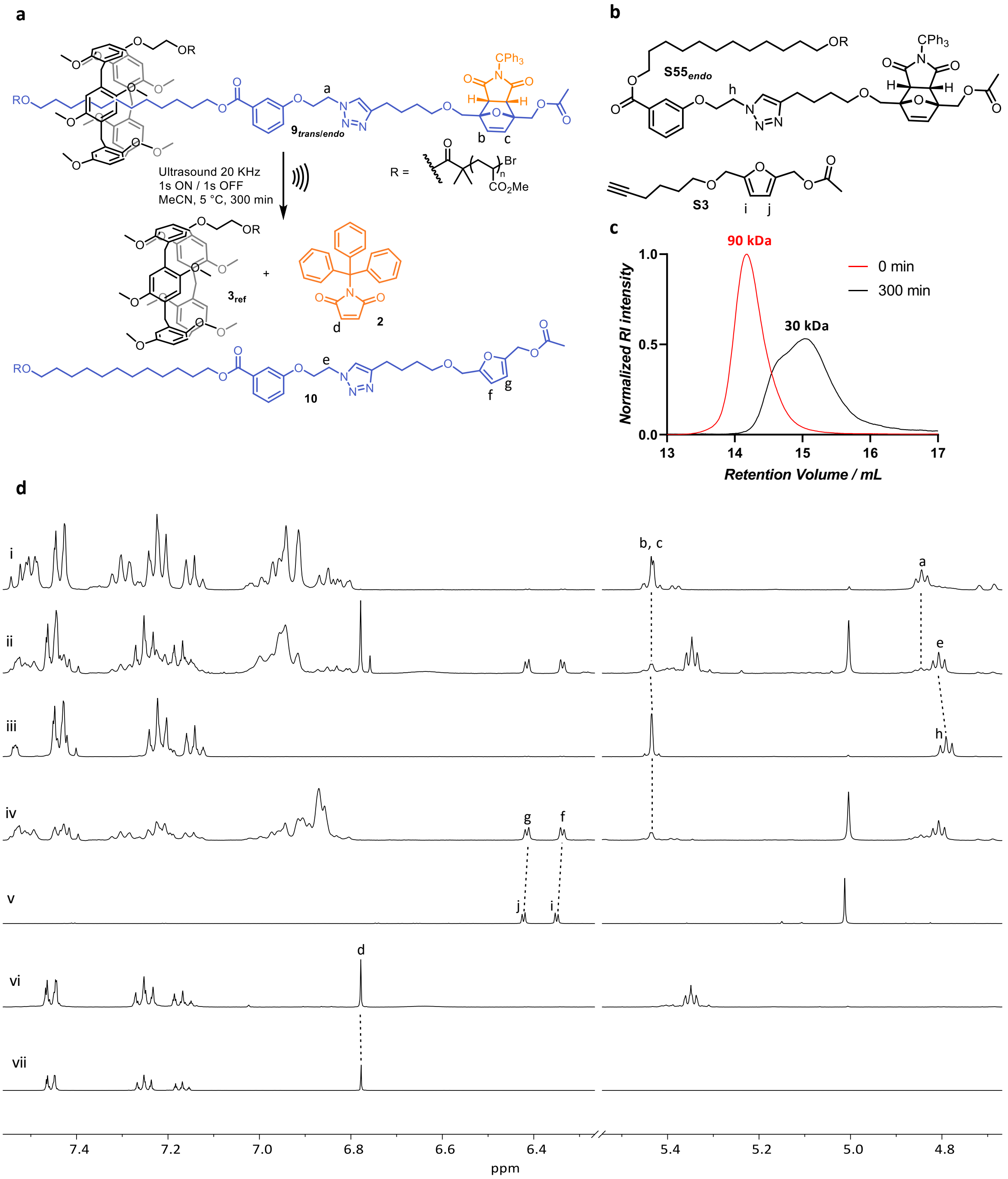

Figure S33. Sonication of polymer **9***trans/endo***-90** in MeCN. Sonication of polymer **9***trans/endo* affords polymer fragments **3**ref and **10** along with small molecule **2** (a). Reference species **S55***endo* and **S3** (b). SEC traces of polymer **9***trans/endo***-90** (c) with M_n values before (red) and after (black) sonication. Partial NMR (400 MHz, Acetone-d_6, 298 K) spectra comparison (d) of the pre-sonication polymer **9***trans/endo***-90** (i), post-sonication polymer before being washed with methanol (ii), reference polymer **S55***endo***-65** (iii), post-sonication polymer after being washed with methanol (iv), reference compound **S3** (v), concentrated methanol washings (vi), and reference compound **2** (vii).

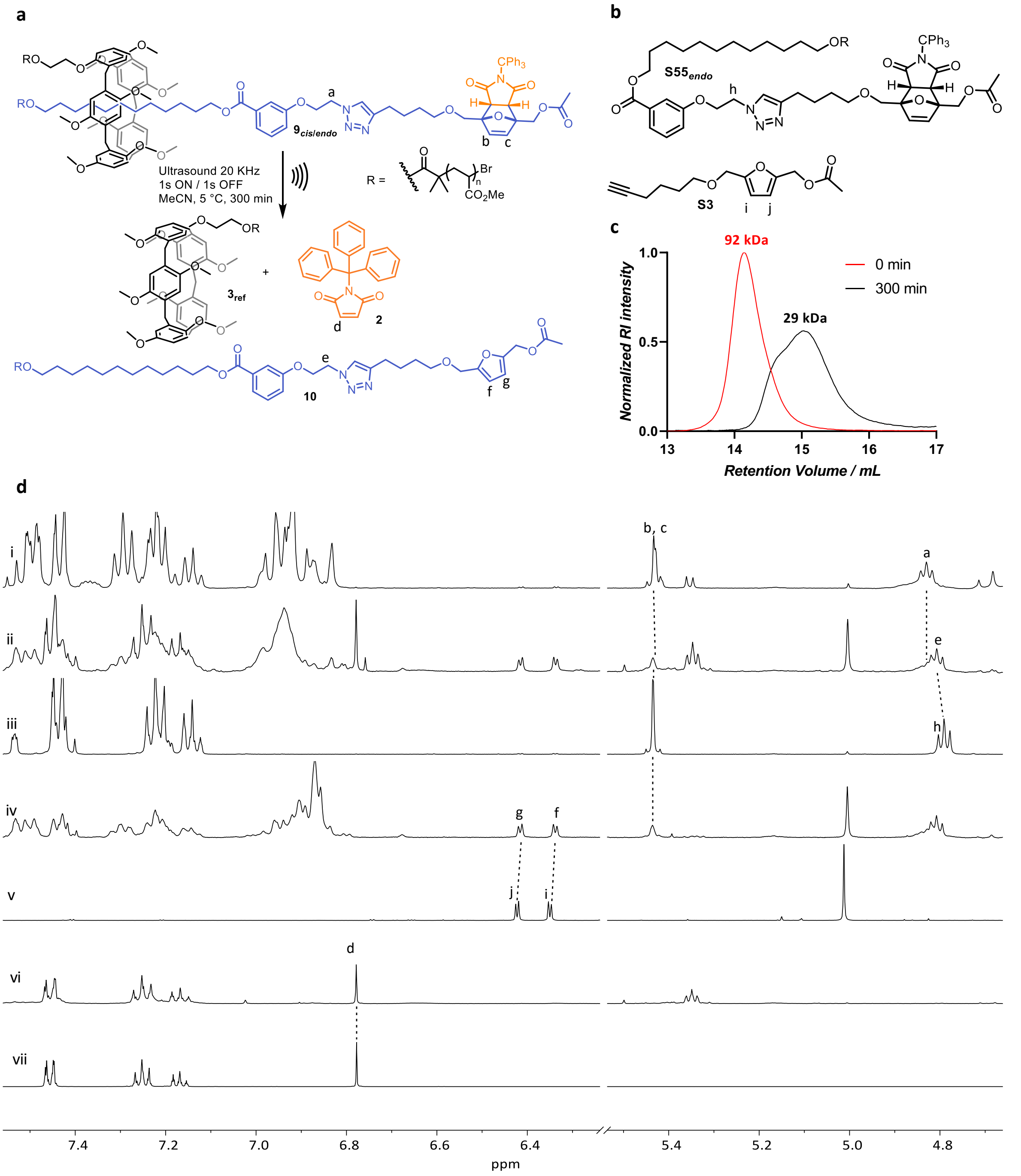

Figure S34. Sonication of polymer **9***cis/endo*-**92** in MeCN. Sonication of polymer **9***cis/endo* affords polymer fragments **3**ref and **10** along with small molecule **2** (a). Reference species **S55***endo* and **S3** (b). SEC traces of polymer **9***cis/endo*-**92** (c) with M_n values before (red) and after (black) sonication. Partial NMR (400 MHz, Acetone-d_6, 298 K) spectra comparison (d) of the pre-sonication polymer **9***cis/endo*-**92** (i), post-sonication polymer before being washed with methanol (ii), reference polymer **S55***endo*-**65** (iii), post-sonication polymer after being washed with methanol (iv), reference compound **S3** (v), concentrated methanol washings (vi), and reference compound **2** (vii).

6.4 Sonication of 3-Cargo Polymers, $\mathbf{1_3}$, and 5-Cargo Polymers, $\mathbf{1_5}$

Sonication of polymers $\mathbf{1_3a}_{-210}$, $\mathbf{1_3b}_{-142}$, $\mathbf{1_3b}_{-171}$, $\mathbf{1_3b}_{-178}$, $\mathbf{1_3c}_{-174}$, $\mathbf{1_3d}_{-89}$, $\mathbf{1_5}_{-60}$, $\mathbf{1_5}_{-165}$, and $\mathbf{1_5}_{-215}$ (all *trans* isomers with regards to the rotaxane structure and mixtures, to varying extents, of *exo* and *endo* isomers with regards to the incorporated Diels-Alder structures), using the methodology described in the general procedure (see *Section 6.1*) and with the solvent used being acetonitrile, was carried out to determine the extent of activation of the Diels-Alder structures within systems with multiple cargo units. SEC analysis of the sonicated polymers showed complete cleavage (M_n of the post-sonication material was less than half of that of the pre-sonication polymer).

Comparison of the ^{1}H NMR spectra of pre- and post-sonication polymers showed that all materials demonstrated the desired retro Diels-Alder reaction upon mechanical force application; this is, as with the 1-cargo systems, evidenced by clear formation of furan species along with the respective maleimide group while the intact Diels-Alder structure clearly decreased in relative intensity. Notably, the furan groups resultant from the desired retro Diels-Alder reaction demonstrate visibly different environments (by ^{1}H NMR spectroscopy) depending on whether they are 'internal' (the 2 or 4 units closest to the rotaxane's macrocycle in the pre-sonication 3- and 5-cargo structures respectively) or 'terminal' (the final unit furthest from the rotaxane's macrocycle in the pre-sonication structures). Using this difference, the extent of activation for internal and terminal Diels-Alder units can be calculated separately. Similar values in this case indicate an individual mechanophore can release all cargo units during mechanical activation. Comparisons of the post-sonication ^{1}H NMR spectra with that of a reference polymer demonstrate a large quantity of rotaxanes are also left entirely intact.

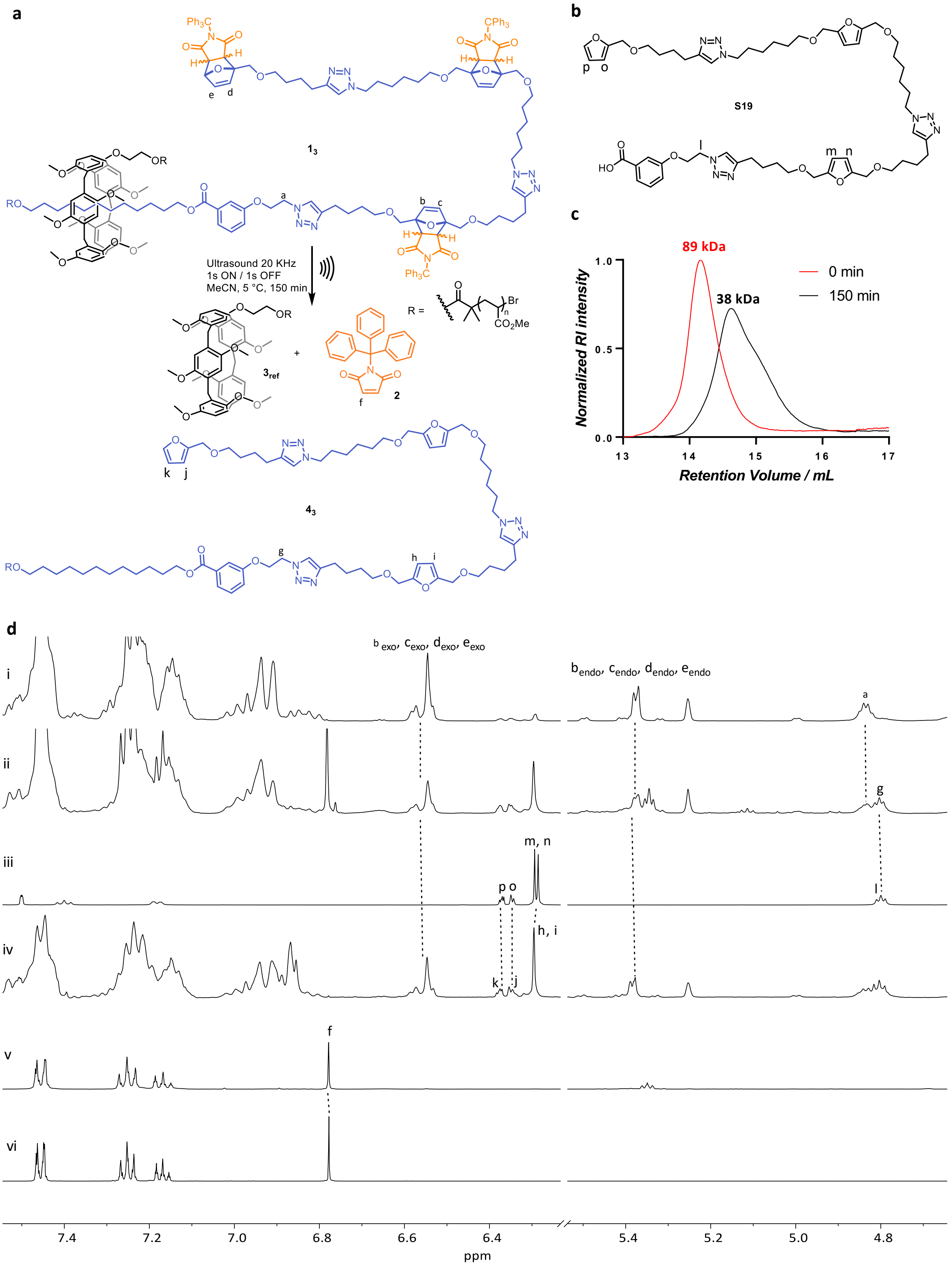

Figure S35. Sonication of polymer **1_3d$_{-89}$** in MeCN. Sonication of polymer **1_3** affords polymer fragments **3_{ref}** and **4_3** along with small molecule **2** (a). Reference compound **S19** (b). SEC traces of polymer **1_3d$_{-89}$** (c) with M_n values before (red) and after (black) sonication. Partial NMR (400 MHz, Acetone-d_6, 298 K) spectra comparison (d) of the pre-sonication polymer **1_3d$_{-89}$** (i), post-sonication polymer before being washed with methanol (ii), reference compound **S19** (iii), post-sonication polymer after being washed with methanol (iv), concentrated methanol washings (v) and reference compound **2** (vi).

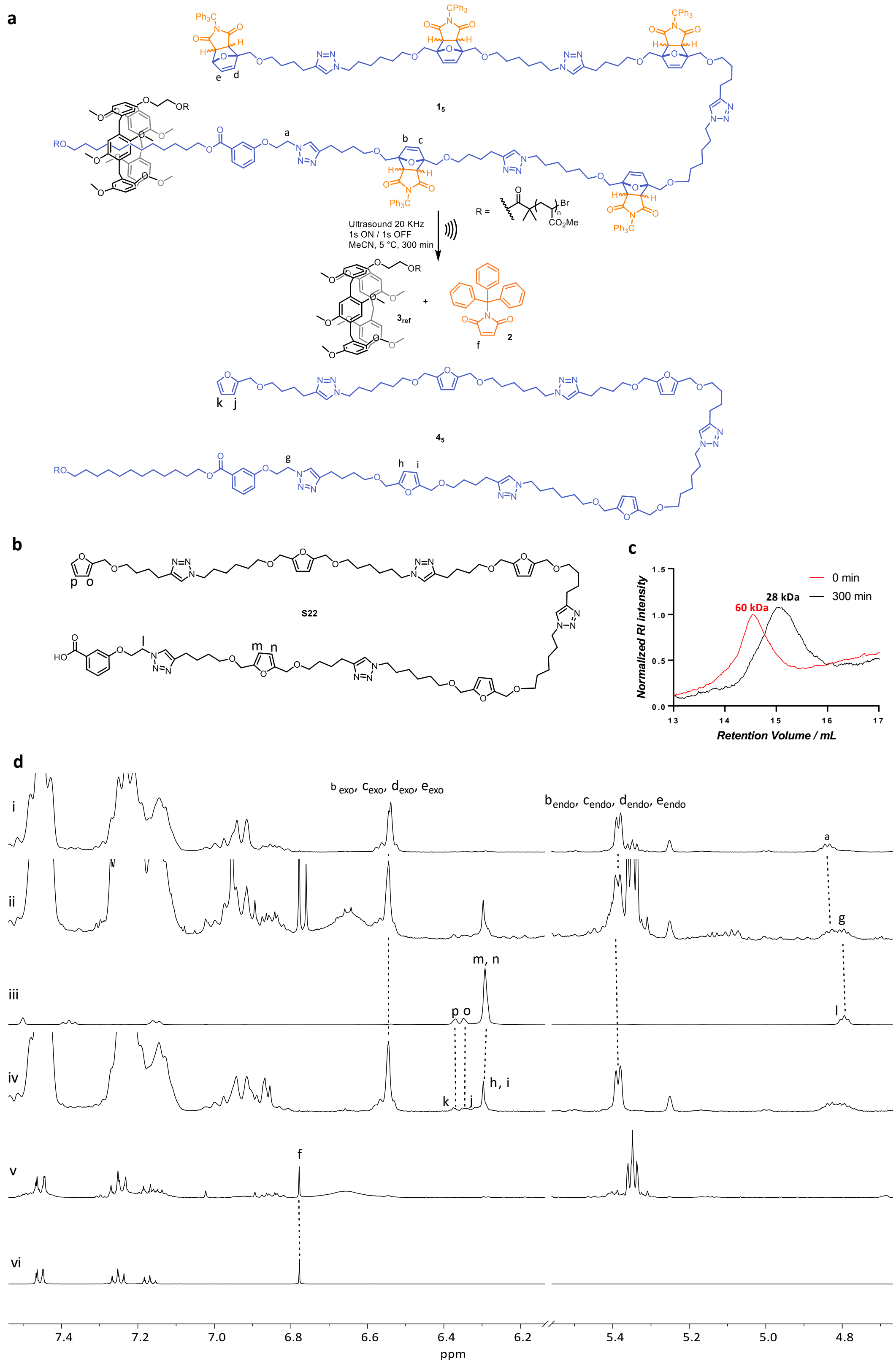

Figure S36. Sonication of polymer $\mathbf{1}_{5\text{-}60}$ in MeCN. Sonication of polymer $\mathbf{1}_5$ affords polymer fragments $\mathbf{3}_{\mathbf{ref}}$ and $\mathbf{4}_5$ along with small molecule **2** (a). Reference compound **S22** (b). SEC traces of polymer $\mathbf{1}_{5\text{-}60}$ (c) with M_n values before (red) and after (black) sonication. (d) Partial NMR (400 MHz, Acetone-d_6, 298 K) spectra comparison (d) of the pre-sonication polymer $\mathbf{1}_{5\text{-}60}$ (i), post-sonication polymer before being washed with methanol (ii), reference compound **S22** (iii), post-sonication polymer after being washed with methanol (iv), concentrated methanol washings (v) and reference compound **2** (vi).

6.5 Dethreading of the Macrocycle after Complete Cargo Release

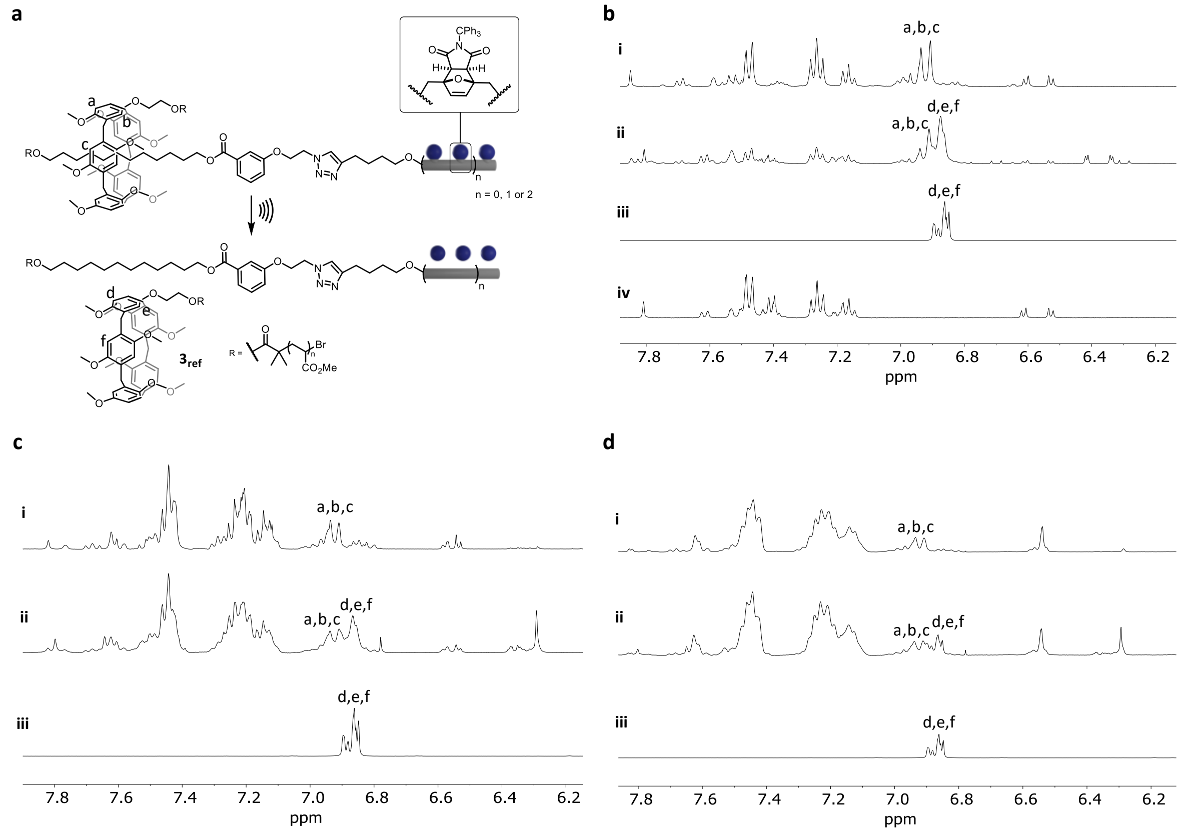

Figure S37. Sonication of the 1-, 3-, and 5-cargo systems can lead to the release of all cargo units on a single mechanophore which in turn results in the dethreading of the macrocycle (polymer $\mathbf{3_{ref}}$) from the axle (a). Partial ^{1}H NMR (400 MHz, Acetone-d$_6$, 298 K) spectra (b) of pre-sonication polymer $\mathbf{9}_{\mathbf{\mathit{trans/exo}}\text{-}\mathbf{109}}$ (i), the same polymer post-sonication after being washed with methanol (ii), polymer $\mathbf{3_{ref}}$ (iii) and polymer $\mathbf{S55}_{\mathit{exo}\text{-}\mathbf{112}}$ (iv). Partial ^{1}H NMR (400 MHz, Acetone-d$_6$, 298 K) spectra (c) of pre-sonication polymer $\mathbf{1_3a_{\text{-}210}}$ (i), the same polymer post-sonication after being washed with methanol (ii) and polymer $\mathbf{3_{ref}}$ (iii). Partial ^{1}H NMR (400 MHz, Acetone-d$_6$, 298 K) spectra of pre-sonication polymer $\mathbf{1_{5\text{-}215}}$ (i), the same polymer post-sonication after being washed with methanol (ii) and polymer $\mathbf{3_{ref}}$ (iii).

For the 1-, 3-, and 5-cargo mechanophores, complete activation of all loaded cargo units allows for the dethreading of the macrocycle from the axle. The ^{1}H NMR spectra of the free thread and the thread when in the rotaxane structure are similar for all useful regions (*Figure S37b-ii*, *b-iv*). Fortunately, the aromatic peaks in the pillar[5]arene macrocycle show a slightly different chemical shift between the free structure and the threaded species; both of these species can be observed after activation of the mechanophores during sonication (*Figure S37c*, *d*) indicating that dethreading has occurred.

6.6 Sonication of Alternative-Cargo Polymers – Drug-Containing-Cargo Release

Sonication of rotaxane polymer **11**$_{\textbf{-127}}$**,** using the methodology described in the general procedure (see *Section 6.1*) and with the solvent used being acetonitrile, was carried out to determine the extent of activation of the Diels-Alder cargo structure wherein the released maleimide species was attached through a dipeptide linker to the anticancer drug doxorubicin.

Comparison of the ^{1}H NMR spectra of pre- and post-sonication polymer showed that the desired retro-Diels-Alder reaction indeed occurred upon mechanical force application; this is evidenced by clear formation of the desired furan species along with the respective maleimide group while the intact *exo* and *endo* Diels-Alder structures clearly decreased in relative intensity. Comparison to reference species also demonstrated that the post-sonication material had some intact rotaxane species remaining. Importantly, washing of the post-sonication polymer with methanol allowed us to remove and analyse any contained small molecules through NMR spectroscopy; in the ^{1}H NMR spectrum of these concentrated MeOH washings are clear peaks of the intact cargo including, but not limited to, peaks of the N-(triphenylmethyl)maleimide (*Figure S38*, $H_{g\text{-}j}$), peptide linker (*Figure S38*, $H_{k\text{-}m}$) and doxorubicin (*Figure S38*, $H_{n\text{-}r}$) structures which all match well with synthesised reference compound **12**. Analysis of these same sample by mass spectrometry also revealed evidence of cargo release with the desired isotopic distribution being observed (*Figure S39*).

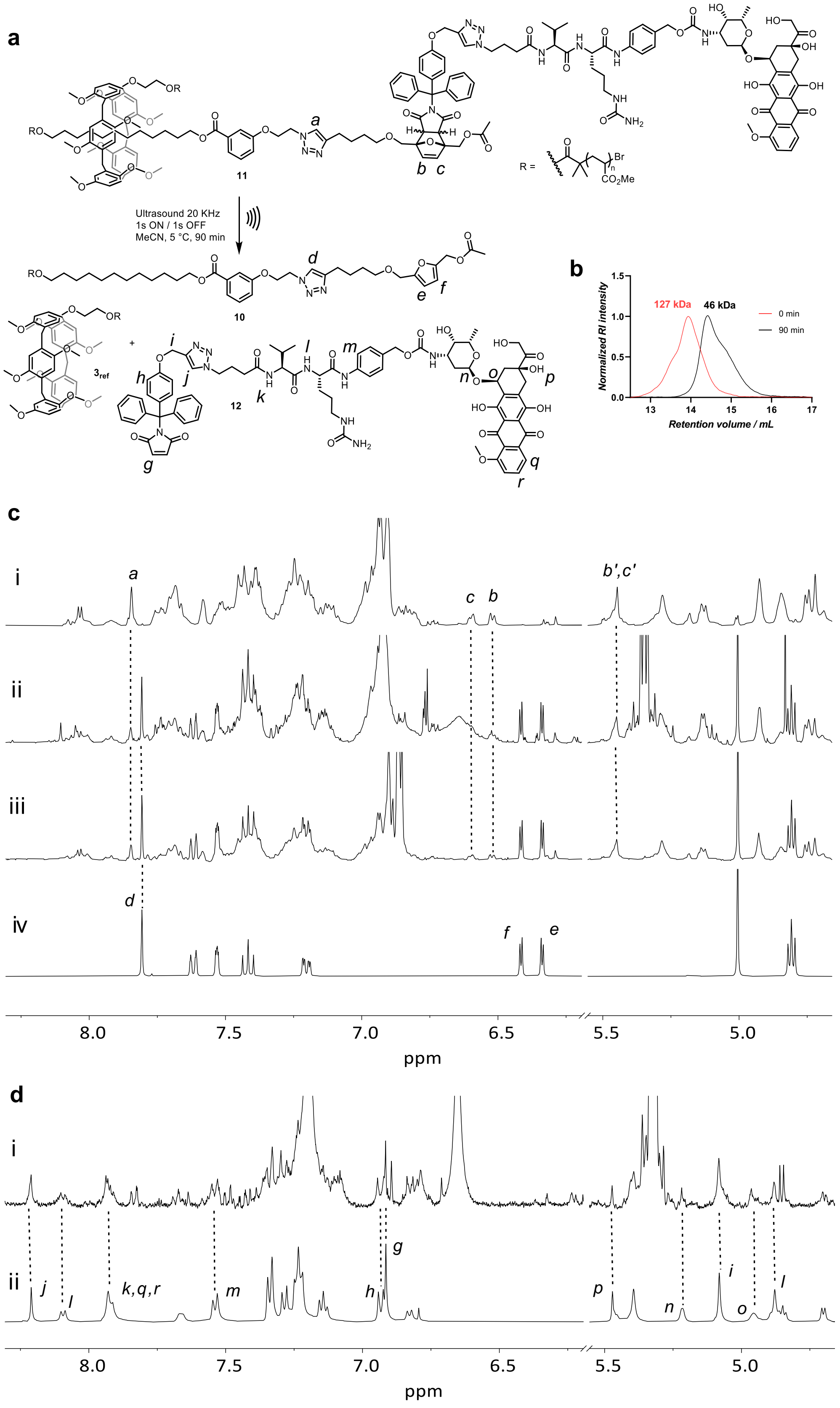

Figure S38. Sonication of polymer **11**$_{-127}$ in MeCN. Sonication of polymer **11** affords polymer fragments **3**$_{ref}$ and **10** along with small molecule **12** (a). SEC traces of polymer **11**$_{-127}$ (b) with M_n values before (red) and after (black) sonication. Partial NMR (400 MHz, Acetone-d_6, 298 K) spectra comparison (c) of the pre-sonication polymer **11**$_{-127}$ (i), post-sonication polymer before being washed with methanol (ii), post-sonication polymer after being

washed with methanol (iii), and reference polymer **S60-80** (iv). Partial NMR (400 MHz, DMSO-d_6, 298 K) spectra comparison (d) of concentrated methanol washings (i) and reference **12** (ii).

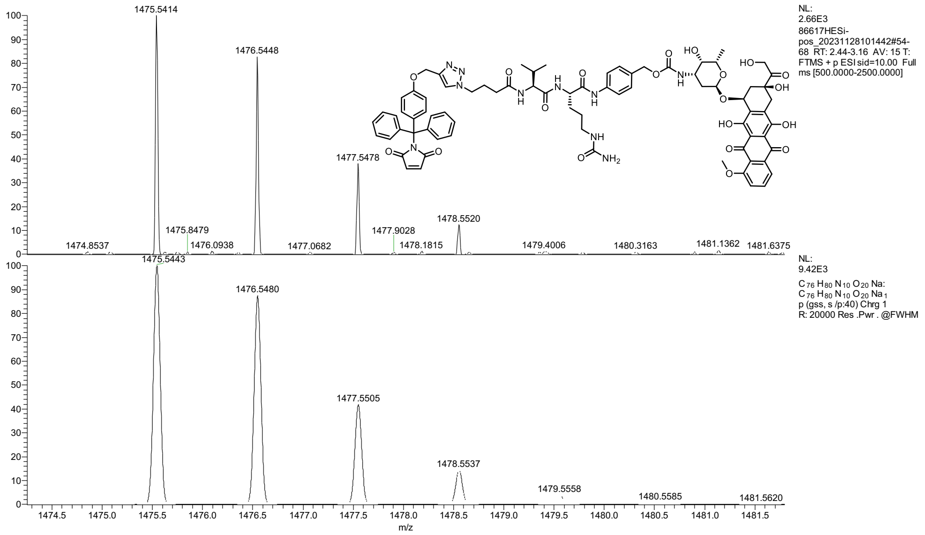

Figure S39. Mass spectrometry analysis of the concentrated methanol washings from the post-sonication polymer **11-127**; the measured isotopic pattern is found for the correct release of the drug-containing-cargo maleimide molecule. Top: Measured isotopic distribution for $C_{76}H_{80}N_{10}O_{20}Na$ ($[M+Na]^+$, +ESI). Bottom: Simulated isotopic distribution for $C_{76}H_{80}N_{10}O_{20}Na^+$.

6.7 Sonication of Alternative-Cargo Polymers – N-(1-pyrenyl)maleimide-Cargo Release

Sonication of polymer **13-119,** using the methodology described in the general procedure (see *Section 6.1*) and with the solvent used being acetonitrile, was carried out to determine the extent of activation of the Diels-Alder cargo structure wherein the released cargo species was the non-fluorescent N-(1-pyrenyl)maleimide species. This small molecule could be extracted from the post-sonication polymer with methanol; concentration of these washings allowed us to react the extracted N-(1-pyrenyl)maleimide with 1-dodecanethiol (0.08 µL, 2 equivalents relative to the pre-sonication polymer) in DMSO-d_6 (1 mL) producing a fluorescent adduct that could then be detected by fluorescence spectroscopy.

Comparison of the ^{1}H NMR spectra of pre- and post-sonication polymer showed that the desired retro-Diels-Alder reaction indeed occurred upon mechanical force application; this is evidenced by clear formation of the desired furan species along with the respective maleimide group while the intact Diels-Alder structure clearly decreased in relative intensity. Comparison to reference species also demonstrated that the post-sonication material had some intact rotaxane species remaining. Importantly, analysis of the concentrated methanol washings of the post-sonication polymer allowed us to observe the release of the N-(1-pyrenyl)maleimide cargo through ^{1}H NMR spectroscopy. It appears that, also, in some instances of mechanical force being applied to the rotaxane structure we observed the macrocycle simply passing over the Diels-Alder structure; this is evidenced by an upfield-shifted triazole proton (*Figure S40*, H_j) found in reference polymer **S59-77**.

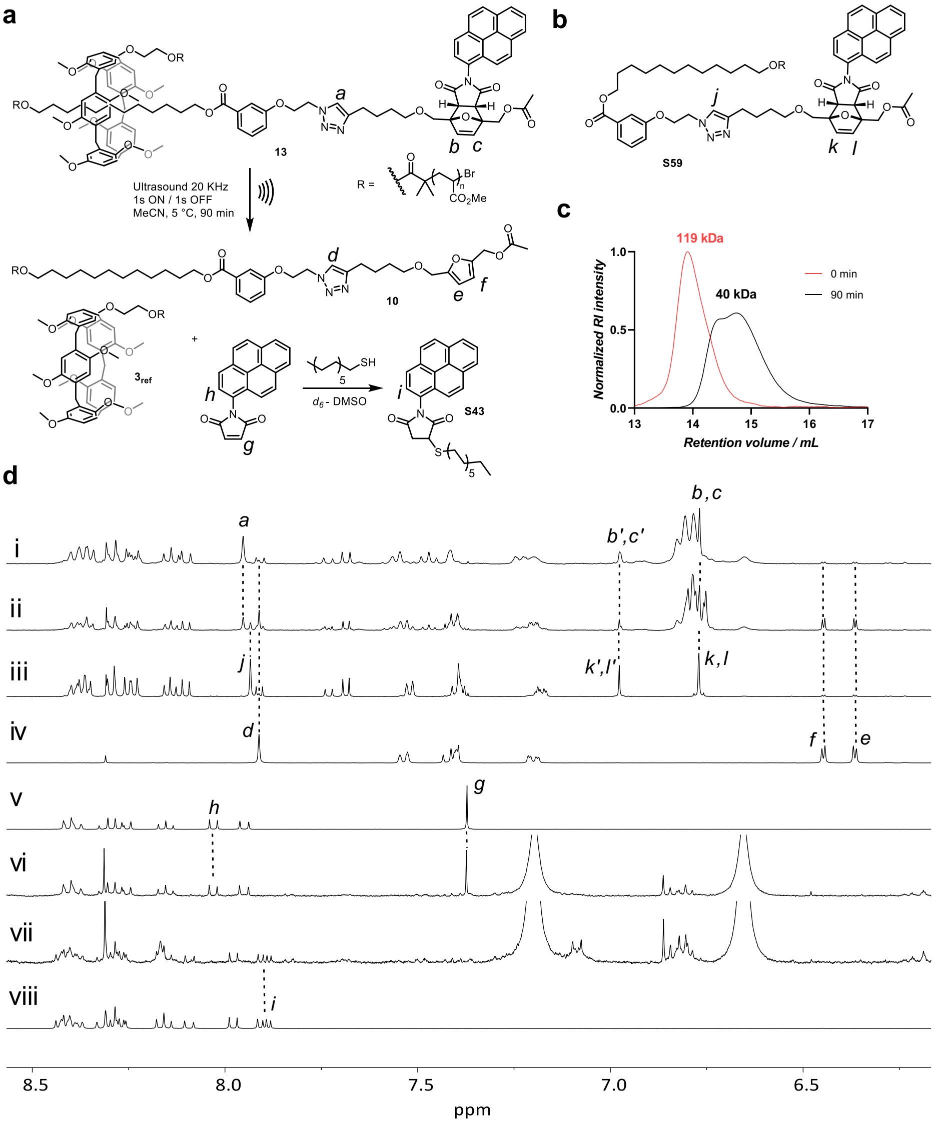

Figure S40. Sonication of polymer **13**$_{\text{-}119}$ in MeCN. Sonication of polymer **13** affords polymer fragments **3**$_{\mathbf{ref}}$ and **10** along with small molecule N-(1-pyrenyl)maleimide which reacted with alkane thiol to form **S43** (a). Reference species **S59** (b). SEC traces of polymer **13**$_{\text{-}119}$ (c) with M_n values before (red) and after (black) sonication. Partial NMR (400 MHz, DMSO-d_6, 298 K) spectra comparison (d) of the pre-sonication polymer **13**$_{\text{-}119}$ (i), post-sonication polymer after being washed with methanol (ii), reference polymer **S59**$_{\text{-}77}$ (iii), reference polymer **10**$_{\text{-}80}$ (iv), N-(1-pyrene)maleimide (v), concentrated methanol washings (vi), concentrated methanol washings mixed with 1-dodecanethiol (vii) and reference **S43** (viii).

The fluorescence spectrum (*Figure S41t*) of the concentrated methanol washings from the post-sonication polymer **13**$_{\text{-}119}$ in DMSO-d_6 showed relatively weak emission which perhaps indicates the formed N-(1-pyrenyl)maleimide partially decomposed due to the presence of radical species produced under harsh conditions of the sonication experiment. Nonetheless addition of 1-dodecanethiol to this same sample caused the emission intensity to increase dramatically indicating formation of the fluorescent adduct, **S43**. After diluting the sample by 20 times the original volume with DMSO-d_6, the emission went back to the intensity of that before addition of 1-dodecanethiol, which implied an amount of the released N-(1-pyrenyl)maleimide cargo survived the sonication and was subsequently able to react with 1-dodecanethiol to form **S43**.

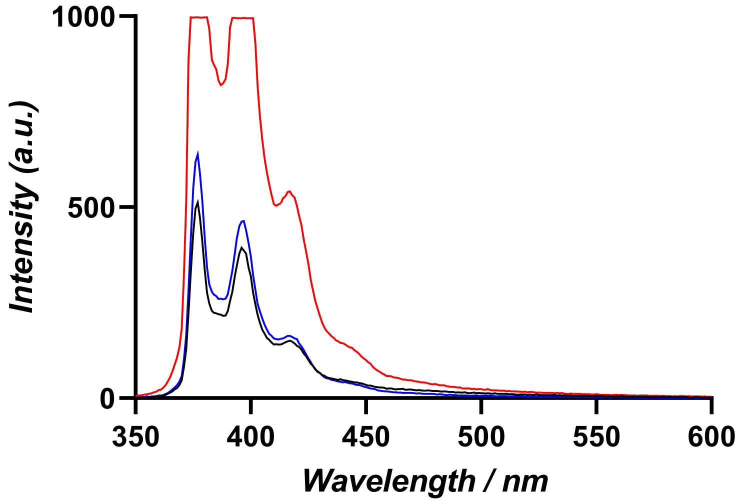

Figure S41. Fluorescence spectra, taken in DMSO-d_6, of the concentrated methanol washings of the post-sonication polymer **13**$_{-119}$ before (black) and after (red) adding 1-dodecanethiol, and after further diluting, by 20 times the original volume, the sample post-addition of 1-dodecanethiol (blue). The excitation wavelength was 342 nm.

6.7.1 Sonication of Polymer 13_{-119} in the Presence of 1-Dodecanethiol

Additionally, the sonication of polymer **13-119** was carried out, using the methodology described in the general procedure (see *Section 6.1*) and with the solvent used being a mixture of acetonitrile and water (in a 9 : 1 ratio respectively), in the presence of 1-dodecanethiol (50 equivalents relative to the polymer **13-119**) in order to see whether the released non-fluorescent N-(1-pyrenyl)maleimide could form the fluorescent species, **S43**, *in situ*. The usual analysis of the concentrated methanol washings of the post-sonication polymer **13-119** in this case successfully revealed evidence of the desired formed adduct species.

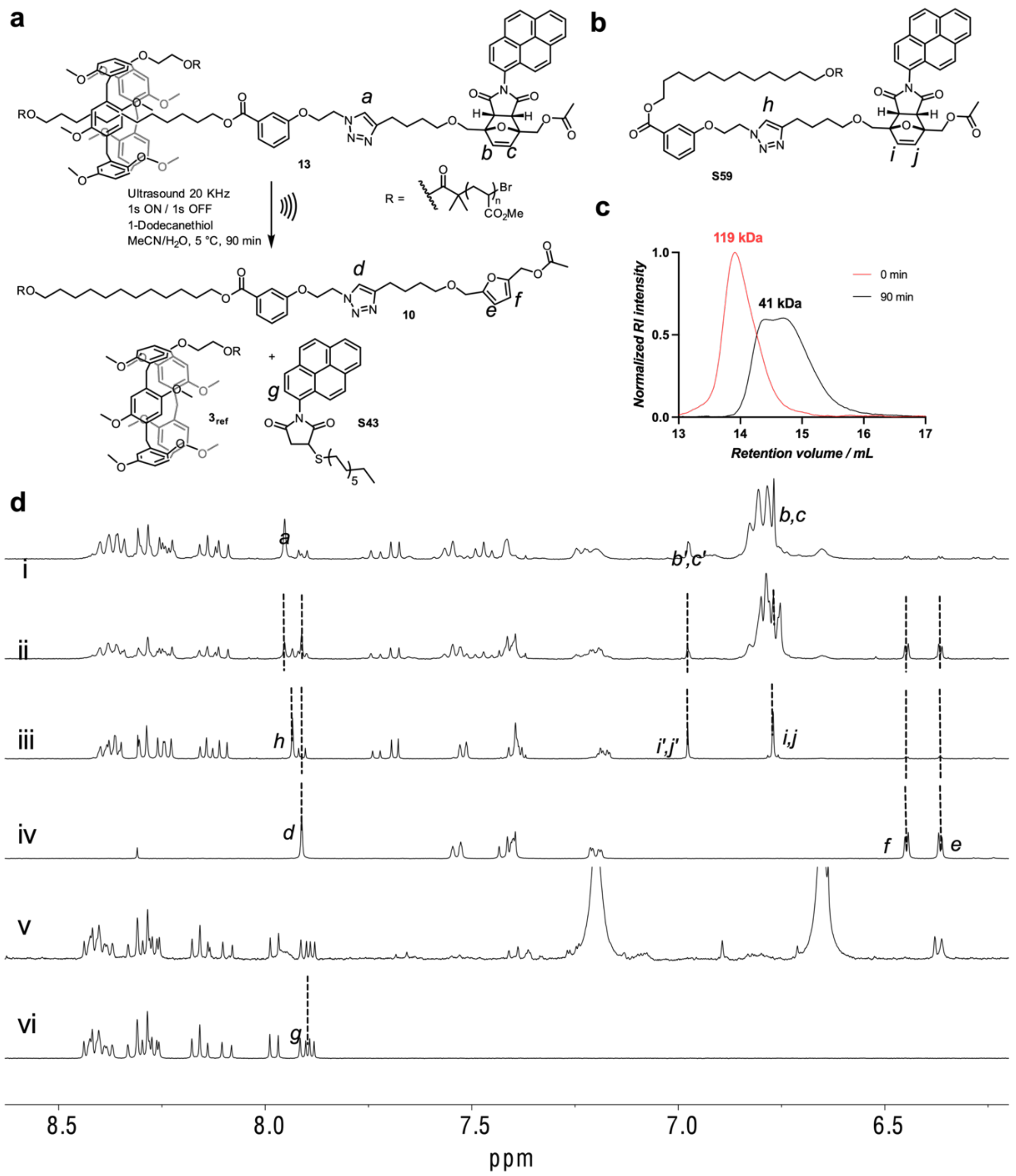

Figure S42. Sonication of polymer **13-119** (20 mg, 0.17 µmol, 1 equiv.) in MeCN/H_2O (9/1) with 1-dodecanethiol (2.0 µL, 8.4 µmol, 50 eq.). Sonication of polymer **13** affords polymer fragments **3ref** and **10** along with small molecule N-(1-pyrenyl)maleimide which reacted with alkane thiol to form **S43** *in situ* (a). Reference species **S59** (b). SEC traces of polymer **13-119** (c) with M_n values before (red) and after (black) sonication. Partial NMR (400 MHz, DMSO-d_6, 298 K) spectra comparison (d) of the pre-sonication polymer **13-119** (i), post-sonication polymer after being washed with methanol (ii), reference polymer **S59-77** (iii), reference polymer **10-80** (iv), concentrated methanol washings (v) and reference **S43** (vi).

6.7.2 Sonication of Control Polymer $\mathbf{S59}_{\mathbf{-77}}$

Sonication of polymer **S59**$_{\mathbf{-77}}$, using the methodology described in the general procedure (*Section 6.1*) and with the solvent used being acetonitrile, was carried out in order to show that activation of the mechanophore in polymer **13**$_{\mathbf{-119}}$ under sonication conditions was due to mechanical force and not other effects. SEC analysis of the sonicated polymers showed complete cleavage (M_n of the post-sonication material was less than half of that of the pre-sonication polymer). Comparison of ^{1}H NMR spectra of the pre- and post-sonication polymers show only a small inconsequential amount of furan species (1%) having formed during the sonication procedure along with a respective quantity of maleimide; this shows that the species cannot undergo the desired retro Diels-Alder reaction to any meaningful extent without the polymer-attached rotaxane actuator used in polymer **13**$_{\mathbf{-119}}$.

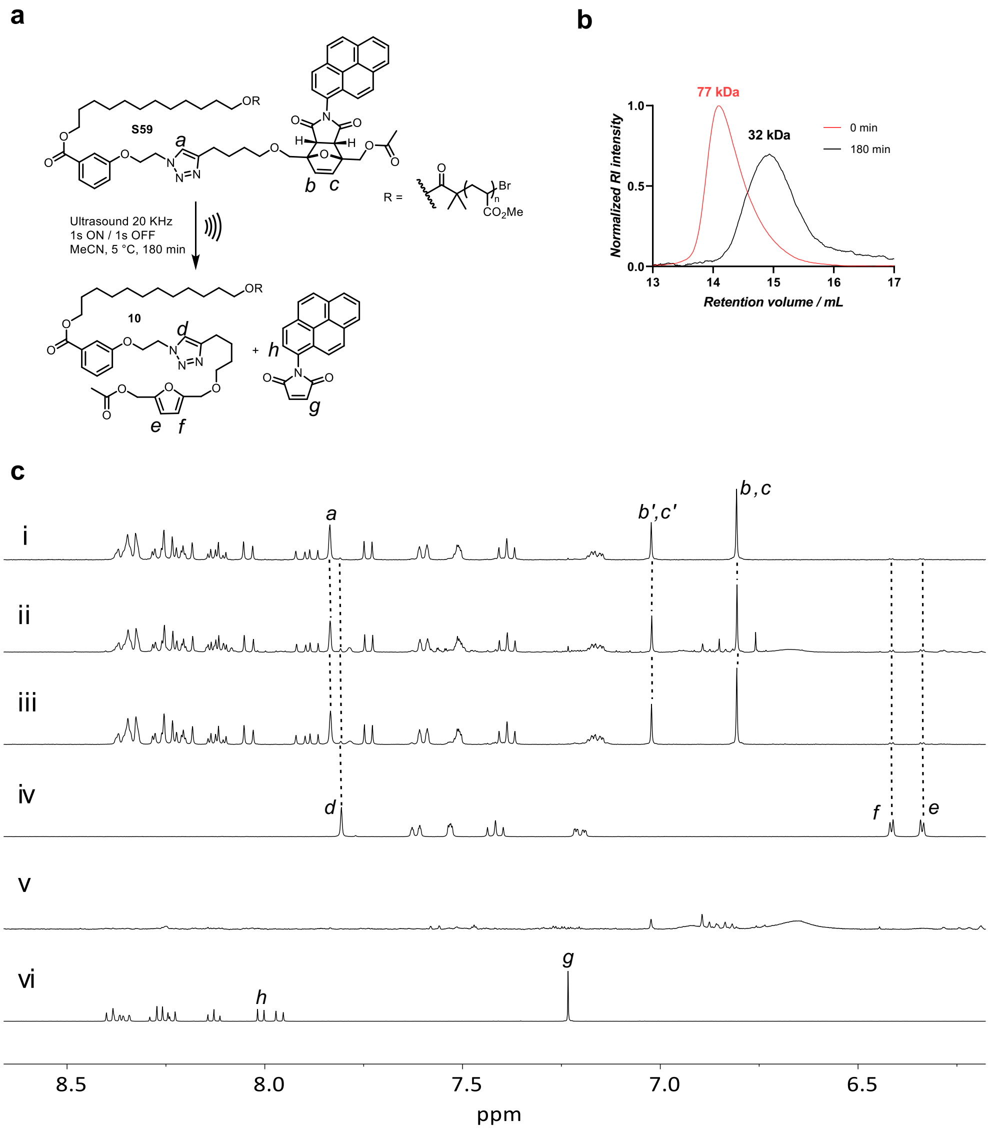

Figure S43. Sonication of polymer **S59**$_{\mathbf{-77}}$ in MeCN. Sonication of polymer **S59** affords polymer fragment **10** along with small molecule N-(1-pyrenyl)maleimide (a). SEC traces of polymer **S59**$_{\mathbf{-77}}$ (b) with M_n values before (red) and after (black) sonication. Partial NMR (400 MHz, Acetone-d_6, 298 K) spectra comparison (c) of the pre-sonication polymer **S59**$_{\mathbf{-77}}$ (i), post-sonication polymer before being washed with methanol (ii), post-sonication polymer after being washed with methanol (iii), reference polymer **10**$_{\mathbf{-80}}$ (iv), concentrated methanol washings (v), N-(1-pyrenyl)maleimide (vi).

6.8 Sonication of Alternative-Cargo Polymers – Trityl-Cargo Release

Sonication of polymer **14**$_{-124}$**,** using the methodology described in the general procedure (see *Section 6.1*) and with the solvent used being a mixture of THF and H_2O (in a 75 : 1 ratio respectively), was carried out to determine the extent of activation of the stoppering mechanophore structure through release of a cationic trityl-derivative species through cleavage of the appropriate C-N bond. Water was used in the sonication mixture to trap the trityl cation species as the corresponding trityl alcohol derivative; evidence of this formation was shown by analysis of the post-sonication polymer indicating desired bond cleavage (shown by formation of reference polymer species **S61**$_{-72}$). ^{1}H NMR spectroscopy analysis of the concentrated methanol washings from the post-sonication polymer indeed revealed the presence of the formed 4,4'-dimethoxytrityl alcohol.

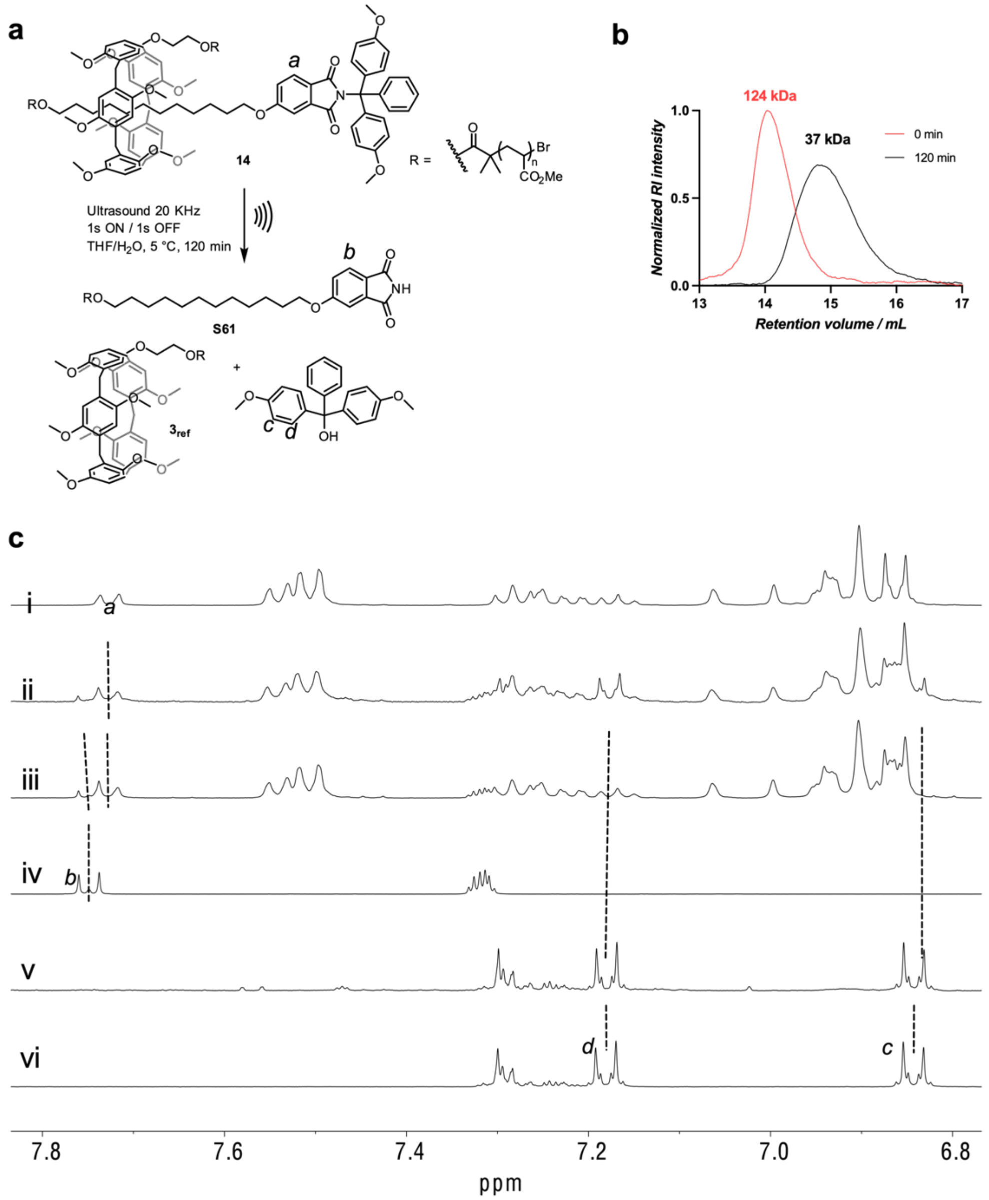

Figure S44. Sonication of polymer **14**$_{-124}$ in THF/H_2O (75/1). Sonication of polymer **14** affords polymer fragments **3**$_{\mathbf{ref}}$ and **S61** along with small molecule 4,4'-dimethoxytrityl alcohol (a). SEC traces of polymer **14**$_{-124}$ (b) with M_n values before (red) and after (black) sonication. Partial NMR (400 MHz, Acetone-d_6, 298 K) spectra comparison (c) of the pre-sonication polymer **14**$_{-124}$ (i), post-sonication polymer before being washed with methanol (ii), post-sonication polymer after being washed with methanol (iii), reference compound **S61**$_{-72}$ (iv), concentrated methanol washings (v), 4,4'-dimethoxytrityl alcohol (vi).

6.8.1 Sonication of Control Polymer, S60-95

Sonication of polymers **S60-95**, using the methodology described in the general procedure (*Section 6.1*) and with the solvent used being a mixture of THF and H_2O (in a 75 : 1 ratio respectively), was carried out in order to show that activation of the mechanophore in polymer **14-124** under sonication conditions was due to mechanical force and not other effects. SEC analysis of the sonicated polymers showed complete cleavage (M_n of the post-sonication material was less than half of that of the pre-sonication polymer). Comparison of ^{1}H NMR spectra of the pre- and post-sonication polymers show no amount of the mechanophore has activated through the cleavage of the C-N bond or otherwise noticeably; this shows that the species cannot undergo the desired bond cleavage to any meaningful extent without the polymer-attached rotaxane actuator used in polymer **14-124**.

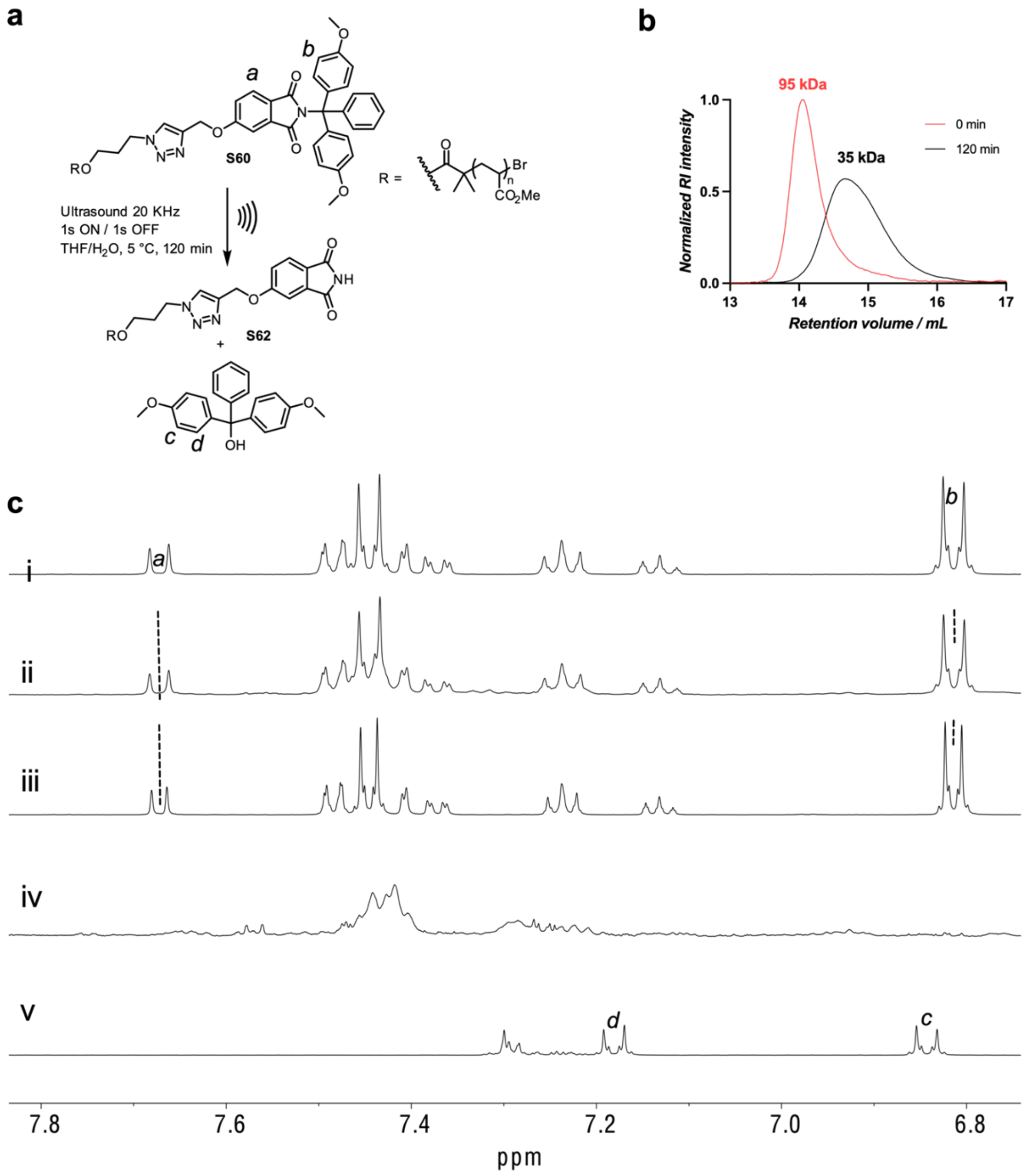

Figure S45. Sonication of polymer **S60-95** in THF/H_2O: 75/1. Sonication of polymer **S60** affords polymer fragment **S62** along with small molecule 4,4'-dimethoxytrityl alcohol (a). SEC traces of polymer **S60-95** (b) with M_n values before (red) and after (black) sonication. Partial NMR (400 MHz, Acetone-d_6, 298 K) spectra comparison (c) of the pre-sonication polymer **S60-95** (i), post-sonication polymer before being washed with methanol (ii), post-sonication polymer after being washed with methanol (iii), concentrated methanol washings (iv), 4,4'-dimethoxytrityl alcohol (v).

7 Activation in Bulk by Compression

7.1 General Procedure for Compression Experiments

The appropriate polymer (30 mg) was formed into a rough spherical shape by hand. The material was placed inbetween the anvils within a standard 13 mm KBr pellet die. 10 tonnes of compressive force was then applied; as the material was compressed, the pressure was relieved gradually by rearrangement of the material so over the course of a period of up to 90 minutes it was ensured that 10 tonnes of force was being continuously applied. The pressure was then released and the flattened material folded in half as many times as possible before being placed back into the pellet die and compressed further. This cyclical process of folding followed by compression was repeated until SEC analysis of the material showed adequate reduction in the M_n (at least 50% of the initial pre-compression M_n). At this point, the material was dissolved in DCM and carefully filtered (0.45 µm PTFE membrane) to remove any metal particulate before being condensed *in vacuo*. The crude polymer material was analysed by ^{1}H NMR spectroscopy before being thoroughly dried once more and subsequently directly washed over with MeOH (at least 5 x 10 mL). The MeOH washings were collected, condensed *in vacuo* and analysed by ^{1}H NMR spectroscopy along with the washed polymer material itself.

7.2 Control Experiment for Activation in Bulk

In order to confirm that activation of the mechanophores using the methodology described in *Section 7.1* was due to mechanical force and not other effects, we carried out control experiments using polymer **S55**$_{\textbf{\textit{exo}}\textbf{-112}}$ where the Diels-Alder unit is located at the end of a polymer chain instead of the centre – additionally, the compound is no longer a rotaxane as there is no encompassing macrocycle unit as is found in the chain-centred mechanophore-containing polymers. Subjecting polymer **S55**$_{\textbf{\textit{exo}}\textbf{-112}}$ to the compression activation methodology showed no decomposition of the mechanophore unit at all, as evidenced in the ^{1}H NMR spectrum by no formation of furan or maleimide peaks along with identical integration of mechanophore peaks relative to polymer peaks both before and after subjection to said methodology.

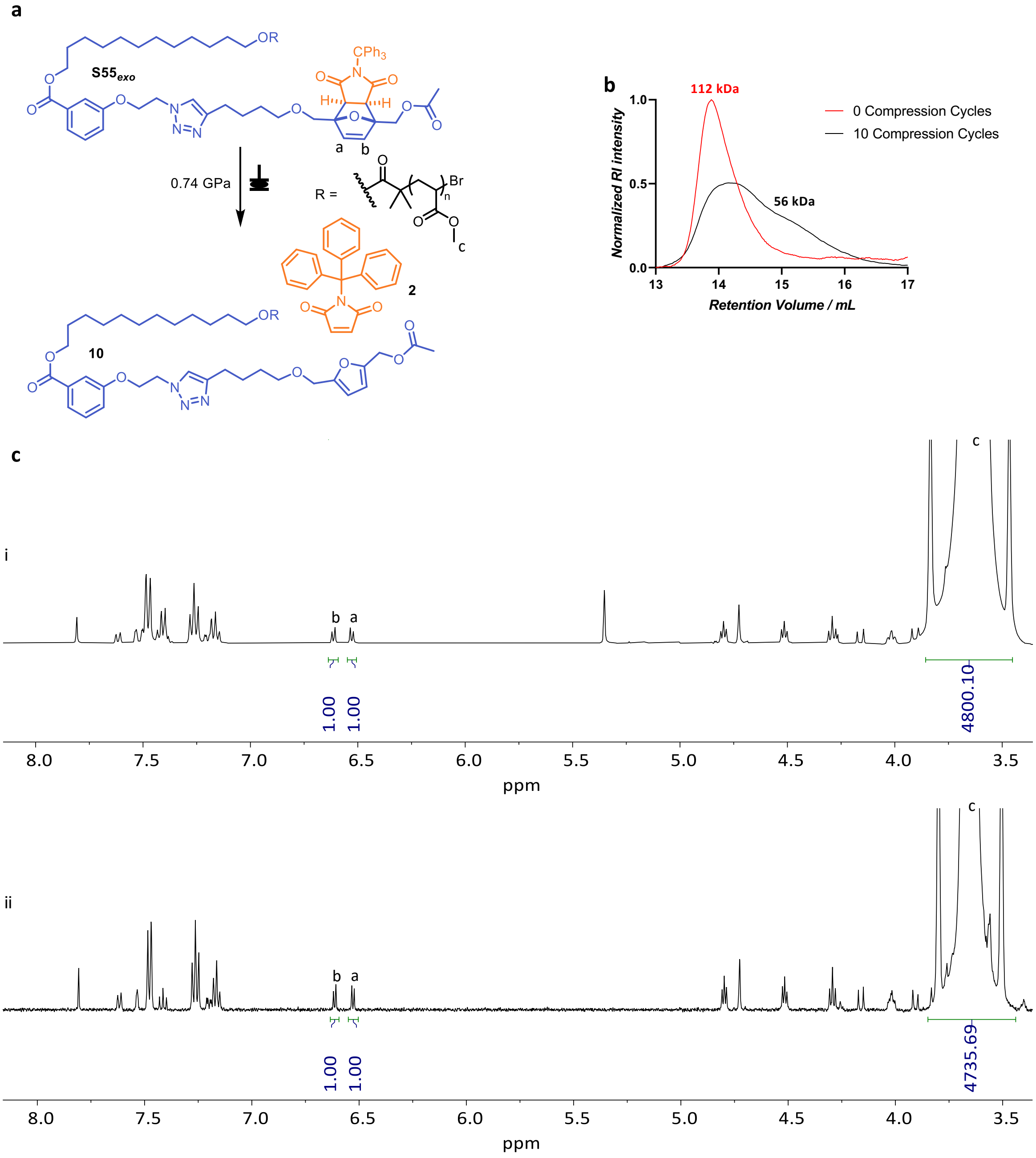

Figure S46. Bulk activation of control polymer **S55**$_{exo\text{-}112}$. Activation of the Diels-Alder structure of **S55**$_{exo}$ would result in polymer **10** and small molecule **2** (a). SEC traces (b) of control polymer **S55**$_{exo\text{-}112}$ with M_n values before (red) and after (black) compression. Partial NMR (500 MHz, Acetone-d_6, 298 K) spectra comparison of polymer **S55**$_{exo\text{-}112}$ (c) before (i) and after compression with the resultant polymer having been washed with MeOH (ii).

7.3 Bulk Activation of 1-, 3-, and 5-Cargo Polymers

Polymers **9***trans/exo***-114** (1-cargo), **1**$_3$**a**$_{-210}$ (3-cargo), **1**$_{5-165}$ (5-cargo) and **1**$_{5-215}$ (5-cargo) were subjected to the general bulk activation methodology described in *Section 7.1*. Analysis of the material post-activation was carried out in an identical manner to that of the sonication of the same polymers, as described in *Section 8.2* and *8.3* for 1-cargo systems and *Section 8.4* for 3-/5-cargo systems.

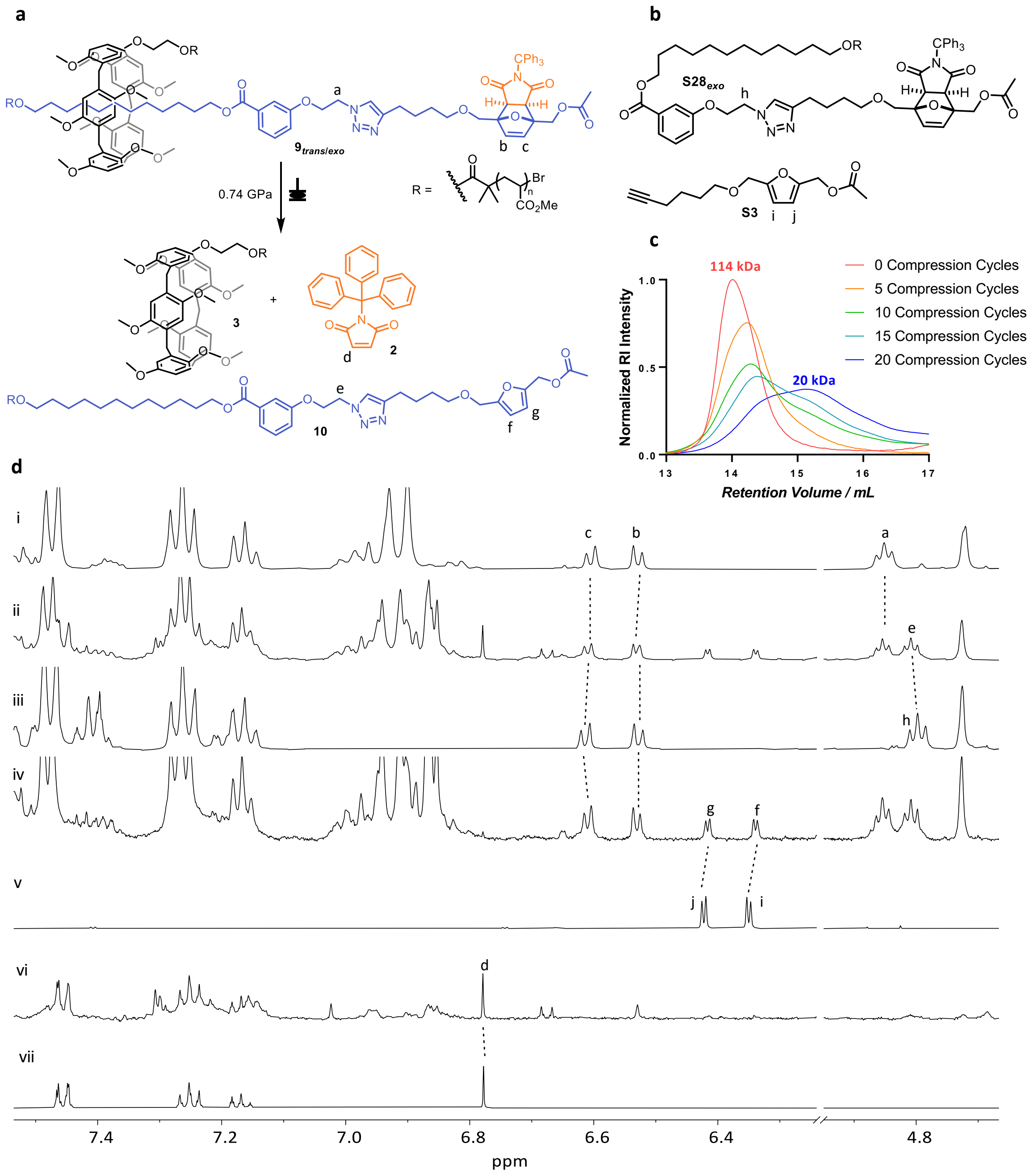

Figure S47. Bulk activation of polymer **9***trans/exo***-114**. Bulk activation of polymer **9***trans/exo* affords polymer fragments **3**$_{ref}$ and **10** along with small molecule **2** (a). Reference species **S55***exo* and **S3** (b). SEC traces of polymer **9***trans/exo***-114** (c) acquired over the course of the activation. Partial NMR (400 MHz, Acetone-d_6, 298 K) spectra comparison (d) of the pre-compression polymer **9***trans/exo***-114** (i), post-compression polymer before being washed with methanol (ii), reference polymer **S55***exo***-112** (iii), post-compression polymer after being washed with methanol (iv), reference **S3** (v), concentrated methanol washings (vi) and reference compound **2** (vii).

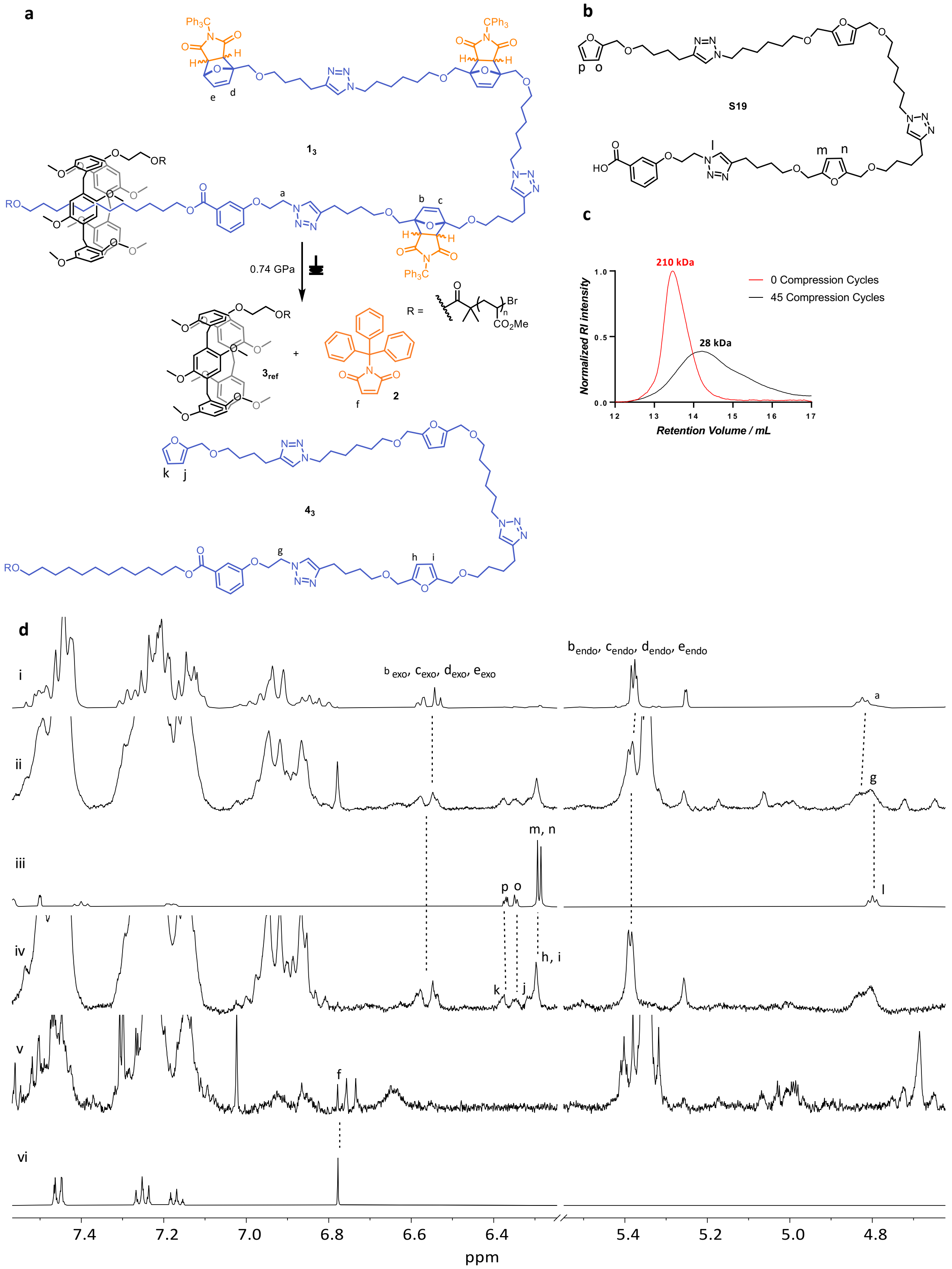

Figure S48. Bulk activation of polymer $\mathbf{1_3a_{210}}$. Bulk activation of polymer $\mathbf{1_3}$ affords polymer fragments $\mathbf{3_{ref}}$ and $\mathbf{4_3}$ along with small molecule **2** (a). Reference compound **S19** (b). SEC traces of polymer $\mathbf{1_3a_{210}}$ (c) with M_n values before (red) and after (black) compression. Partial NMR (400 MHz, Acetone-d_6, 298 K) spectra comparison (d) of the pre-compression polymer $\mathbf{1_3a_{210}}$ (i), post-compression polymer before being washed with methanol (ii), reference compound **S19** (iii), post-compression polymer after being washed with methanol (iv), concentrated methanol washings (v) and reference compound **2** (vi).

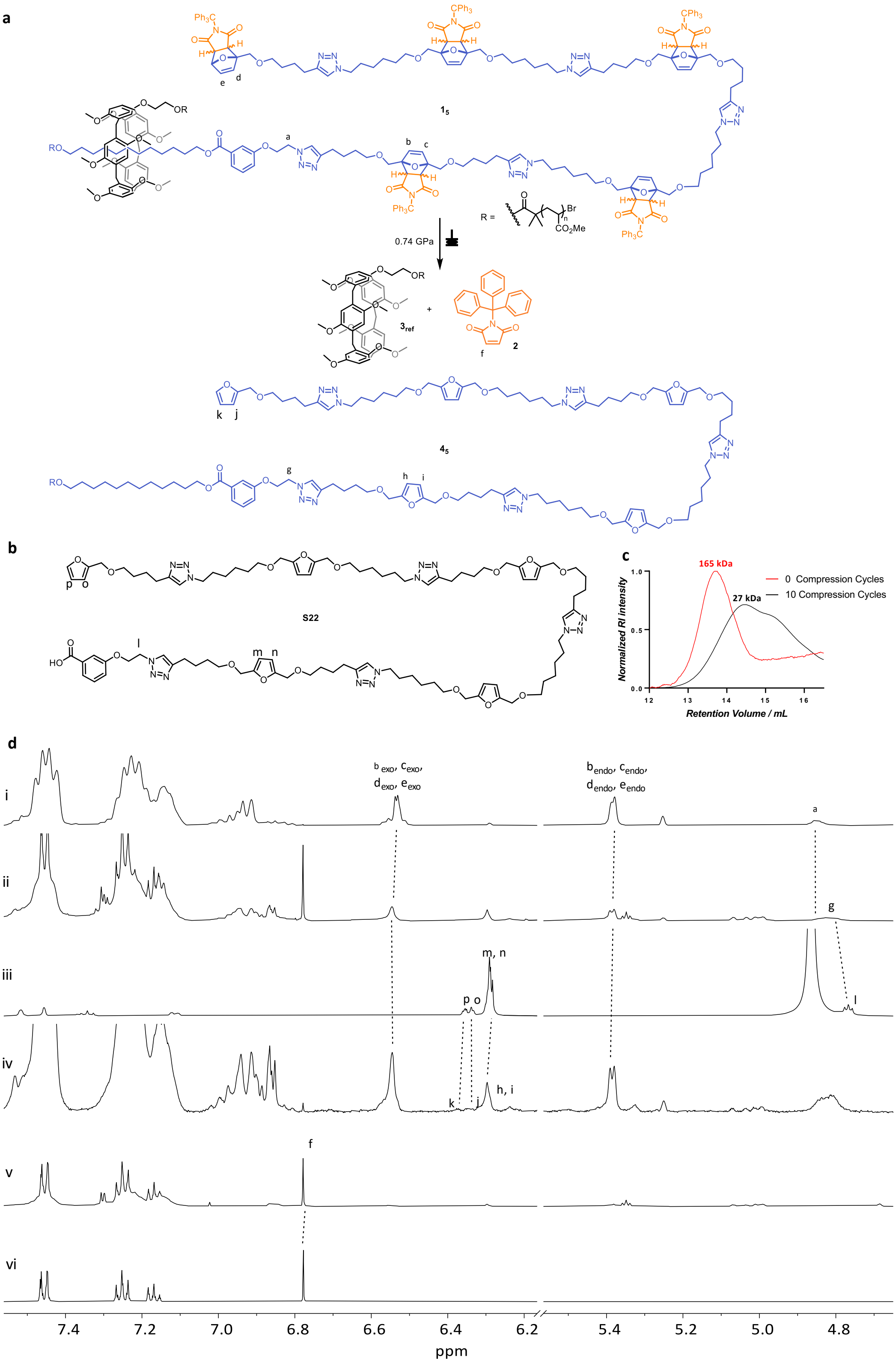

Figure S49. Bulk activation of polymer **$1_{5\text{-}165}$**. Bulk activation of polymer **1_5** affords polymer fragments **3_{ref}** and **4_5** along with small molecule **2** (a). Reference compound **S22** (b). SEC traces of polymer **$1_{5\text{-}165}$** (c) with M_n values before (red) and after (black) compression. Partial NMR (400 MHz, Acetone-d_6, 298 K) spectra comparison (d) of the pre-compression polymer **$1_{5\text{-}165}$** (i), post-compression polymer before being washed with methanol (ii), reference compound **S22** (iii), post-solid-state-activation polymer after being washed with methanol (iv), concentrated methanol washings (v) and reference compound **2** (vi).

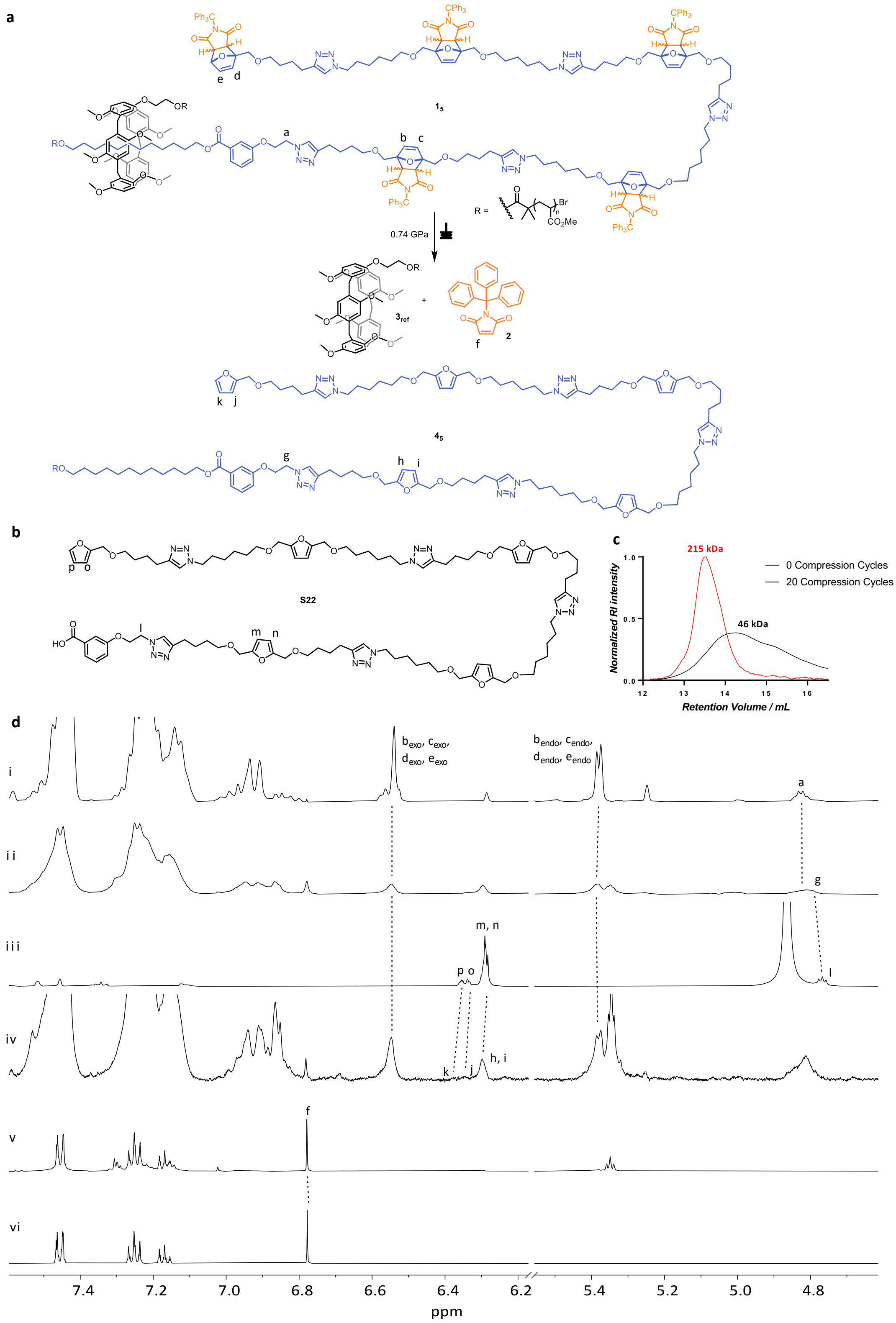

Figure S50. Bulk activation of polymer **$1_{5\text{-}215}$**. Bulk activation of polymer **1_5** affords polymer fragments **3_{ref}** and **4_5** along with small molecule **2** (a). Reference compound **S22** (b). SEC traces of polymer **$1_{5\text{-}215}$** (c) with M_n values before (red) and after (black) compression. Partial NMR (400 MHz, Acetone-d_6, 298 K) spectra comparison (d) of the pre-compression polymer **$1_{5\text{-}215}$** (i), post-compression polymer before being washed with methanol (ii), reference compound **S22** (iii), post-compression polymer after being washed with methanol (iv), concentrated methanol washings (v) and reference compound **2** (vi).

7.4 Bulk Activation Using PMA Matrix

The bulk activation of polymer **9**$_{trans/exo\text{-}114}$ within a PMA matrix, **S56**$_{\text{-}153}$, was investigated. For this purpose, polymer **9**$_{trans/exo\text{-}114}$ (6 mg) was dissolved in DCM (1 mL) along with matrix polymer **S56**$_{\text{-}153}$ (18 mg); the solution was allowed to dry under air for 30 min in a Teflon mould (10 x 5 x 5 mm, h x w x d). The resultant polymer blend film, **S63**, was further dried under vacuum for 24 h before use in a standard compression experiment (see *Section 7.1*). Analysis of the material post-activation was carried out in an identical manner to that of the bulk activation of non-matrix-integrated 1-cargo systems, as described in *Section 6.3*, with the resultant extent of activation of the Diels-Alder adduct and subsequent cargo-release being 6%.

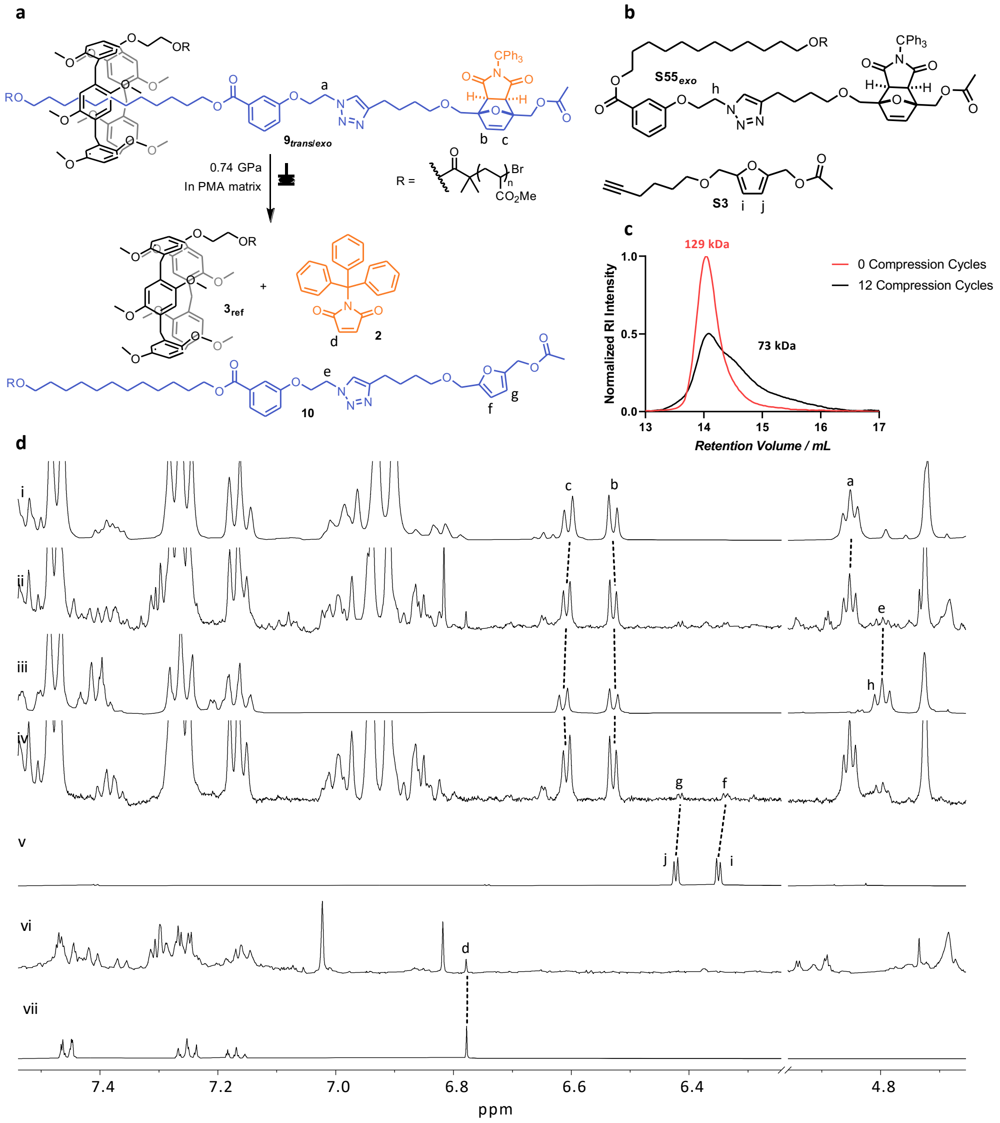

Figure S51. Bulk activation of polymer blend **S63**. Bulk activation of polymer **9**$_{trans/exo}$ affords polymer fragments **3**$_{ref}$ and **10** along with small molecule **2** (a). Reference species **S55**$_{exo\text{-}112}$ and **S3** (b). SEC traces of polymer blend **S63** (c) acquired over the course of the activation. Partial NMR (400 MHz, Acetone-d_6, 298 K) spectra comparison (d) of the pre-compression polymer **9**$_{trans/exo\text{-}114}$ (i), post-compression polymer blend **S63** before being washed with methanol (ii), reference polymer **S55**$_{exo\text{-}112}$ (iii), post-compression polymer blend **S63** after being washed with methanol (iv), reference **S3** (v), concentrated methanol washings (vi) and reference compound **2** (vii).

8 Calculation of Extent of Mechanophore Activation

Here we explain the methods used to calculate the extent of mechanophore activation after either sonication or compression.

8.1 Co-conformational Isomerism in Cargo-loaded Rotaxanes

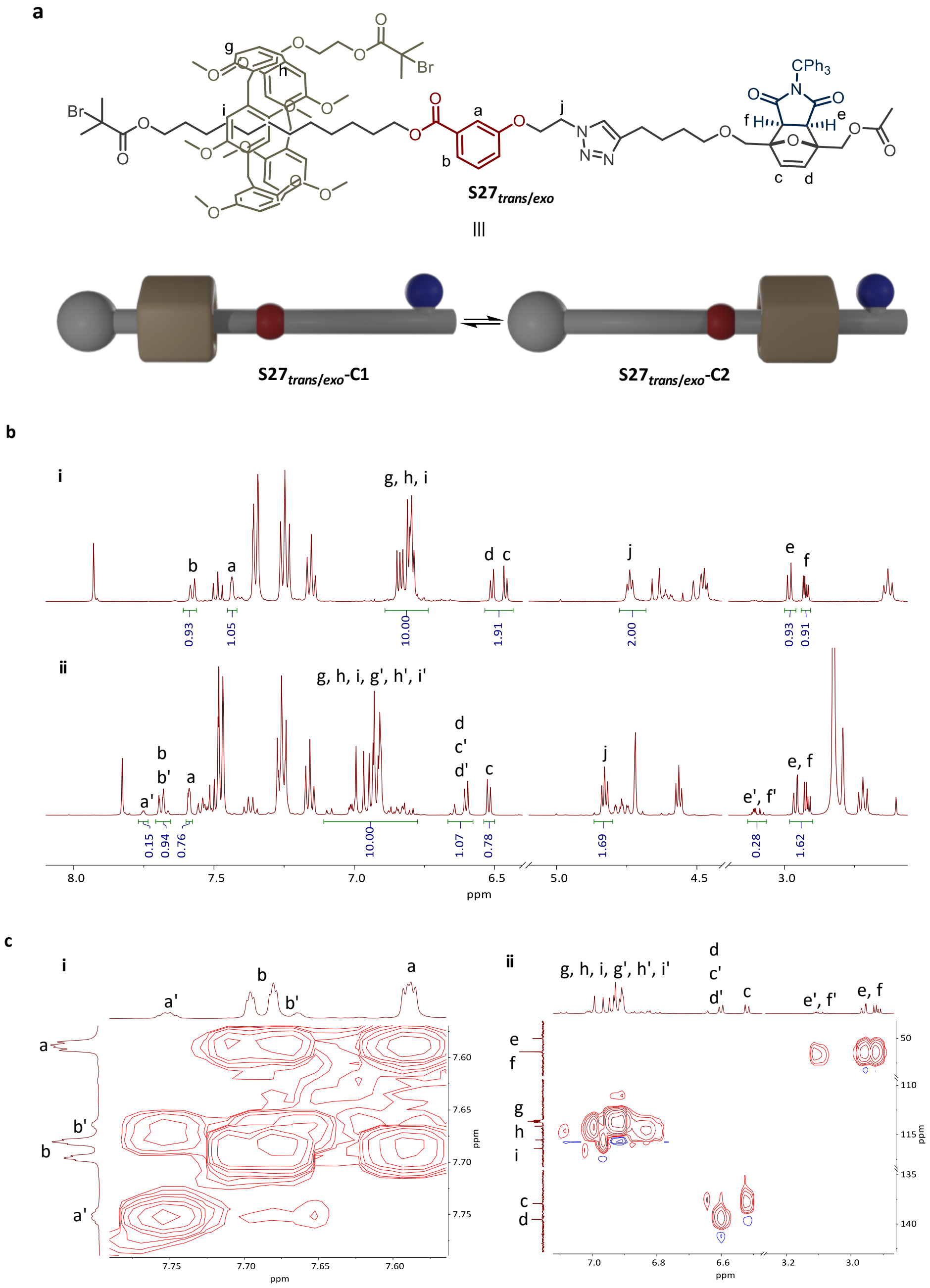

Figure S52. **S27$_{trans/exo}$** is used as an example to demonstrate the different co-conformations that the macrocycle can adopt in all the cargo-loaded rotaxanes reported here (a); different colours on various sections of the structure are used corresponding to those same moieties represented by the cartoon. Partial ^{1}H NMR (500 MHz, 298 K) spectra of rotaxane **S27$_{trans/exo}$** (b) in DMSO-d_6 (i), and acetone-d_6 (ii); signals for those structures where the macrocycle sits on positions represented in cartoons **S27$_{trans/exo}$-C1** and **S27$_{trans/exo}$-C2** are marked as x and x' respectively. c) Partial 2D COSY ^{1}H NMR (i) and 2D HSQC ^{1}H-^{13}C NMR (ii) spectra (500 MHz, Acetone-d_6, 298 K) of rotaxane **S27$_{trans/exo}$**.

The pillar[5]arene macrocycle can resides over two distinct regions of the axle: the C12 alkyl chain connected to the isobutyrate unit and the C4 alkyl chain linking the 1,4-triazole ring and the (first) DA adduct. To investigate this shuttling phenomenon, we used rotaxane **S27*trans/exo*** as an example. As expected, changing the solvent environment of the compound had an effect on the shuttling. The ^{1}H NMR spectrum of **S27*trans/exo*** (*Figure S52b-i*) in DMSO-d_6 is dominated by a single co-conformer (**S27*trans/exo*-C1**), where the macrocycle resides over the C12 chain.

When dissolved in acetone-d_6, the ^{1}H NMR spectrum of the same compound (*Figure S52b-i*) now displays two noticeable and distinguishable sets of signals indicating the presence of a second co-conformation. It is likely that the dominant state is still **S27*trans/exo*-C1** but the set of minor signals corresponds to **S27*trans/exo*-C2**. This observation is supported by diagnostic peaks related the macrocycle (H_g, H_h, and H_i), the central benzene ring on the axle (H_a and H_b), and the cargo unit's olefinic (H_c and H_d) and bridging (H_e and H_f) atoms. Both sets of signals for H_a and H_b were confirmed by ^{1}H COSY NMR (*Figure S52c-i*). Atoms $H_{c\text{-}i}$ are bonded to tertiary carbon atoms and so both sets of signals were in this case confirmed by ^{1}H-^{13}C HSQC NMR (*Figure S52c-ii*); it should be noted that those carbon signals belonging to the structure **S27*trans/exo*-C2** were too weak to be observable but the coupled signals in the ^{1}H NMR spectrum showed a shift in their correlation relative to the signals for the structure **S27*trans/exo*-C2**).

Integration of these two sets of signals (for atoms H_a, H_c, H_d, H_e, and H_f) showed that, in acetone-d_6, 84 ± 1% of the species exist in the **S27*trans/exo*-C1** state with the remainder being **S27*trans/exo*-C2**. As these important diagnostic signals can often be hidden in the post-sonication spectra, we used the integration of signal corresponding to linker atoms H_j as a proxy. In the specific example shown in *Figure S52*, appropriate integration of H_j determines a value of 85% for **S27*trans/exo*-C1** which is almost identical to the value obtained using the aforementioned more typically yet oft-hidden diagnostic signals.

8.2 Calculations for 1-Cargo Mechanophores with No Unstoppering

Polymers **9**$_{trans/exo\text{-}109}$, **9**$_{trans/exo\text{-}114}$, **9**$_{trans/endo\text{-}90}$, and **9**$_{cis/endo\text{-}92}$ all display no noticeable extent of unstoppering during mechanical activation. The method of calculating their extent of activation is described below using the sonication of **9**$_{trans/exo}$**-1** as an example.

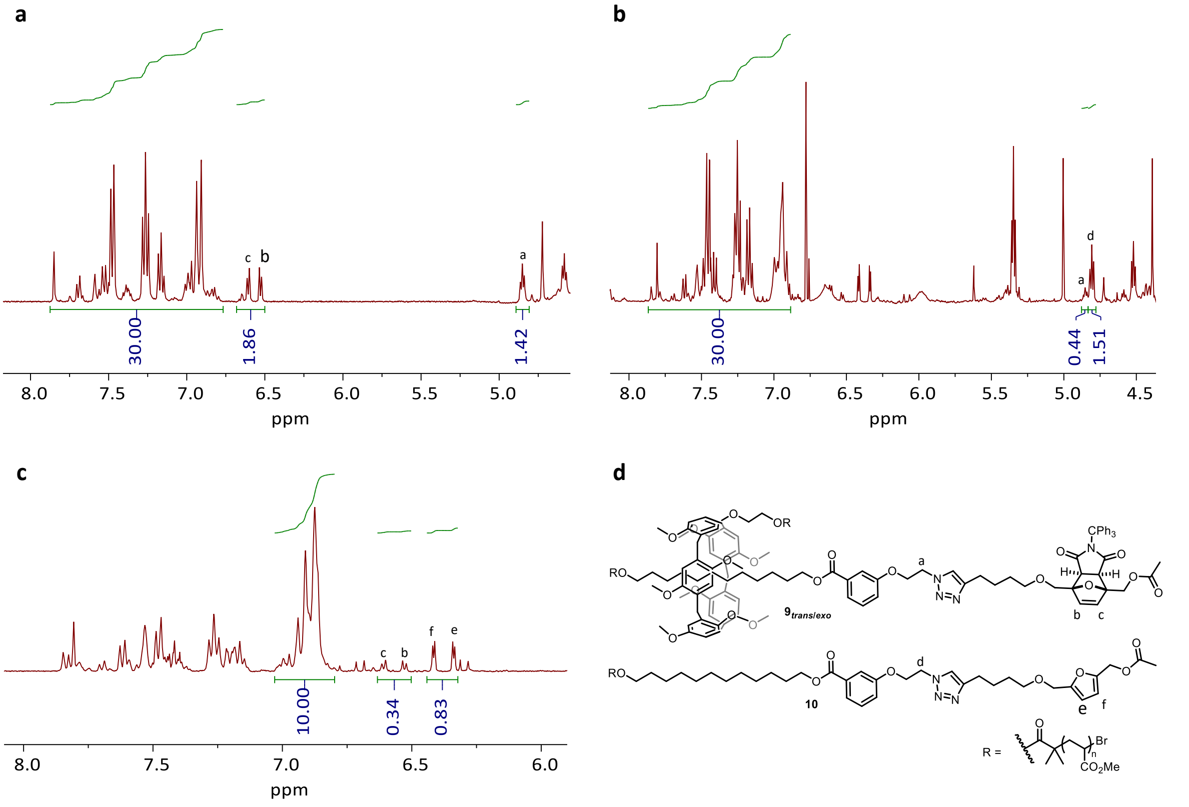

Figure S53. Partial ^{1}H NMR (400 MHz, Acetone-d_6, 298 K) spectra of polymer **9**$_{trans/exo\text{-}109}$ (a), post-sonication polymer **9**$_{trans/exo\text{-}109}$ before being washed with methanol (b) and post-sonication polymer **9**$_{trans/exo\text{-}109}$ after being washed with methanol (c). Structure of polymers **9**$_{trans/exo}$ and **10** (d).

The positional integrity (*P*) caused by shuttling of the macrocycle in the rotaxane can be determined by the ratio of the actual (I_a) and theoretical ($I_{a,t}$) values of the integration of signal H_a in the ^{1}H NMR spectra of polymer **9**$_{trans/exo\text{-}109}$, pre-sonication, as per the formula below:

$$P = \frac{I_a}{I_{a,t}} \times 100\%$$

In this case, ***P* = 71%**; [(1.42 / 2.00) x 100%].

The percentage of intact rotaxane (*R*) was determined from the ^{1}H NMR spectrum of polymer **9**$_{trans/exo}$**-1**, post-sonication and after having been washed with methanol, by the formula below:

$$R = \frac{I_a/P}{I_d + I_a/P} \times 100\%$$

In this case, ***R* = 29%**; [((0.44 / 0.71) / (1.51 + (0.44 / 0.71))) × 100%].

The extent of retro-Diels-Alder reaction (*C*) having occurred during the sonication of polymer $\mathbf{9}_{\textit{trans/exo-109}}$ was finally determined by the formula below:

$$C = \frac{I_{e,f}}{I_{e,f} + I_{b,c}} \times 100\%$$

In this case, ***C* = 71%**; [(0.83 / (0.83 + 0.34)) × 100%].

8.3 Calculations for 1-Cargo Mechanophores with Unstoppering

Polymer **9*cis/exo*-114** is the only 1-cargo system we investigated that displayed a noticeable extent of unstoppering during mechanical activation. The method of calculating the extent of activation for this polymer is described below.

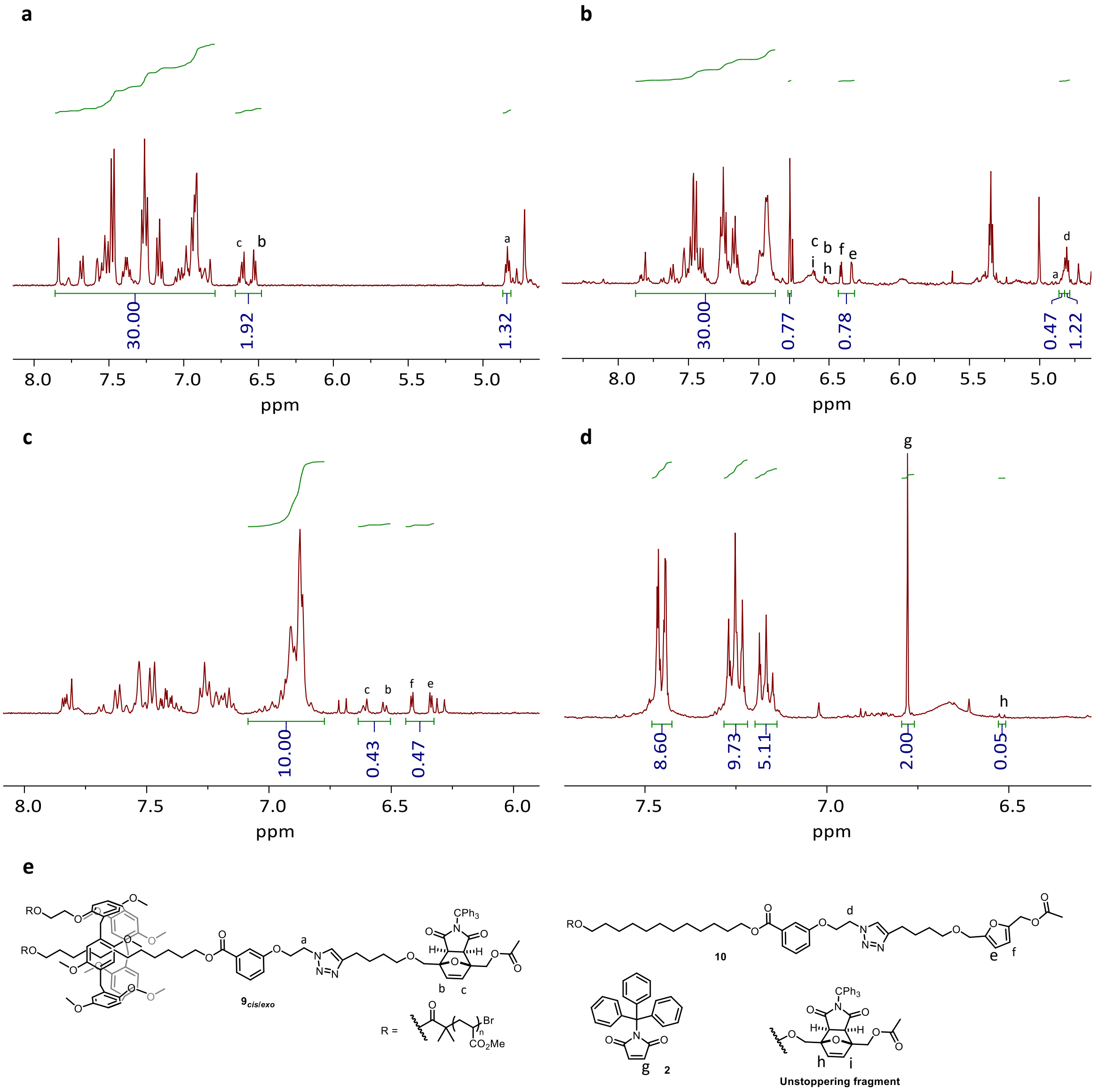

Figure S54. Partial ^{1}H NMR (400 MHz, Acetone-d_6, 298 K) spectra of polymer **9*cis/exo*-114** (a), post-sonication polymer **9*cis/exo*-114** before being washed with methanol (b), post-sonication polymer **9*cis/exo*-114** after being washed with methanol (c), and the concentrated methanol washings (d). Structures of polymer **9*cis/exo***, the axle after successful mechanophore activation **10**, released cargo unit **2**, and a partial representation of the still-loaded cargo fragment that would exist after the process of unstoppering (e).

Here, we use values determined from integration of various specific signals in the ^{1}H NMR spectra of the mechanophore-containing polymer post-sonication, both before and after being washed with methanol, along with the concentrated methanol wash itself. The nomenclature of the designation for each of these values is as follows:

$\boldsymbol{I}^{\mathbf{x}}_{\mathrm{y}}$, where ***I*** represents a total numerical value of integration, **x** represents the ^{1}H NMR spectrum being

used (β = post-sonication polymer before being washed with methanol – *Figure S54b*, γ = post-sonication polymer after being washed with methanol – *Figure S54c*, and δ = the concentrated methanol washings – *Figure S54d*), and **y** represents a list of the signal designations being integrated.

Firstly, we used the method defined previously in *Section 0* to determine the positional integrity, ***P* = 66%;** [(1.32 / 2.00) × 100%], and percentage of intact rotaxane, ***R* = 37%**; [((0.47 / 0.66) / (1.22 + (0.47 / 0.66))) × 100%].

The total integration of signals H_b, H_h, H_c, and H_i ($I^{\beta}_{b,h,c,i}$) in the ^{1}H NMR spectrum of polymer $\mathbf{9}_{cis/exo\text{-}114}$, post-sonication and before being washed with methanol, includes the olefinic signals of the intact Diels-Alder adduct on both the rotaxane polymer and the fragment that results from unstoppering. The integration of signals H_h and H_i ($I^{\beta}_{h,i}$) from the unstoppering-resultant fragment could be determined by formula below:

$$I^{\beta}{}_{h,i} = 2\, I^{\delta}{}_{h} \times \frac{I^{\beta}{}_{g}}{I^{\delta}{}_{g}}$$

In this case, $\boldsymbol{I^{\beta}_{h,i}}$ **= 0.04**; [2.00 × 0.05 × (0.77 / 2.00)].

The percentage of unstoppering (*U*) can be determined by the formula below:

$$U = \frac{I^{\beta}{}_{h,i}}{I^{\beta}{}_{b,h,c,i} + I^{\beta}{}_{e,f}} \times 100\%$$

However, signals H_b, H_h, H_c and H_i were obscured by an undetermined impurity in the required pre-methanol-washed ^{1}H NMR spectrum of post-sonication polymer $\mathbf{9}_{cis/exo\text{-}114}$. Therefore, we determined *U* by an alternative method below:

$$U = \frac{I^{\beta}{}_{h,i}}{I^{\beta}{}_{b,c} + I^{\beta}{}_{h,i} + I^{\beta}{}_{e,f}} \times 100\%$$

The integration of peaks H_b and H_c ($I^{\beta}_{b,c}$) can be determined by the formula below:

$$I^{\beta}{}_{b,c} = I^{\gamma}{}_{b,c} \times \frac{I^{\beta}{}_{e,f}}{I^{\gamma}{}_{e,f}}$$

In this case, $\boldsymbol{I^{\beta}_{b,c}}$ **= 0.71**; [0.43 × (0.78 / 0.47)] and therefore ***U* = 3%**; [(0.04 / (0.71 + 0.04 + 0.78)) × 100%].

The extent of retro-Diels-Alder reaction (*C*) having occurred during the sonication of polymer $\mathbf{9}_{cis/exo\text{-}114}$ was finally determined by the formula below:

$$C = \frac{I^{\beta}{}_{e,f}}{I^{\beta}{}_{b,c} + I^{\beta}{}_{h,i} + I^{\beta}{}_{e,f}} \times 100\%$$

In this case, ***C* = 51%** [(0.78 / (0.71 + 0.04 + 0.78)) × 100%].

8.4 Calculations for 3- and 5-Cargo Mechanophores

Here we use polymer $\mathbf{1_3a_{-210}}$ as an example of how we calculated the extent of mechanophore activation for those polymers that contained multiple cargo units.

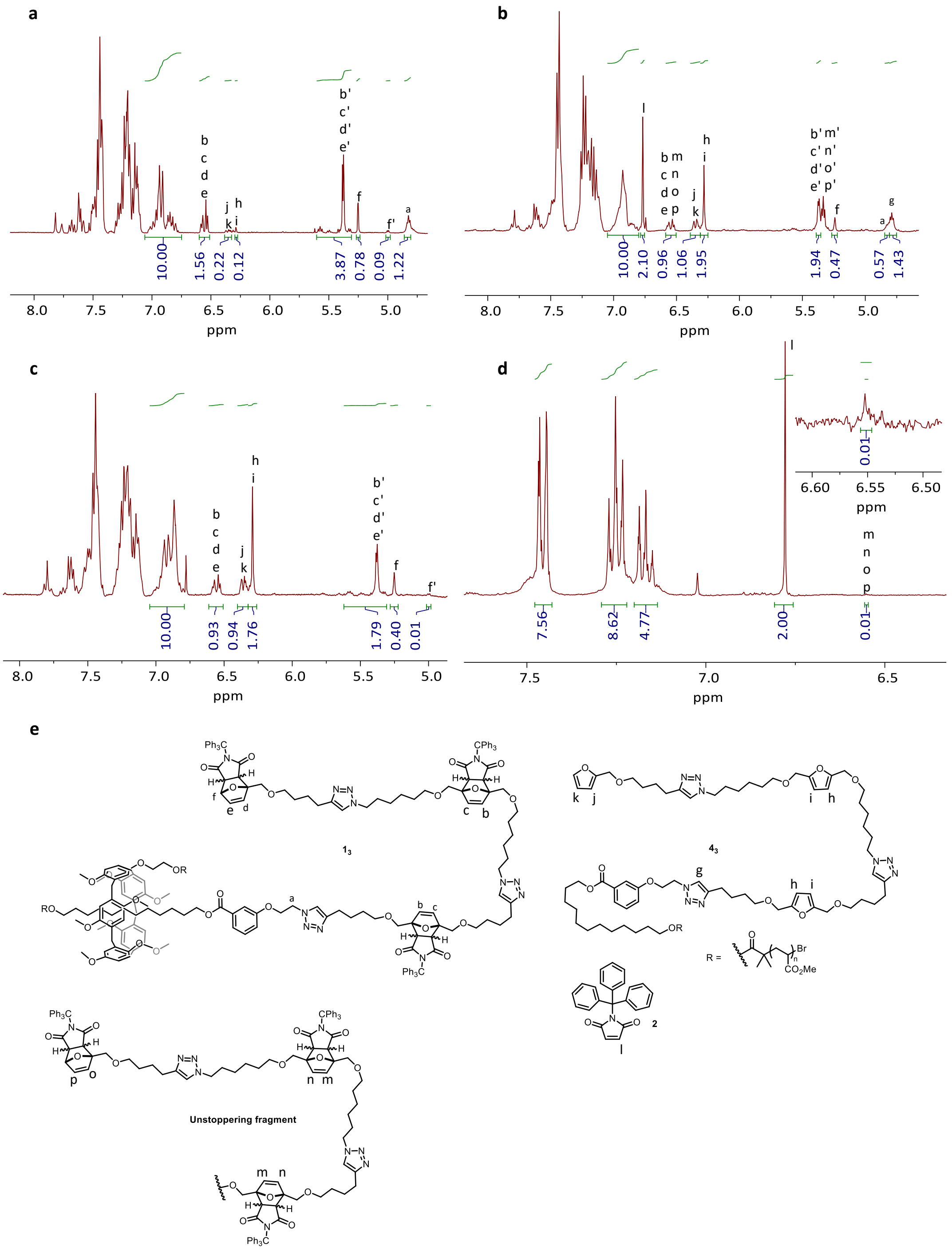

Figure S55. Partial ^{1}H NMR (400 MHz, Acetone-d_6, 298 K) spectra of polymer $\mathbf{1_3a_{-210}}$ (a), post-sonication polymer $\mathbf{1_3a_{-210}}$ before being washed with methanol (b), post-sonication polymer $\mathbf{1_3a_{-210}}$ after being washed with methanol (c), and the concentrated methanol washings (d). Structures of polymer $\mathbf{1_3}$, the axle after successful mechanophore activation $\mathbf{4_3}$, released cargo unit **2**, and a partial representation of the still-loaded cargo fragment that would exist after the process of unstoppering (e). Note: *exo* and *endo* adducts are denoted as x and x' respectively.

As with the 1-cargo system described in *Section 8.3*, we use values determined from integration of various specific signals in the ^{1}H NMR spectra of the mechanophore-containing polymer post-sonication, both before and after being washed with methanol, along with the concentrated methanol wash itself. The nomenclature of the designation for each of these values is as follows:

$\boldsymbol{I^{x}_{y}}$, where ***I*** represents a total numerical value of integration, **x** represents the ^{1}H NMR spectrum being used (β = post-sonication polymer before being washed with methanol – *Figure S55b*, γ = post-sonication polymer after being washed with methanol – *Figure S55c*, and δ = the concentrated methanol washings – *Figure S55d*), and **y** represents a list of the signal designations being integrated.

Firstly, we used the method defined in *Section 8.3* to determine the positional integrity ***P* = 61%**; [(1.22 / 2.00) × 100%], and percentage of intact rotaxane ***R* = 40%**; [((0.57 / 0.61) / (1.43 + (0.57 / 0.61))) × 100%].

The total integration of signals $H_{b\text{-}e}$ and $H_{m\text{-}p}$ ($I^{\beta}_{b\text{-}e,m\text{-}p}$) in the ^{1}H NMR spectrum of polymer **1₃a₋₂₁₀**, post-sonication and before being washed with methanol, includes the olefenic signals of the intact Diels-Alder adduct on both the rotaxane polymer and the fragment that results from unstoppering. The integration of signals $H_{m\text{-}p}$ ($I^{\beta}_{m\text{-}p}$) from the unstoppering-resultant fragment could be determined by the formula below:

$$I^{\beta}{}_{m-p} = I^{\delta}{}_{m-p} \times \frac{I^{\beta}{}_{l}}{I^{\delta}{}_{l}}$$

In this case, $\boldsymbol{I^{\beta}_{m\text{-}p}}$ **= 0.01**; [0.01 × (2.10 / 2.00)].

The percentage of unstoppering (*U*) was determined by the formula below.

$$U = \frac{I^{\beta}{}_{m-p}}{I^{\beta}{}_{b-e,m-p}} \times 100\%$$

In this case, ***U* = 1%**; [(0.01 / 0.96) × 100%].

As the loading of the cargo is not 100% efficient (see *Sections 4.1.23* and *4.1.25*), furan signals can be found in the pre-sonication polymer $\mathbf{1_3a_{-210}}$. Since the furan signals at the internal and terminal positions (see *Section 3* for definitions of internal and terminal cargo positioning) show different shifts, the calculations for these signals can be processed separately.

The percentages of furan species at the internal ($F_{internal}$) and terminal positions ($F_{terminal}$) along with the total value (F_{total}) in the pre-sonication polymer were determined by the formulae below (where *n* = number of cargo units in the polymer – either or 3 or 5):

$$F_{internal} = \frac{I_{h,i}}{(I_{b-e} - 2\,I_{f}) + I_{h,i} + (I_{b\prime-e\prime} - 2\,I_{f\prime})} \times 100\%$$

$$F_{terminal} = \frac{I_{j,k}}{2\,I_{f} + I_{j,k} + 2\,I_{f\prime}} \times 100\%$$

$$F_{total} = \frac{1}{n}\,[(n-1)\,F_{internal} + F_{terminal}]$$

In this case, $F_{internal}$ **= 3%**; [(0.12 / ((1.56 – (2.00 × 0.78)) + 0.12 + (3.87 – (2.00 × 0.09)))) × 100%], $F_{terminal}$ **= 11%**; [(0.22 / ((2 × 0.78) + 0.22 + (2 × 0.09))) × 100%] and F_{total} **= 6%**; [(((3 – 1) × 3%) + 11%) / 3].

The extent of retro-Diels-Alder reaction ($C_{internal}$, $C_{terminal}$, and C_{total}) having occurred during the sonication of polymer **1₃a₋₂₁₀** was finally determined by the formulae below:

$$C_{internal} = \left(\frac{I^{\Upsilon}{}_{h,i} \times (100\% - U)}{(I^{\Upsilon}{}_{b-e} - 2\, I^{\Upsilon}{}_{f}) + I^{\Upsilon}{}_{h,i} + (I^{\Upsilon}{}_{b'-e'} - 2\, I^{\Upsilon}{}_{f'})} \times 100\%\right) - F_{internal}$$

$$C_{terminal} = \left(\frac{I^{\Upsilon}{}_{j,k} \times (100\% - U)}{2\, I^{\Upsilon}{}_{f} + I^{\Upsilon}{}_{j,k} + 2\, I^{\Upsilon}{}_{f'}} \times 100\%\right) - F_{terminal}$$

$$C_{total} = \frac{1}{n}\,[(n-1)\, C_{internal} + C_{terminal}]$$

In this case, $C_{internal}$ **= 45%**; [((1.76 × (100% - 1%)) / ((0.93 – (2.00 × 0.40)) + 1.76 + (1.79 – (2.00 × 0.01))) × 100%) - 3%], $C_{terminal}$ **= 42%**; [((0.94 × (100% - 1%)) / ((2.00 × 0.40) + 0.94 + (2.00 × 0.01)) × 100%) - 11%], and C_{total} **= 44%**; [(((3 - 1) × 45%) + 42%) / 3].

8.5 Calculations for N-(1-pyrenyl)maleimide-Cargo Polymer, 13₋₁₁₉

Mechanical activation of polymer **13₋₁₁₉** results in the desired retro-Diels-Alder (rDA) reaction occurring along with the process of dethreading (DT) but without any noticeable extent of unstoppering. The method of calculating the extent of correct mechanophore activation is described below.

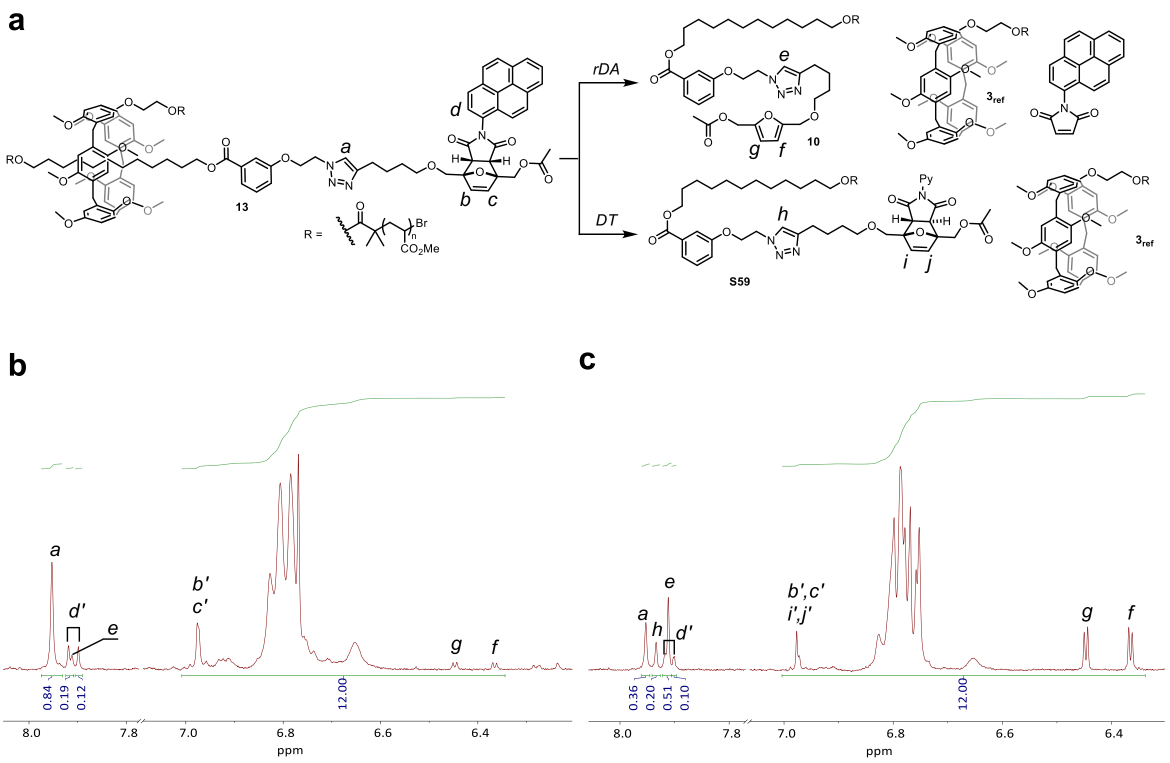

Figure S56. Polymer **13₋₁₁₉** affords polymer fragments **10**, **3ref**, **S59** along with small molecule N-(1-pyrenyl)maleimide after activation (a). Partial NMR (400 MHz, DMSO-d_6, 298 K) spectra of the pre-sonication

polymer **13**$_{-119}$ (b) and post-sonication polymer after being washed with methanol (c).

In the following calculations, we use values determined from integration of specific signals in the ^{1}H NMR spectra of pre- and post-sonication polymer **13**$_{-119}$ after being washed with methanol. The nomenclature of the designation for each of these values is as follows:

I^{x}_{y}, where ***I*** represents a total numerical value of integration, **x** represents the ^{1}H NMR spectrum being used (β = pre-sonication polymer – *Figure S56b*, γ = post-sonication polymer after being washed with methanol – *Figure S56c*), and **y** represents a list of the signal designations being integrated. It should be noted that, in this particular case, the peak requiring integration is $H_{d'}$ which partially overlaps with signal H_e; $H_{d'}$ being a doublet allows us to take the non-overlapped peak of the two as the half-integration of the total peak which we then carry through to our calculations.

Firstly, it was necessary to calculate the extent of activated Diels-Alder structures in the pre-sonication polymer; hence, the percentage of furan species (*F*) in the pre-sonication polymer was determined by the formula below:

$$F = \frac{I^{\beta}{}_{\frac{d'}{2},e} - I^{\beta}{}_{\frac{d'}{2}}}{I^{\beta}{}_{\frac{d'}{2},e} - I^{\beta}{}_{\frac{d'}{2}} + I^{\beta}{}_{a}} \times 100\%$$

In this case, ***F* = 8%**; [(0.19 - 0.12) / (0.19 - 0.12 + 0.84) × 100%].

The percentage of desired retro-Diels-Alder reaction (*C*), intact rotaxane (*R*), and undesired dethreading (*D*) during the sonication of polymer **13**$_{-119}$ were determined by the formulae below:

$$C = \frac{I^{\gamma}{}_{\frac{d'}{2},e} - I^{\gamma}{}_{\frac{d'}{2}}}{I^{\gamma}{}_{\frac{d'}{2},e} - I^{\gamma}{}_{\frac{d'}{2}} + I^{\gamma}{}_{a} + I^{\gamma}{}_{b}} \times 100\% - F$$

$$R = \frac{I^{\gamma}{}_{a}}{I^{\gamma}{}_{\frac{d'}{2},e} - I^{\gamma}{}_{\frac{d'}{2}} + I^{\gamma}{}_{a} + I^{\gamma}{}_{b}} \times 100\%$$

$$D = 100\% - \mathrm{C} - \mathrm{F} - \mathrm{R}$$

In this case, ***C* = 34%**; [(0.51 - 0.10) / (0.51 - 0.10 + 0.36 + 0.20) × 100% - 8%], ***R* = 37%**; [0.36 / (0.51 - 0.10 + 0.36 + 0.20) × 100%], ***D* = 21%**; [100% - 34% - 8% - 37%].

8.6 Calculations for Trityl-Cargo Polymer, 14$_{-124}$

The method of calculating the extent of correct mechanophore activation for polymer **14**$_{-124}$ is described below.

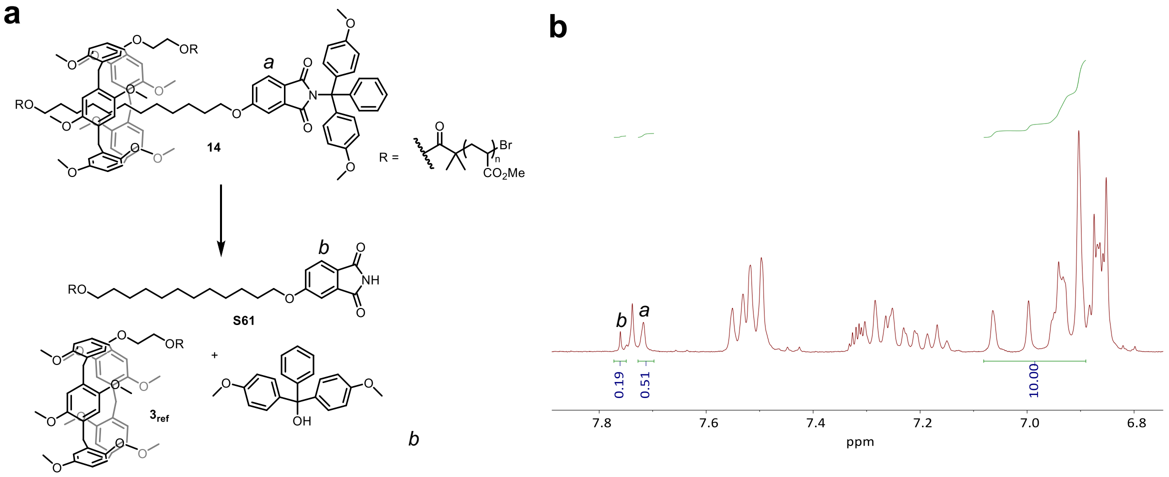

Figure S57. Polymer **14$_{\text{-}124}$** affords polymer fragment **S61** and other fragments after activation (a). Partial NMR (400 MHz, Acetone-d_6, 298 K) spectrum of post-sonication polymer **14$_{\text{-}124}$** after being washed with methanol (b).

In the calculation, we use values determined from the integration of half of the symmetrical peaks H_a ($I_{a/2}$) and H_b ($I_{b/2}$) due to one half of each of their respective signals overlapping in the ^{1}H NMR spectrum of post-sonication polymer **14$_{\text{-}124}$** after being washed with methanol. The nomenclature of the designation for each of these values is as follows:

$\boldsymbol{I}_{\mathbf{y}}$, where $\boldsymbol{I}$ represents a total numerical value of integration, and **y** represents a list of the signal designations being integrated.

The extent of cleavage of the desired C-N bond (C_{CN}) having occurred during the sonication was determined by the formula below:

$$C_{CN} = \frac{I_{\frac{a}{2}}}{I_{\frac{a}{2}} + I_{\frac{b}{2}}} \times 100\%$$

In this case, $\boldsymbol{C_{CN}}$ **= 27%** [0.19 / (0.19 + 0.51) × 100%].

8.7 Summary of Mechanophores Activated by Sonication

Table S2. Analysis of mechanical activation *via* ultrasound of our reported mechanophores which bear N-(triphenylmethyl)maleimide as their cargo.

No.	Polymer	Rotaxane isomer	DA adduct			Pre-sonication		Post-sonication		Percentage (%)									
										Pre-sonication				Post-sonication					
						M_n (kDa)	$Đ$	M_n (kDa)	$Đ$	Furan content[a]			Positional integrity[b]	Intact rotaxane[c,d]	Unstoppering[c]	Conversion of retro-DA reaction[e]			Error[i]
										Internal positions	Terminal position	Total				Internal DA adducts[f]	Terminal DA adduct[g]	All DA adducts[h]	
**1**	$\mathbf{9}_{trans/exo-109}$	*Trans*	One cargo	Triphenyl methyl N-substituent	*Exo*	109	1.12	30	1.24	0			71	29	0	N/A		71	0
**2**	$\mathbf{9}_{cis/exo-114}$	*Cis*			*Exo*	114	1.12	30	1.28				66	37	3			51	9
**3**	$\mathbf{9}_{trans/endo-90}$	*Trans*			*Endo*	90	1.16	30	1.32				83	29	0			71	0
**4**	$\mathbf{9}_{cis/endo-92}$	*Cis*			*Endo*	92	1.17	29	1.32				68	35	0			62	3
**5**	$\mathbf{S28}_{exo-112}$	Thread			*Exo*	112	1.25	31	1.63				N/A					2	N/A
**6**	$\mathbf{S28}_{endo-65}$	Thread			*Endo*	65	1.20	30	1.38									4	
**7**	$\mathbf{1_3a}_{-210}$	*Trans*	Three cargo		*Endo-Endo-Exo, Endo-Endo-Endo*	210	1.32	48	1.30	3	11	6	61	40	1	45	42	44	15
**8**	$\mathbf{1_3b}_{-142}$	*Trans*			*Exo-Endo-Exo, Endo-Exo-Exo, Exo-Endo-Endo, Endo-Exo-Endo*	142	1.32	46	1.36	3	7	4	71	50	2	30	25	28	20
**9**	$\mathbf{1_3b}_{-171}$	*Trans*				171	1.27	47	1.36	5	9	6	70	44	2	36	42	38	17
**10**	$\mathbf{1_3b}_{-178}$	*Trans*				178	1.33	54	1.33	5	8	6	64	50	1	32	26	30	19
**11**	$\mathbf{1_3c}_{-174}$	*Trans*			*Exo-Exo-Exo, Exo-Exo-Endo*	174	1.25	51	1.26	1	3	2	74	52	4	31	24	29	15
**12**	$\mathbf{1_3d}_{-89}$	*Trans*			All isomers	89	1.15	38	1.35	6	11	8	73	46	2	33	27	31	21
**13**	$\mathbf{1}_{5-60}$	*Trans*	Five cargo		All isomers	60	1.20	28	1.27	0	0	0	61	64	Trace[j]	16	20	17	19
**14**	$\mathbf{1}_{5-165}$	*Trans*				165	1.37	75	1.40	3	7	4	64	68	Trace[j]	15	18	16	16
**15**	$\mathbf{1}_{5-215}$	*Trans*				215	1.23	46	1.33	5	5	5	67	51	5	22	23	22	22

Notes:
a) Percentage of non-Diels-Alder-adducted furan species in the pre-sonication mechanophore. b) Percentage of macrocycle located over the alkyl chain pre-sonication (co-conformer C1, *see Section 8.1*). c) As a percentage of all rotaxane species in the post-sonication material. d) Data from post-sonication polymer before it was washed with methanol. e) Data from post-sonication polymer after it was washed with methanol. f) As a percentage of internal DA adducts only. g) As a percentage of terminal DA adducts only. h) As a percentage of all DA adducts. i) This value is given as the remaining percentage that is unaccounted for after subtracting the intact rotaxane along with the rotaxanes that go through the process of unstoppering and those that undergo the desired cargo activation. The large error comes from the accumulation of the uncertainty on the integration of ^1H NMR spectra. j) There is a very small peak required for determining this value overlapping with a significantly larger peak meaning accurate integration could not be carried out.

8.8 Summary of Alternative-Cargo Mechanophores Activated by Sonication

Table S3. Analysis of mechanical activation *via* ultrasound of our reported mechanophores with alternative cargo molecules.

No.	Mechanophore	Rotaxane isomer	Cargo	Pre-sonication		Post-sonication		Percentage (%)			
								Pre-sonication	Post-sonication		
				M_n (kDa)	$Đ$	M_n (kDa)	$Đ$	Furan content	Intact rotaxane	Dethreading	Conversion of rDA or CN bond cleavage
**1**	**11**$_{-127}$	*Trans*	Doxorubicin attached through peptide linker to functionalised maleimide	127	1.31	46	1.27	0	33[b]	0	69[c]
**2**	**13**$_{-119}$	*Trans*	N-(1-pyrenyl)maleimide	119	1.20	40	1.35	8	37	21	34
**3**	**13**$_{-119}$ [a]	*Trans*				41	1.33		37	20	35
**4**	**S59**$_{-77}$	Thread		77	1.29	32	1.28	5	NA		1
**5**	**14**$_{-124}$	*Trans*	Trityl cation derivative	124	1.19	37	1.38	NA	73	0	27
**6**	**S60**$_{-95}$	Thread		95	1.15	35	1.34		NA		0

Notes:
a) This entry corresponds specifically to the sonication of **13**$_{-119}$ in the presence of 1-dodecanethiol (see *Section 6.7.1*). b) Data from the post-sonication polymer after being washed with methanol, as according to the calculation method described in *Section 8.3*. c) Data was also calculated according to the calculation method described in *Section 8.3*; however, as another peak overlaps with the *endo* DA adduct olefin peak, we use the integration of the peak of the *exo* DA adduct olefin peak to calculate the *endo* value according to the ratio of the isomers present in rotaxane **S33** – the error here is 2%.

8.9 Summary of Mechanophores Activated in the Solid-State

Table S4. Analysis of mechanical activation *via* solid-state-compression of all our reported mechanophores.

No.	Polymer	Rotaxane isomer	DA adduct			Pre-compression		Post-compression		Percentage (%)										
										Pre-compression				Post-compression						
										Furan content[a]			Positional integrity[b]	Intact rotaxane[c,d]	Unstoppering[c]	Conversion of retro-DA reaction[e]			Error[i]
						M_n (kDa)	*Đ*	M_n (kDa)	*Đ*	Internal positions	Terminal position	Total				Internal DA adducts[f]	Terminal DA adduct[g]	All DA adducts[h]	
1	$\mathbf{9}_{trans/exo\text{-}114}$	*Trans*	One cargo	Triphenyl methyl N-substituent	*Exo*	114	1.17	20	2.39	0			81	53	0	N.A.		30	17
2	$\mathbf{S28}_{exo\text{-}112}$	Thread			*Exo*	112	1.25	56	2.00				N.A.					0	N.A.
3	$\mathbf{1_3a}_{\text{-}210}$	*Trans*	Three cargo		*Endo-Endo-Exo, Endo-Endo-Endo*	210	1.32	28	4.68	3	11	6	61	58	0	23	14	20	22
4	$\mathbf{1}_{5\text{-}165}$	*Trans*	Five cargo		All isomers	165	1.37	27	3.07	3	6	4	64	52	19	13	6	12	17
5	$\mathbf{1}_{5\text{-}215}$	*Trans*				215	1.23	46	2.19	5	4	5	67	39	2	9	5	8	51

Notes:

a) Percentage of non-Diels-Alder-adducted furan species in the pre-sonication mechanophore. b) Percentage of macrocycle located over the alkyl chain pre-sonication (co-conformer C1, *see Section 8.1*). c) As a percentage of all rotaxane species in the post-sonication material. d) Data from post-sonication polymer before it was washed with methanol. e) Data from post-sonication polymer after it was washed with methanol. f) As a percentage of internal DA adducts only. g) As a percentage of terminal DA adducts only. h) As a percentage of all DA adducts. i) This value is given as the remaining percentage that is unaccounted for after subtracting the intact rotaxane along with the mechanophores that go through the process of unstoppering and those that undergo the desired cargo activation. The large error comes from the accumulation of the uncertainty on the integration of ^{1}H NMR spectra.

9 NMR Spectra

9.1 Small Molecule NMR Spectra

9.1.1 Spectra of 2

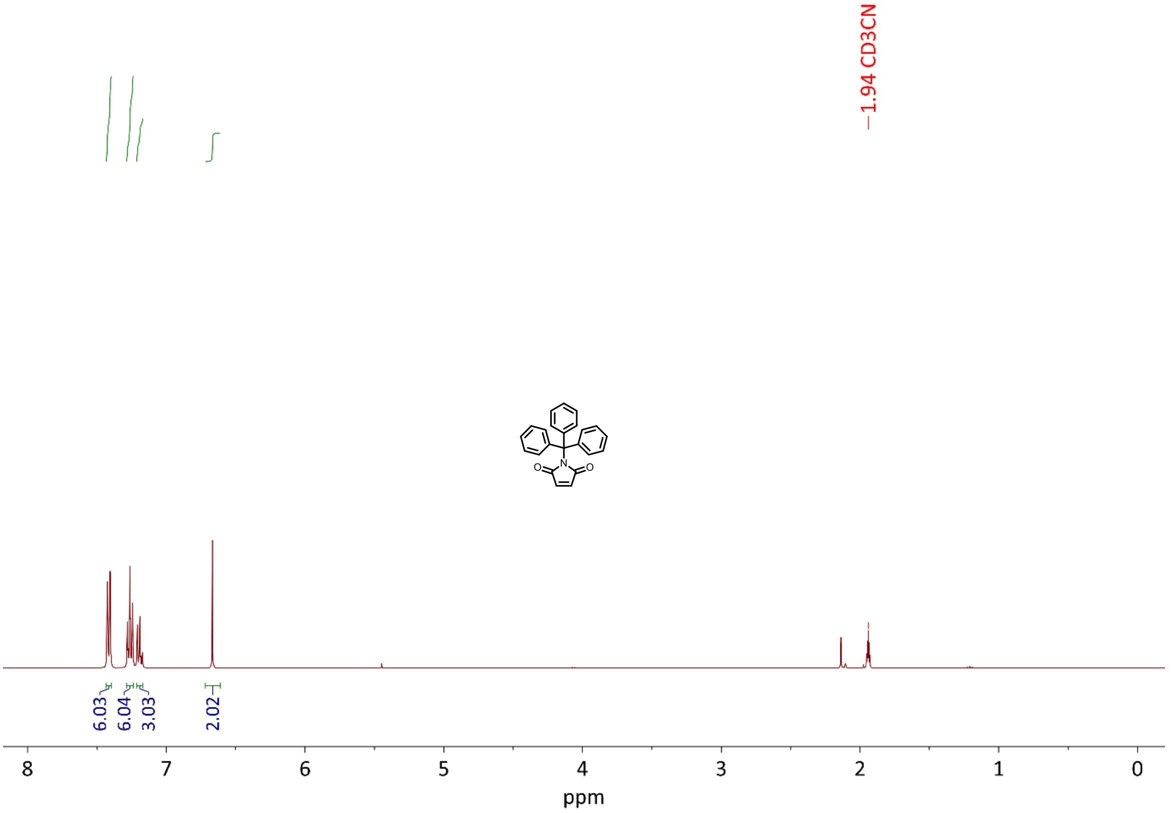

Spectrum S1. ^{1}H NMR (400 MHz, CD_3CN, 298 K) spectrum of compound **2**.

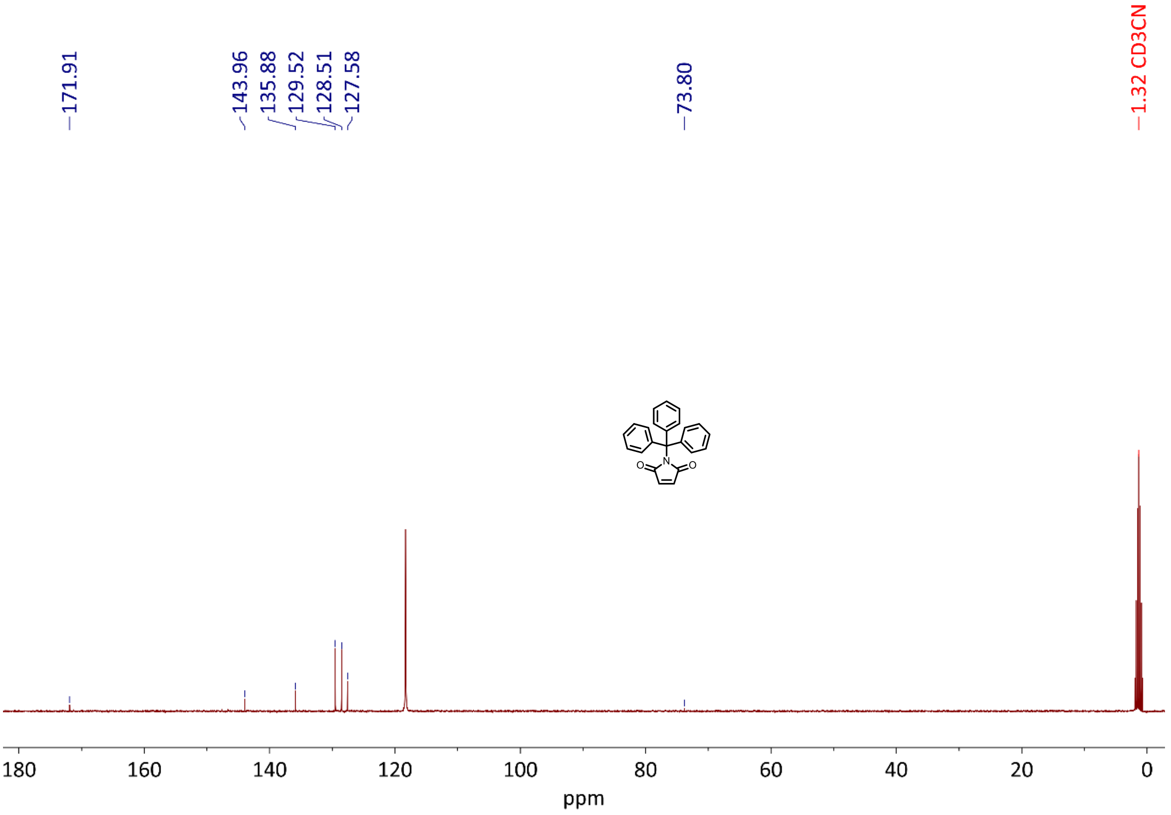

Spectrum S2. ^{13}C NMR (101 MHz, CD_3CN, 298 K) spectrum of compound **2**.

9.1.2 Spectra of S1

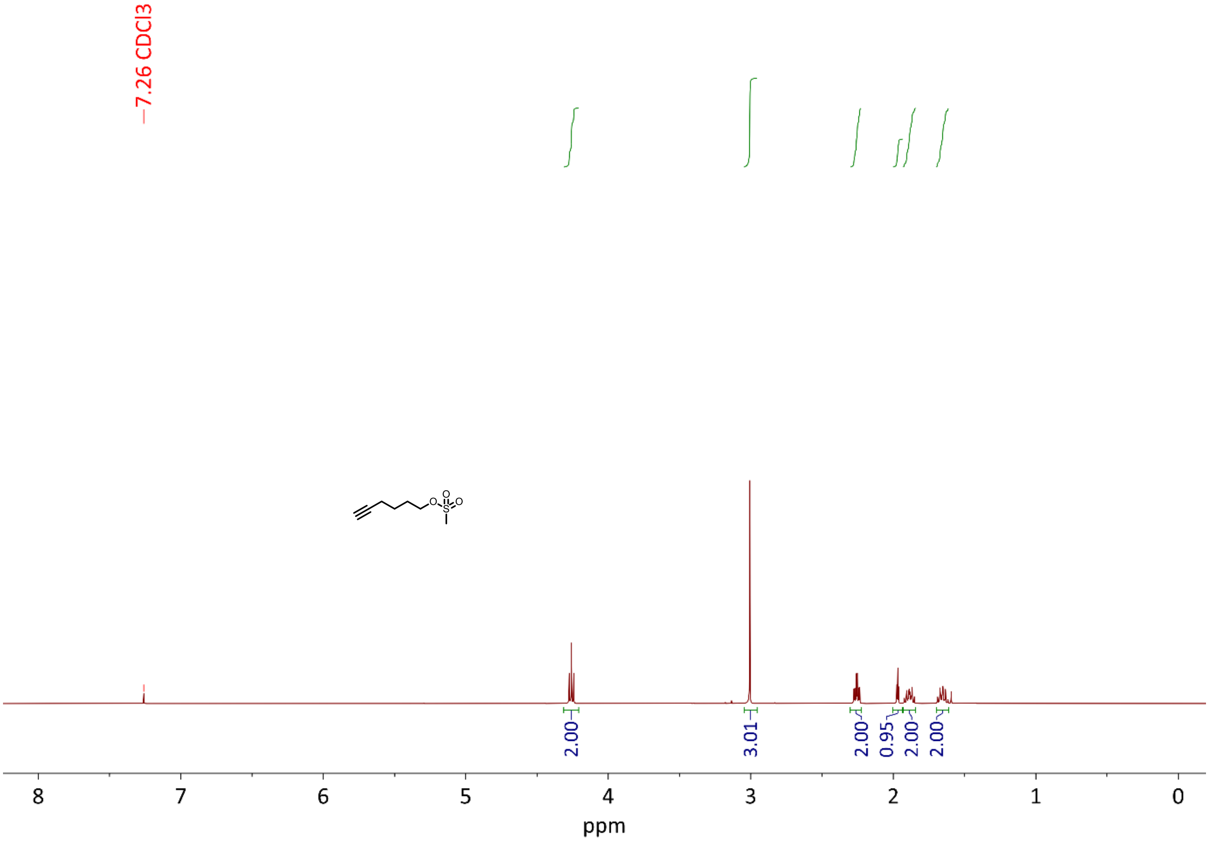

Spectrum S3. ^{1}H NMR (400 MHz, $CDCl_3$, 298 K) spectrum of compound **S1**.

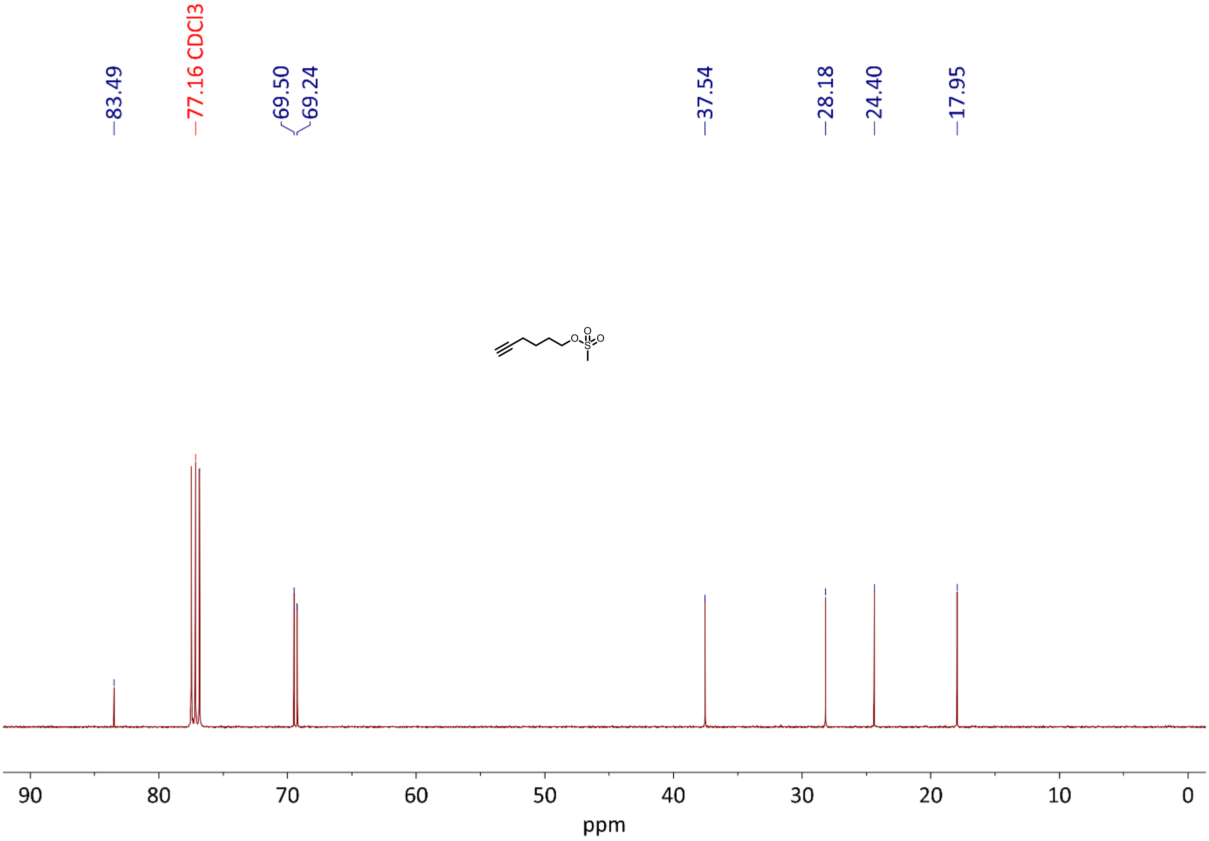

Spectrum S4. ^{13}C NMR (101 MHz, $CDCl_3$, 298 K) spectrum of compound **S1**.

9.1.3 Spectra of S2

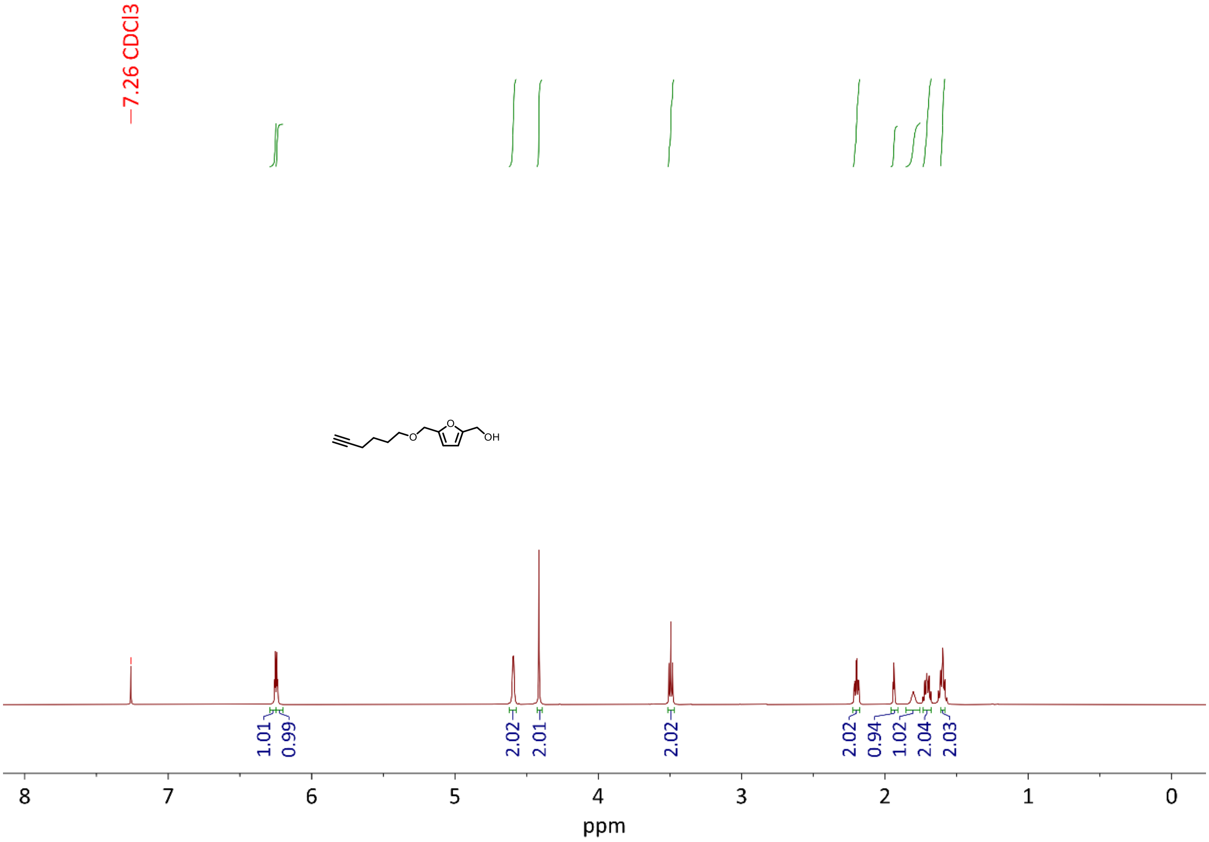

Spectrum S5. ^{1}H NMR (500 MHz, $CDCl_3$, 298 K) spectrum of compound **S2**.

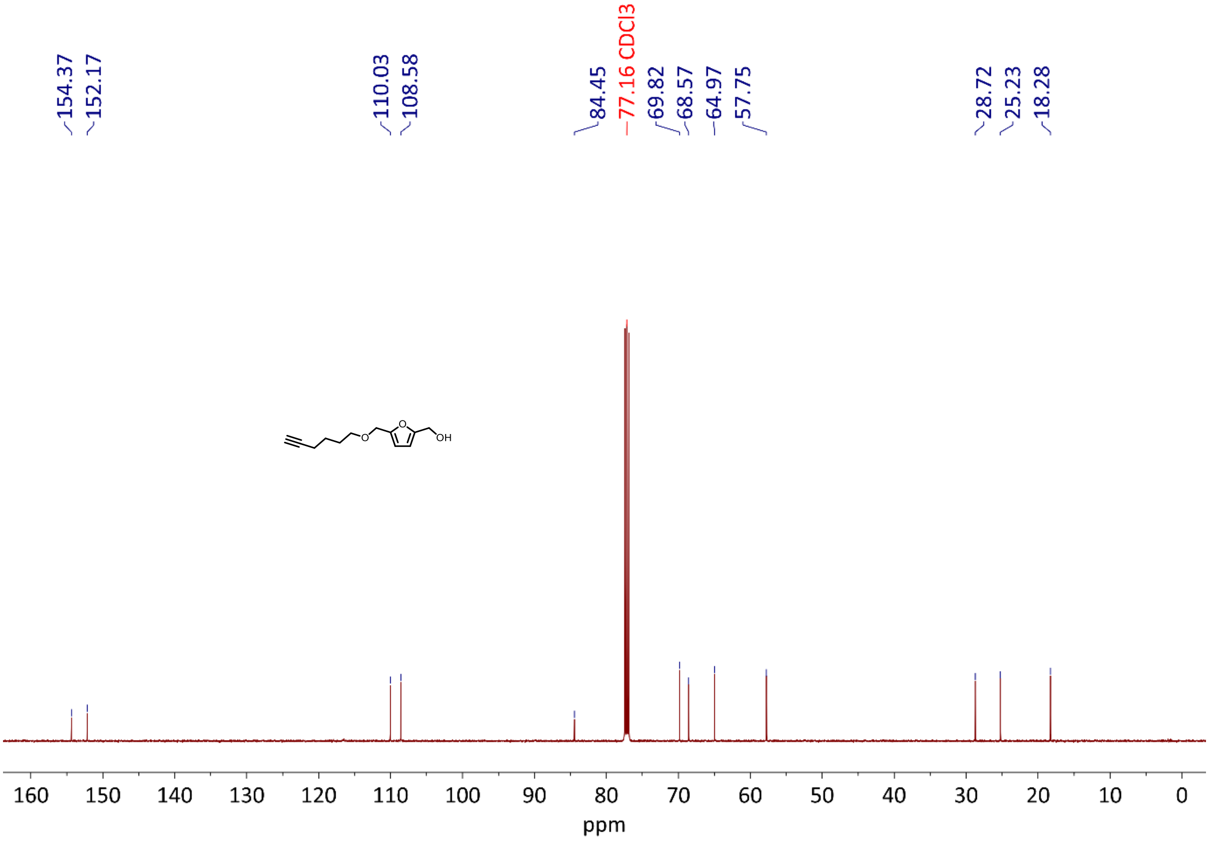

Spectrum S6. ^{13}C NMR (126 MHz, $CDCl_3$, 298 K) spectrum of compound **S2**.

9.1.4 Spectra of S3

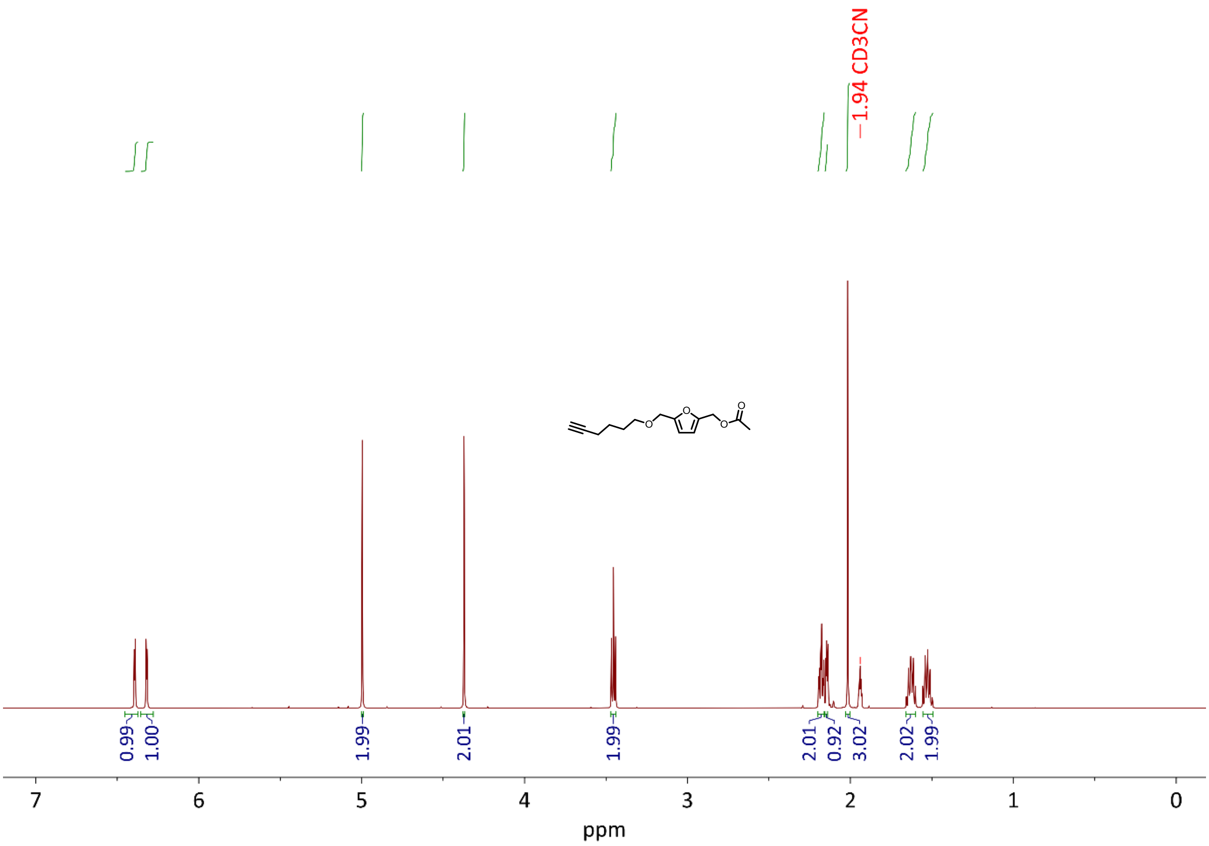

Spectrum S7. ^{1}H NMR (500 MHz, CD_3CN, 298 K) spectrum of compound **S3**.

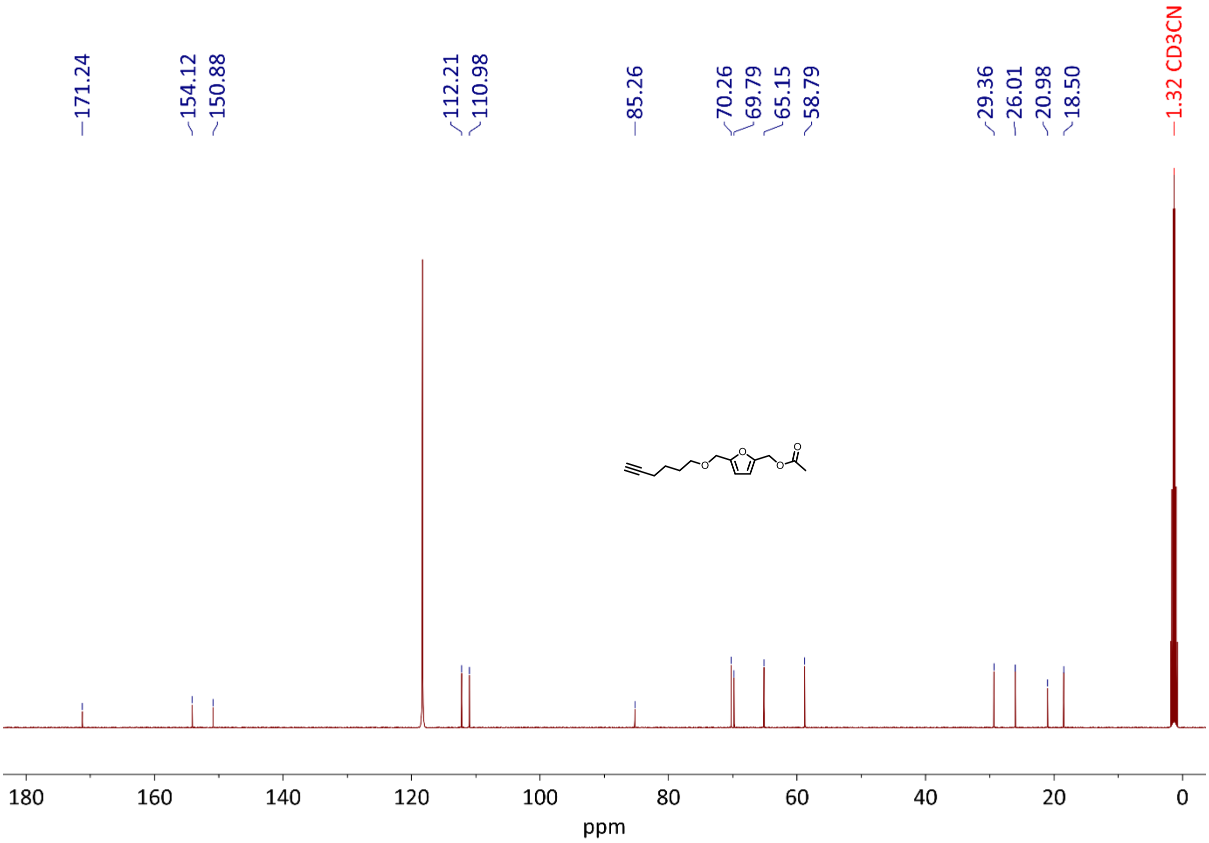

Spectrum S8. ^{13}C NMR (126 MHz, CD_3CN, 298 K) spectrum of compound **S3**.

9.1.5 Spectra of S4$_{exo}$

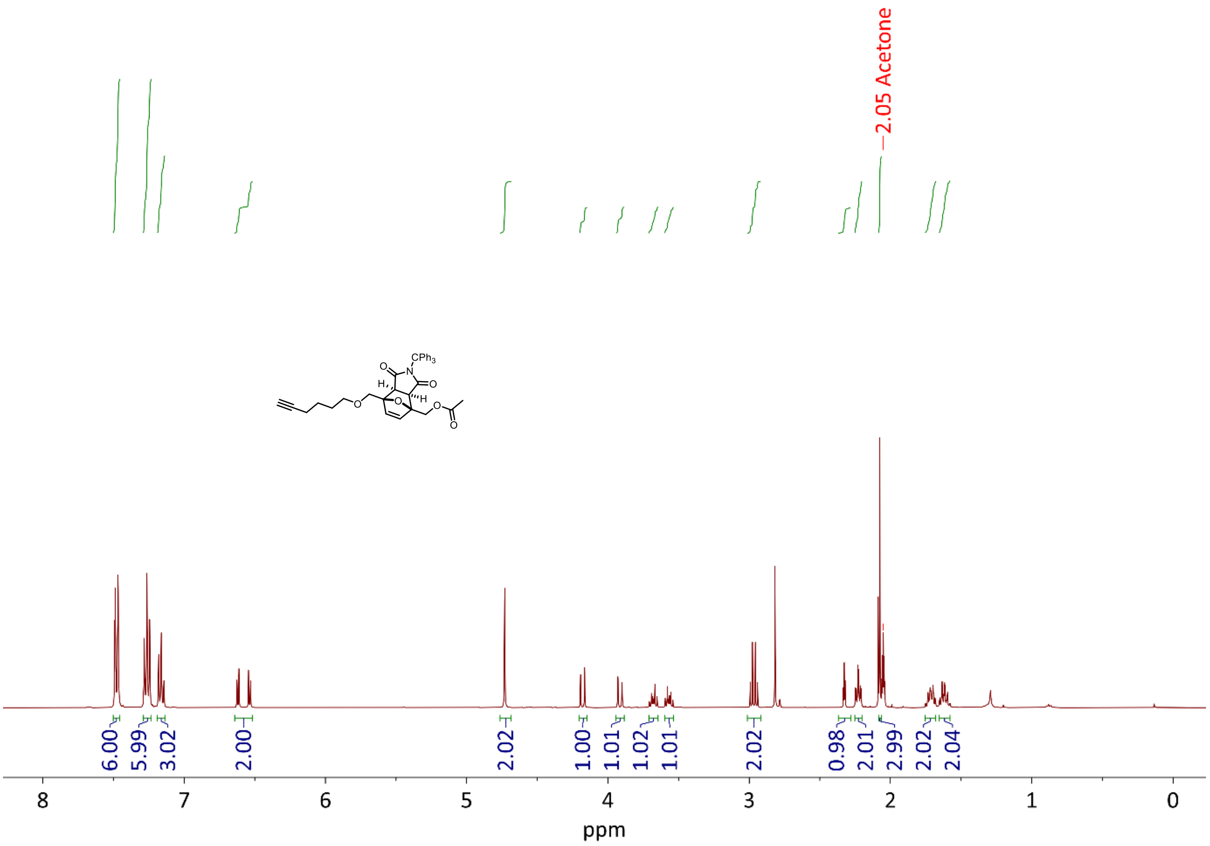

Spectrum S9. ^{1}H NMR (400 MHz, Acetone-d_6, 298 K) spectrum of compound **S4$_{exo}$**.

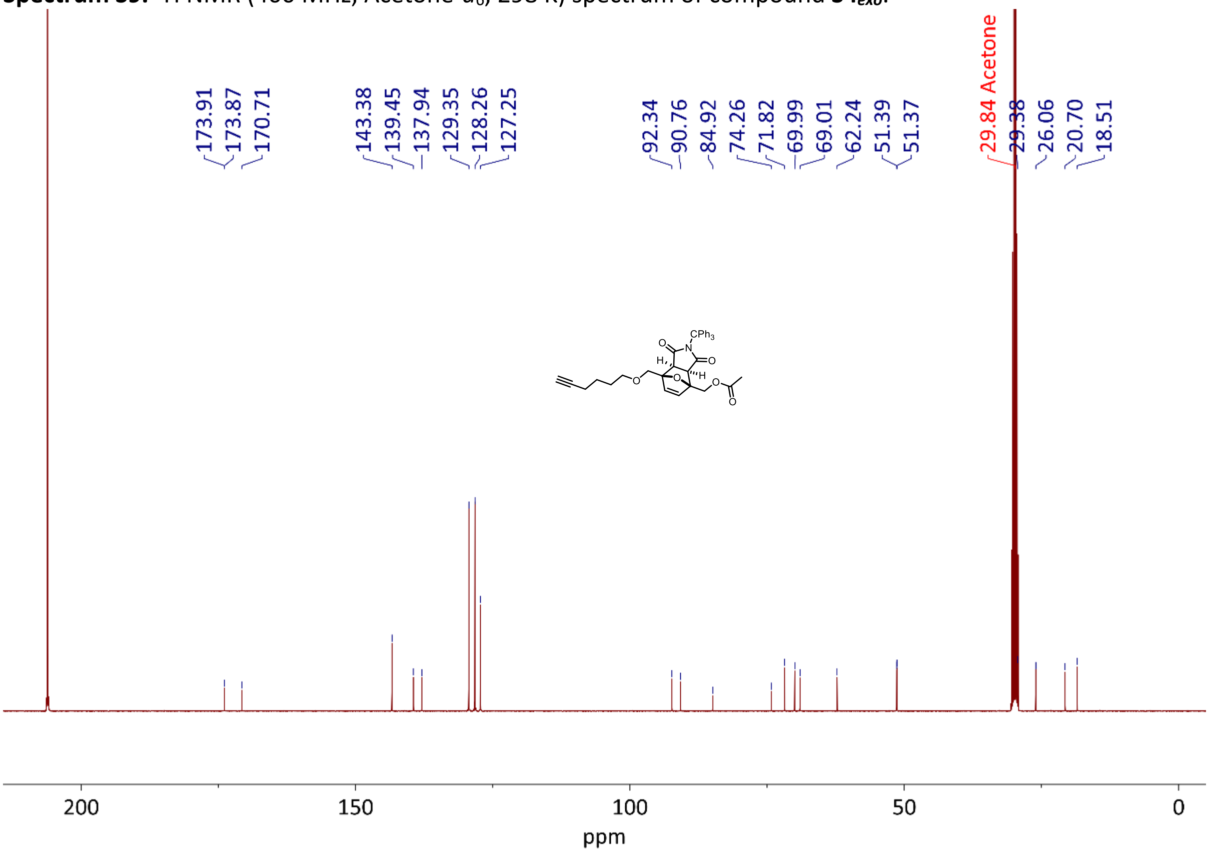

Spectrum S10. ^{13}C NMR (101 MHz, Acetone-d_6, 298 K) spectrum of compound **S4$_{exo}$**.

9.1.6 Spectra of S4$_{endo}$

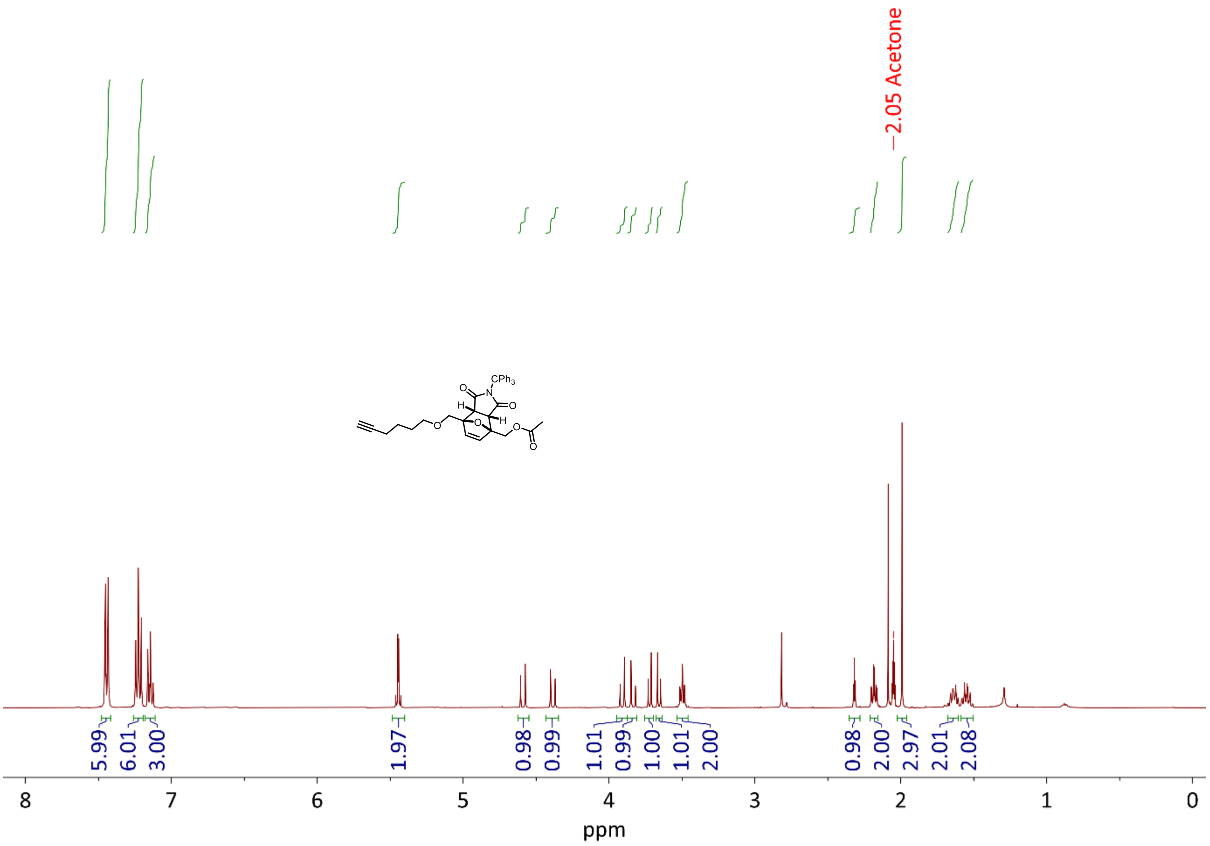

Spectrum S11. ^{1}H NMR (400 MHz, Acetone-d_6, 298 K) spectrum of compound **S4$_{endo}$**.

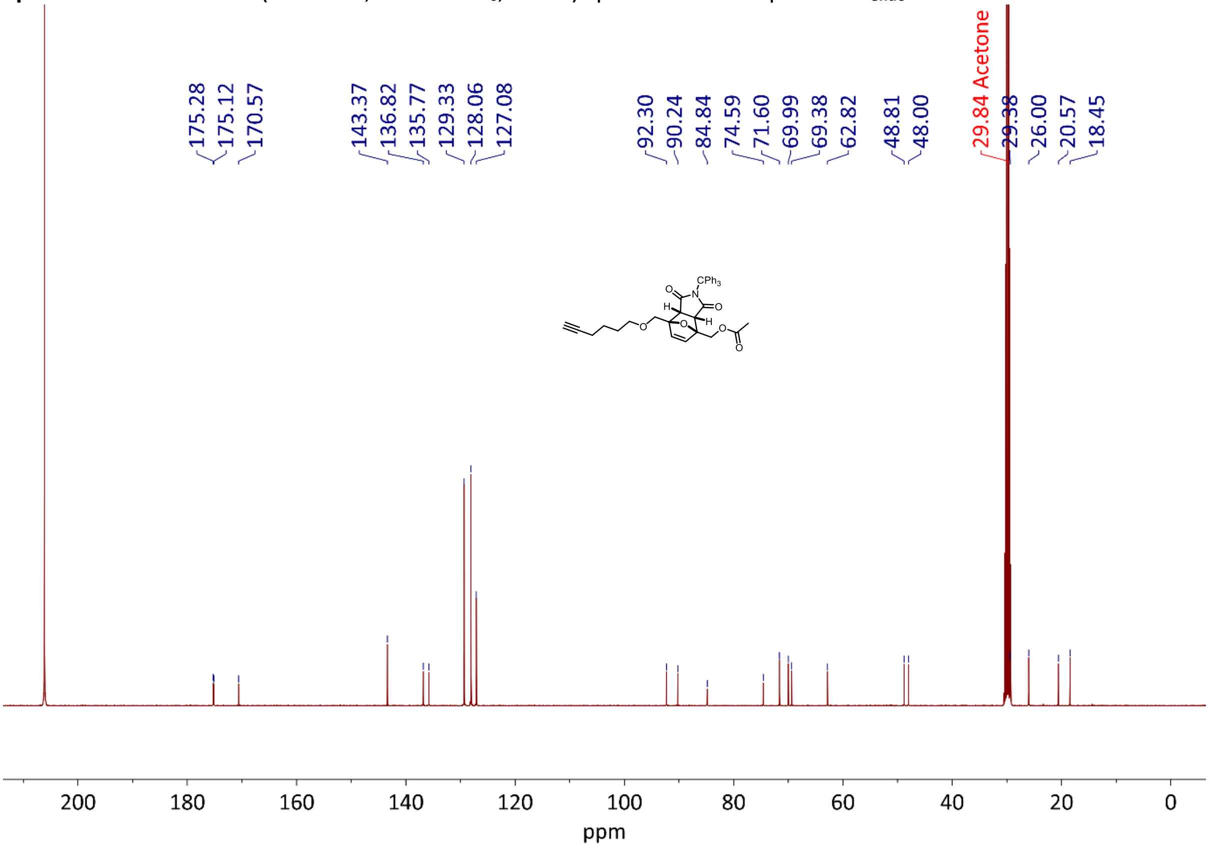

Spectrum S12. ^{13}C NMR (101 MHz, Acetone-d_6, 298 K) spectrum of compound **S4$_{endo}$**.

9.1.7 Spectra of S5

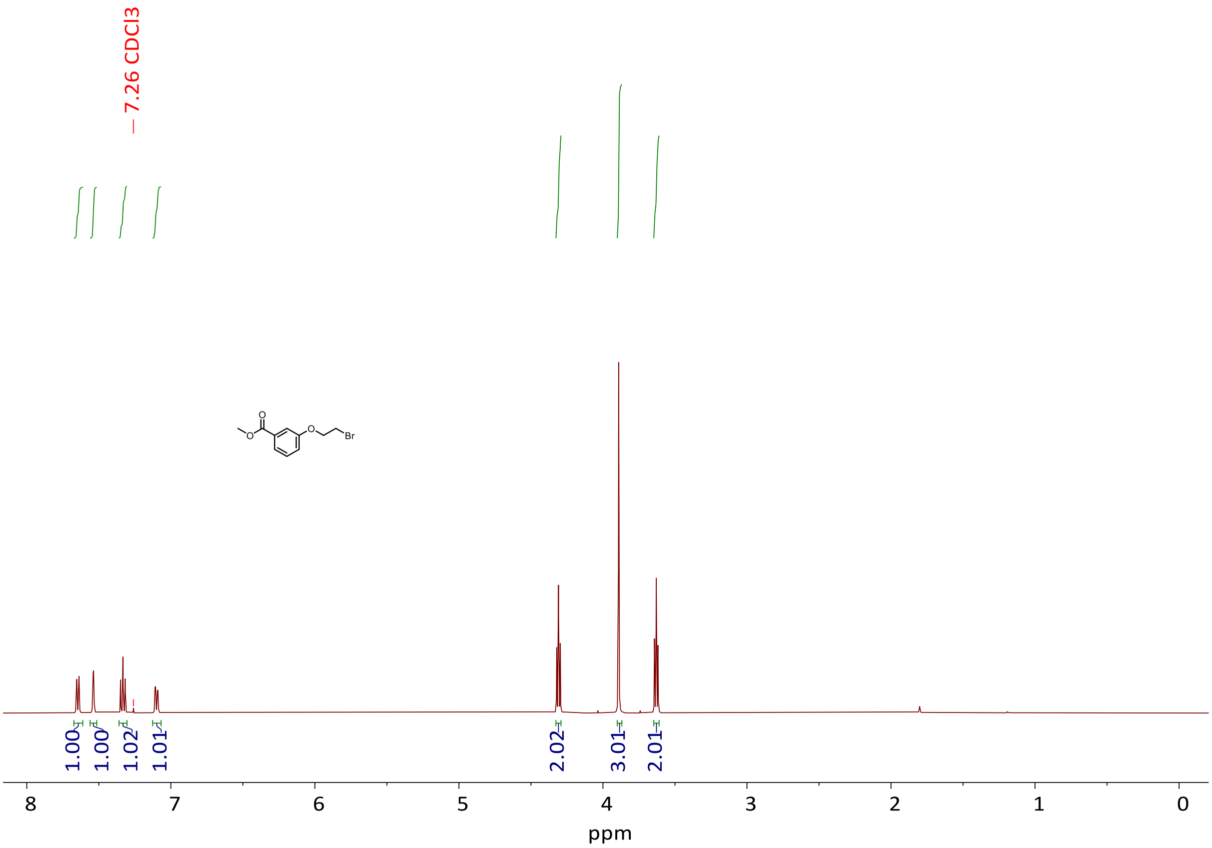

Spectrum S13. ^{1}H NMR (500 MHz, $CDCl_3$, 298 K) spectrum of compound **S5**.

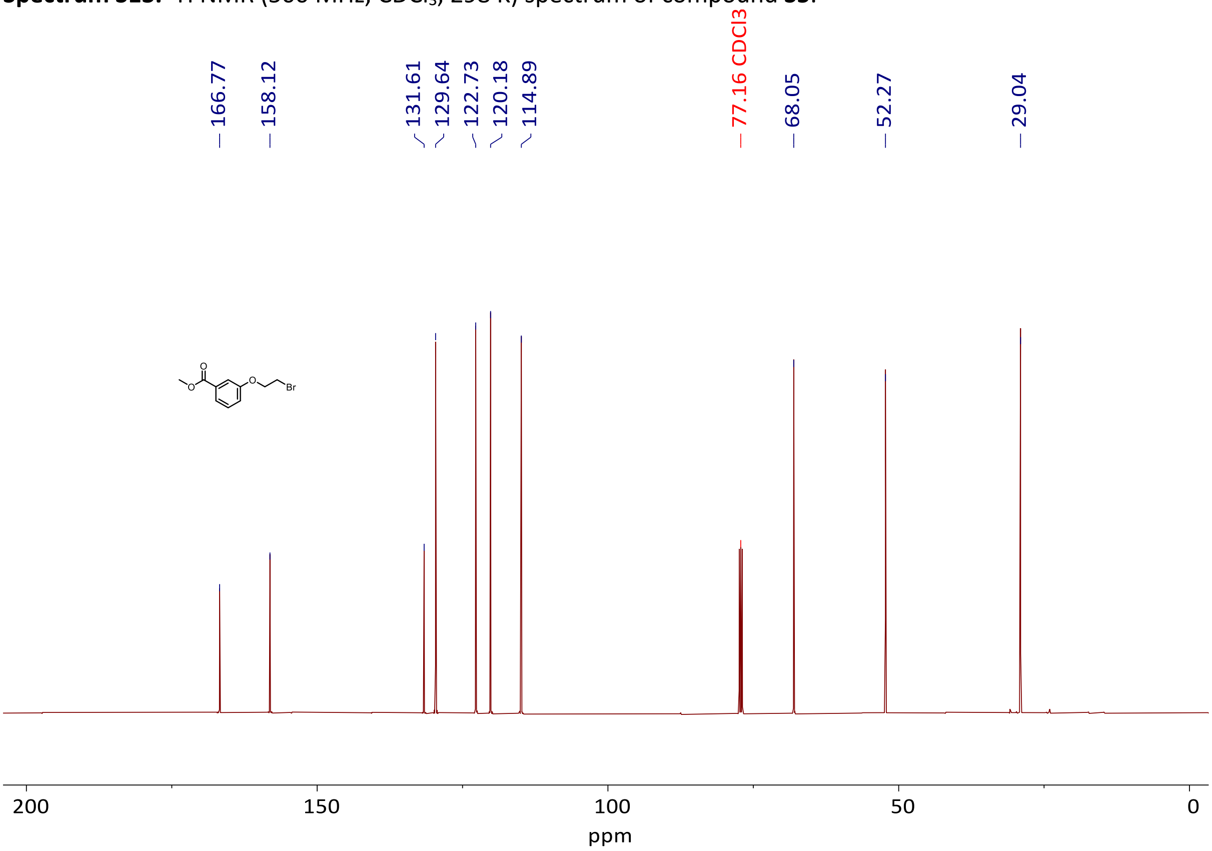

Spectrum S14. ^{13}C NMR (126 MHz, $CDCl_3$, 298 K) spectrum of compound **S5**.

9.1.8 Spectra of S6

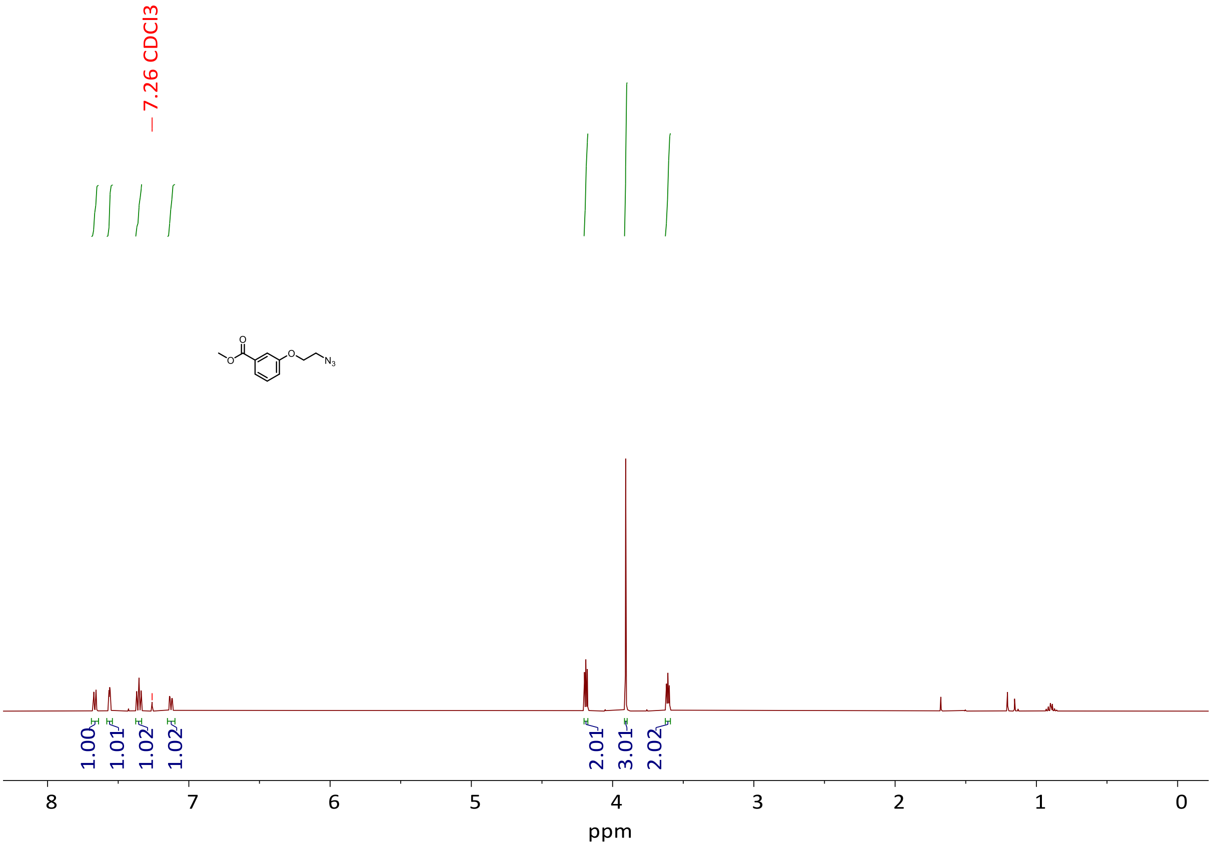

Spectrum S15. ^{1}H NMR (500 MHz, $CDCl_3$, 298 K) spectrum of compound **S6**.

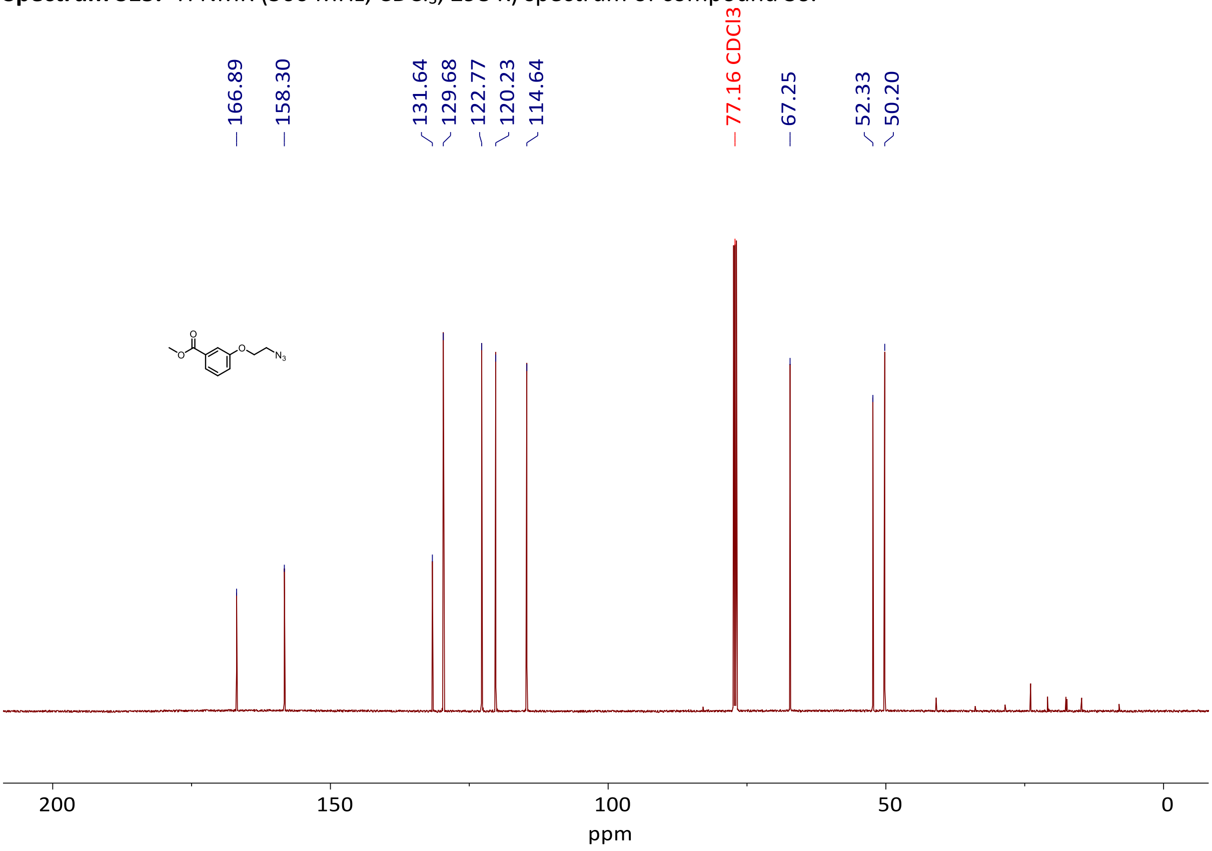

Spectrum S16. ^{13}C NMR (126 MHz, $CDCl_3$, 298 K) spectrum of compound **S6**.

9.1.9 Spectra of S7

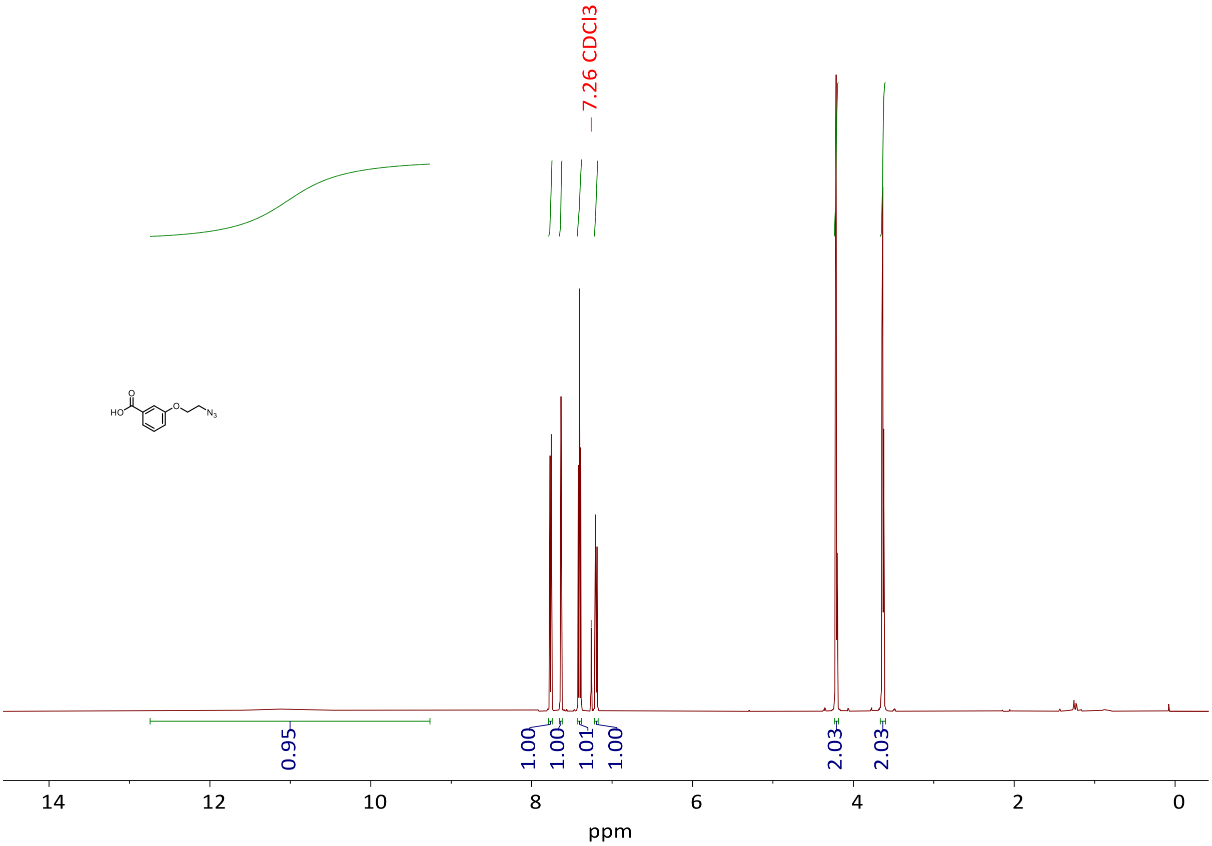

Spectrum S17. ^{1}H NMR (500 MHz, $CDCl_3$, 298 K) spectrum of compound **S7**.

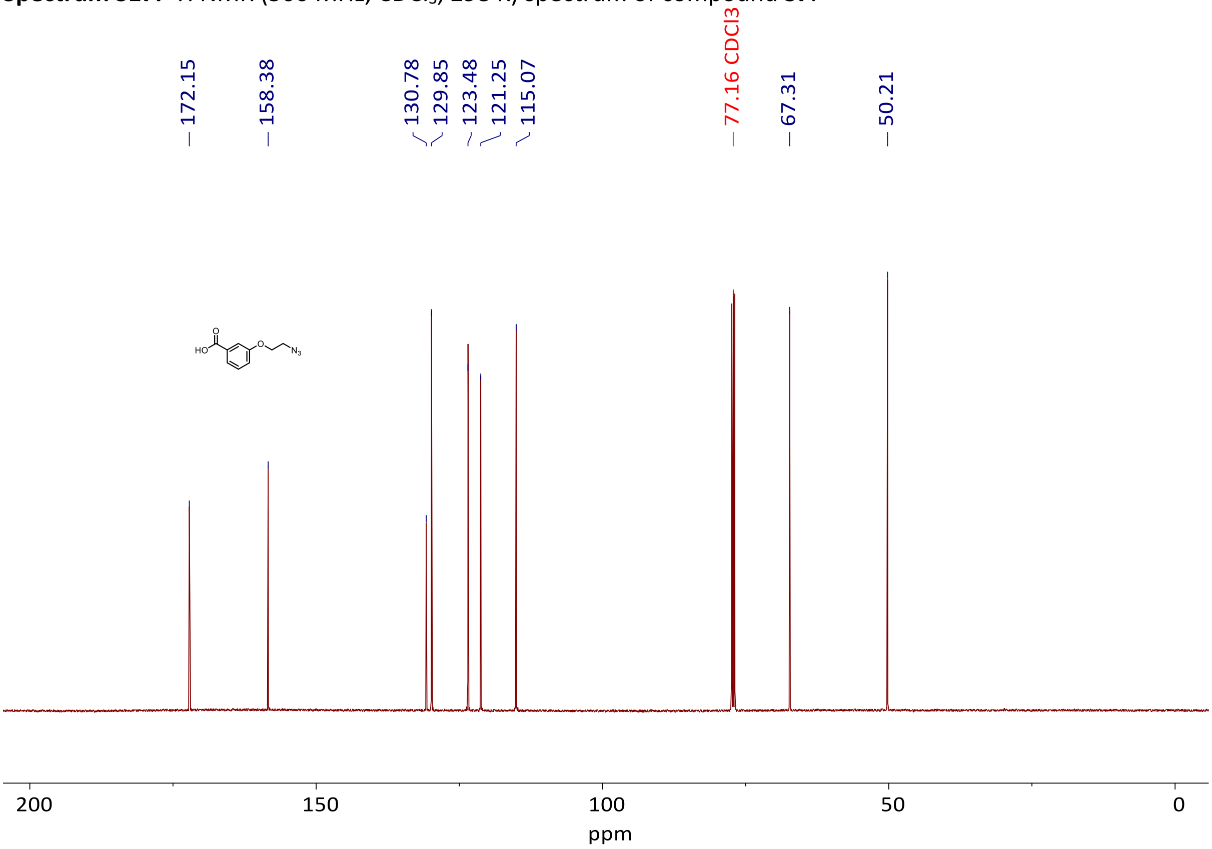

Spectrum S18. ^{13}C NMR (126 MHz, $CDCl_3$, 298 K) spectrum of compound **S7**.

9.1.10 Spectra of $S8_{exo}$

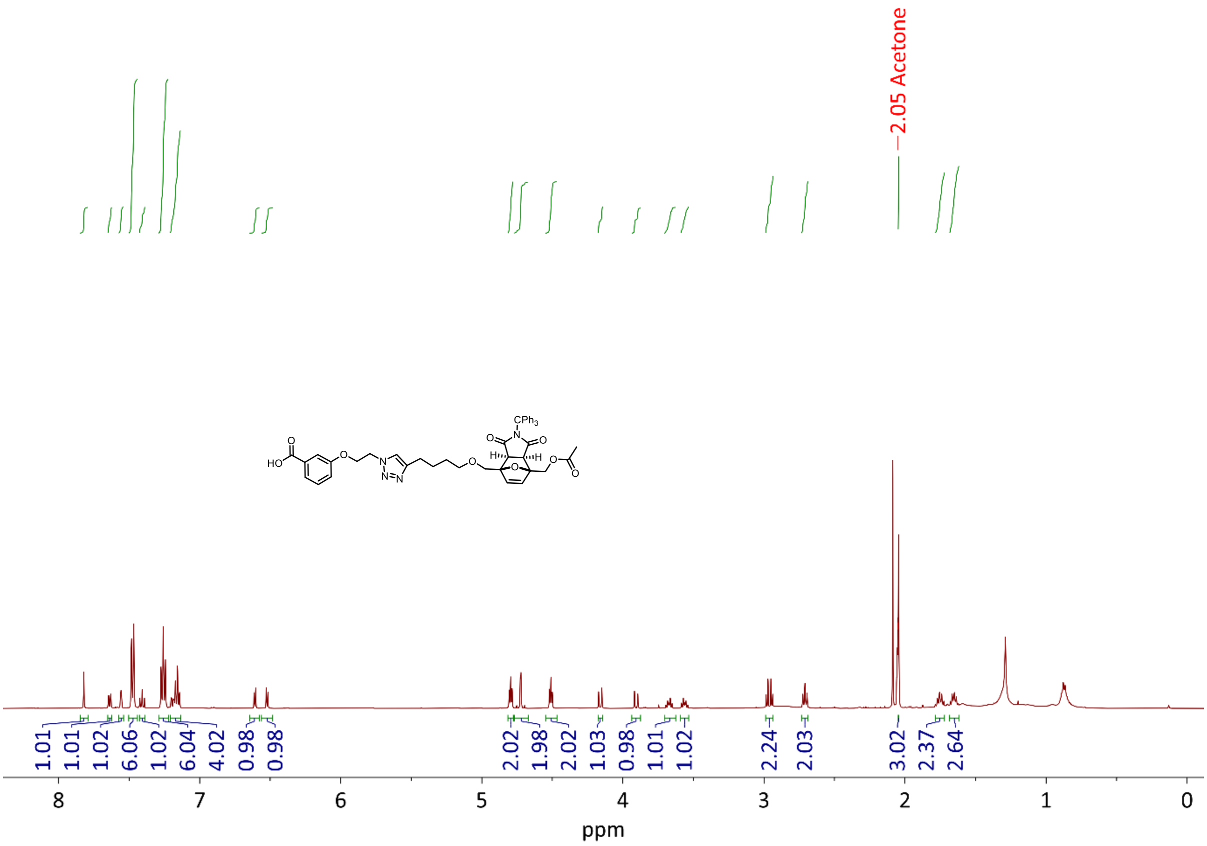

Spectrum S19. ^{1}H NMR (500 MHz, Acetone-d_6, 298 K) spectrum of compound $\mathbf{S8}_{exo}$.

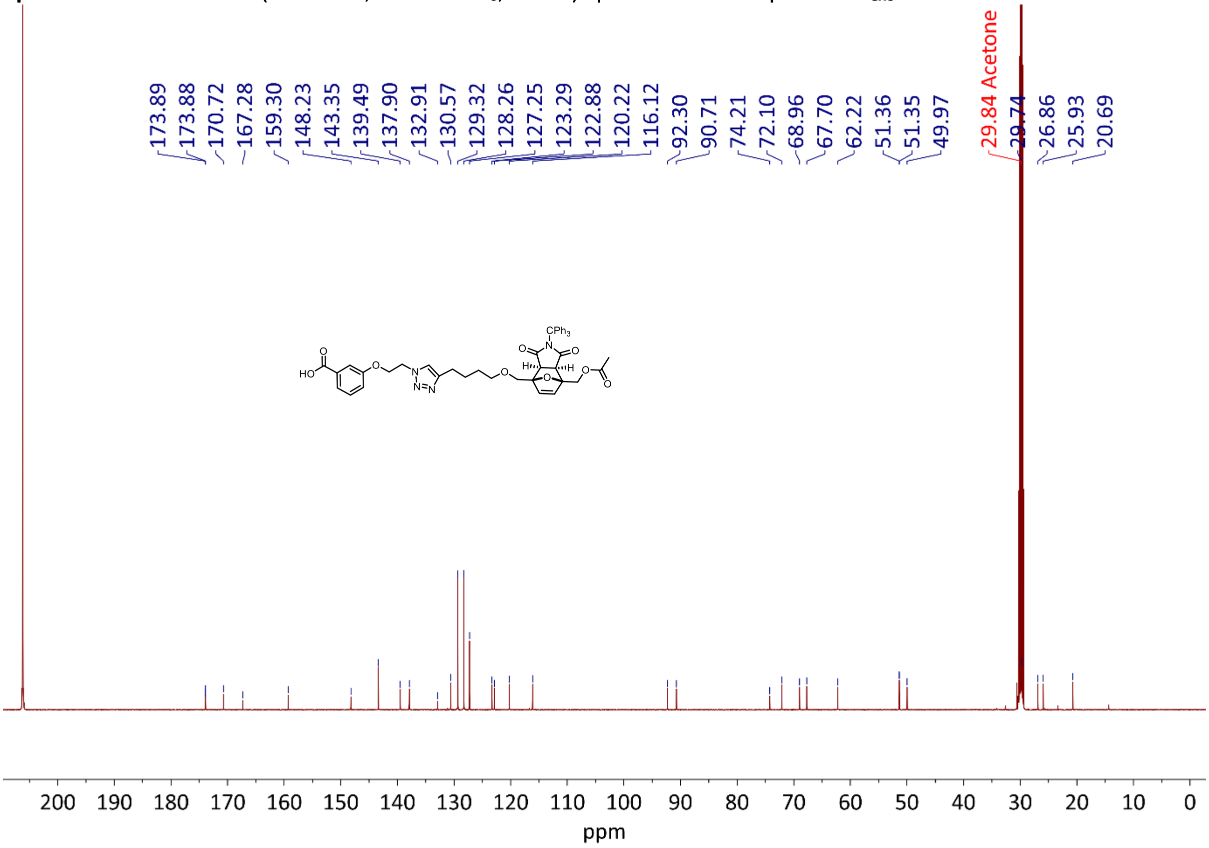

Spectrum S20. ^{13}C NMR (126 MHz, Acetone-d_6, 298 K) spectrum of compound $\mathbf{S8}_{exo}$.

9.1.11 Spectra of S8$_{endo}$

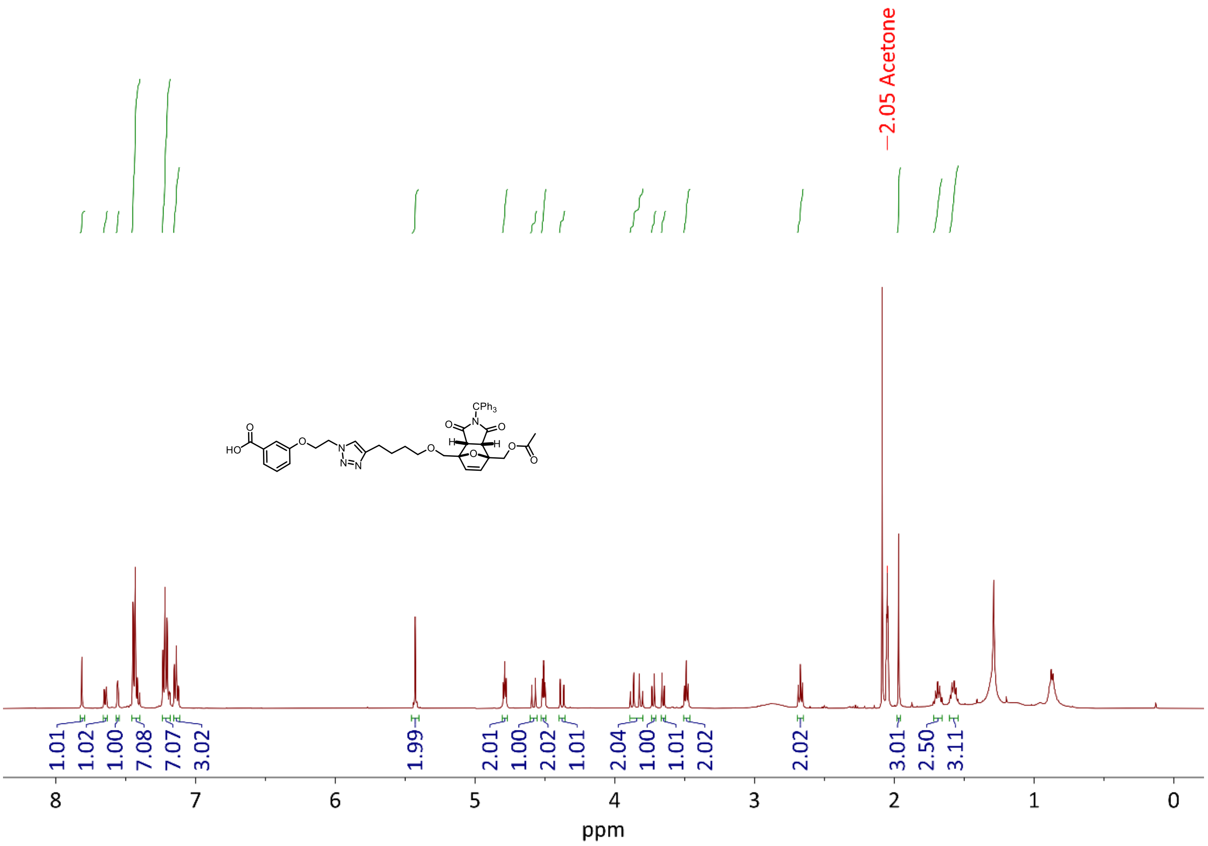

Spectrum S21. ^{1}H NMR (500 MHz, Acetone-d_6, 298 K) spectrum of compound **S8$_{endo}$**.

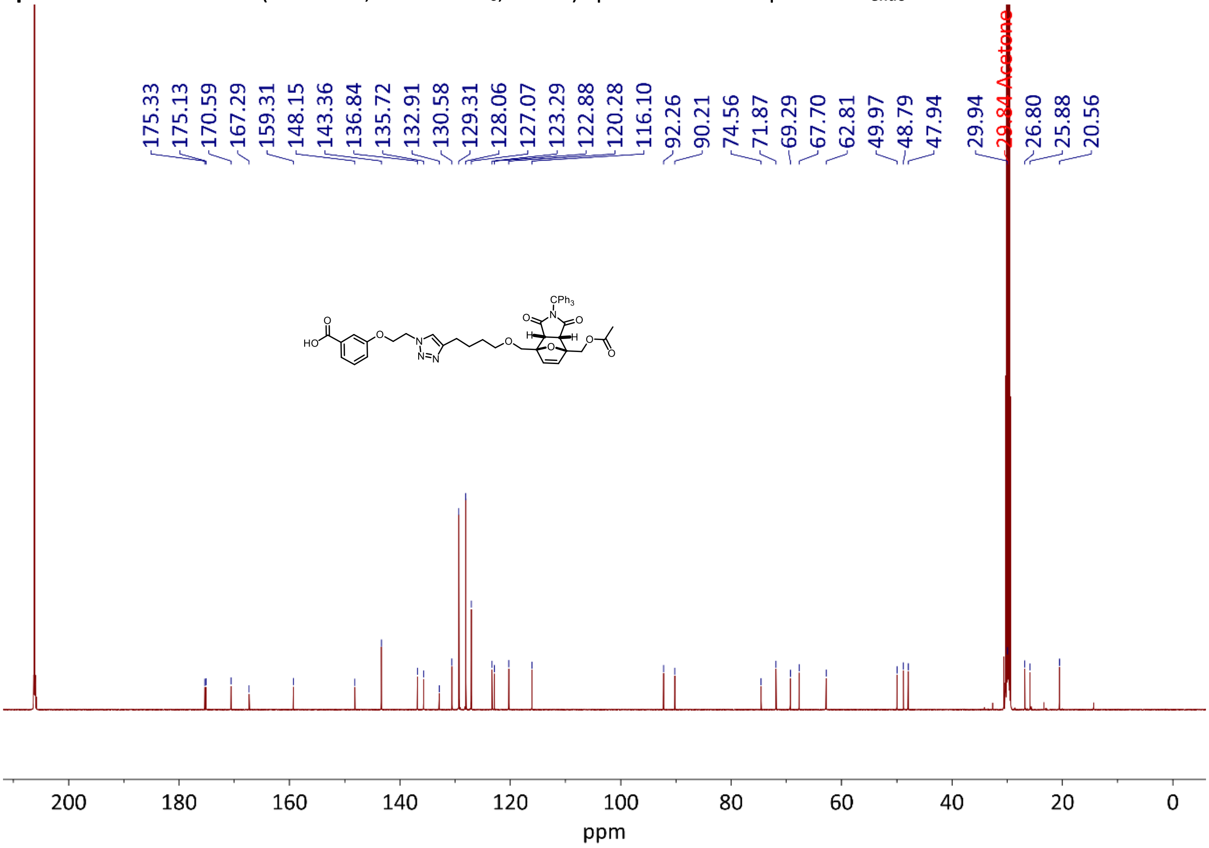

Spectrum S22. ^{13}C NMR (126 MHz, Acetone-d_6, 298 K) spectrum of compound **S8$_{endo}$**.

9.1.12 Spectra of S9

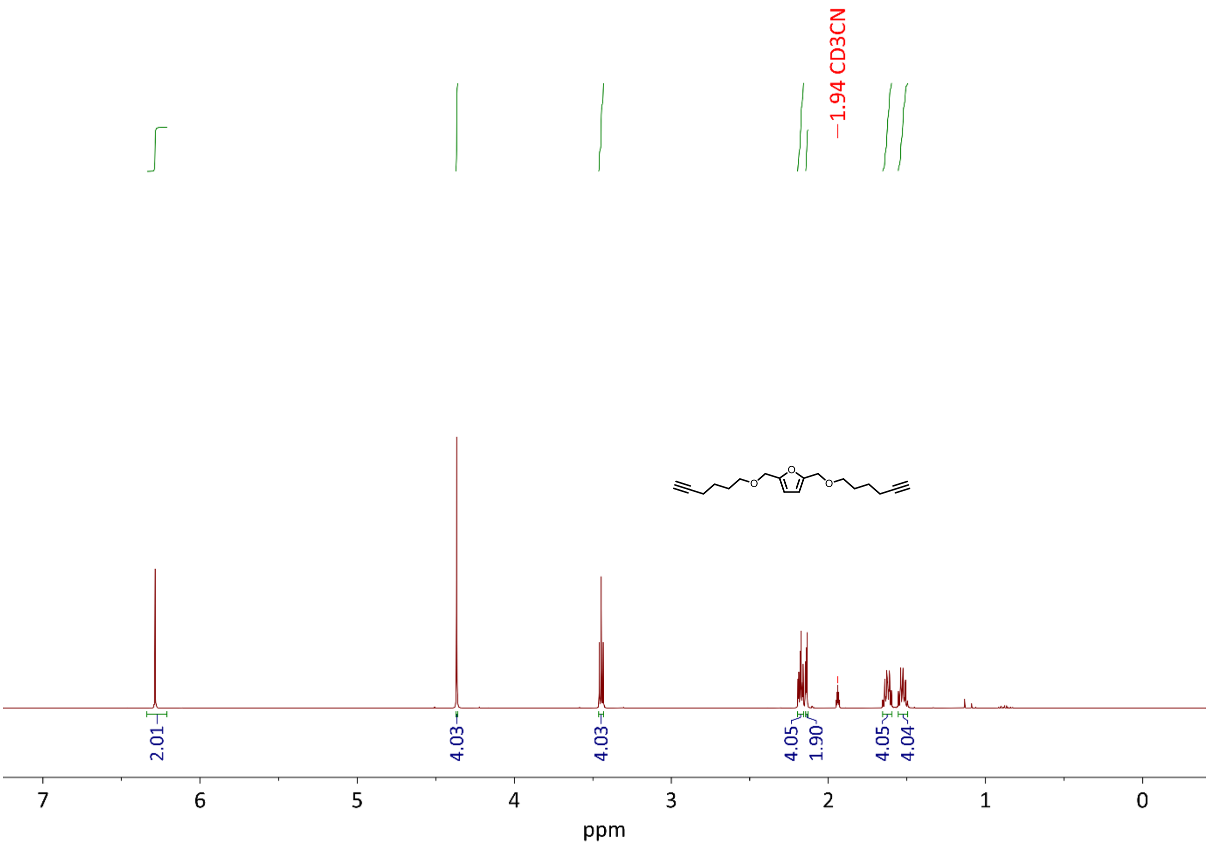

Spectrum S23. ^{1}H NMR (500 MHz, CD_3CN, 298 K) spectrum of compound **S9**.

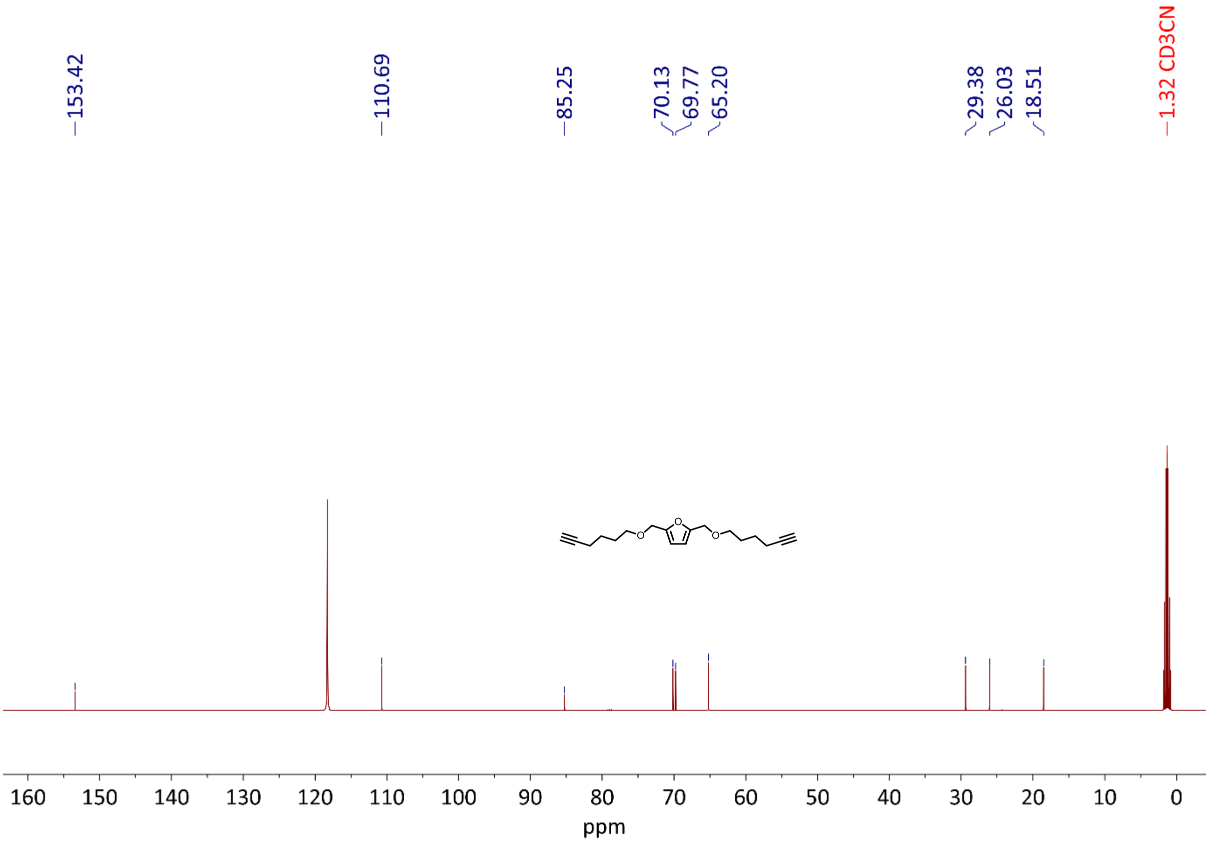

Spectrum S24. ^{13}C NMR (126 MHz, CD_3CN, 298 K) spectrum of compound **S9**.

9.1.13 Spectra of S10

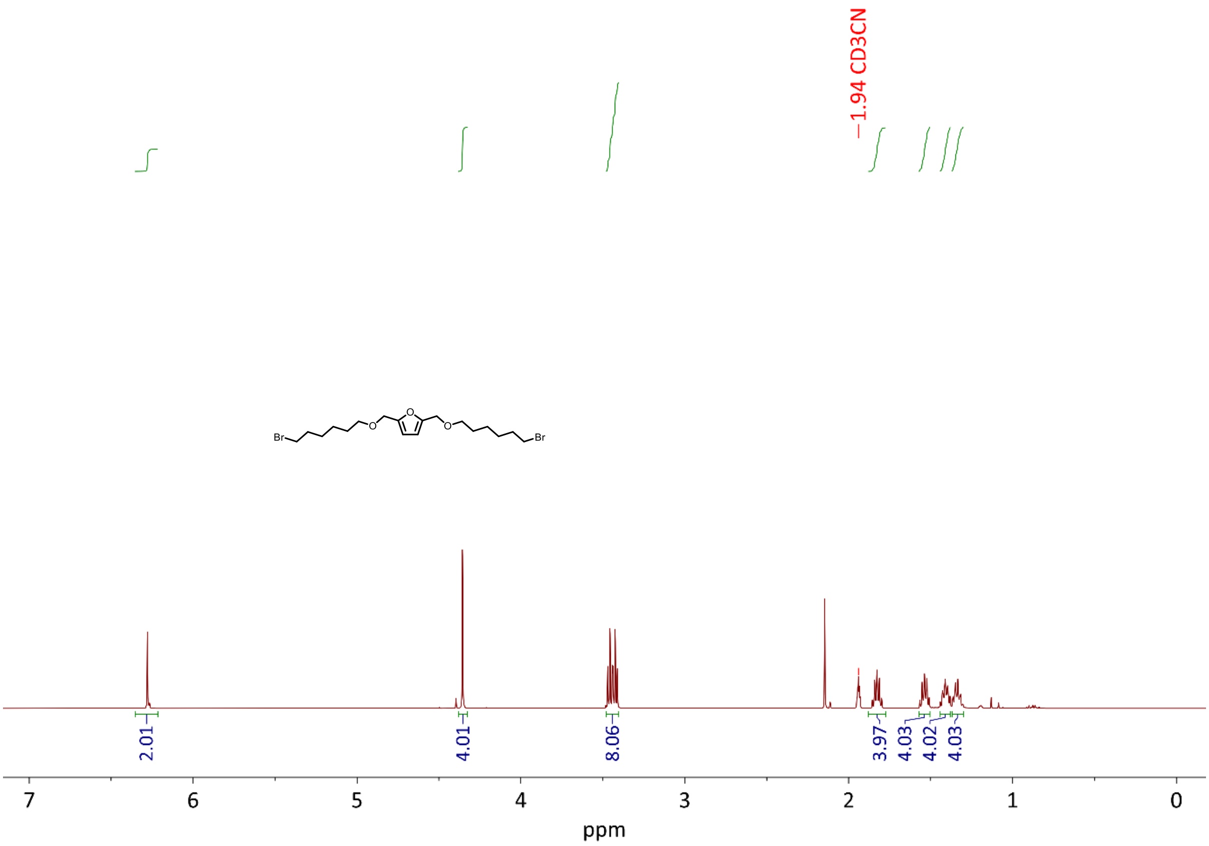

Spectrum S25. ^{1}H NMR (500 MHz, CD_3CN, 298 K) spectrum of compound **S10**.

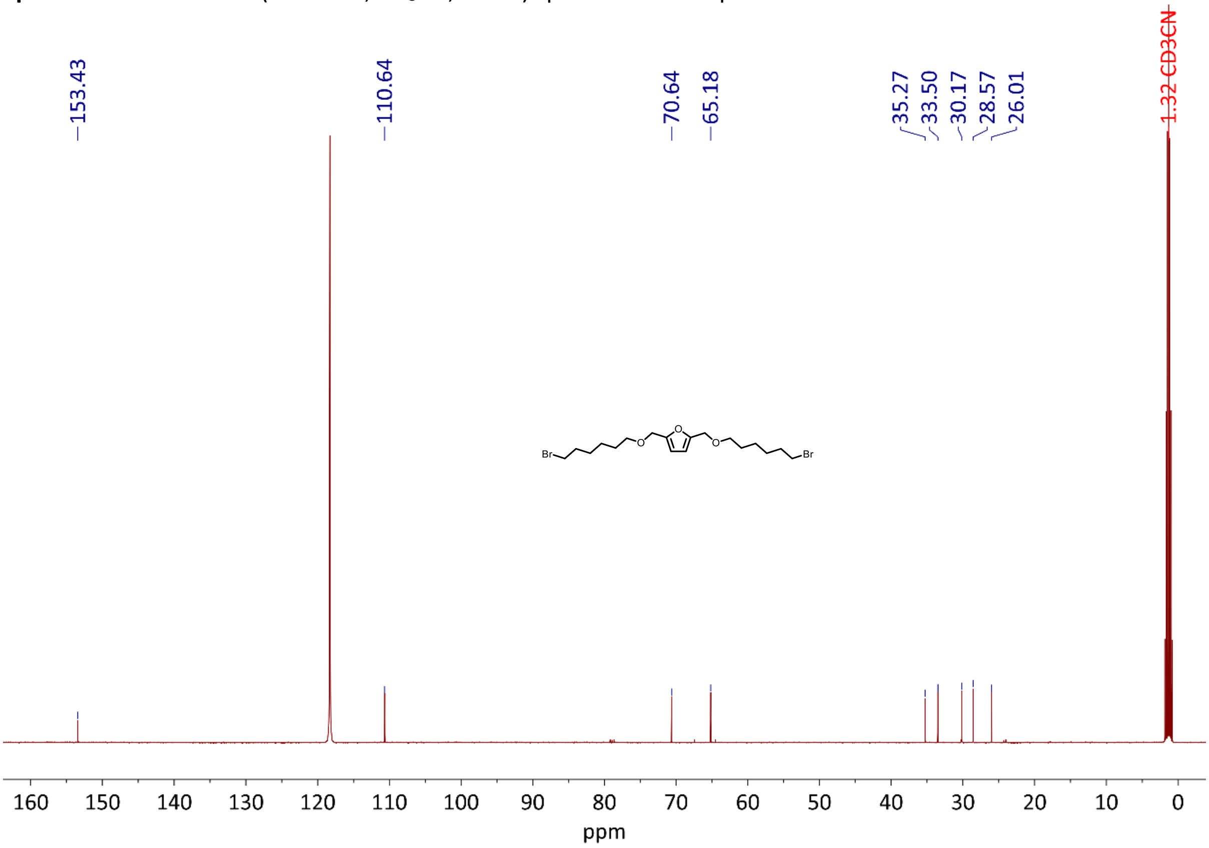

Spectrum S26. ^{13}C NMR (126 MHz, CD_3CN, 298 K) spectrum of compound **S10**.

9.1.14 Spectra of S11

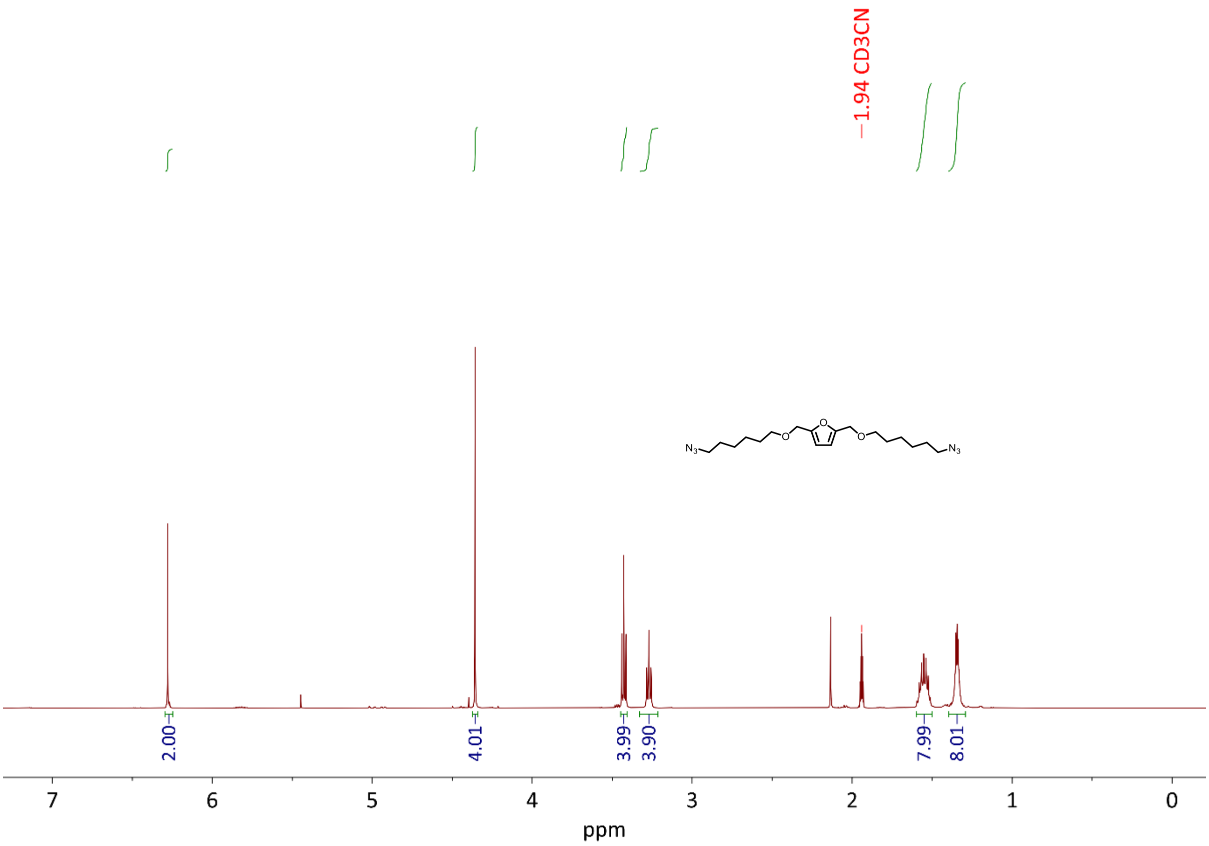

Spectrum S27. ^{1}H NMR (500 MHz, CD_3CN, 298 K) spectrum of compound **S11**.

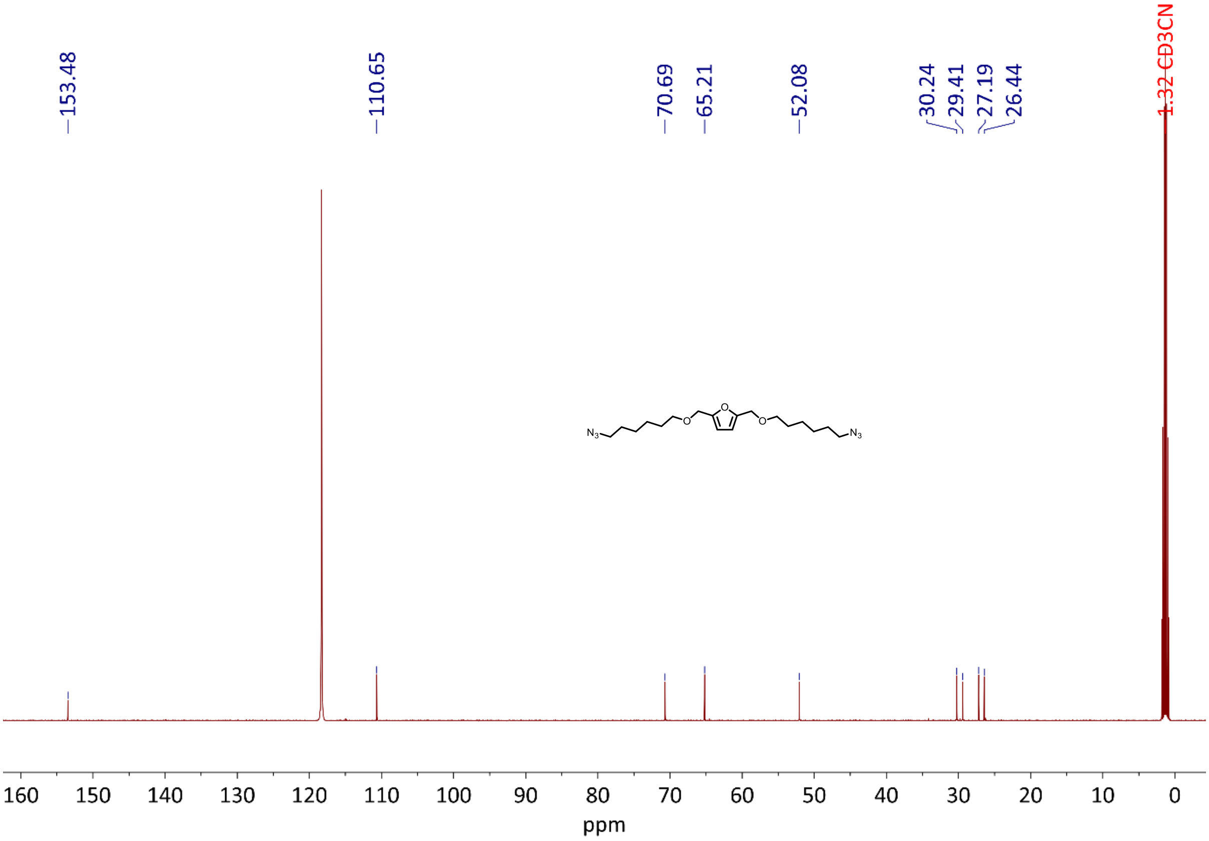

Spectrum S28. ^{13}C NMR (126 MHz, CD_3CN, 298 K) spectrum of compound **S11**.

9.1.15 Spectra of S12

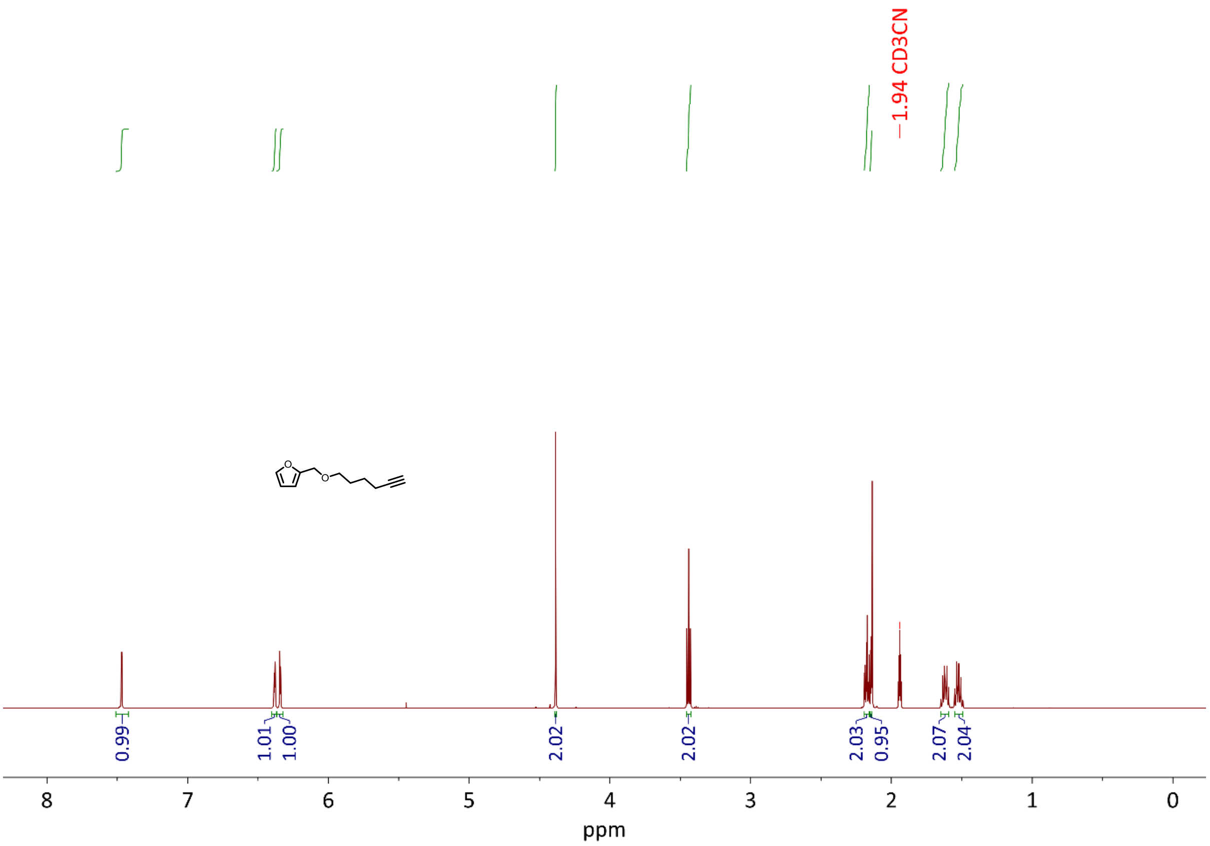

Spectrum S29. ^{1}H NMR (500 MHz, CD_3CN, 298 K) spectrum of compound **S12**.

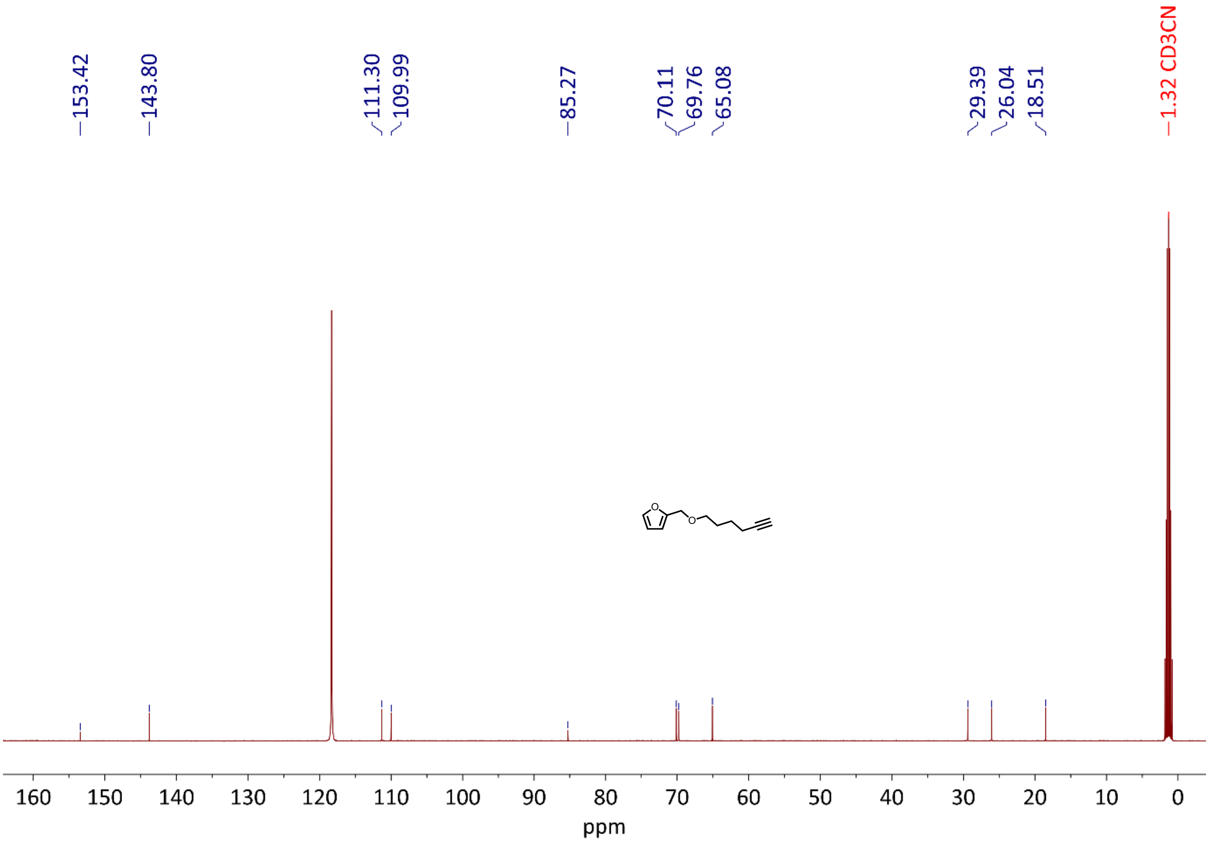

Spectrum S30. ^{13}C NMR (126 MHz, CD_3CN, 298 K) spectrum of compound **S12**.

9.1.16 Spectra of S13

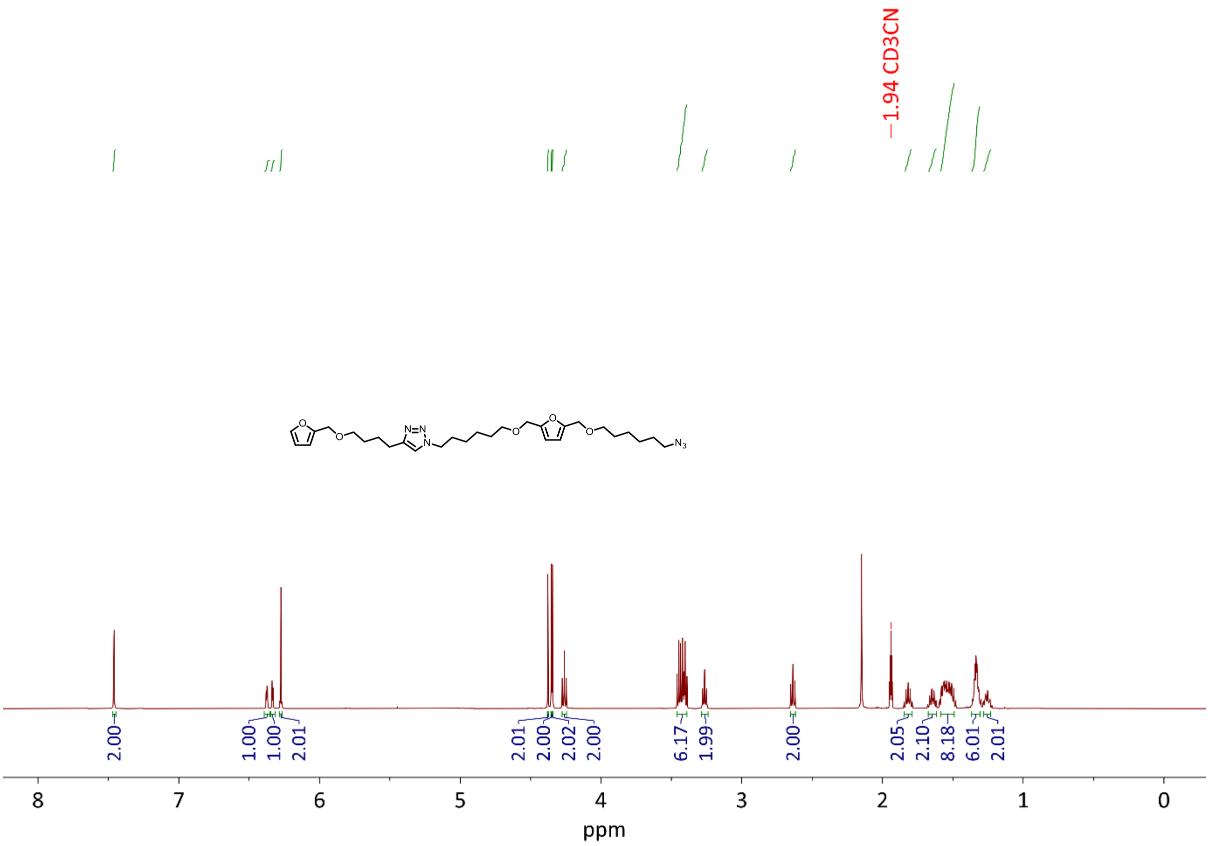

Spectrum S31. ^{1}H NMR (500 MHz, CD_3CN, 298 K) spectrum of compound **S13**.

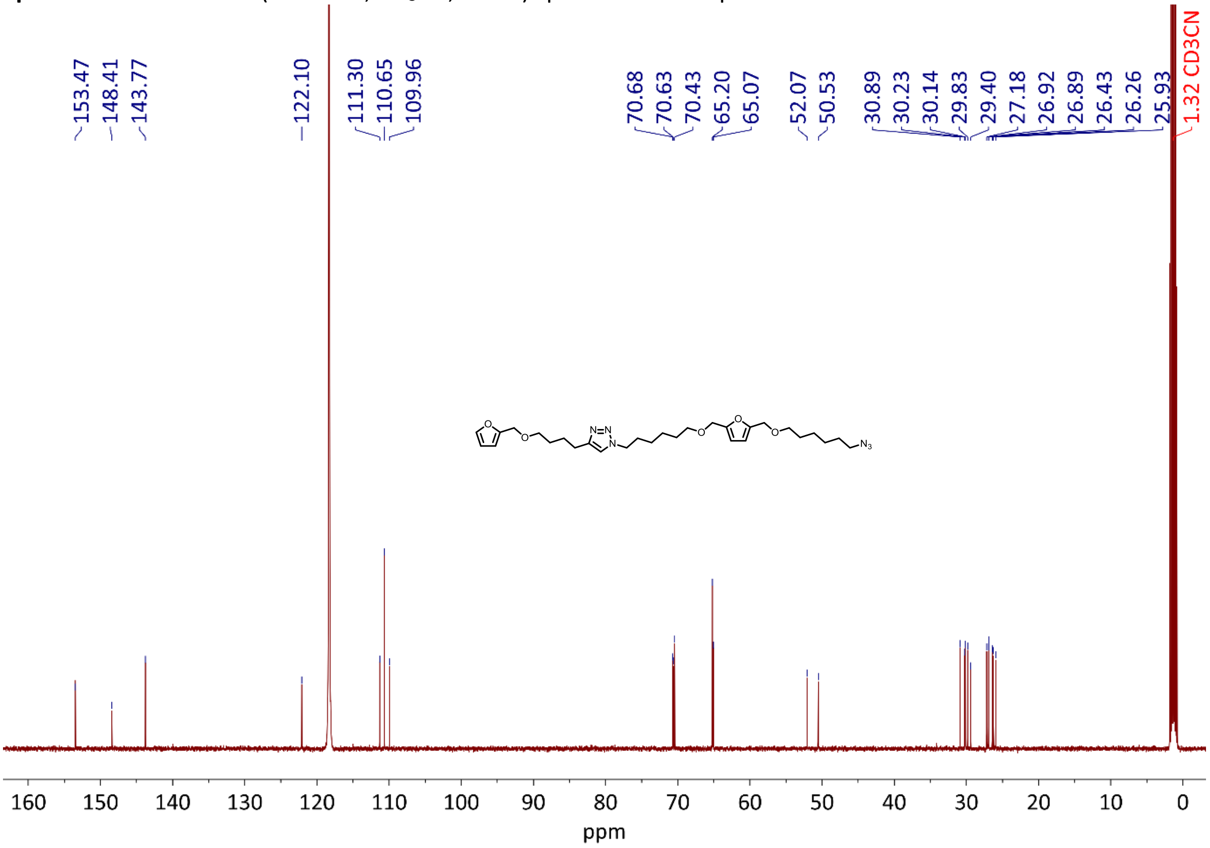

Spectrum S32. ^{13}C NMR (126 MHz, CD_3CN, 298 K) spectrum of compound **S13**.

9.1.17 Spectra of S14

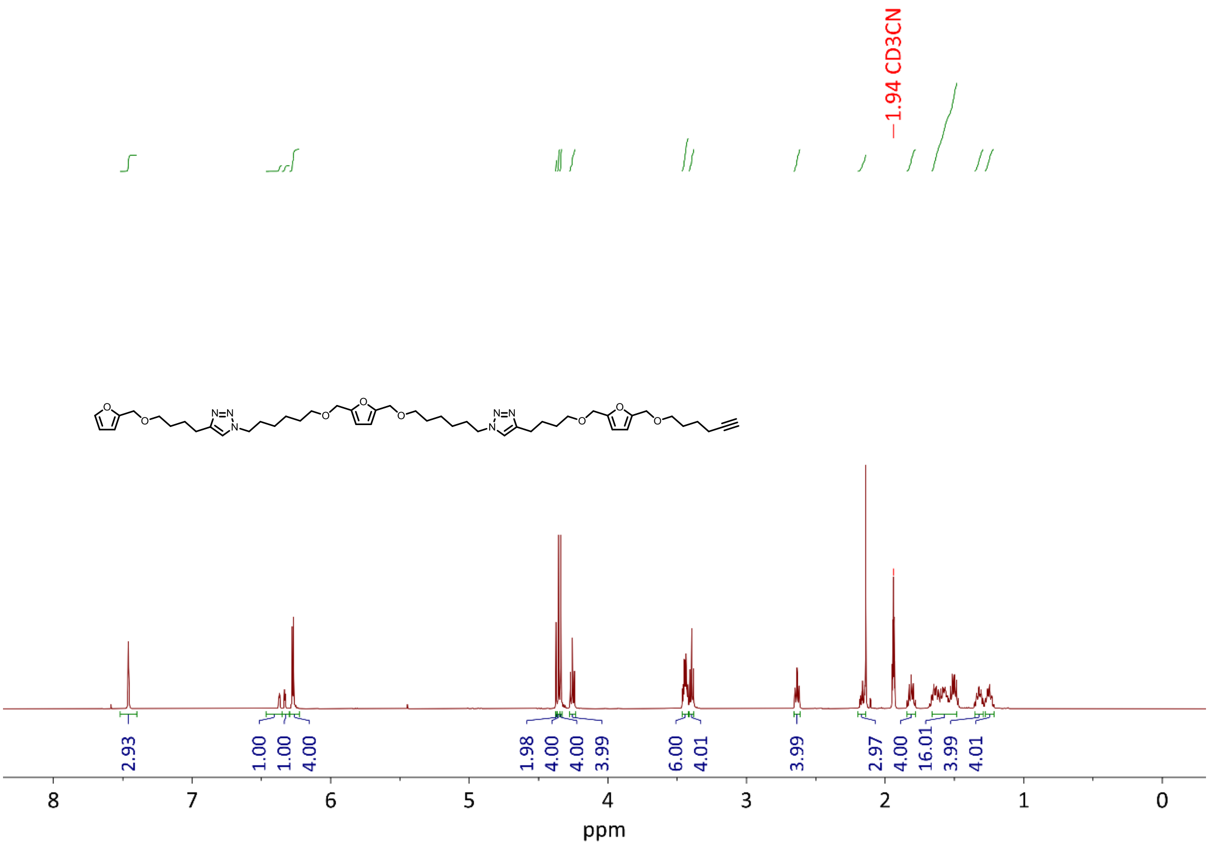

Spectrum S33. ^{1}H NMR (500 MHz, CD_3CN, 298 K) spectrum of compound **S14**.

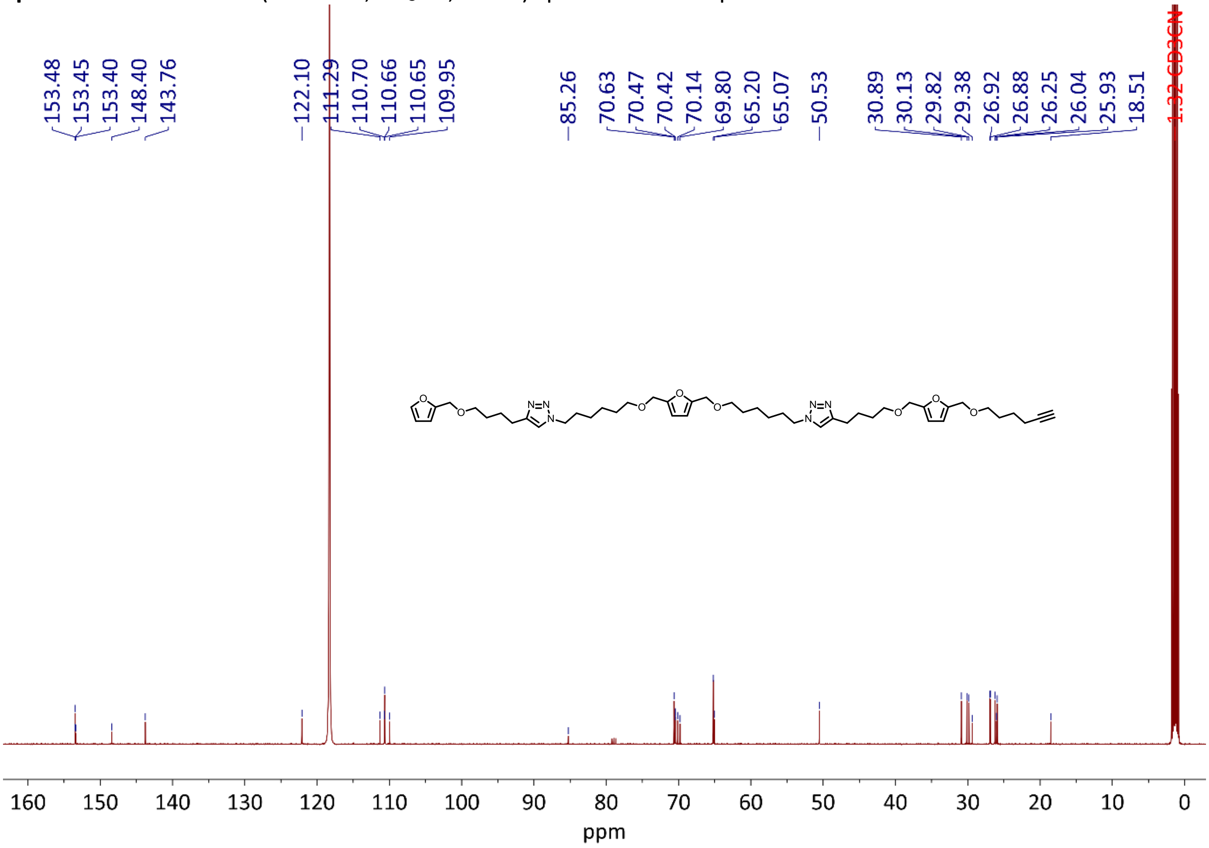

Spectrum S34. ^{13}C NMR (126 MHz, CD_3CN, 298 K) spectrum of compound **S14**.

9.1.18 Spectra of S15

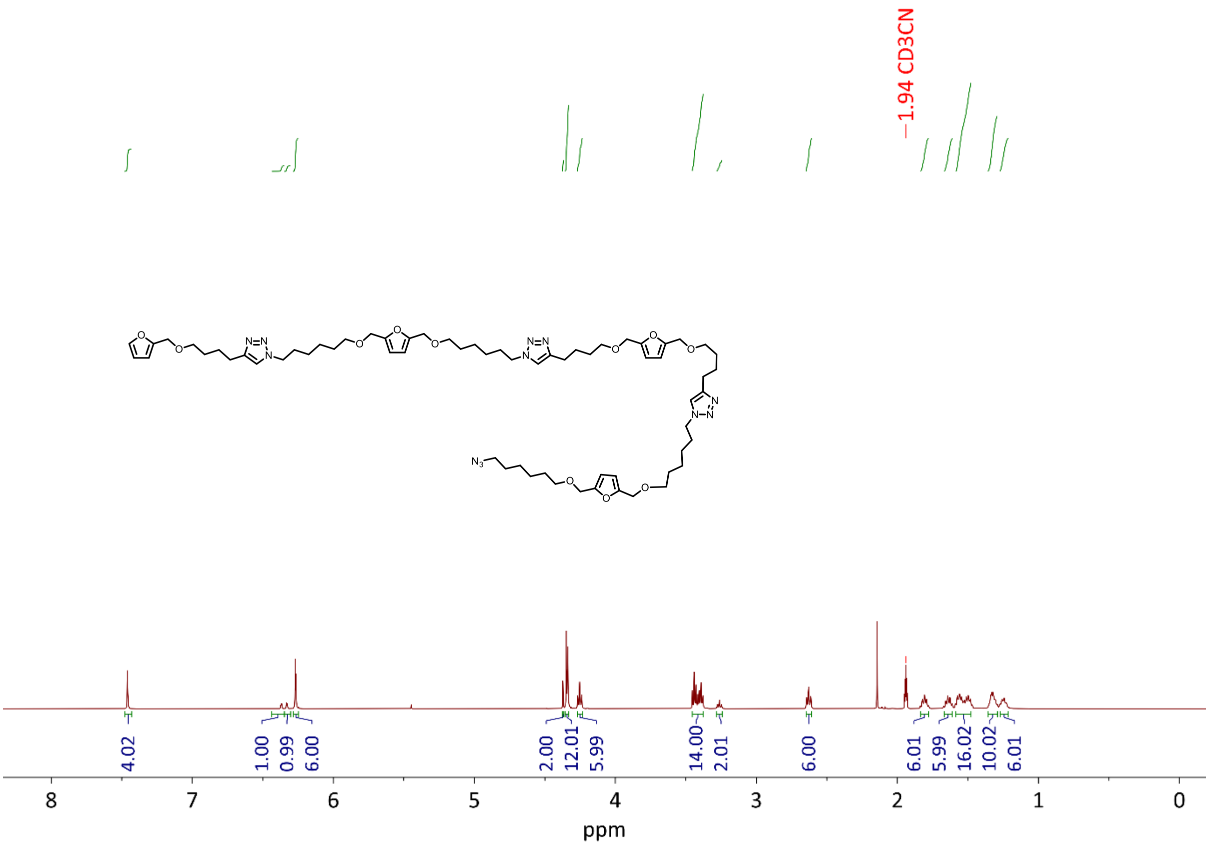

Spectrum S35. ^{1}H NMR (500 MHz, CD_3CN, 298 K) spectrum of compound **S15**.

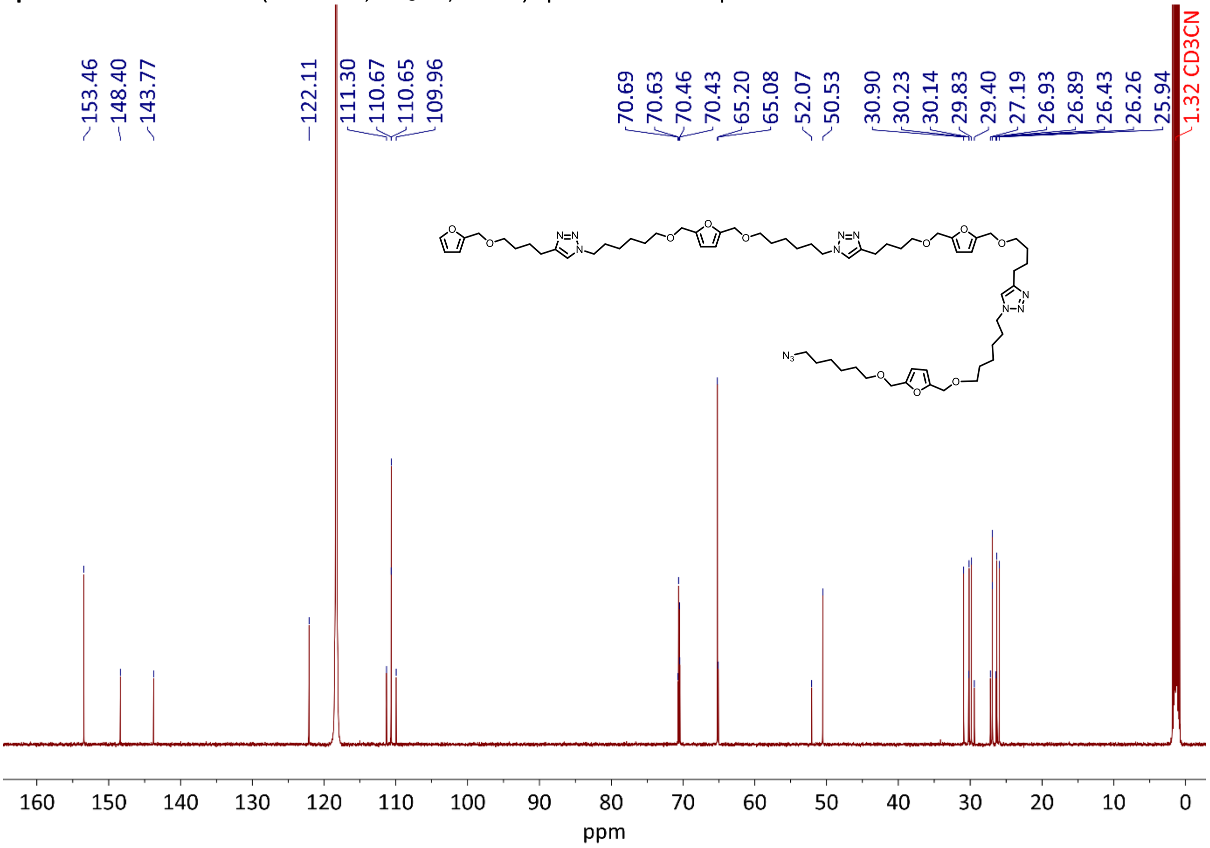

Spectrum S36. ^{13}C NMR (126 MHz, CD_3CN, 298 K) spectrum of compound **S15**.

9.1.19 Spectra of S16

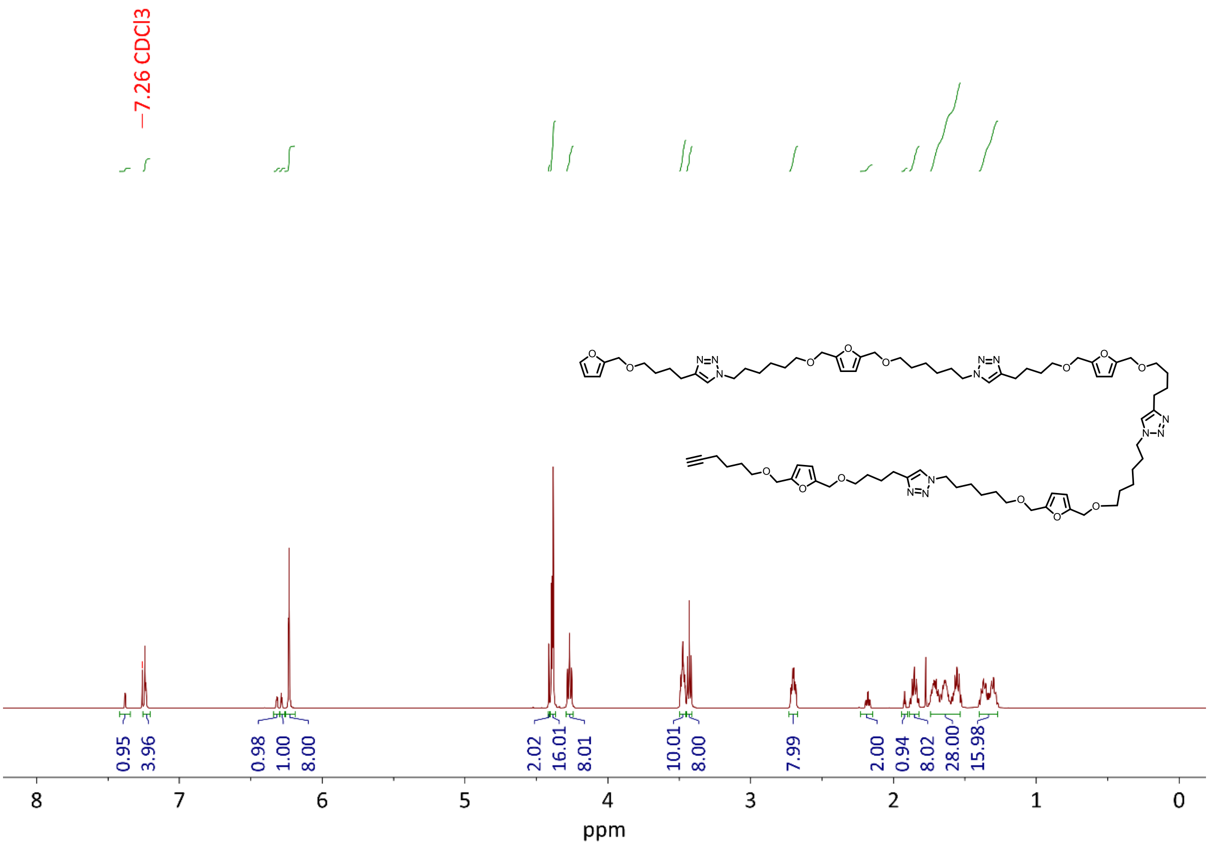

Spectrum S37. ^{1}H NMR (500 MHz, $CDCl_3$, 298 K) spectrum of compound **S16**.

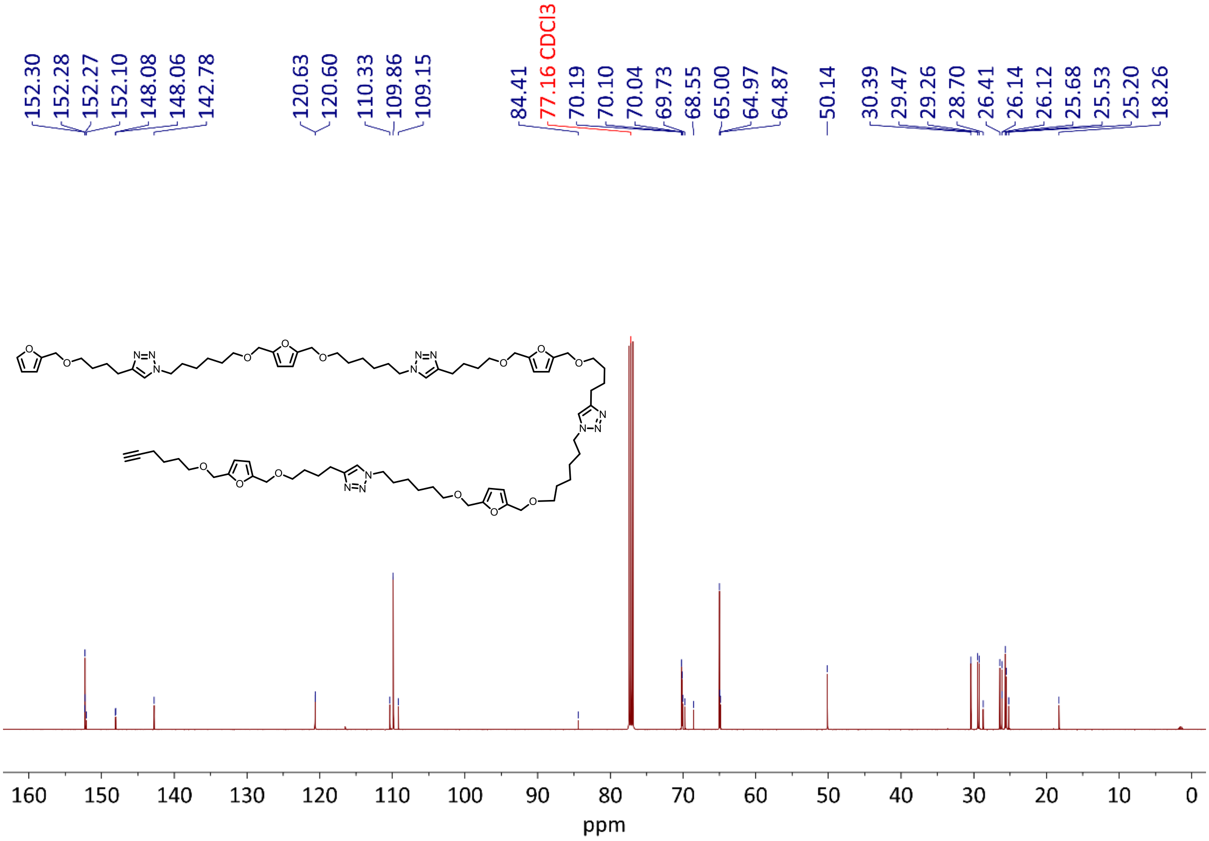

Spectrum S38. ^{13}C NMR (126 MHz, $CDCl_3$, 298 K) spectrum of compound **S16**.

9.1.20 Spectra of S17a

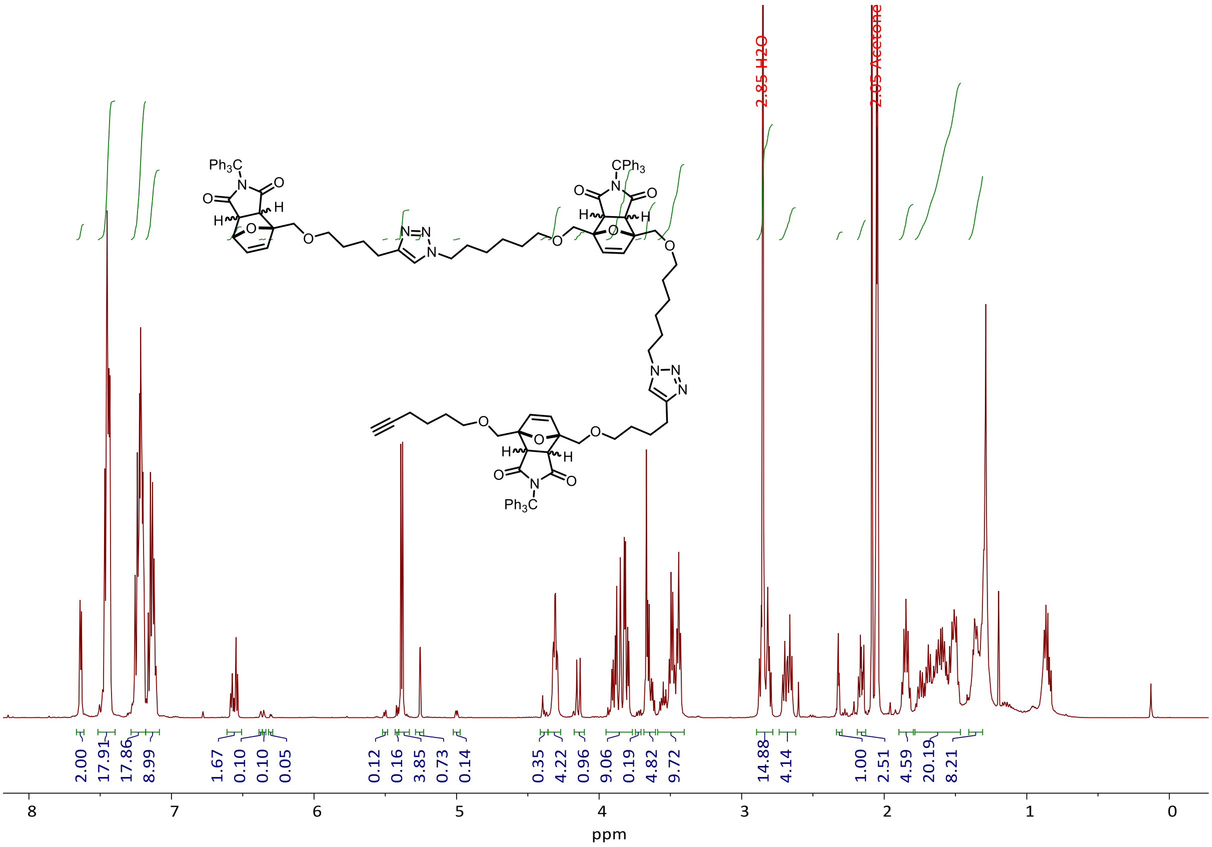

Spectrum S39. ^{1}H NMR (500 MHz, Acetone-d_6, 298 K) spectrum of compound **S17a**.

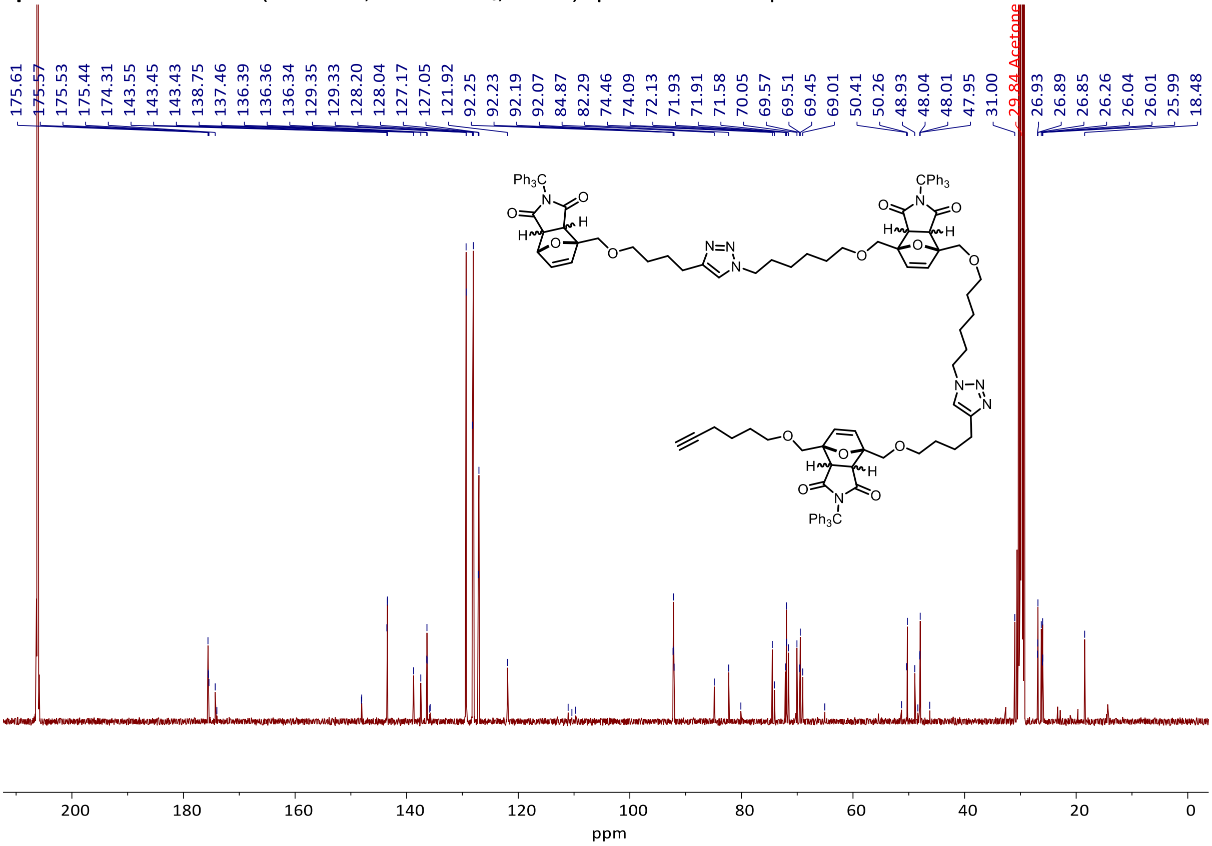

Spectrum S40. ^{13}C NMR (126 MHz, Acetone-d_6, 298 K) spectrum of compound **S17a**.

9.1.21 Spectra of S17b

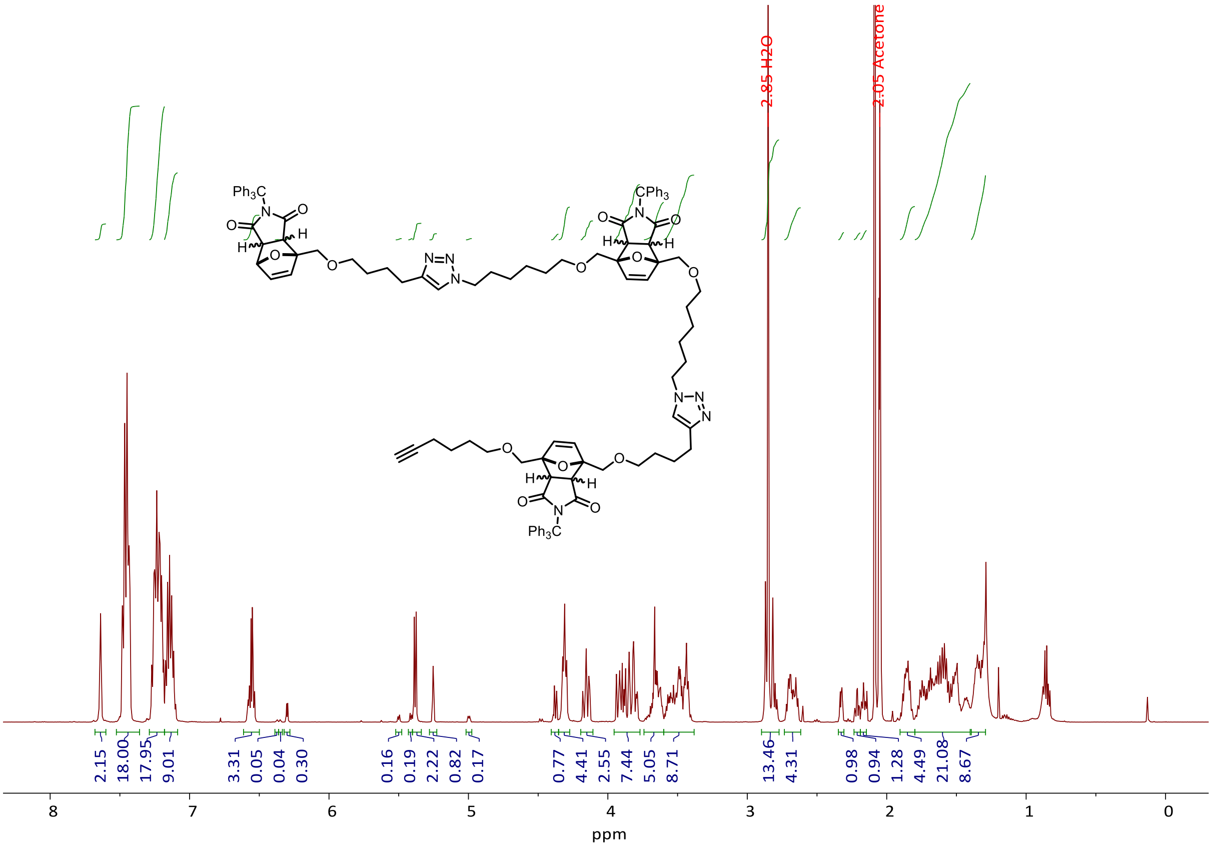

Spectrum S41. ^{1}H NMR (500 MHz, Acetone-d_6, 298 K) spectrum of compound **S17b**.

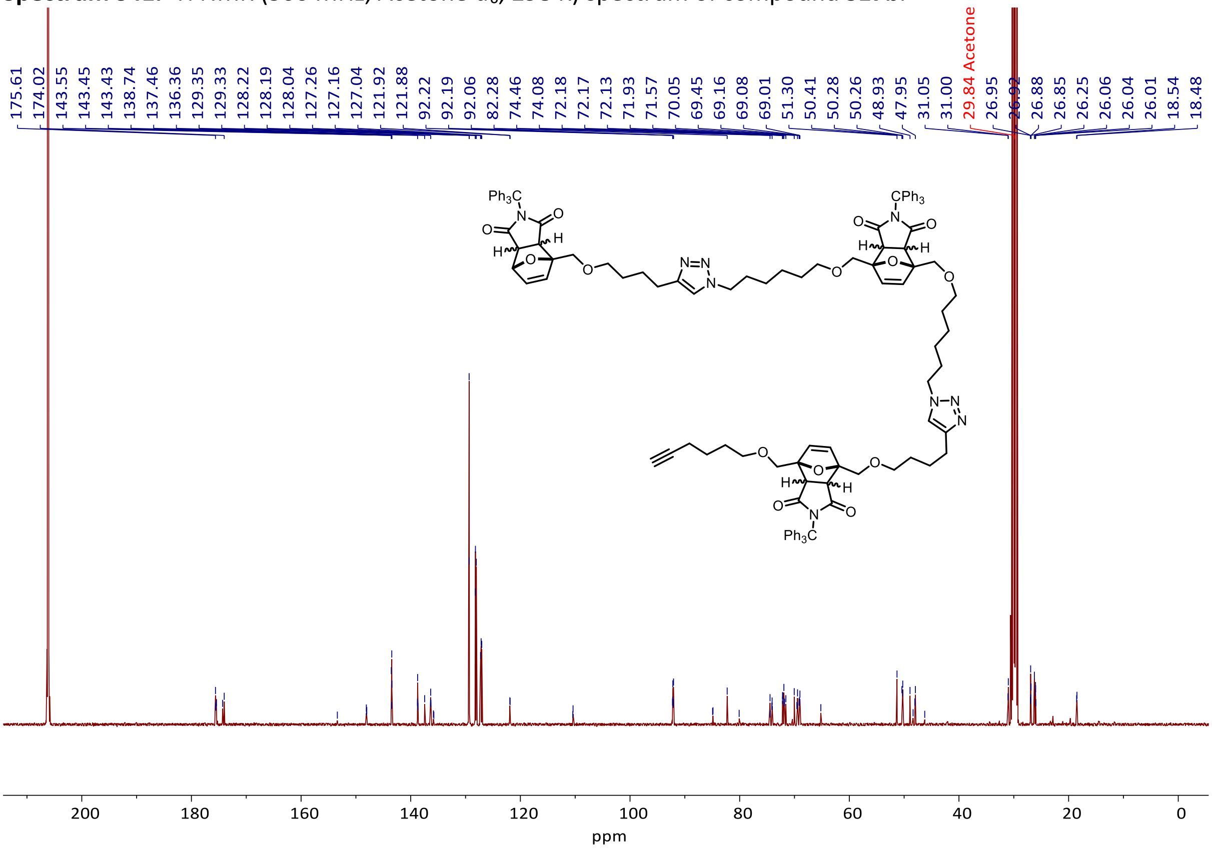

Spectrum S42. ^{13}C NMR (126 MHz, Acetone-d_6, 298 K) spectrum of compound **S17b**.

9.1.22 Spectra of S17c

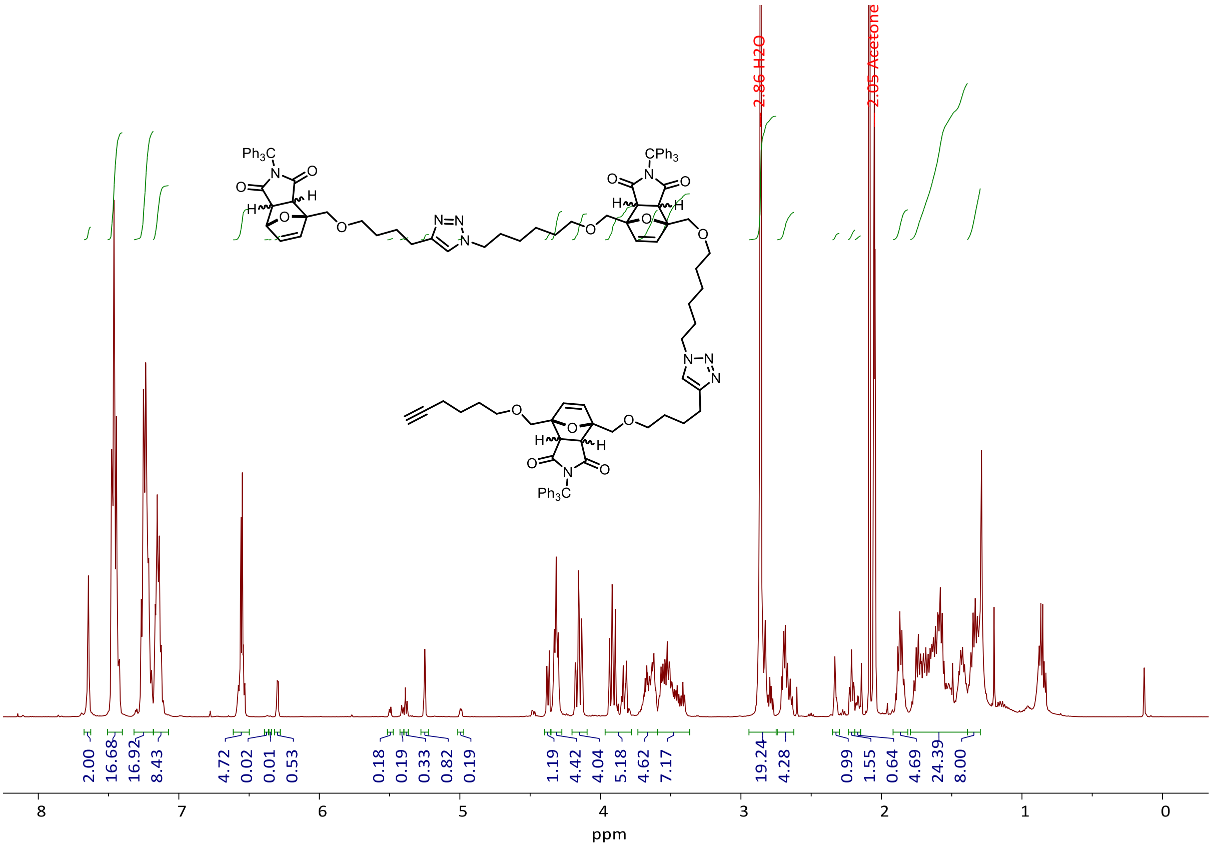

Spectrum S43. ^{1}H NMR (500 MHz, Acetone-d_6, 298 K) spectrum of compound **S17c**.

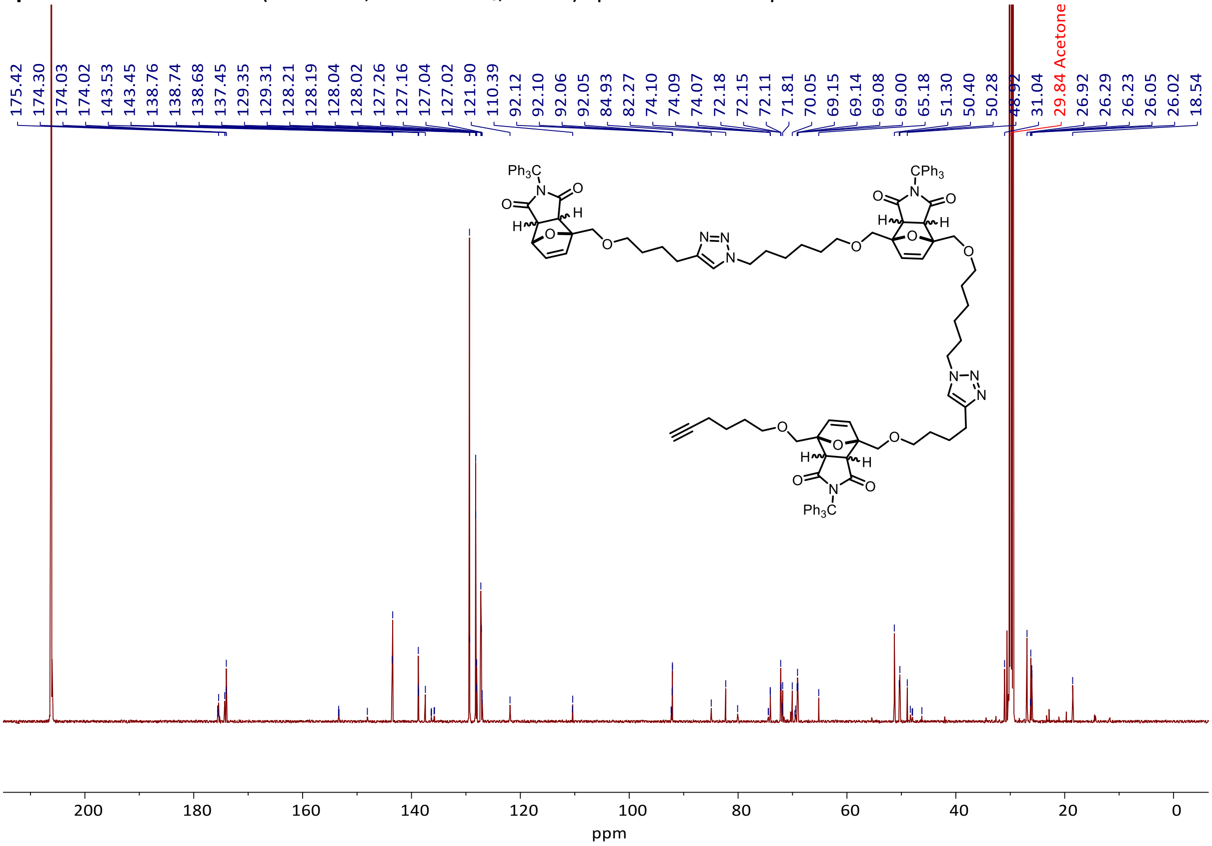

Spectrum S44. ^{13}C NMR (126 MHz, Acetone-d_6, 298 K) spectrum of compound **S17c**.

9.1.23 Spectra of S18a

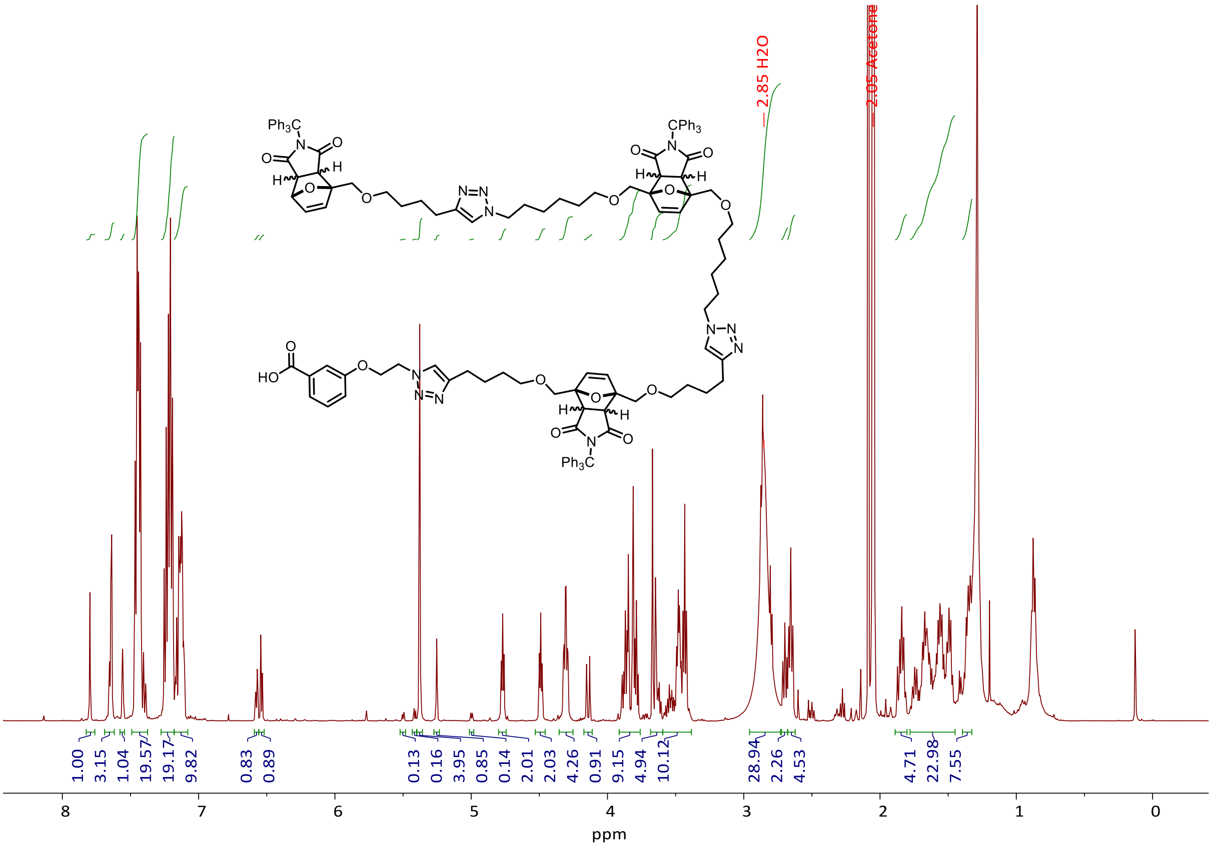

Spectrum S45. ^{1}H NMR (500 MHz, Acetone-d_6, 298 K) spectrum of compound **S18a**.

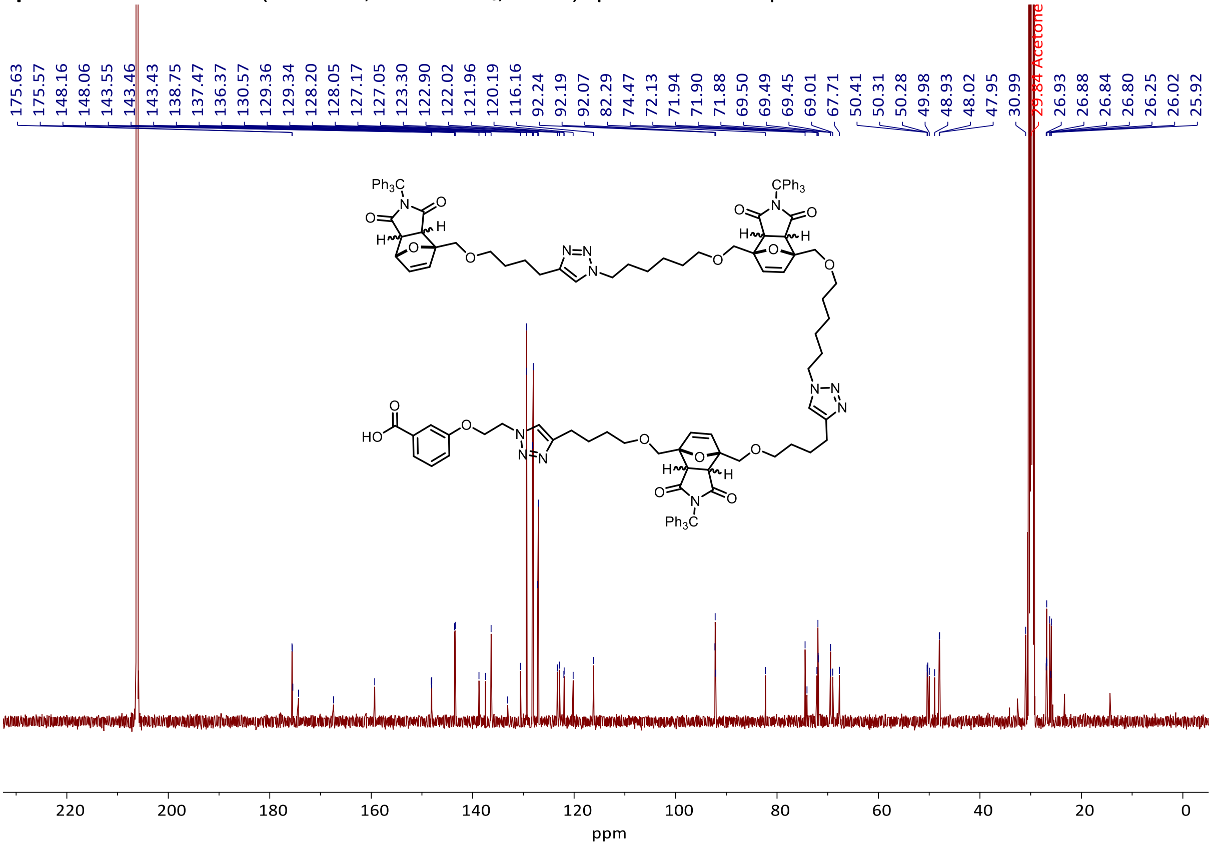

Spectrum S46. ^{13}C NMR (126 MHz, Acetone-d_6, 298 K) spectrum of compound **S18a**.

9.1.24 Spectra of S18b

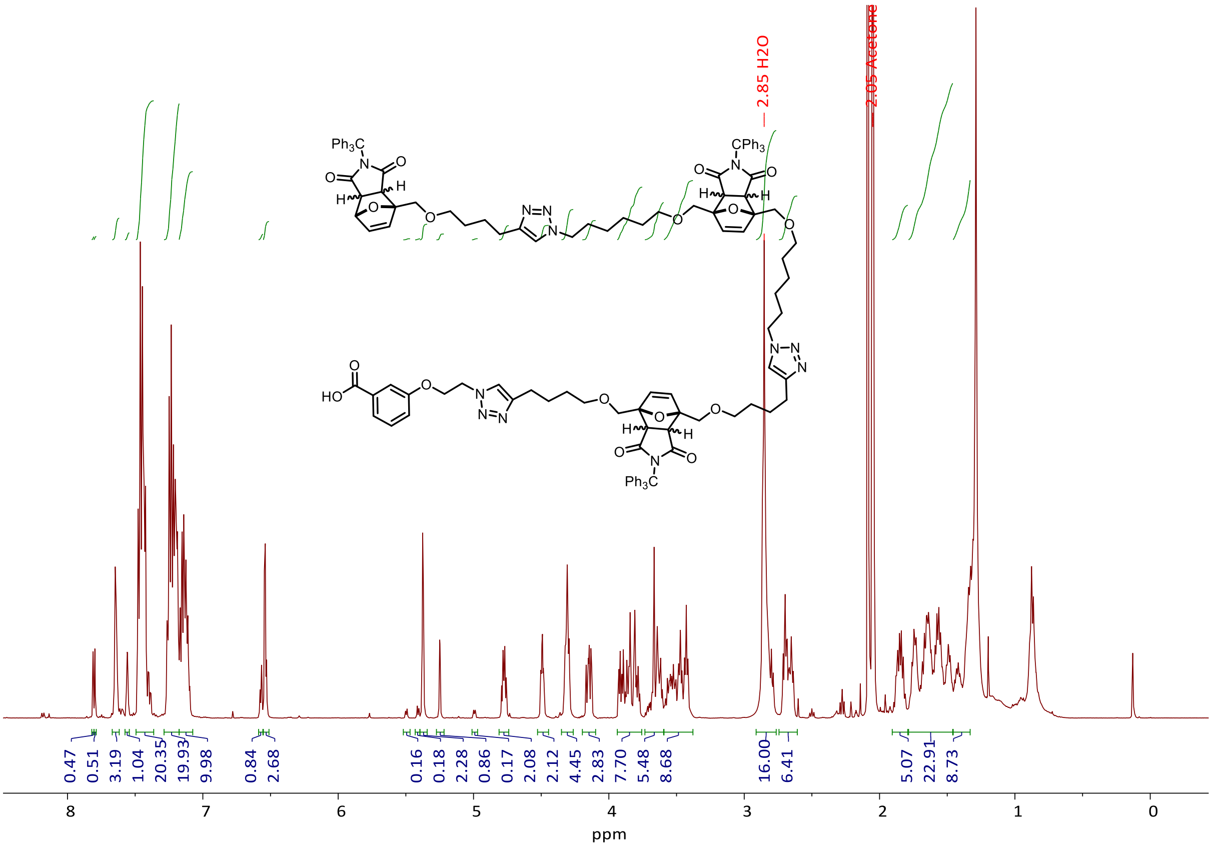

Spectrum S47. ^{1}H NMR (500 MHz, Acetone-d_6, 298 K) spectrum of compound **S18b**.

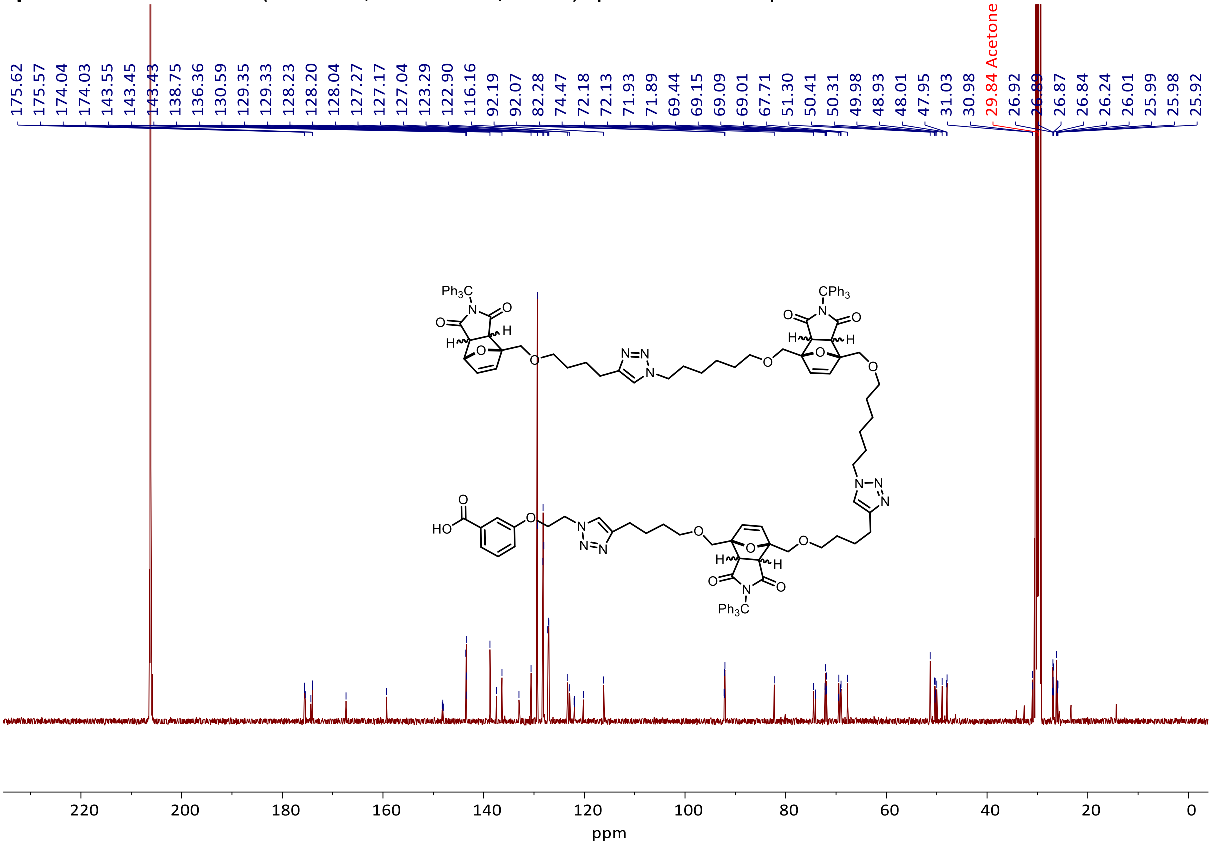

Spectrum S48. ^{13}C NMR (126 MHz, Acetone-d_6, 298 K) spectrum of compound **S18b**.

9.1.25 Spectra of S18c

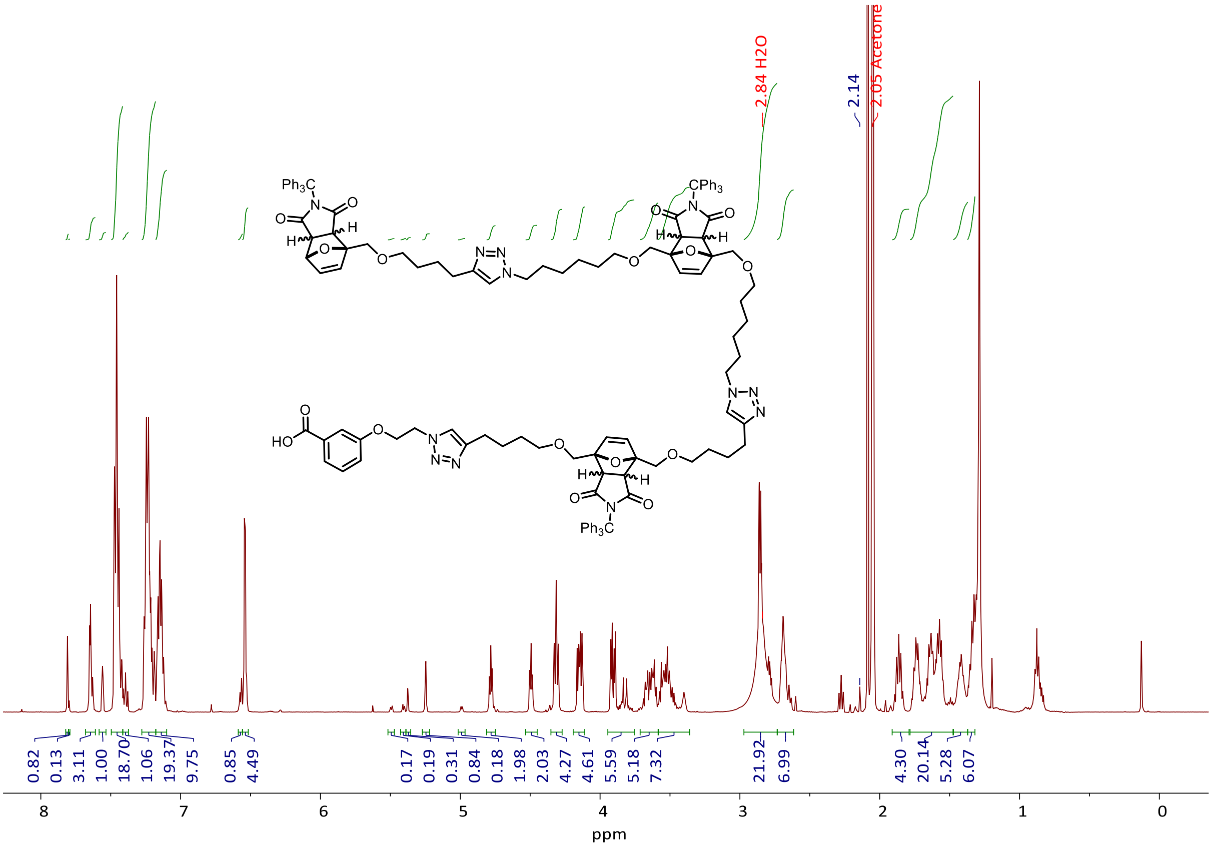

Spectrum S49. ^{1}H NMR (500 MHz, Acetone-d_6, 298 K) spectrum of compound **S18c**.

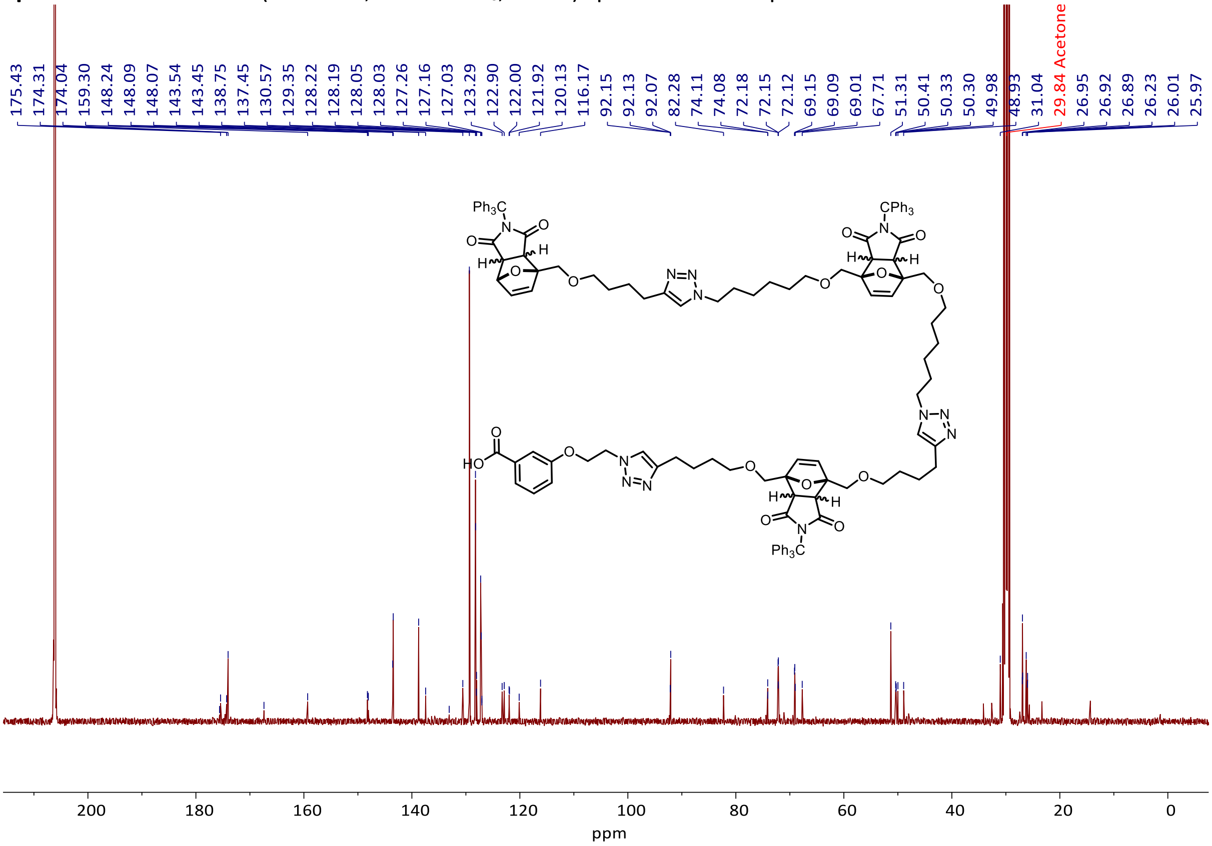

Spectrum S50. ^{13}C NMR (126 MHz, Acetone-d_6, 298 K) spectrum of compound **S18c**.

9.1.26 Spectra of S18d

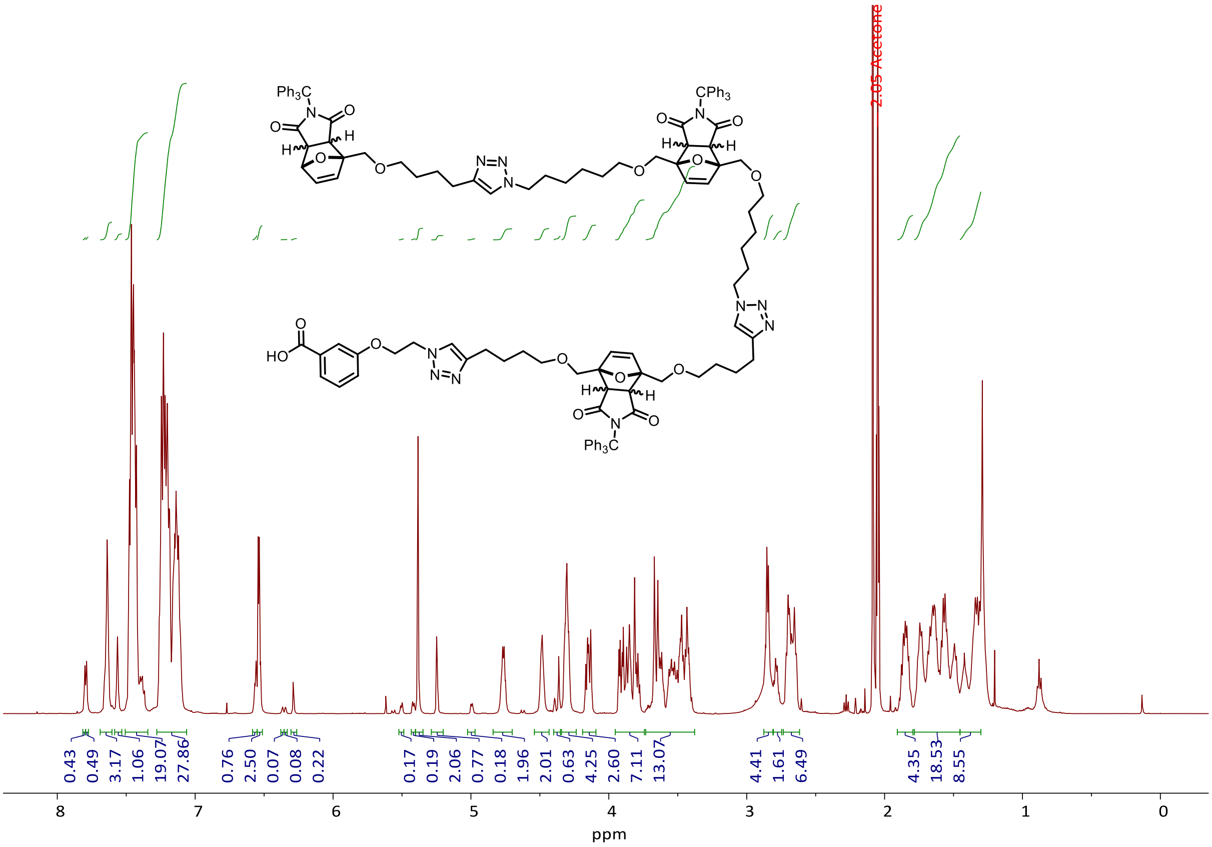

Spectrum S51. ^{1}H NMR (500 MHz, Acetone-d_6, 298 K) spectrum of compound **S18d**.

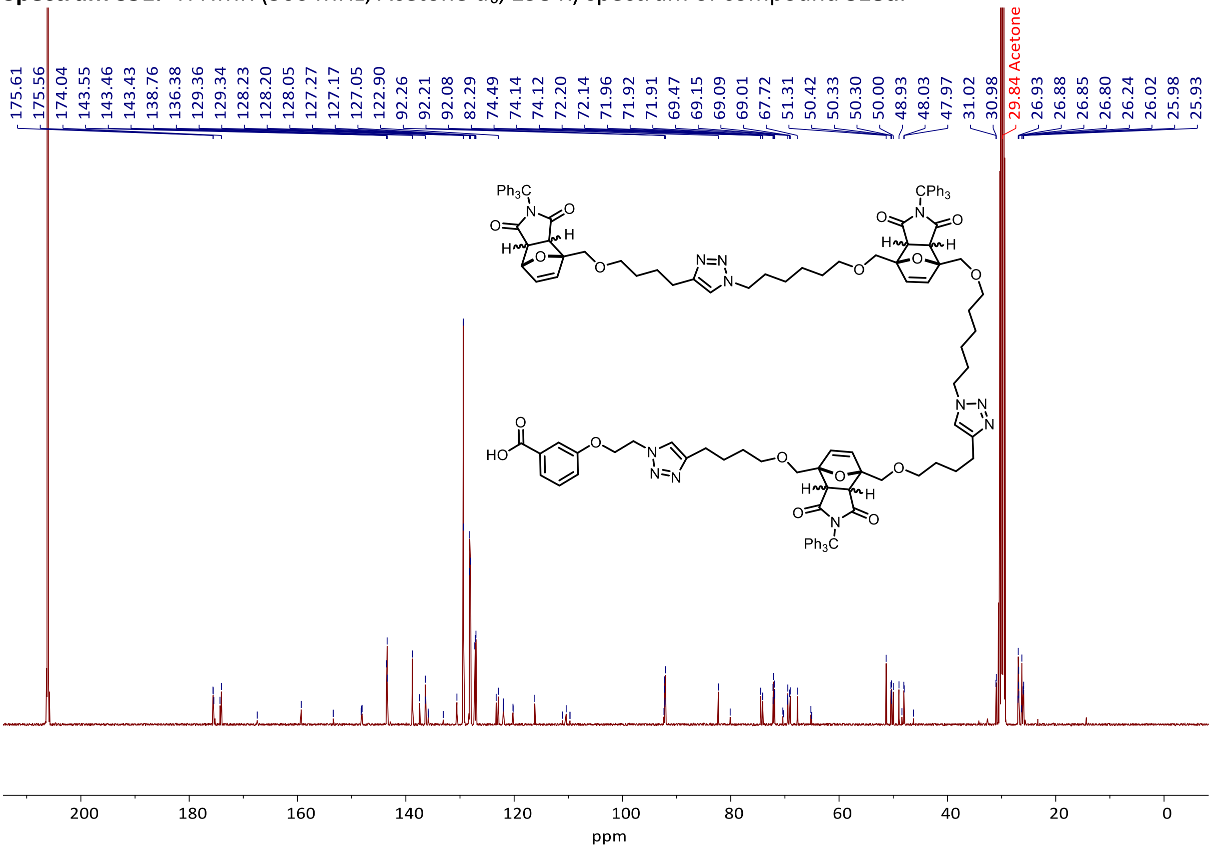

Spectrum S52. ^{13}C NMR (126 MHz, Acetone-d_6, 298 K) spectrum of compound **S18d**.

9.1.27 Spectra of S19

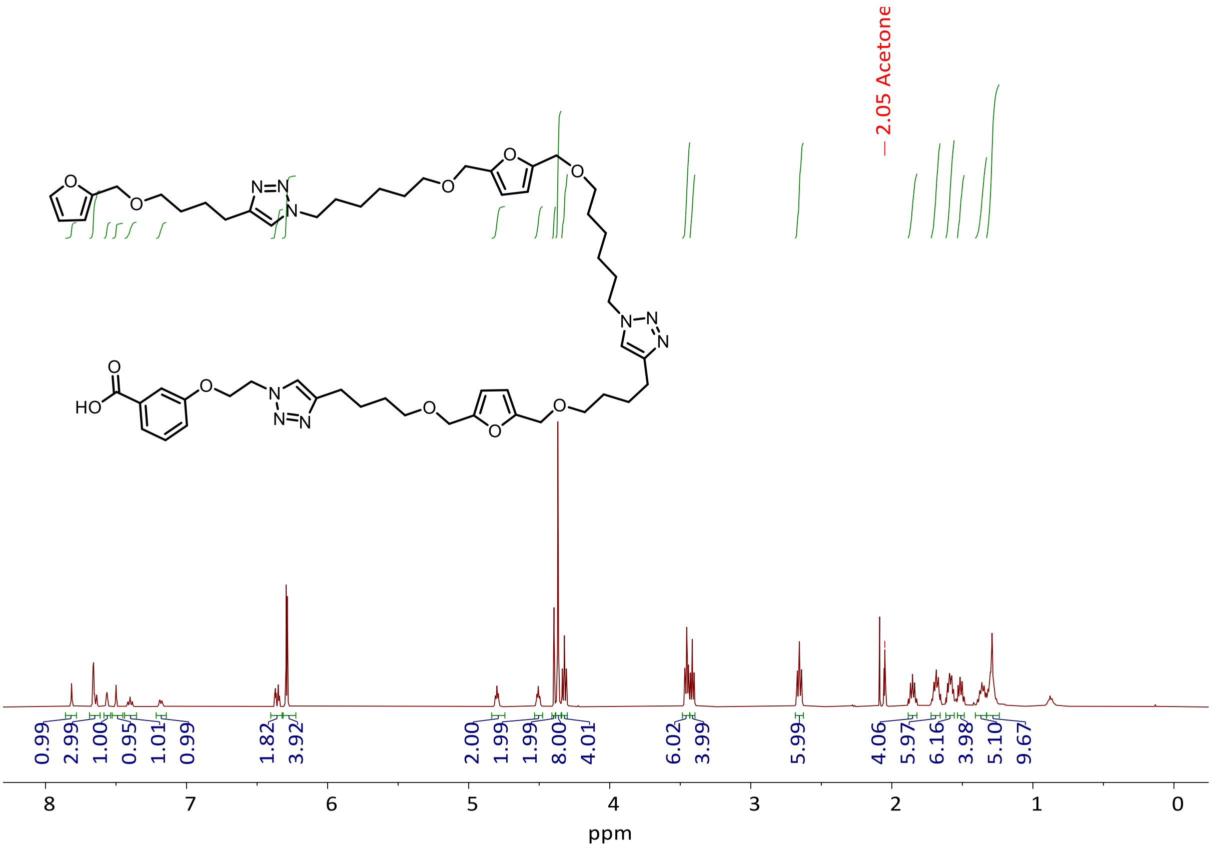

Spectrum S53. ^{1}H NMR (500 MHz, Acetone-d_6, 298 K) spectrum of compound **S19**.

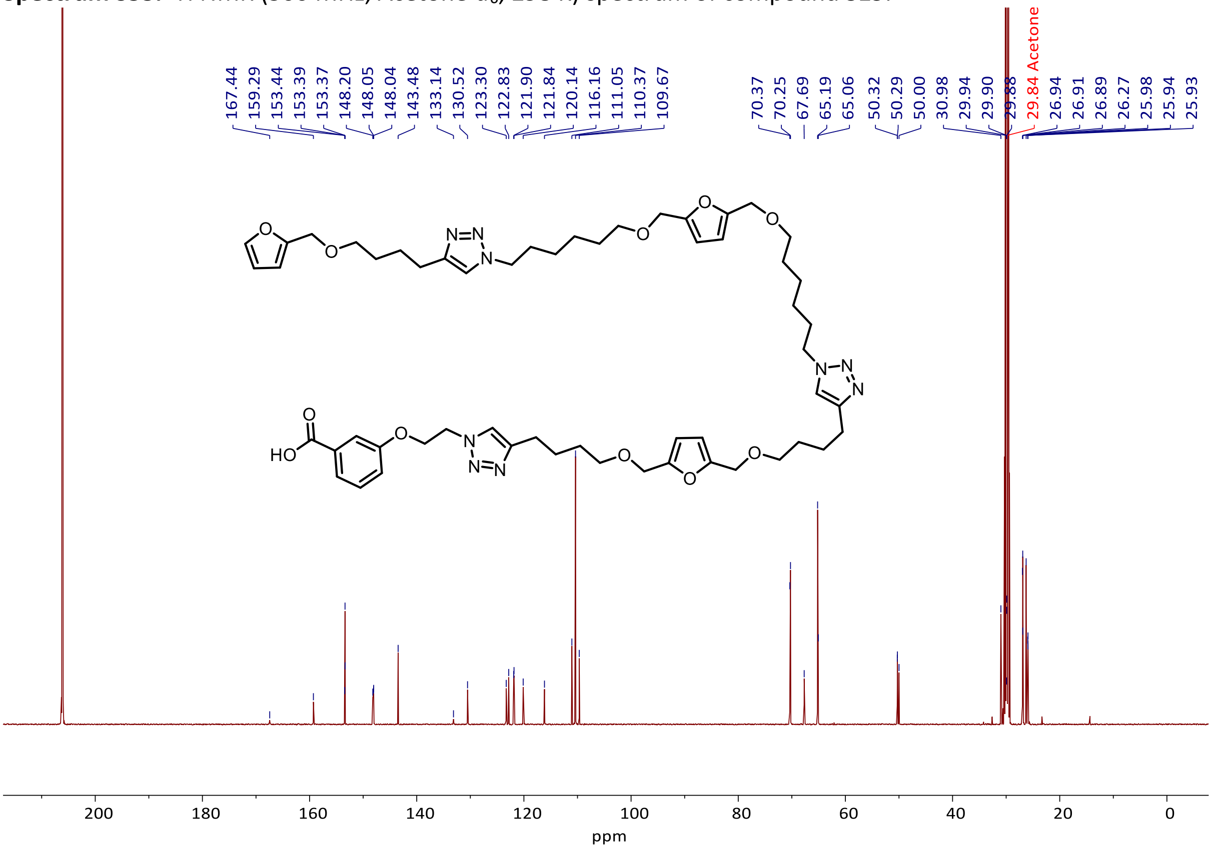

Spectrum S54. ^{13}C NMR (126 MHz, Acetone-d_6, 298 K) spectrum of compound **S19**.

9.1.28 Spectra of S20

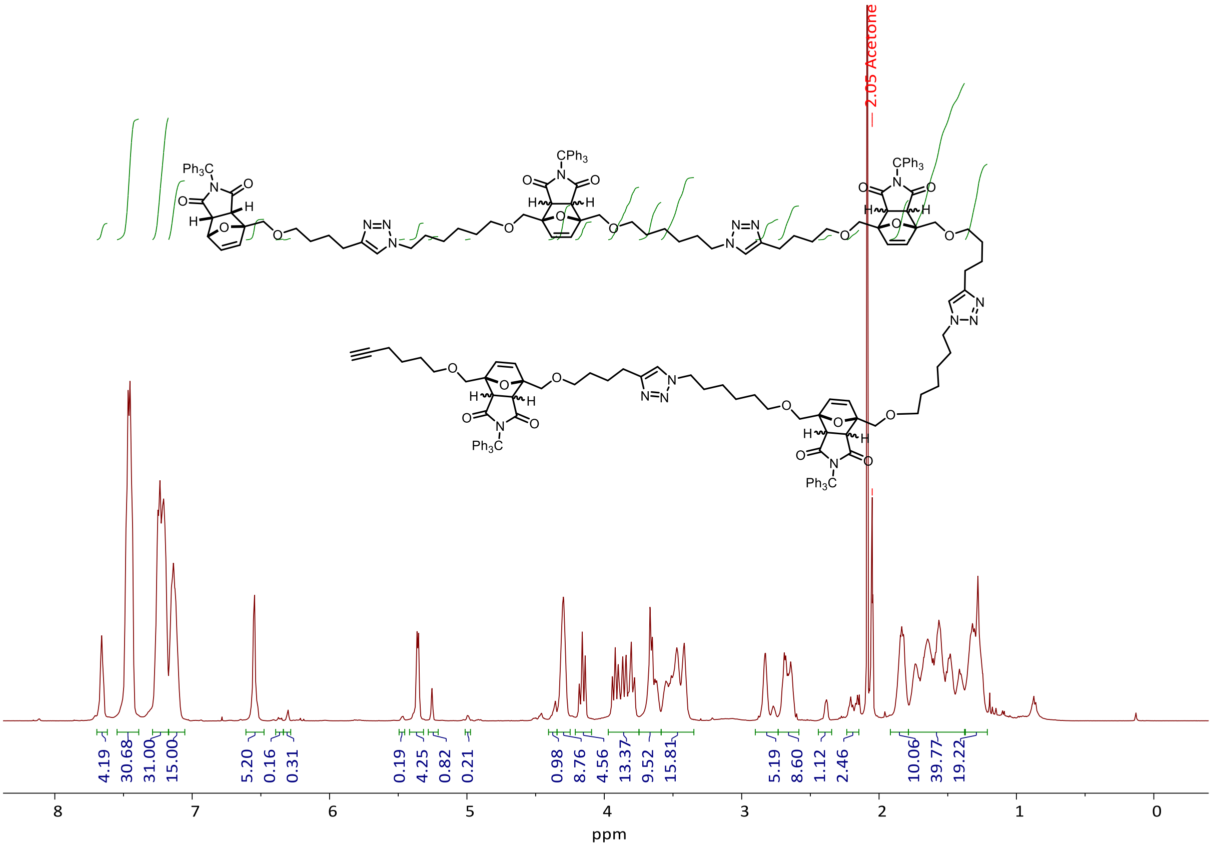

Spectrum S55. ^{1}H NMR (500 MHz, Acetone-d_6, 298 K) spectrum of compound **S20**.

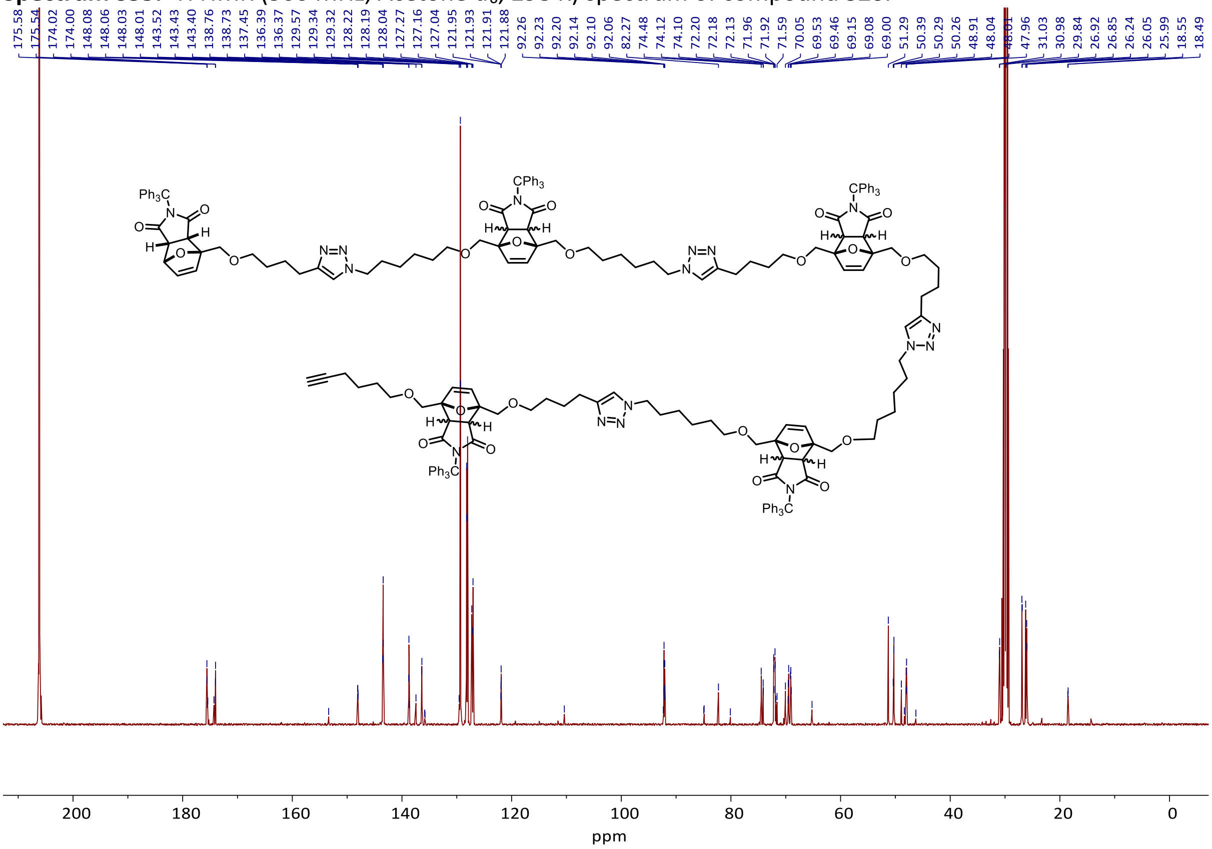

Spectrum S56. ^{13}C NMR (126 MHz, Acetone-d_6, 298 K) spectrum of compound **S20**.

9.1.29 Spectra of S21

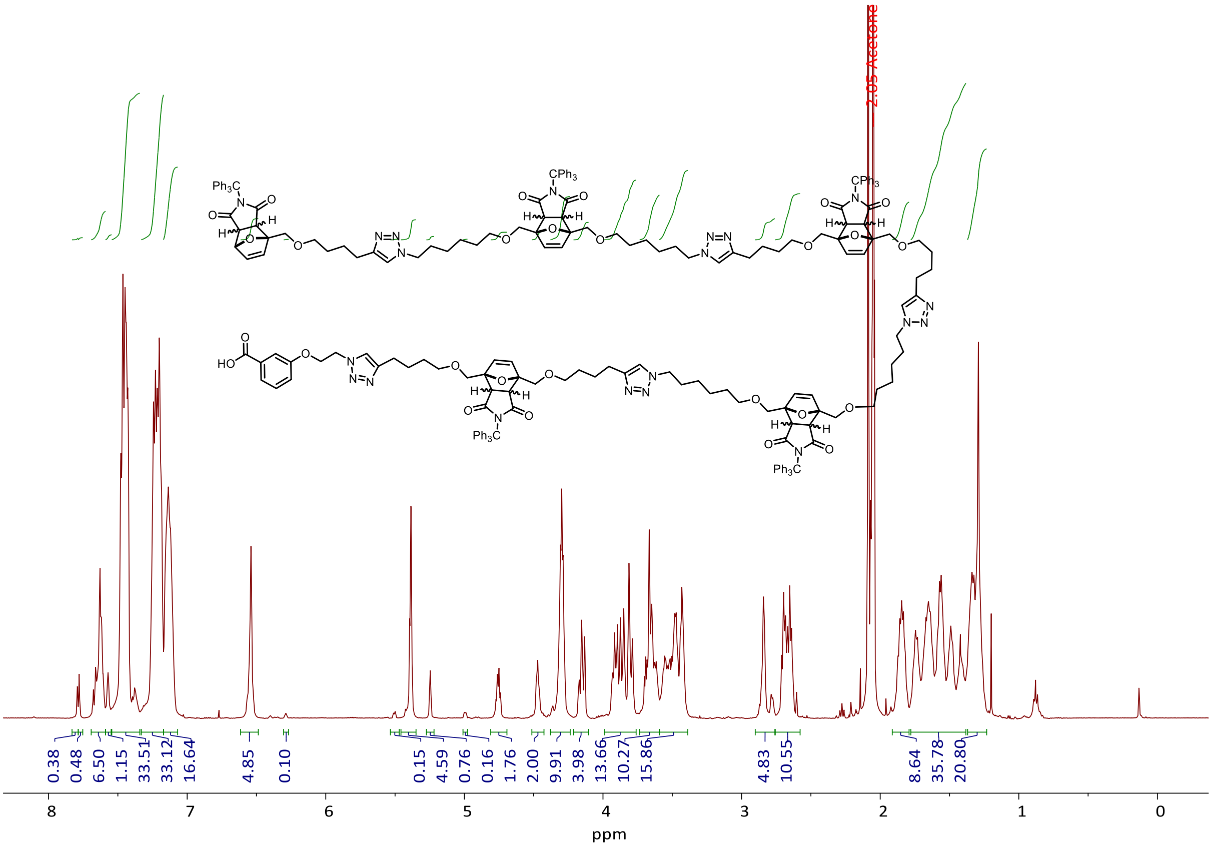

Spectrum S57. ^{1}H NMR (500 MHz, Acetone-d_6, 298 K) spectrum of compound **S21**.

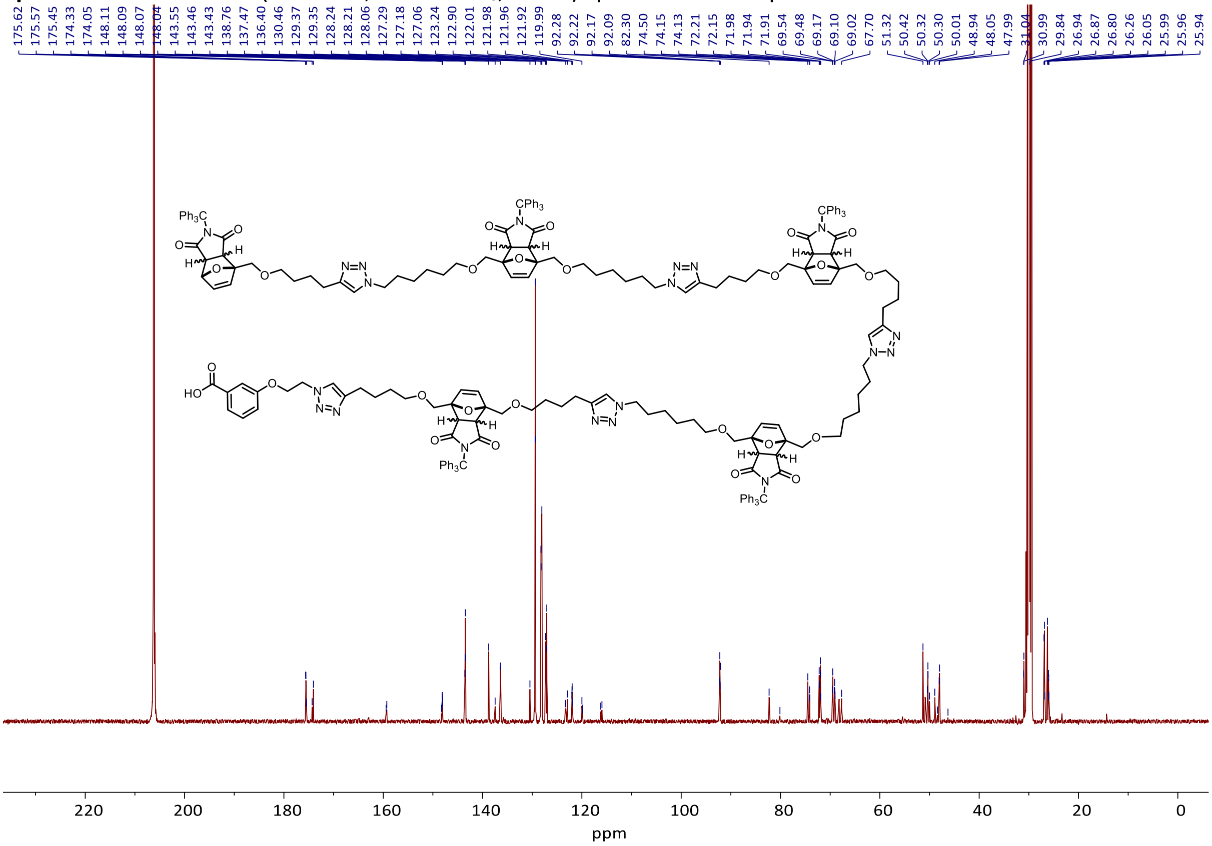

Spectrum S58. ^{13}C NMR (126 MHz, Acetone-d_6, 298 K) spectrum of compound **S21**.

9.1.30 Spectra of S22

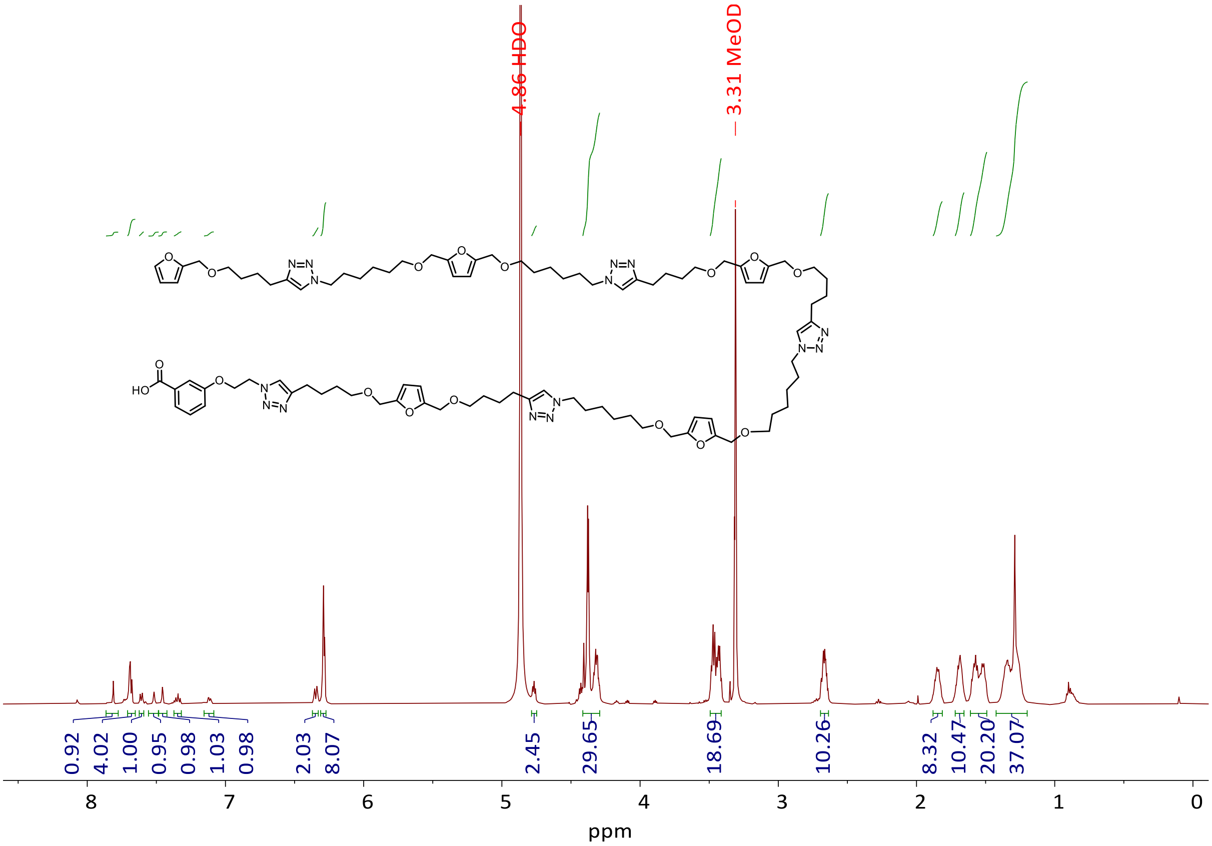

Spectrum S59. ^{1}H NMR (500 MHz, Methanol-d_4, 298 K) spectrum of compound **S22**.

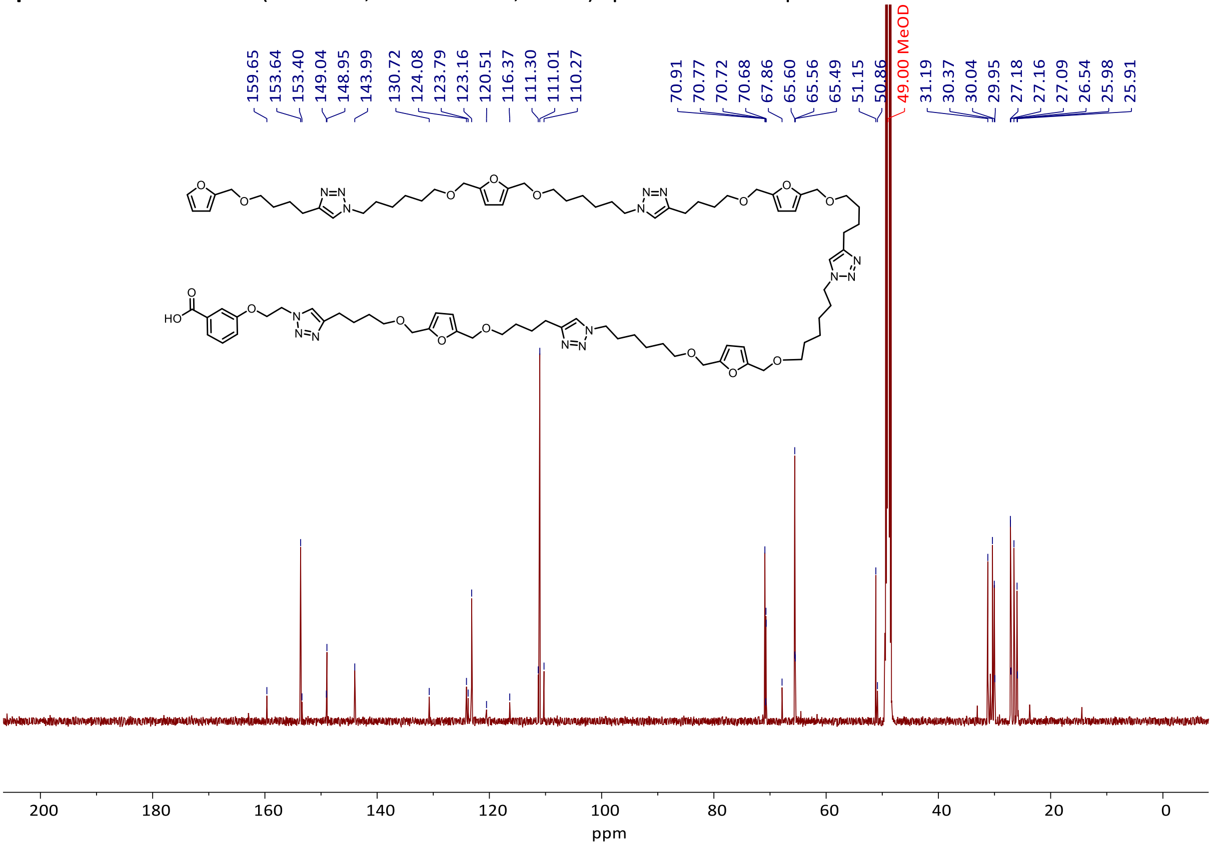

Spectrum S60. ^{13}C NMR (126 MHz, Methanol-d_4, 298 K) spectrum of compound **S22**.

9.1.31 Spectra of 5

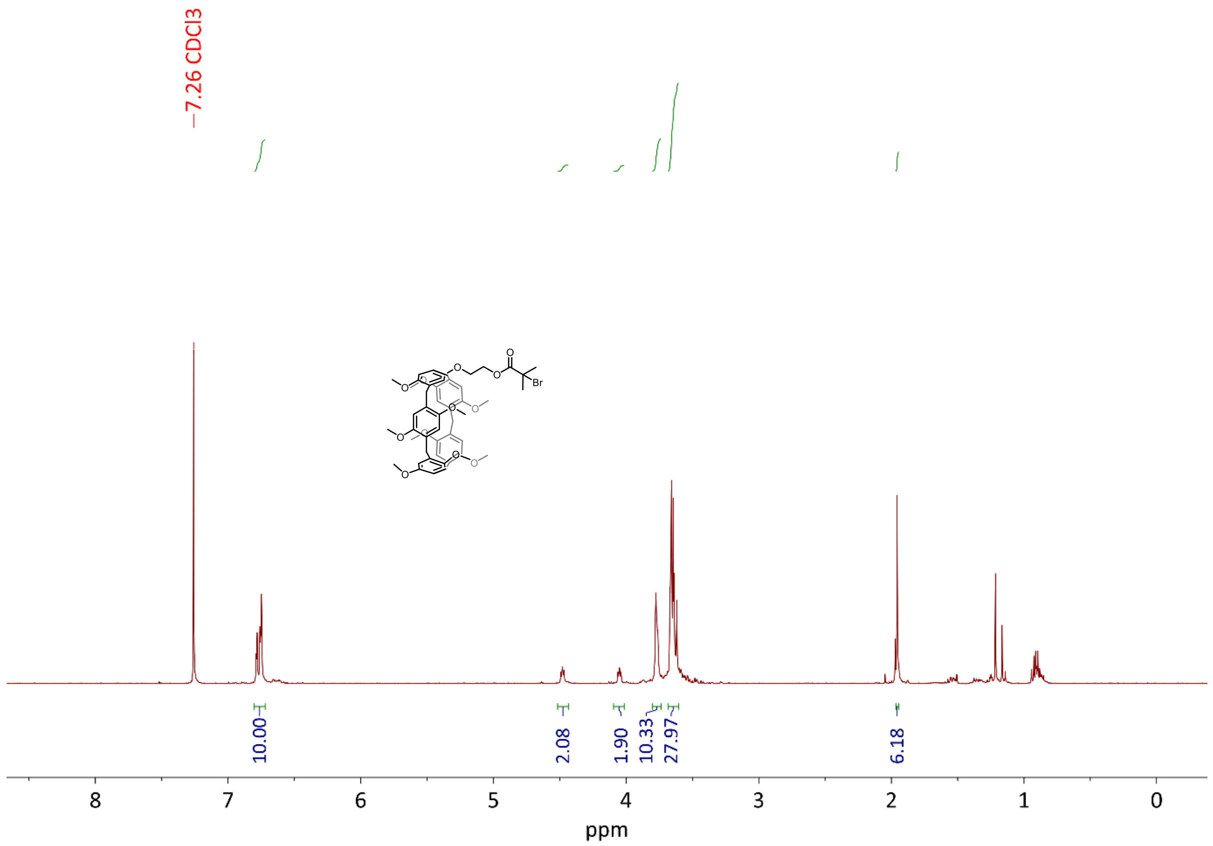

Spectrum S61. ^{1}H NMR (500 MHz, $CDCl_3$, 298 K) spectrum of compound **5**.

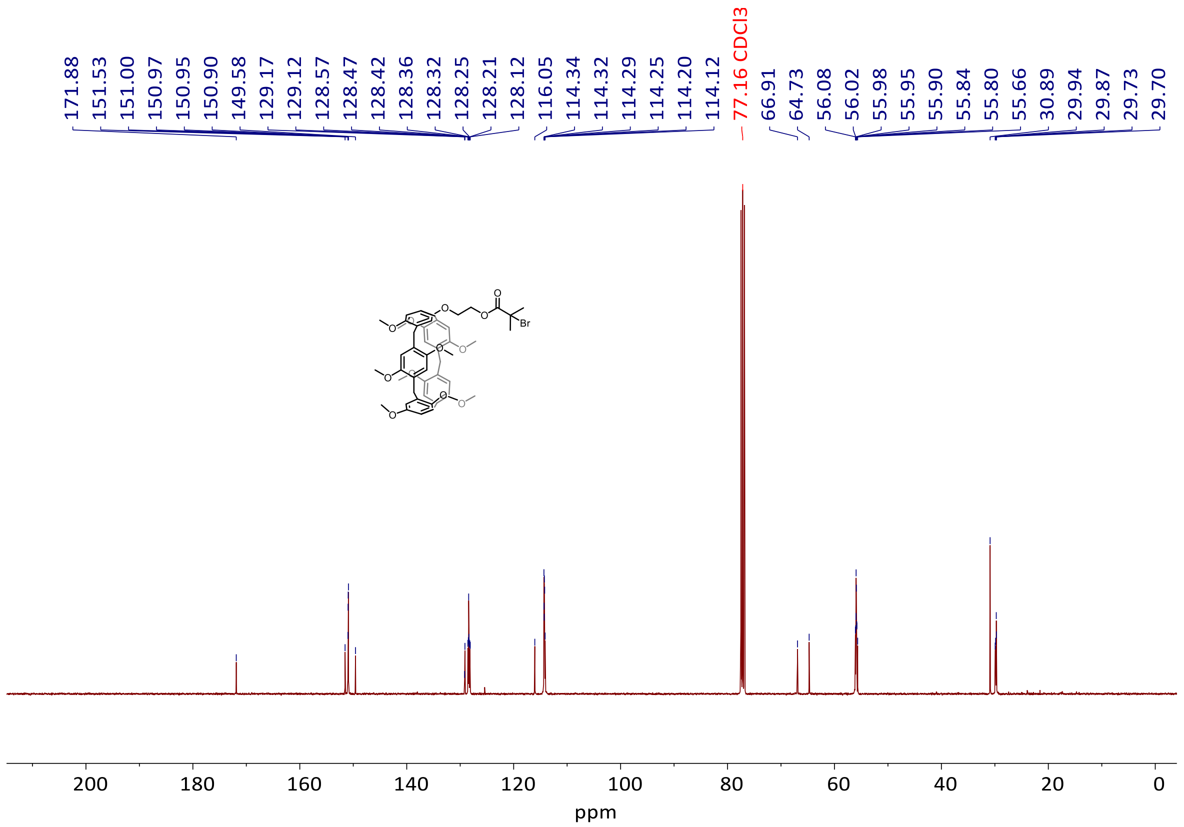

Spectrum S62. ^{13}C NMR (126 MHz, $CDCl_3$, 298 K) spectrum of compound **5**.

9.1.32 Spectra of 6

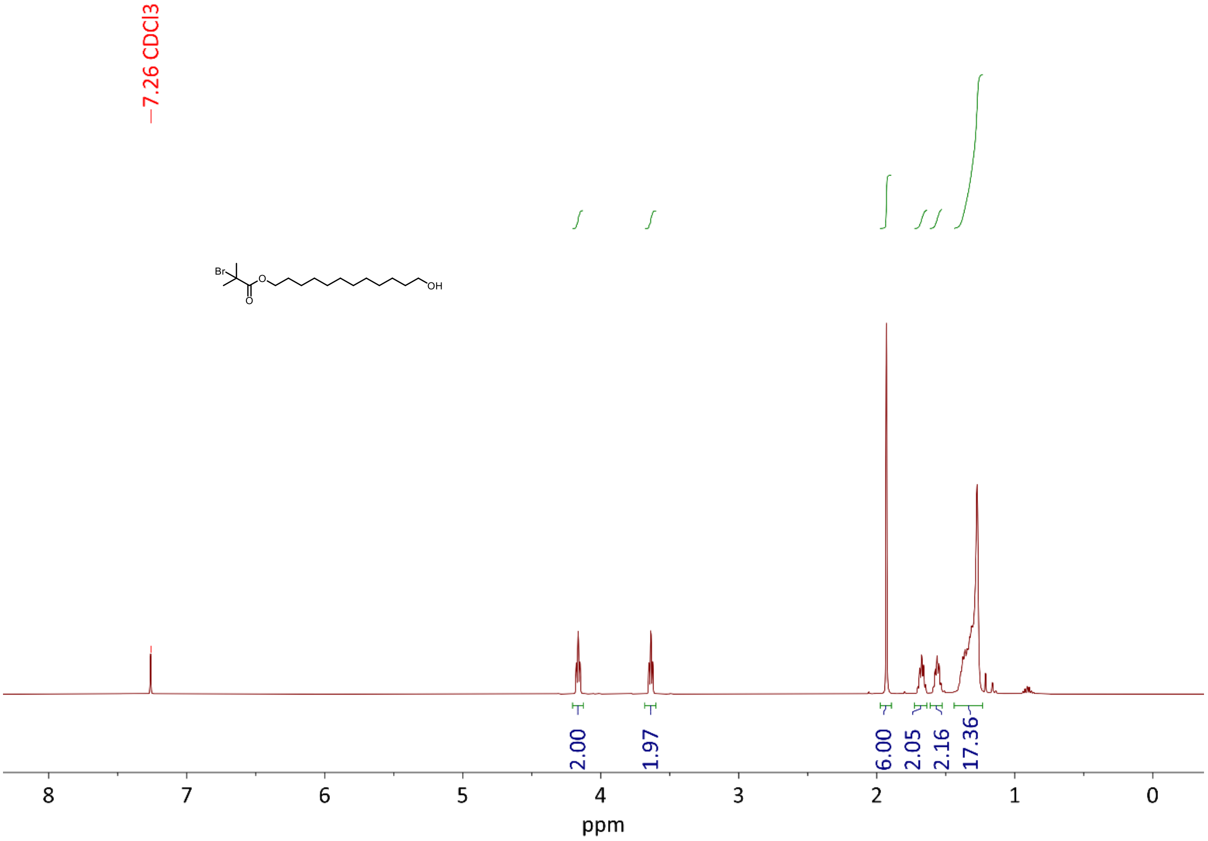

Spectrum S63. ^{1}H NMR (500 MHz, $CDCl_3$, 298 K) spectrum of compound **6**.

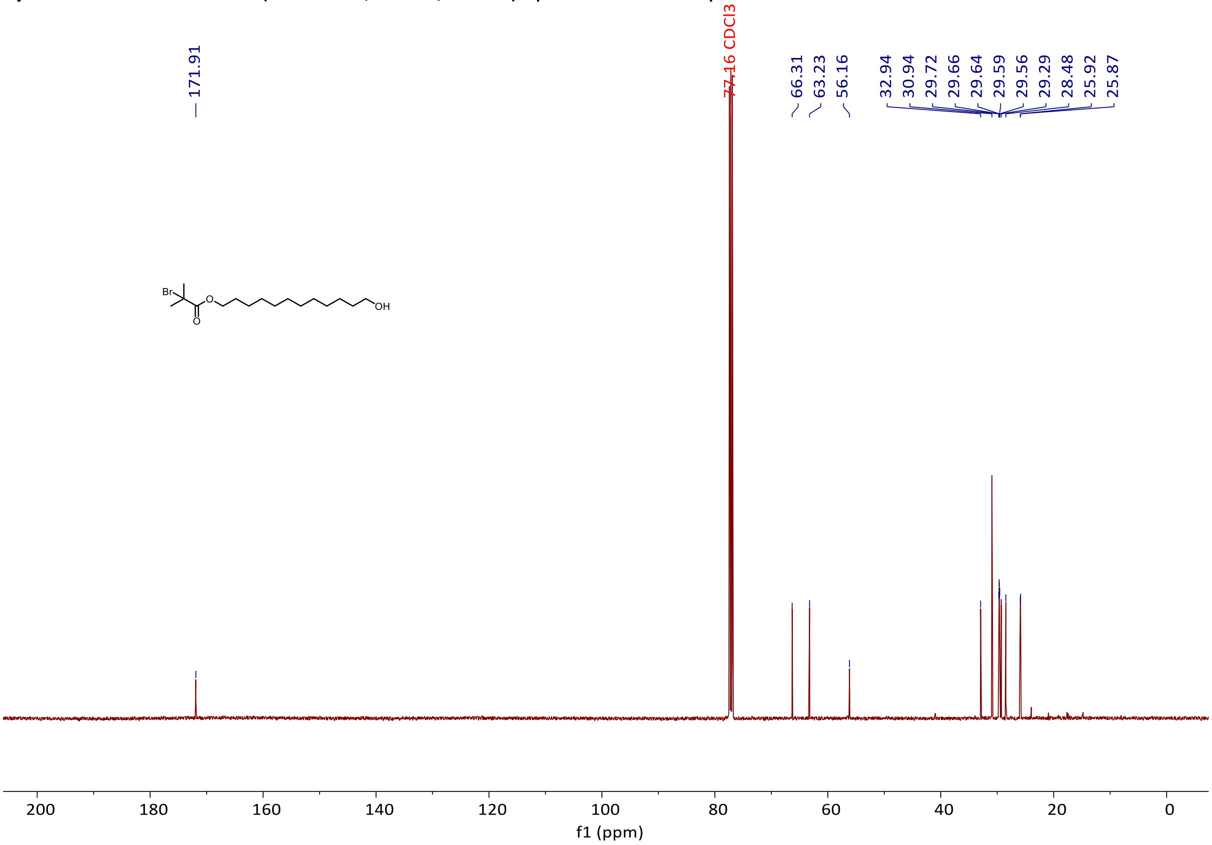

Spectrum S64. ^{13}C NMR (126 MHz, $CDCl_3$, 298 K) spectrum of compound **6**.

9.1.33 Spectra of **7**$_{trans}$

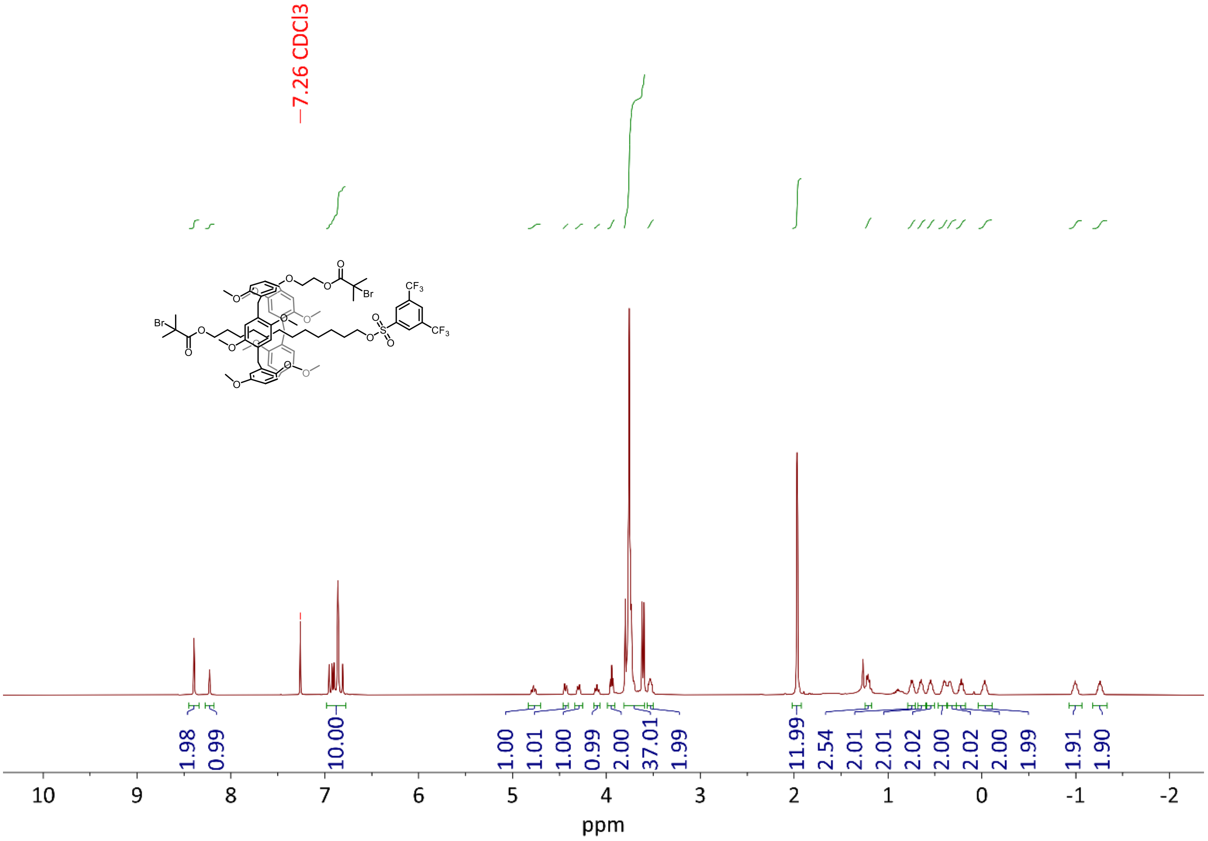

Spectrum S65. ^{1}H NMR (500 MHz, $CDCl_3$, 298 K) spectrum of compound **7**$_{trans}$.

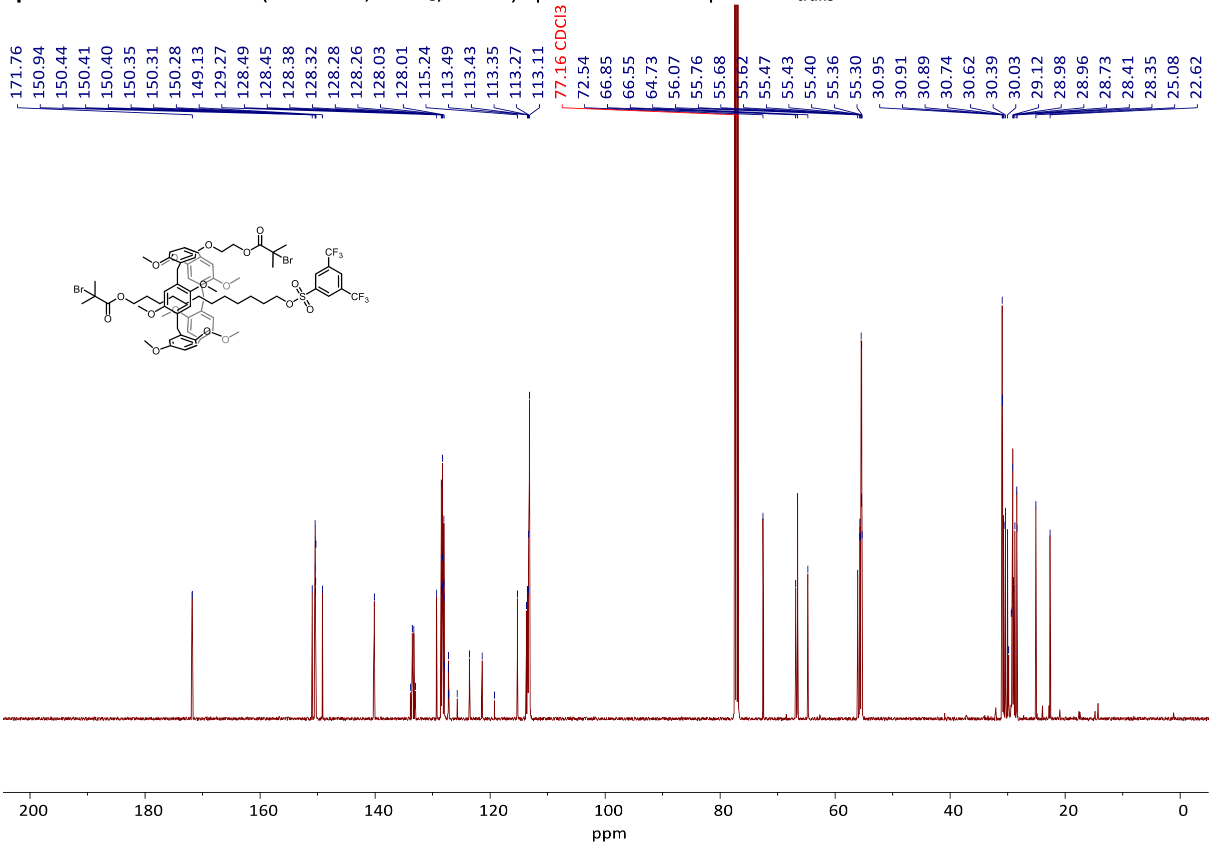

Spectrum S66. ^{13}C NMR (126 MHz, $CDCl_3$, 298 K) spectrum of compound **7**$_{trans}$.

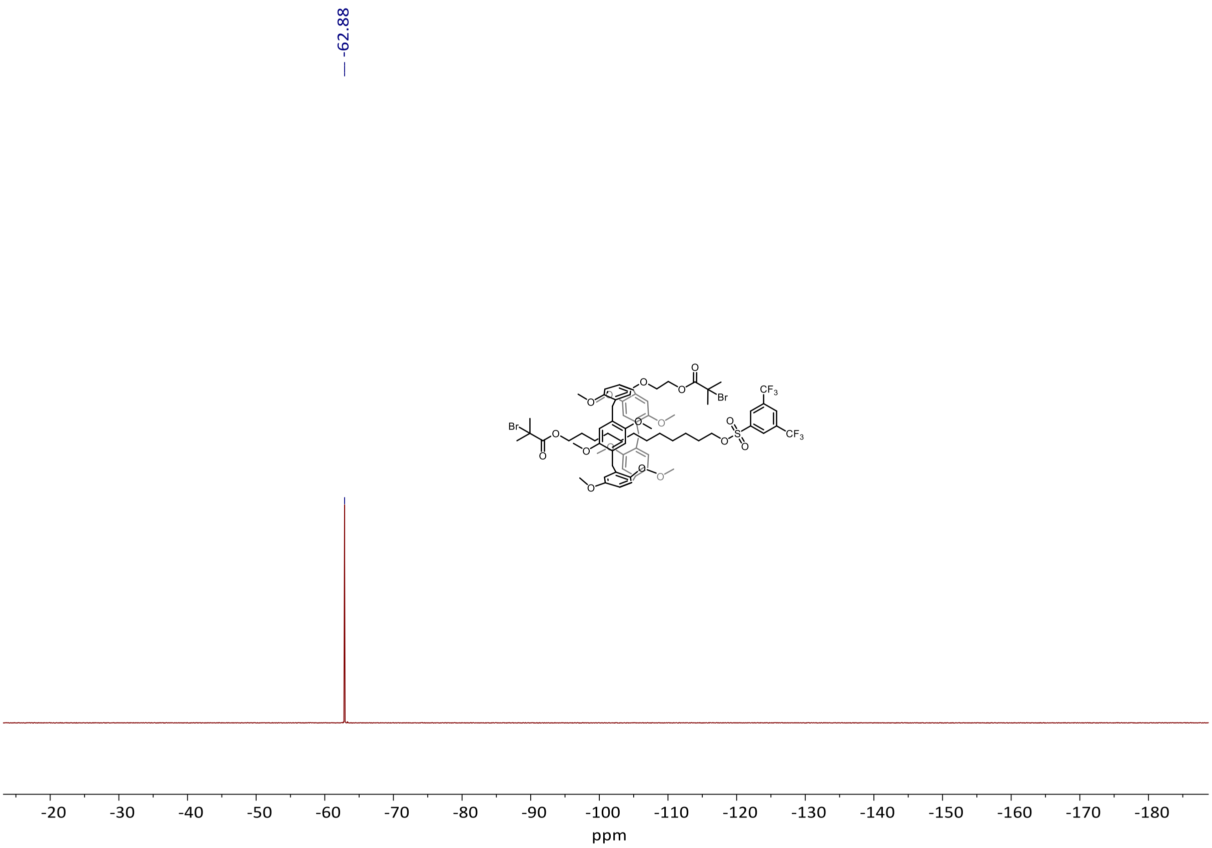

Spectrum S67. ^{19}F NMR (471 MHz, $CDCl_3$, 298 K) spectrum of compound **7_{trans}**.

9.1.34 Spectra of $\mathbf{7}_{cis}$

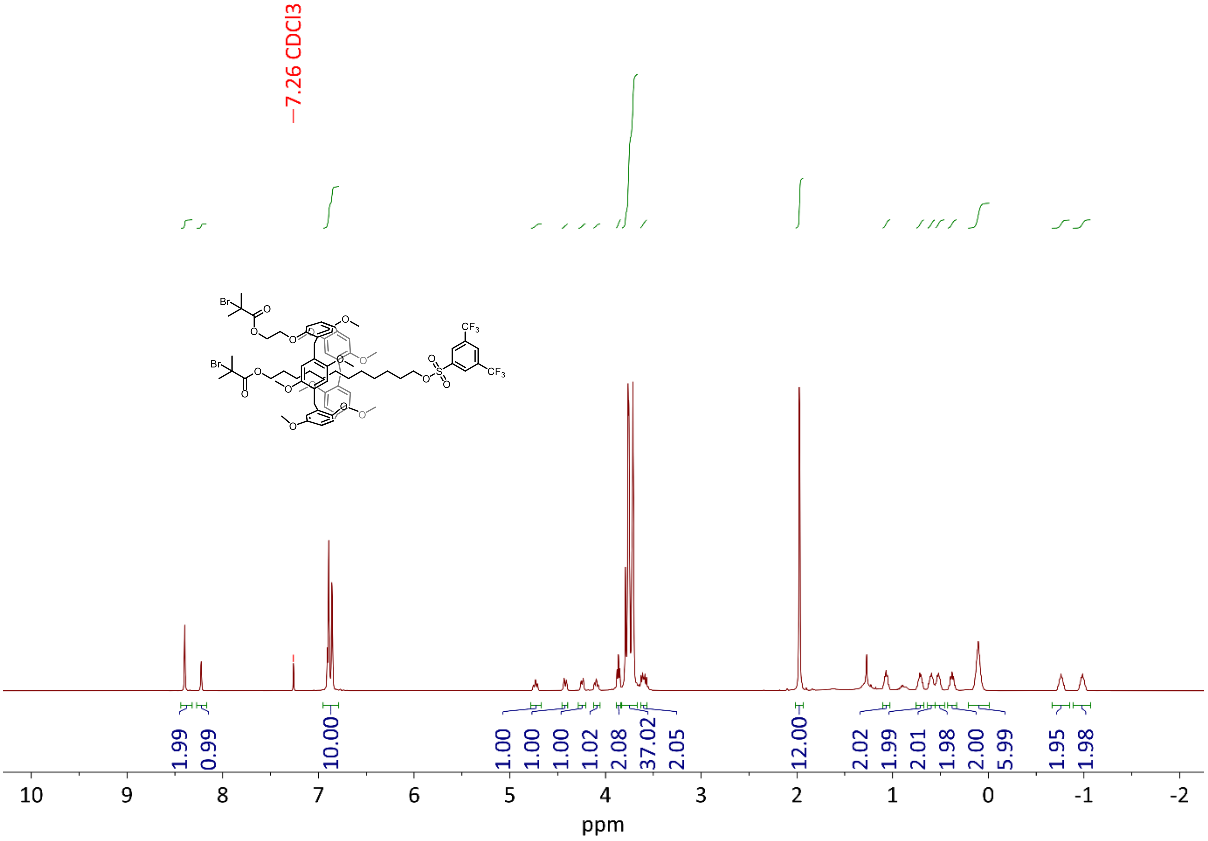

Spectrum S68. ^{1}H NMR (500 MHz, $CDCl_3$, 298 K) spectrum of compound $\mathbf{7}_{cis}$.

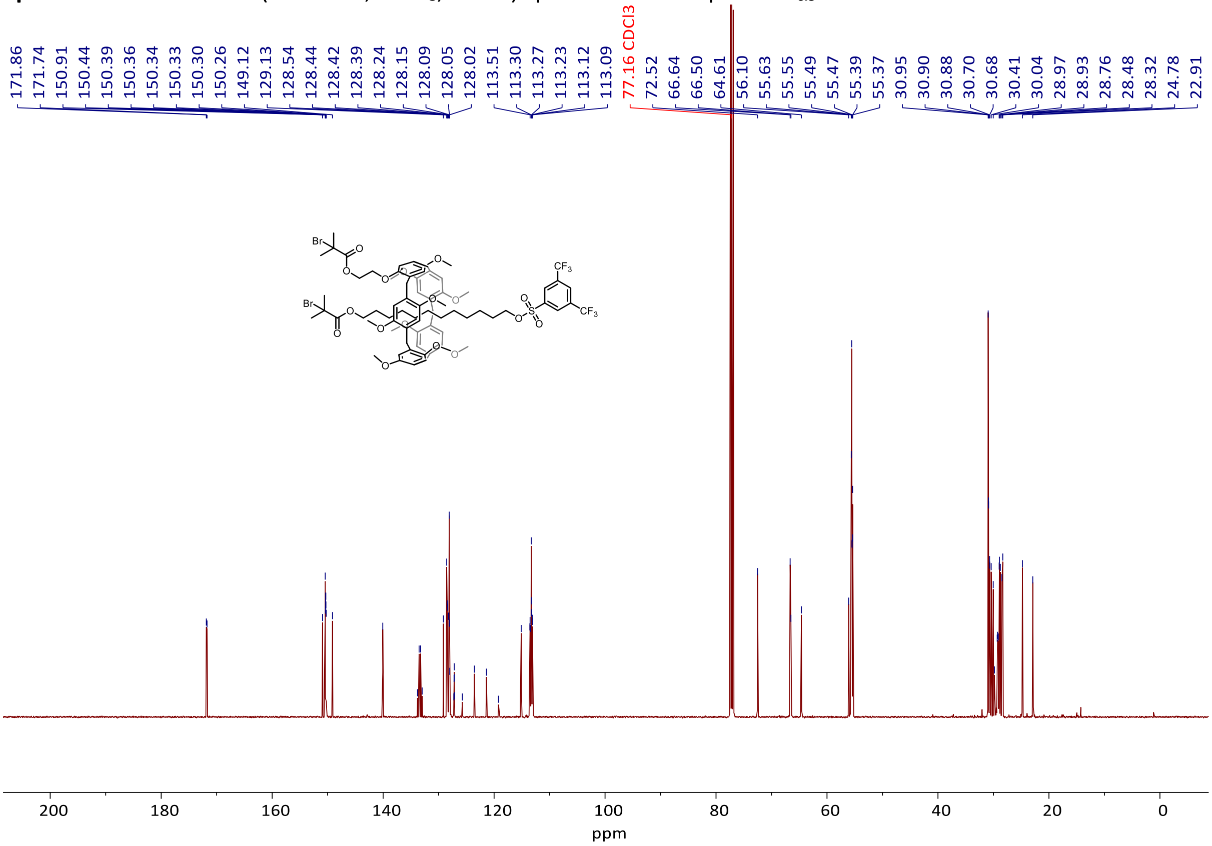

Spectrum S69. ^{13}C NMR (126 MHz, $CDCl_3$, 298 K) spectrum of compound $\mathbf{7}_{cis}$.

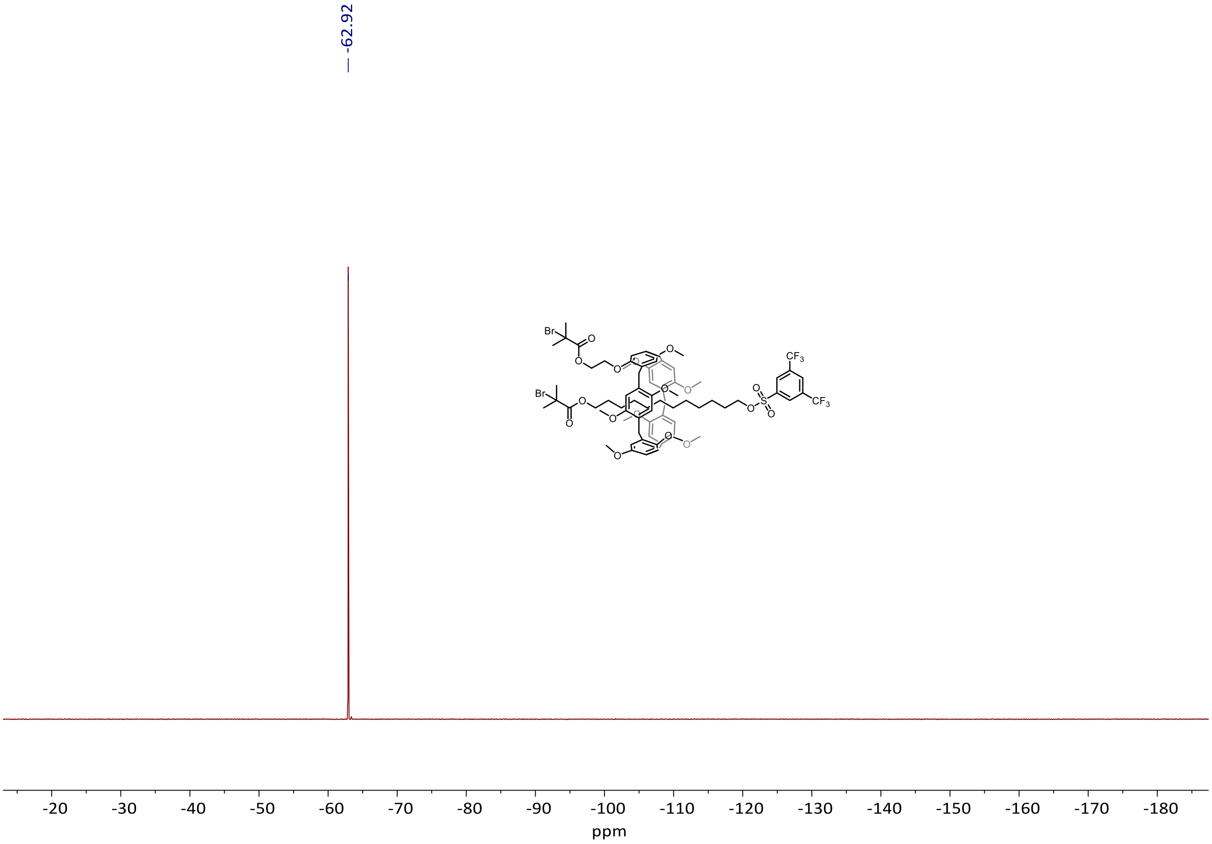

Spectrum S70. ^{19}F NMR (471 MHz, $CDCl_3$, 298 K) spectrum of compound $\mathbf{7}_{\boldsymbol{cis}}$.

9.1.35 Spectra of S24

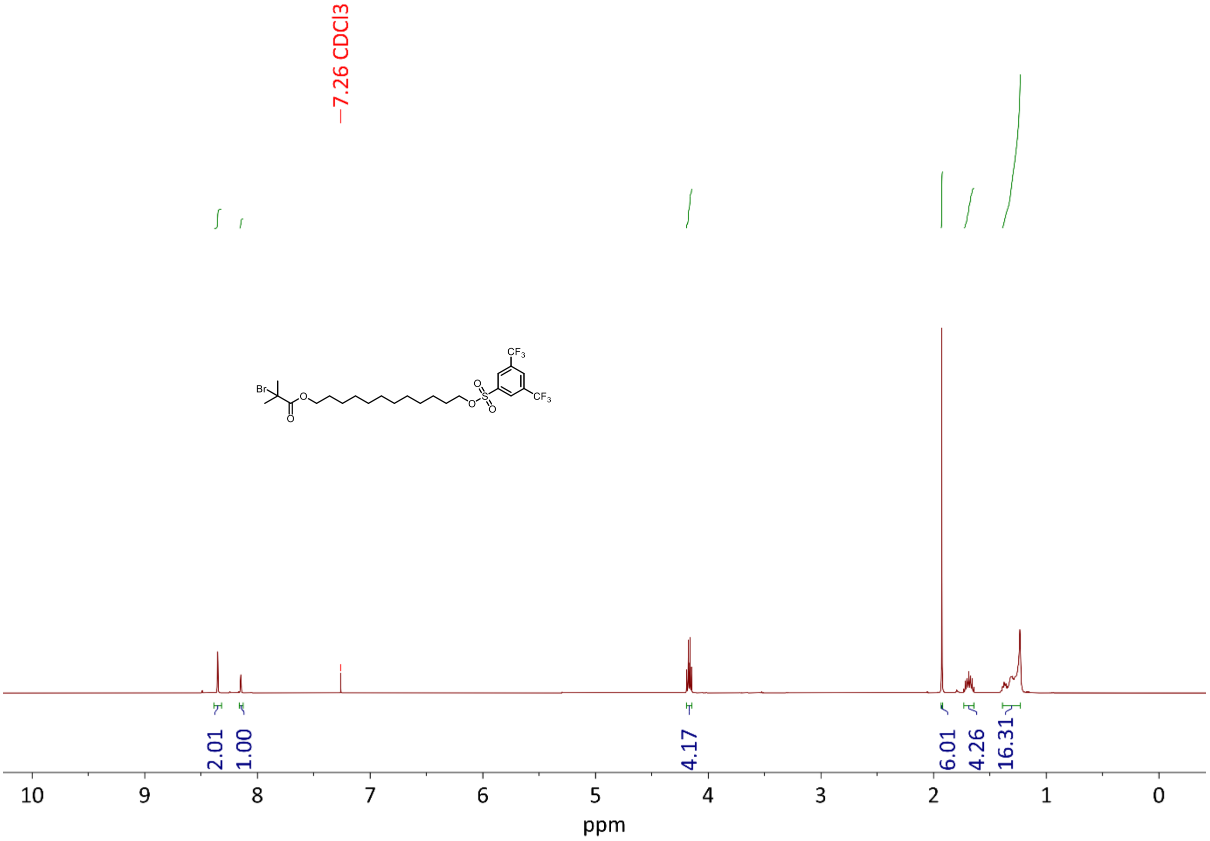

Spectrum S71. ^{1}H NMR (500 MHz, $CDCl_3$, 298 K) spectrum of compound **S24**.

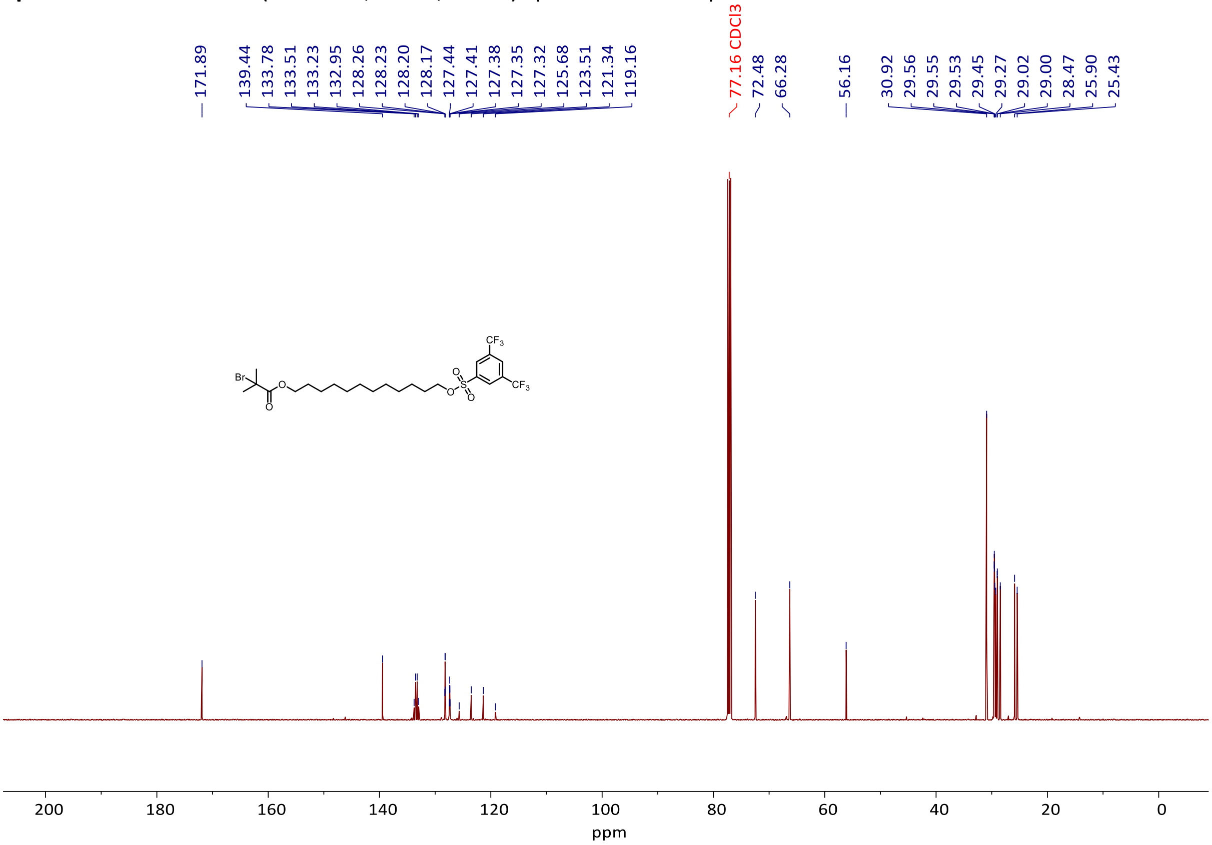

Spectrum S72. ^{13}C NMR (126 MHz, $CDCl_3$, 298 K) spectrum of compound **S24**.

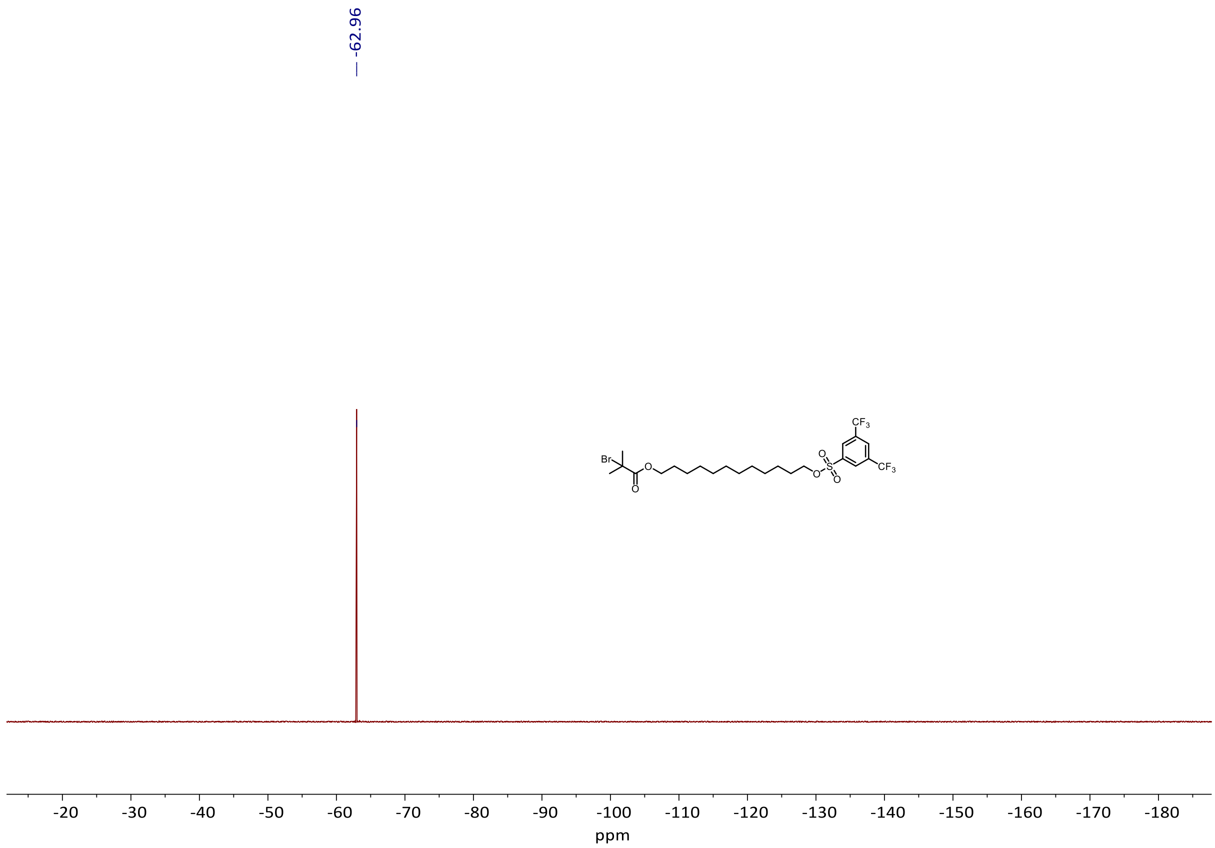

Spectrum S73. ^{19}F NMR (471 MHz, $CDCl_3$, 298 K) spectrum of compound **S24**.

9.1.36 Spectra of S25*trans*

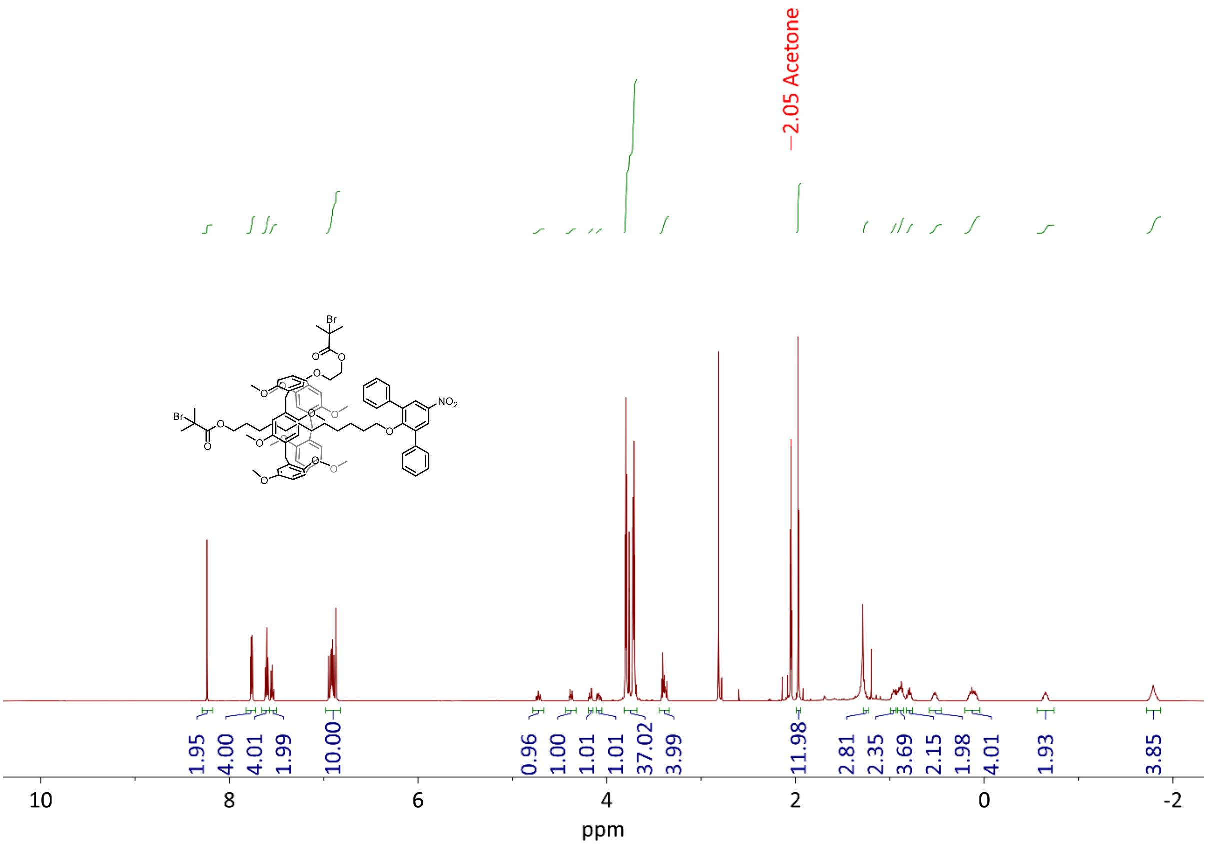

Spectrum S74. ^{1}H NMR (500 MHz, Acetone-d_6, 298 K) spectrum of compound **S25*trans***.

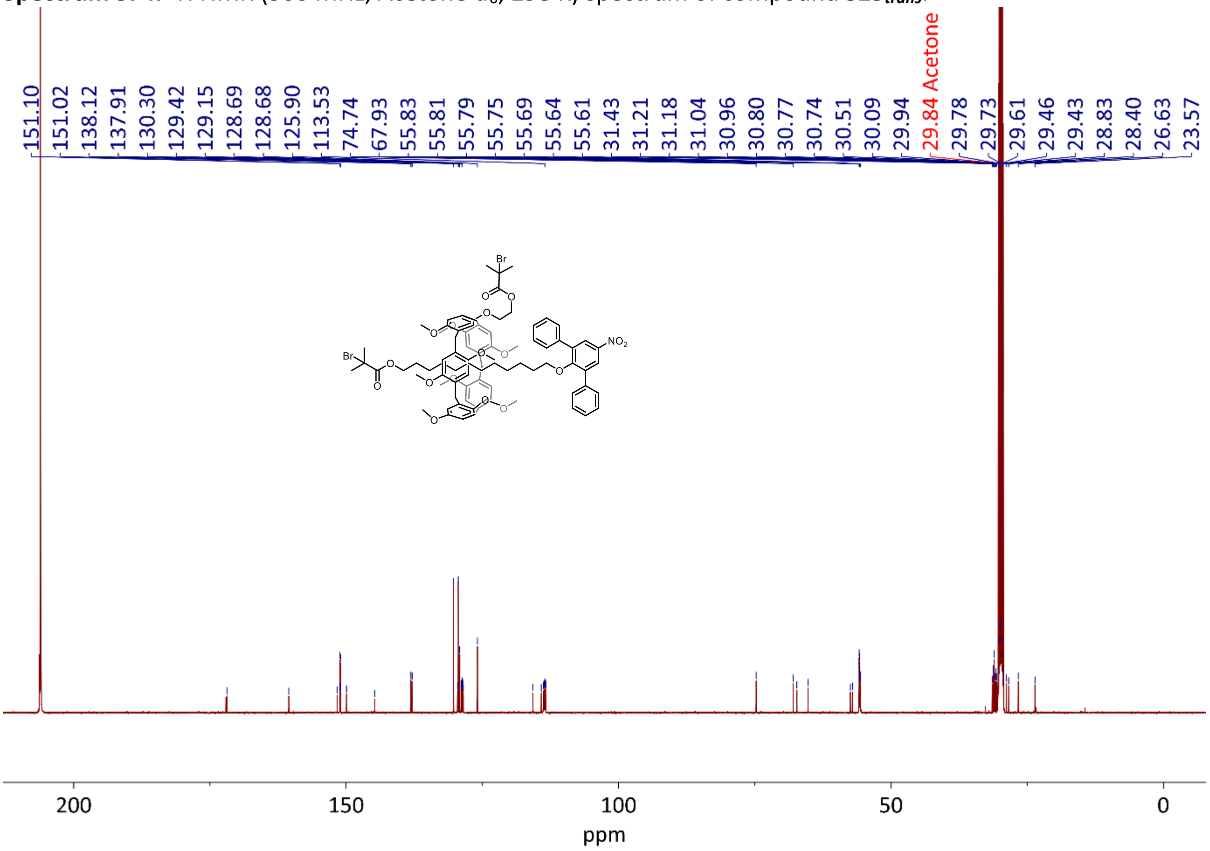

Spectrum S75. ^{13}C NMR (126 MHz, Acetone-d_6, 298 K) spectrum of compound **S25*trans***.

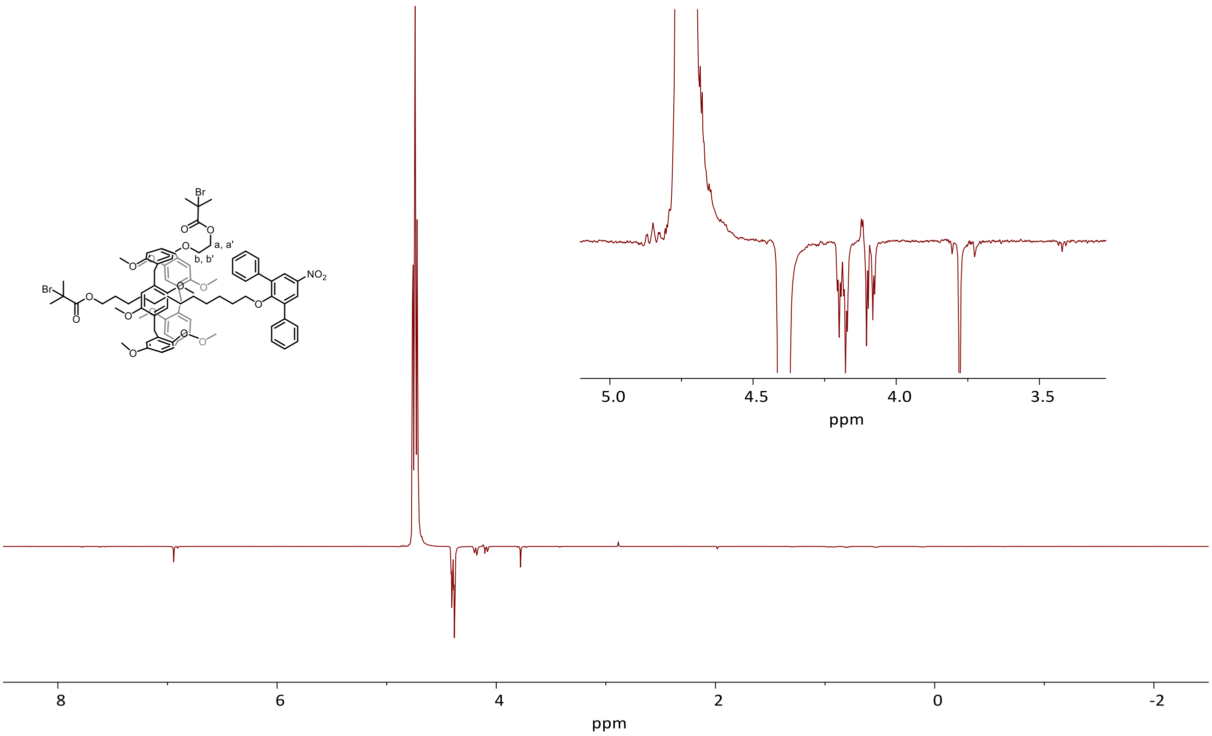

Spectrum S76. 1D selective ROESY ^{1}H NMR (500 MHz, Acetone-d_6, 298 K) spectrum of compound **S25*trans*** with selectively inverted proton H_a.

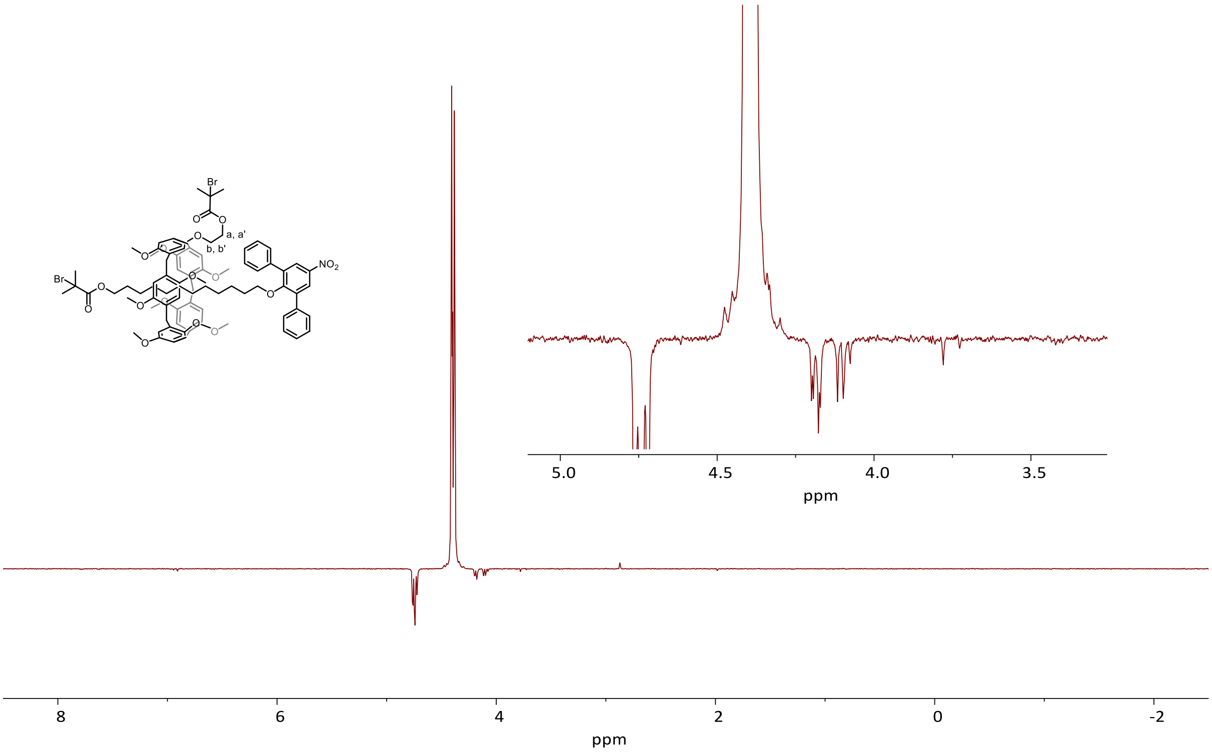

Spectrum S77. 1D selective ROESY ^{1}H NMR (500 MHz, Acetone-d_6, 298 K) spectrum of compound **S25*trans*** with selectively inverted proton $H_{a'}$.

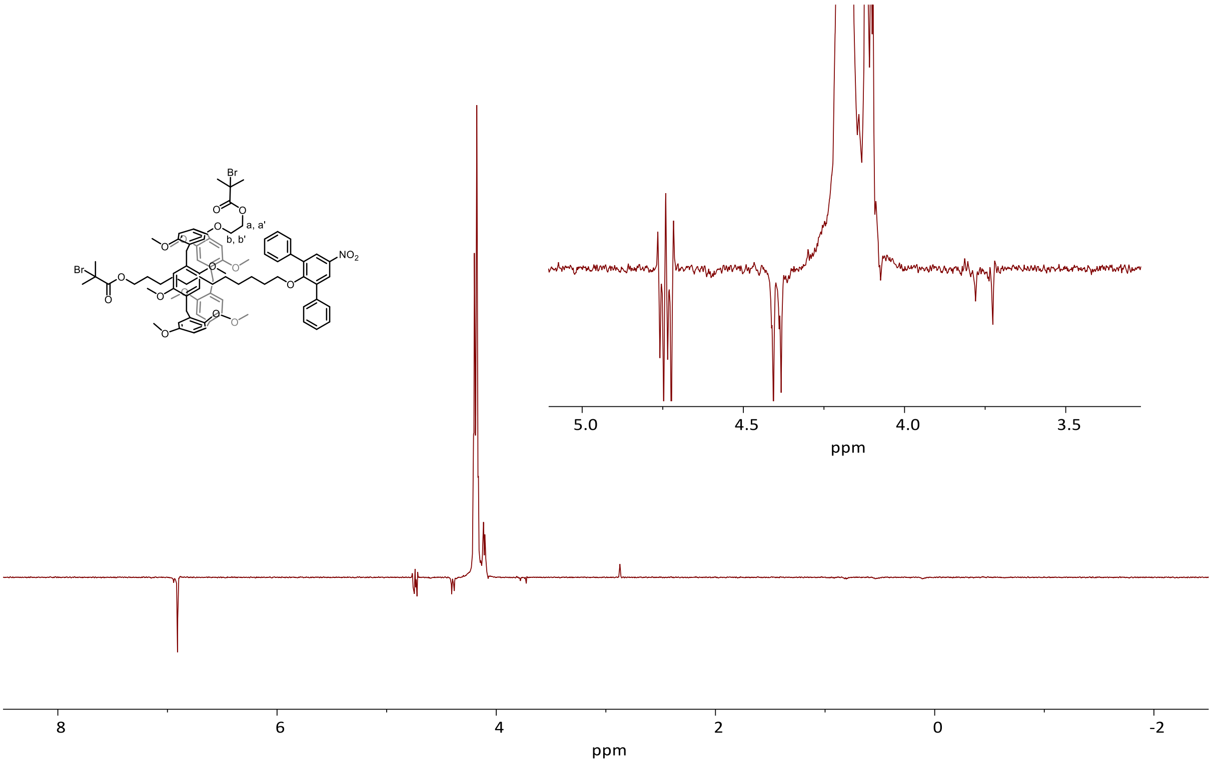

Spectrum S78. 1D selective ROESY ^{1}H NMR (500 MHz, Acetone-d_6, 298 K) spectrum of compound **S25**$_{\boldsymbol{trans}}$ with selectively inverted proton H_b.

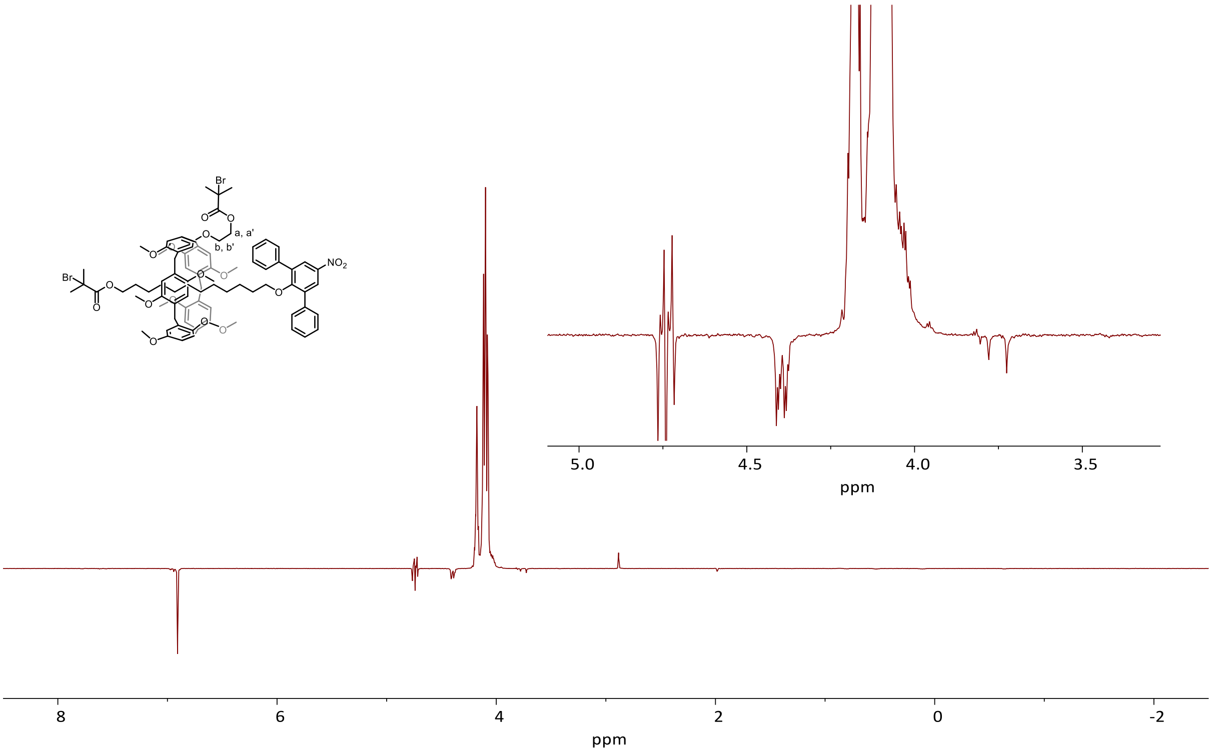

Spectrum S79. 1D selective ROESY ^{1}H NMR (500 MHz, Acetone-d_6, 298 K) spectrum of compound **S25**$_{\boldsymbol{trans}}$ with selectively inverted proton $H_{b'}$.

9.1.37 Spectra of S25$_{cis}$

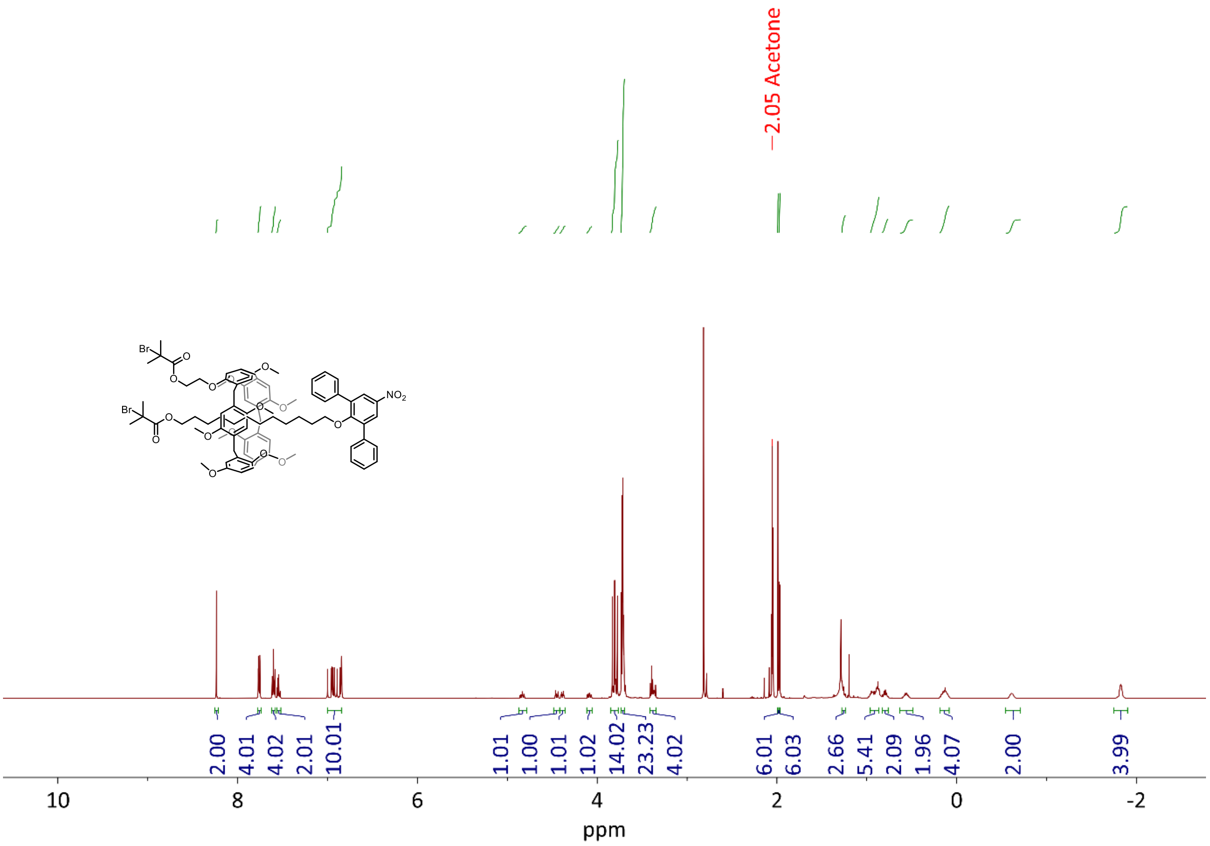

Spectrum S80. ^{1}H NMR (500 MHz, Acetone-d_6, 298 K) spectrum of compound **S25$_{cis}$**.

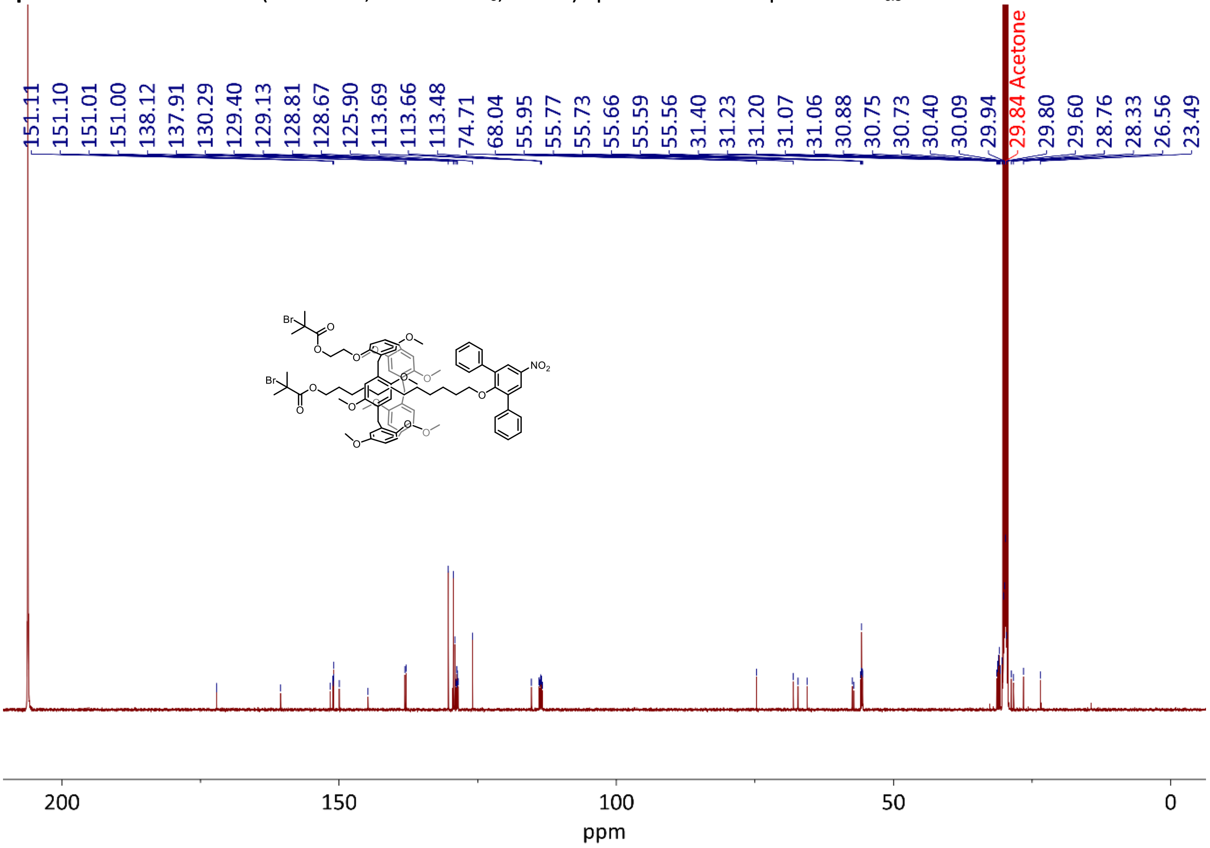

Spectrum S81. ^{13}C NMR (126 MHz, Acetone-d_6, 298 K) spectrum of compound **S25$_{cis}$**.

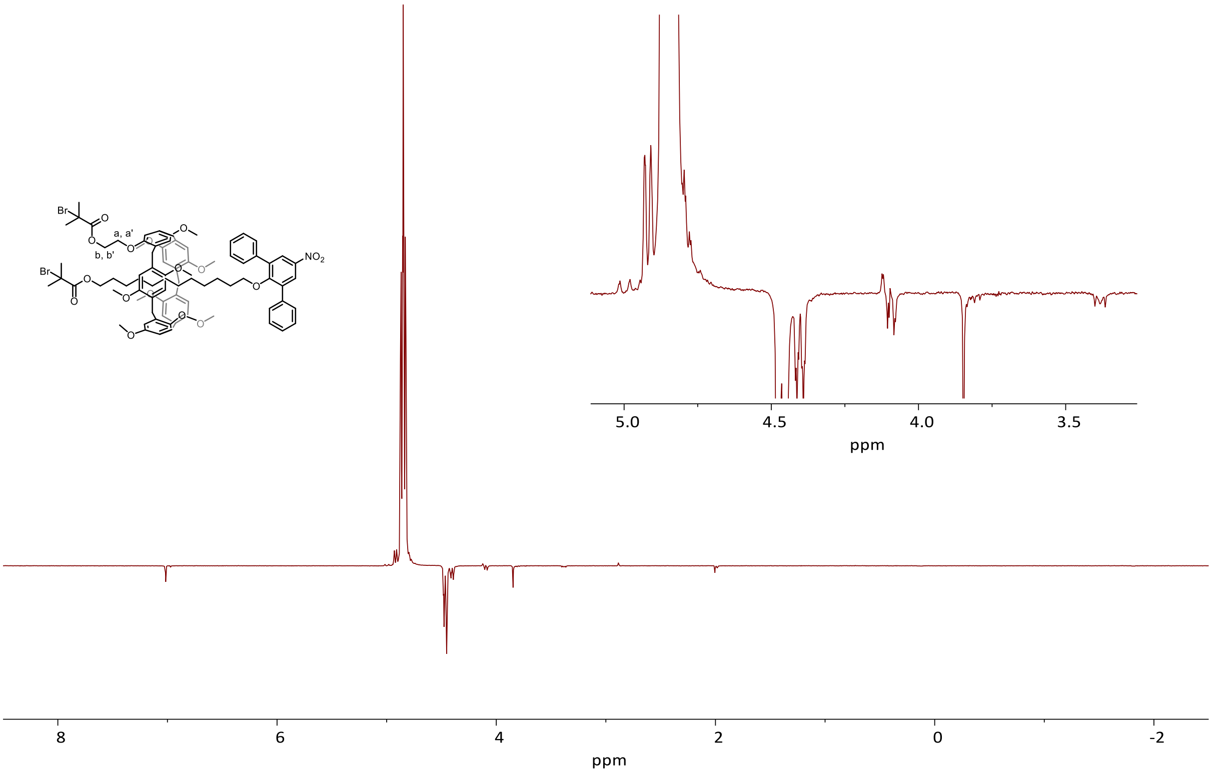

Spectrum S82. 1D selective ROESY ^{1}H NMR (500 MHz, Acetone-d_6, 298 K) spectrum of compound **S25*cis*** with selectively inverted proton H_a.

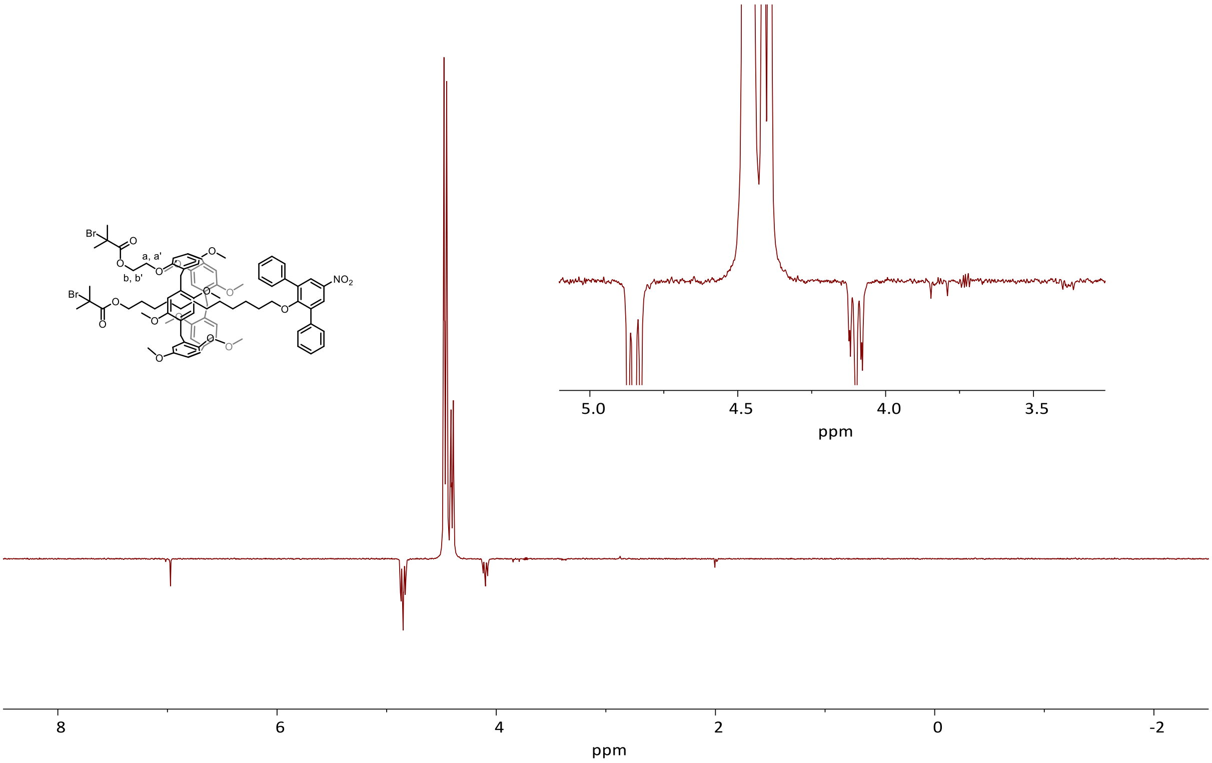

Spectrum S83. 1D selective ROESY ^{1}H NMR (500 MHz, Acetone-d_6, 298 K) spectrum of compound **S25*cis*** with selectively inverted proton $H_{a'}$.

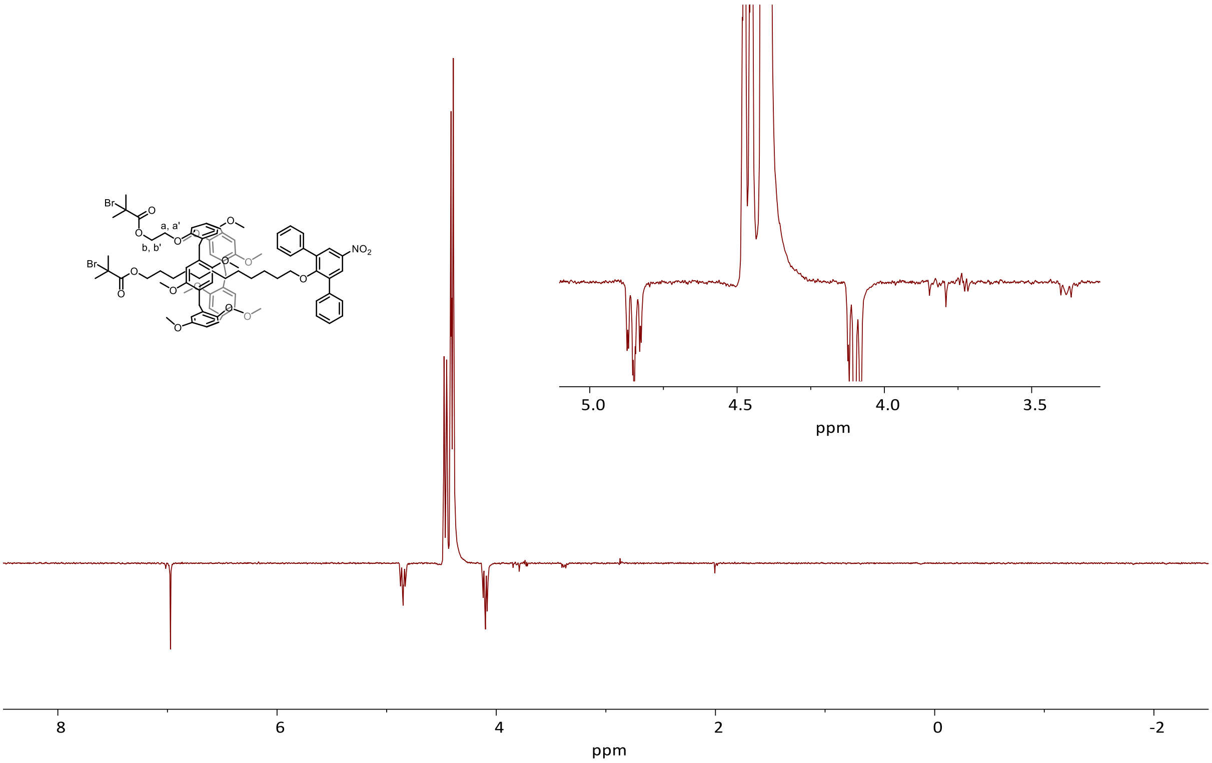

Spectrum S84. 1D selective ROESY ^{1}H NMR (500 MHz, Acetone-d_6, 298 K) spectrum of compound **S25$_{cis}$** with selectively inverted proton H_b.

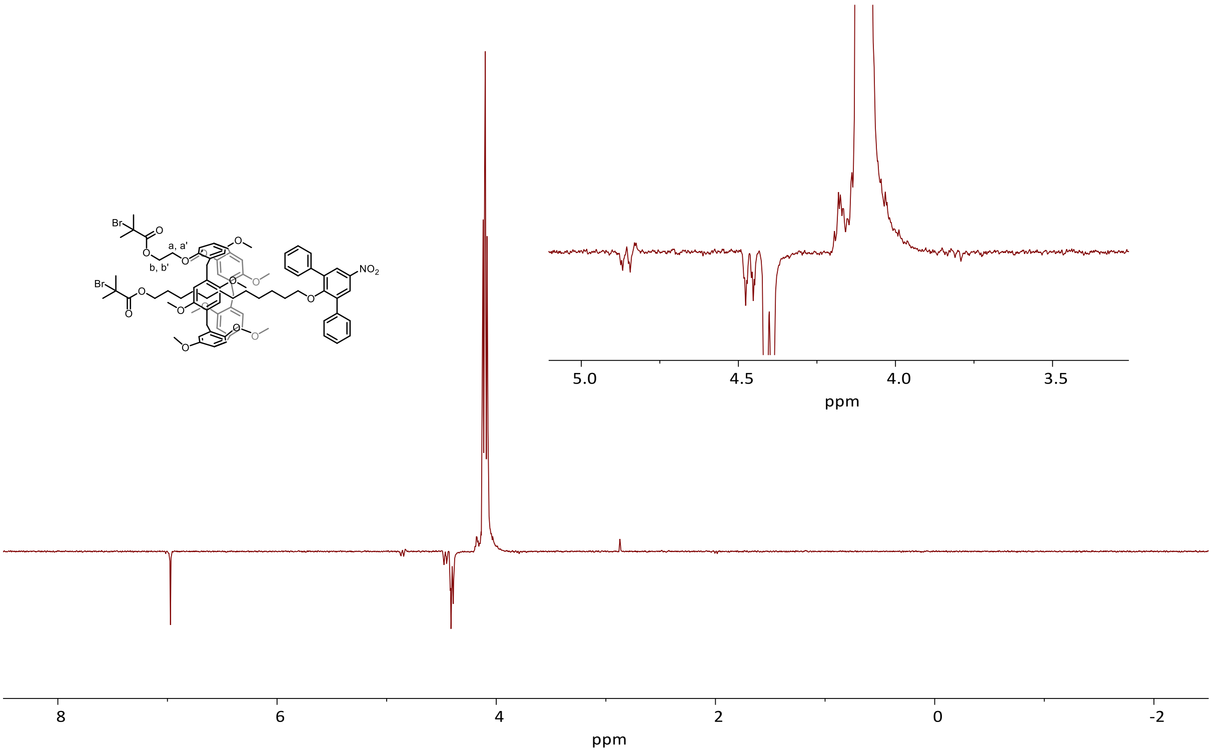

Spectrum S85. 1D selective ROESY ^{1}H NMR (500 MHz, Acetone-d_6, 298 K) spectrum of compound **S25$_{cis}$** with selectively inverted proton $H_{b'}$.

9.1.38 Spectra of S26*exo*

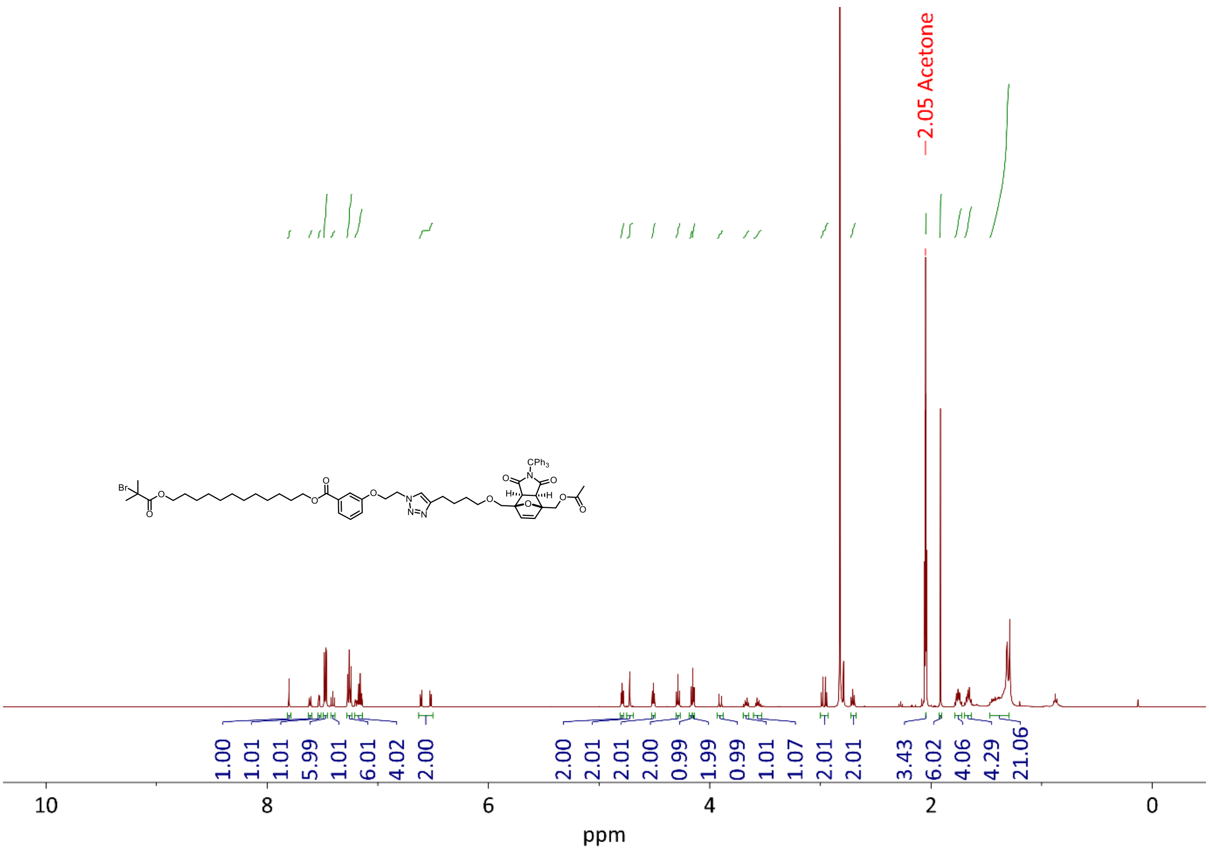

Spectrum S86. ^{1}H NMR (500 MHz, Acetone-d_6, 298 K) spectrum of compound **S26*exo***.

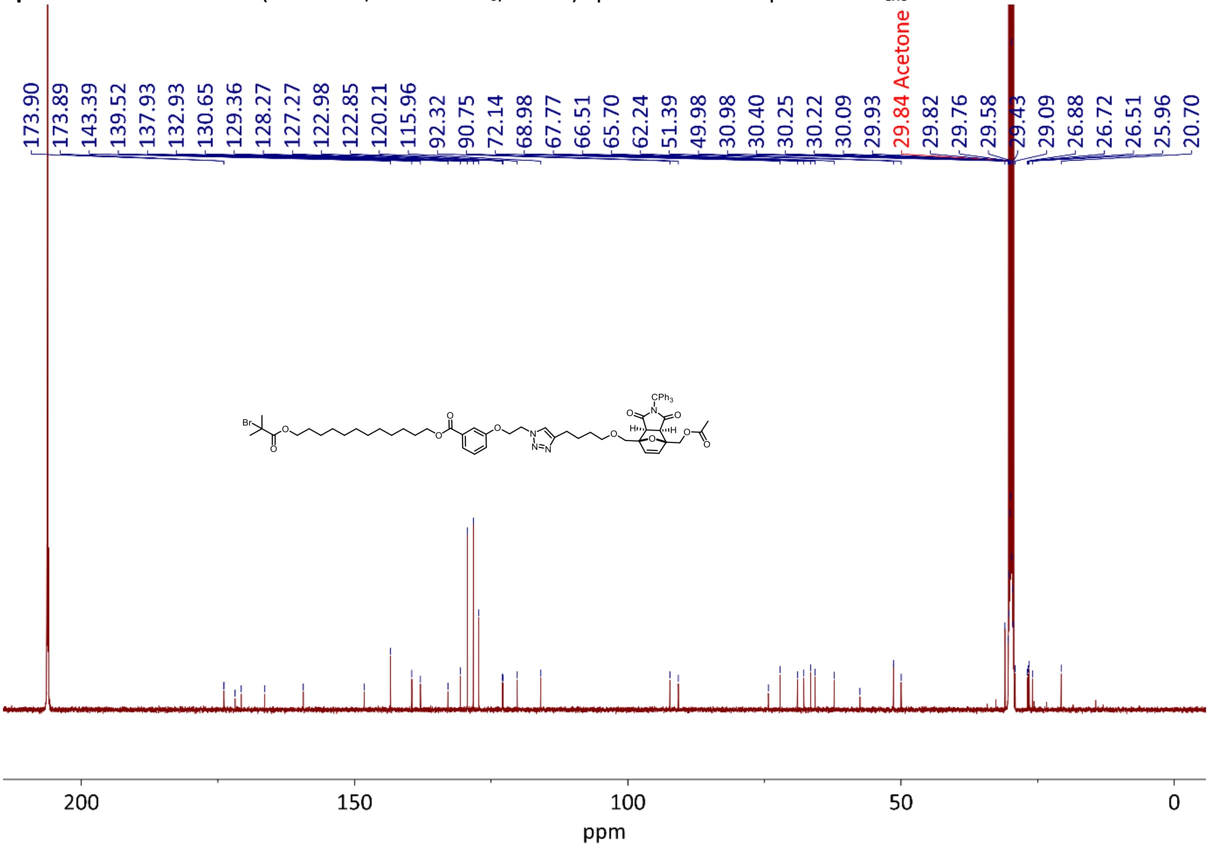

Spectrum S87. ^{13}C NMR (126 MHz, Acetone-d_6, 298 K) spectrum of compound **S26*exo***.

9.1.39 Spectra of S26$_{endo}$

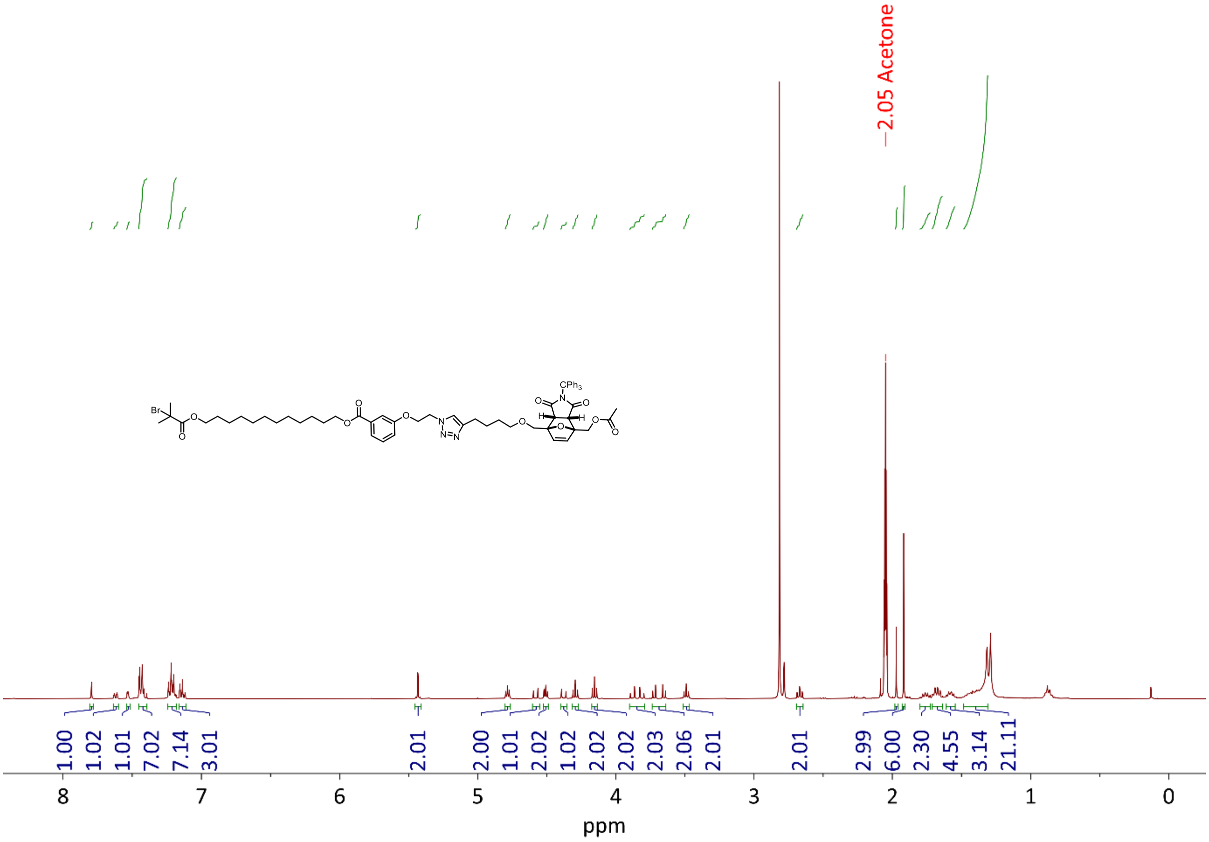

Spectrum S88. ^{1}H NMR (400 MHz, Acetone-d_6, 298 K) spectrum of compound **S26$_{endo}$**.

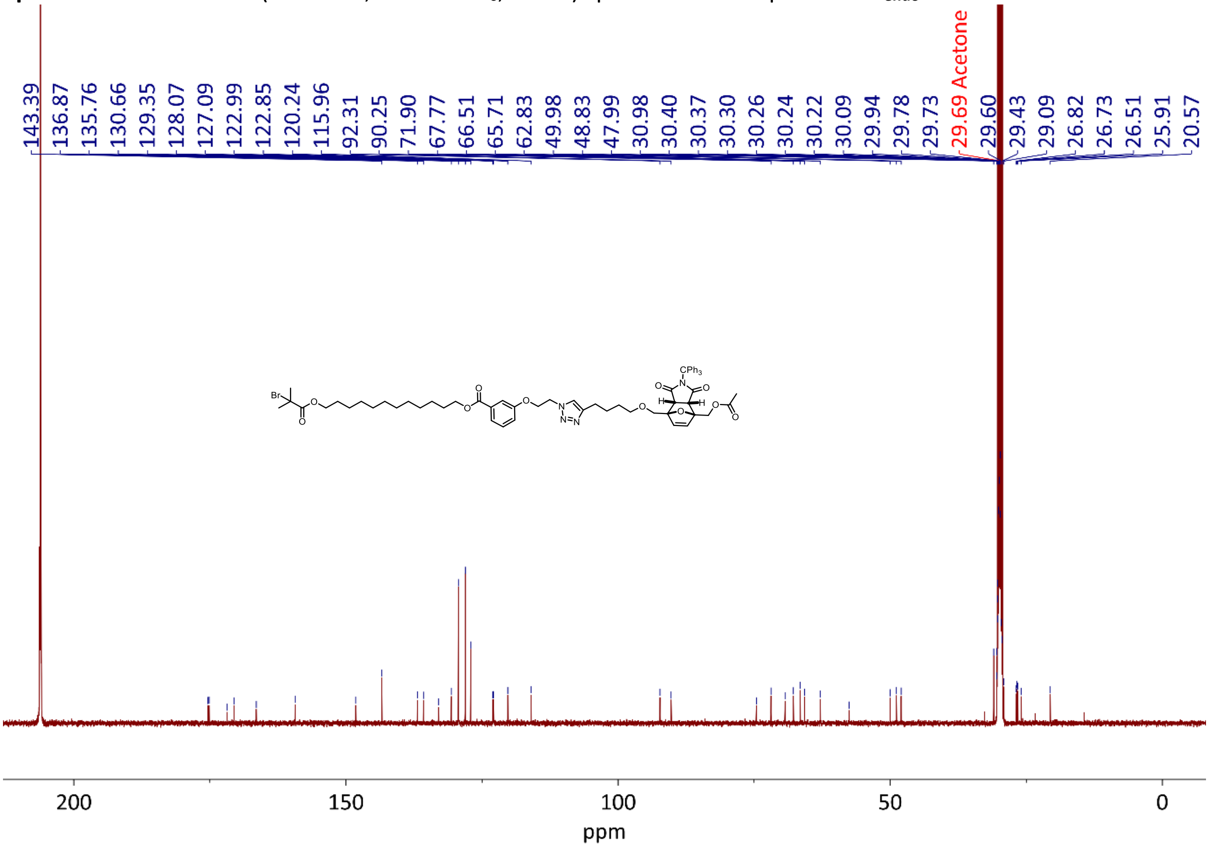

Spectrum S89. ^{13}C NMR (101 MHz, Acetone-d_6, 298 K) spectrum of compound **S26$_{endo}$**.

9.1.40 Spectra of S27$_{trans/exo}$

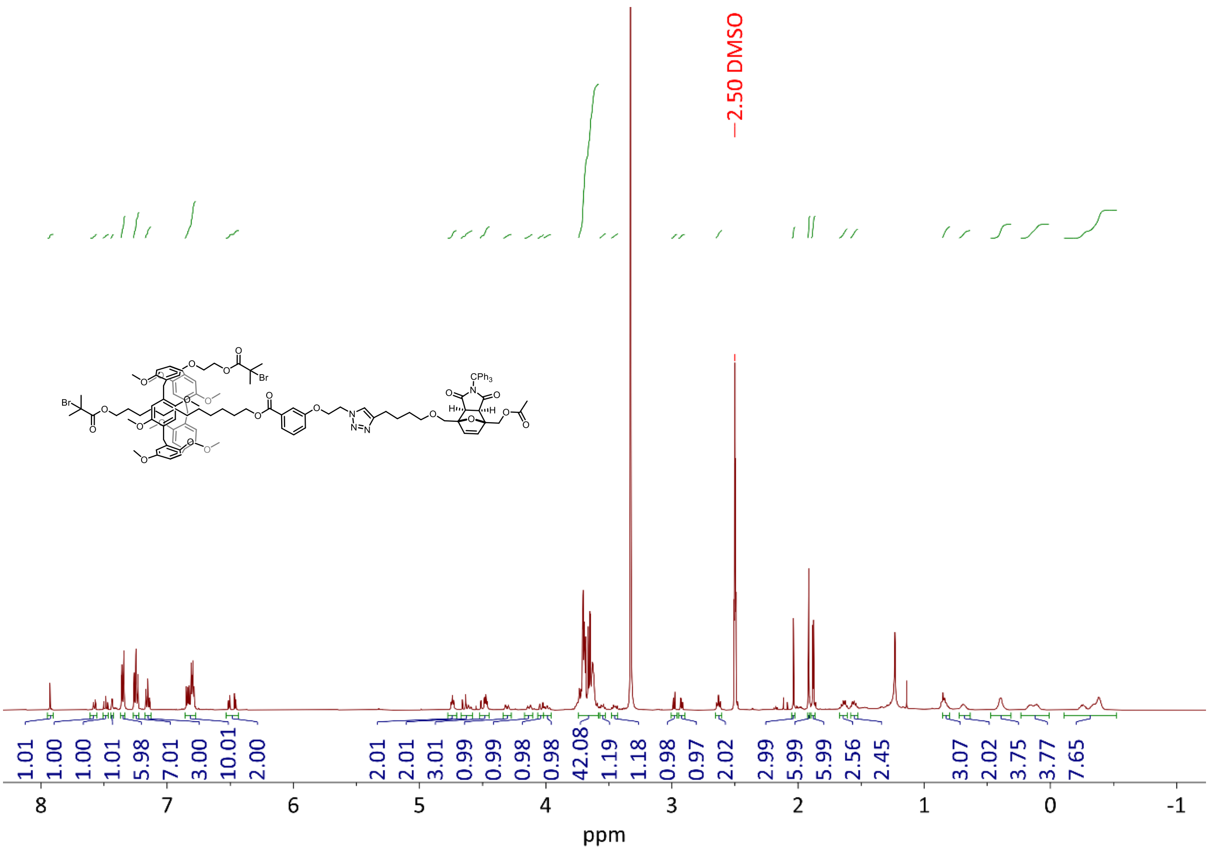

Spectrum S90. ^{1}H NMR (500 MHz, DMSO-d_6, 298 K) spectrum of compound **S27$_{trans/exo}$**.

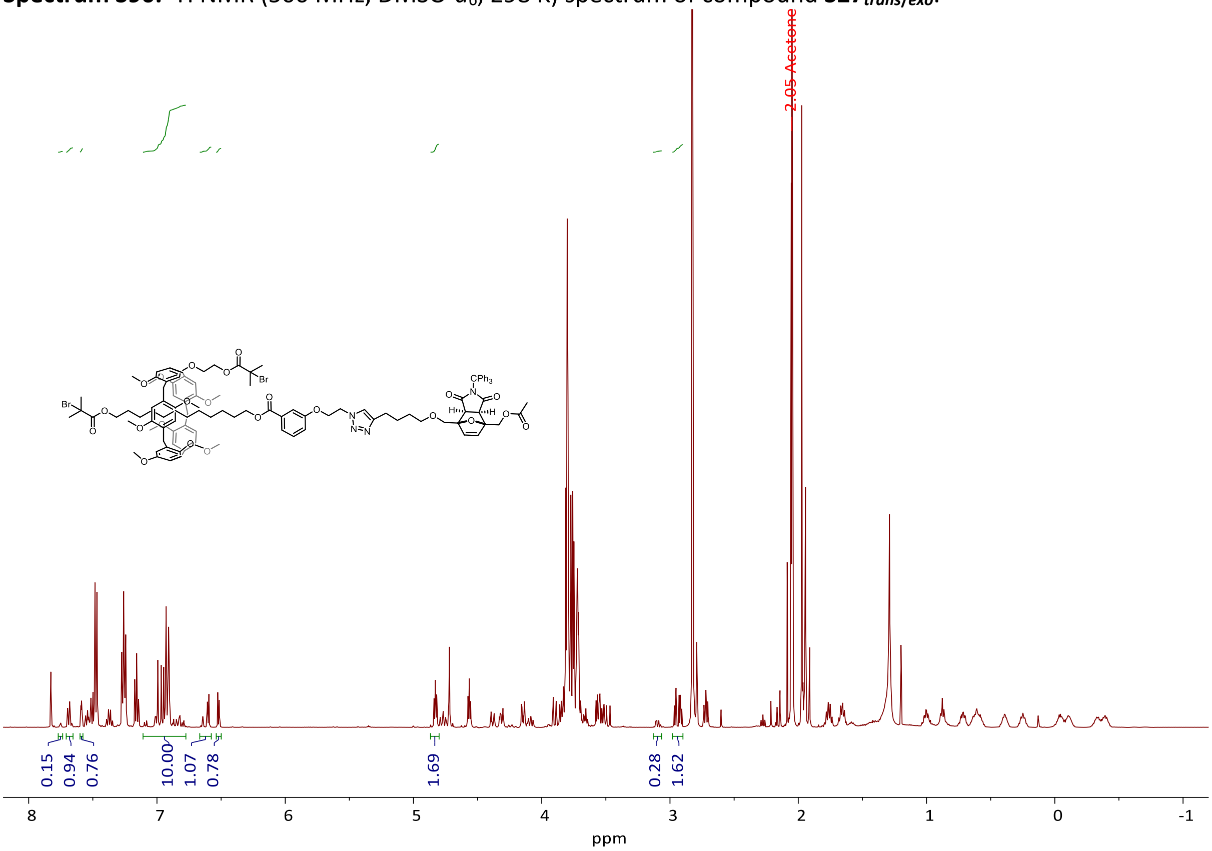

Spectrum S91. ^{1}H NMR (500 MHz, Acetone-d_6, 298 K) spectrum of compound **S27$_{trans/exo}$**.

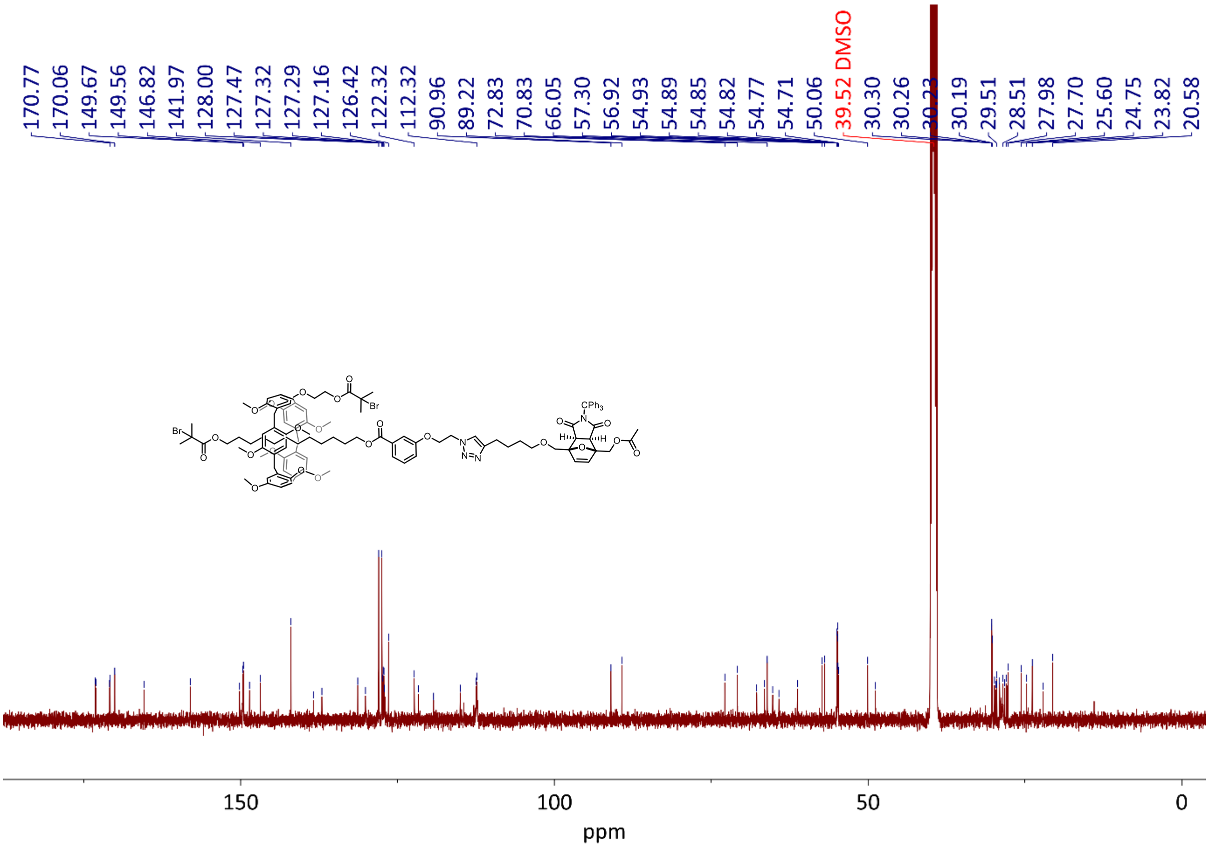

Spectrum S92. ^{13}C NMR (126 MHz, DMSO-d_6, 298 K) spectrum of compound **S27*trans/exo***.

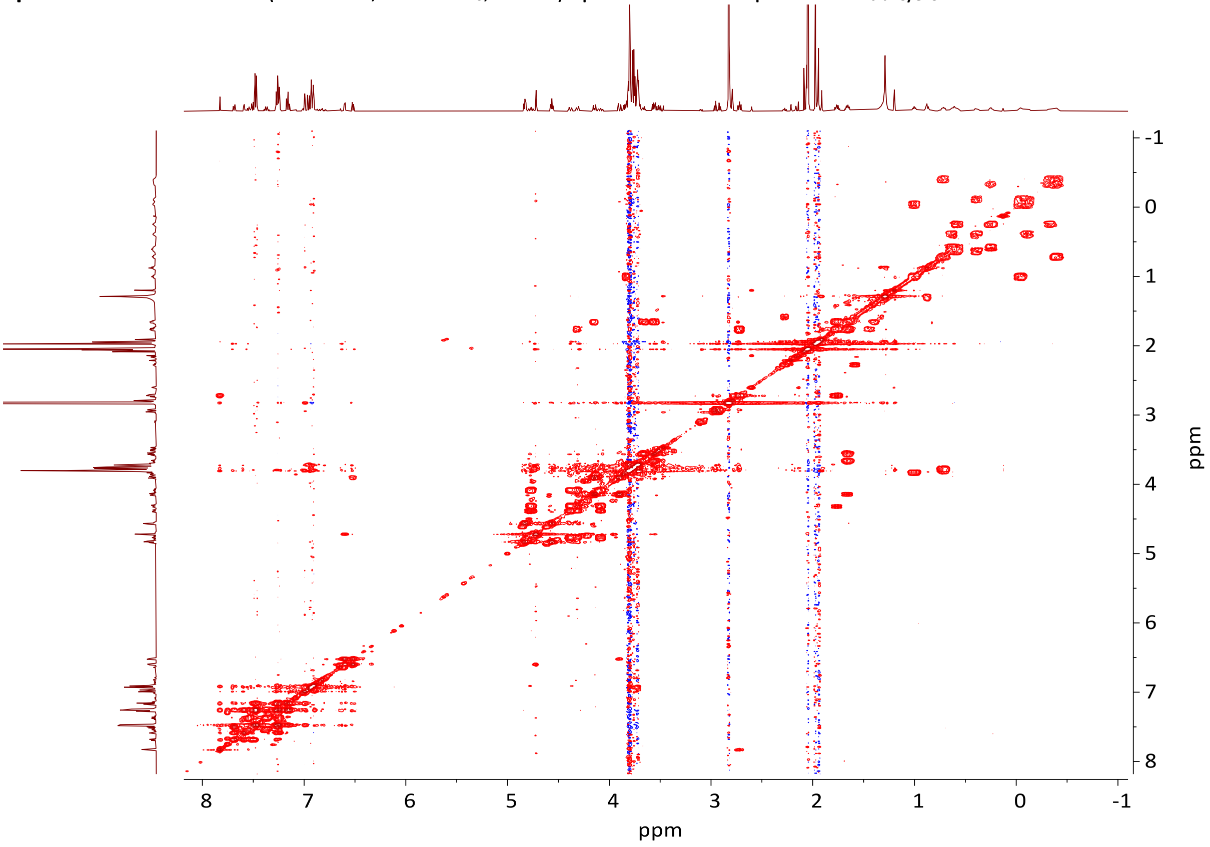

Spectrum S93. COSY ^{1}H NMR (500 MHz, Acetone-d_6, 298 K) spectrum of compound **S27*trans/exo***.

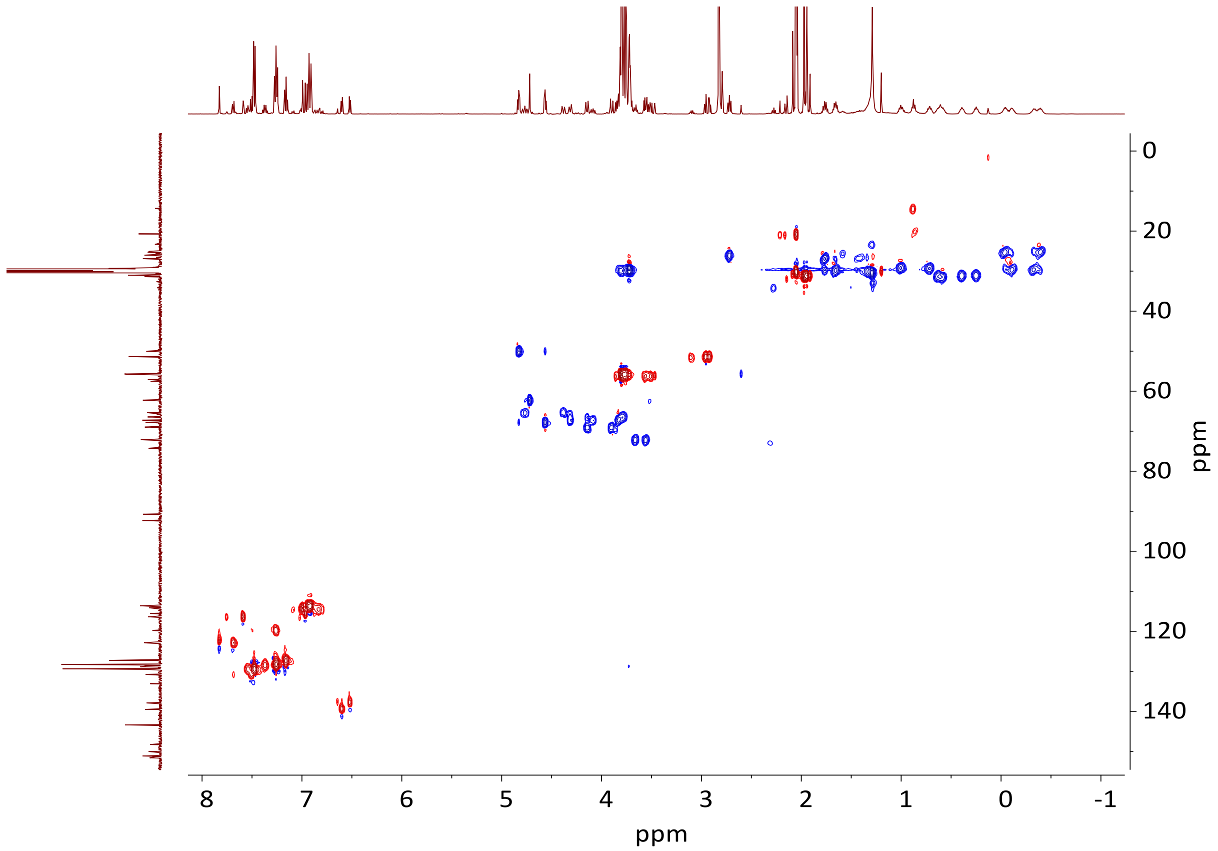

Spectrum S94. HSQC ^{1}H-^{13}C NMR (500 MHz, Acetone-d_6, 298 K) spectrum of compound $\mathbf{S27}_{\textit{trans/exo}}$.

9.1.41 Spectra of S27$_{cis/exo}$

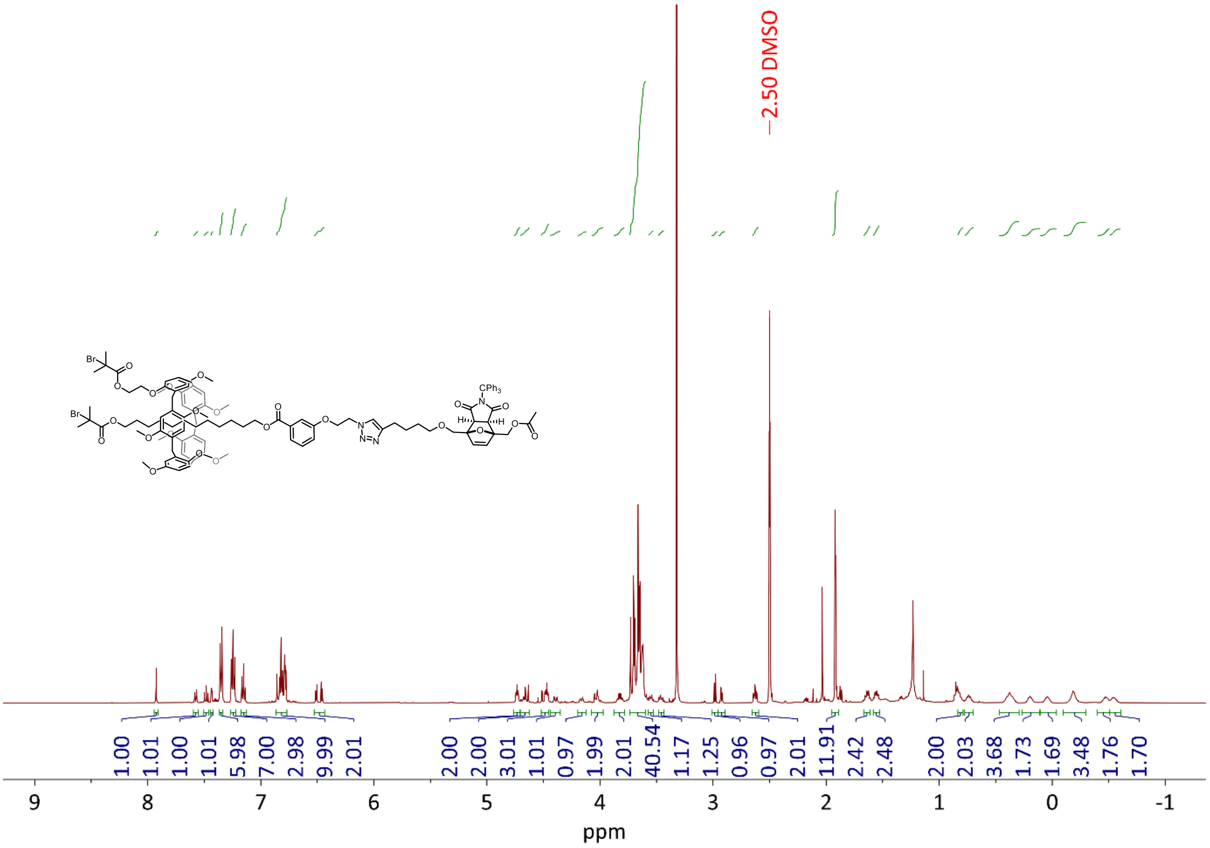

Spectrum S95. ^{1}H NMR (500 MHz, DMSO-d_6, 298 K) spectrum of compound **S27**$_{cis/exo}$.

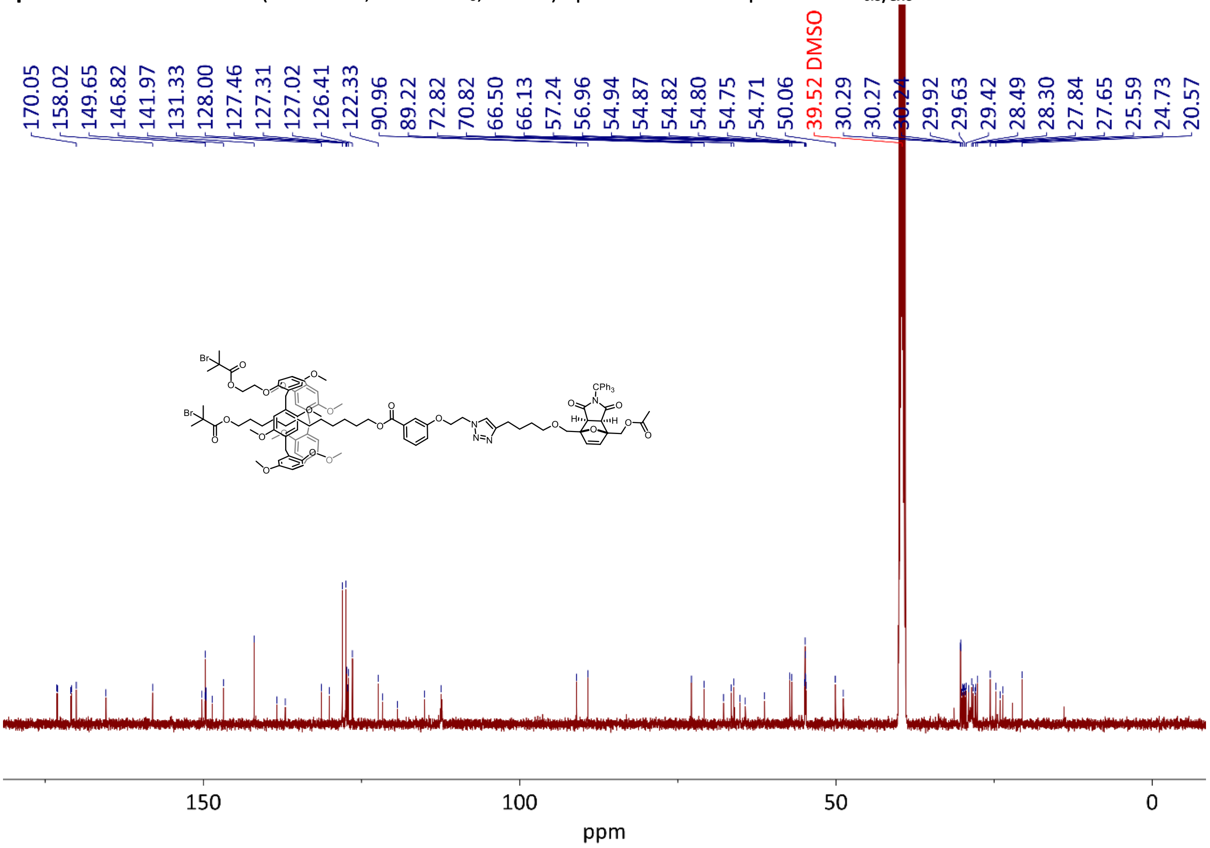

Spectrum S96. ^{13}C NMR (126 MHz, DMSO-d_6, 298 K) spectrum of compound **S27**$_{cis/exo}$.

9.1.42 Spectra of S27$_{trans/endo}$

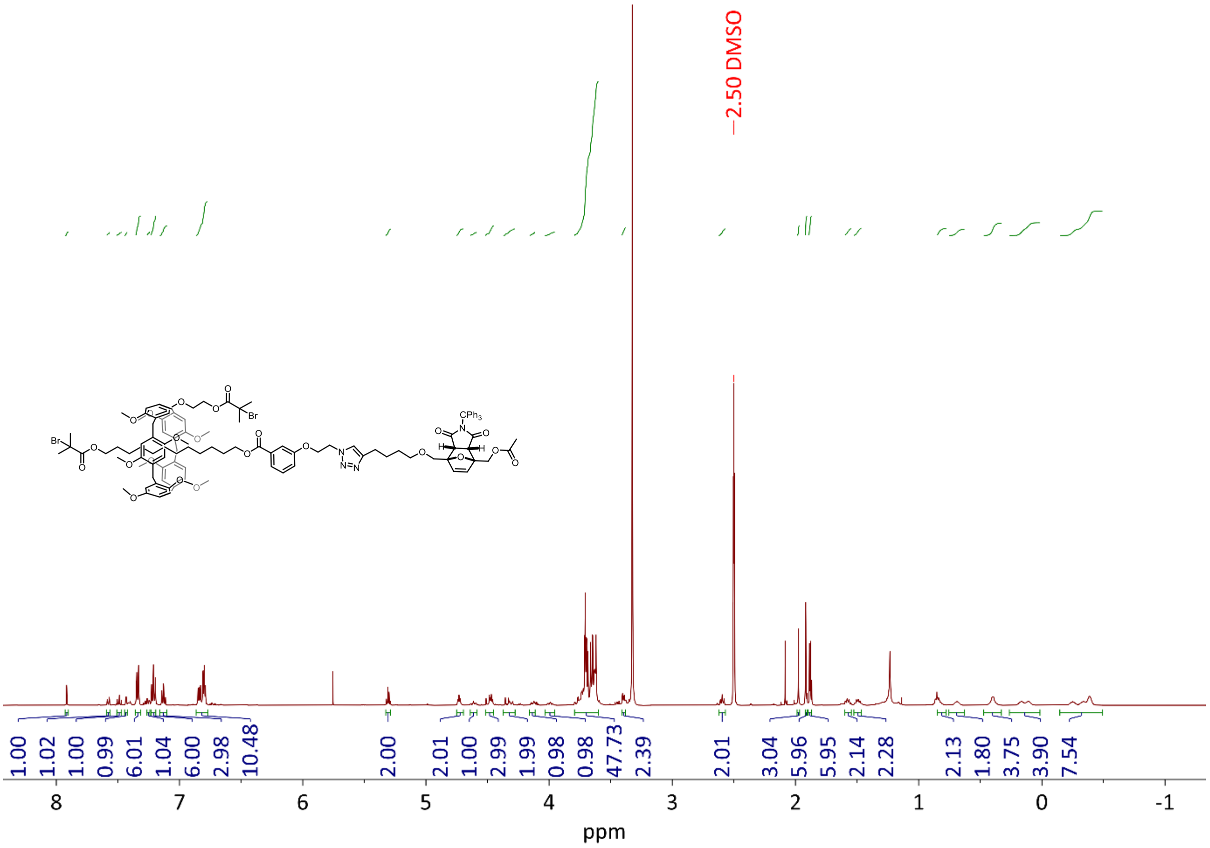

Spectrum S97. ^{1}H NMR (500 MHz, DMSO-d_6, 298 K) spectrum of compound **S27*trans/endo***.

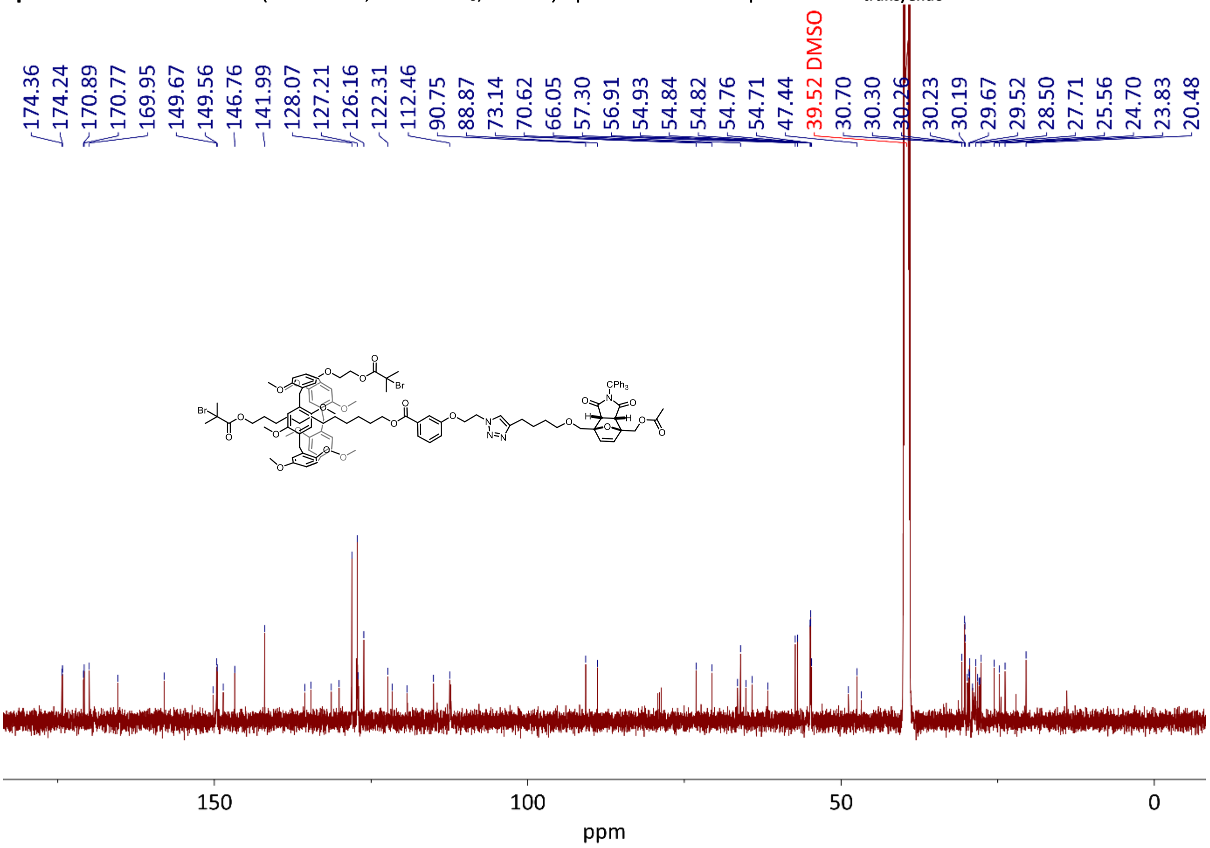

Spectrum S98. ^{13}C NMR (126 MHz, DMSO-d_6, 298 K) spectrum of compound **S27*trans/endo***.

9.1.43 Spectra of S27$_{cis/endo}$

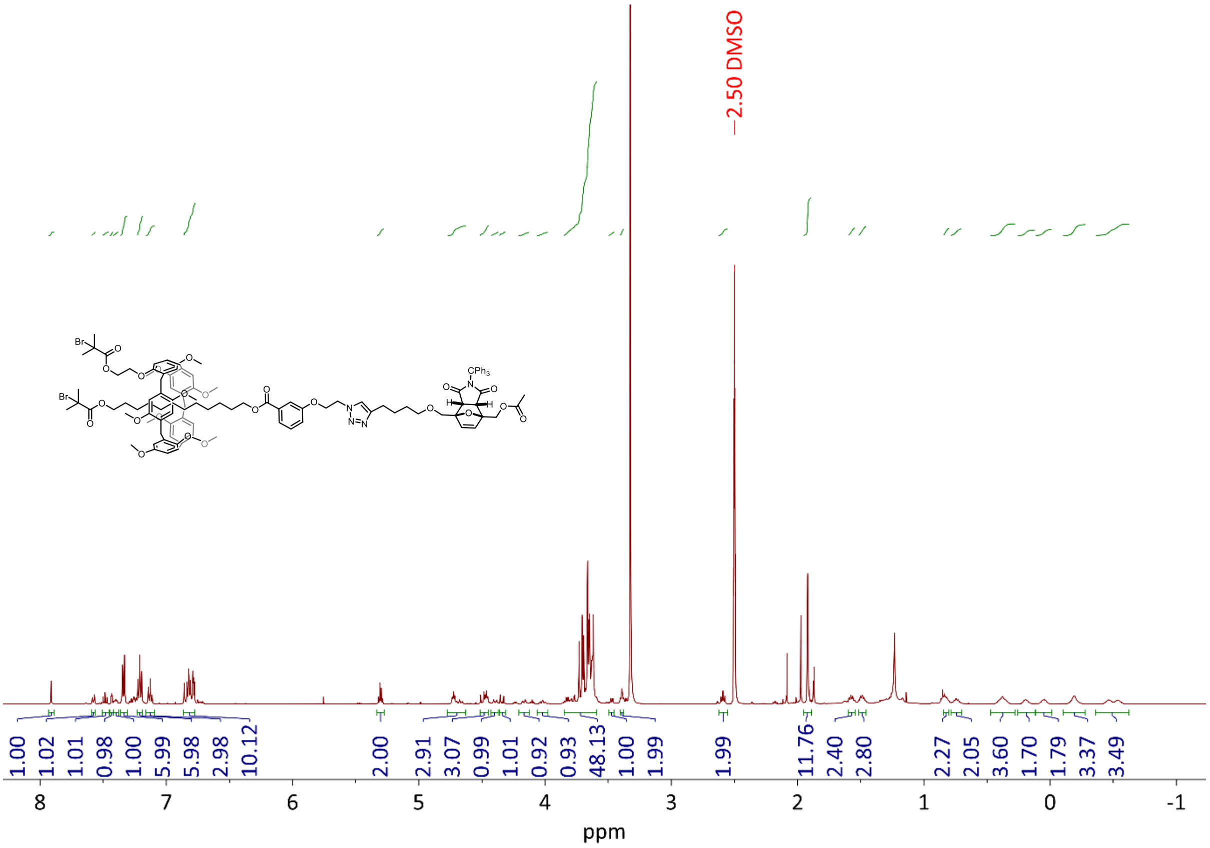

Spectrum S99. ^{1}H NMR (500 MHz, DMSO-d_6, 298 K) spectrum of compound **S27**$_{cis/endo}$.

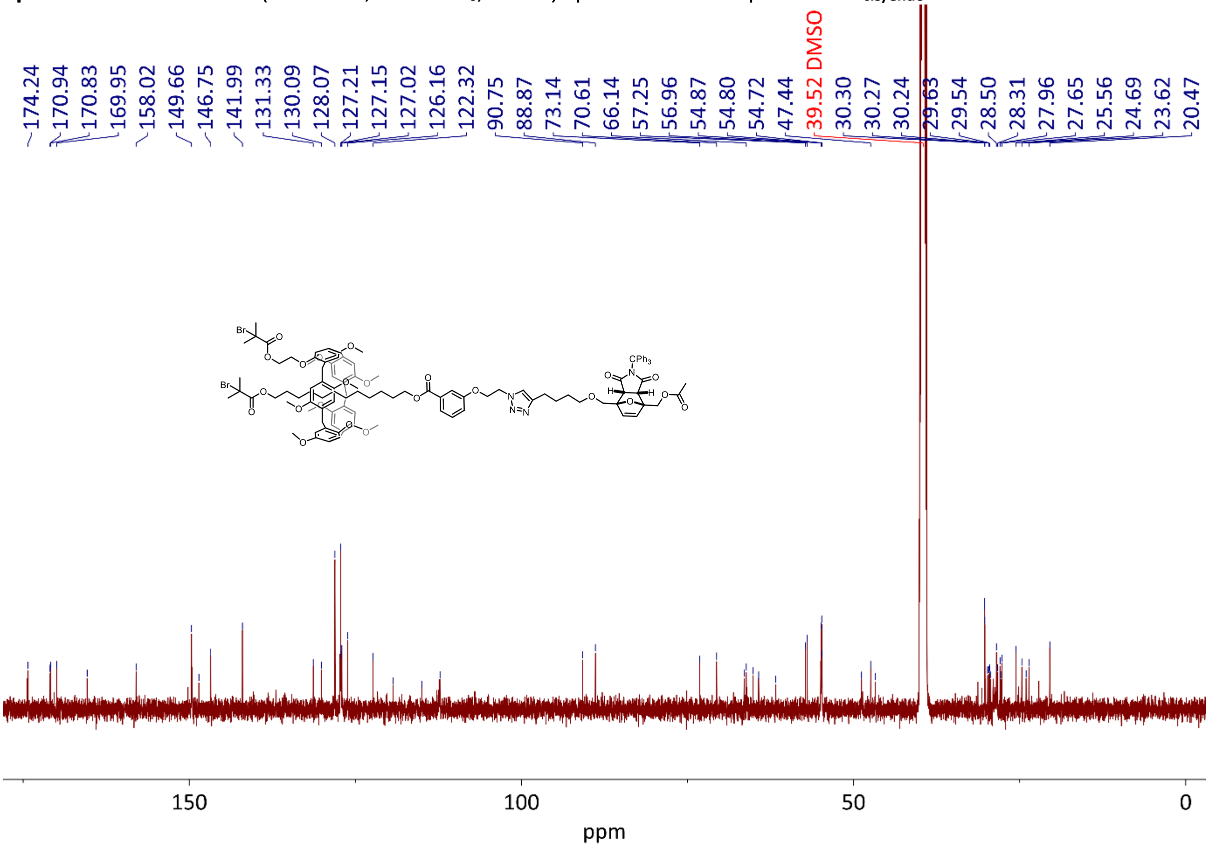

Spectrum S100. ^{13}C NMR (126 MHz, DMSO-d_6, 298 K) spectrum of compound **S27**$_{cis/endo}$.

9.1.44 Spectra of 8_3a

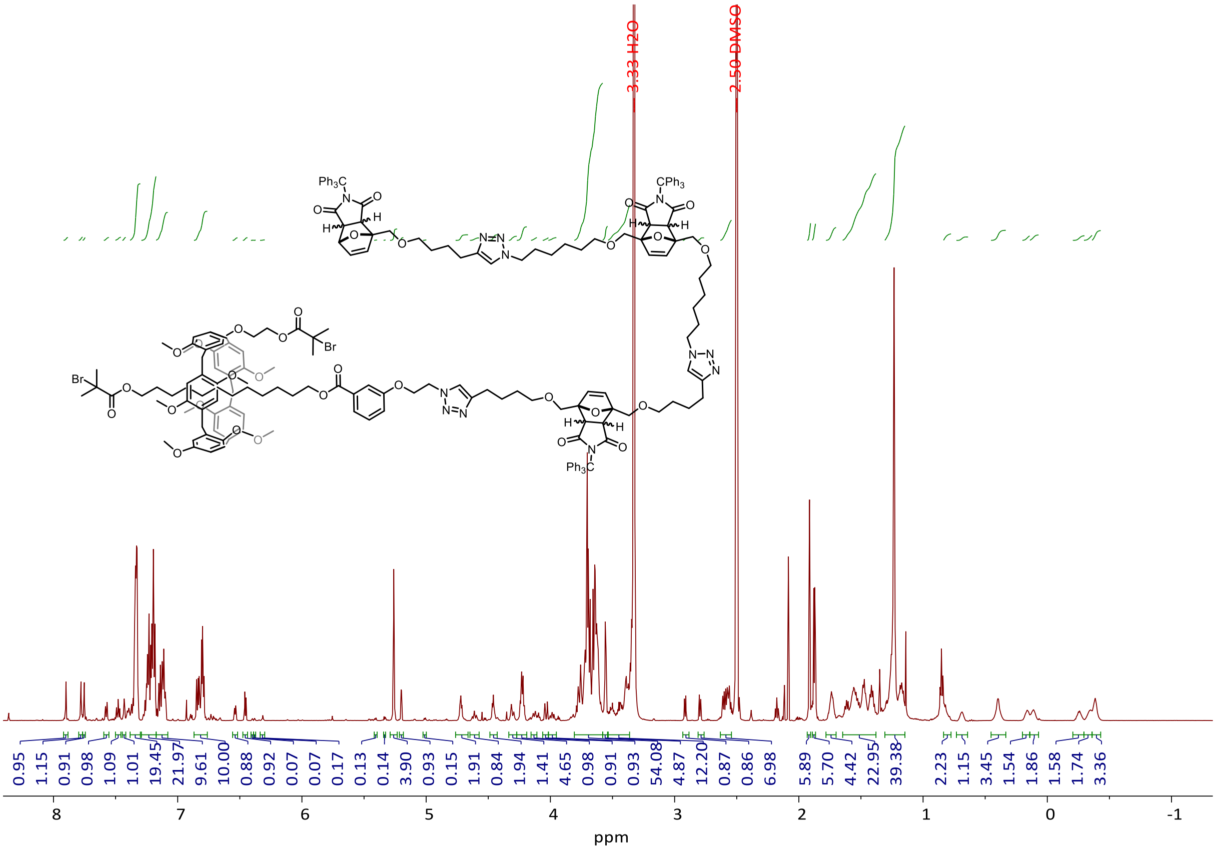

Spectrum S101. ^{1}H NMR (600 MHz, DMSO-d_6, 298 K) spectrum of compound **8_3a**.

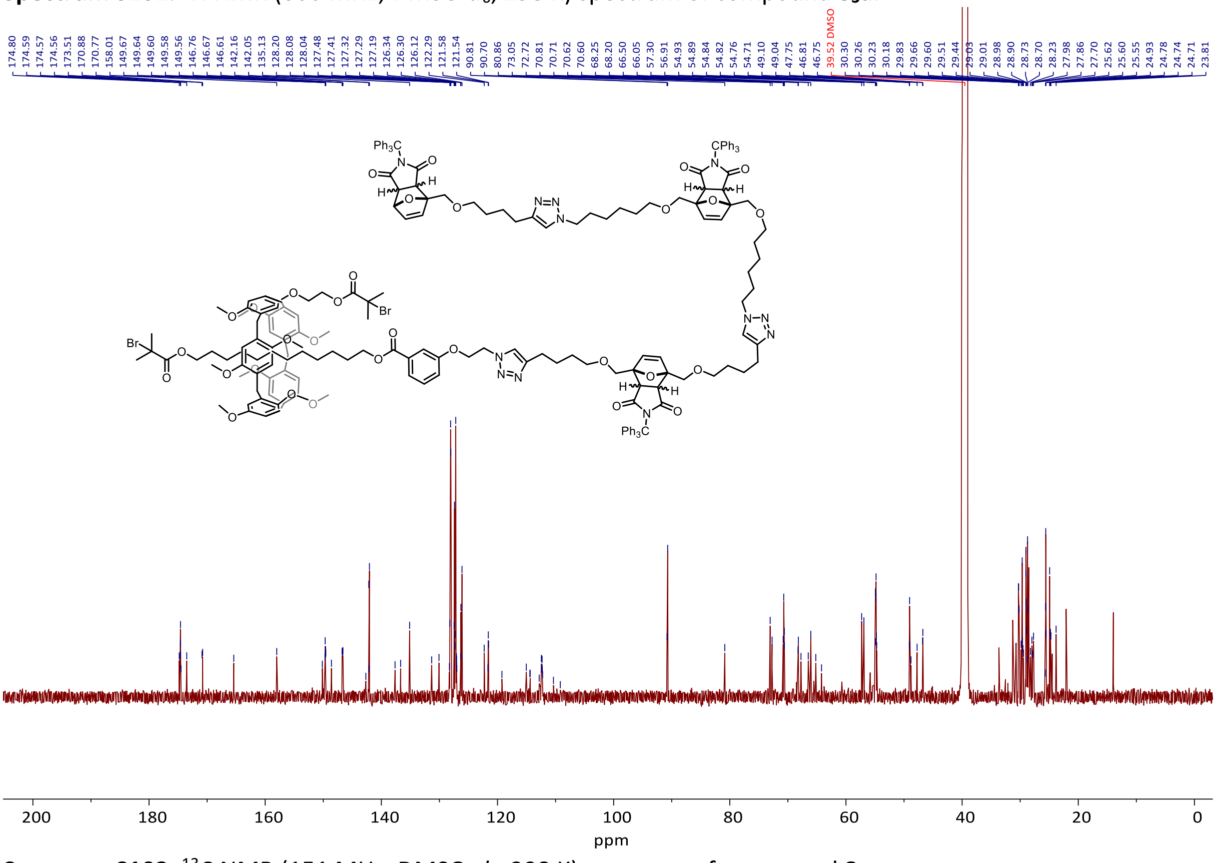

Spectrum S102. ^{13}C NMR (151 MHz, DMSO-d_6, 298 K) spectrum of compound **8_3a**.

9.1.45 Spectra of 8_3b

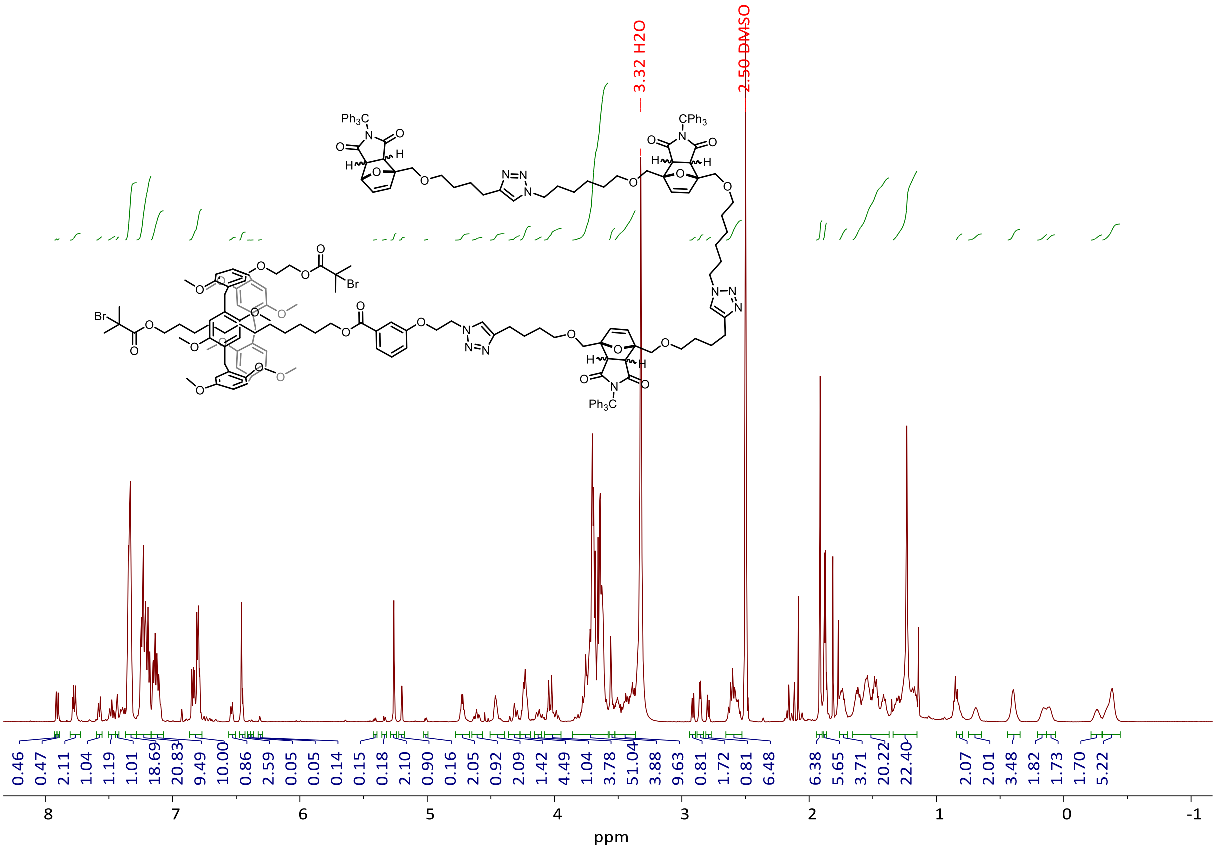

Spectrum S103. ^{1}H NMR (500 MHz, DMSO-d_6, 298 K) spectrum of compound **8_3b**.

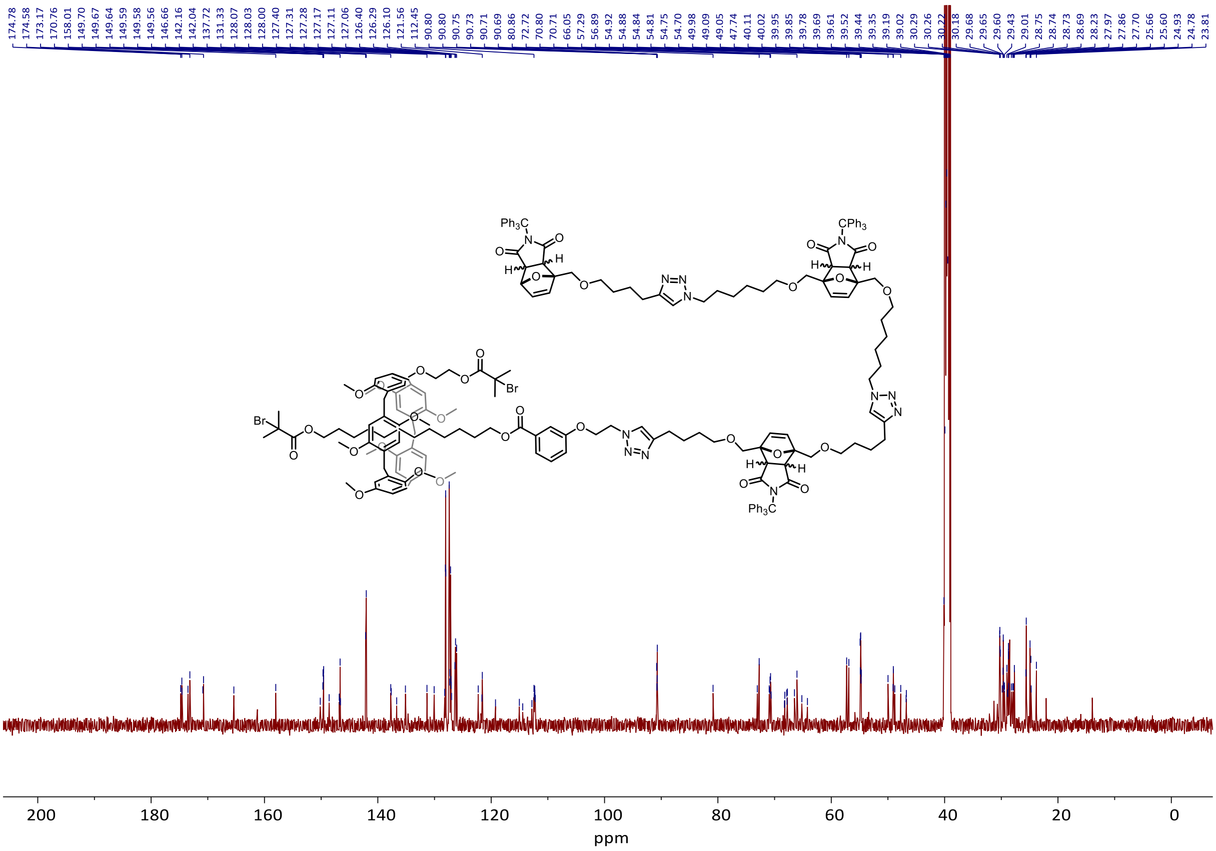

Spectrum S104. ^{13}C NMR (126 MHz, DMSO-d_6, 298 K) spectrum of compound **8_3b**.

9.1.46 Spectra of 8_3c

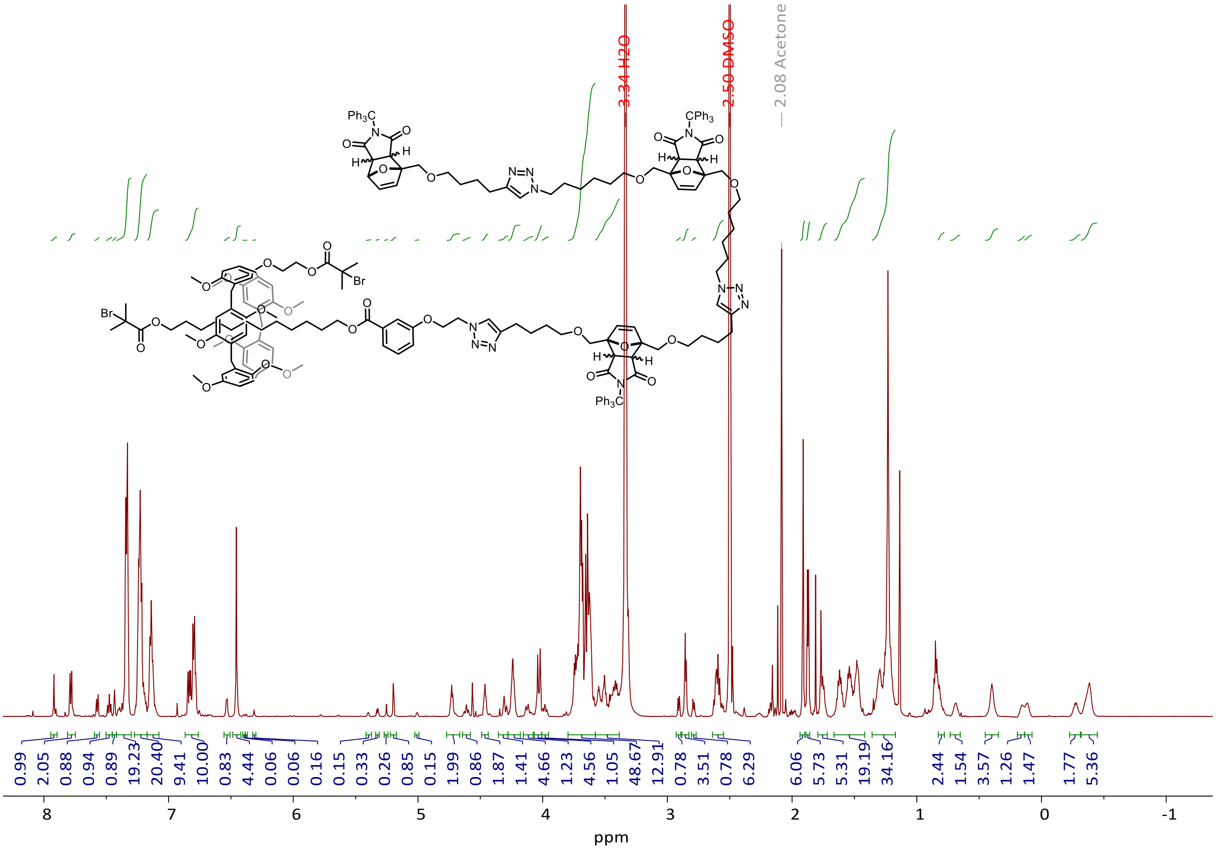

Spectrum S105. ^{1}H NMR (600 MHz, DMSO-d_6, 298 K) spectrum of compound **8_3c**.

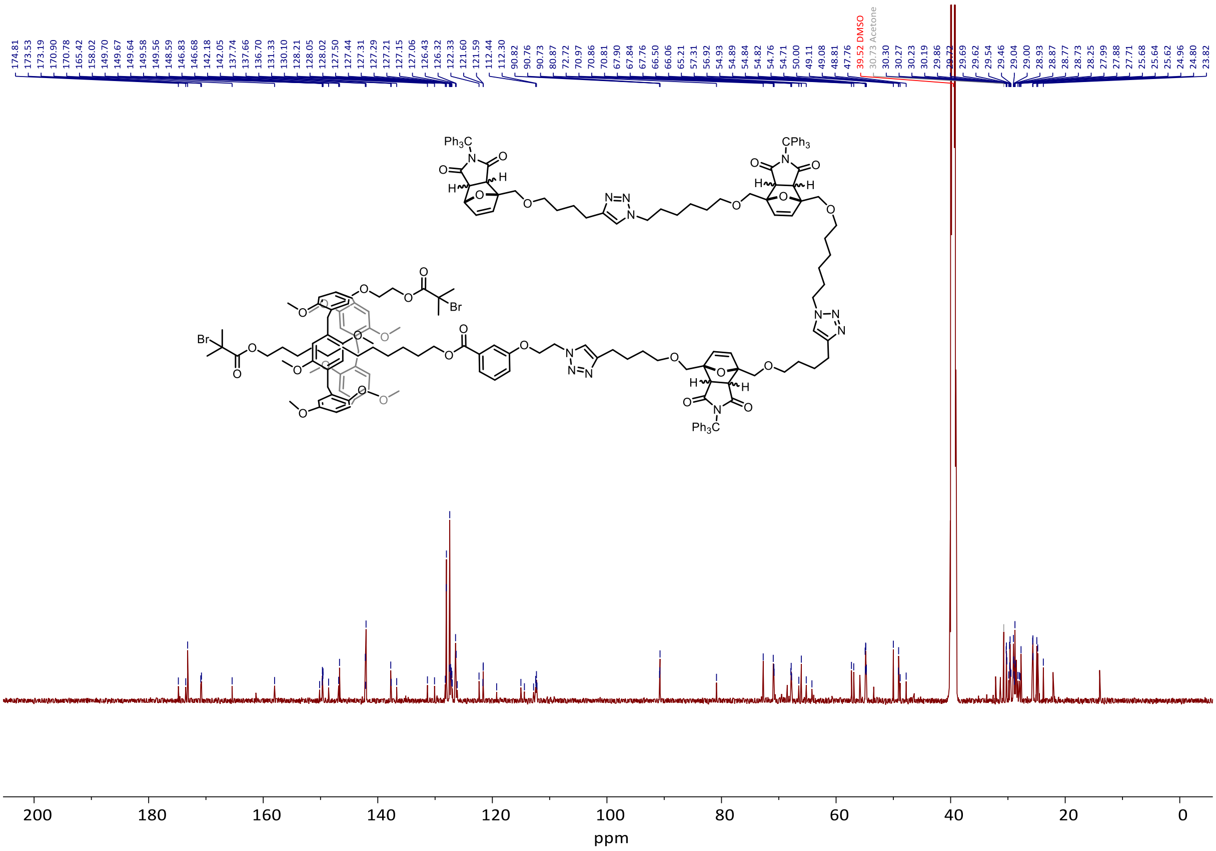

Spectrum S106. ^{13}C NMR (151 MHz, DMSO-d_6, 298 K) spectrum of compound **8_3c**.

9.1.47 Spectra of 8_3d

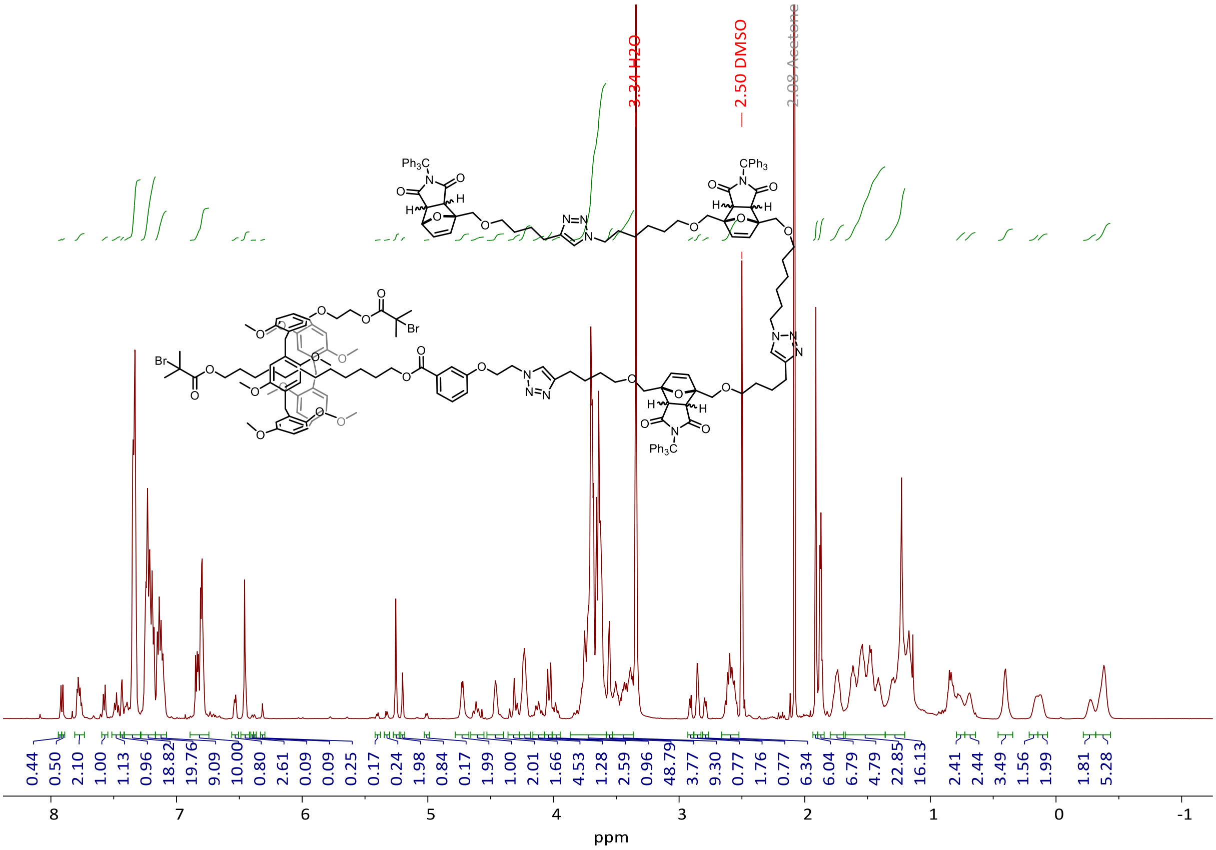

Spectrum S107. ^{1}H NMR (500 MHz, DMSO-d_6, 298 K) spectrum of compound **8_3d**.

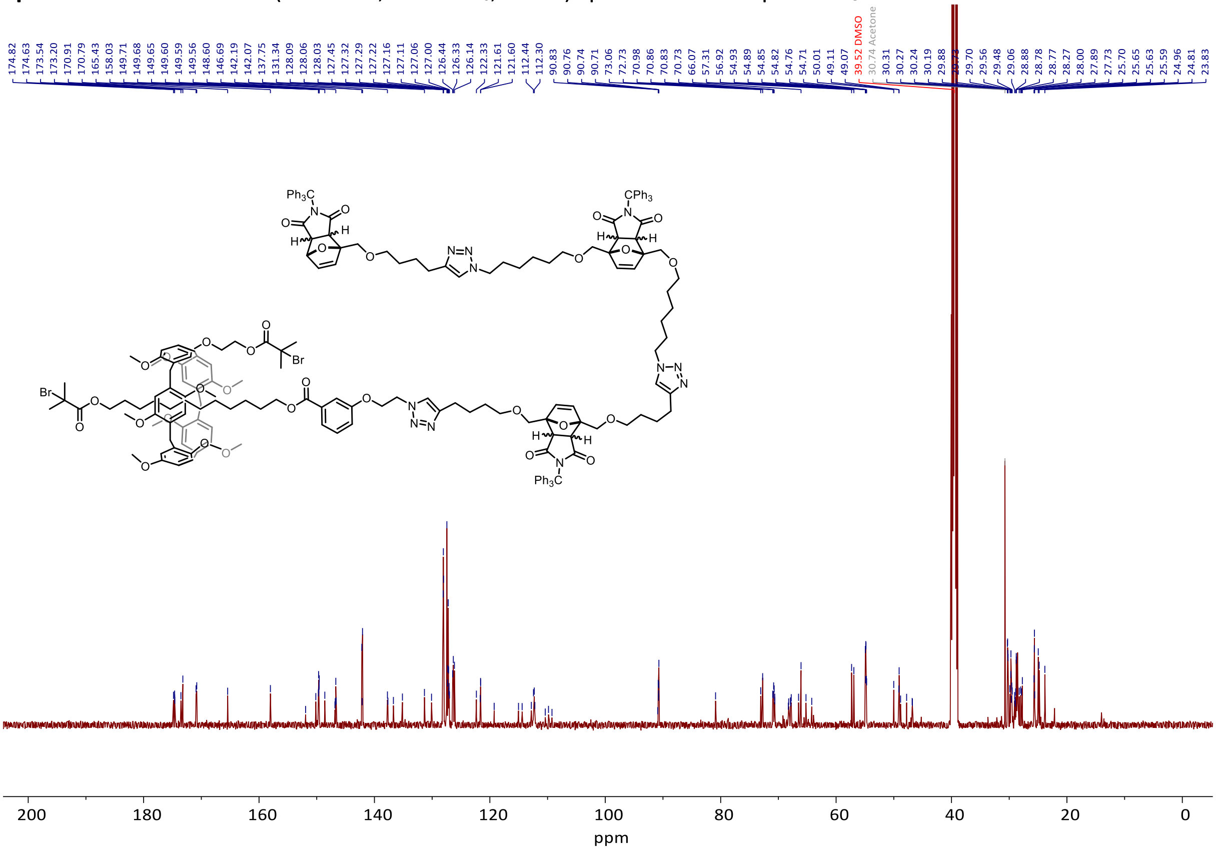

Spectrum S108. ^{13}C NMR (126 MHz, DMSO-d_6, 298 K) spectrum of compound **8_3d**.

9.1.48 Spectra of 8_5

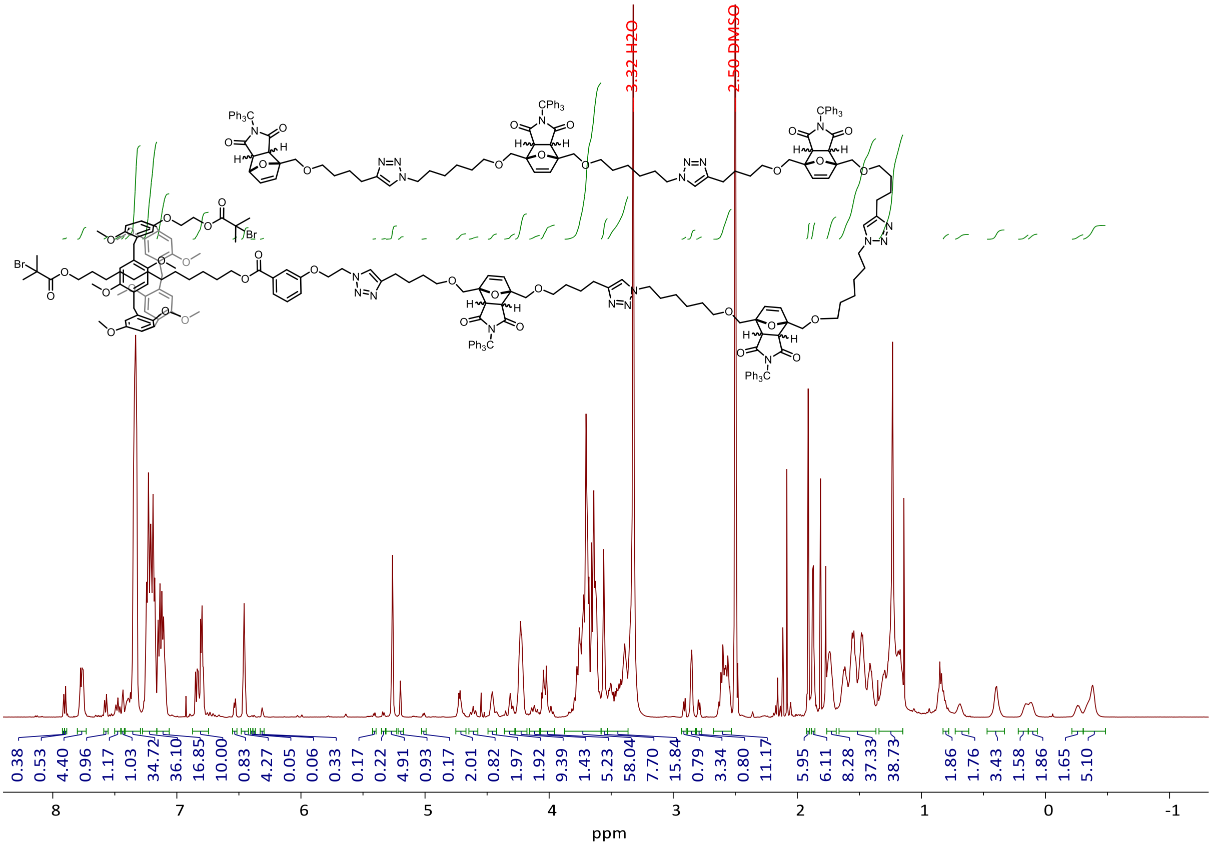

Spectrum S109. ^{1}H NMR (500 MHz, DMSO-d_6, 298 K) spectrum of compound **8_5**.

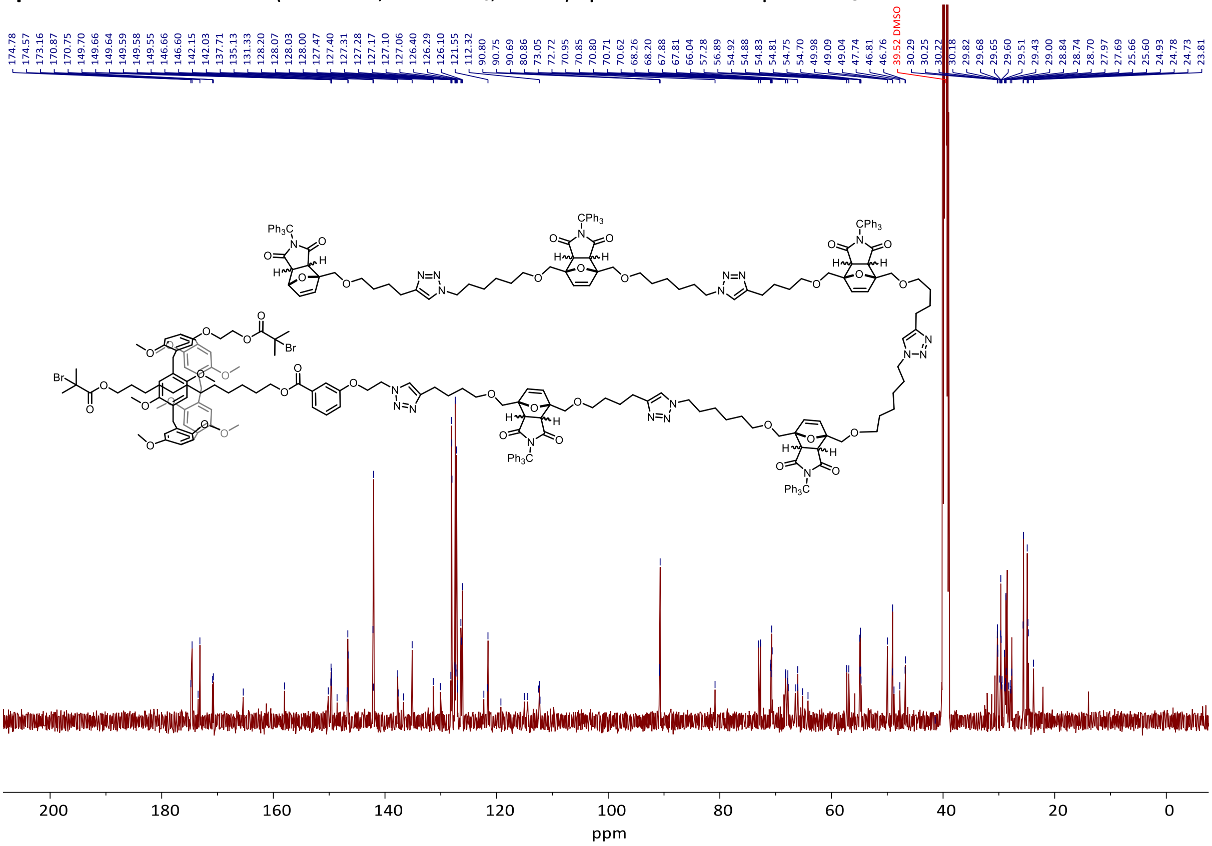

Spectrum S110. ^{13}C NMR (126 MHz, DMSO-d_6, 298 K) spectrum of compound **8_5**.

9.1.49 Spectra of S29

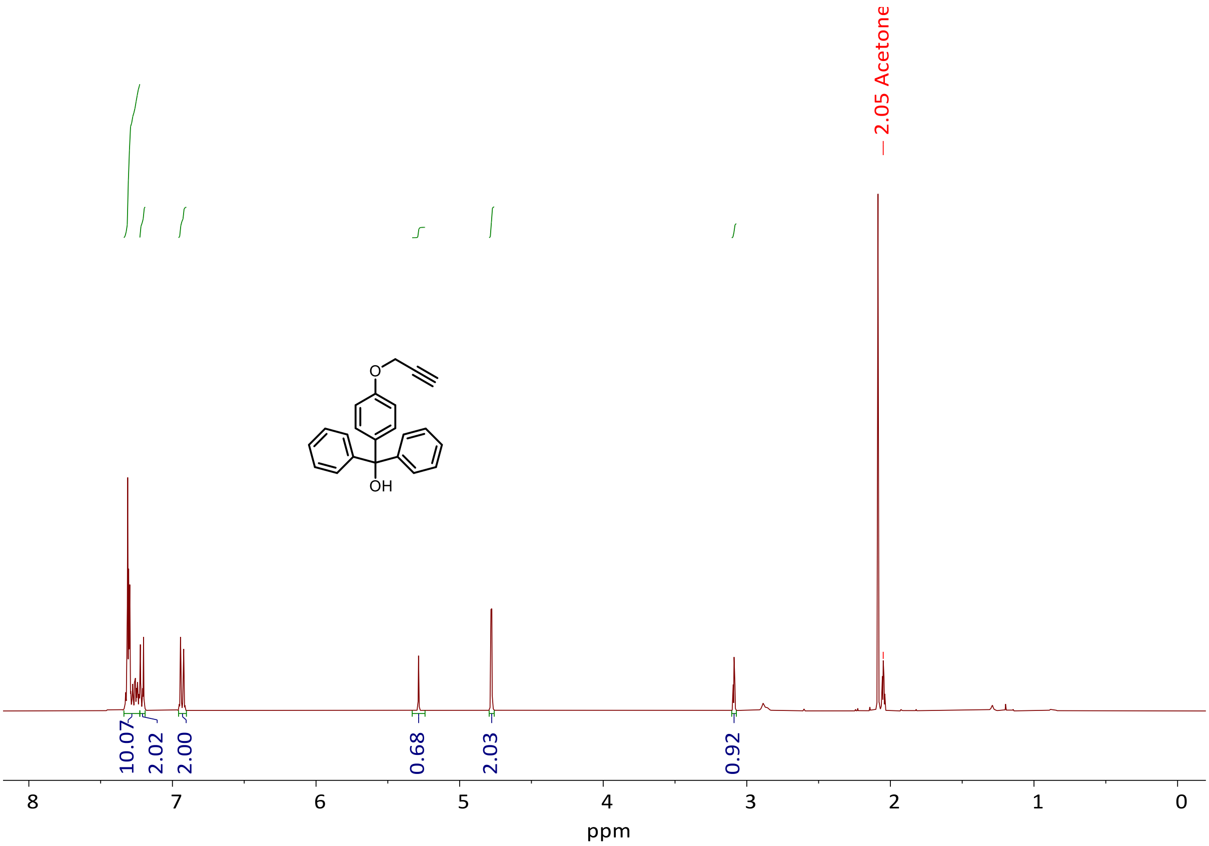

Spectrum S111. ^{1}H NMR (400 MHz, Acetone-d_6, 298 K) spectrum of compound **S29**.

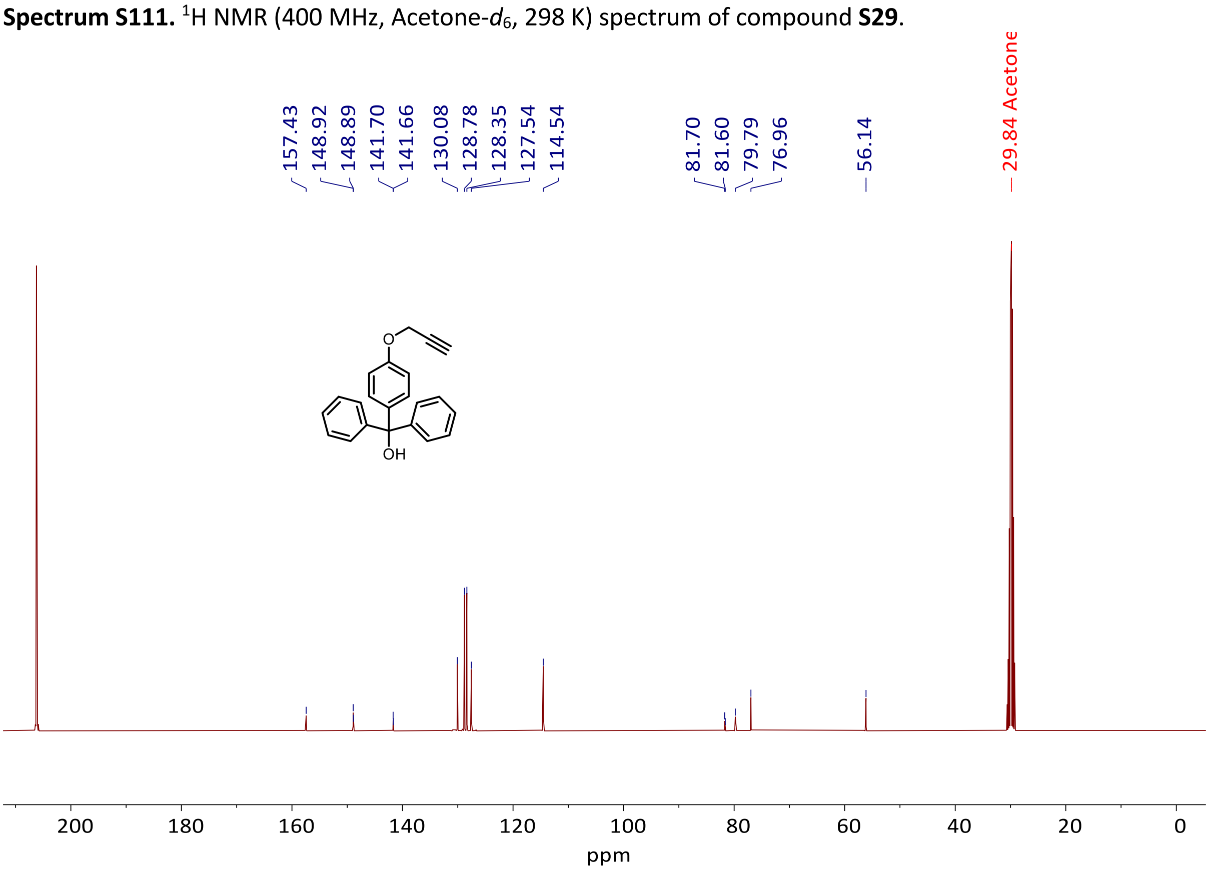

Spectrum S112. ^{13}C NMR (101 MHz, Acetone-d_6, 298 K) spectrum of compound **S29**.

9.1.50 Spectra of S30

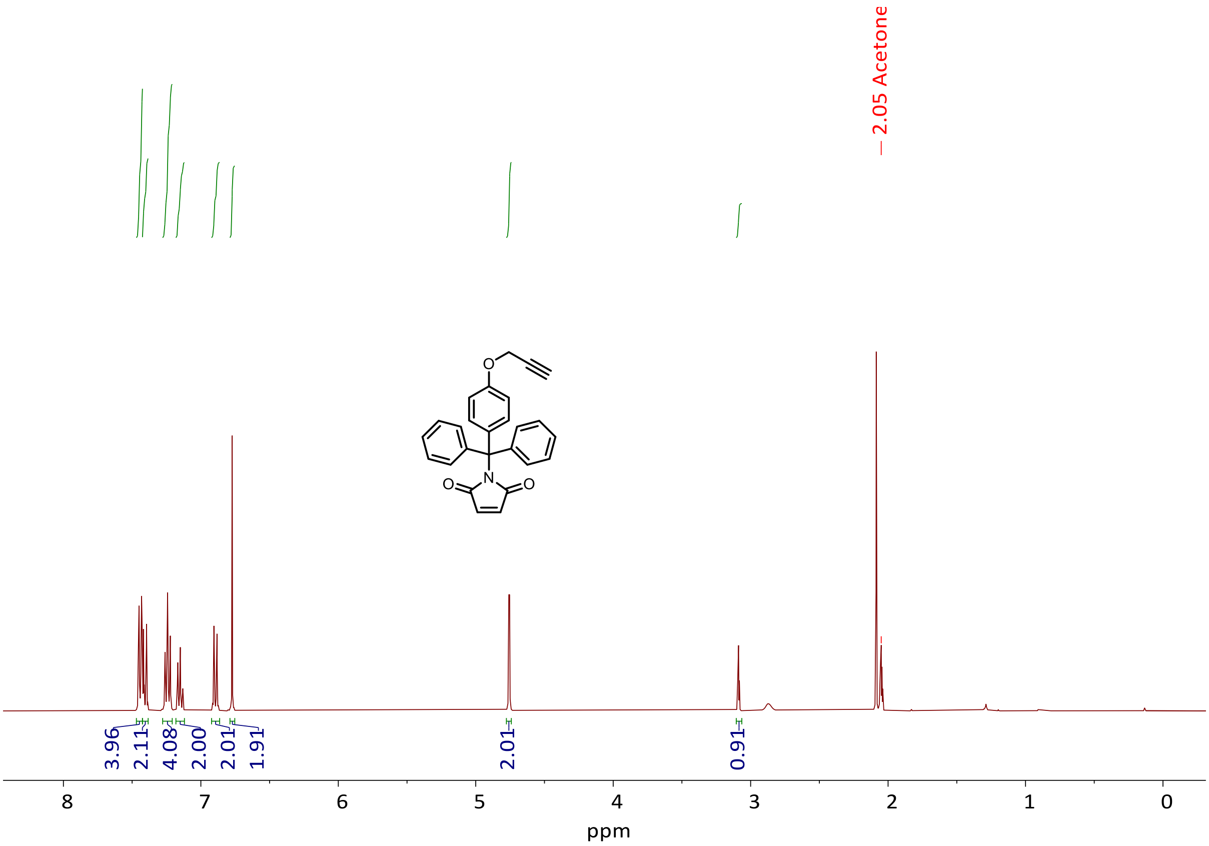

Spectrum S113. ^{1}H NMR (400 MHz, Acetone-d_6, 298 K) spectrum of compound **S30**.

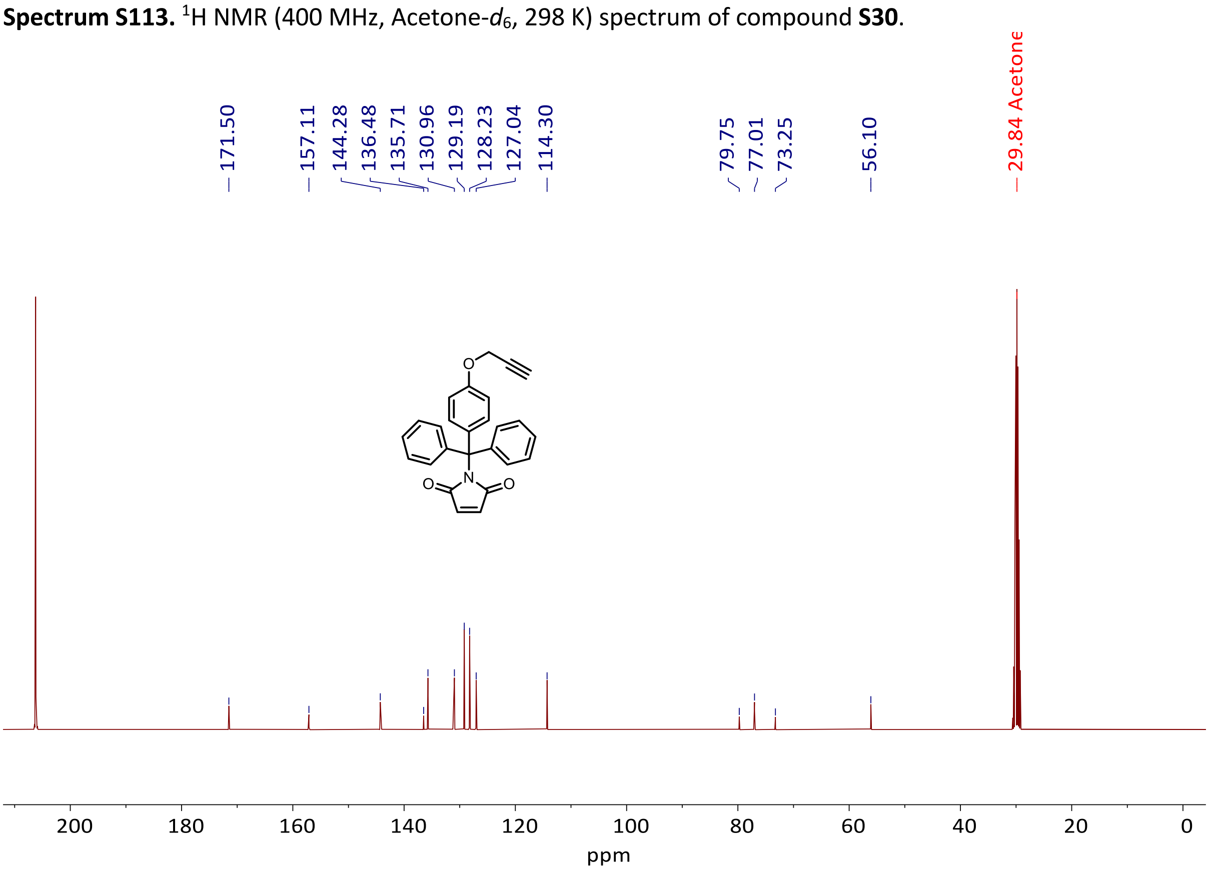

Spectrum S114. ^{13}C NMR (101 MHz, Acetone-d_6, 298 K) spectrum of compound **S30**.

9.1.51 Spectra of S31

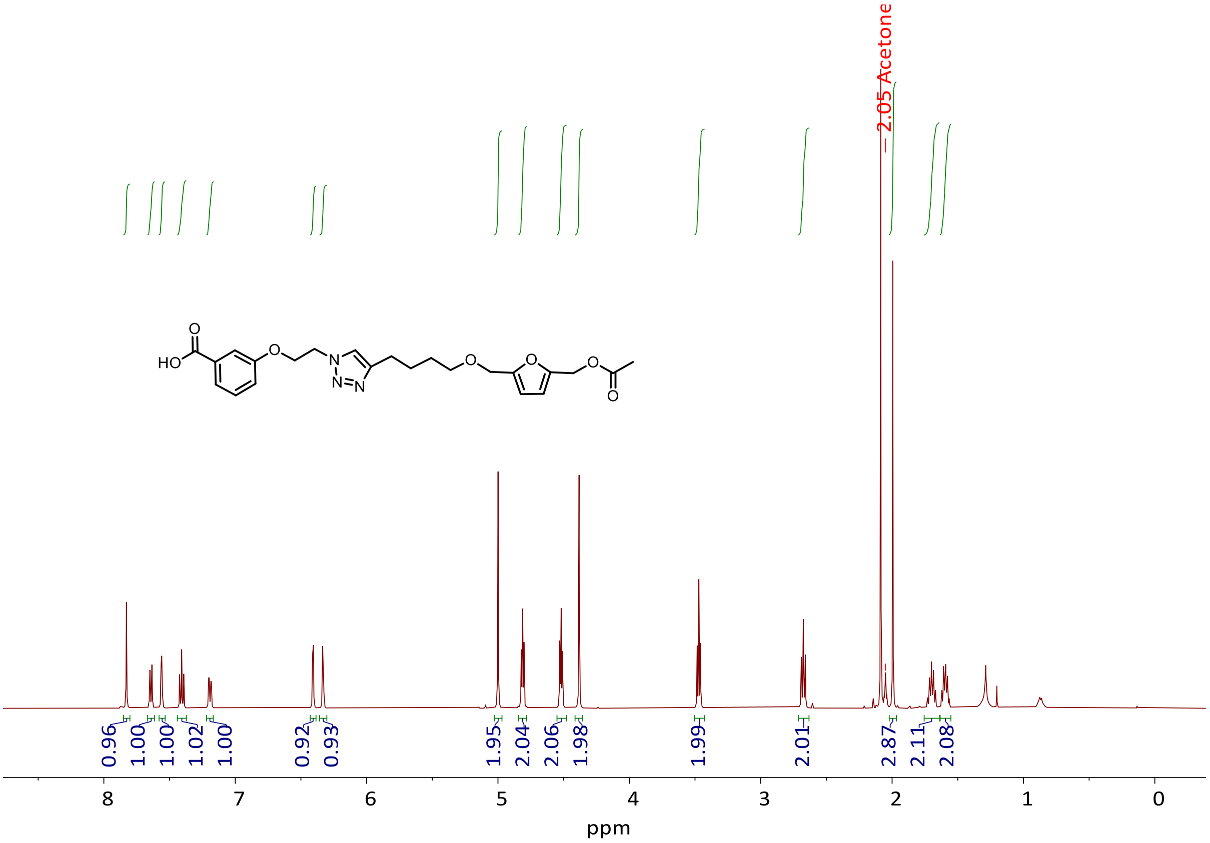

Spectrum S115. ^{1}H NMR (500 MHz, Acetone-d_6, 298 K) spectrum of compound **S31**.

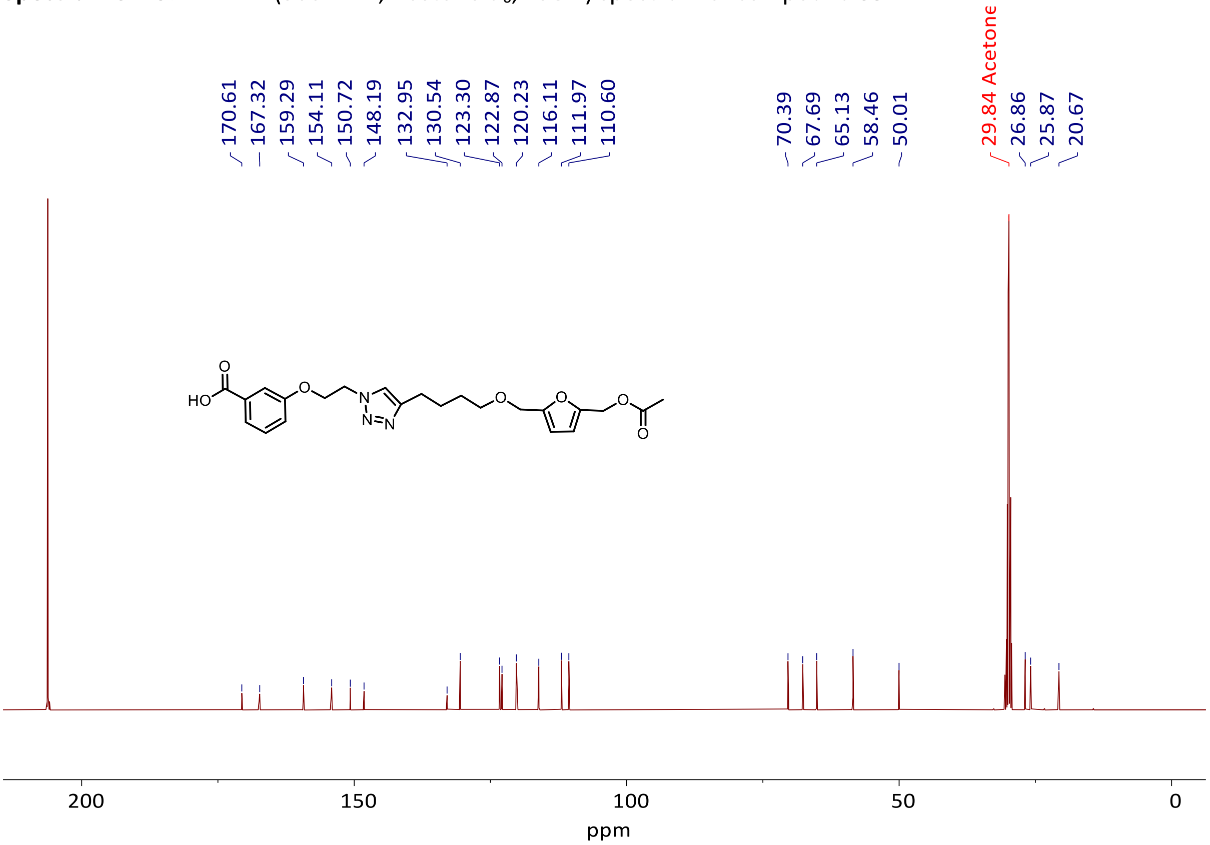

Spectrum S116. ^{13}C NMR (126 MHz, Acetone-d_6, 298 K) spectrum of compound **S31**.

9.1.52 Spectra of S32

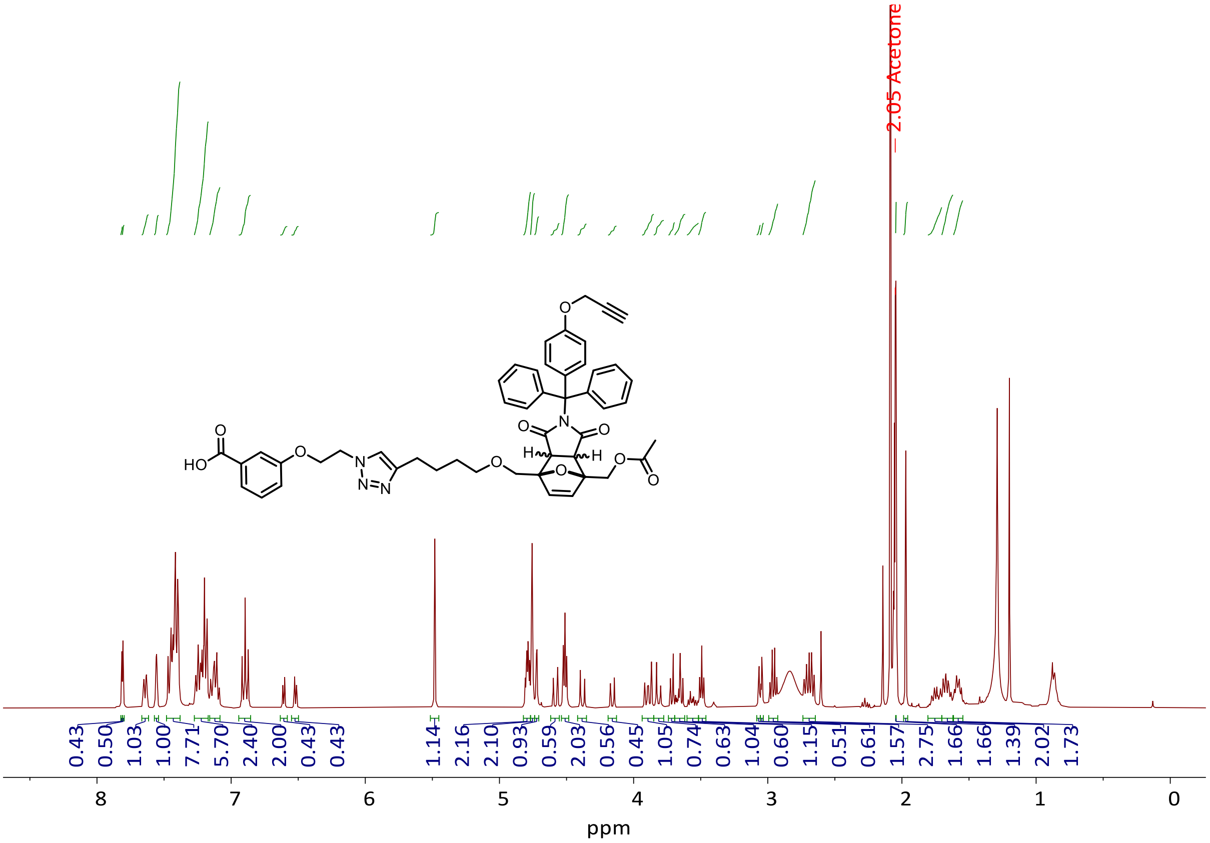

Spectrum S117. ^{1}H NMR (400 MHz, Acetone-d_6, 298 K) spectrum of compound **S32**.

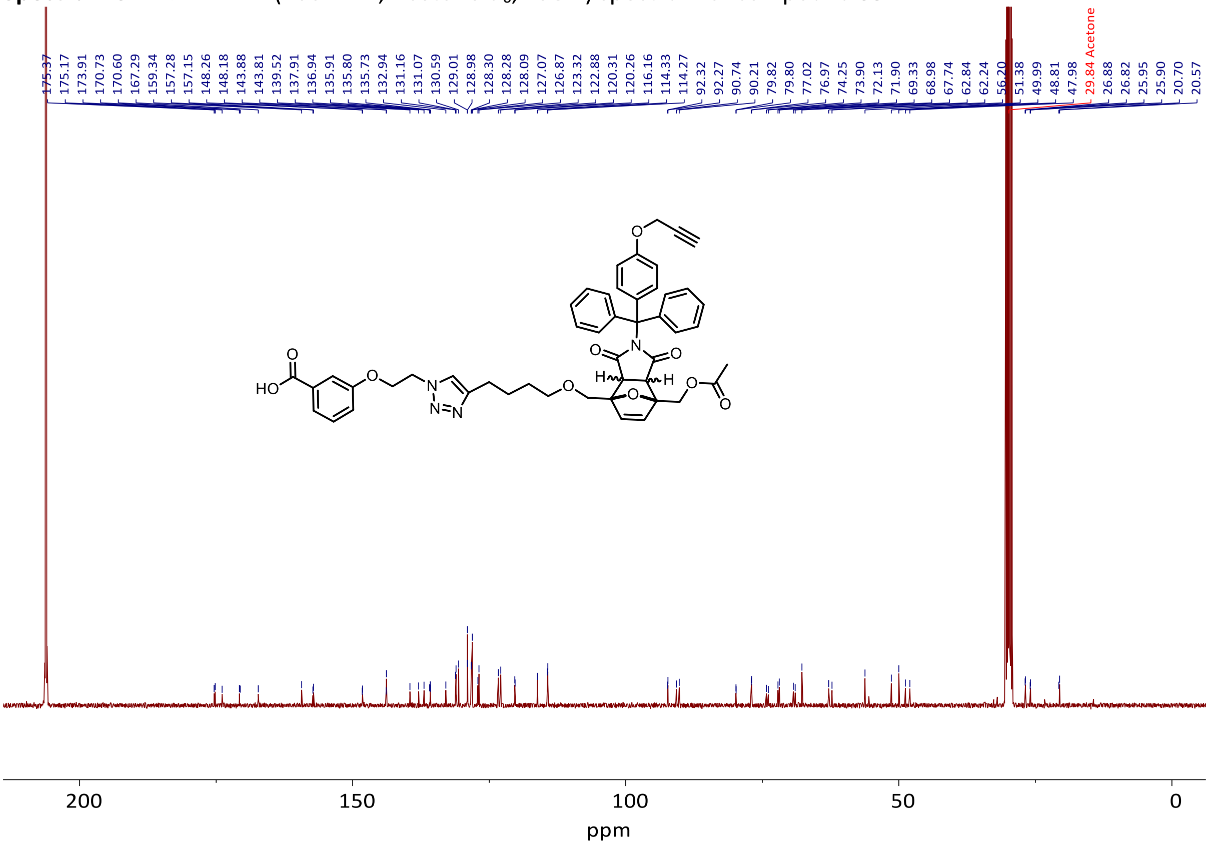

Spectrum S118. ^{13}C NMR (101 MHz, Acetone-d_6, 298 K) spectrum of compound **S32**.

9.1.53 Spectra of S33

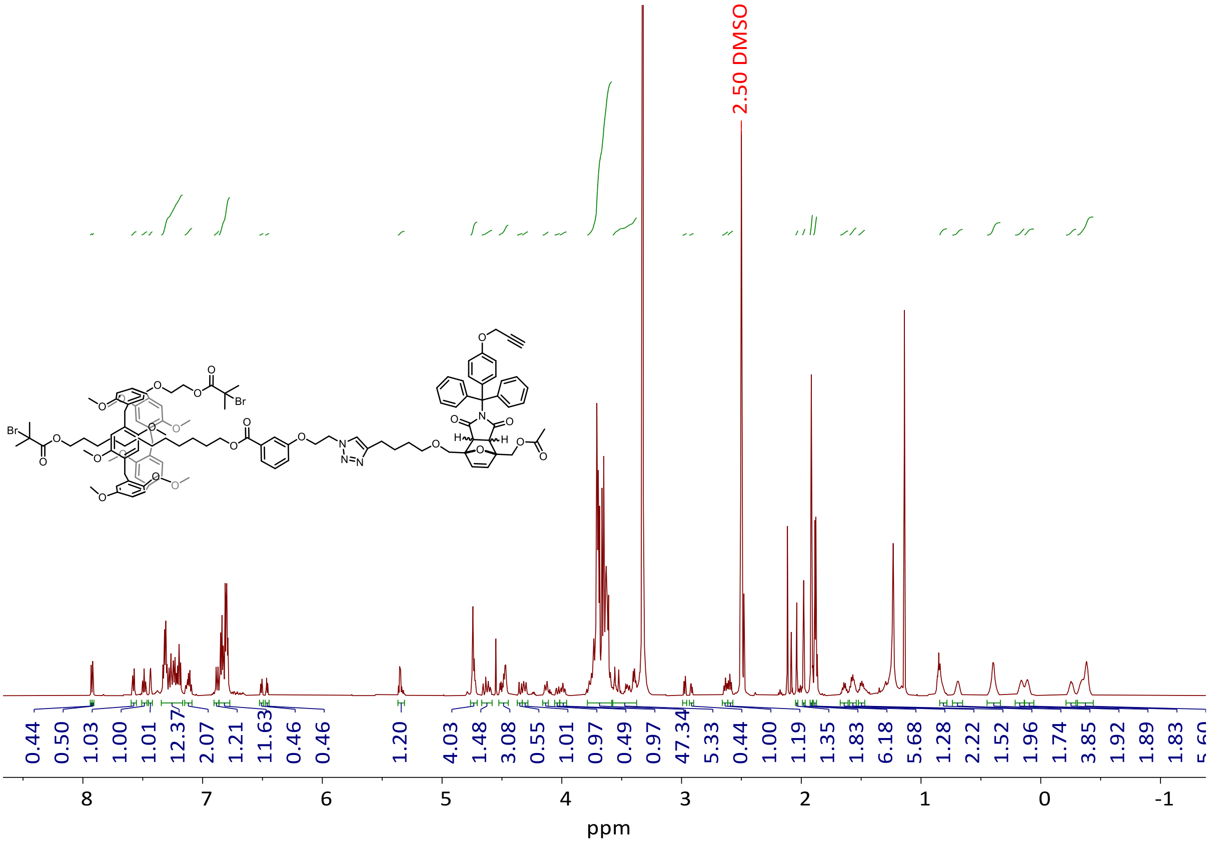

Spectrum S119. ^{1}H NMR (600 MHz, DMSO-d_6, 298 K) spectrum of compound **S33**.

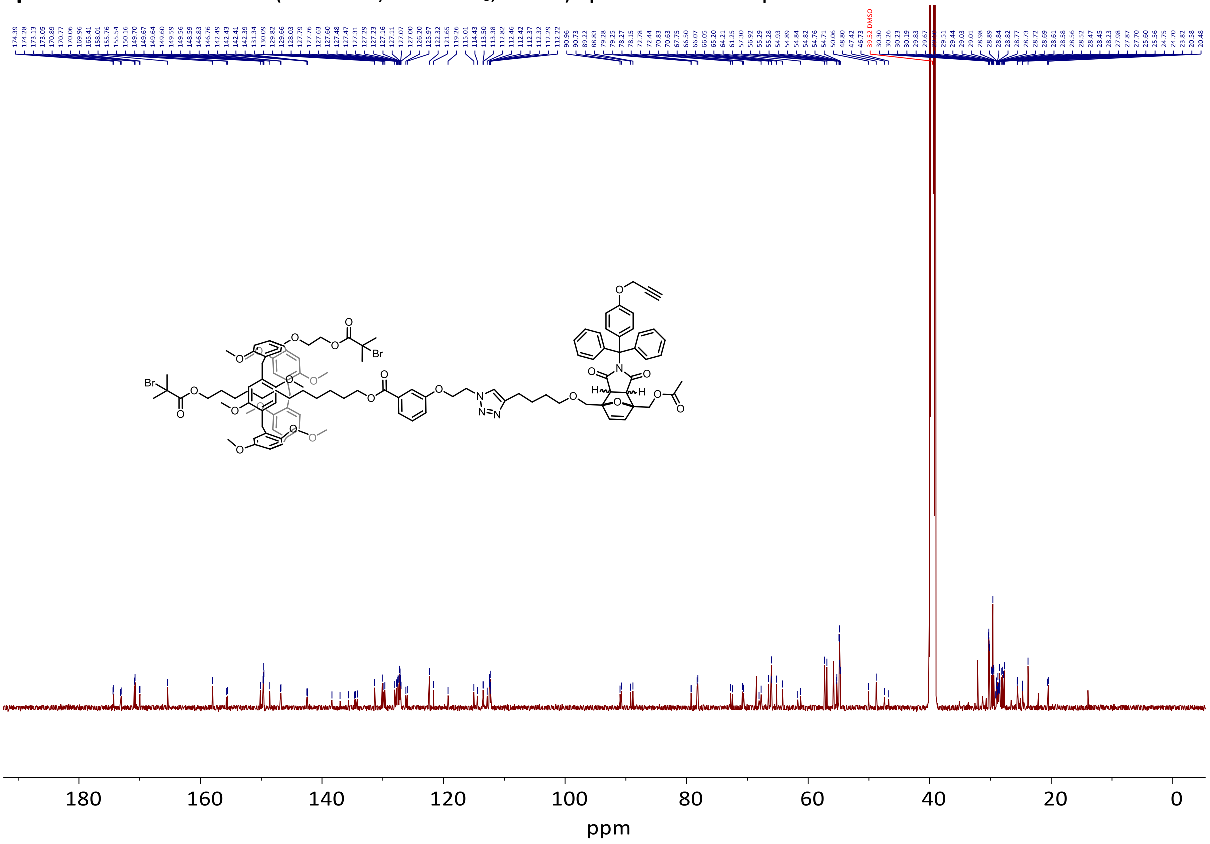

Spectrum S120. ^{13}C NMR (151 MHz, DMSO-d_6, 298 K) spectrum of compound **S33**.

9.1.54 Spectra of S35

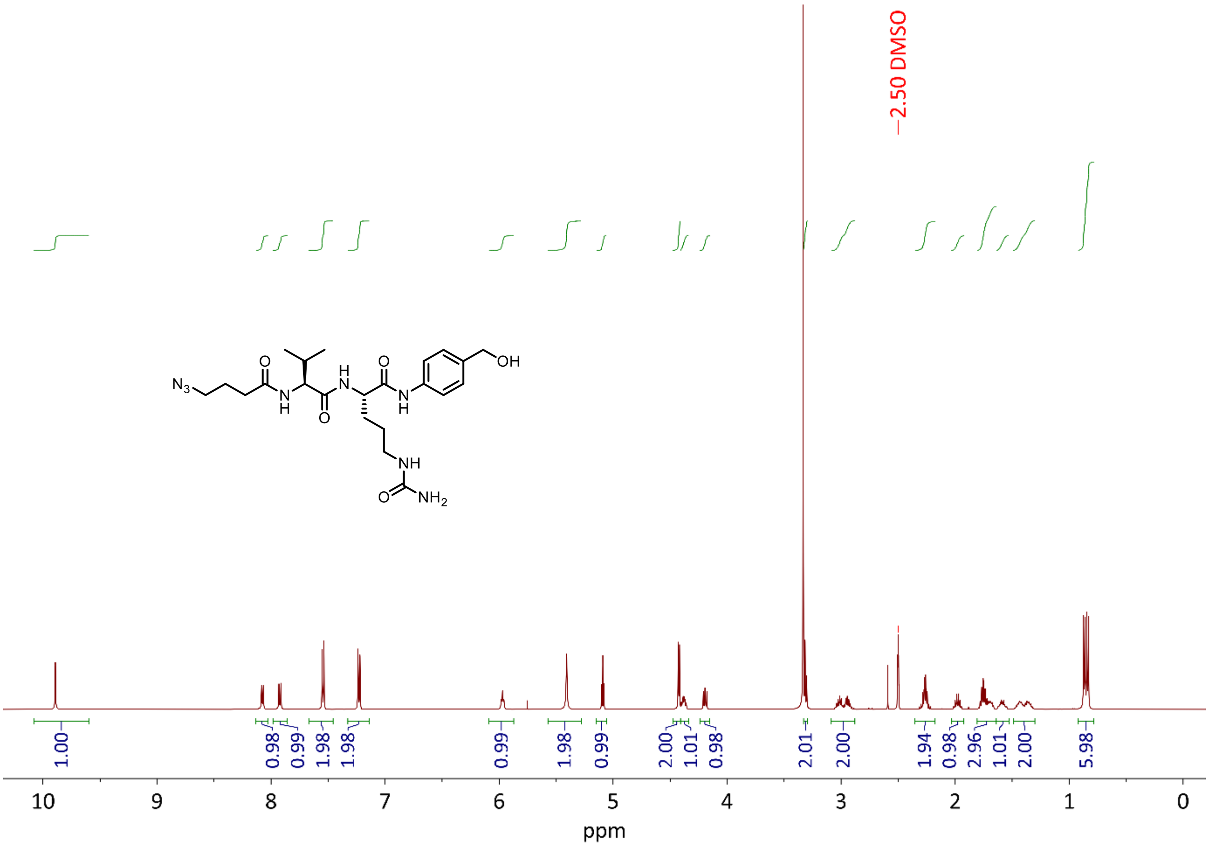

Spectrum S121. ^{1}H NMR (500 MHz, DMSO-d_6, 298 K) spectrum of compound **S35**.

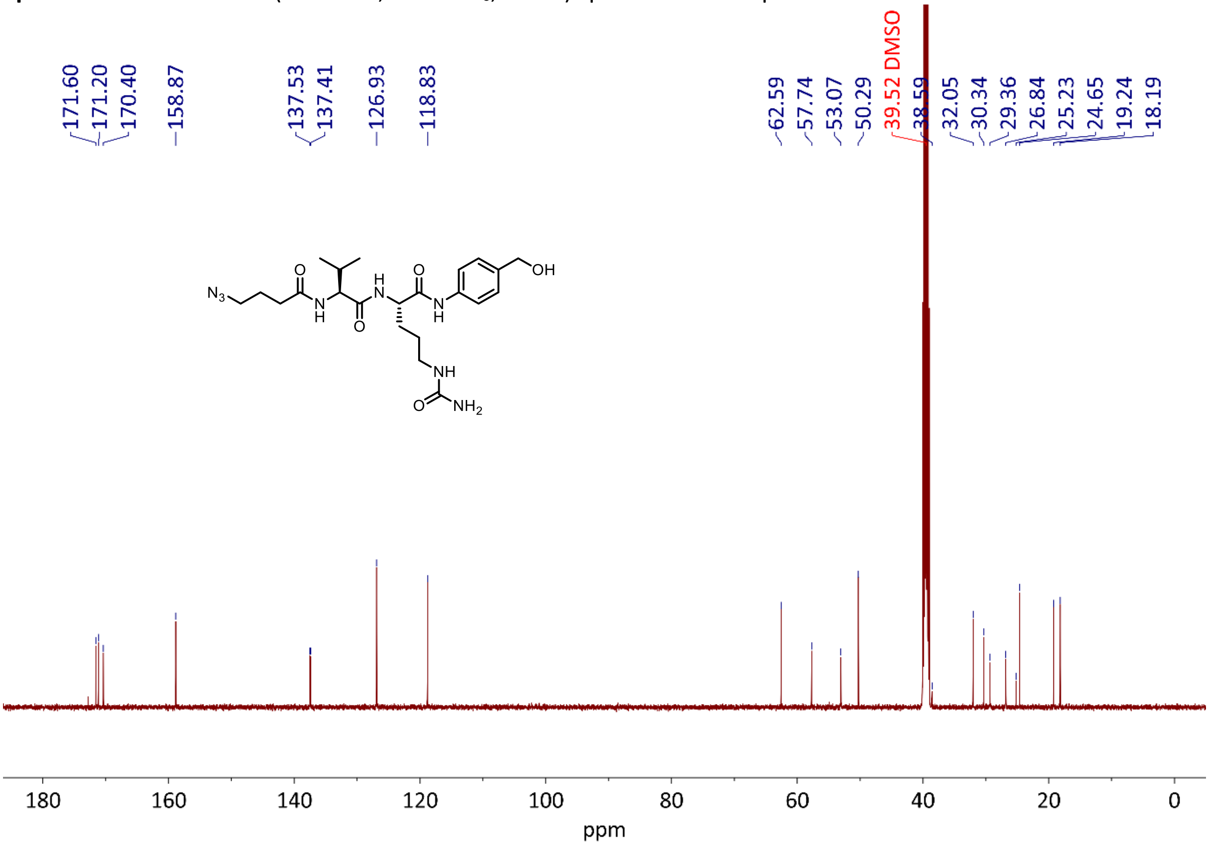

Spectrum S122. ^{13}C NMR (126 MHz, DMSO-d_6, 298 K) spectrum of compound **S35**.

9.1.55 Spectra of S36

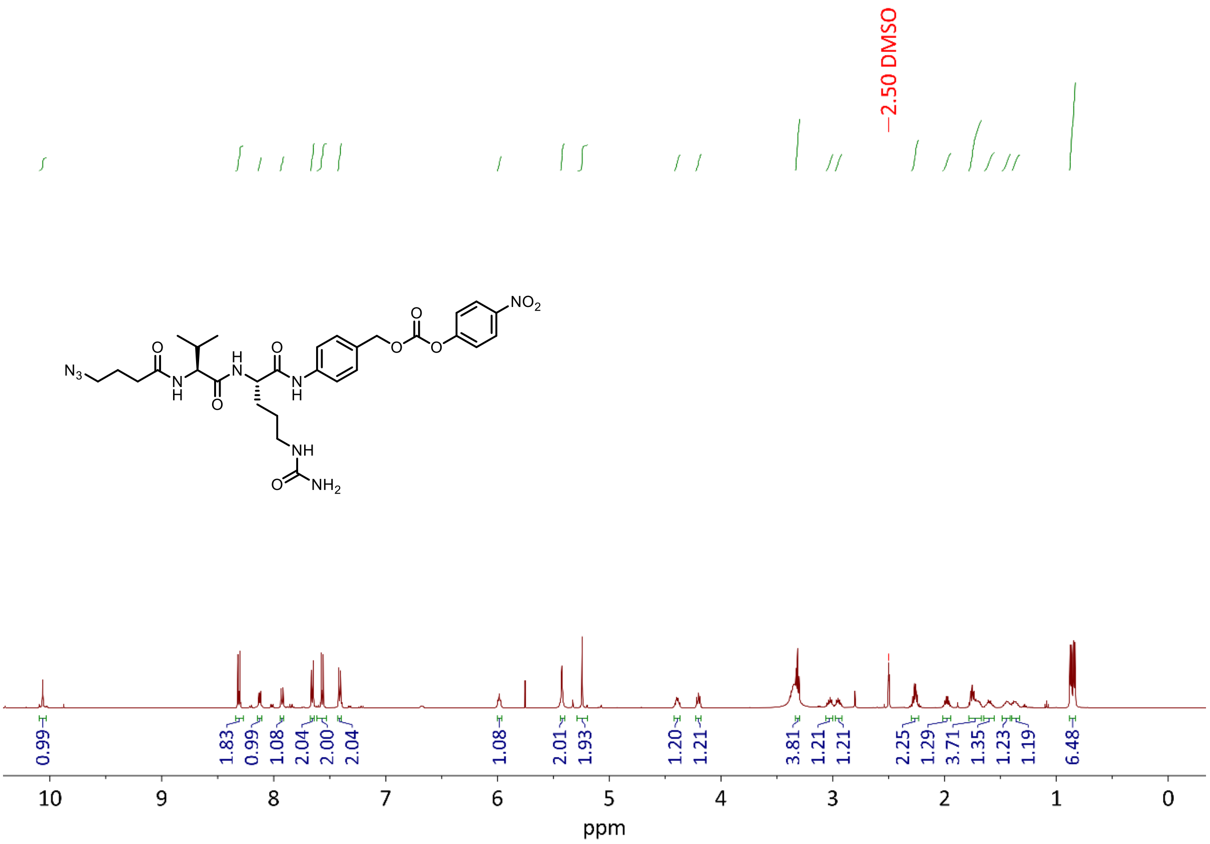

Spectrum S123. ^{1}H NMR (500 MHz, DMSO-d_6, 298 K) spectrum of compound **S36**.

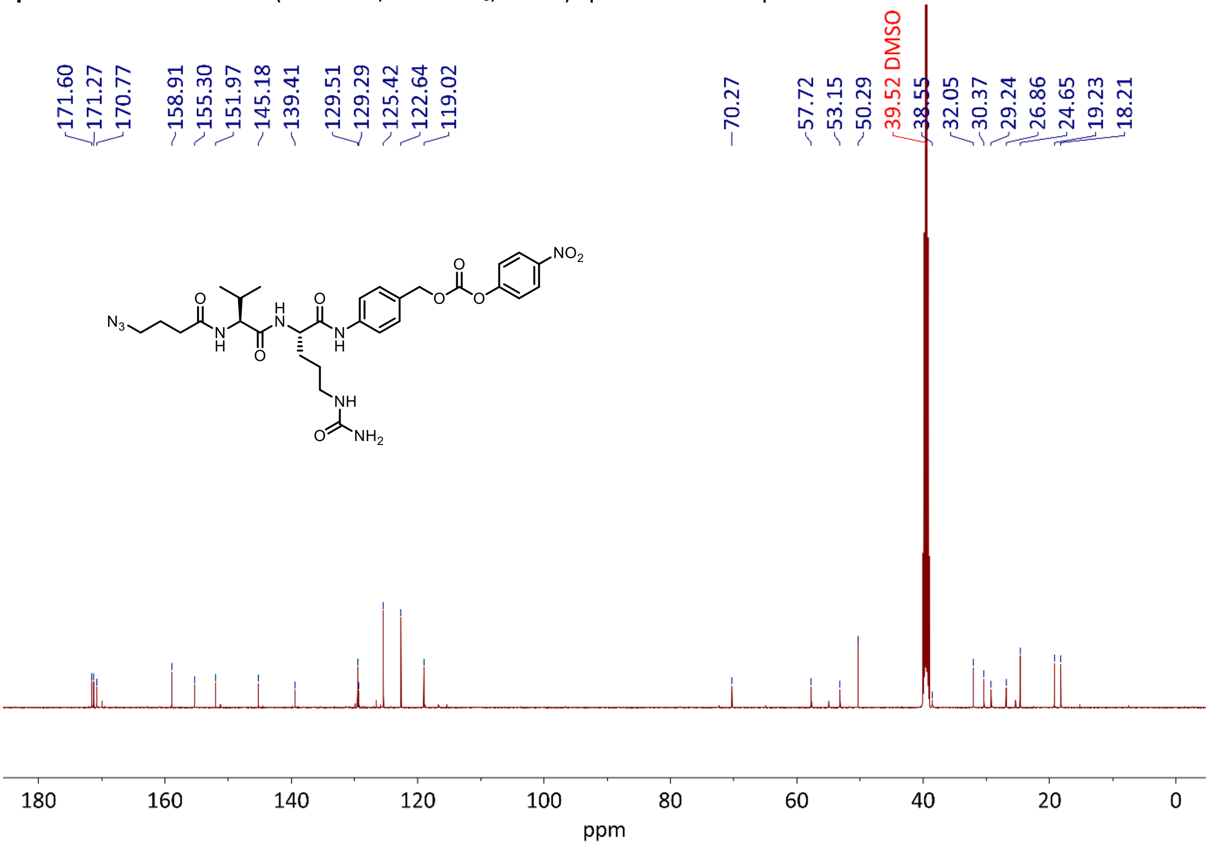

Spectrum S124. ^{13}C NMR (126 MHz, DMSO-d_6, 298 K) spectrum of compound **S36**.

9.1.56 Spectra of S37

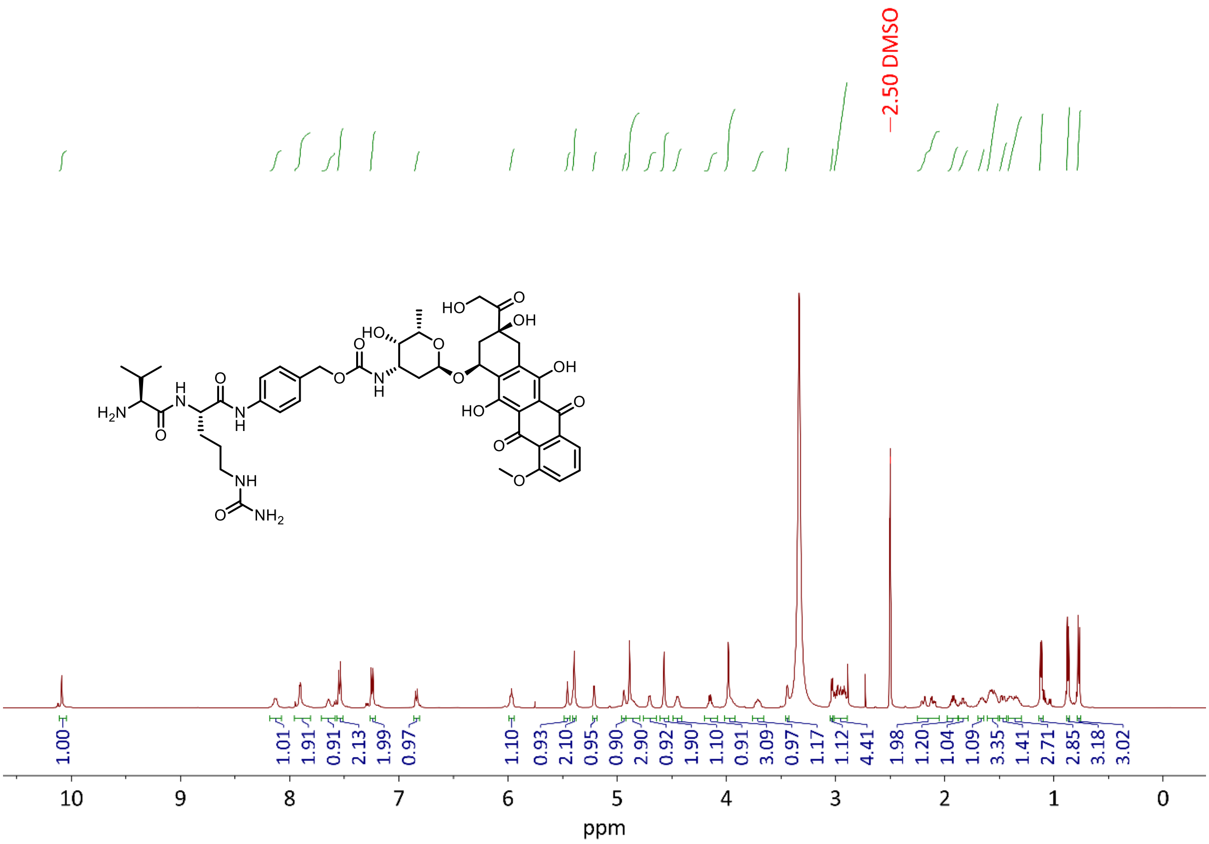

Spectrum S125. ^{1}H NMR (500 MHz, DMSO-d_6, 298 K) spectrum of compound **S37**.

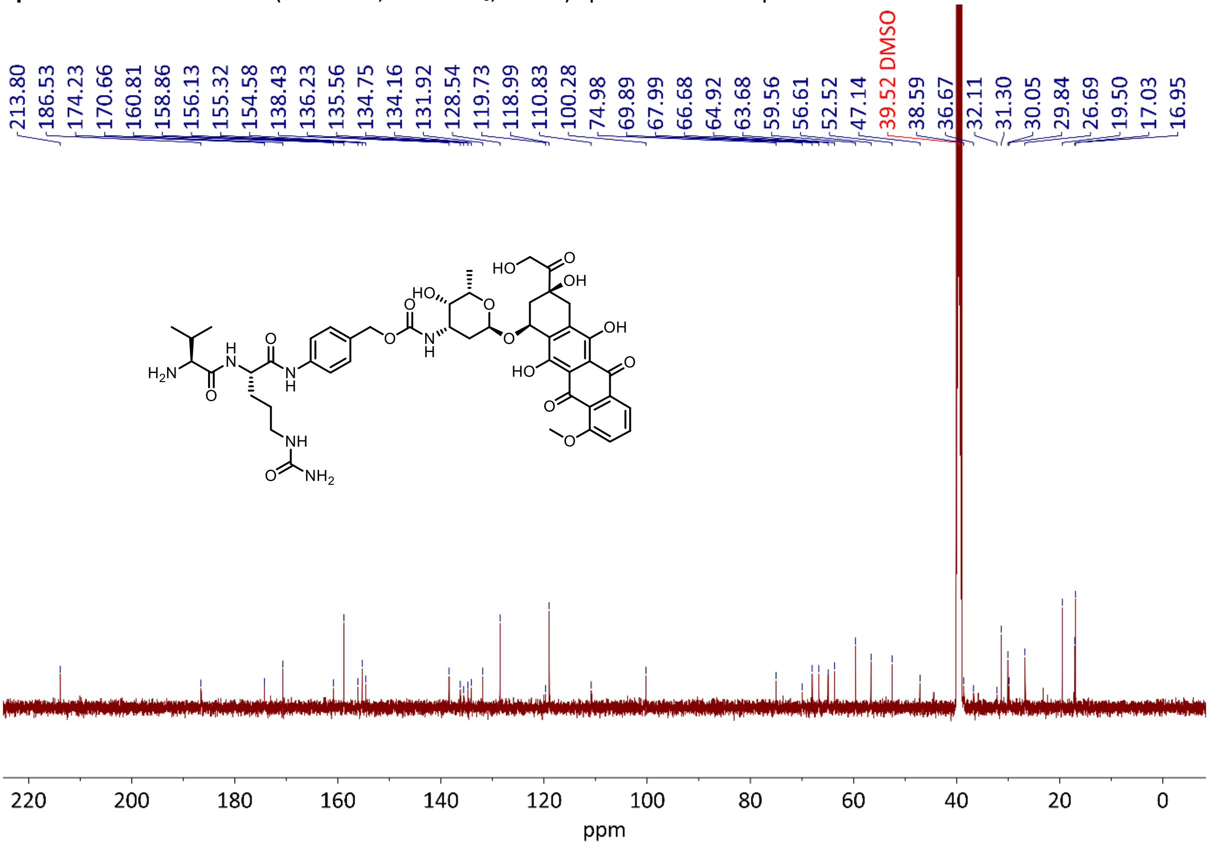

Spectrum S126. ^{13}C NMR (126 MHz, DMSO-d_6, 298 K) spectrum of compound **S37**.

9.1.57 Spectra of S38

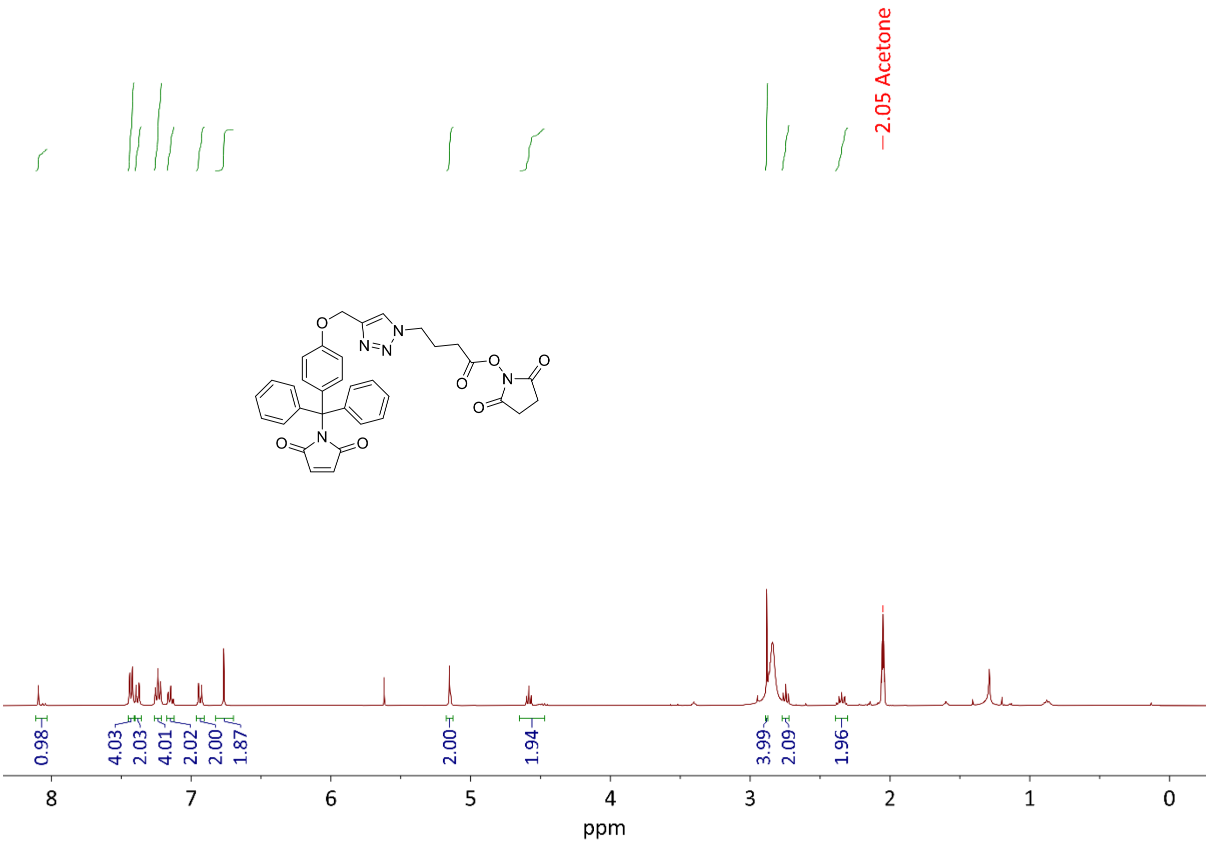

Spectrum S127. ^{1}H NMR (400 MHz, Acetone-d_6, 298 K) spectrum of compound **S38**.

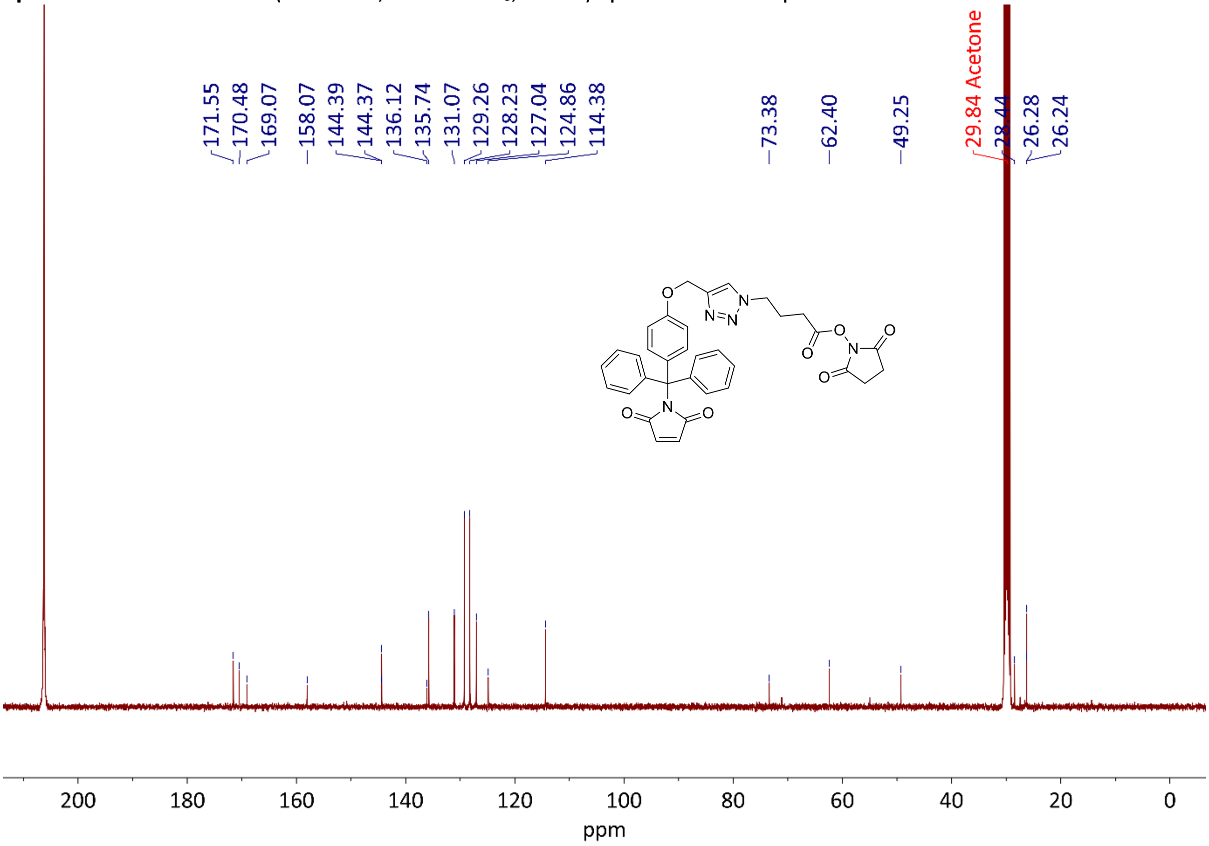

Spectrum S128. ^{13}C NMR (101 MHz, Acetone-d_6, 298 K) spectrum of compound **S38**.

9.1.58 Spectra of 12

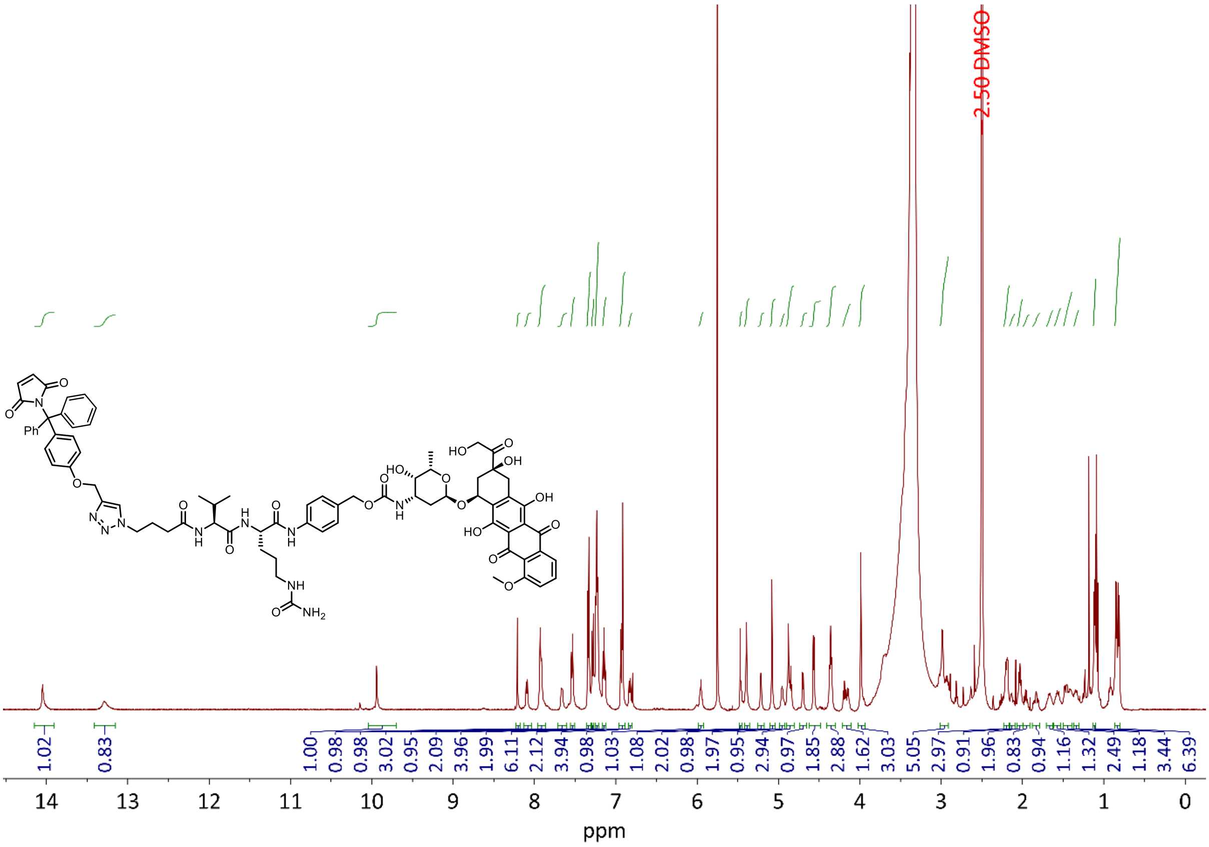

Spectrum S129. ^{1}H NMR (500 MHz, DMSO-d_6, 298 K) spectrum of compound **12**.

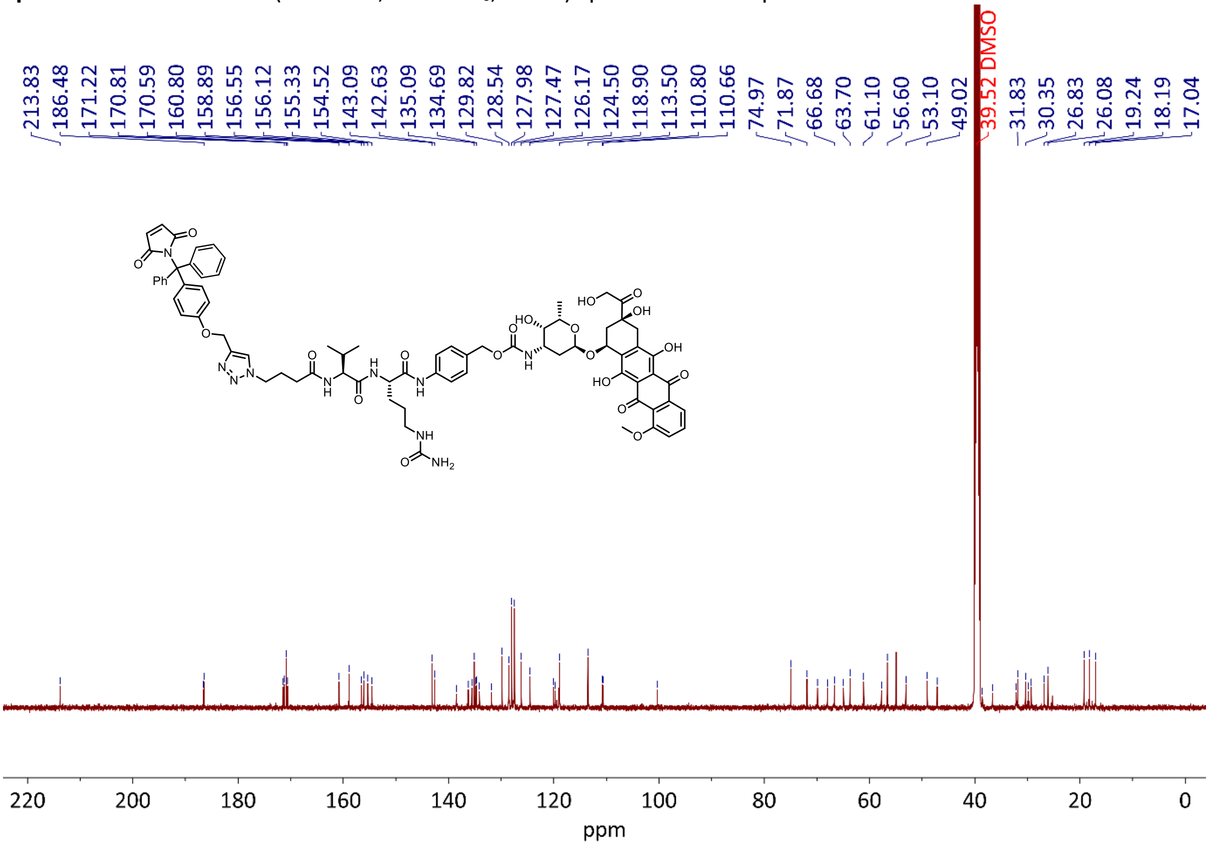

Spectrum S130. ^{13}C NMR (126 MHz, DMSO-d_6, 298 K) spectrum of compound **12**.

9.1.59 Spectra of S39

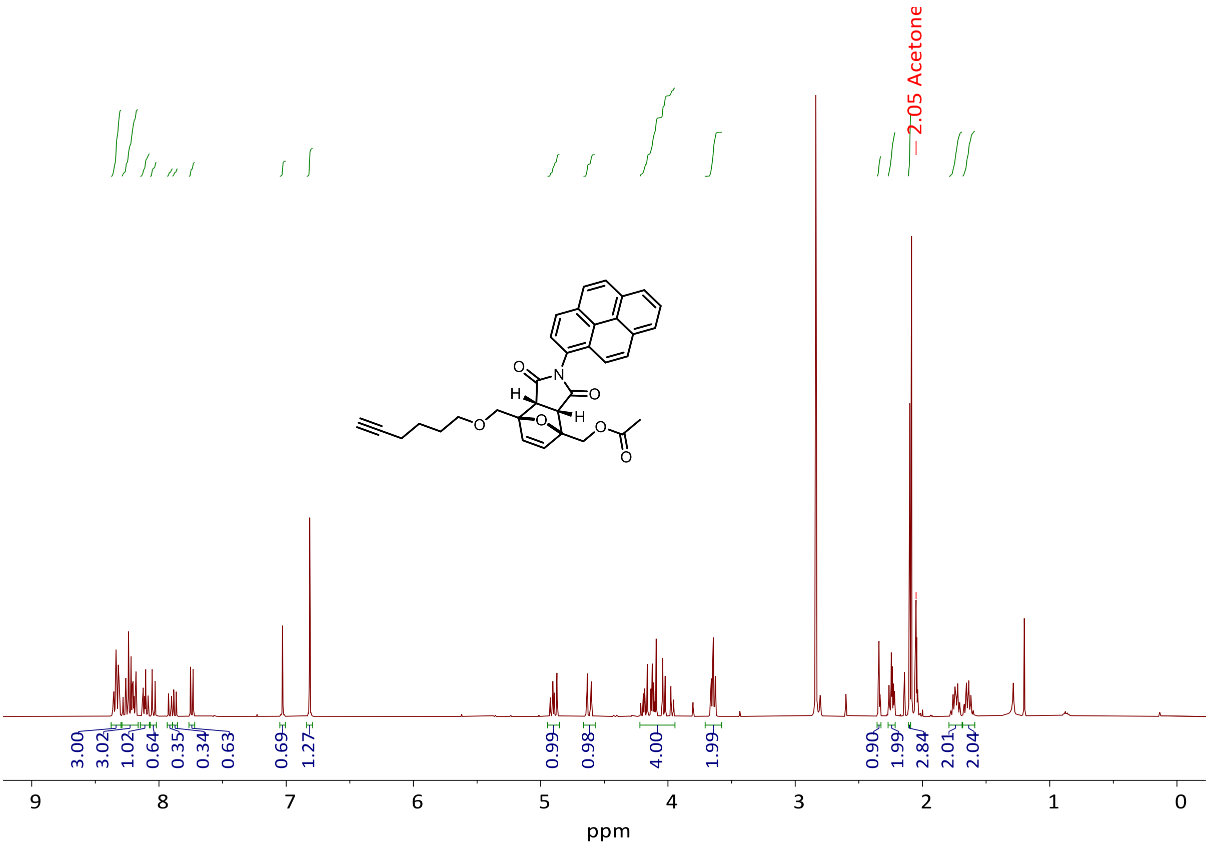

Spectrum S131. ^{1}H NMR (400 MHz, Acetone-d_6, 298 K) spectrum of compound **S39**.

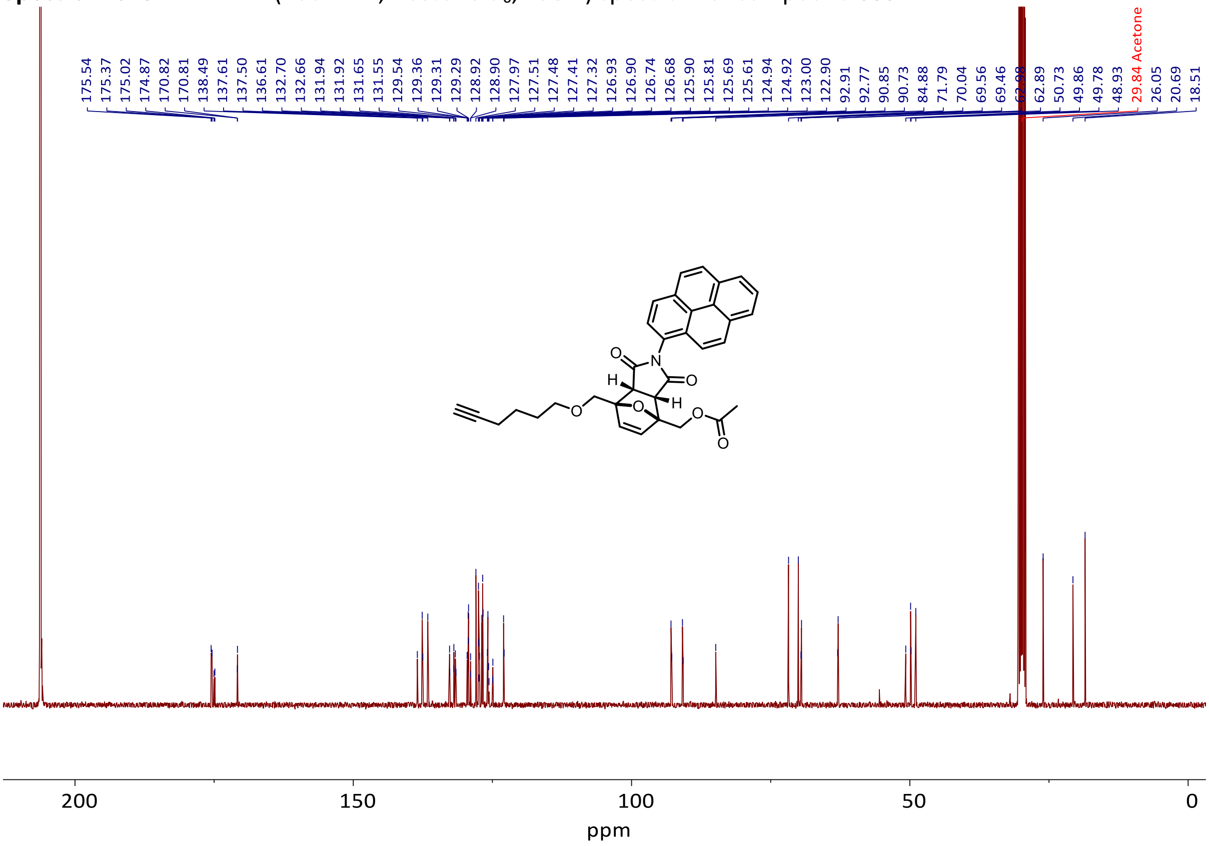

Spectrum S132. ^{13}C NMR (101 MHz, Acetone-d_6, 298 K) spectrum of compound **S39**.

9.1.60 Spectra of S40

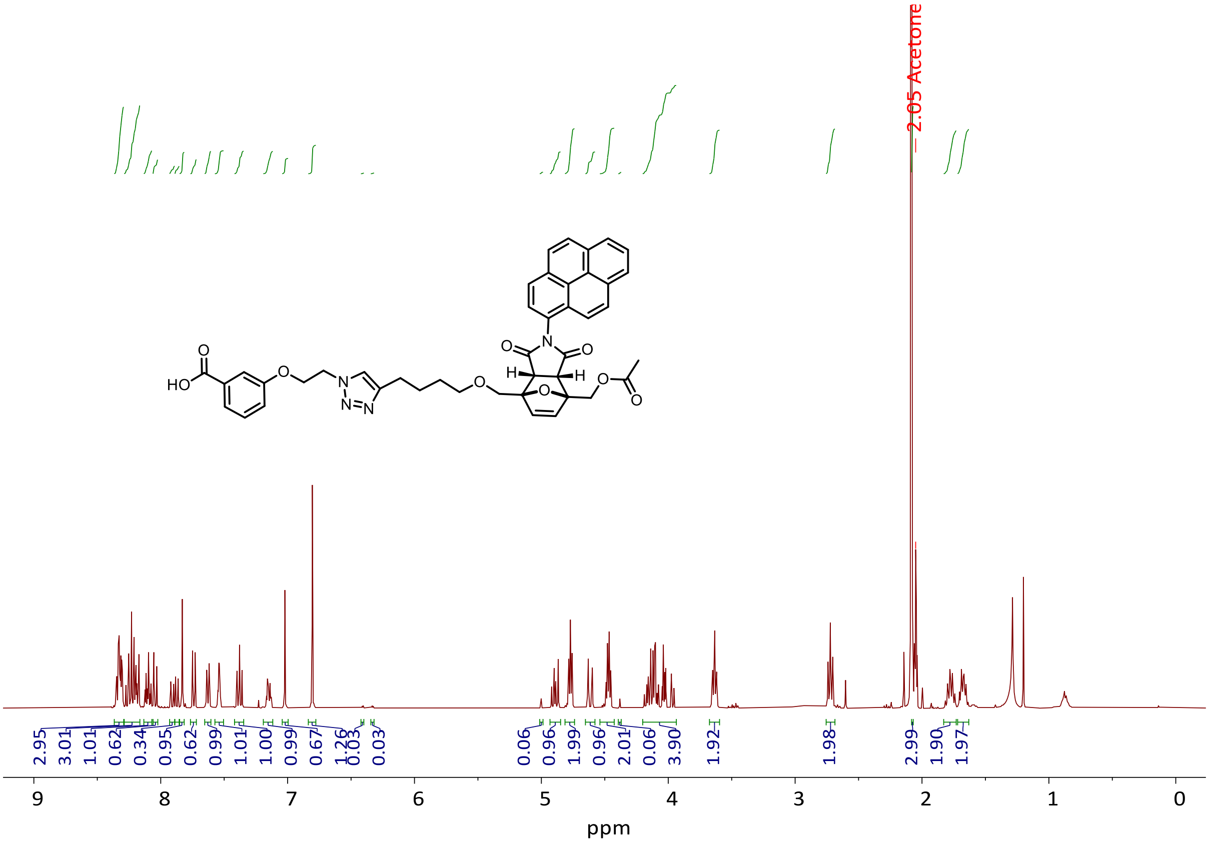

Spectrum S133. ^{1}H NMR (400 MHz, Acetone-d_6, 298 K) spectrum of compound **S40**.

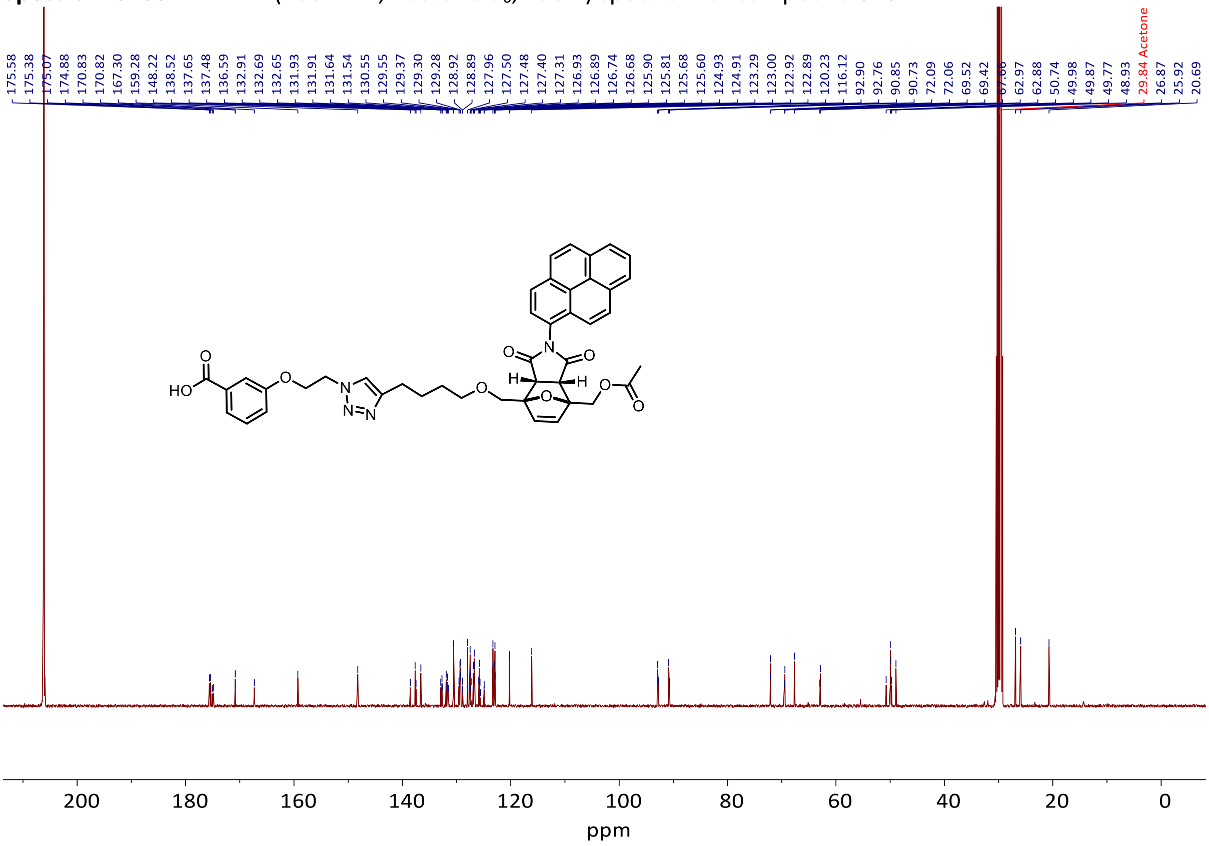

Spectrum S134. ^{13}C NMR (101 MHz, Acetone-d_6, 298 K) spectrum of compound **S40**.

9.1.61 Spectra of S41

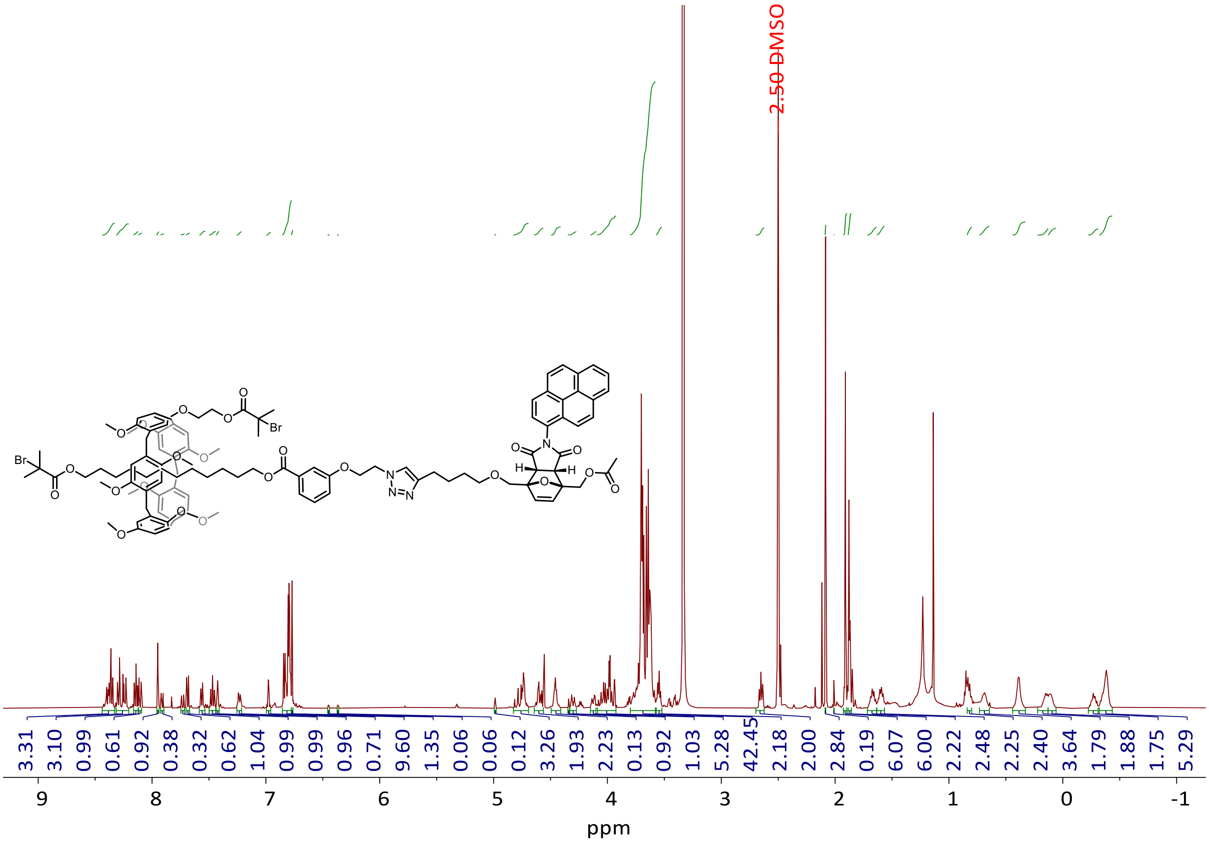

Spectrum S135. ^{1}H NMR (500 MHz, DMSO-d_6, 298 K) spectrum of compound **S41**.

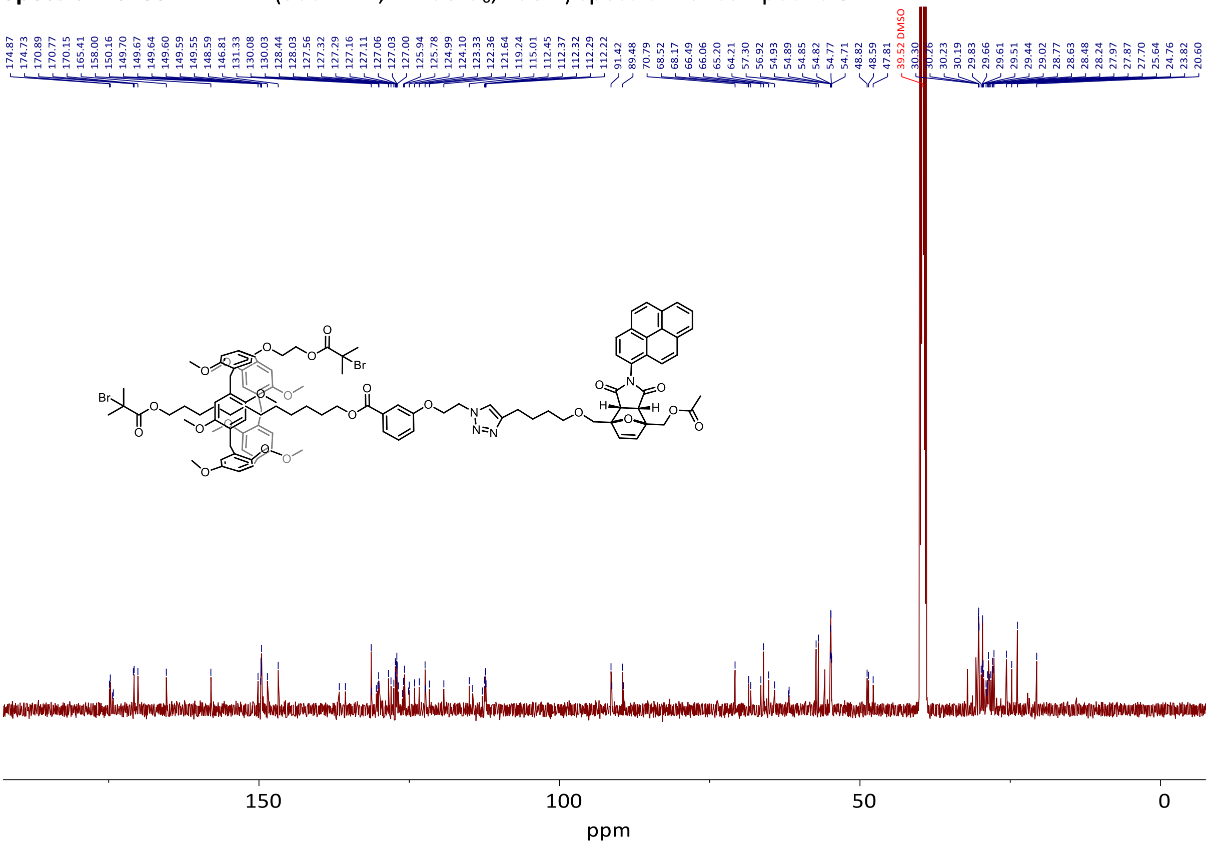

Spectrum S136. ^{13}C NMR (126 MHz, DMSO-d_6, 298 K) spectrum of compound **S41**.

9.1.62 Spectra of S42

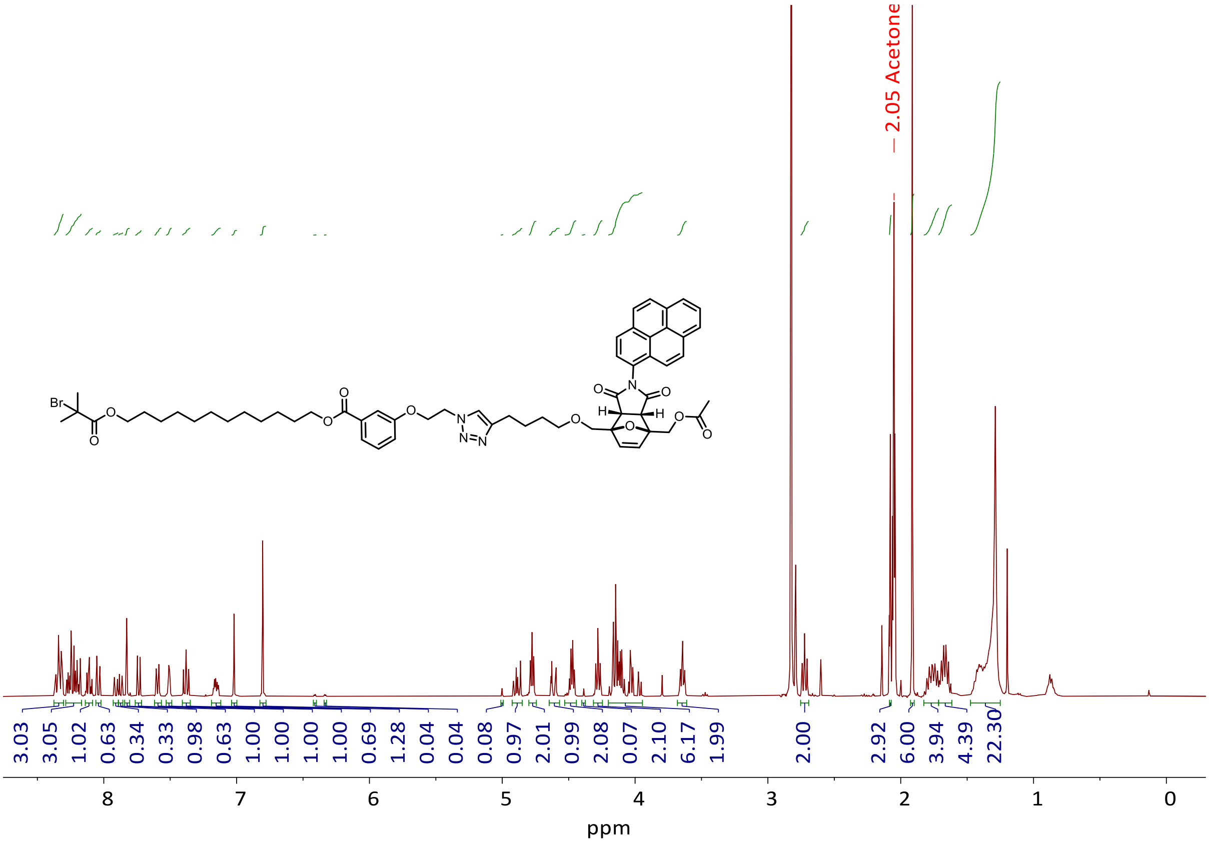

Spectrum S137. ^{1}H NMR (400 MHz, Acetone-d_6, 298 K) spectrum of compound **S42**.

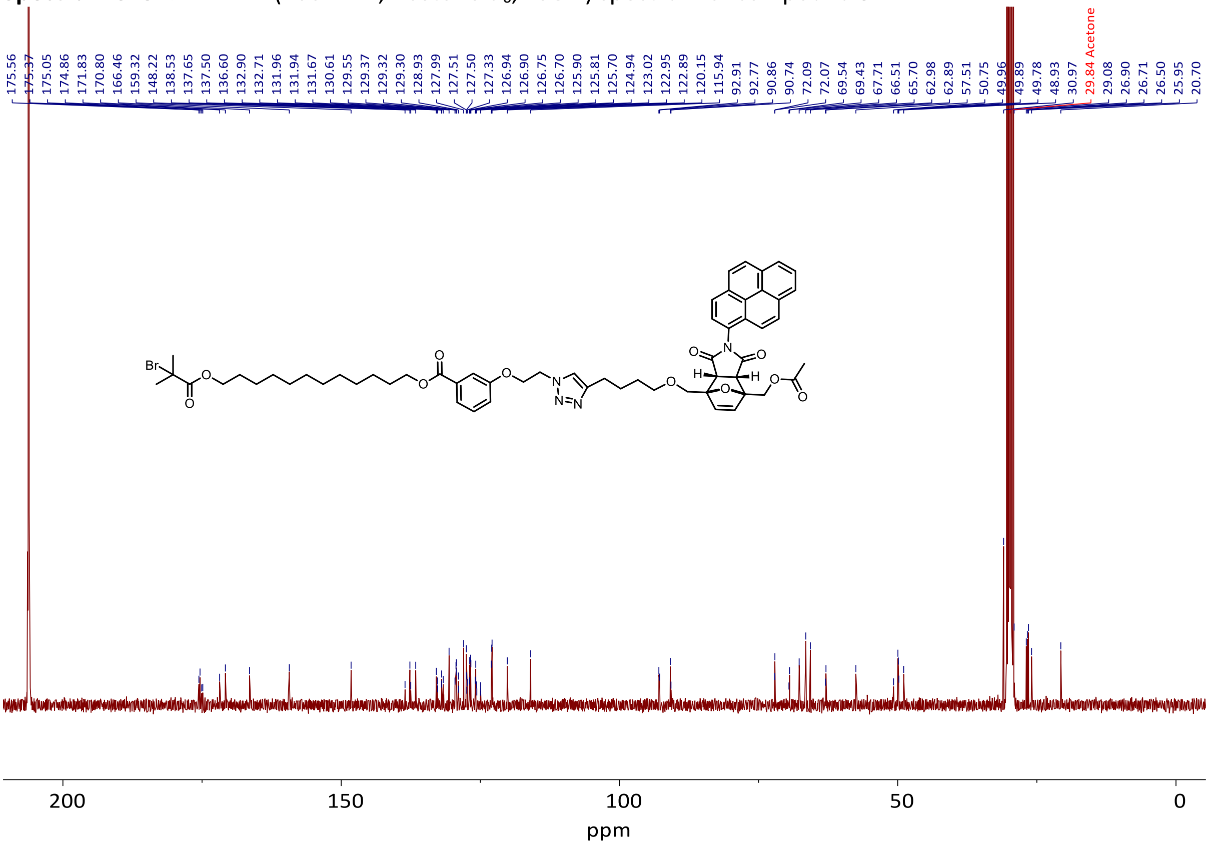

Spectrum S138. ^{13}C NMR (101 MHz, Acetone-d_6, 298 K) spectrum of compound **S42**.

9.1.63 Spectra of S43

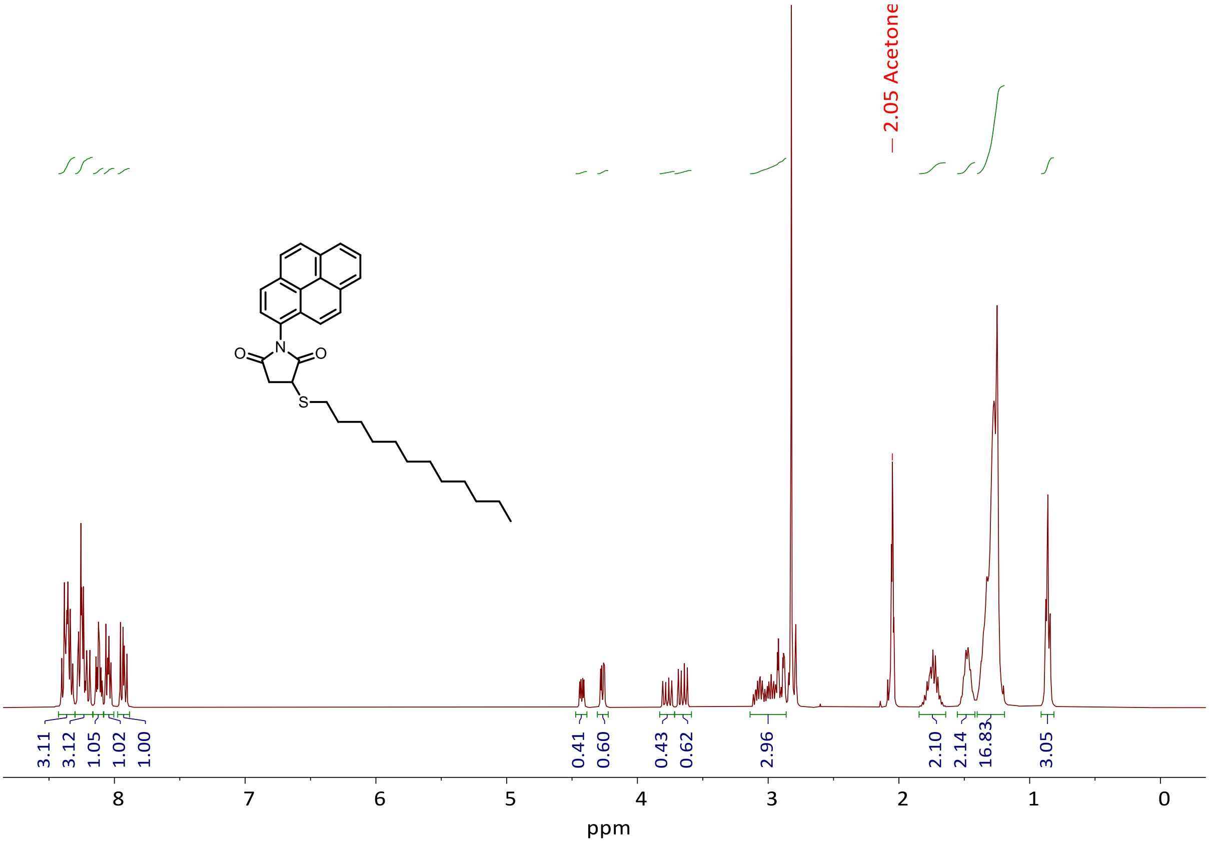

Spectrum S139. ^{1}H NMR (400 MHz, Acetone-d_6, 298 K) spectrum of compound **S43**.

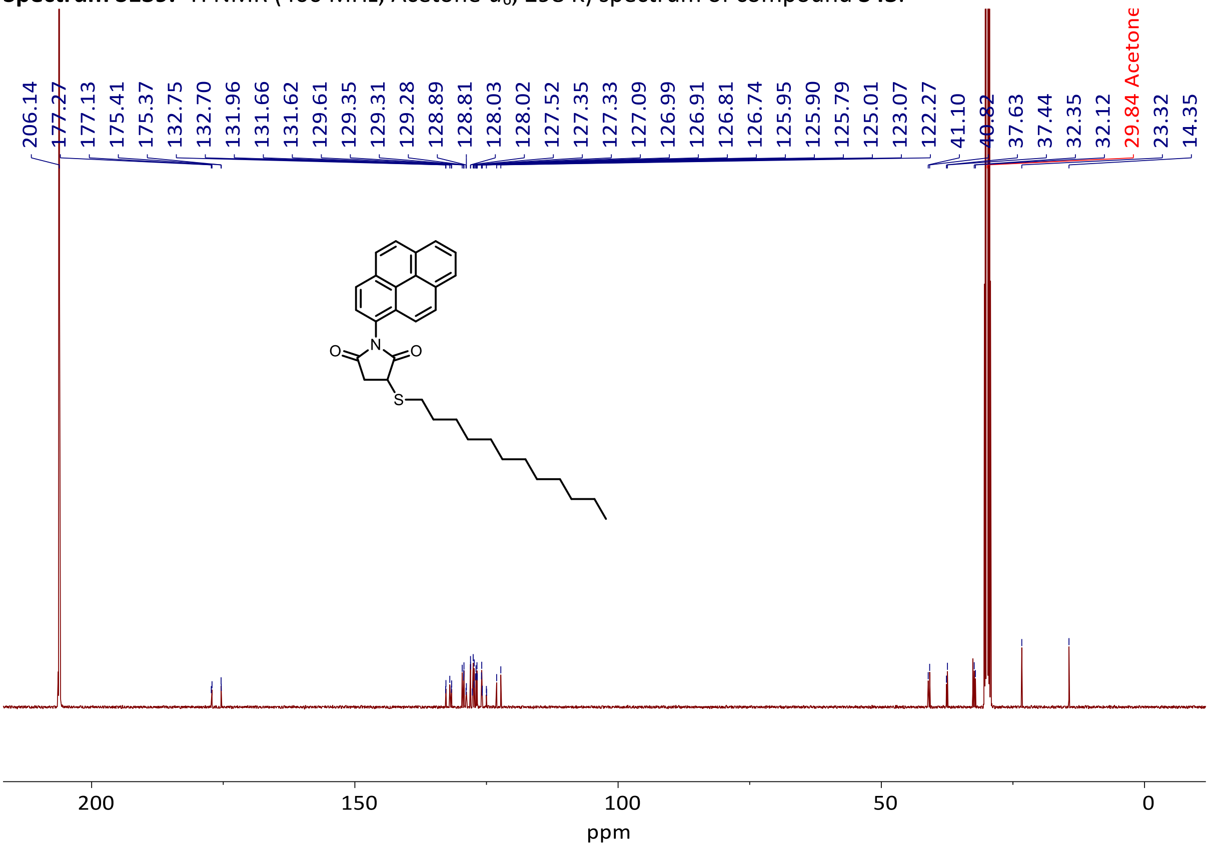

Spectrum S140. ^{13}C NMR (101 MHz, Acetone-d_6, 298 K) spectrum of compound **S43**.

9.1.64 Spectra of S44

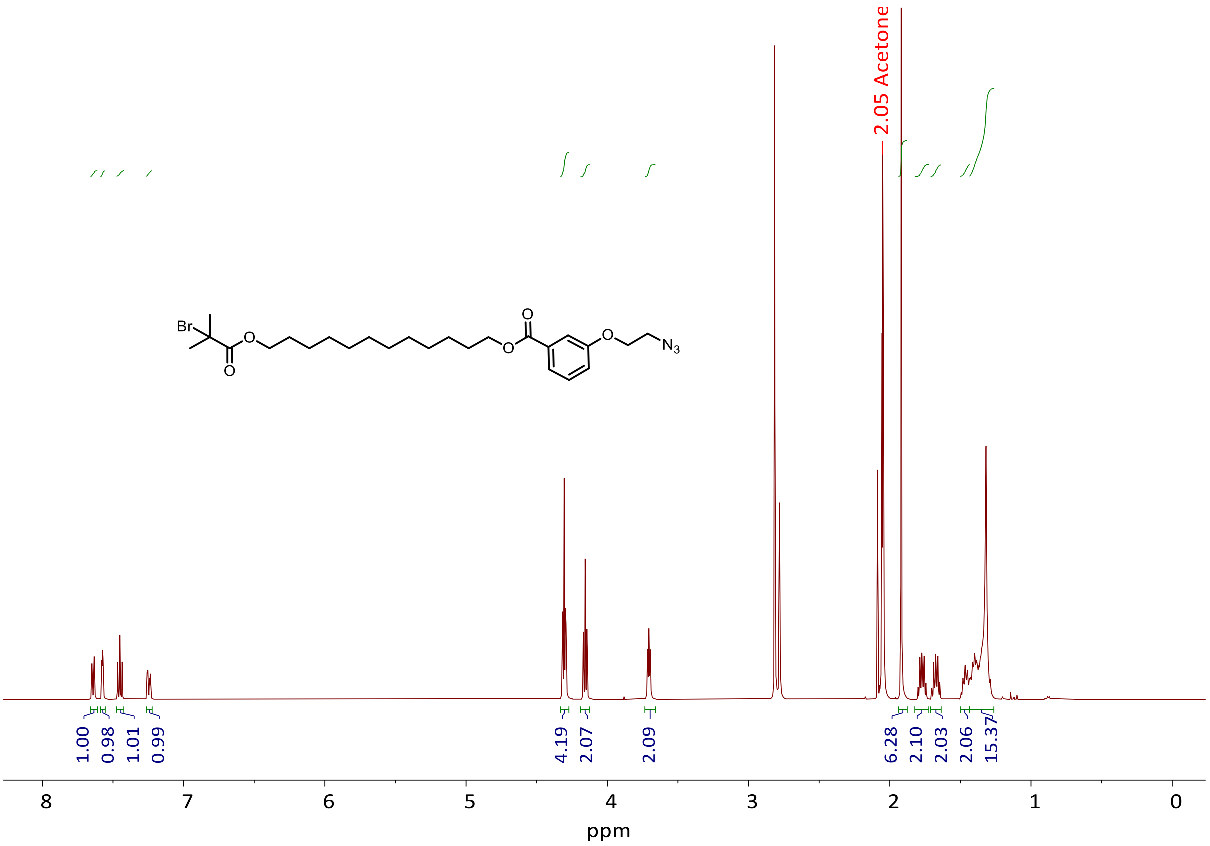

Spectrum S141. ^{1}H NMR (500 MHz, Acetone-d_6, 298 K) spectrum of compound **S44**.

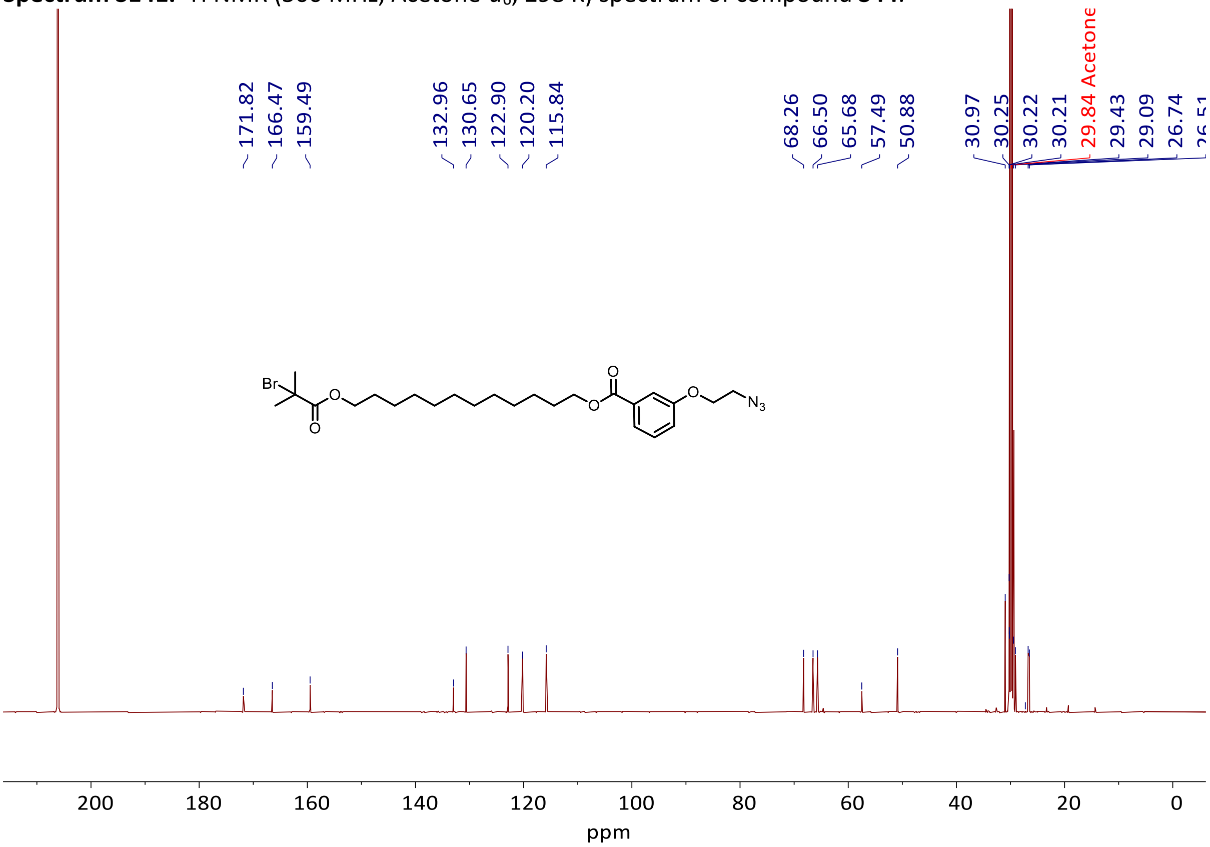

Spectrum S142. ^{13}C NMR (126 MHz, Acetone-d_6, 298 K) spectrum of compound **S44**.

9.1.65 Spectra of S45

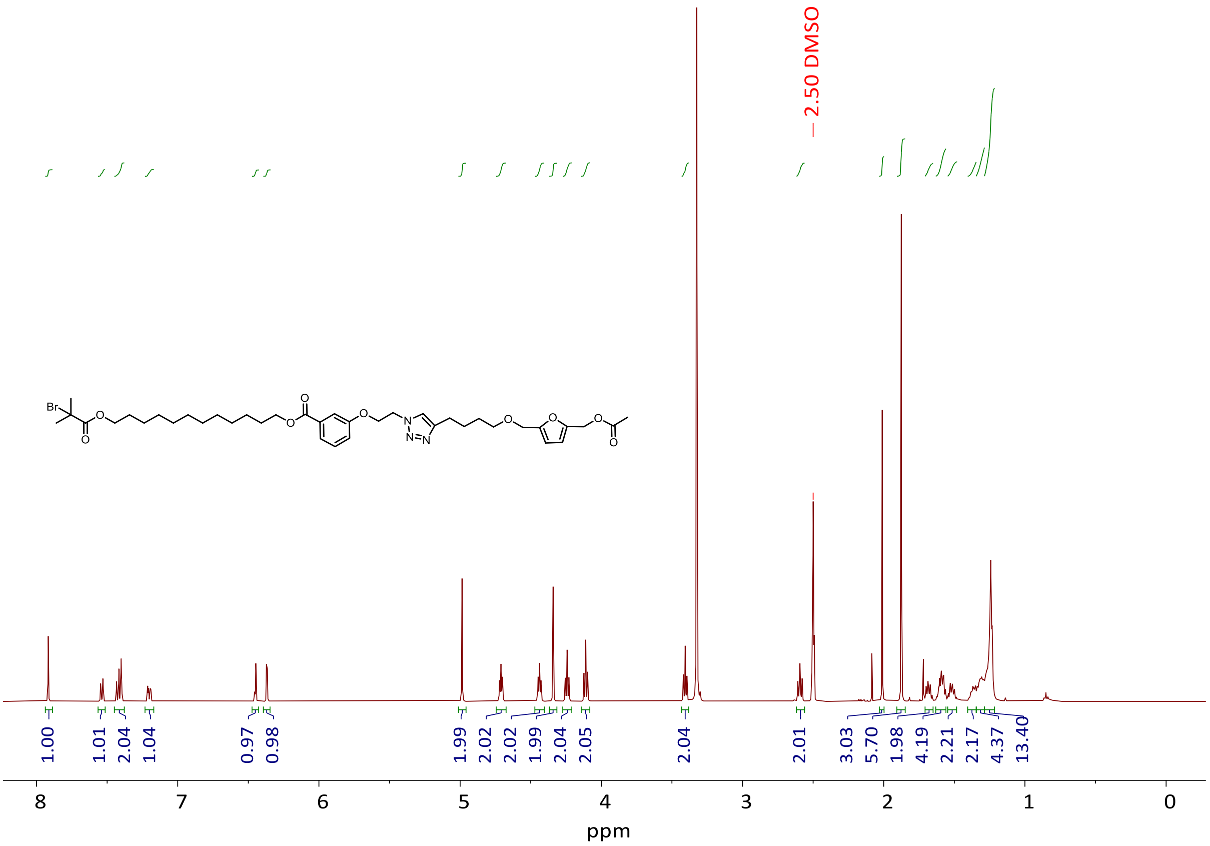

Spectrum S143. ^{1}H NMR (500 MHz, DMSO-d_6, 298 K) spectrum of compound **S45**.

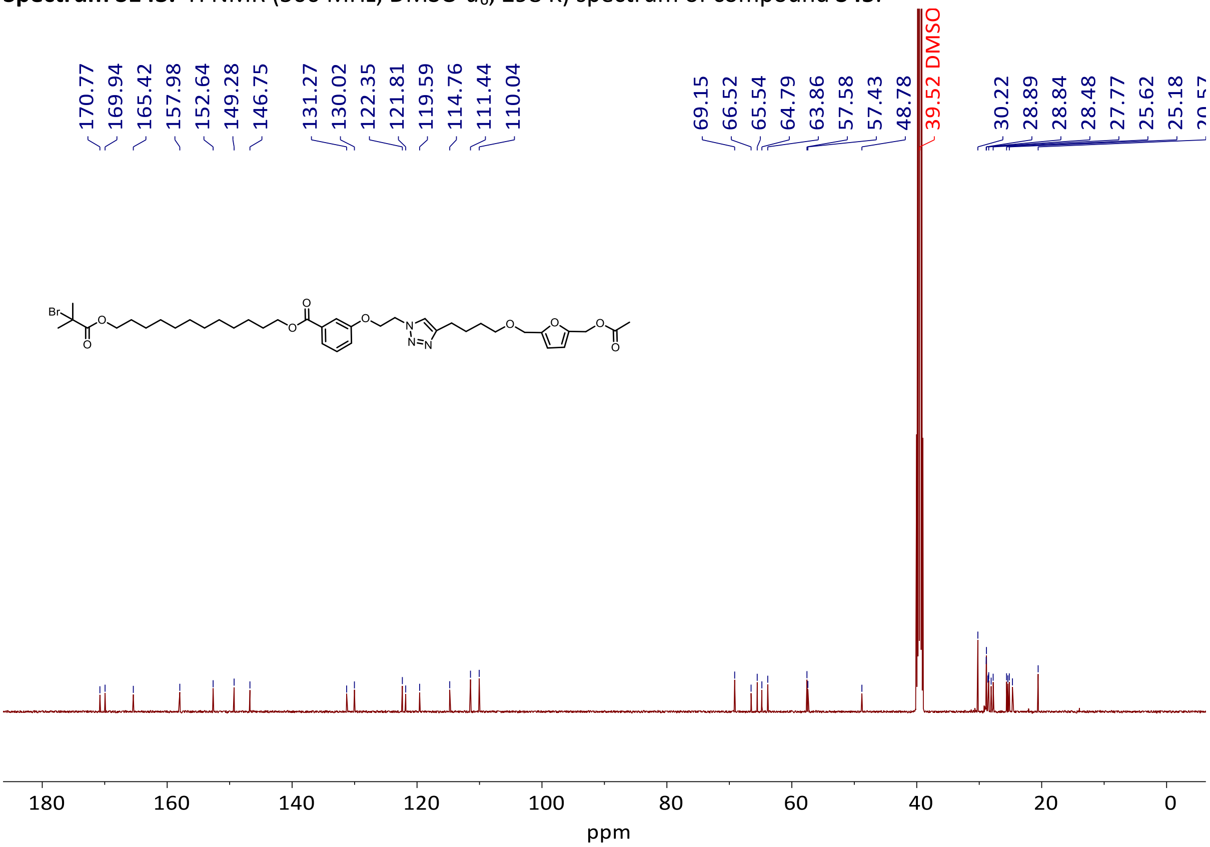

Spectrum S144. ^{13}C NMR (126 MHz, DMSO-d_6, 298 K) spectrum of compound **S45**.

9.1.66 Spectra of S46

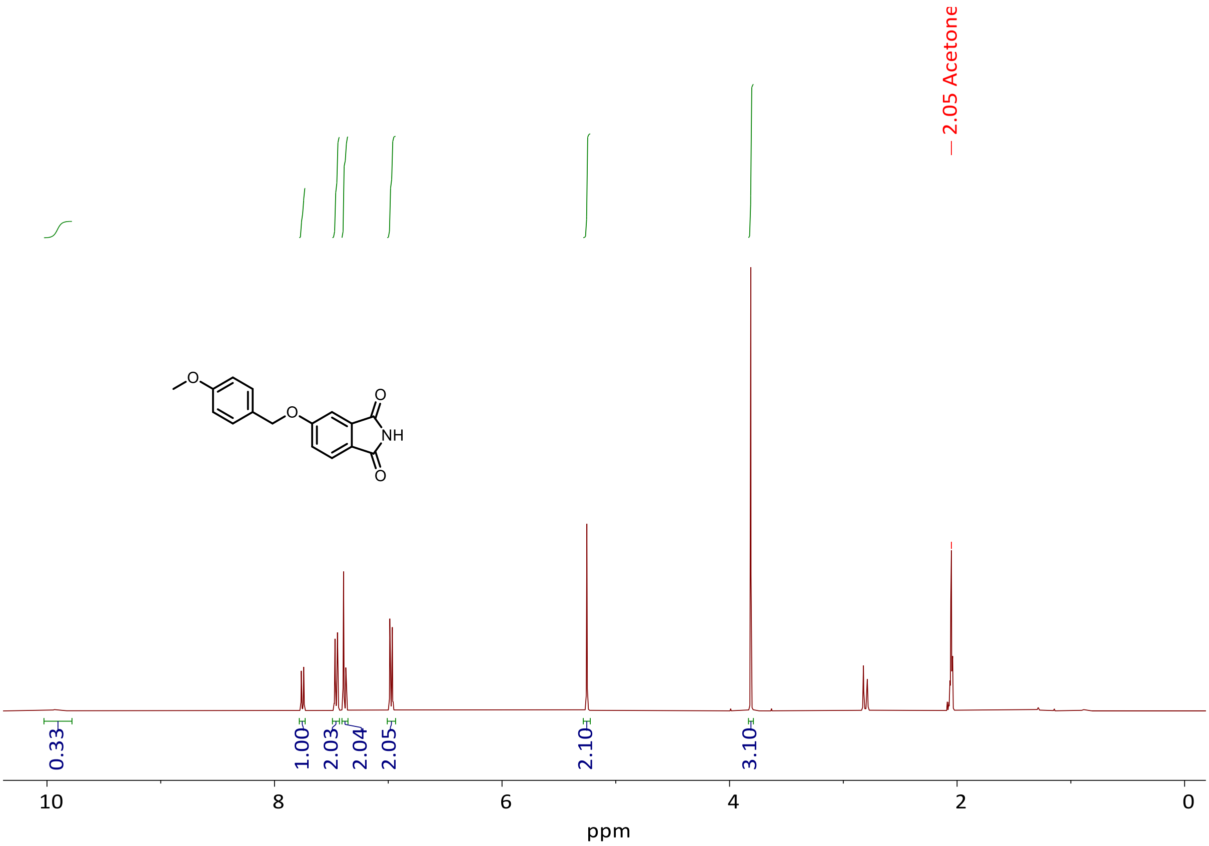

Spectrum S145. ^{1}H NMR (400 MHz, Acetone-d_6, 298 K) spectrum of compound **S46**.

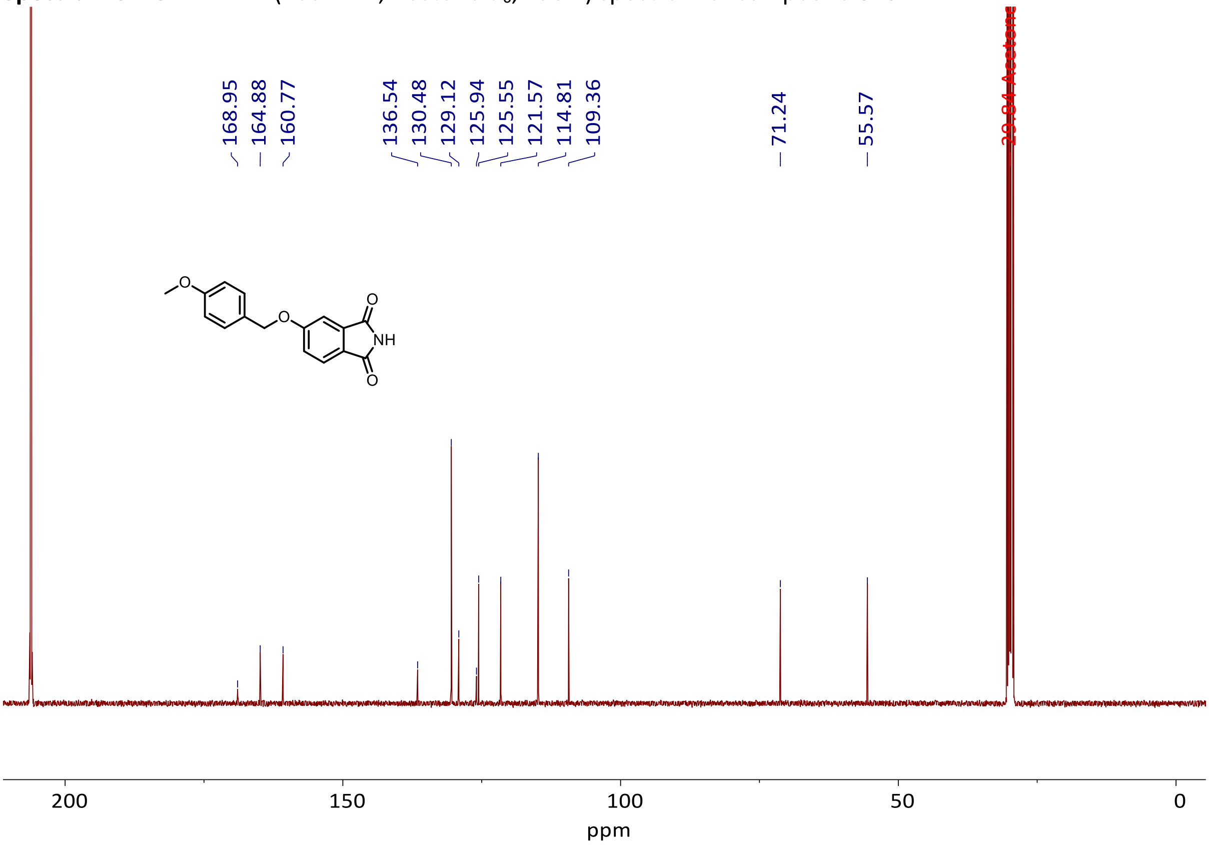

Spectrum S146. ^{13}C NMR (101 MHz, Acetone-d_6, 298 K) spectrum of compound **S46**.

9.1.67 Spectra of S47

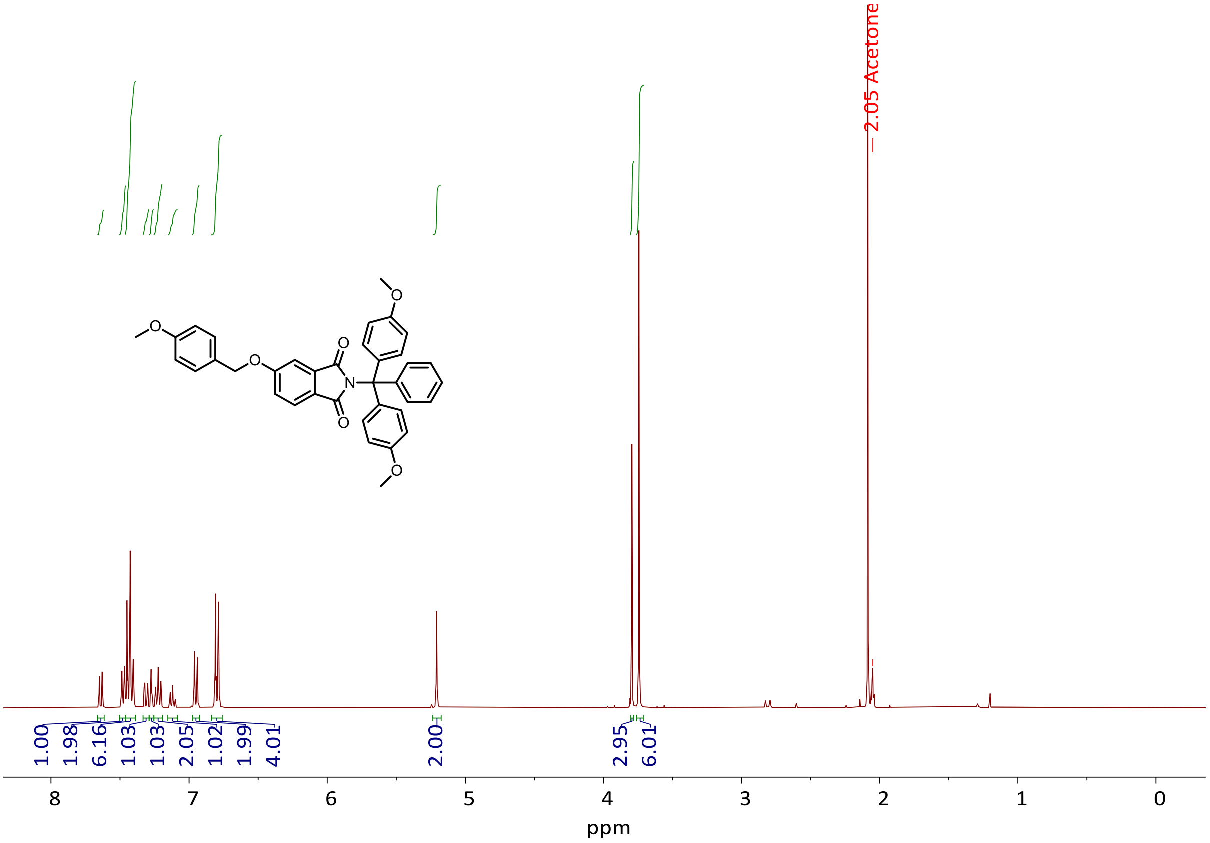

Spectrum S147. ^{1}H NMR (400 MHz, Acetone-d_6, 298 K) spectrum of compound **S47**.

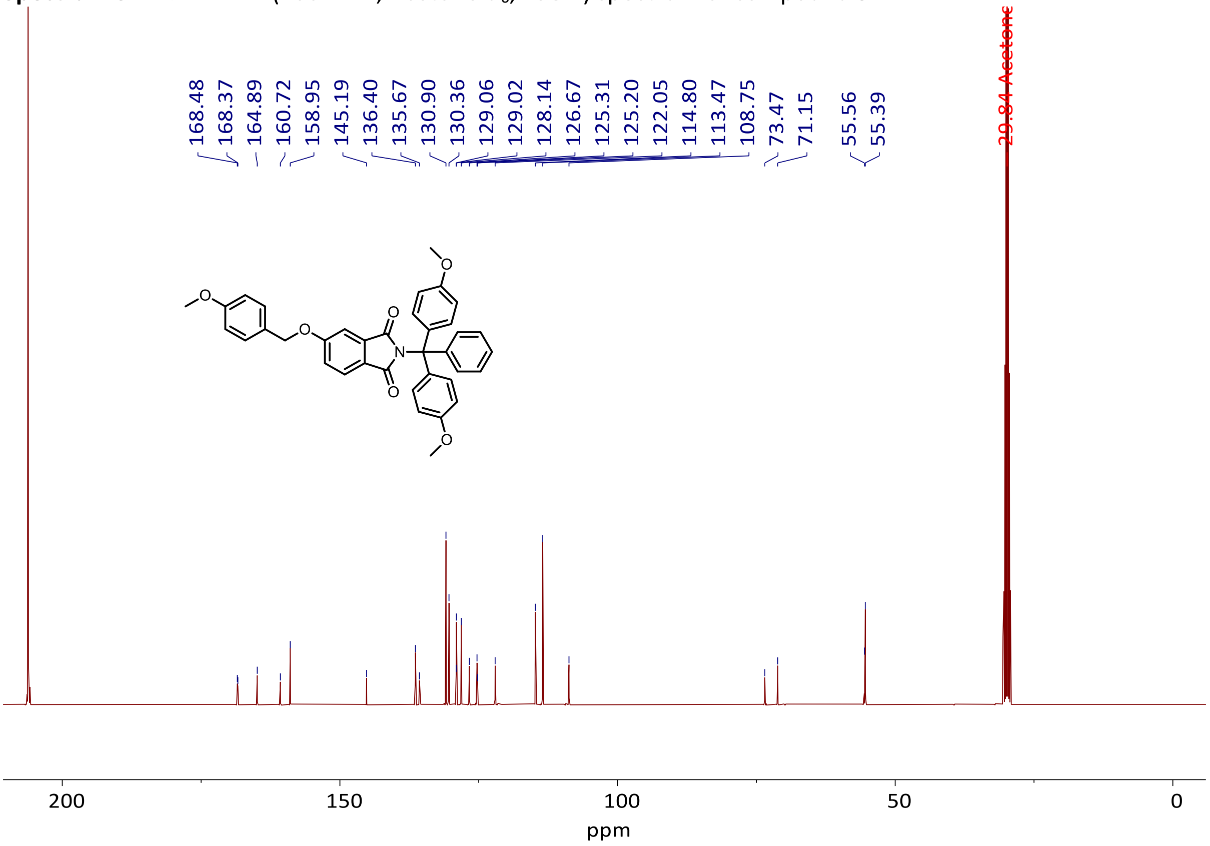

Spectrum S148. ^{13}C NMR (101 MHz, Acetone-d_6, 298 K) spectrum of compound **S47**.

9.1.68 Spectra of S48

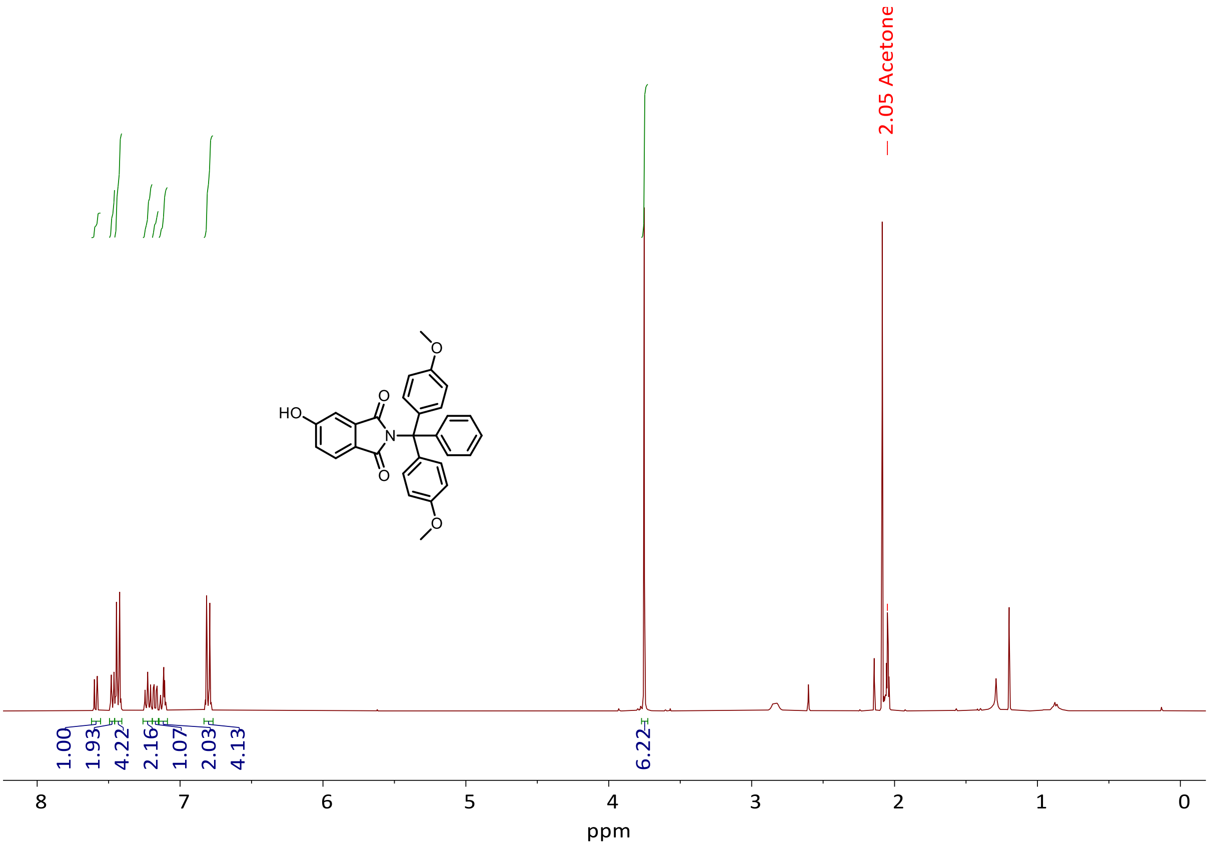

Spectrum S149. ^{1}H NMR (400 MHz, Acetone-d_6, 298 K) spectrum of compound **S48**.

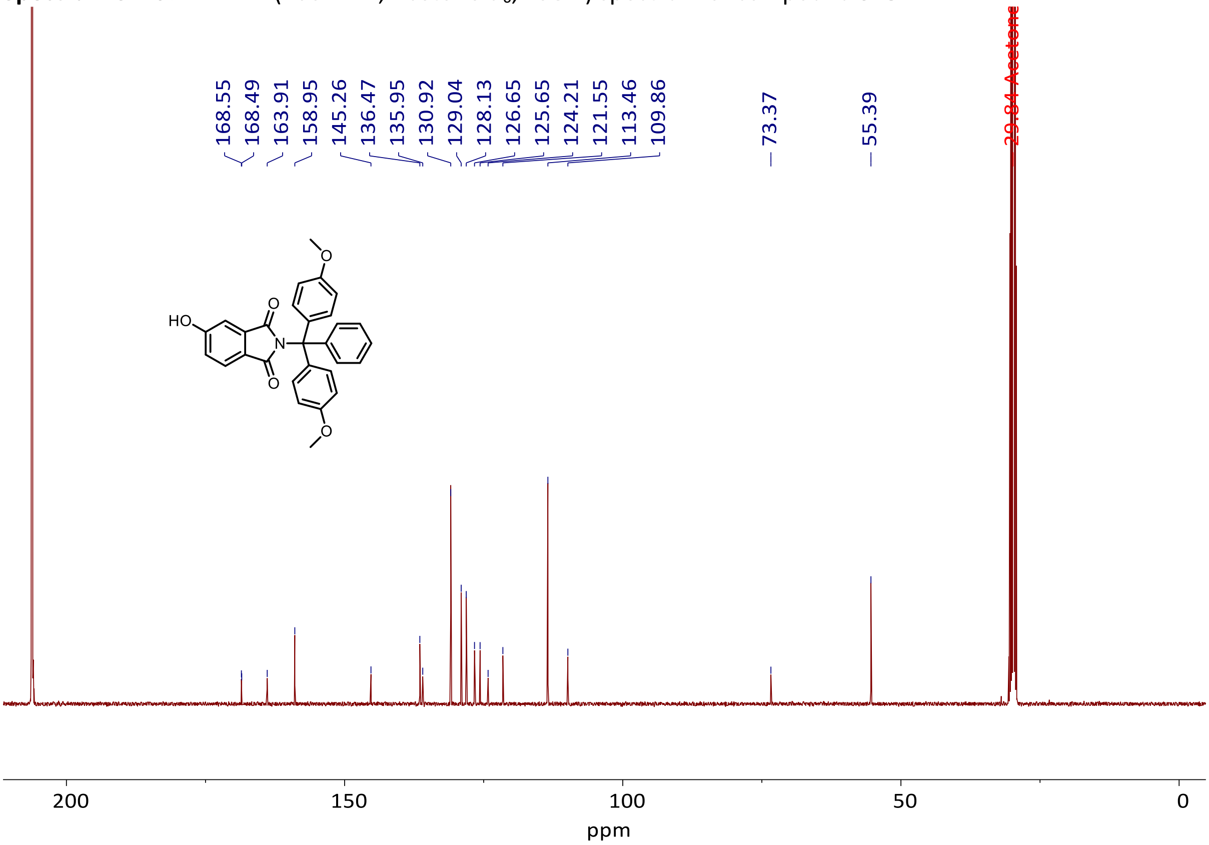

Spectrum S150. ^{13}C NMR (101 MHz, Acetone-d_6, 298 K) spectrum of compound **S48**.

9.1.69 Spectra of S49

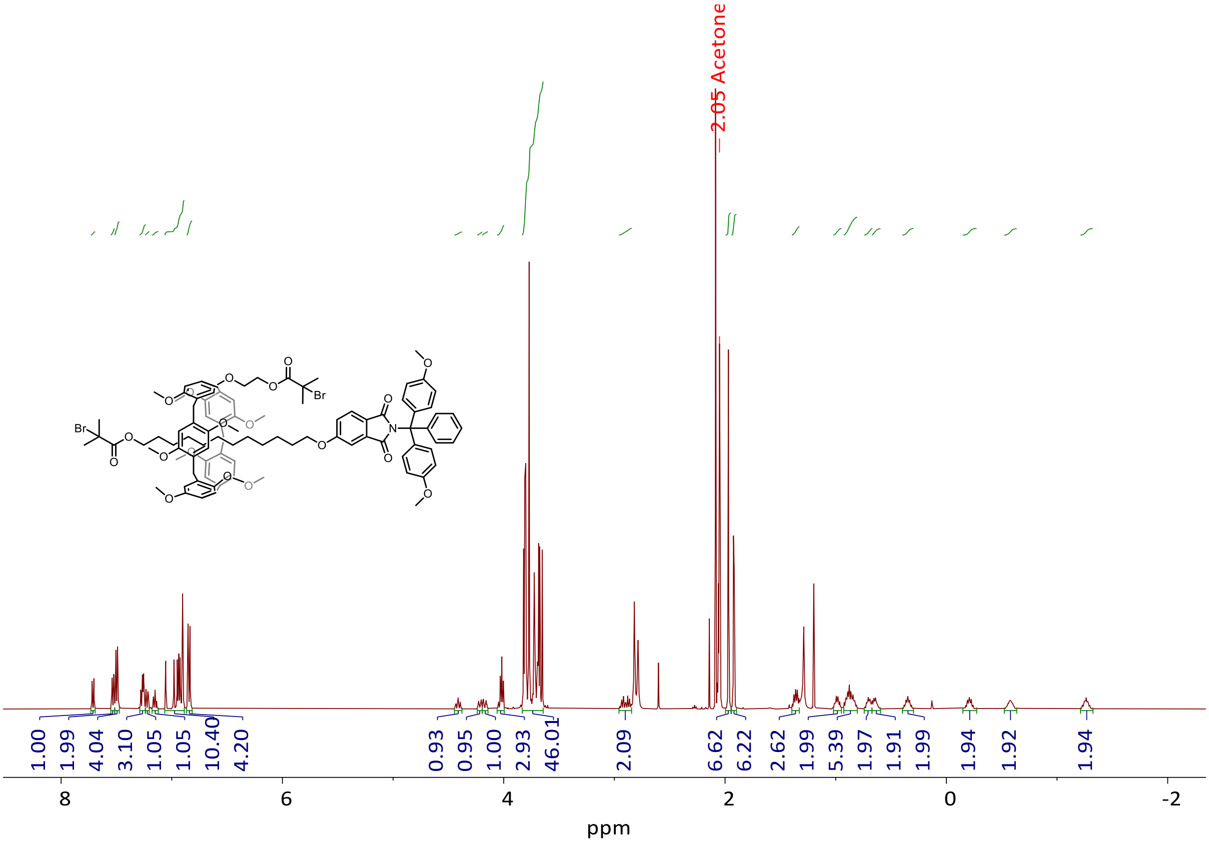

Spectrum S151. ^{1}H NMR (500 MHz, Acetone-d_6, 298 K) spectrum of compound **S49**.

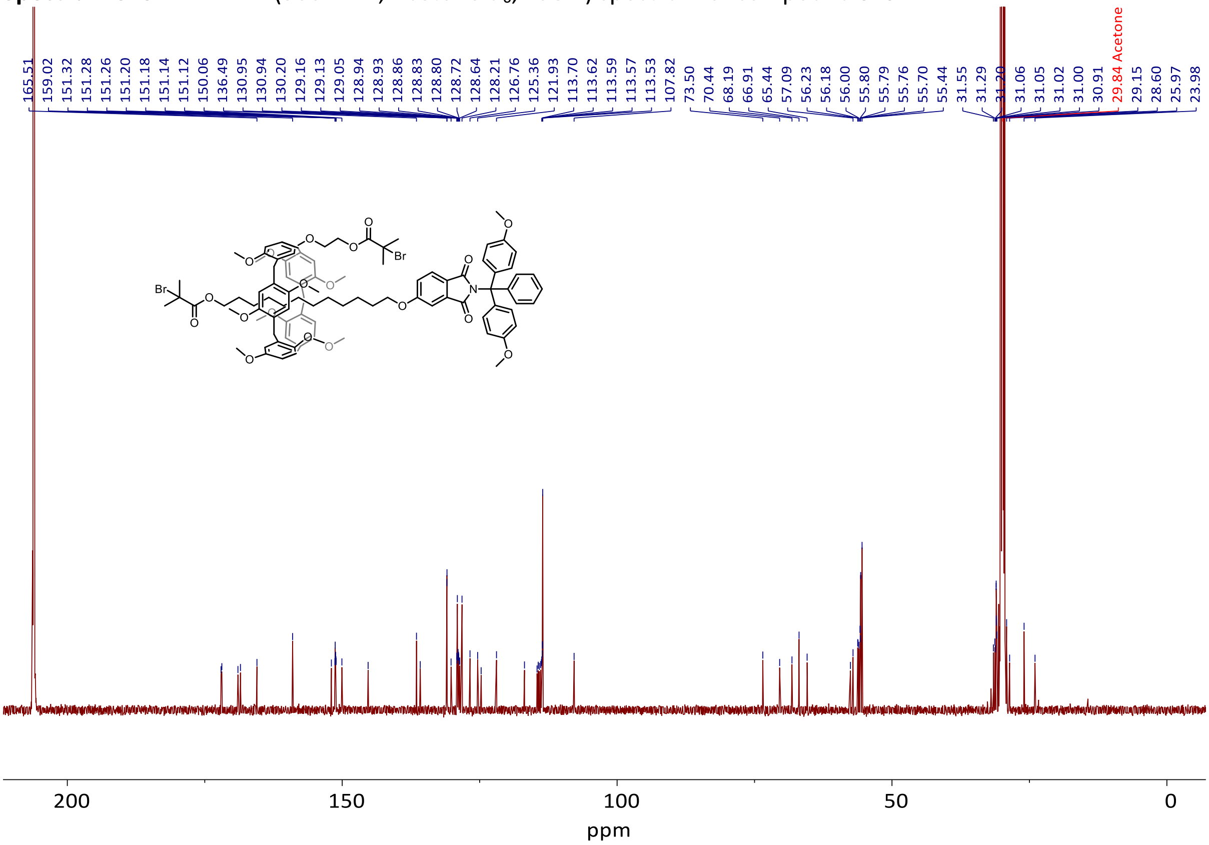

Spectrum 152. ^{13}C NMR (126 MHz, Acetone-d_6, 298 K) spectrum of compound **S49**.

9.1.70 Spectra of S50

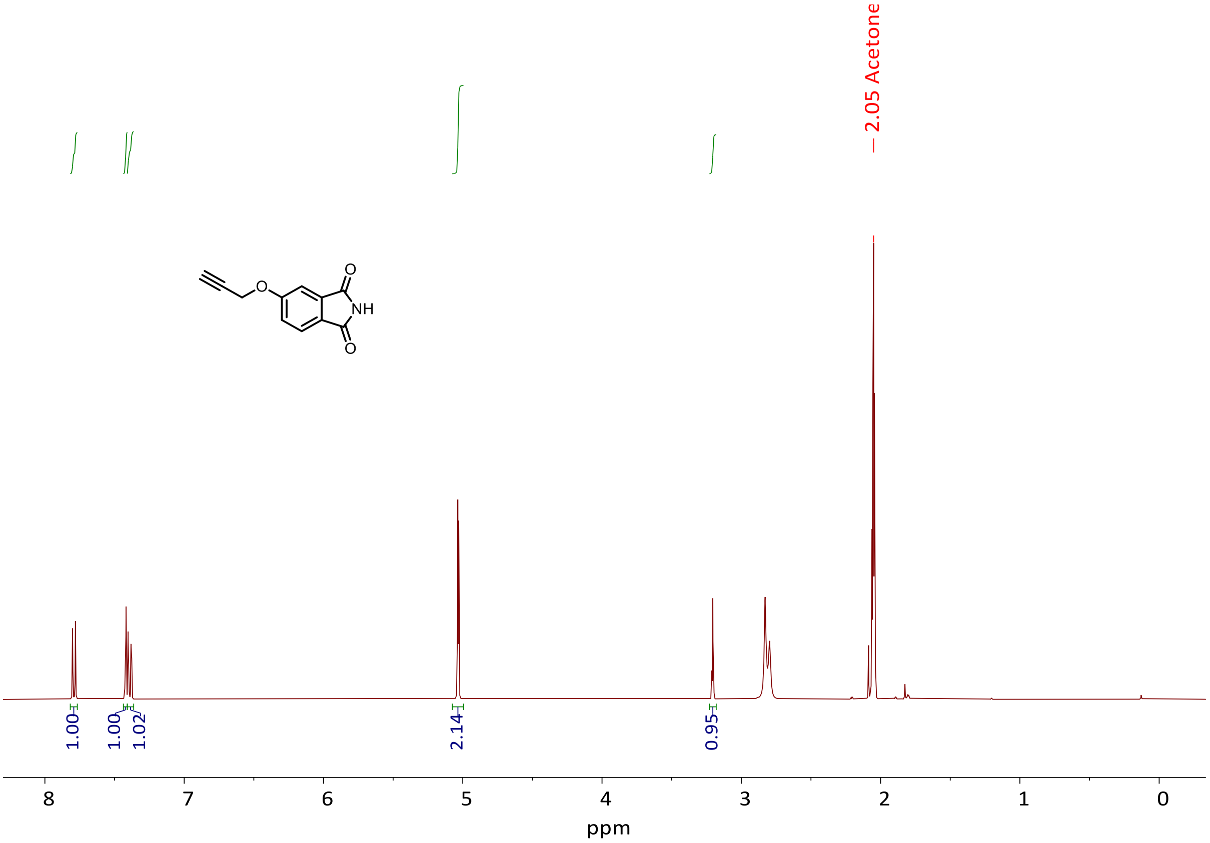

Spectrum S153. ^{1}H NMR (400 MHz, Acetone-d_6, 298 K) spectrum of compound **S50**.

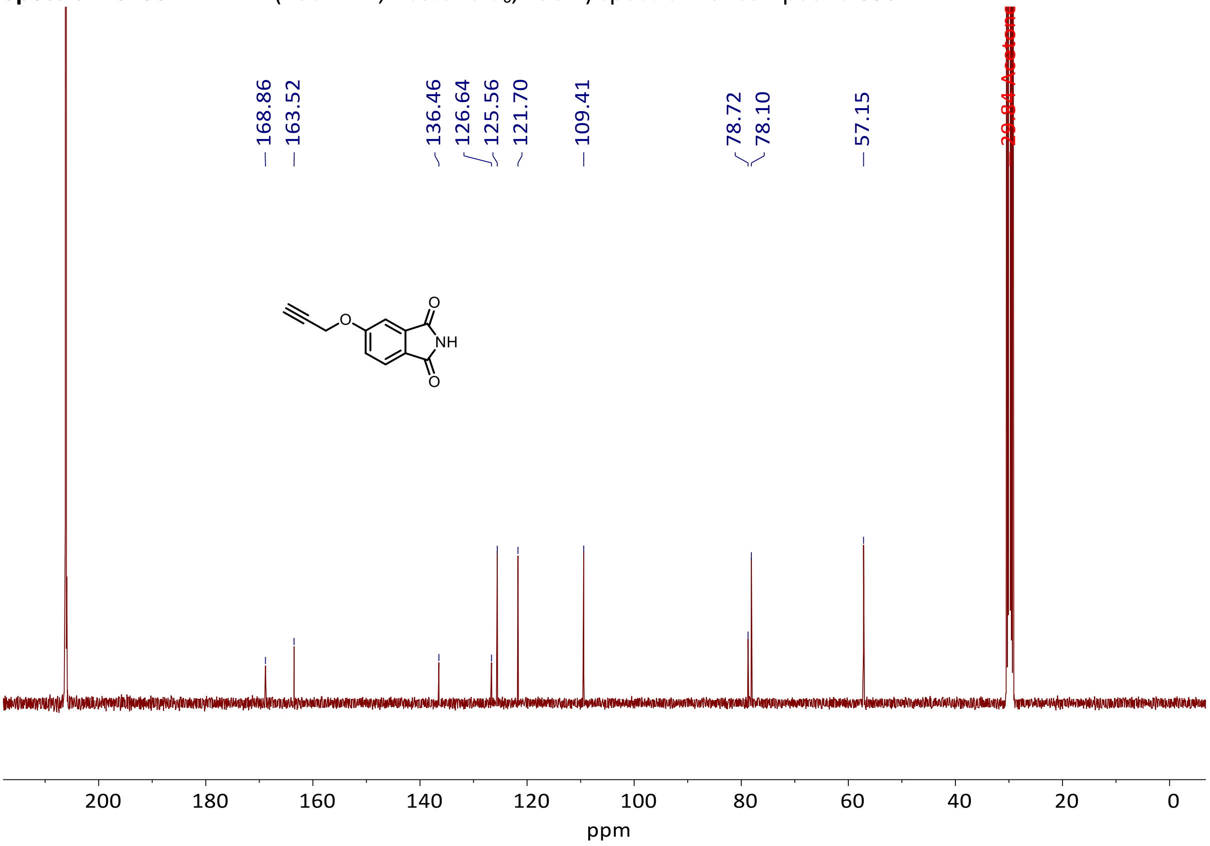

Spectrum S154. ^{13}C NMR (101 MHz, Acetone-d_6, 298 K) spectrum of compound **S50**.

9.1.71 Spectra of S51

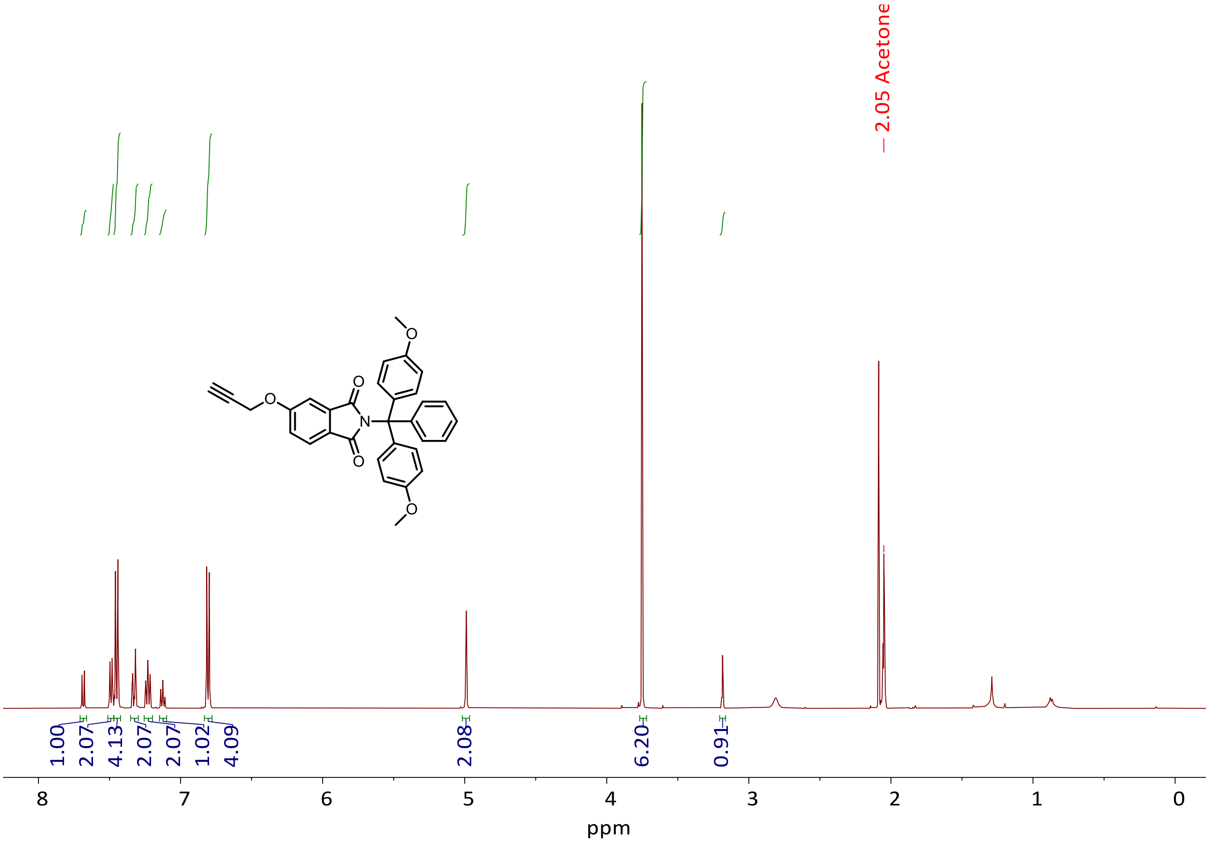

Spectrum S155. ^{1}H NMR (500 MHz, Acetone-d_6, 298 K) spectrum of compound **S51**.

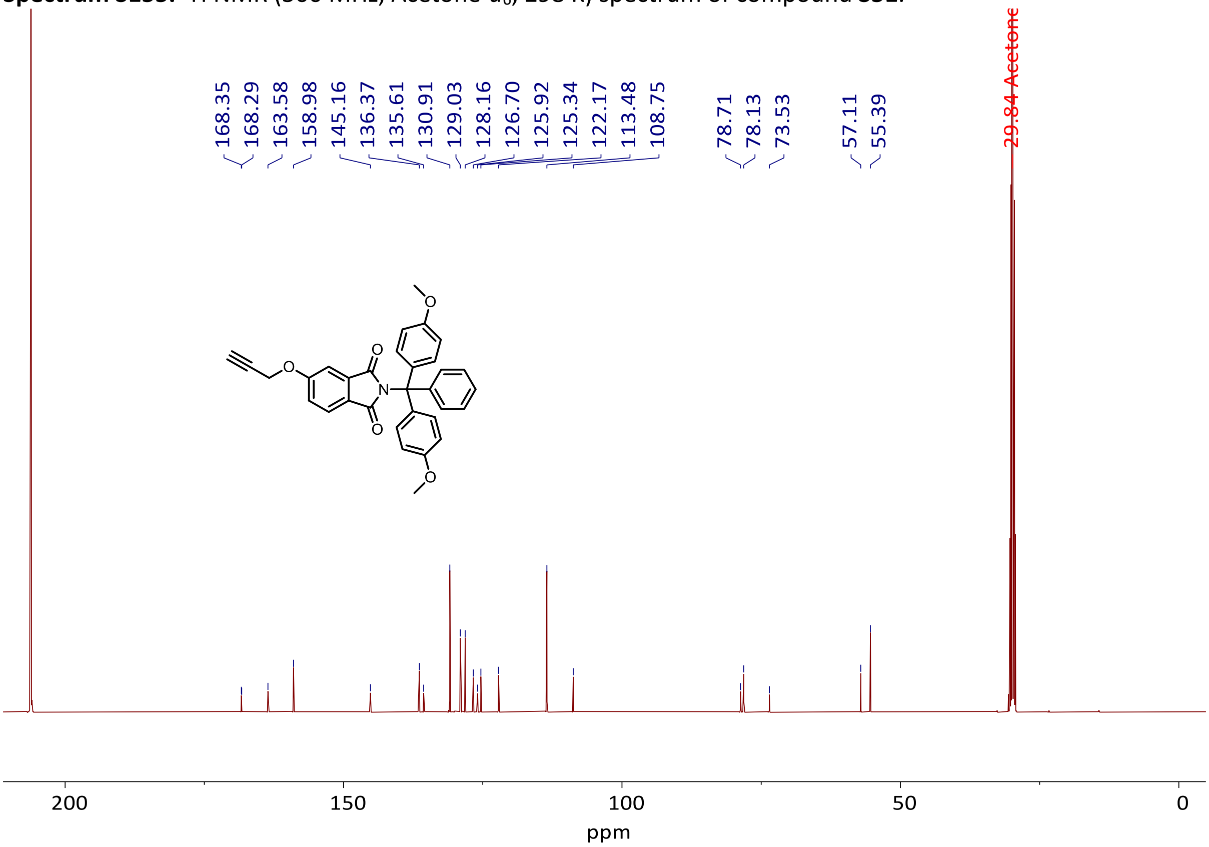

Spectrum S156. ^{13}C NMR (126 MHz, Acetone-d_6, 298 K) spectrum of compound **S51**.

9.1.72 Spectra of S52

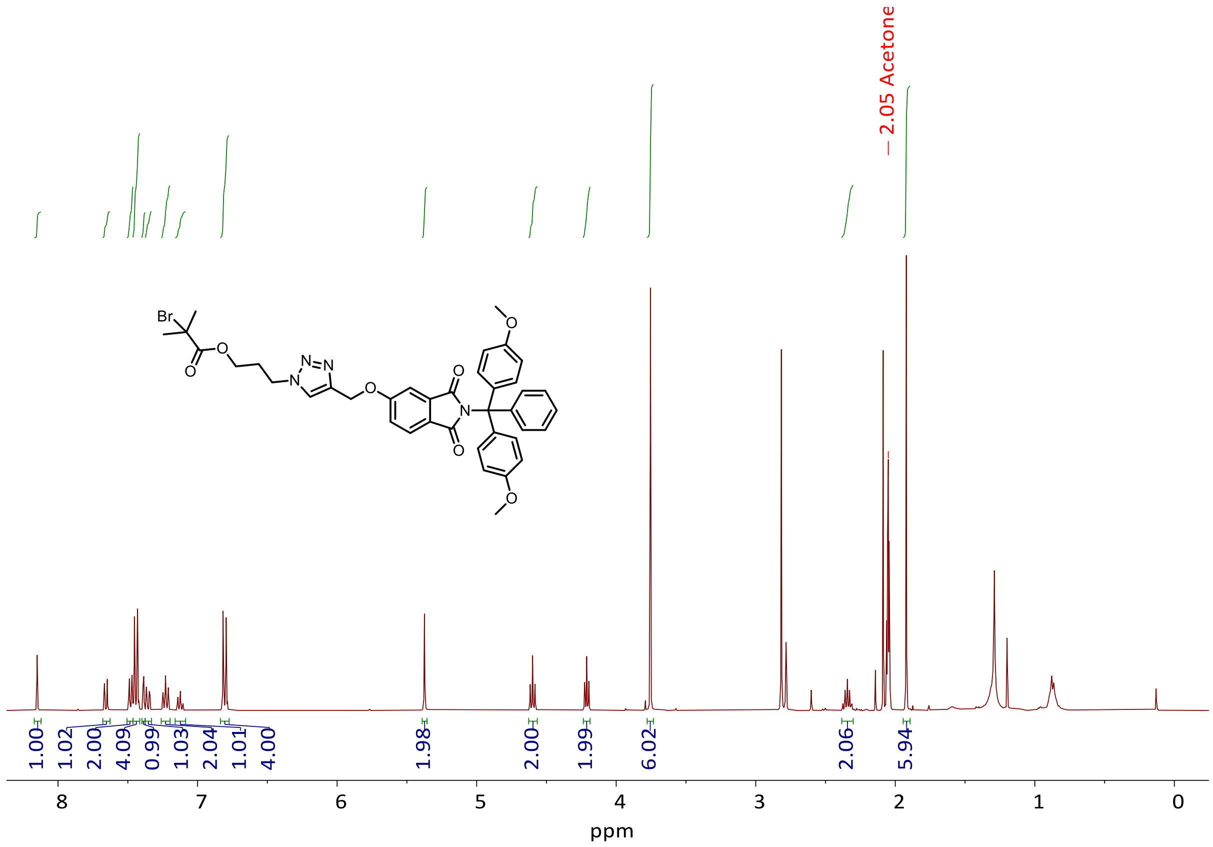

Spectrum S157. ^{1}H NMR (400 MHz, Acetone-d_6, 298 K) spectrum of compound **S52**.

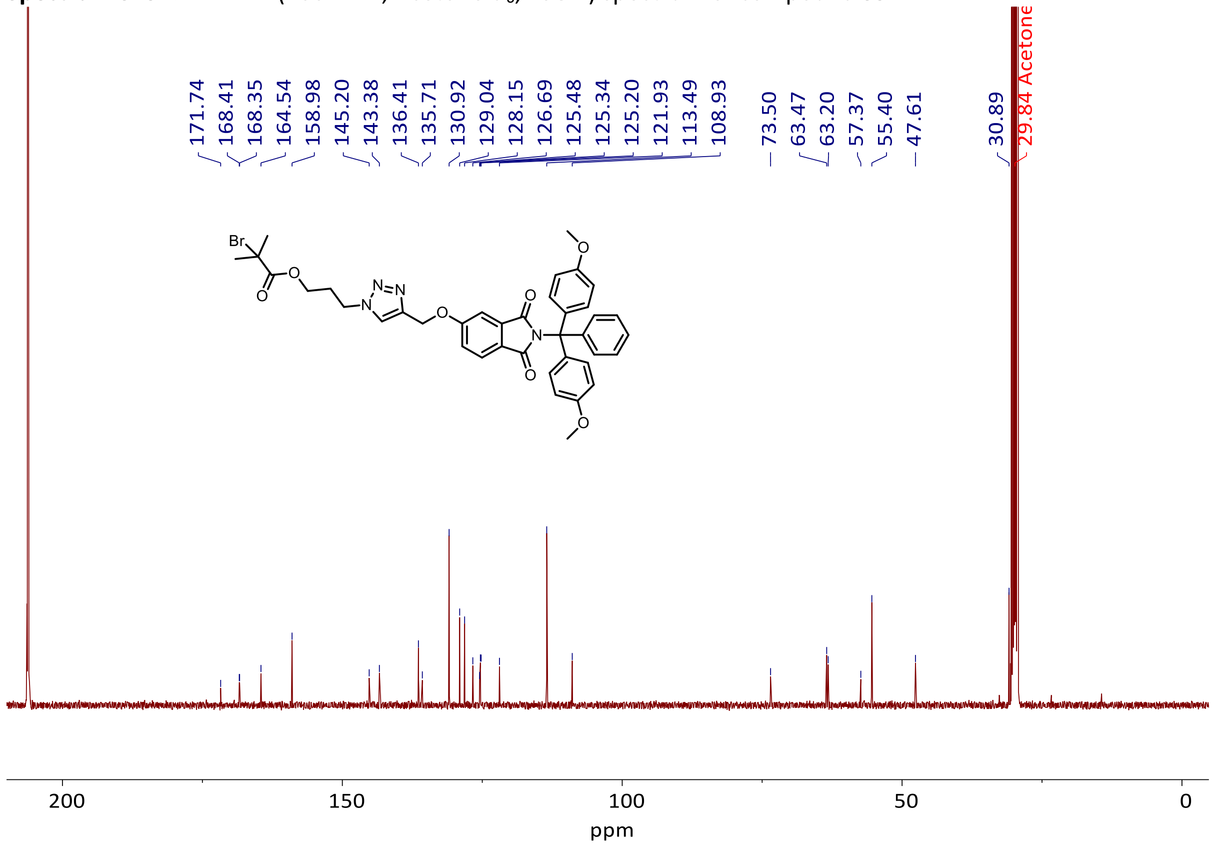

Spectrum S158. ^{13}C NMR (101 MHz, Acetone-d_6, 298 K) spectrum of compound **S52**.

9.1.73 Spectra of S54

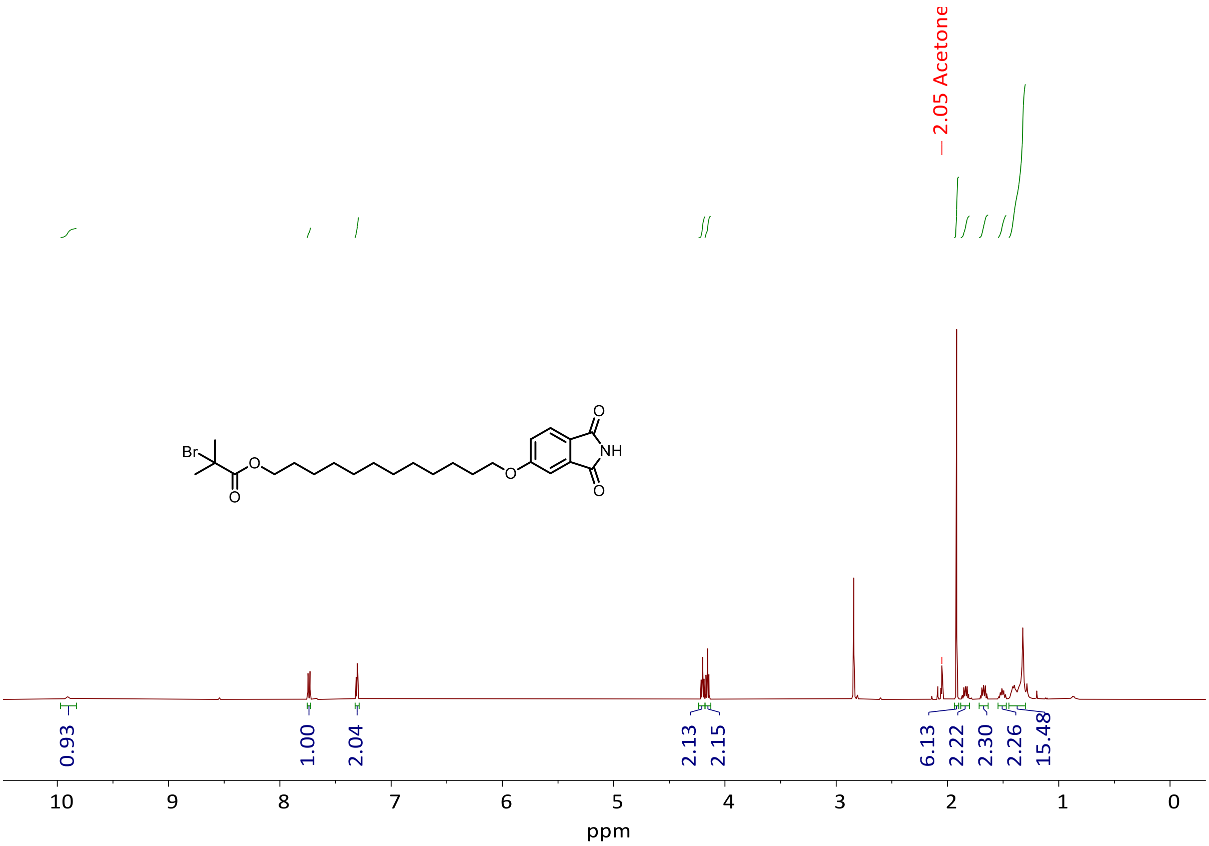

Spectrum S159. ^{1}H NMR (500 MHz, Acetone-d_6, 298 K) spectrum of compound **S54**.

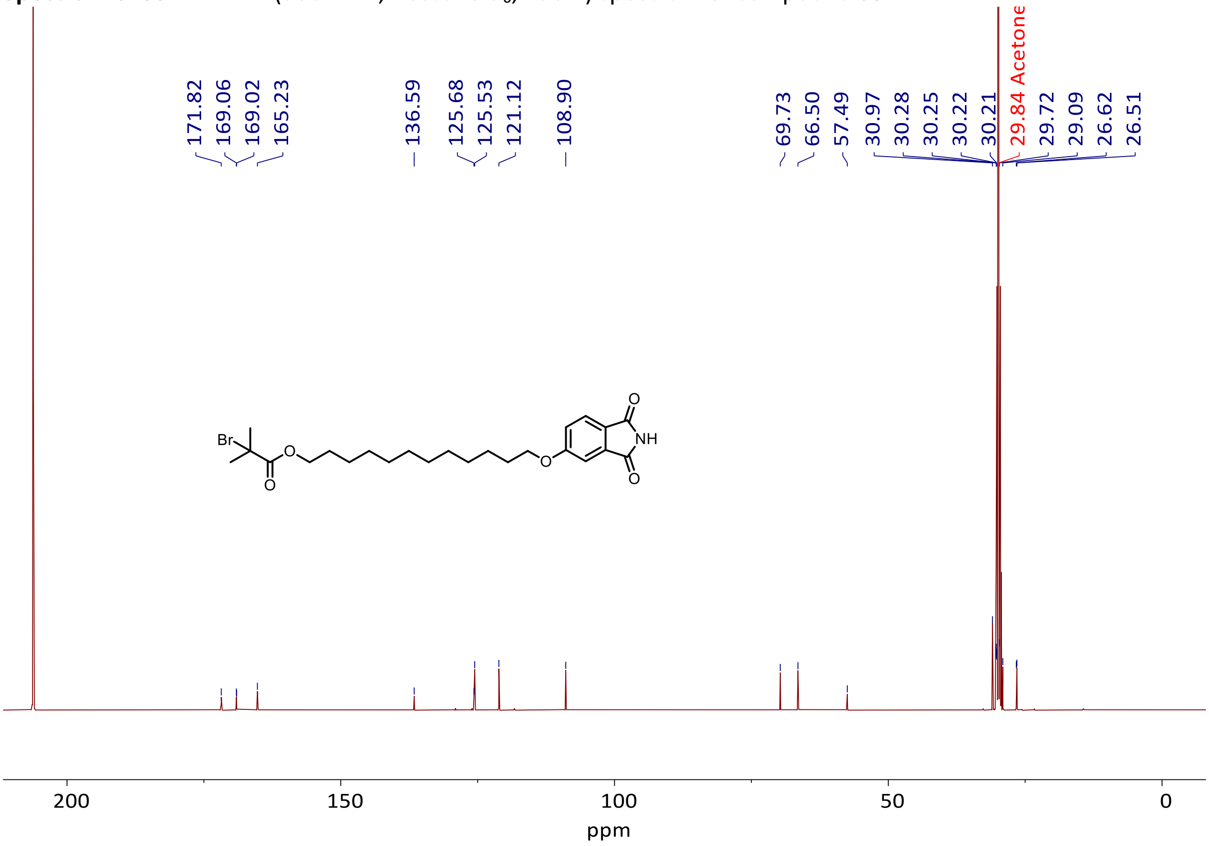

Spectrum S160. ^{13}C NMR (126 MHz, Acetone-d_6, 298 K) spectrum of compound **S54**.

9.2 Polymer NMR Spectra

9.2.1 Spectra of polymer S55*exo*-112

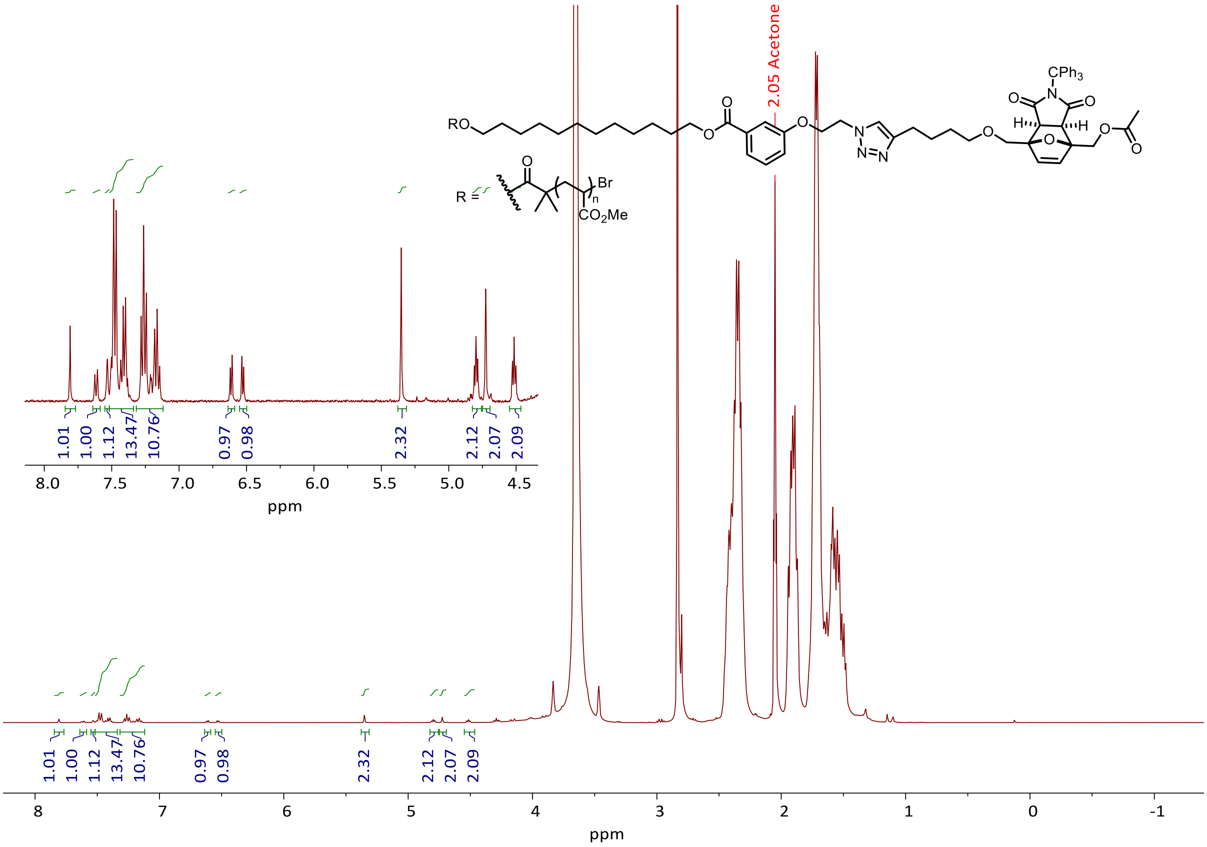

Spectrum S161. ^{1}H NMR (400 MHz, Acetone-d_6, 298 K) spectrum of polymer **S55*exo*-112**.

9.2.2 Spectra of polymer S55$_{endo\text{-}65}$

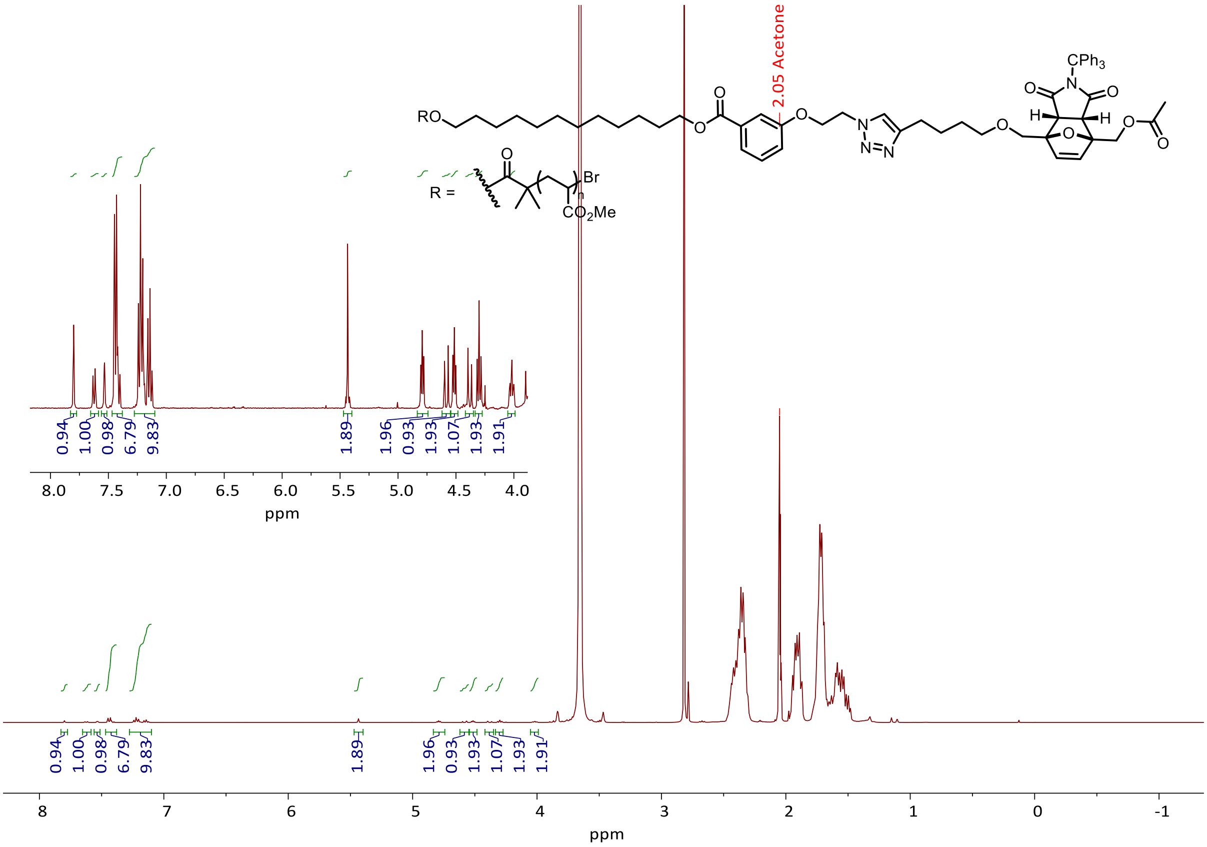

Spectrum S162. ^{1}H NMR (400 MHz, Acetone-d_6, 298 K) spectrum of polymer **S55$_{endo\text{-}65}$**.

9.2.3 Spectra of polymer $9_{trans/exo\text{-}109}$

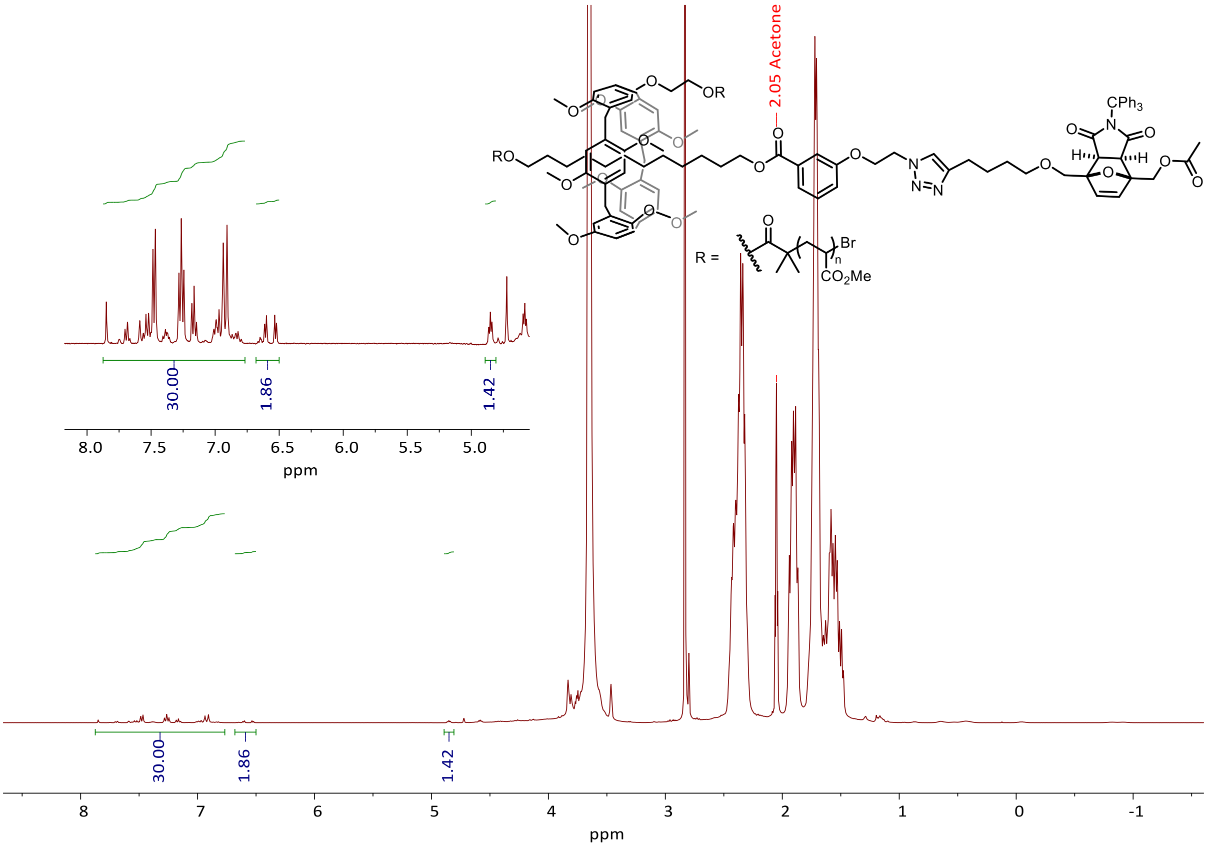

Spectrum S163. ^{1}H NMR (400 MHz, Acetone-d_6, 298 K) spectrum of polymer $\mathbf{9}_{trans/exo\text{-}109}$.

9.2.4 Spectra of polymer $9_{trans/exo\text{-}114}$

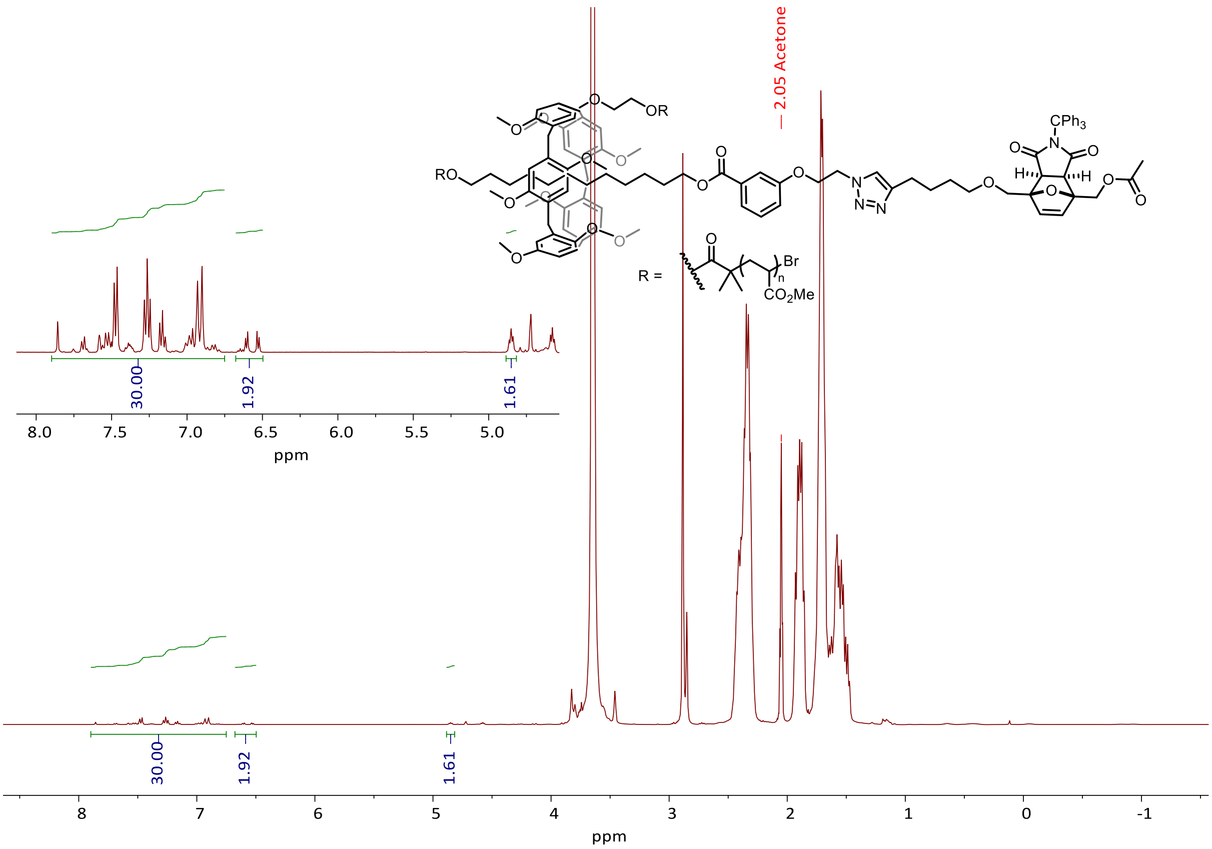

Spectrum S164. ^{1}H NMR (400 MHz, Acetone-d_6, 298 K) spectrum of polymer $\mathbf{9}_{trans/exo\text{-}114}$.

9.2.5 Spectra of polymer $9_{cis/exo\text{-}114}$

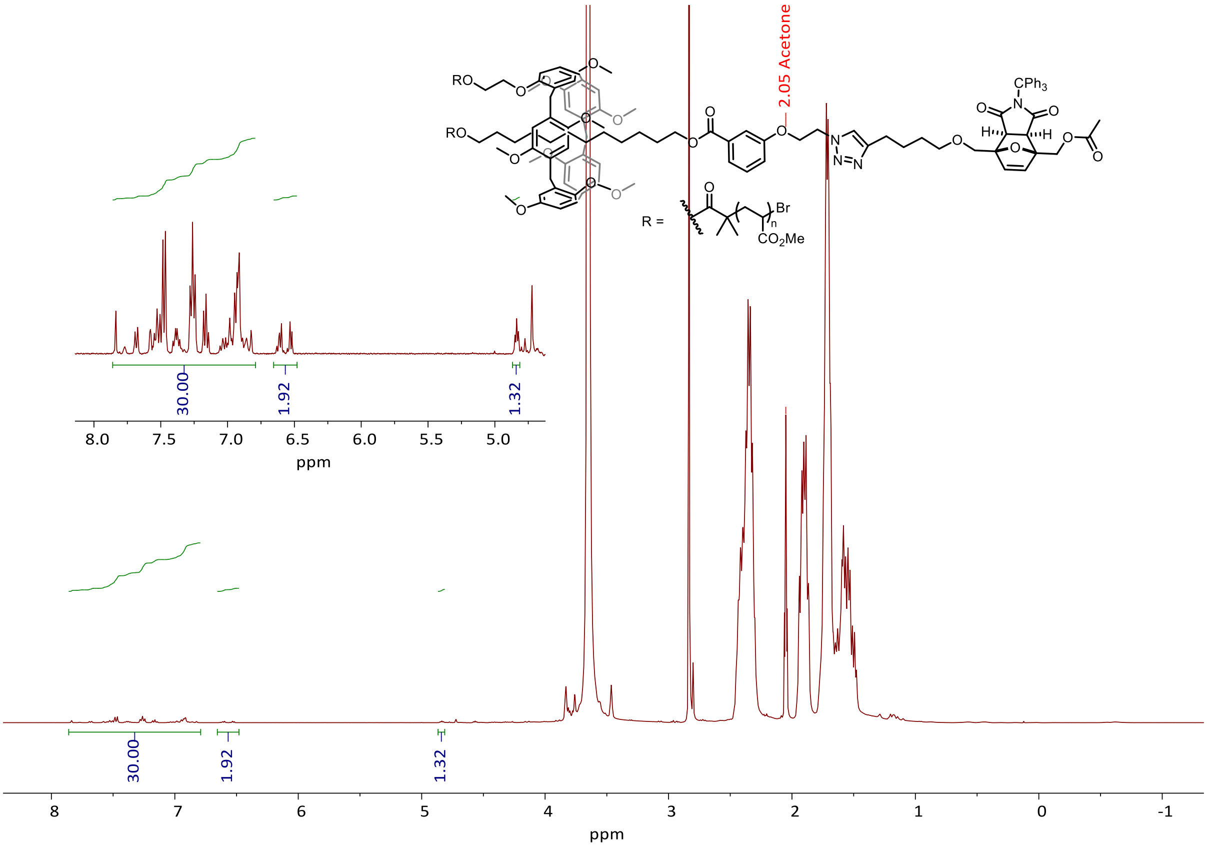

Spectrum S165. ^{1}H NMR (400 MHz, Acetone-d_6, 298 K) spectrum of polymer $\mathbf{9}_{cis/exo\text{-}114}$.

9.2.6 Spectra of polymer $9_{trans/endo\text{-}90}$

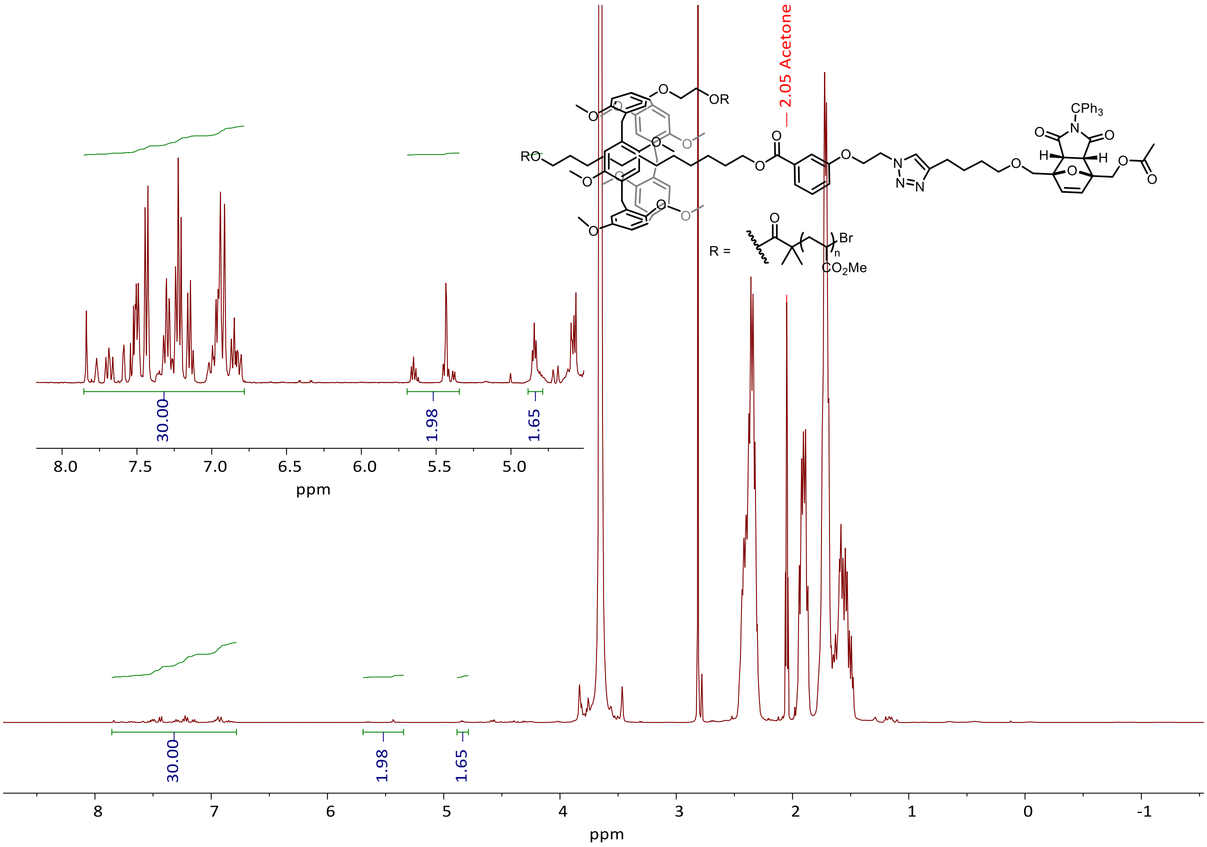

Spectrum S166. ^{1}H NMR (400 MHz, Acetone-d_6, 298 K) spectrum of polymer $\mathbf{9}_{trans/endo\text{-}90}$.

9.2.7 Spectra of polymer $\mathbf{9}_{cis/endo\text{-}92}$

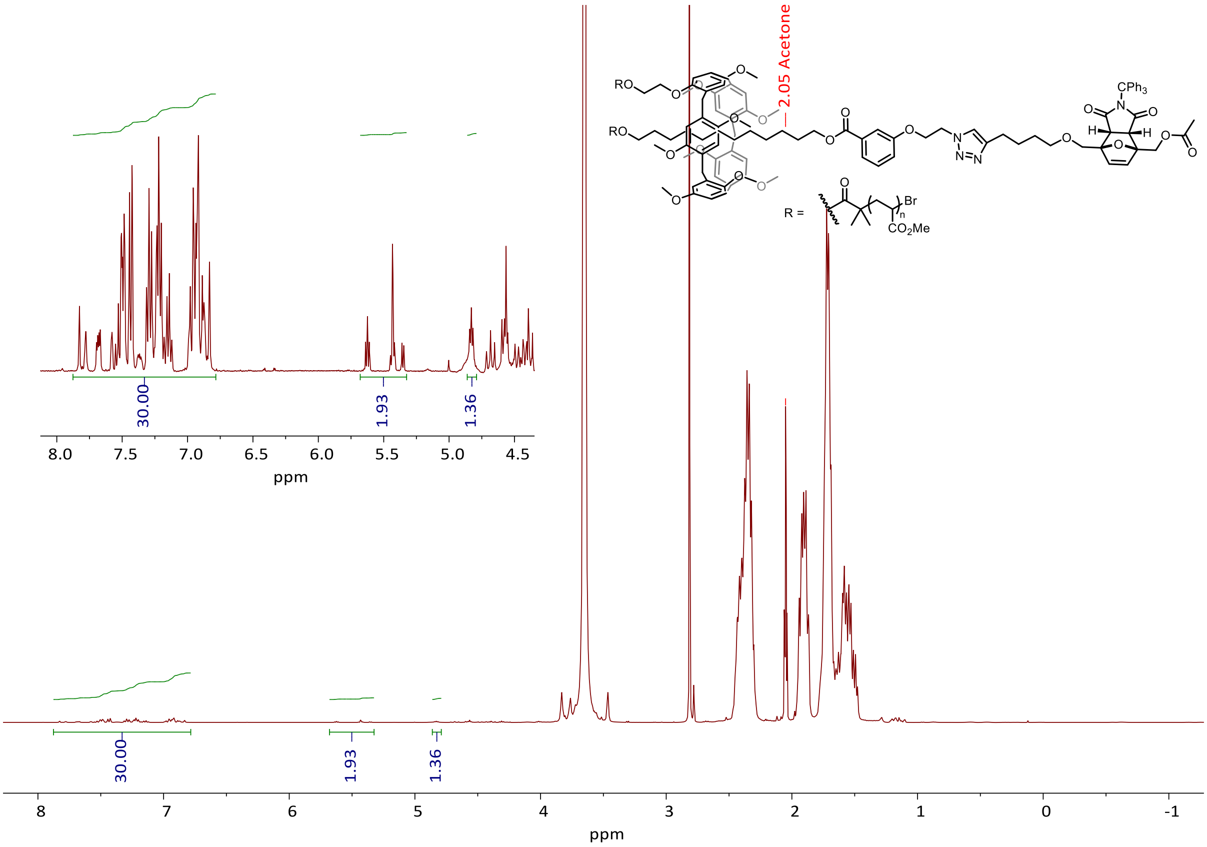

Spectrum S167. ^{1}H NMR (400 MHz, Acetone-d_6, 298 K) spectrum of polymer $\mathbf{9}_{cis/endo\text{-}92}$.

9.2.8 Spectra of polymer 1_3a_{-210}

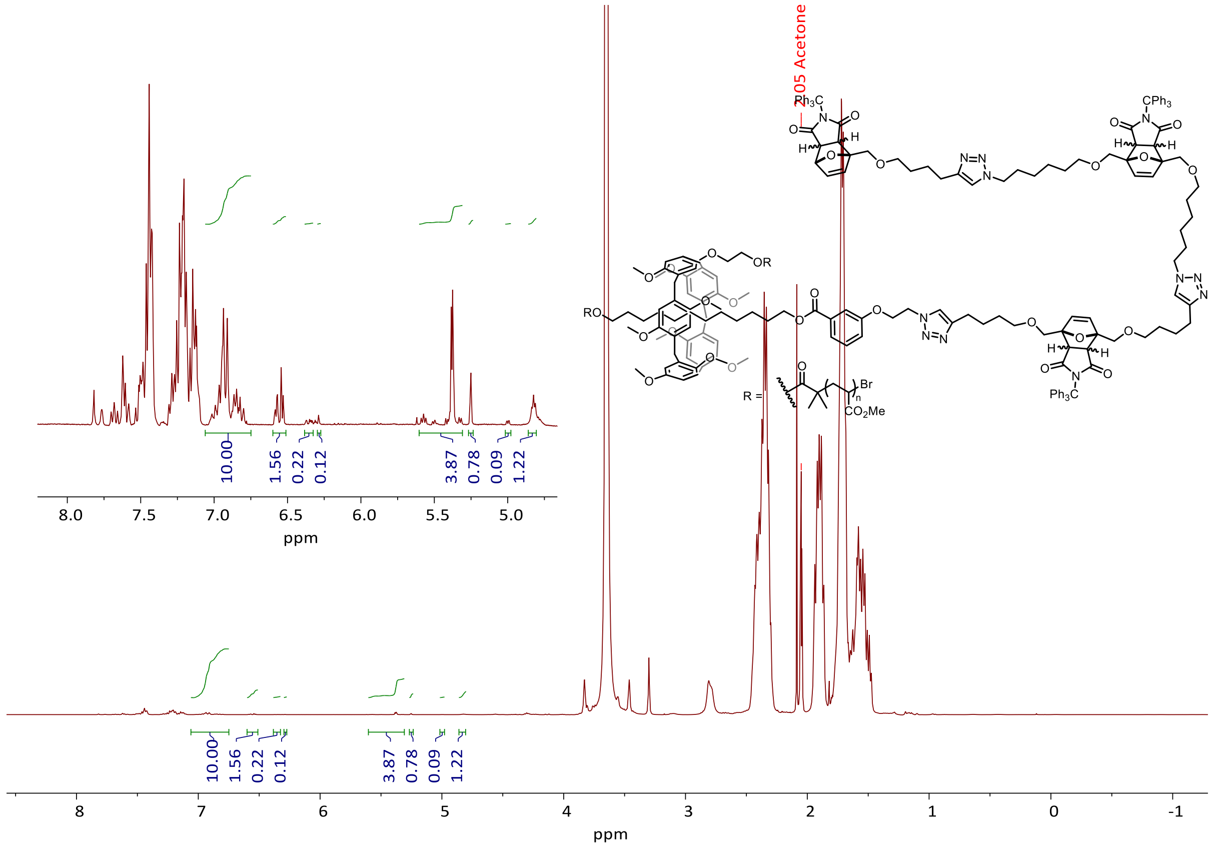

Spectrum S168. ^{1}H NMR (400 MHz, Acetone-d_6, 298 K) spectrum of polymer **1_3a_{-210}**.

9.2.9 Spectra of polymer 1_3b_{-142}

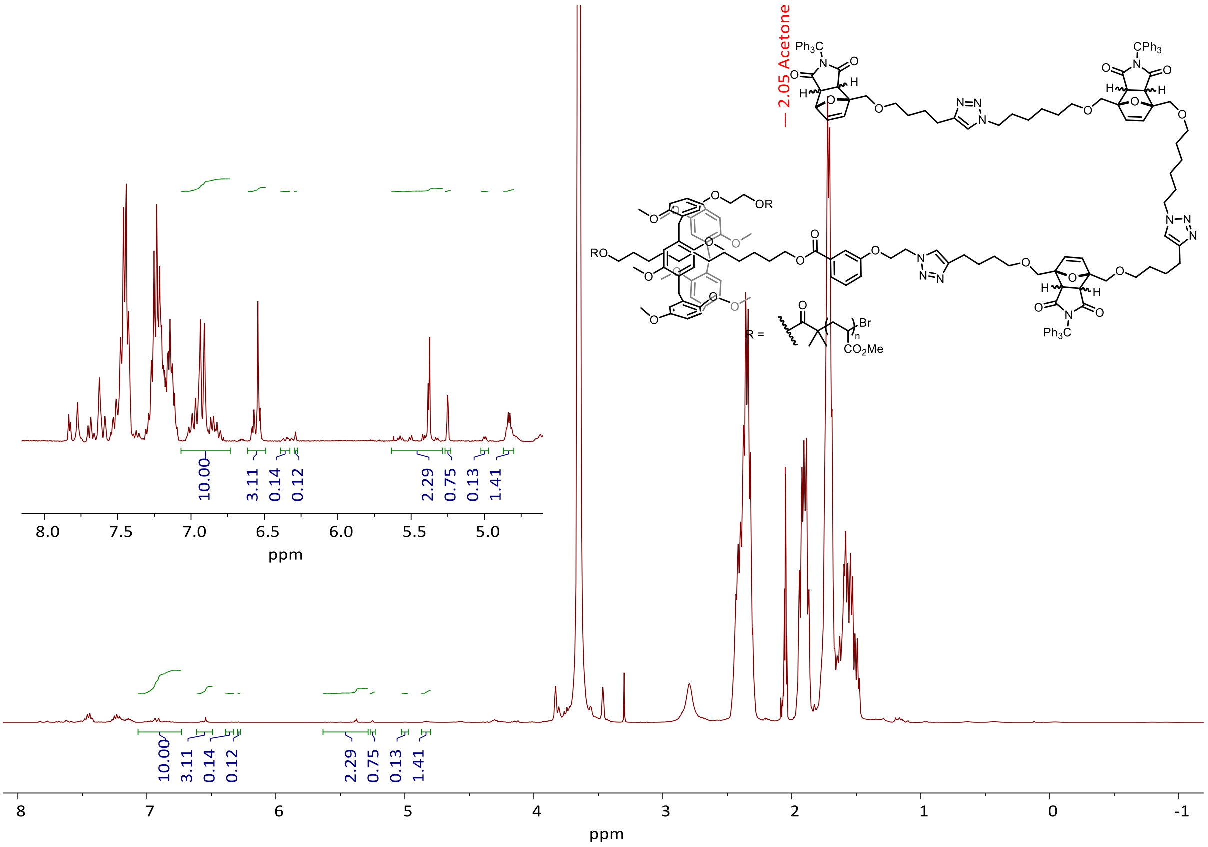

Spectrum S169. ^{1}H NMR (400 MHz, Acetone-d_6, 298 K) spectrum of polymer $\mathbf{1_3b_{-142}}$.

9.2.10 Spectra of polymer 1_3b_{-171}

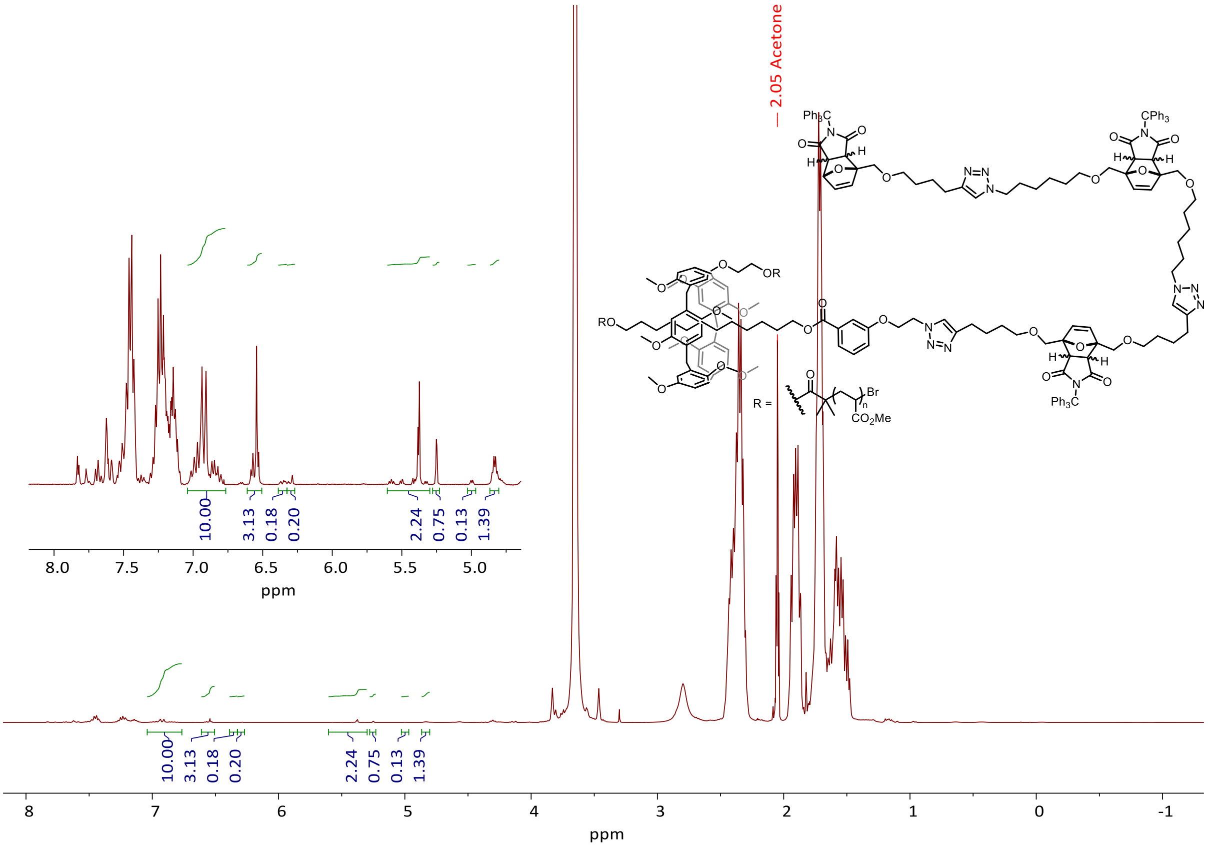

Spectrum S170. ^{1}H NMR (400 MHz, Acetone-d_6, 298 K) spectrum of polymer $\mathbf{1_3b_{-171}}$.

9.2.11 Spectra of polymer **1₃b₋₁₇₈**

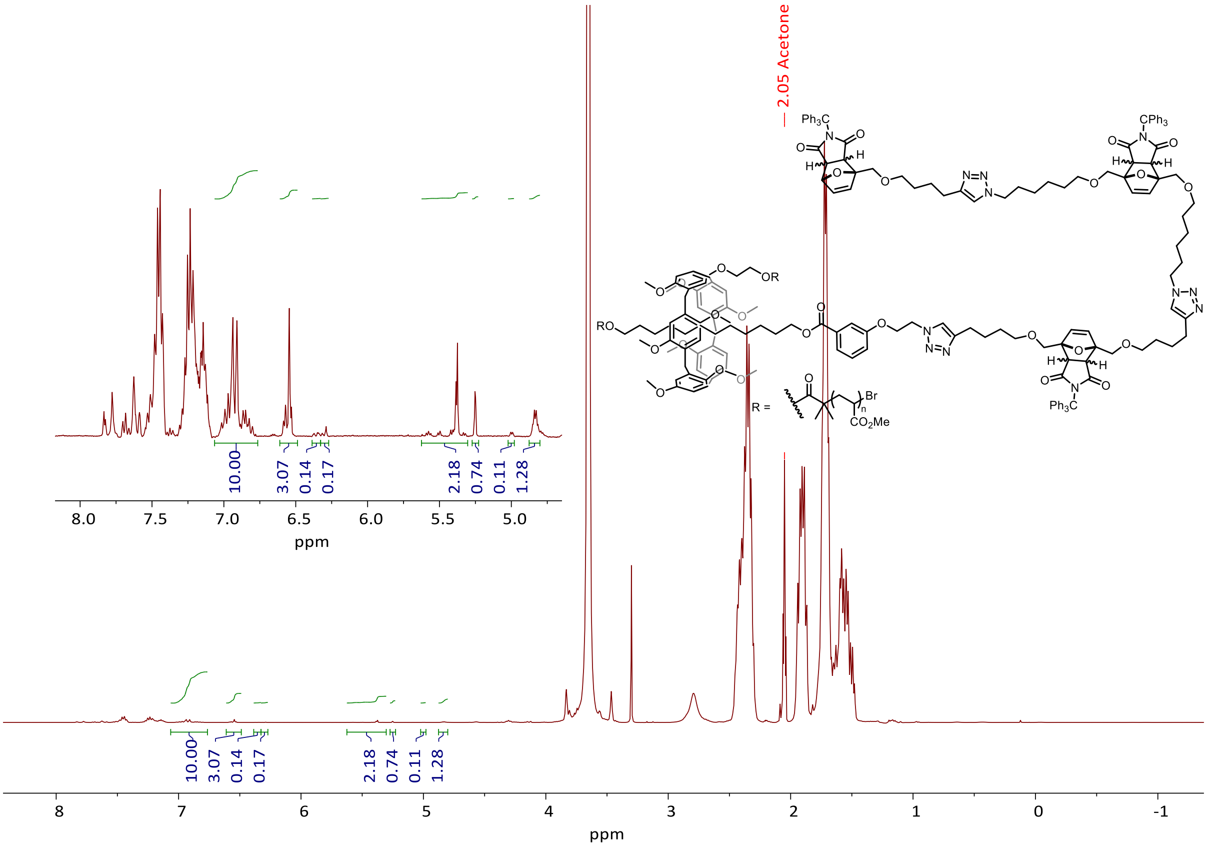

Spectrum S171. ^{1}H NMR (400 MHz, Acetone-d_6, 298 K) spectrum of polymer **1$_3$b$_{-178}$**.

9.2.12 Spectra of polymer 1_3c_{-174}

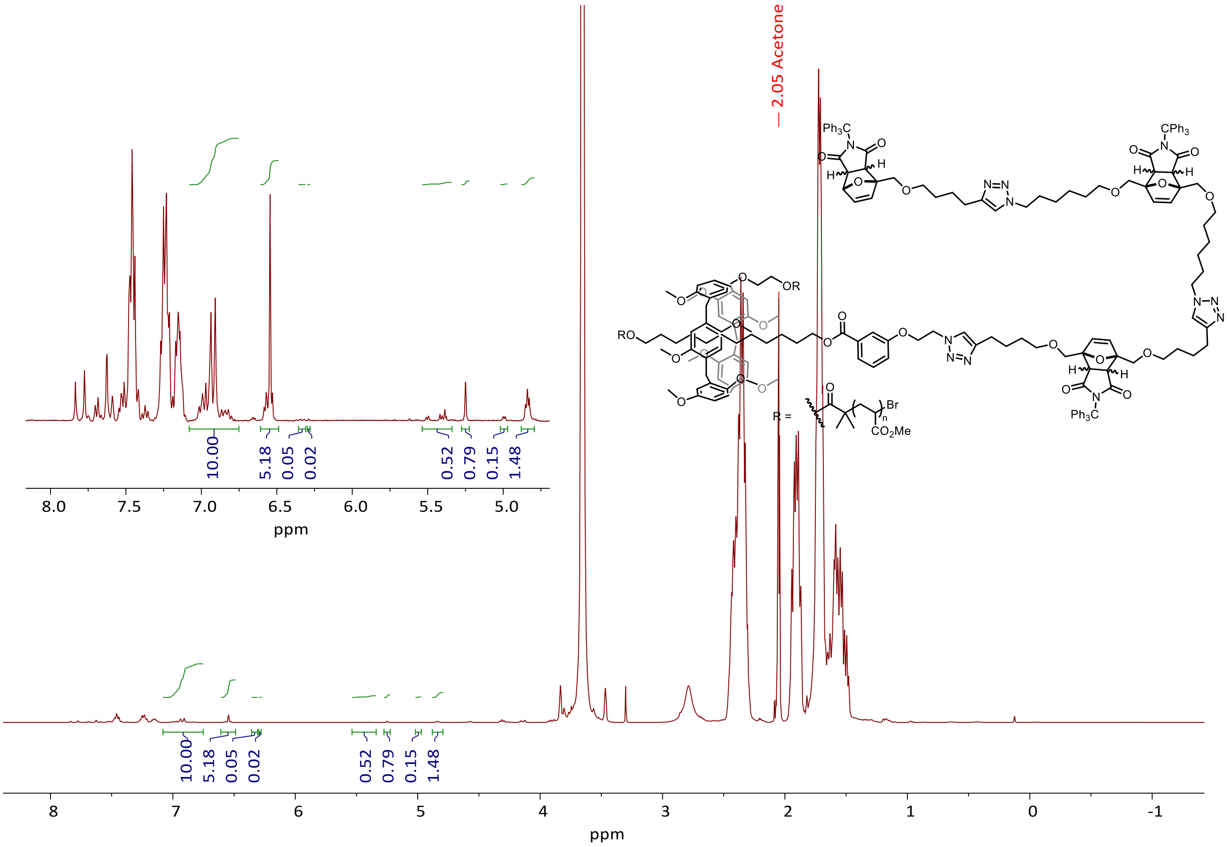

Spectrum S172. ^{1}H NMR (400 MHz, Acetone-d_6, 298 K) spectrum of polymer **1_3c_{-174}**.

9.2.13 Spectra of polymer 1_3d_{-89}

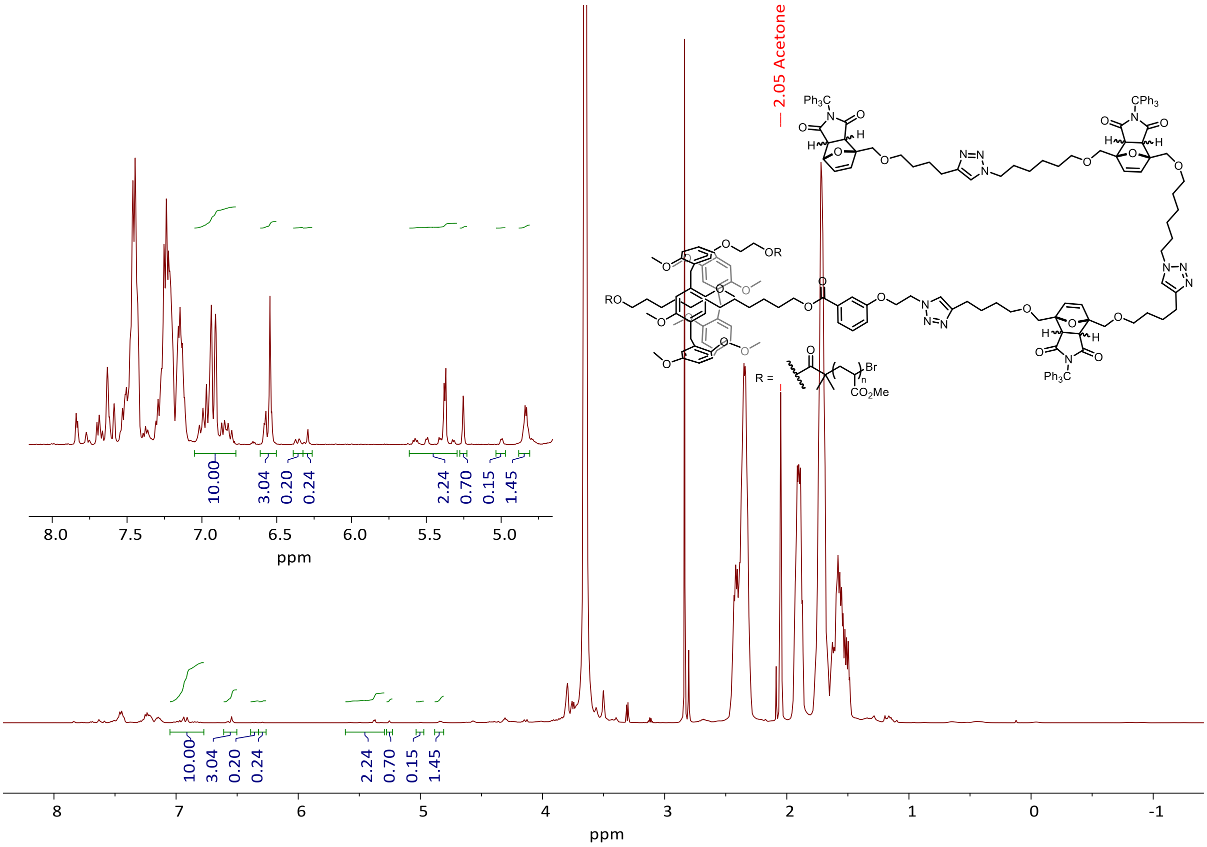

Spectrum S173. ^{1}H NMR (400 MHz, Acetone-d_6, 298 K) spectrum of polymer $\mathbf{1_3d_{-89}}$.

9.2.14 Spectra of polymer $1_{5\text{-}60}$

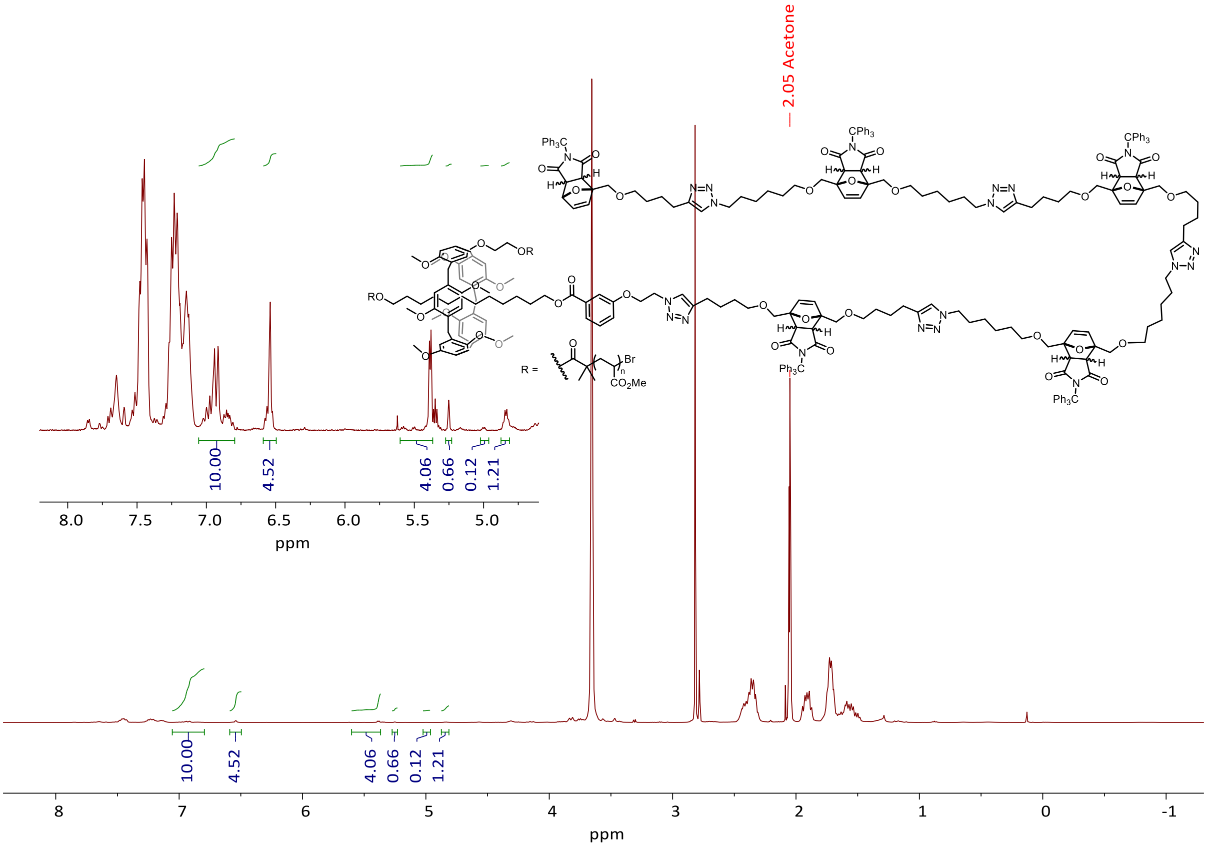

Spectrum S174. ^{1}H NMR (400 MHz, Acetone-d_6, 298 K) spectrum of polymer $\mathbf{1_{5\text{-}60}}$.

9.2.15 Spectra of polymer $1_{5\text{-}165}$

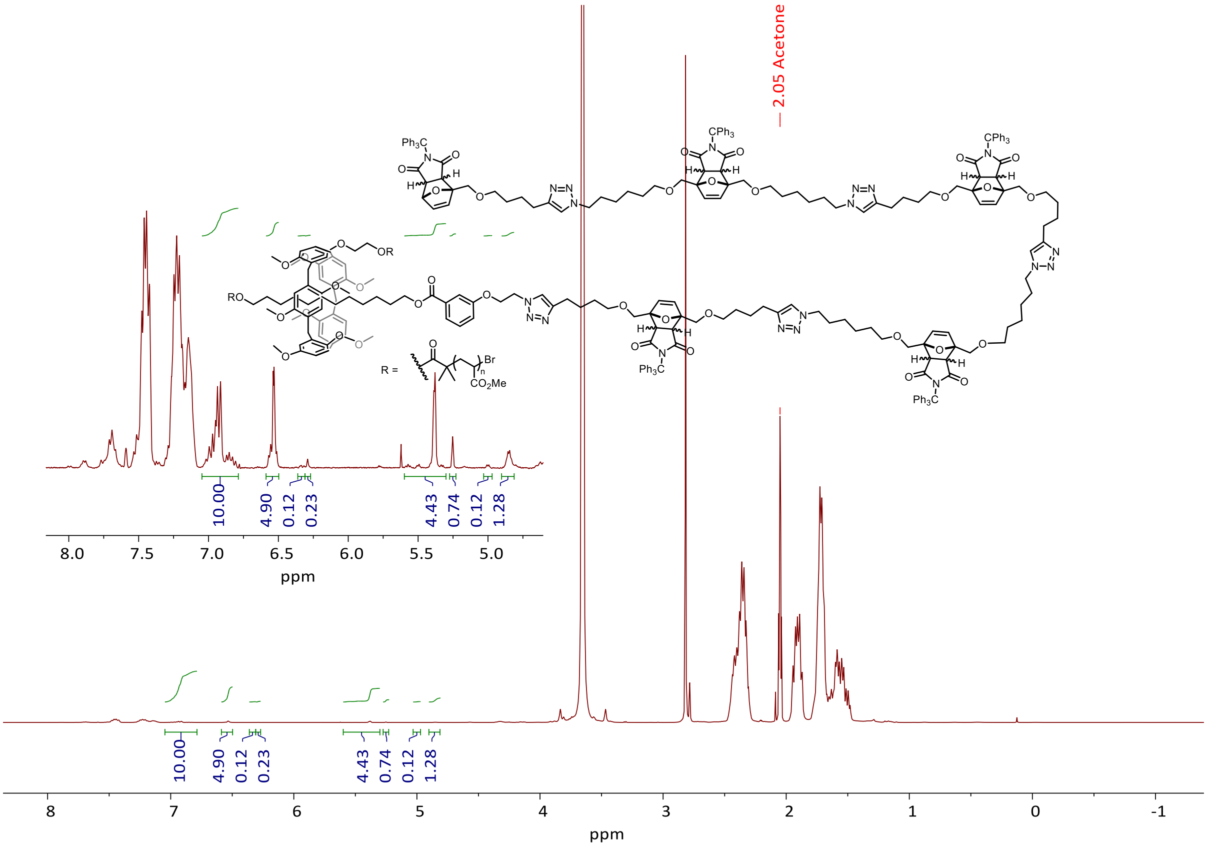

Spectrum S175. ^{1}H NMR (400 MHz, Acetone-d_6, 298 K) spectrum of polymer $\mathbf{1}_{5\text{-}165}$.

9.2.16 Spectra of polymer $1_{5\text{-}215}$

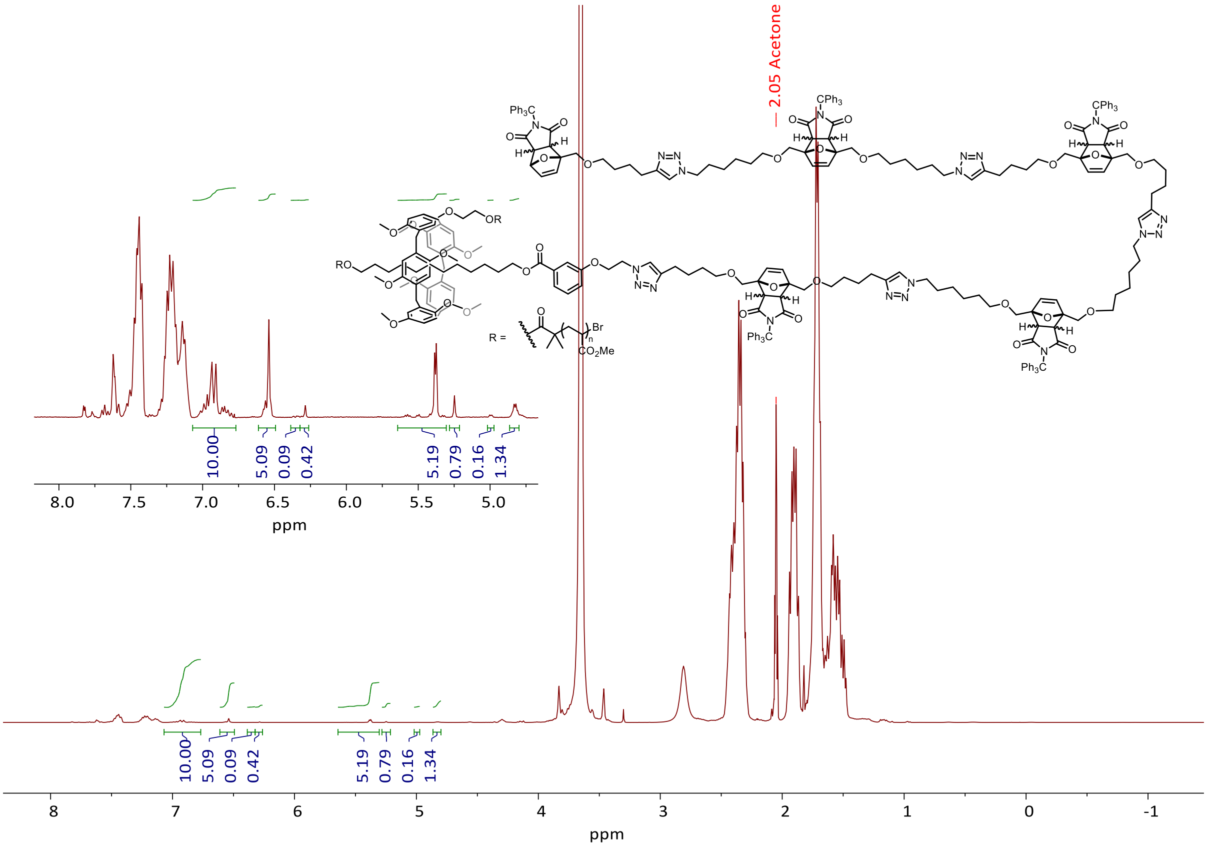

Spectrum S176. ^{1}H NMR (400 MHz, Acetone-d_6, 298 K) spectrum of polymer $\mathbf{1}_{5\text{-}215}$.

9.2.17 Spectra of polymer 3_{ref}

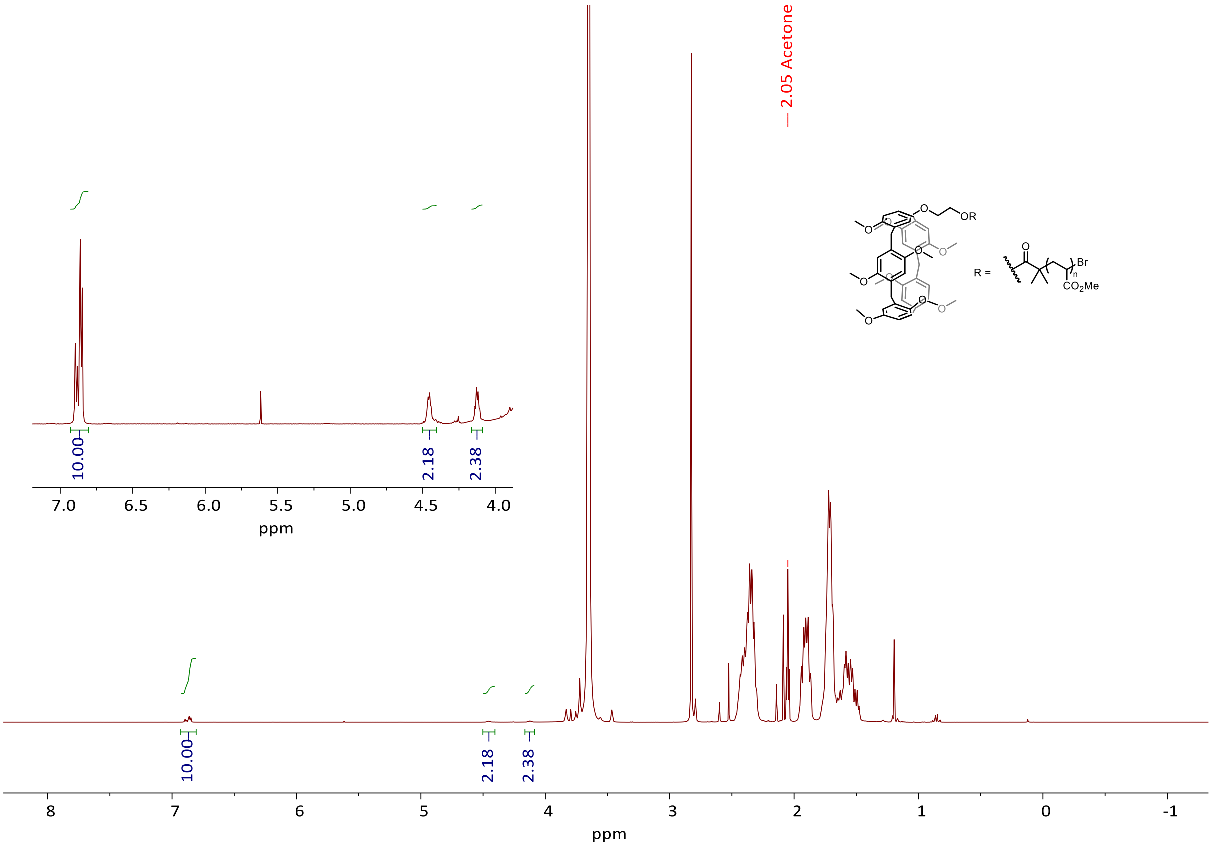

Spectrum S177. ^{1}H NMR (400 MHz, Acetone-d_6, 298 K) spectrum of polymer $\mathbf{3_{ref}}$.

9.2.18 Spectra of polymer S57-108

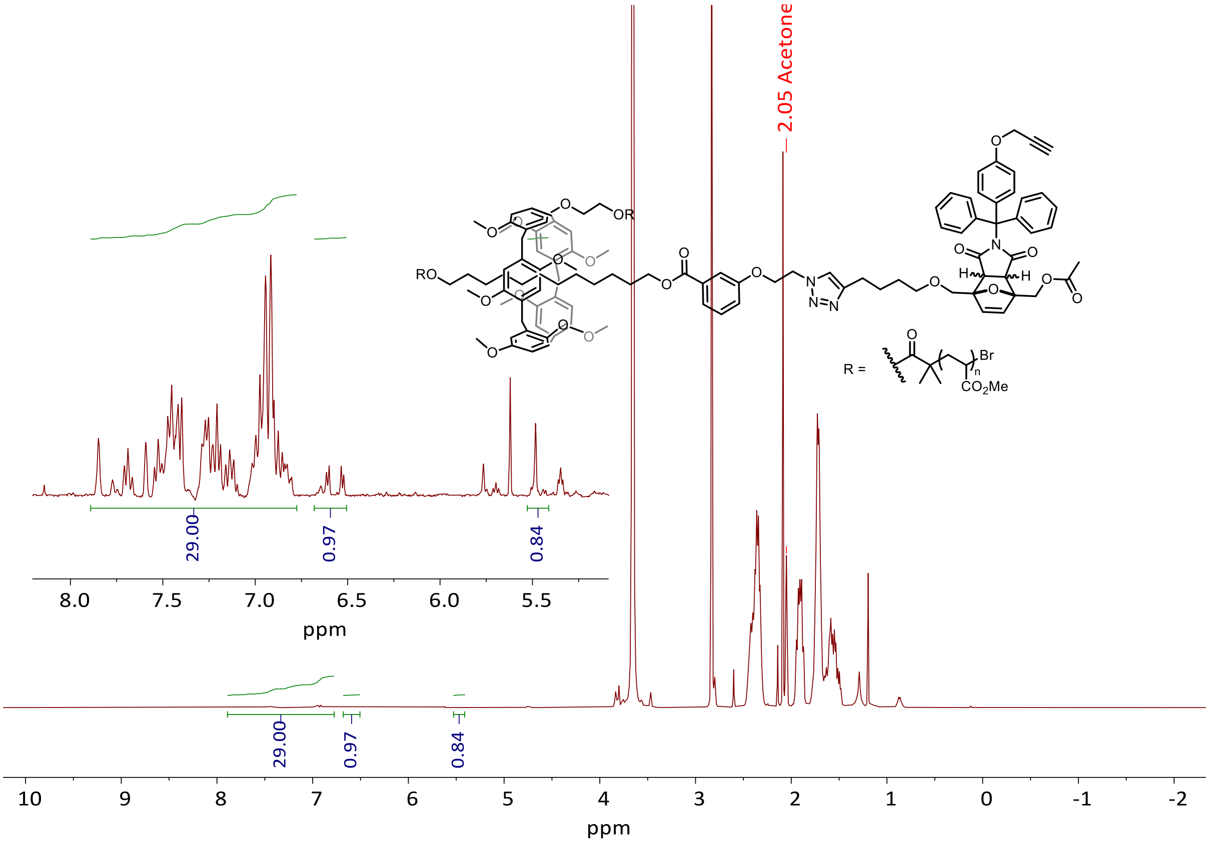

Spectrum S178. ^{1}H NMR (400 MHz, Acetone-d_6, 298 K) spectrum of polymer **S57-108**.

9.2.19 Spectra of polymer S58$_{-122}$

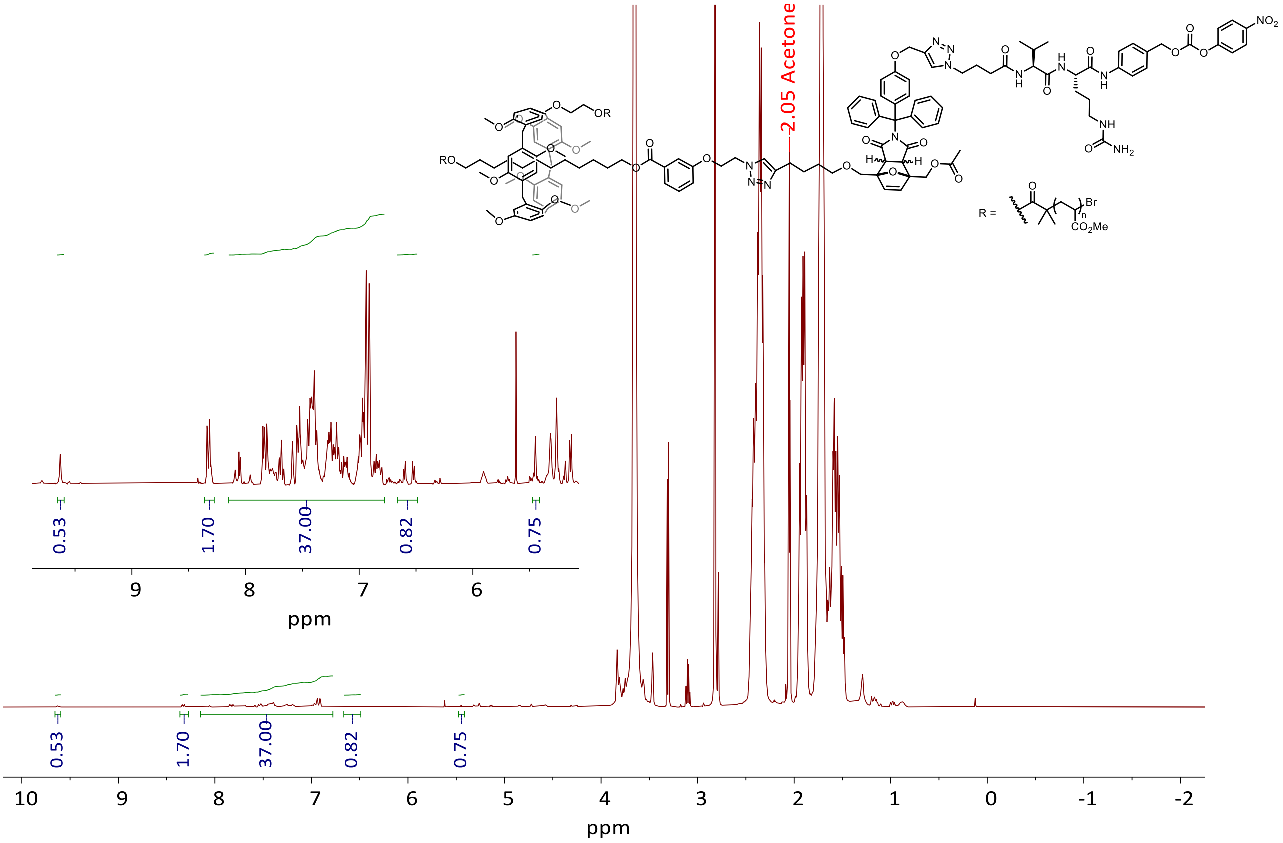

Spectrum S179. ^{1}H NMR (400 MHz, Acetone-d_6, 298 K) spectrum of **S58$_{-122}$**.

9.2.20 Spectra of polymer 11_{-127}

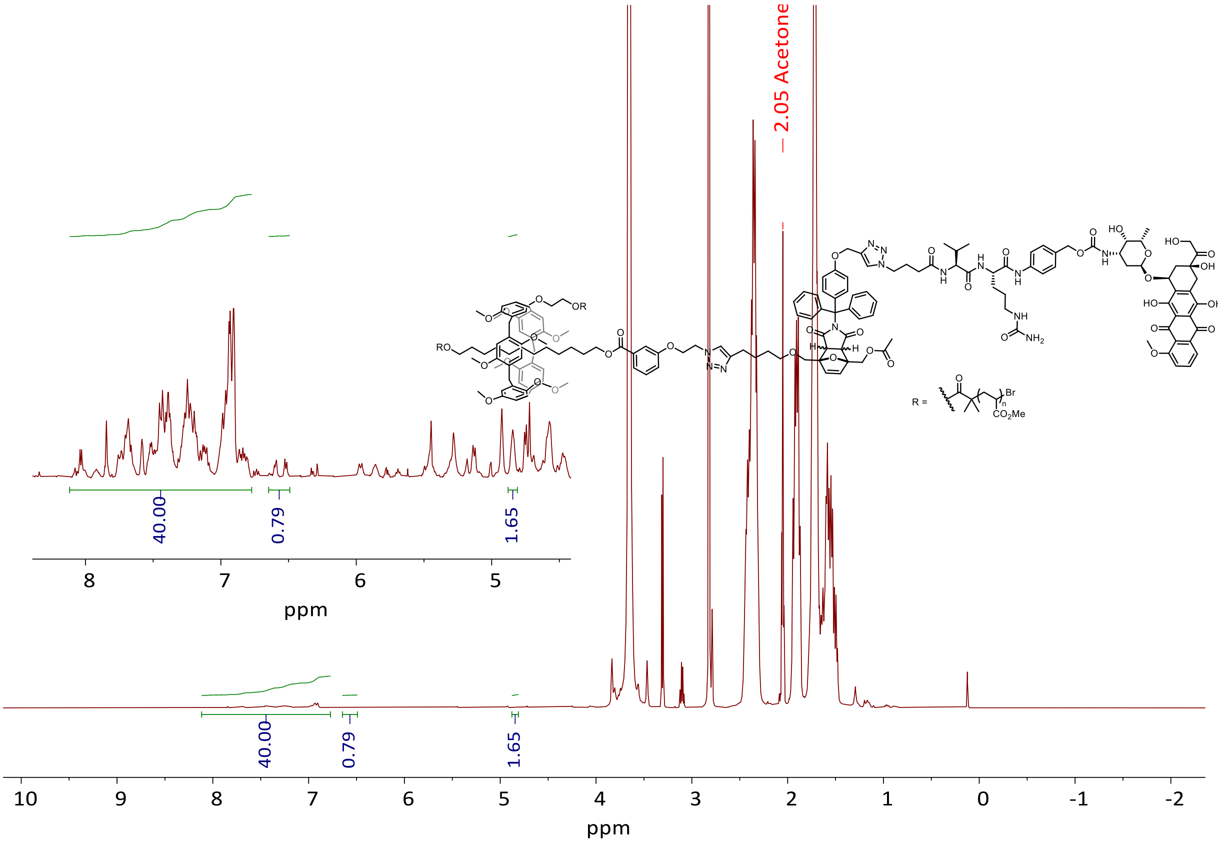

Spectrum S180. ^{1}H NMR (400 MHz, Acetone-d_6, 298 K) spectrum of polymer **11_{-127}**.

9.2.21 Spectra of polymer 13-119

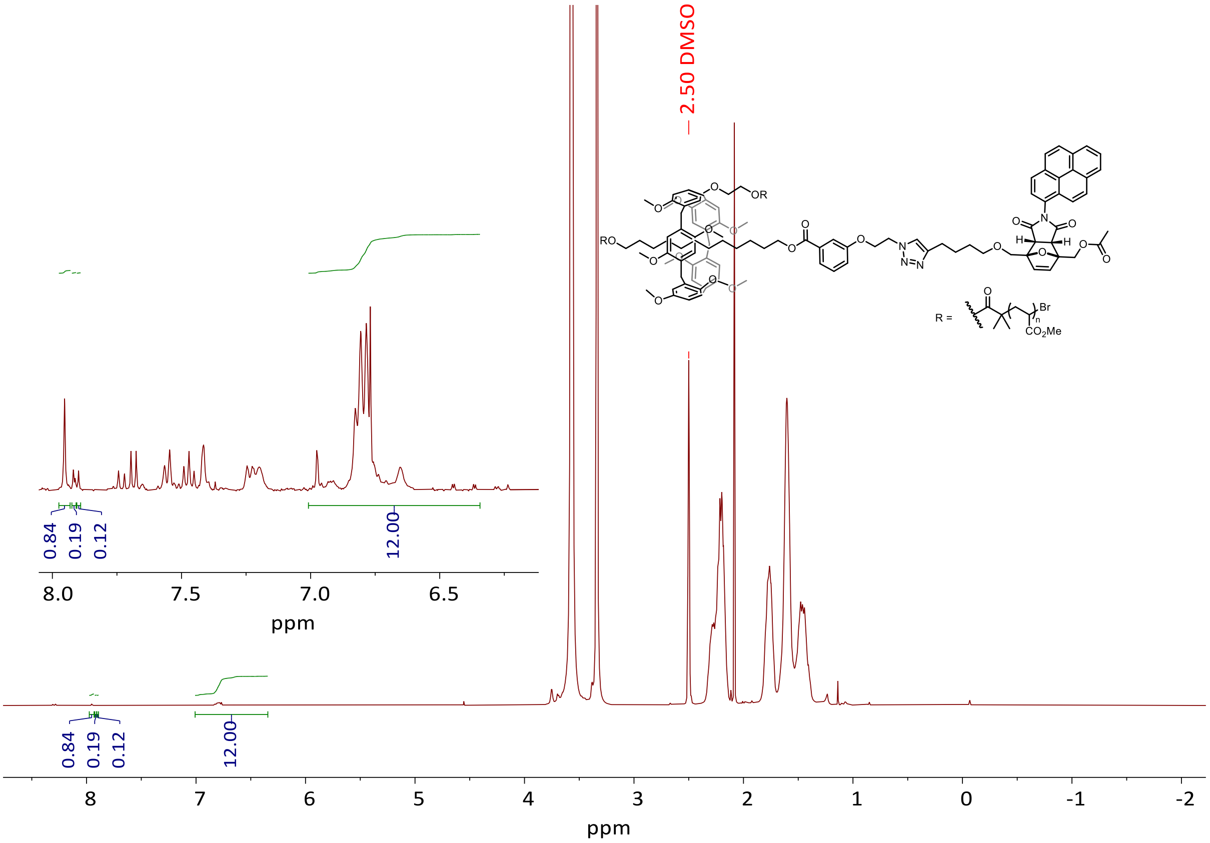

Spectrum S181. ^{1}H NMR (400 MHz, DMSO-d_6, 298 K) spectrum of polymer **13-119**.

9.2.22 Spectra of polymer S59-77

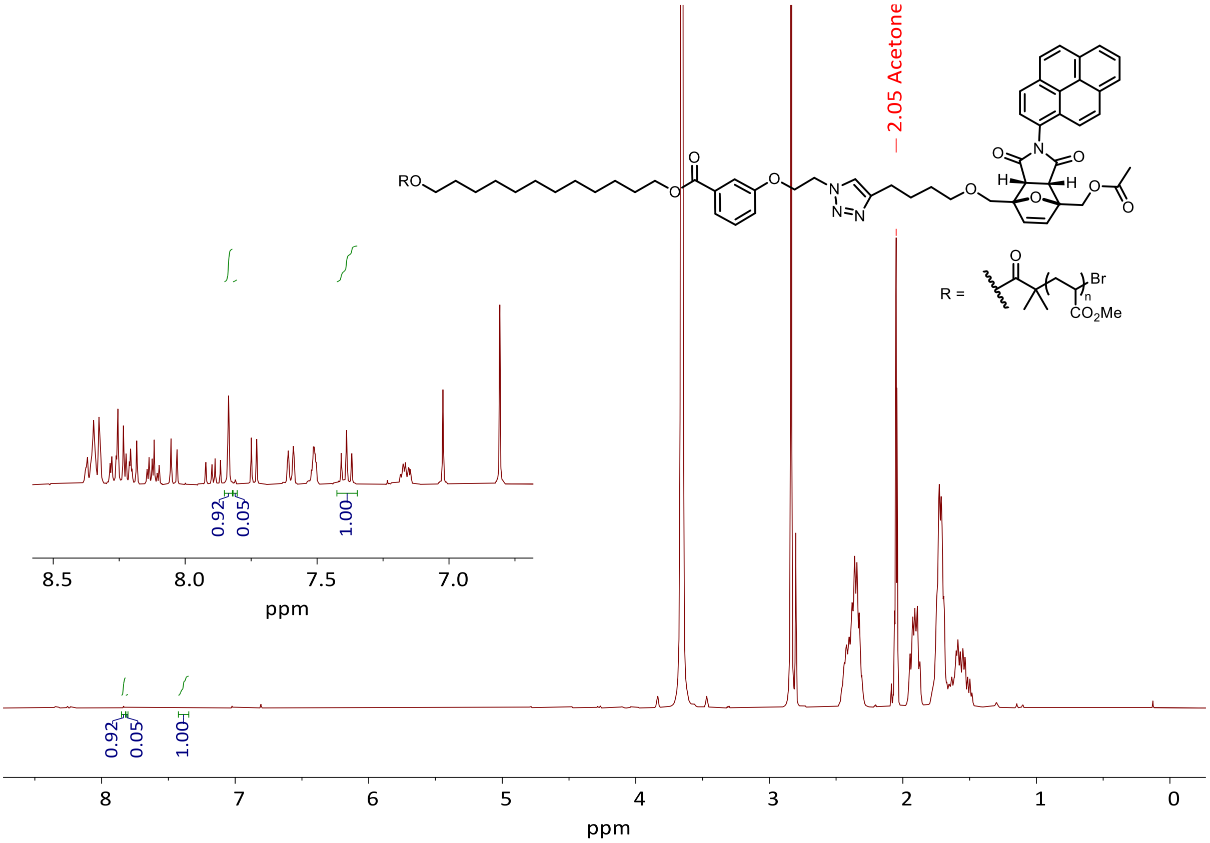

Spectrum S182. ^{1}H NMR (400 MHz, Acetone-d_6, 298 K) spectrum of polymer **S59-77**.

9.2.23 Spectra of polymer 10_{-80}

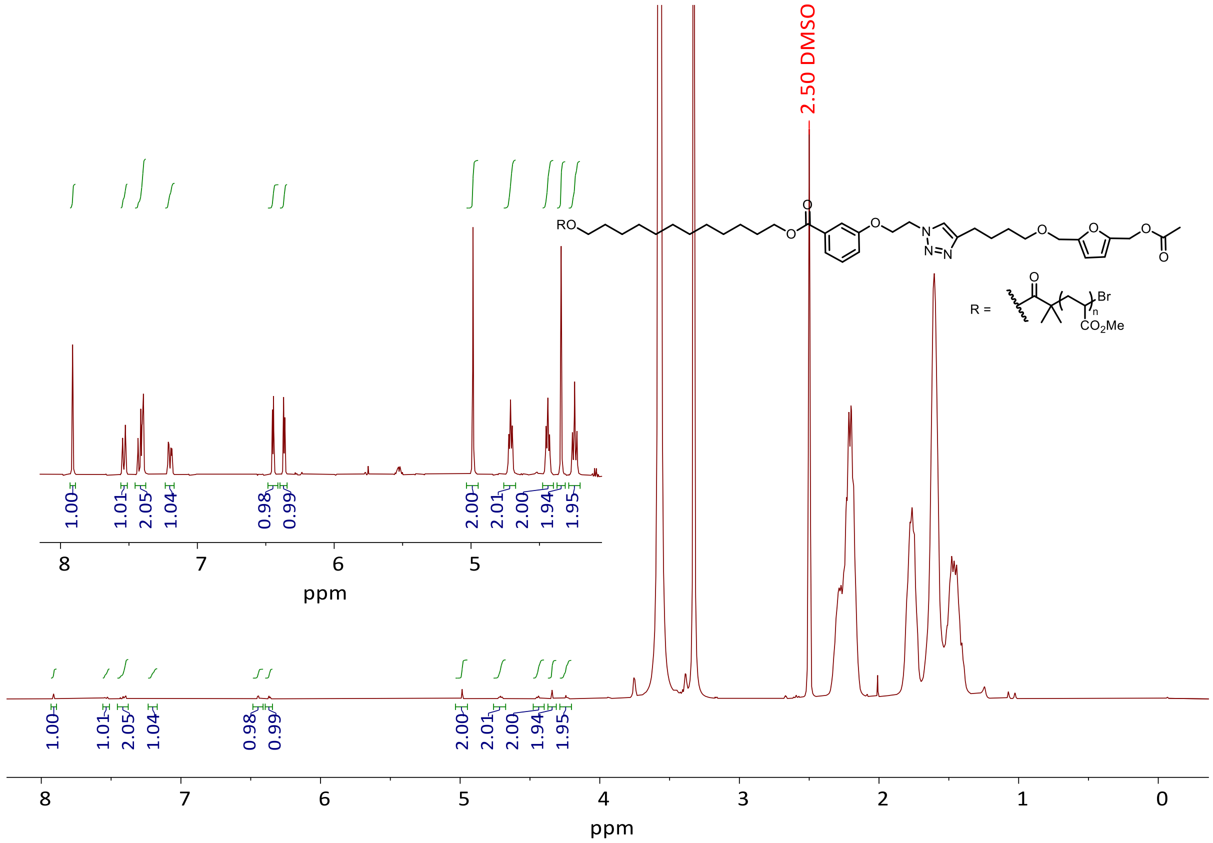

Spectrum S183. ^{1}H NMR (400 MHz, DMSO-d_6, 298 K) spectrum of polymer $\mathbf{10_{-80}}$.

9.2.24 Spectra of polymer 14-124

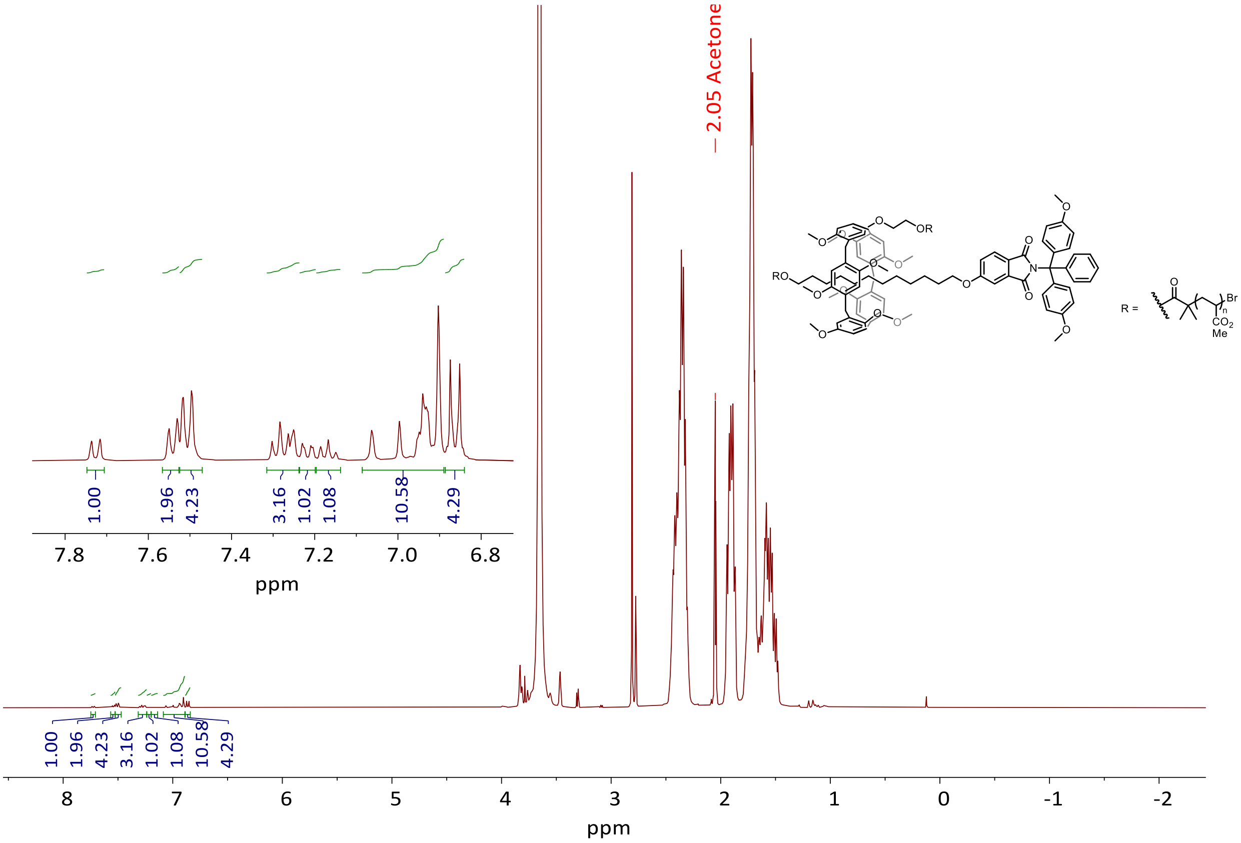

Spectrum S184. ^{1}H NMR (400 MHz, Acetone-d_6, 298 K) spectrum of polymer **14-124**.

9.2.25 Spectra of polymer S60-95

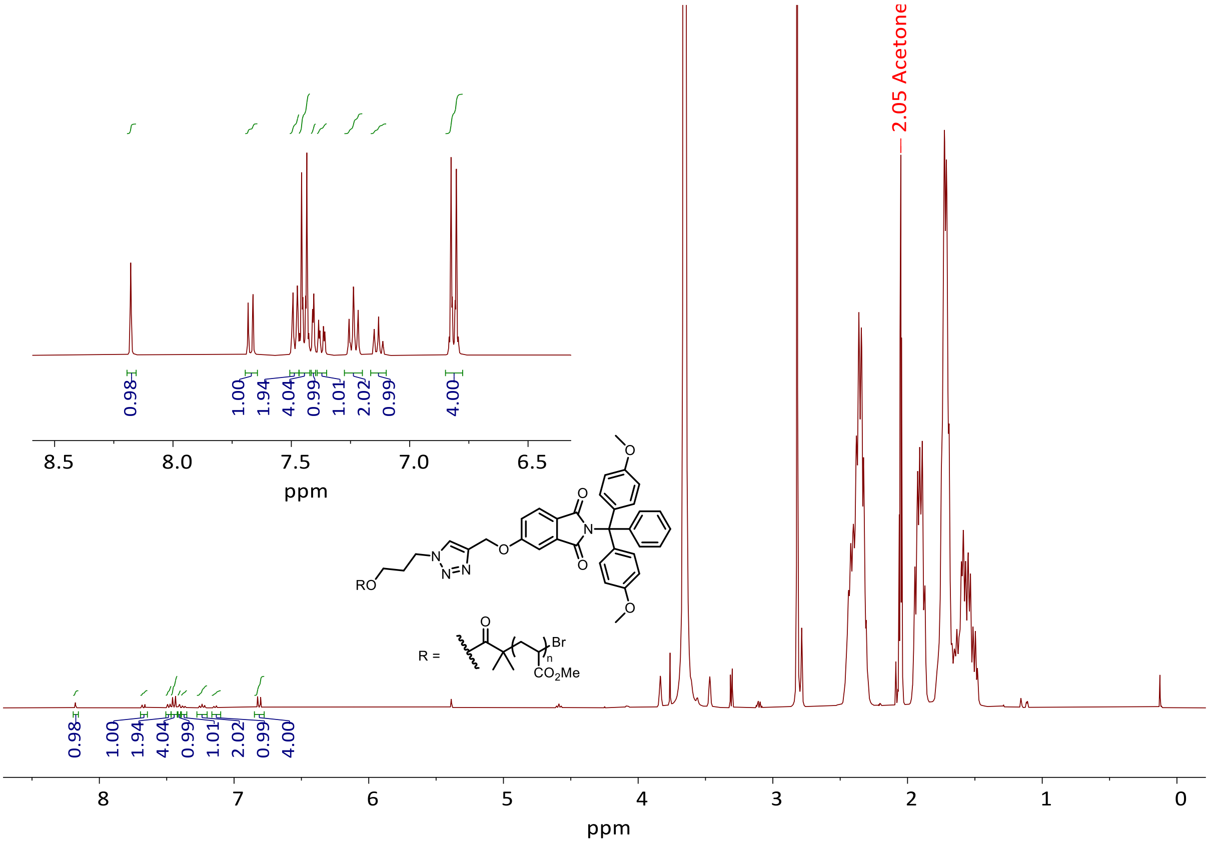

Spectrum S185. ^{1}H NMR (400 MHz, Acetone-d_6, 298 K) spectrum of polymer **S60-95**.

9.2.26 Spectra of polymer S61$_{-72}$

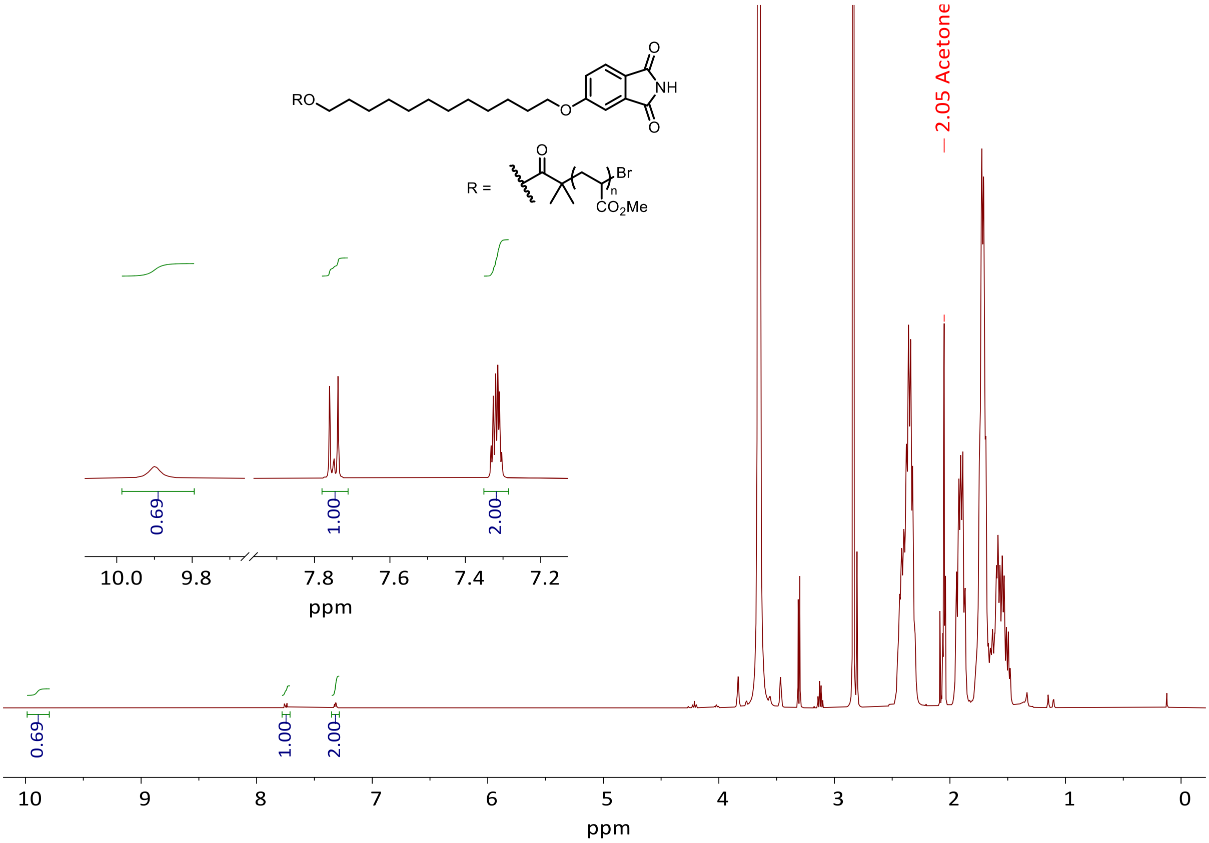

Spectrum S186. ^{1}H NMR (400 MHz, Acetone-d_6, 298 K) spectrum of polymer **S61$_{-72}$**.

9.3 Post-Sonication NMR Spectra

9.3.1 Post-Sonication ^{1}H NMR Spectra of Polymer S55$_{exo\text{-}112}$

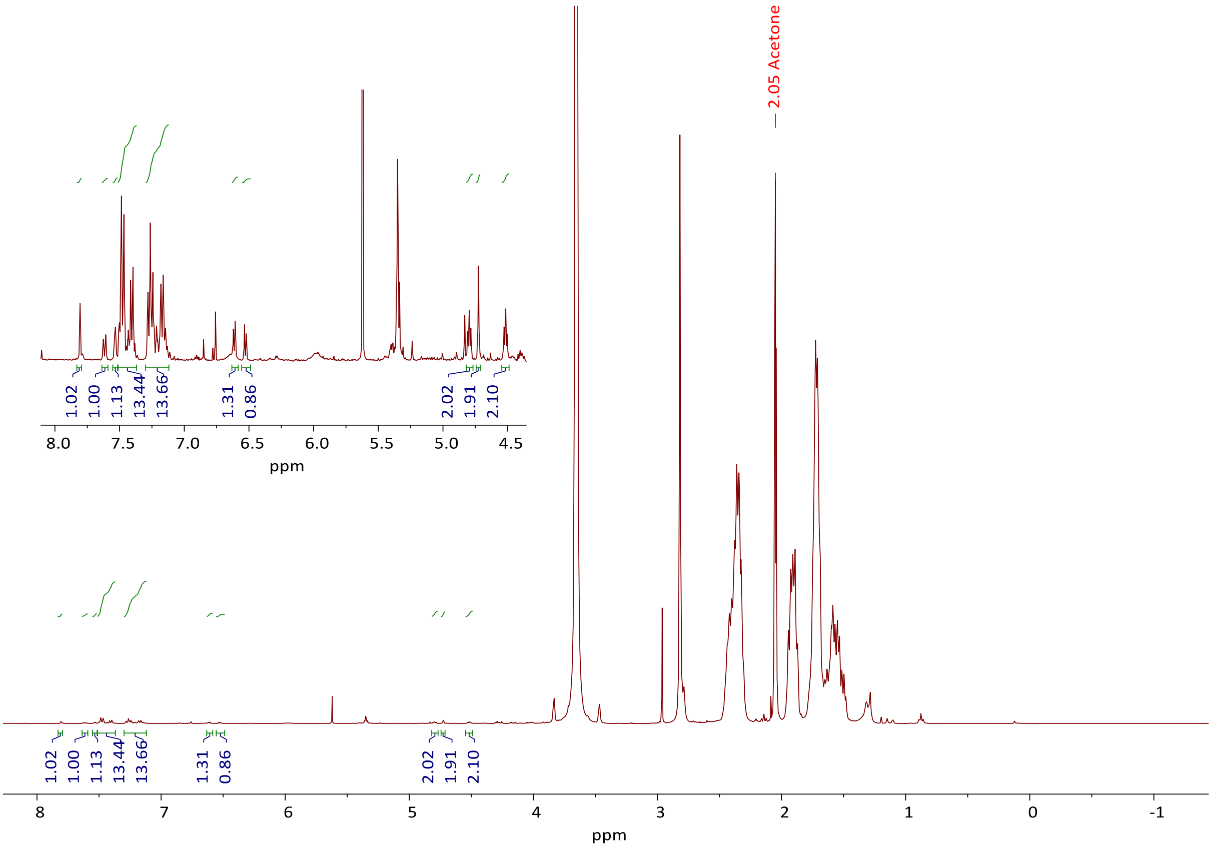

Spectrum S187. ^{1}H NMR (400 MHz, Acetone-d_6, 298 K) spectrum of post-sonication polymer **S55$_{exo\text{-}112}$** before being washed with methanol.

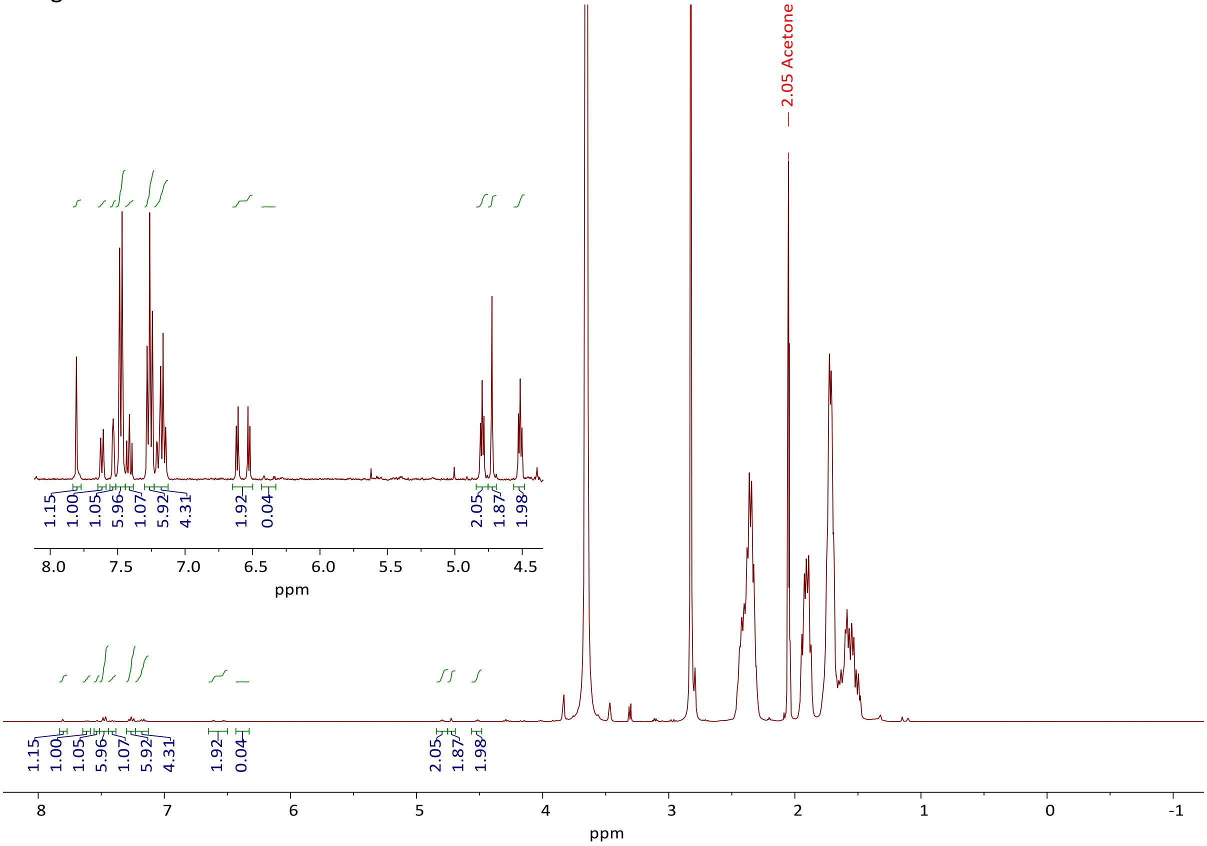

Spectrum S188. ^{1}H NMR (400 MHz, Acetone-d_6, 298 K) spectrum of post-sonication polymer **S55$_{exo\text{-}112}$** after being washed with methanol.

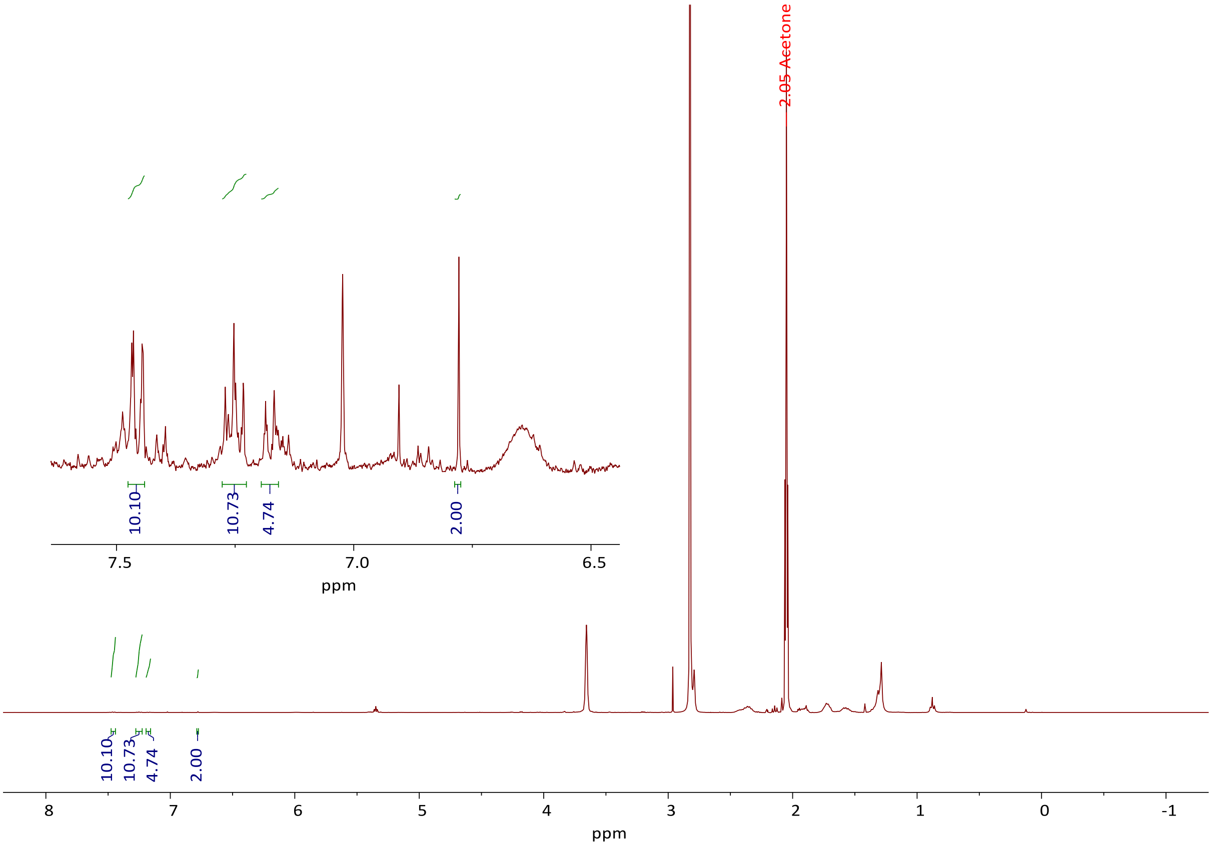

Spectrum S189. ^{1}H NMR (400 MHz, Acetone-d_6, 298 K) spectrum of the concentrated methanol washings from post-sonication polymer **S55$_{exo\text{-}112}$**.

9.3.2 Post-Sonication ^{1}H NMR Spectra of Polymer S55$_{endo\text{-}65}$

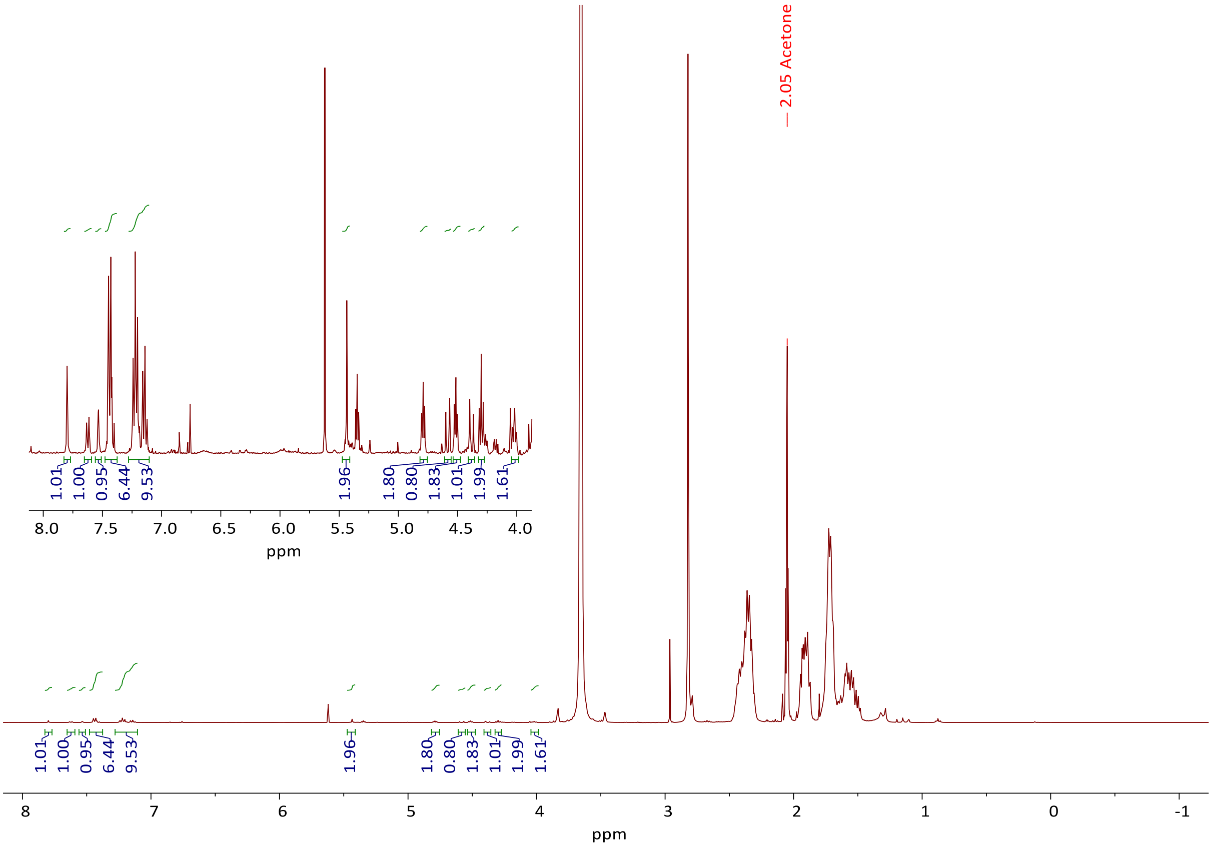

Spectrum S190. ^{1}H NMR (400 MHz, Acetone-d_6, 298 K) spectrum of post-sonication polymer **S55$_{endo\text{-}65}$** before being washed with methanol.

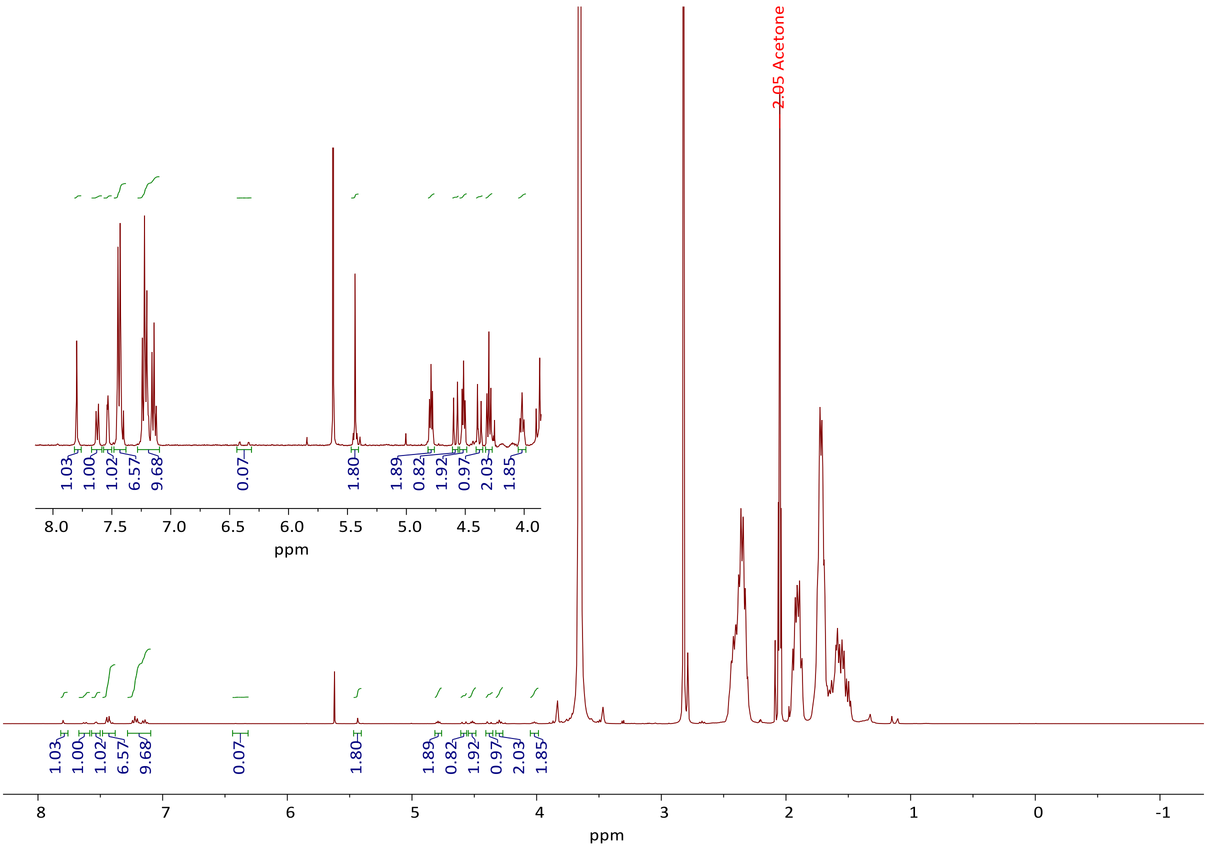

Spectrum S191. ^{1}H NMR (400 MHz, Acetone-d_6, 298 K) spectrum of post-sonication polymer **S55*endo*-65** after being washed with methanol.

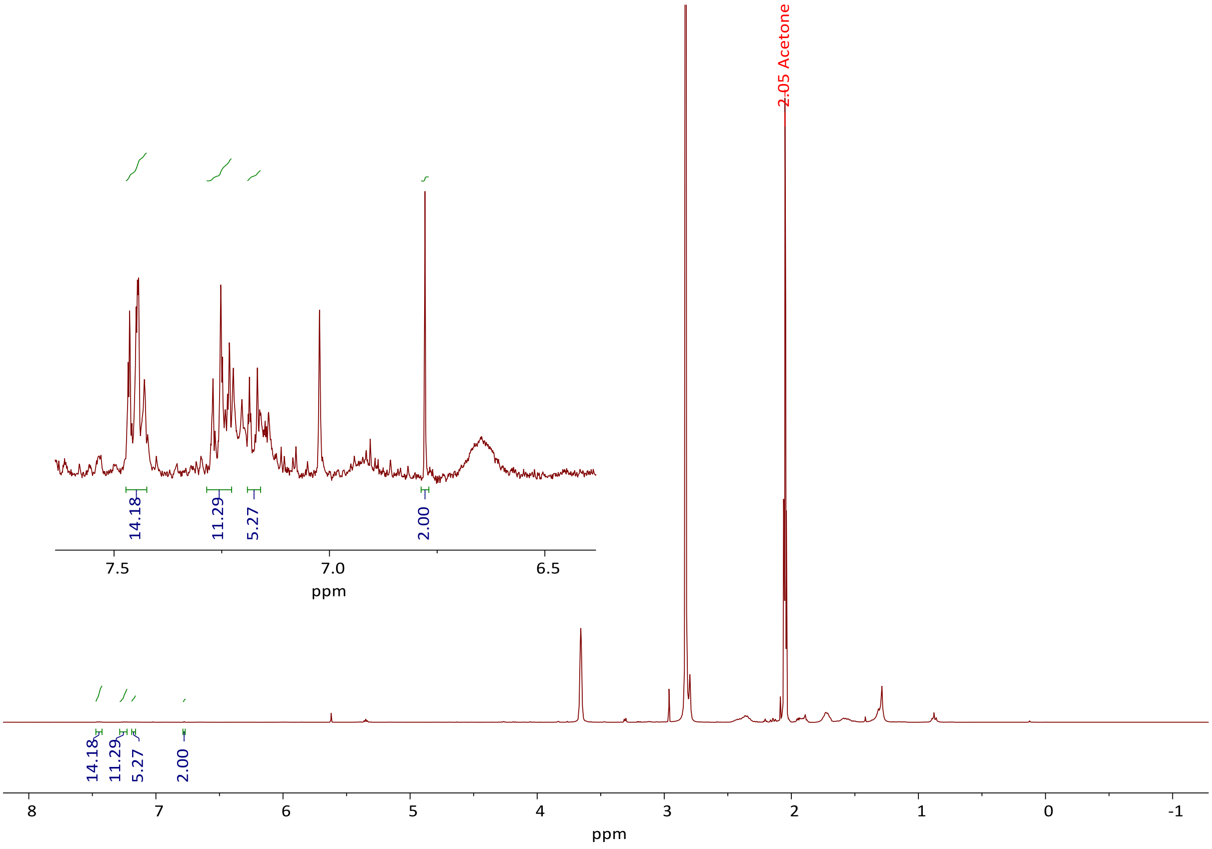

Spectrum S192. ^{1}H NMR (400 MHz, Acetone-d_6, 298 K) spectrum of the concentrated methanol washings from post-sonication polymer **S55*endo*-65**.

9.3.3 Post-Sonication ^{1}H NMR Spectra of Polymer 9$_{trans/exo}$-109

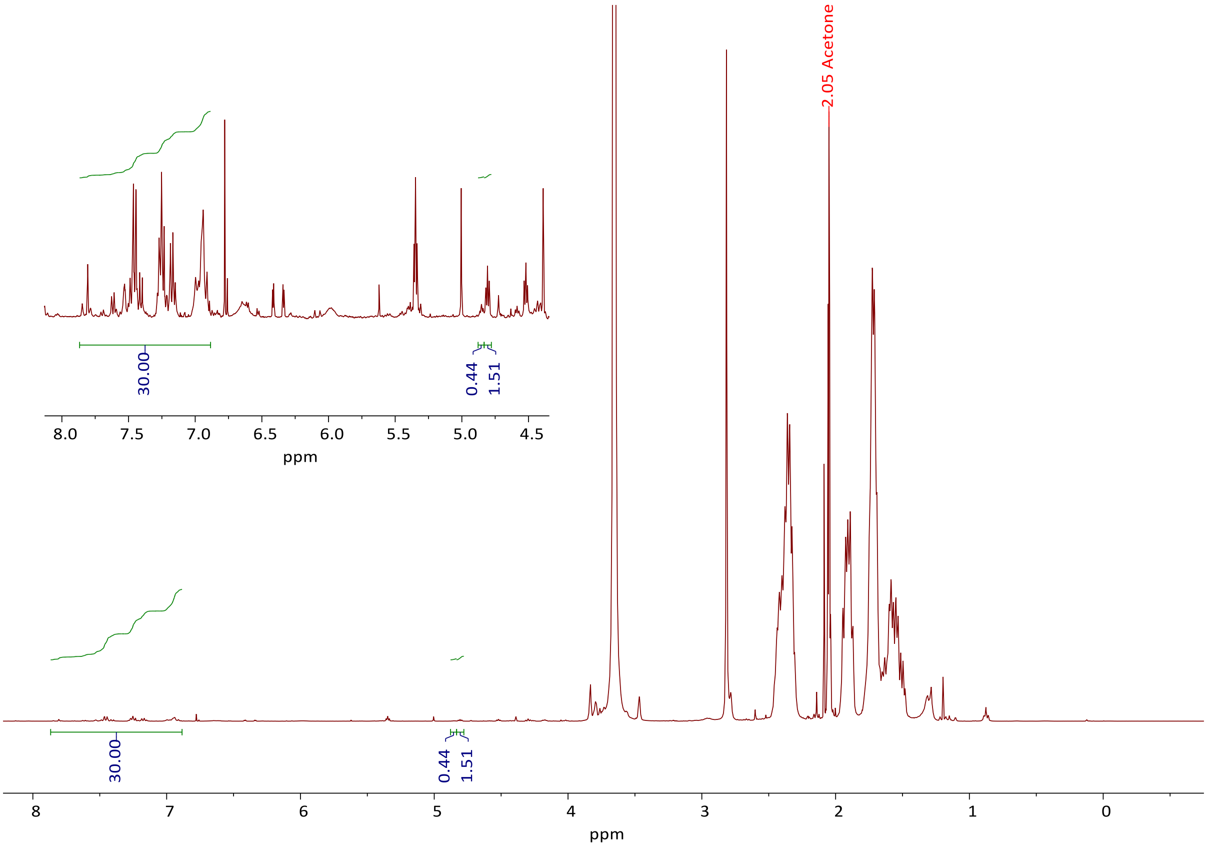

Spectrum S193. ^{1}H NMR (400 MHz, Acetone-d_6, 298 K) spectrum of post-sonication polymer **9**$_{trans/exo\text{-}109}$ before being washed with methanol.

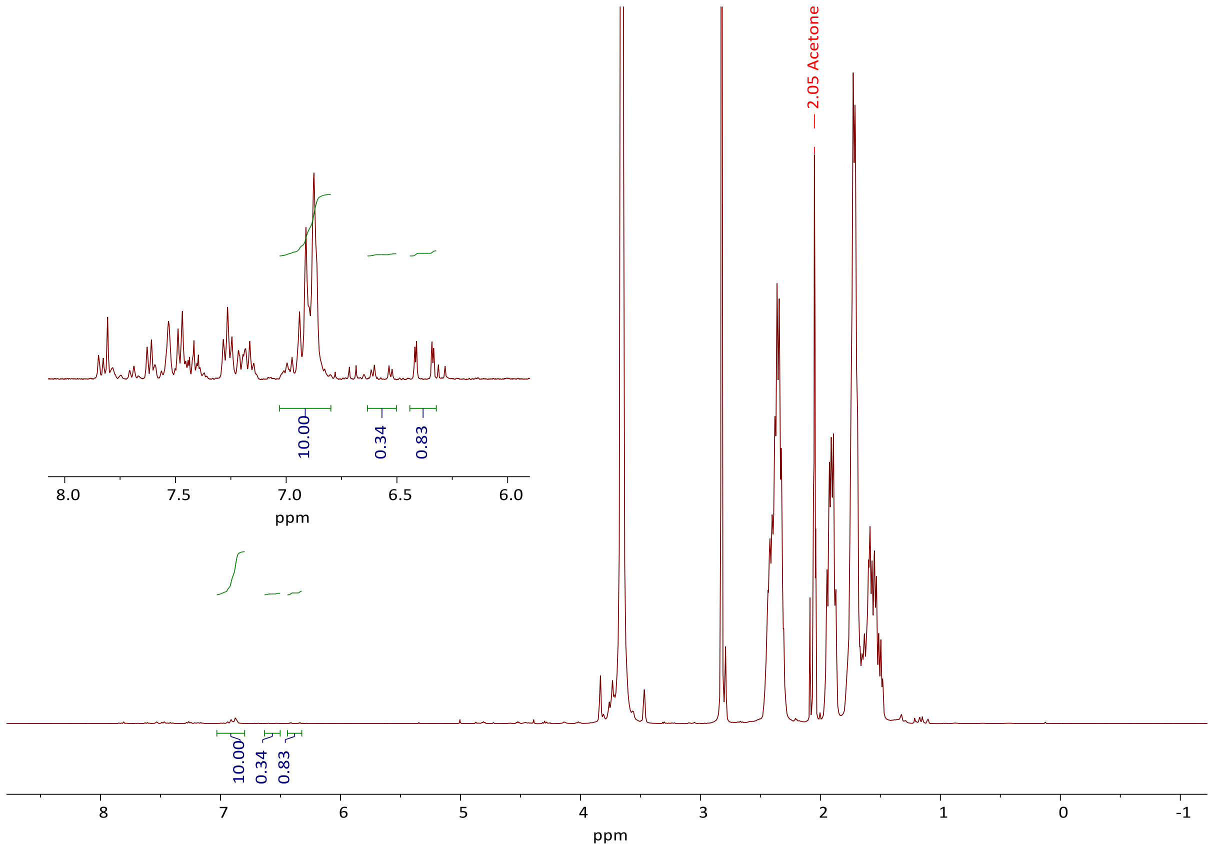

Spectrum S194. ^{1}H NMR (400 MHz, Acetone-d_6, 298 K) spectrum of post-sonication polymer $\mathbf{9}_{\textit{trans/exo}\text{-}109}$ after being washed with methanol.

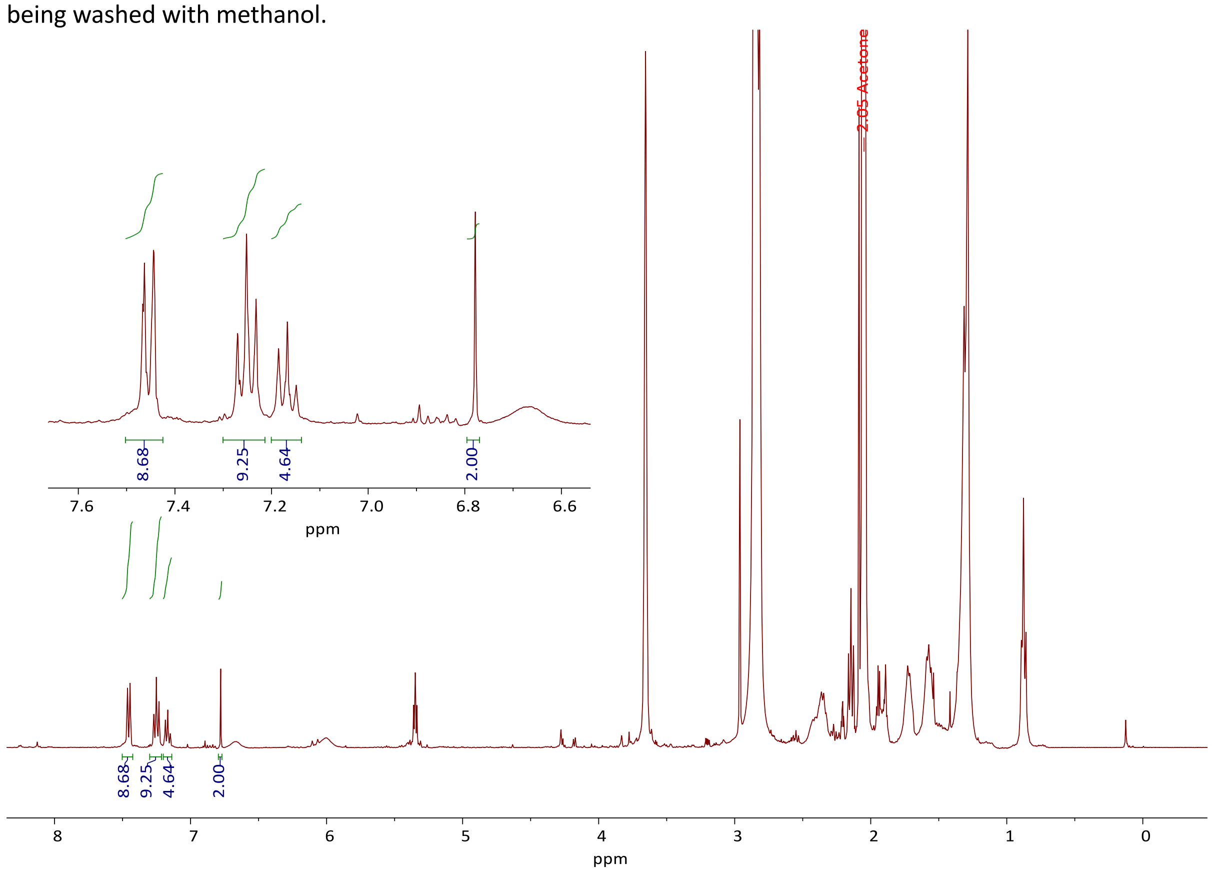

Spectrum S195. ^{1}H NMR (400 MHz, Acetone-d_6, 298 K) spectrum of the concentrated methanol washings from post-sonication polymer $\mathbf{9}_{\textit{trans/exo}\text{-}109}$.

9.3.4 Post-Sonication ^{1}H NMR Spectra of Polymer $\mathbf{9}_{cis/exo\text{-}114}$

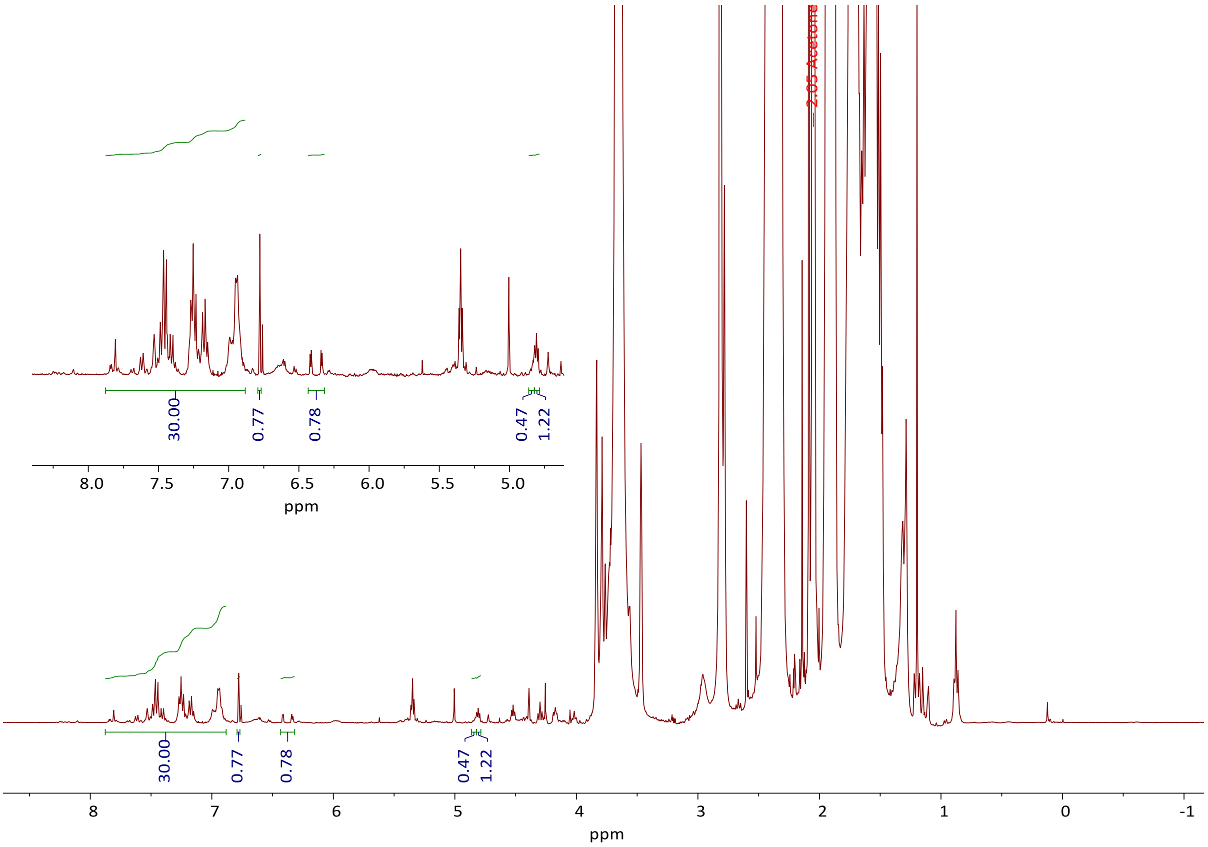

Spectrum S196. ^{1}H NMR (400 MHz, Acetone-d_6, 298 K) spectrum of post-sonication polymer $\mathbf{9}_{cis/exo\text{-}114}$ before being washed with methanol.

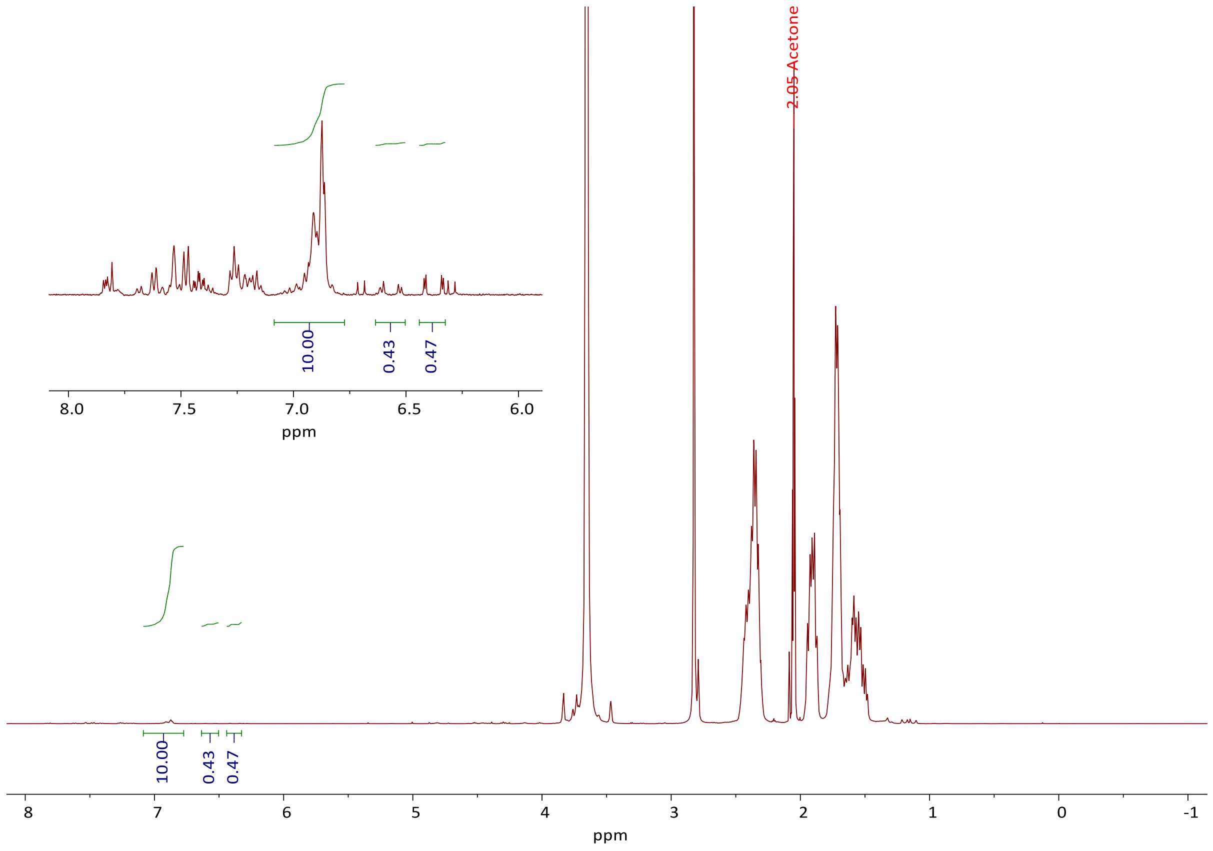

Spectrum S197. ^{1}H NMR (400 MHz, Acetone-d_6, 298 K) spectrum of post-sonication polymer $\mathbf{9}_{cis/exo\text{-}114}$ after being washed with methanol.

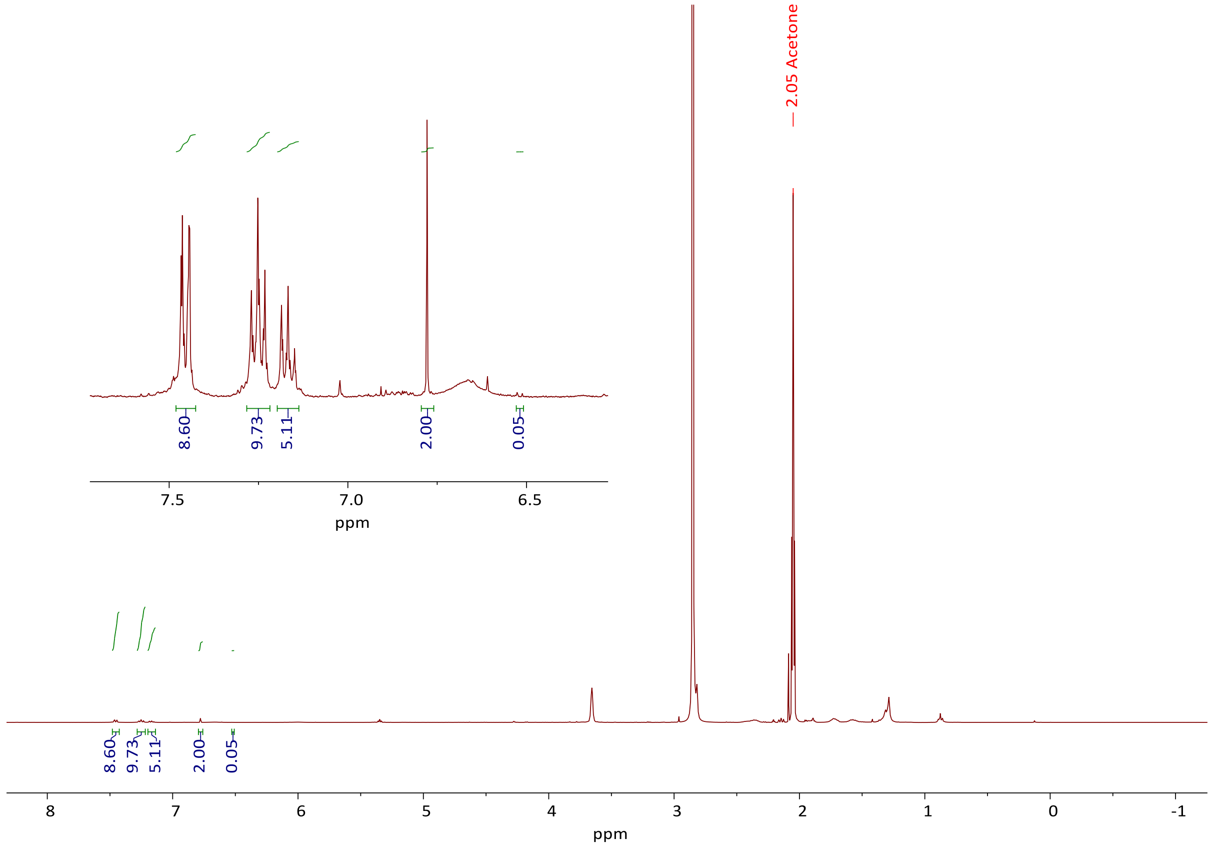

Spectrum S198. ^{1}H NMR (400 MHz, Acetone-d_6, 298 K) spectrum of the concentrated methanol washings from post-sonication polymer $\mathbf{9}_{cis/exo\text{-}114}$.

9.3.5 Post-Sonication ^{1}H NMR Spectra of Polymer $9_{trans/endo\text{-}90}$

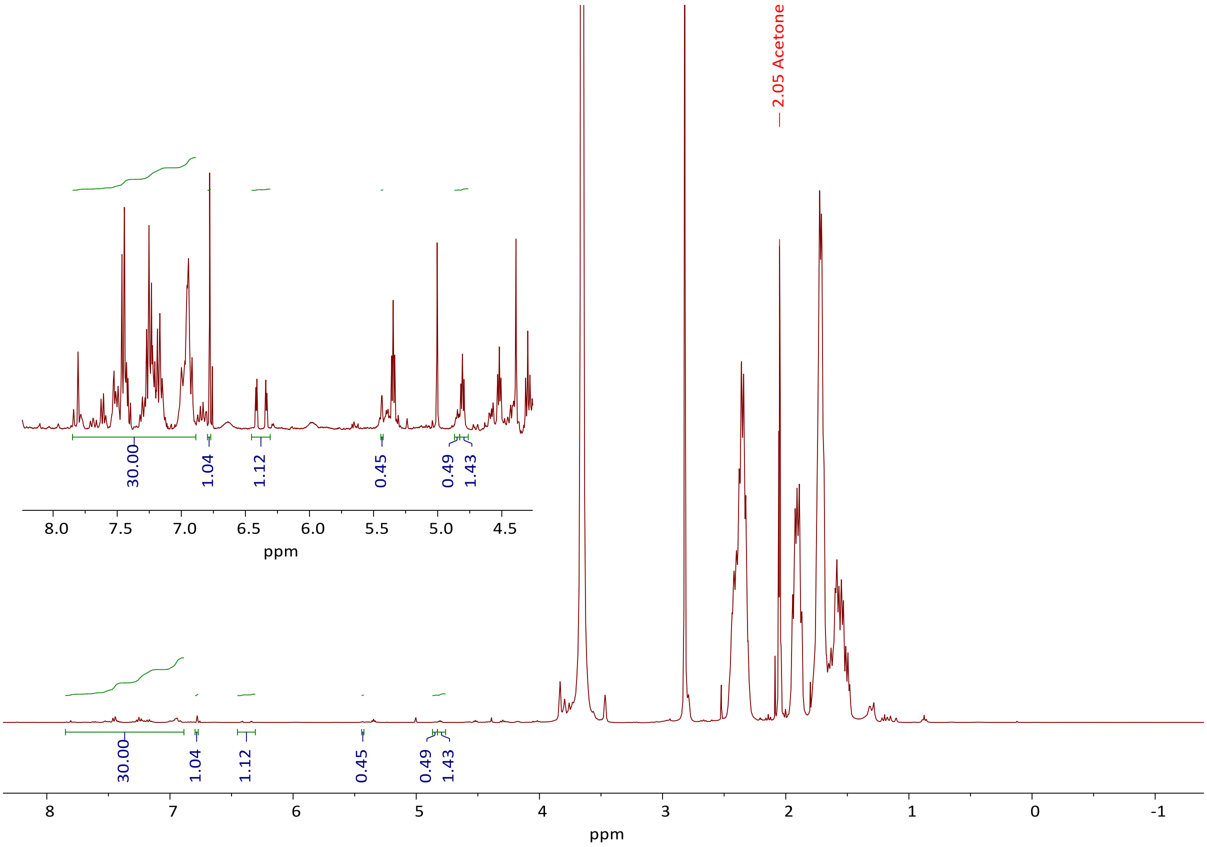

Spectrum S199. ^{1}H NMR (400 MHz, Acetone-d_6, 298 K) spectrum of post-sonication polymer $\mathbf{9}_{trans/endo\text{-}90}$ before being washed with methanol.

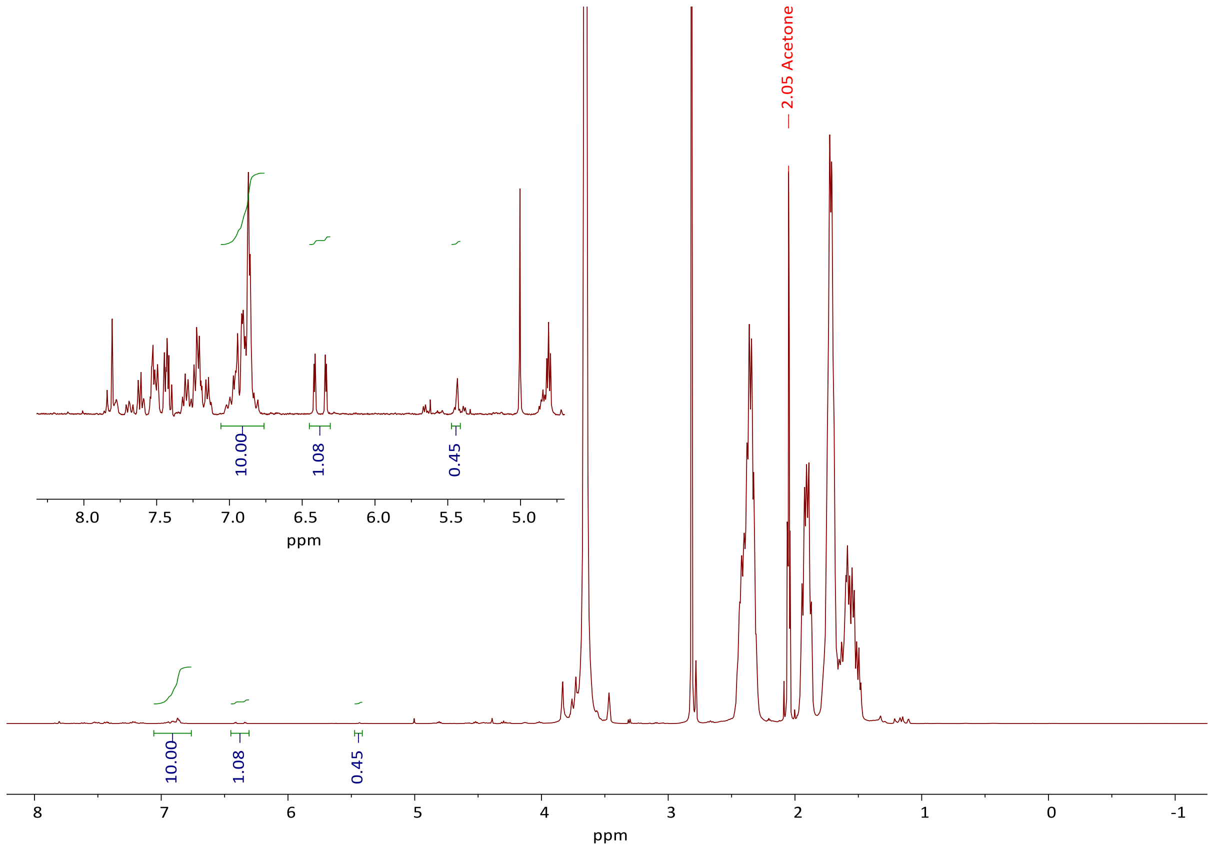

Spectrum S200. ^{1}H NMR (400 MHz, Acetone-d_6, 298 K) spectrum of post-sonication polymer **9*trans/endo*-90** after being washed with methanol.

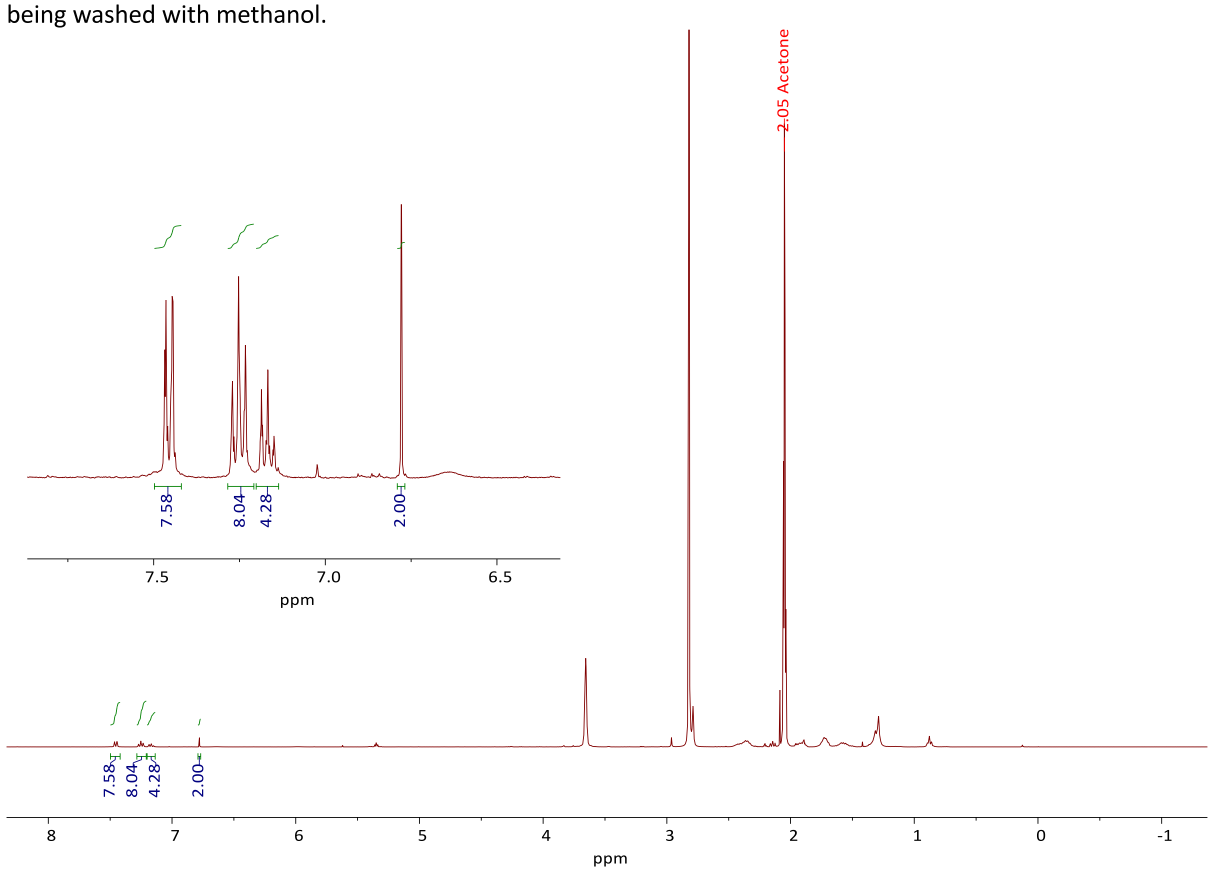

Spectrum S201. ^{1}H NMR (400 MHz, Acetone-d_6, 298 K) spectrum of the concentrated methanol washings from post-sonication polymer **9*trans/endo*-90**.

9.3.6 Post-Sonication ^{1}H NMR Spectra of Polymer $\mathbf{9}_{cis/endo\text{-}92}$

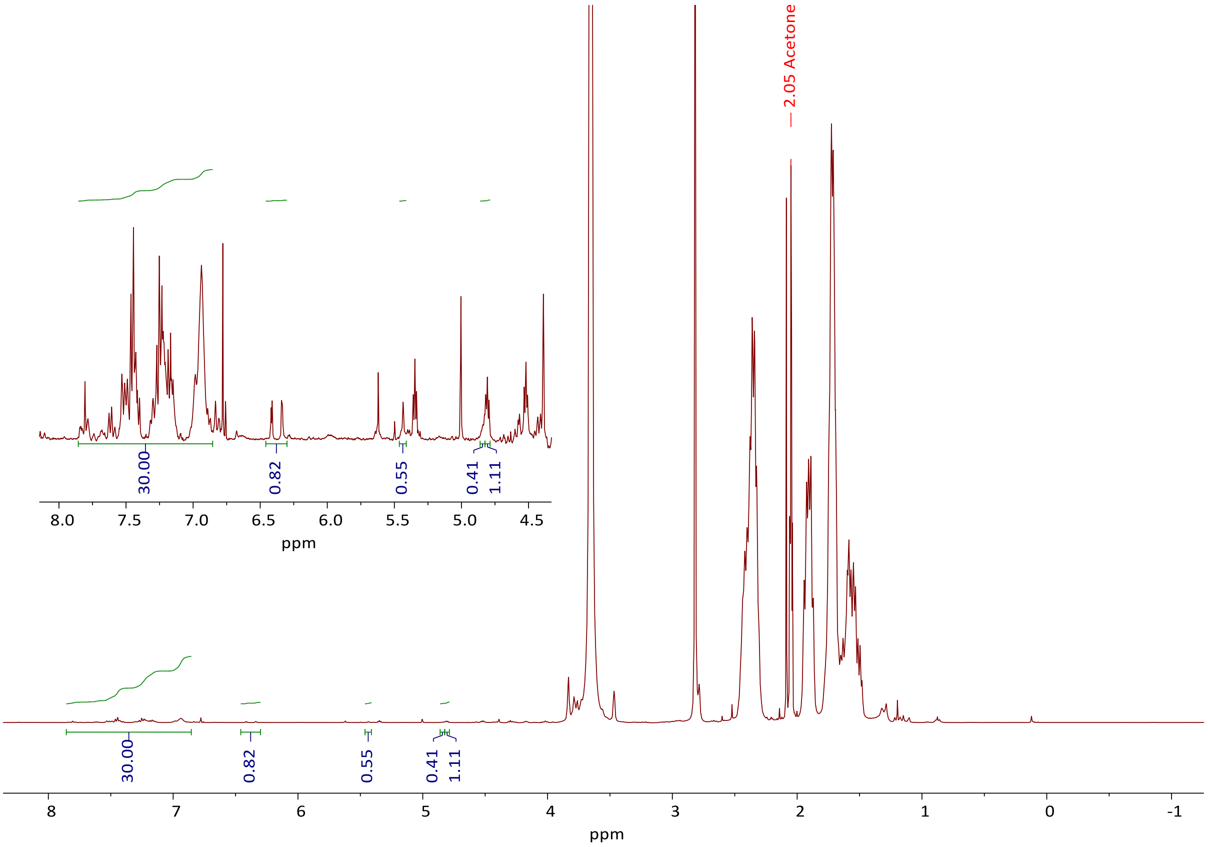

Spectrum S202. ^{1}H NMR (400 MHz, Acetone-d_6, 298 K) spectrum of post-sonication polymer $\mathbf{9}_{cis/endo\text{-}92}$ before being washed with methanol.

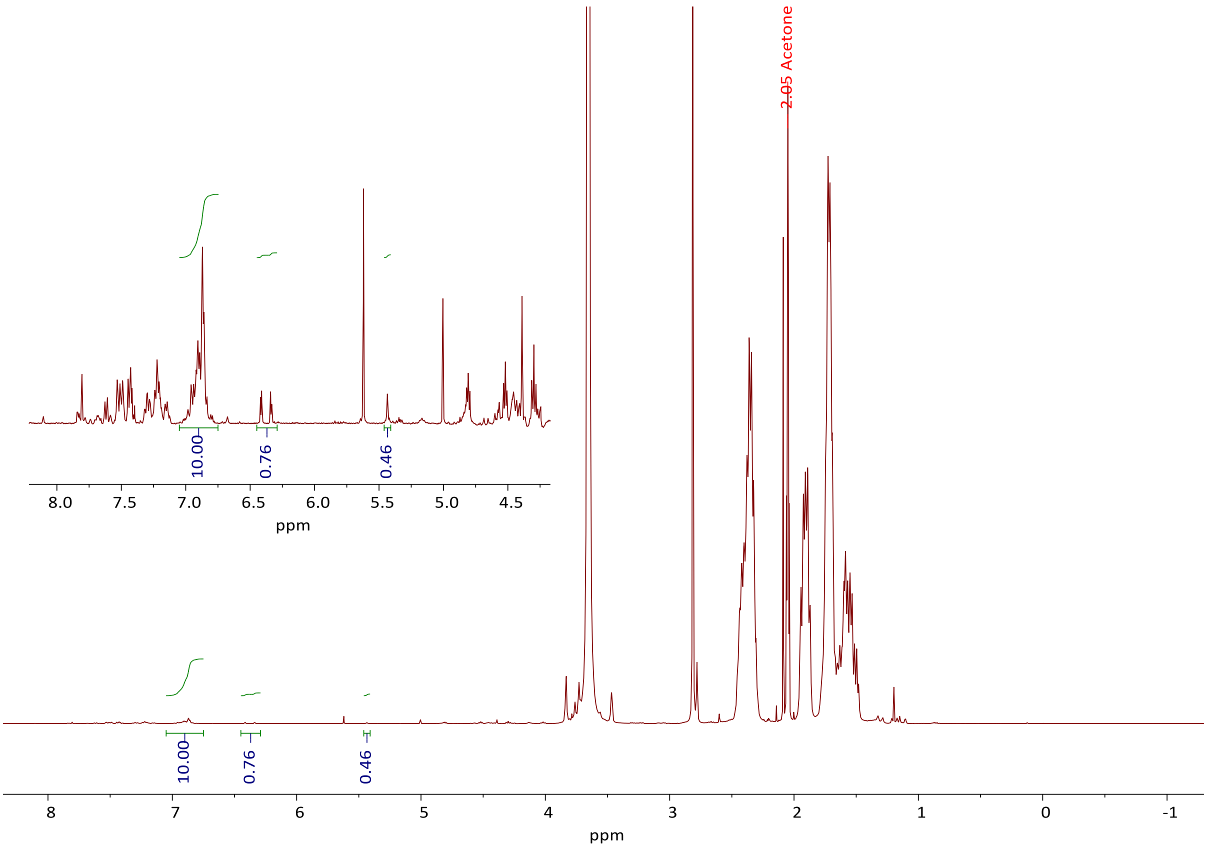

Spectrum S203. ^{1}H NMR (400 MHz, Acetone-d_6, 298 K) spectrum of post-sonication polymer **9**$_{\textbf{\textit{cis/endo}}\textbf{-92}}$ after being washed with methanol.

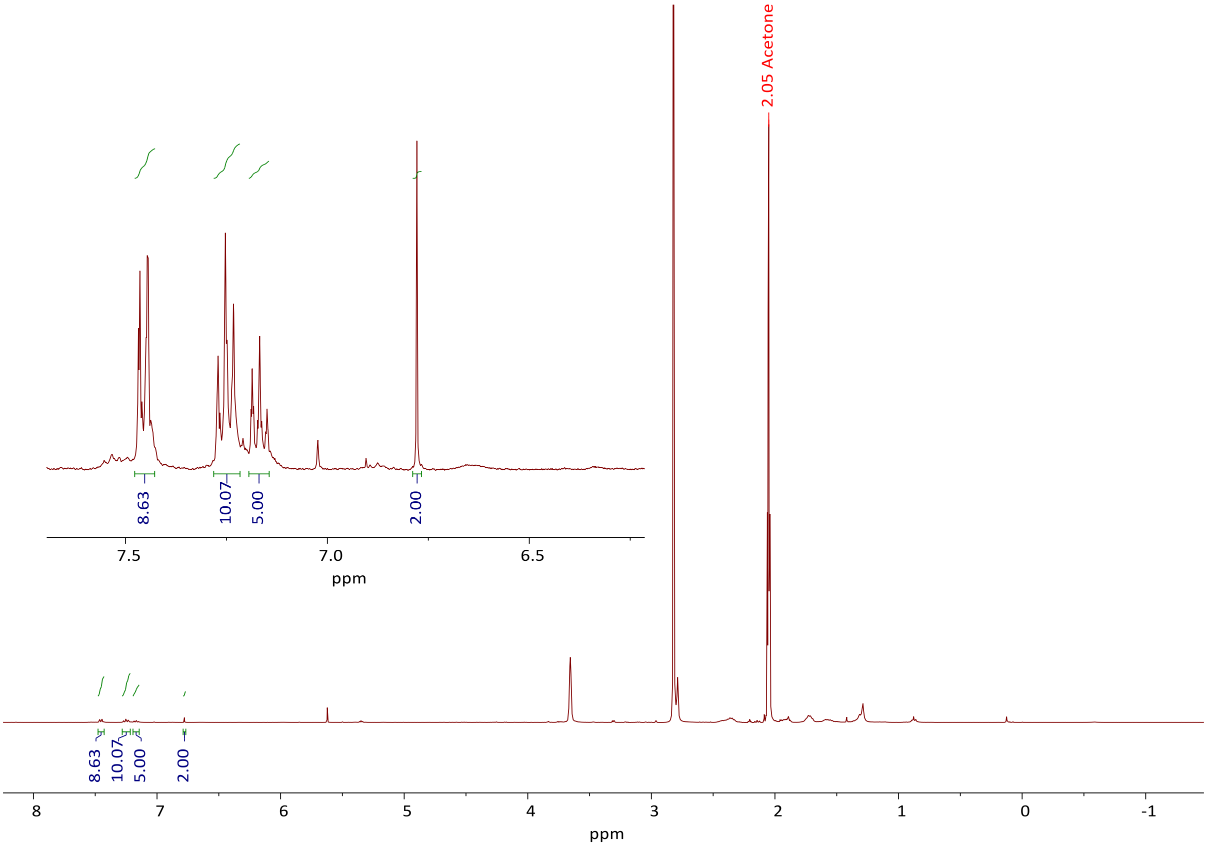

Spectrum S204. ^{1}H NMR (400 MHz, Acetone-d_6, 298 K) spectrum of the concentrated methanol washings from post-sonication polymer **9**$_{\textbf{\textit{cis/endo}}\textbf{-92}}$.

9.3.7 Post-Sonication ^{1}H NMR Spectra of Polymer 1$_3$d$_{-89}$

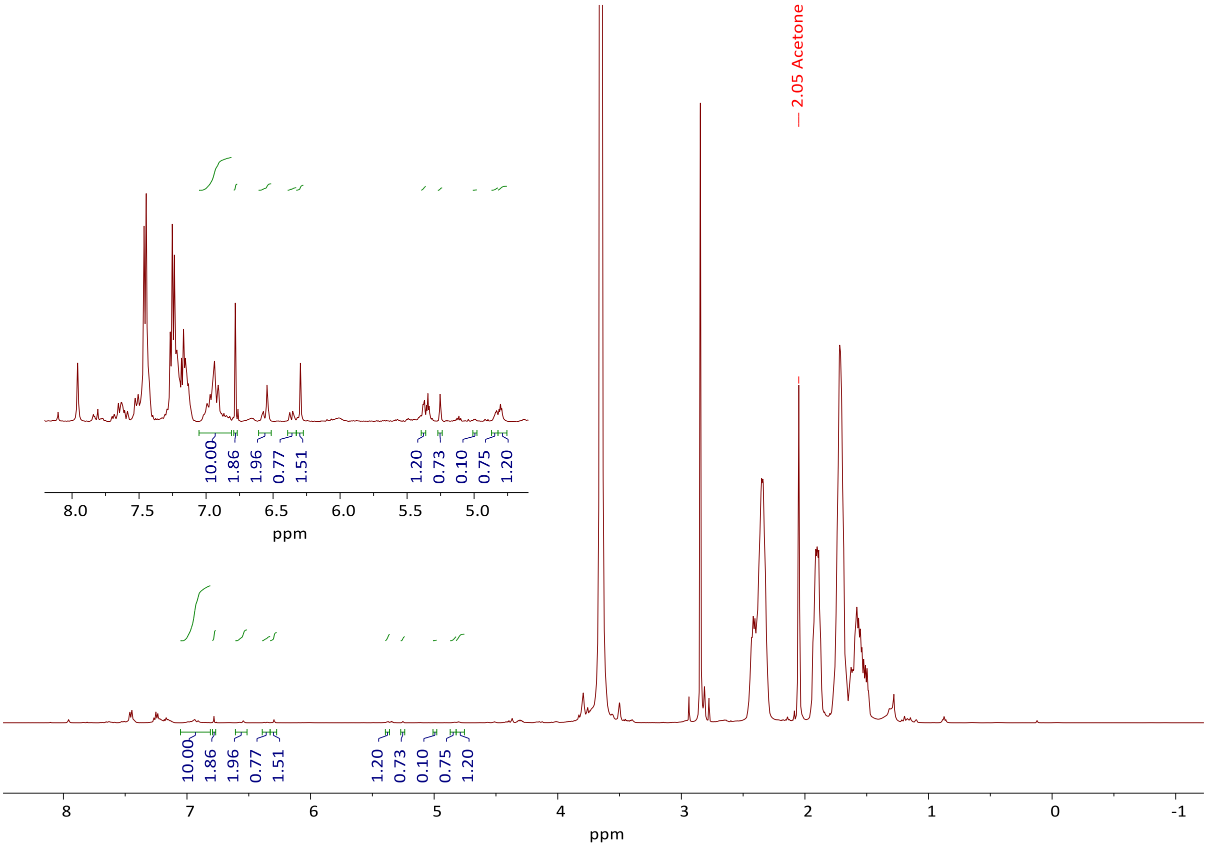

Spectrum S205. ^{1}H NMR (400 MHz, Acetone-d_6, 298 K) spectrum of post-sonication polymer **1$_3$d$_{-89}$** before being washed with methanol.

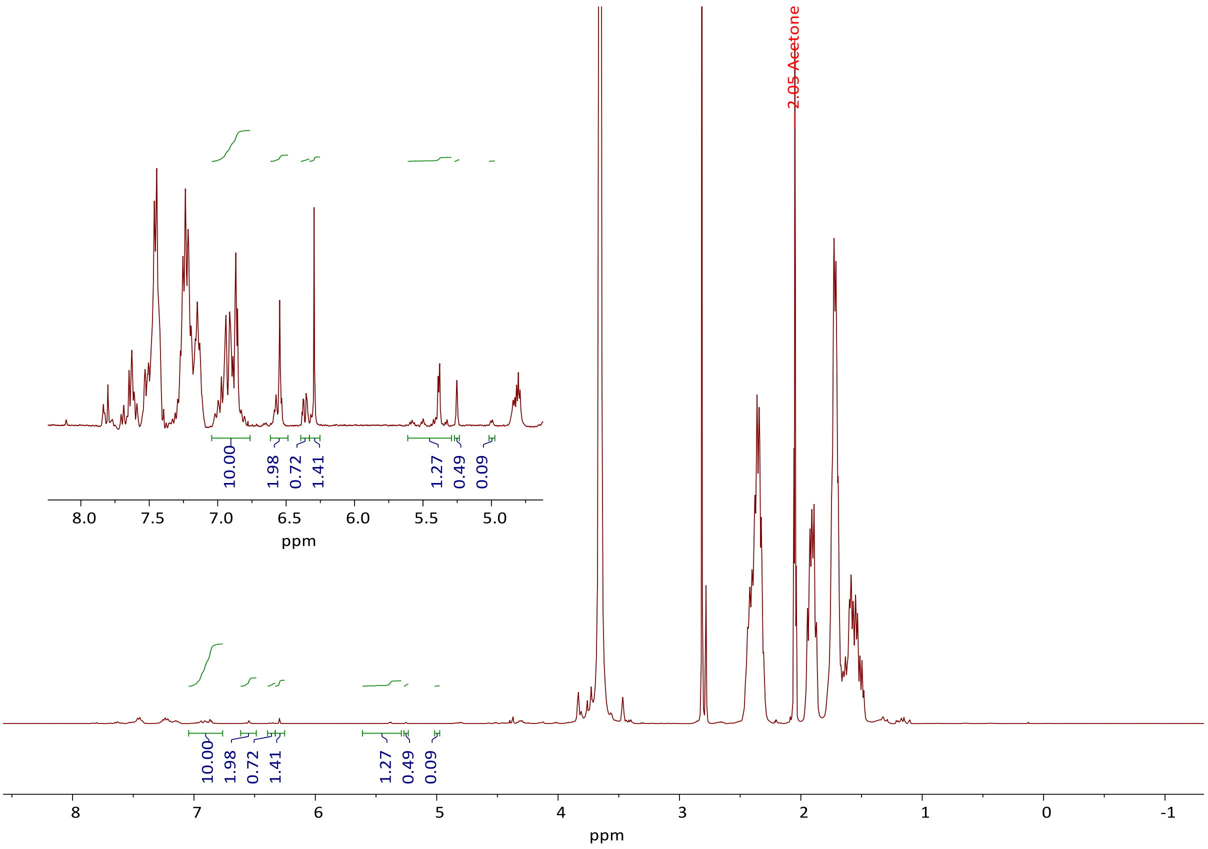

Spectrum S206. ^{1}H NMR (400 MHz, Acetone-d_6, 298 K) spectrum of post-sonication polymer $\mathbf{1_3d_{\text{-}89}}$ after being washed with methanol.

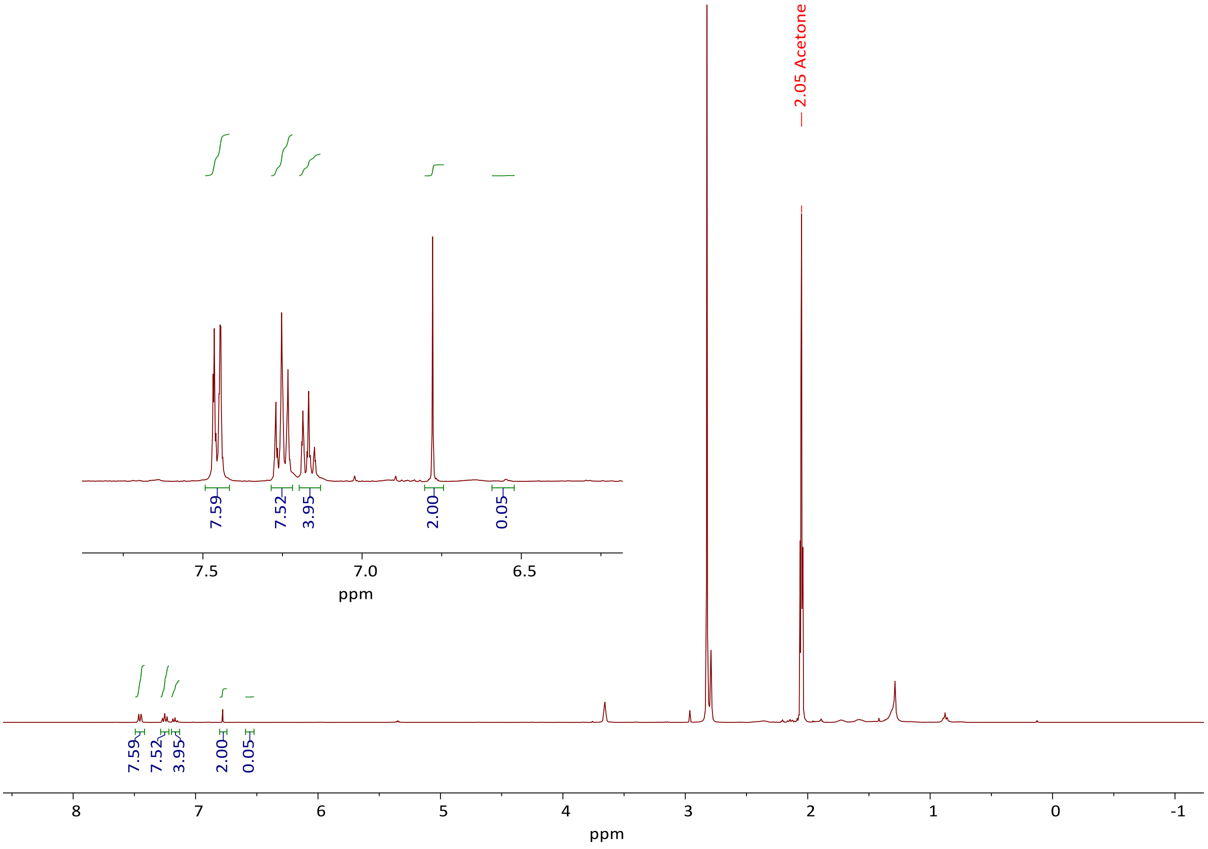

Spectrum S207. ^{1}H NMR (400 MHz, Acetone-d_6, 298 K) spectrum of the concentrated methanol washings from post-sonication polymer $\mathbf{1_3d_{\text{-}89}}$.

9.3.8 Post-Sonication ^{1}H NMR Spectra of Polymer 1_3a$_{-210}$

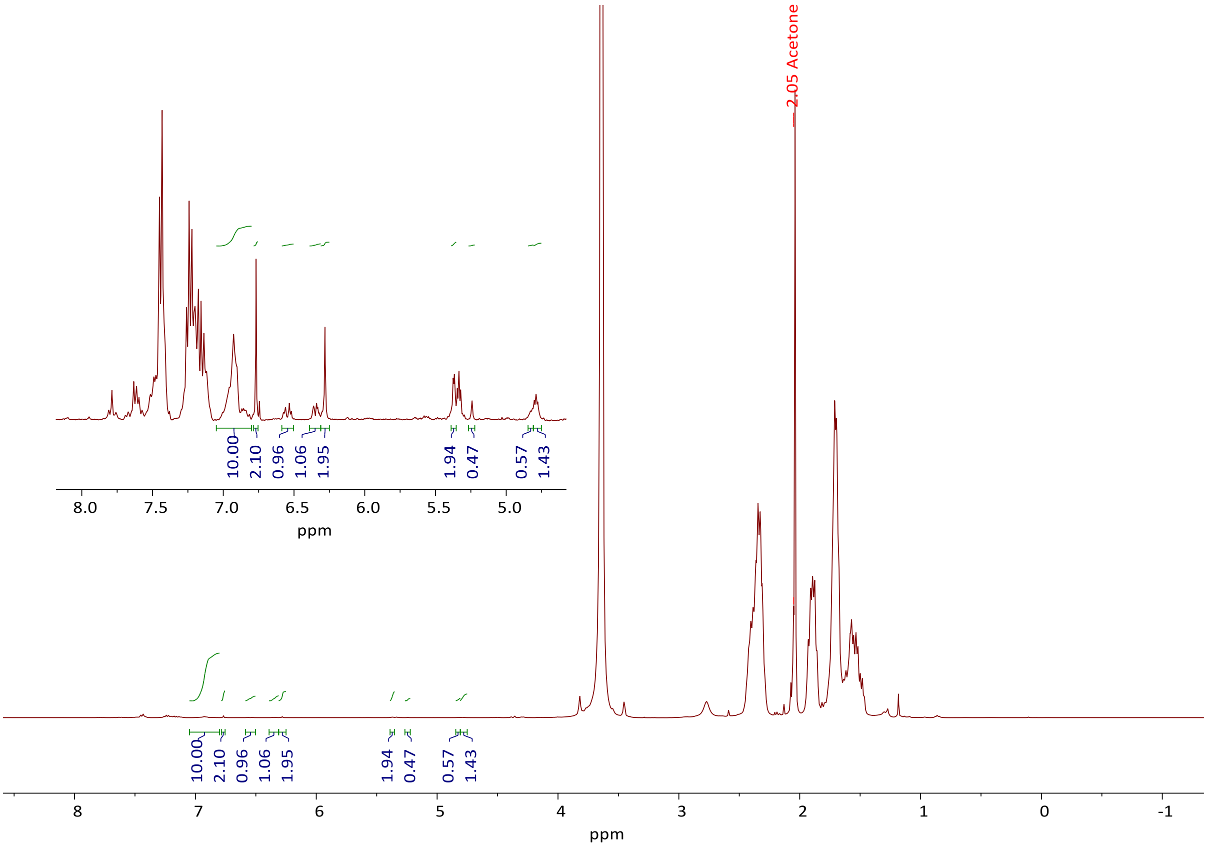

Spectrum S208. ^{1}H NMR (400 MHz, Acetone-d_6, 298 K) spectrum of post-sonication polymer **1_3a$_{-210}$** before being washed with methanol.

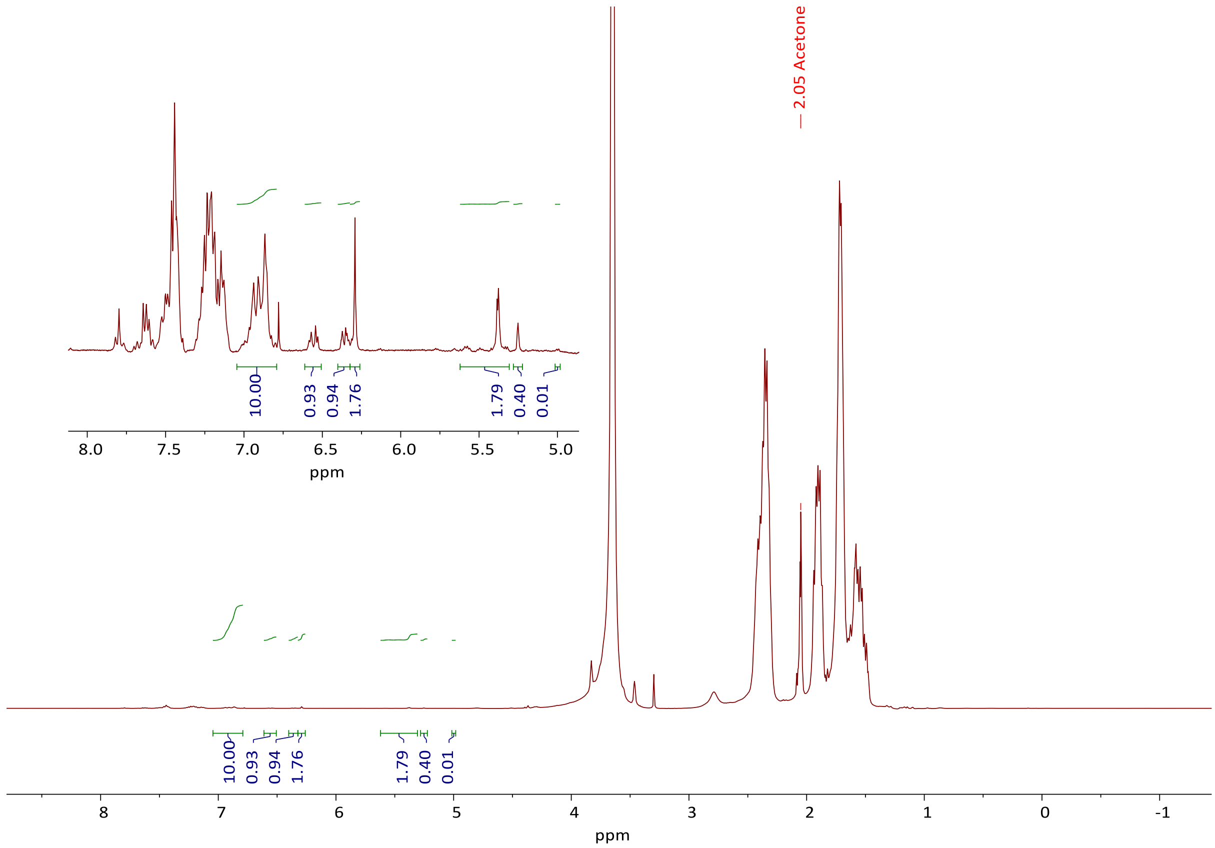

Spectrum S209. ^{1}H NMR (400 MHz, Acetone-d_6, 298 K) spectrum of post-sonication polymer **1_3a$_{-210}$** after being washed with methanol.

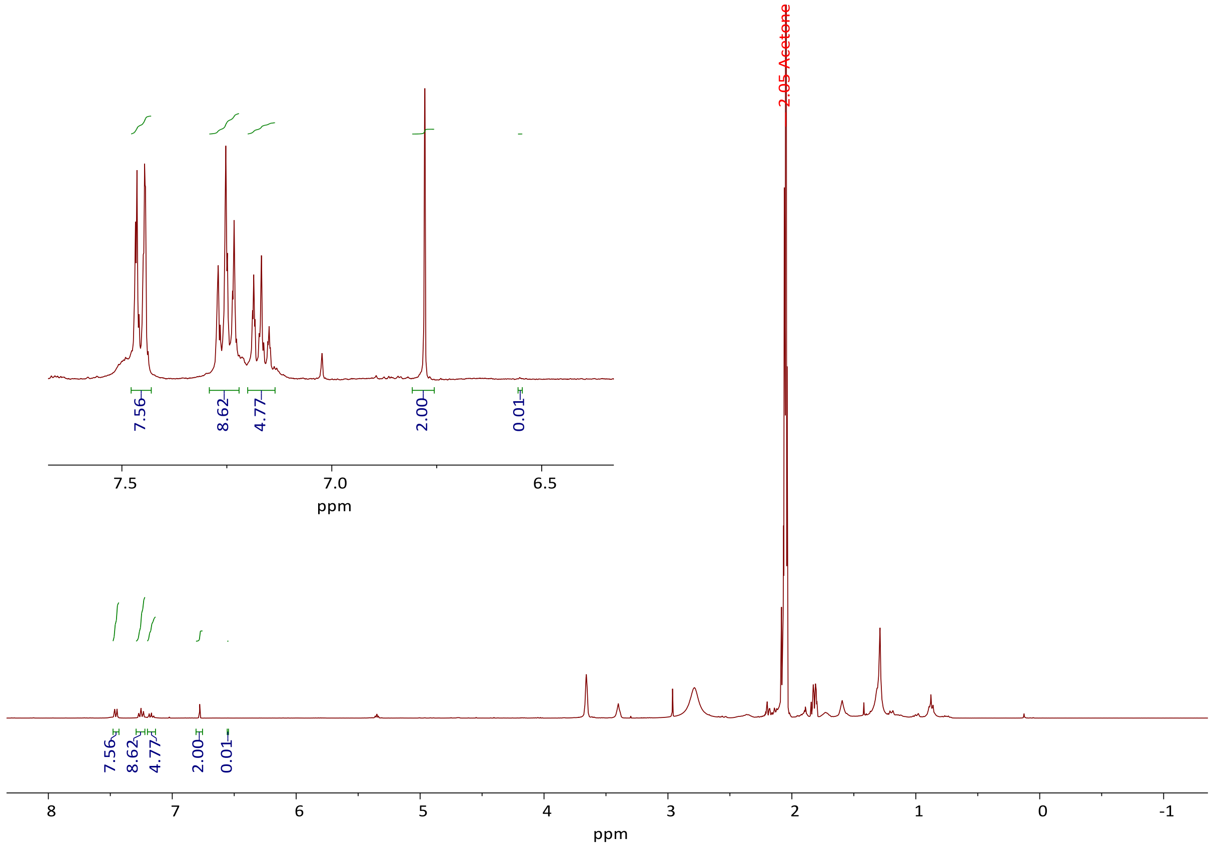

Spectrum S210. ^{1}H NMR (400 MHz, Acetone-d_6, 298 K) spectrum of the concentrated methanol washings from post-sonication polymer **1_3a$_{-210}$**.

9.3.9 Post-Sonication ^{1}H NMR Spectra of Polymer 1_3b$_{-142}$

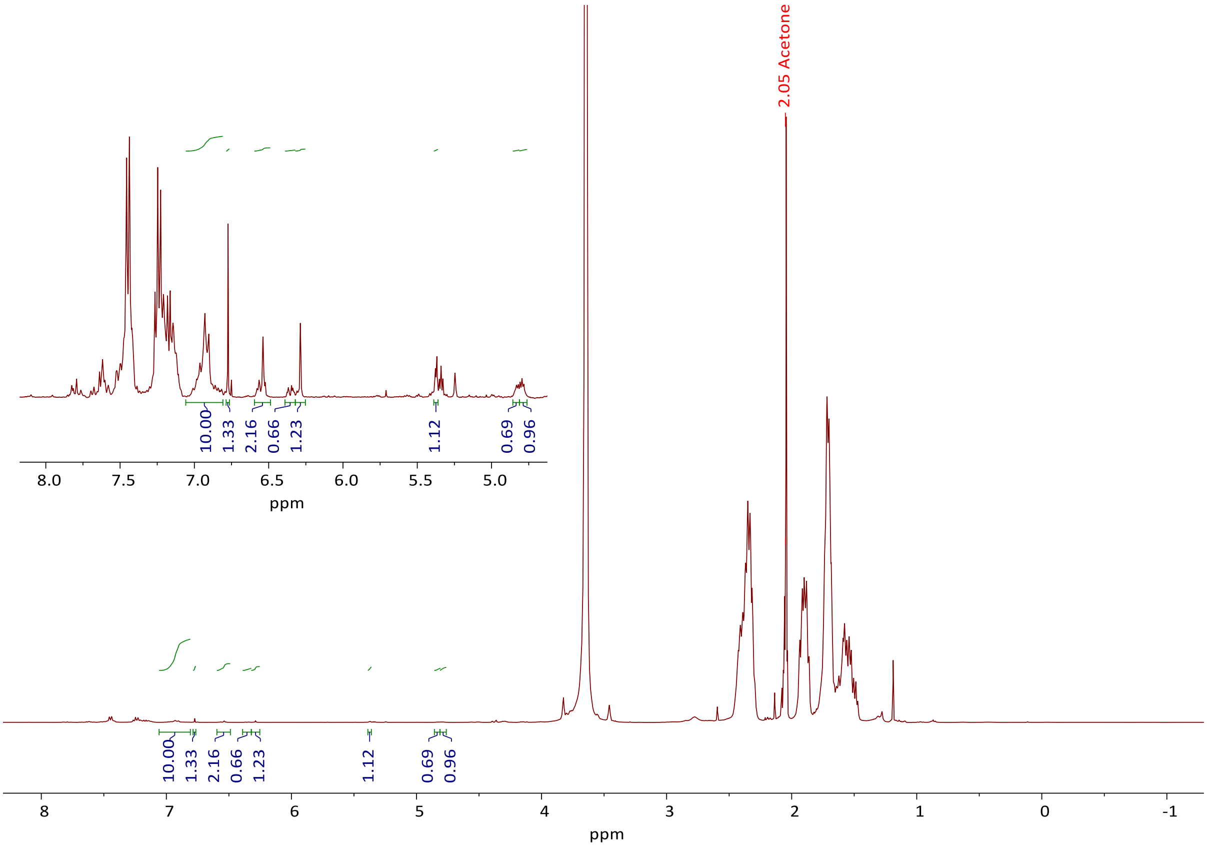

Spectrum S211. ^{1}H NMR (400 MHz, Acetone-d_6, 298 K) spectrum of post-sonication polymer **1_3b$_{-142}$** before being washed with methanol.

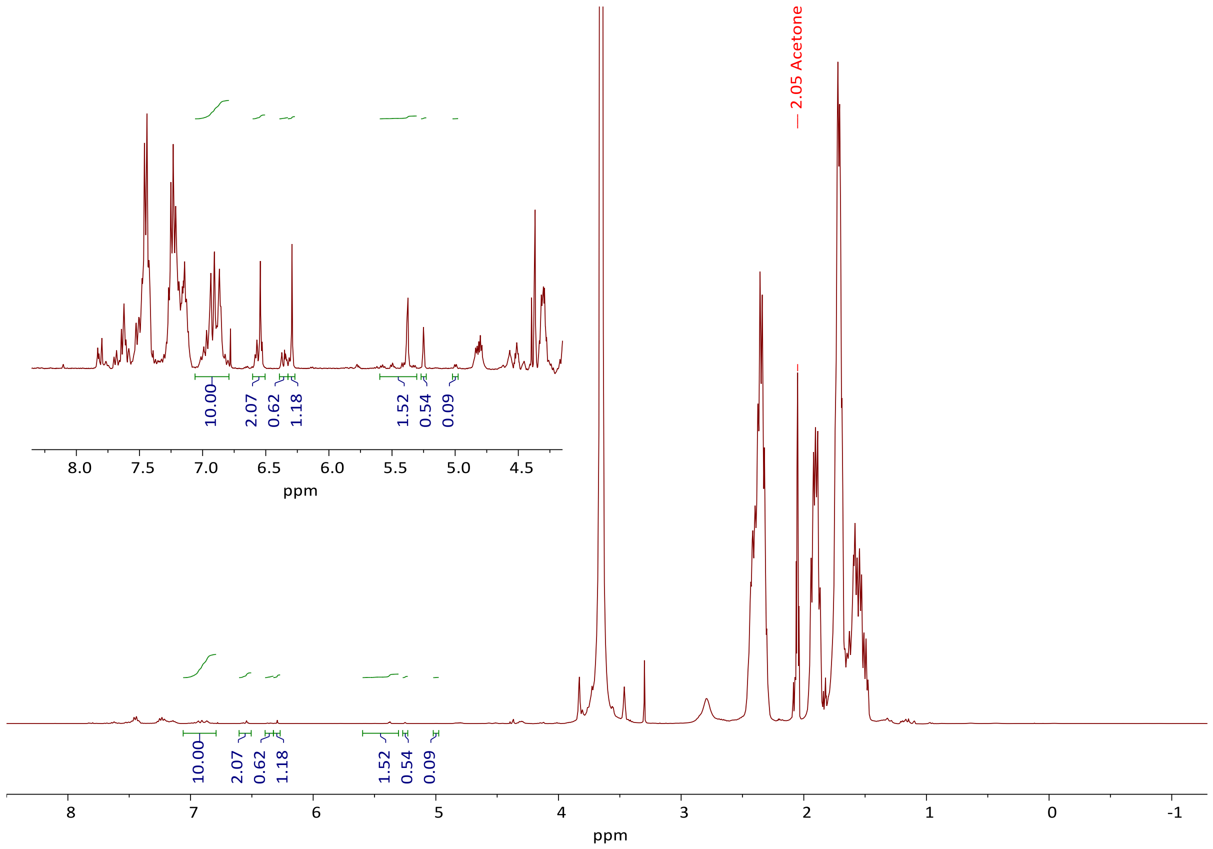

Spectrum S212. ^{1}H NMR (400 MHz, Acetone-d_6, 298 K) spectrum of post-sonication polymer **1₃b₋₁₄₂** after being washed with methanol.

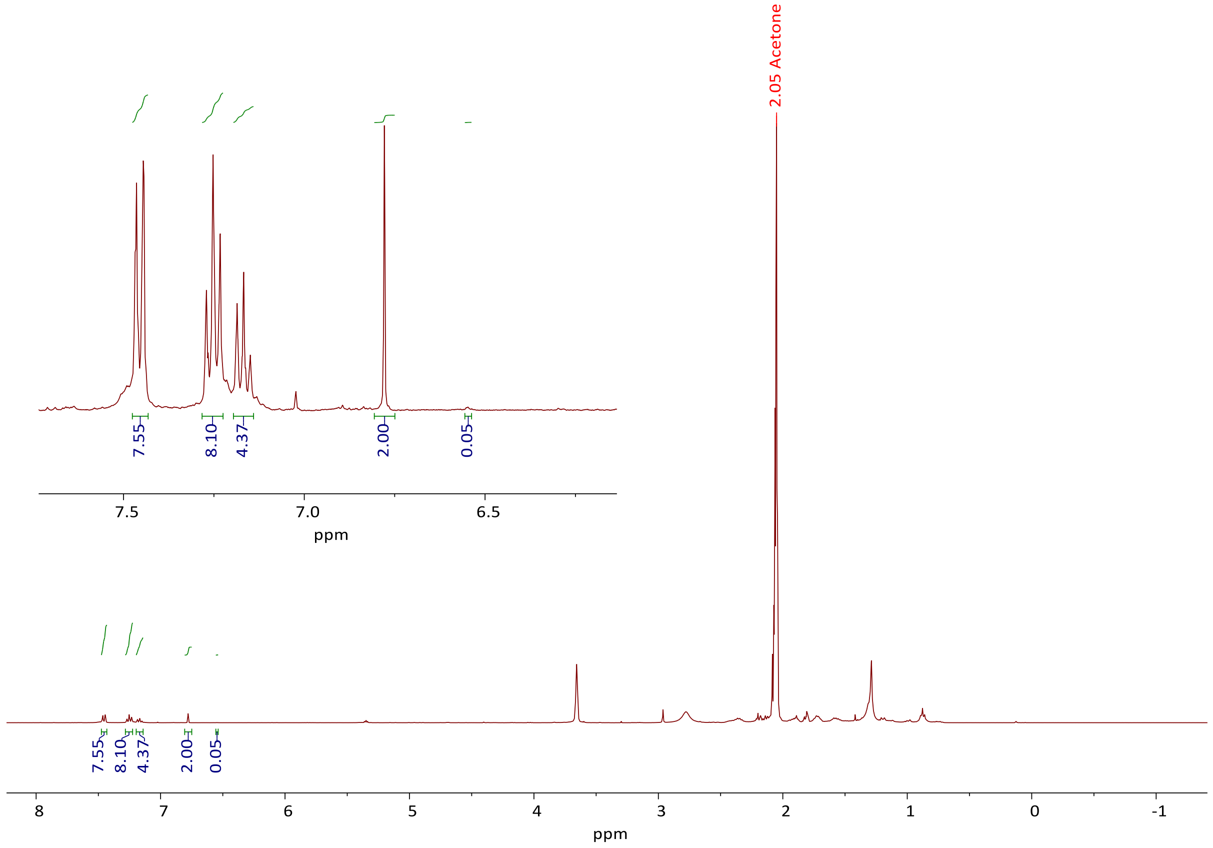

Spectrum S213. ^{1}H NMR (400 MHz, Acetone-d_6, 298 K) spectrum of the concentrated methanol washings from post-sonication polymer **1_3b$_{-142}$**.

9.3.10 Post-Sonication ^{1}H NMR Spectra of Polymer 1_3b_{-171}

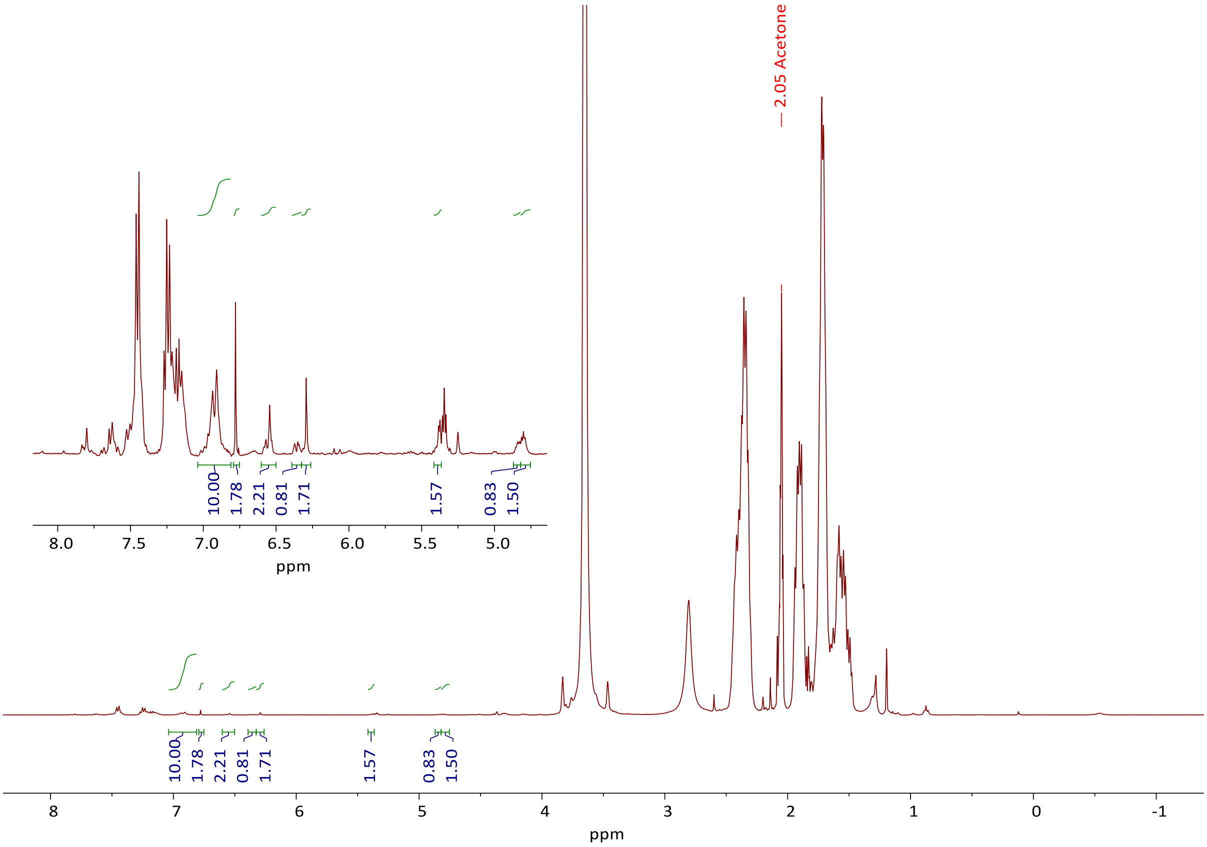

Spectrum S214. ^{1}H NMR (400 MHz, Acetone-d_6, 298 K) spectrum of post-sonication polymer $\mathbf{1_3b_{-171}}$ before being washed with methanol.

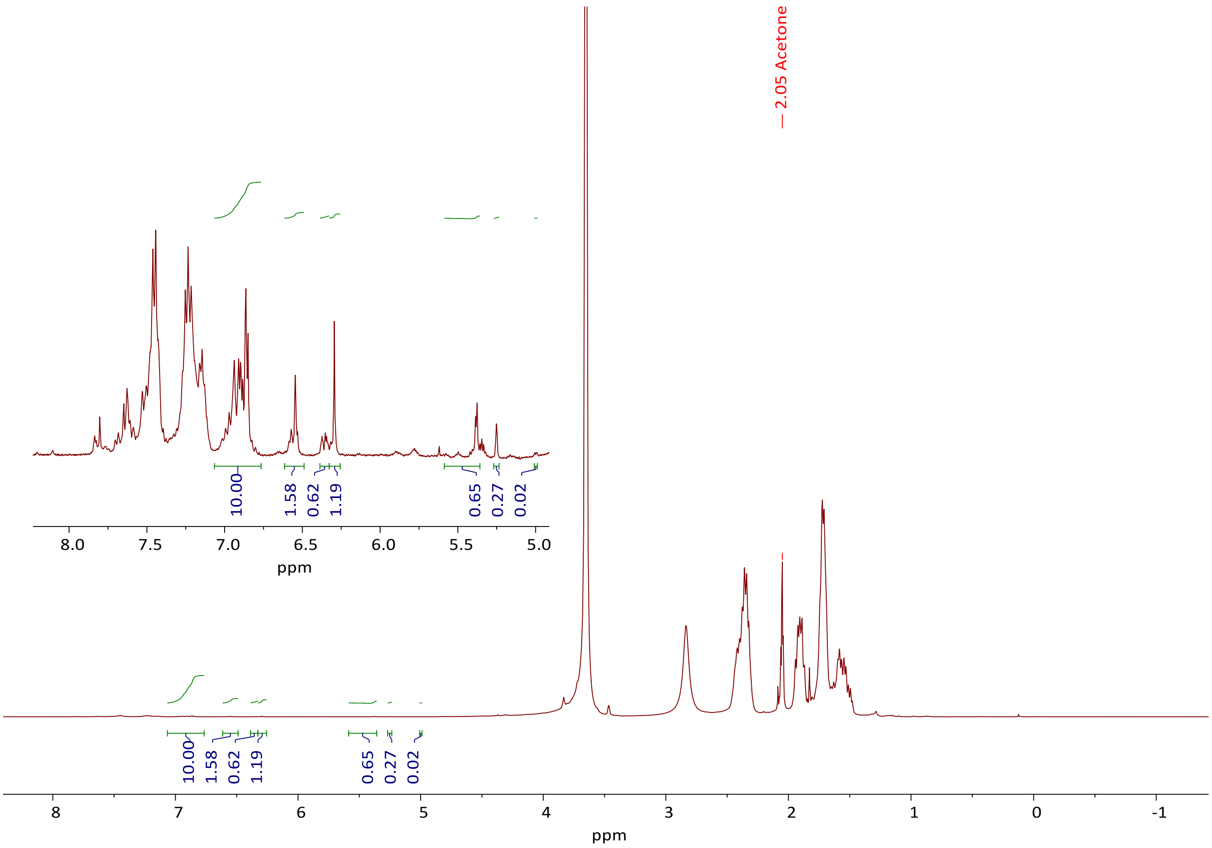

Spectrum S215. ^{1}H NMR (400 MHz, Acetone-d_6, 298 K) spectrum of post-sonication polymer **1₃b₋₁₇₁** after being washed with methanol.

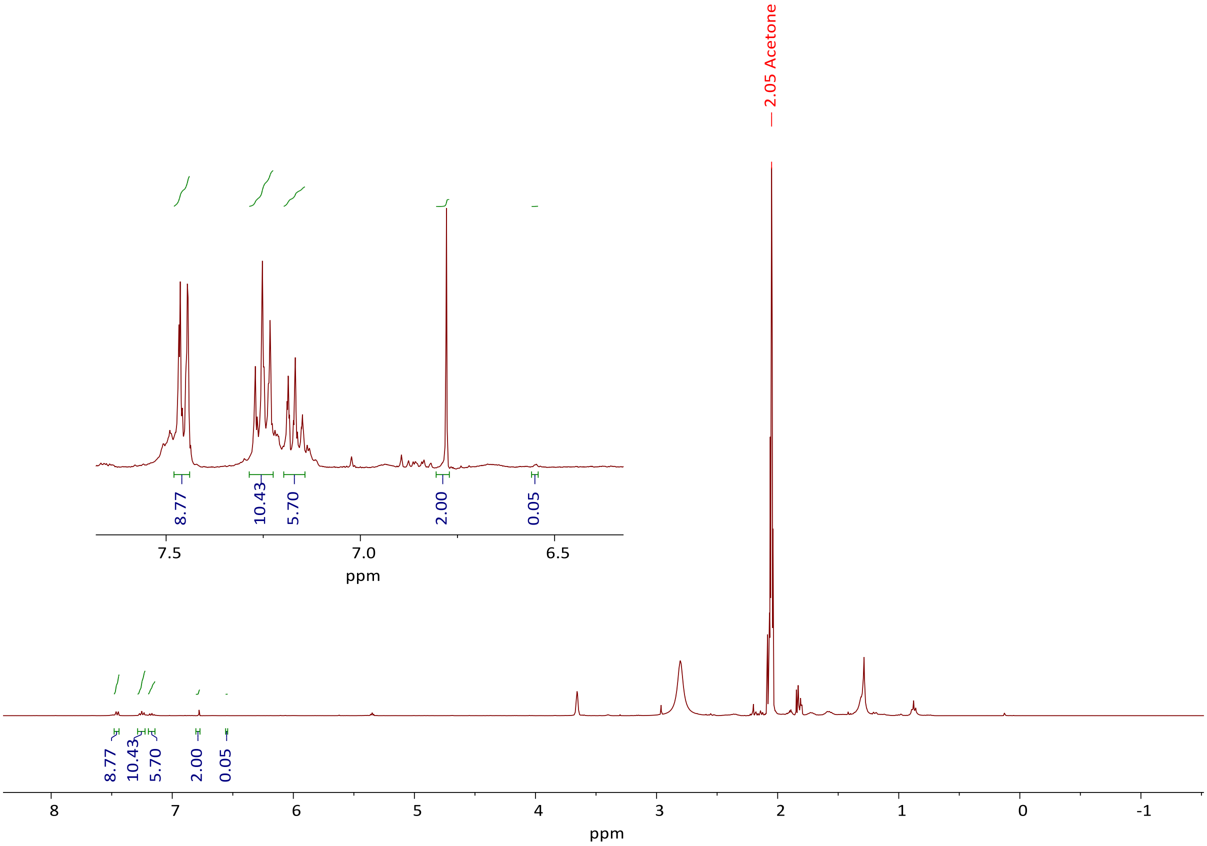

Spectrum S216. ^{1}H NMR (400 MHz, Acetone-d_6, 298 K) spectrum of the concentrated methanol washings from post-sonication polymer **1₃b₋₁₇₁**.

9.3.11 Post-Sonication ^{1}H NMR Spectra of Polymer 1_3b-178

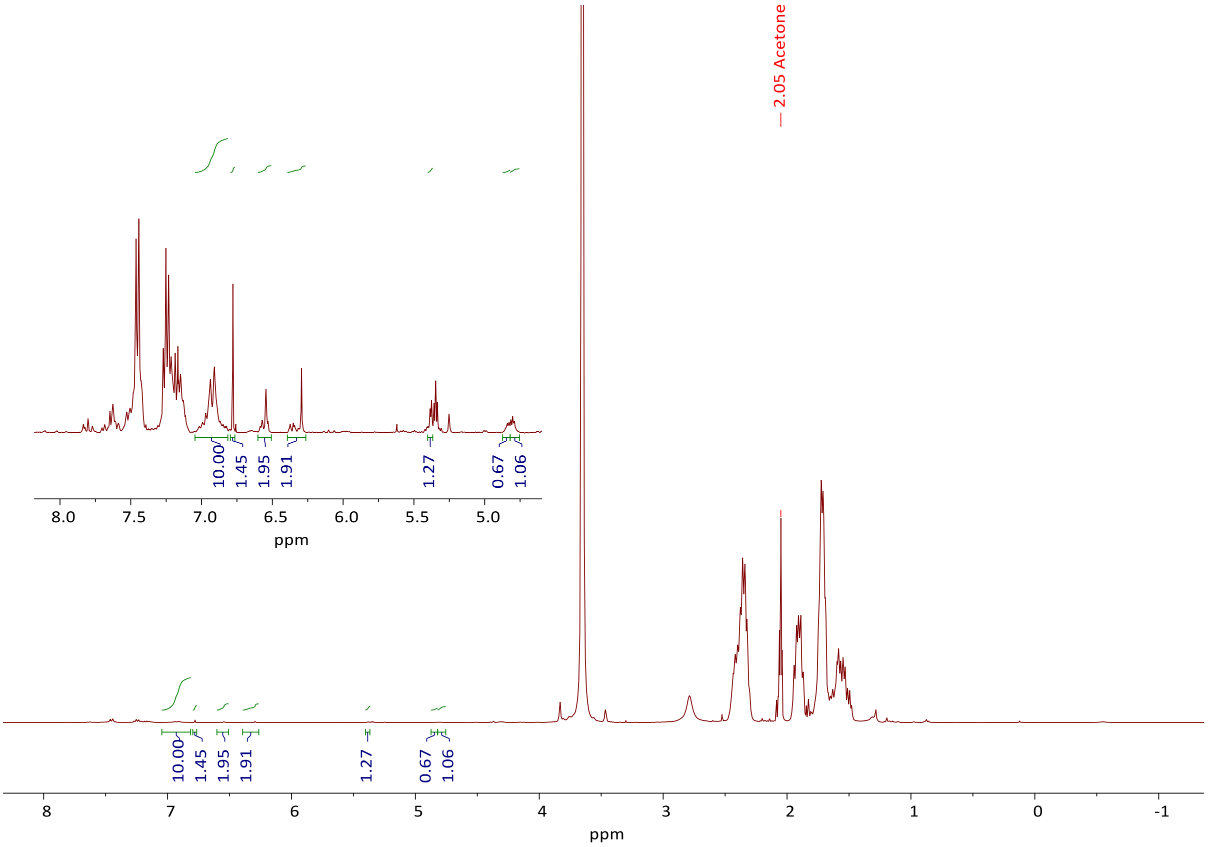

Spectrum S217. ^{1}H NMR (400 MHz, Acetone-d_6, 298 K) spectrum of post-sonication polymer **1_3b-178** before being washed with methanol.

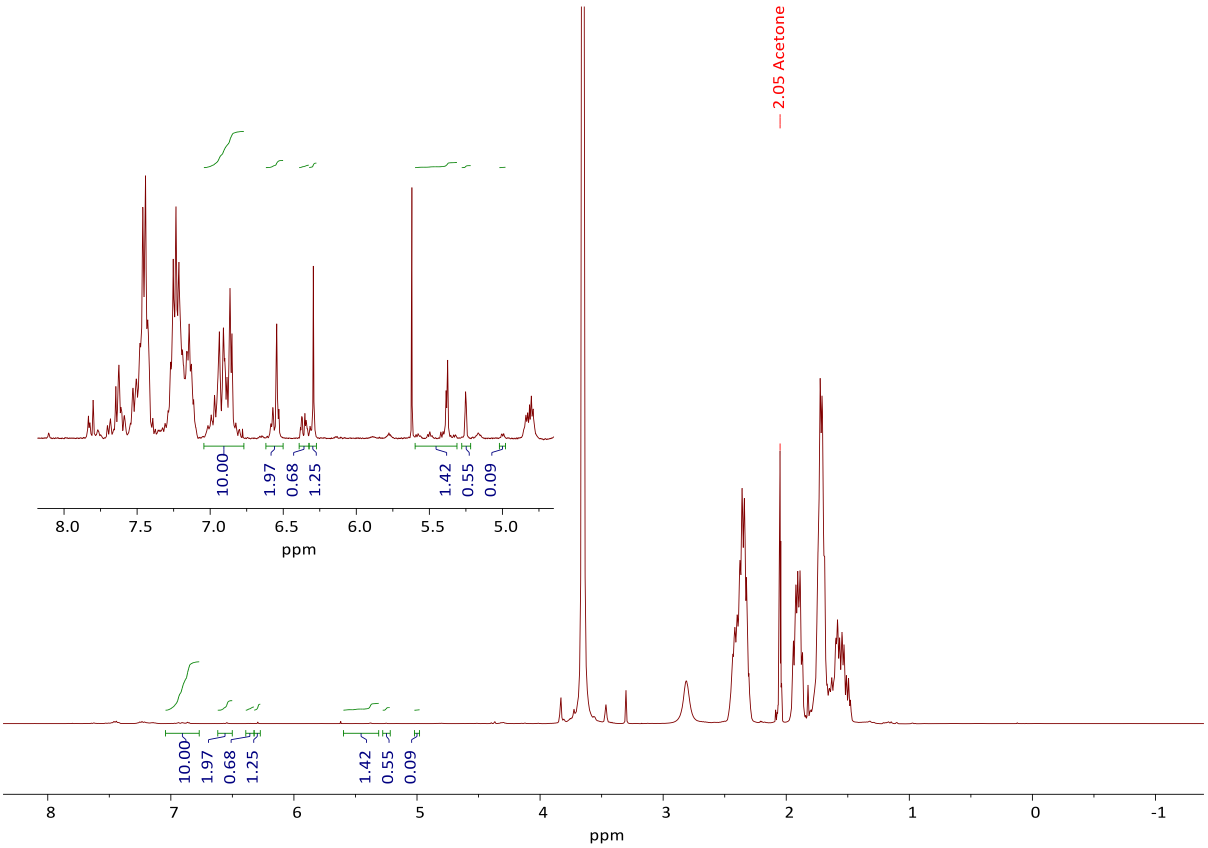

Spectrum S218. ^{1}H NMR (400 MHz, Acetone-d_6, 298 K) spectrum of post-sonication polymer $\mathbf{1_3b_{-178}}$ after being washed with methanol.

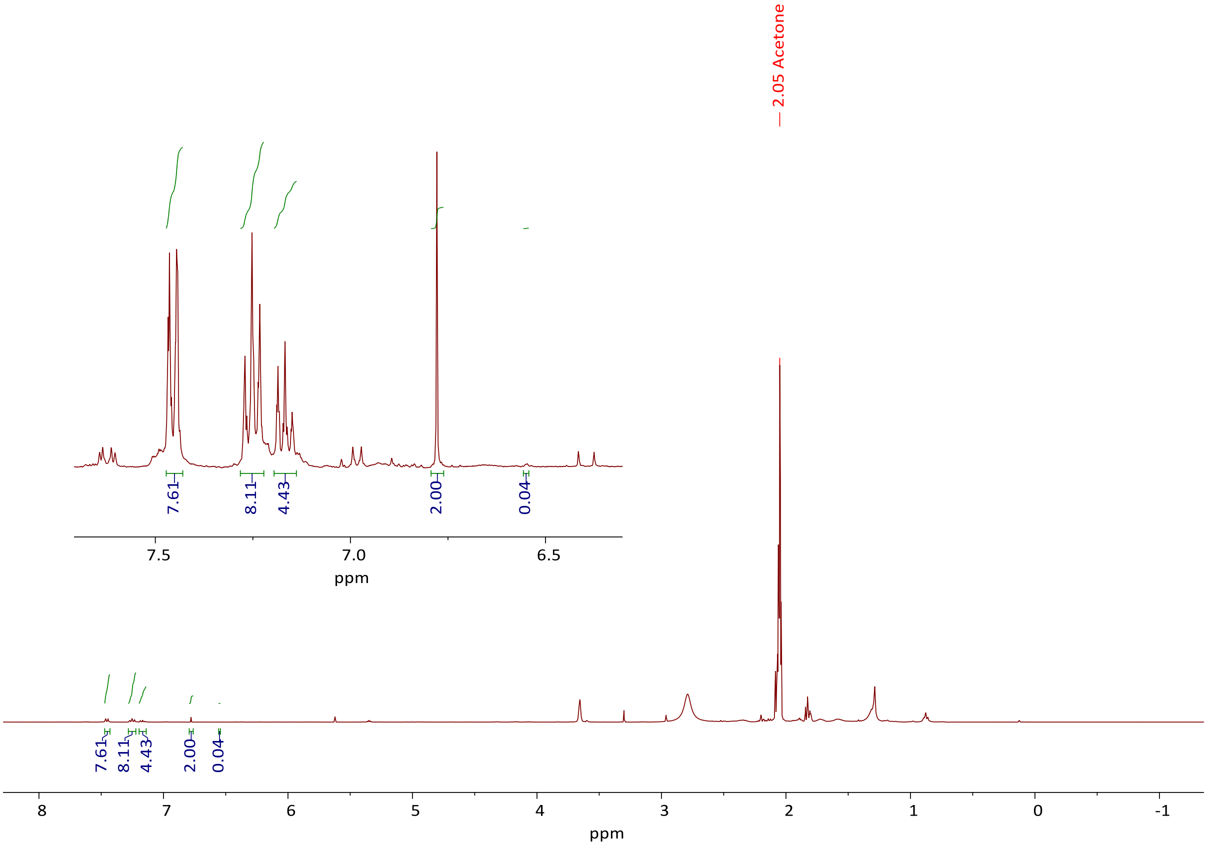

Spectrum S219. ^{1}H NMR (400 MHz, Acetone-d_6, 298 K) spectrum of the concentrated methanol washings from post-sonication polymer $\mathbf{1_3b_{-178}}$.

9.3.12 Post-Sonication ^{1}H NMR Spectra of Polymer **1$_{3C-174}$**

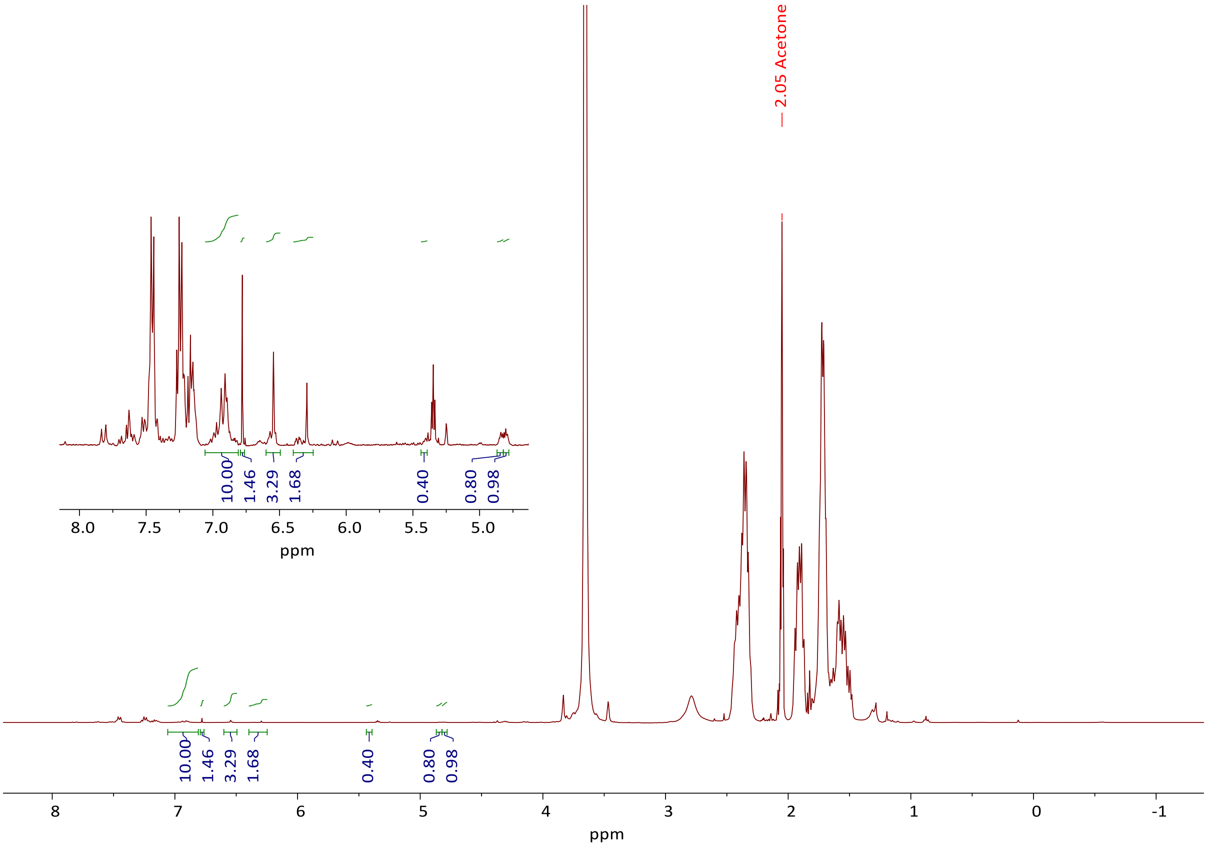

Spectrum S220. ^{1}H NMR (400 MHz, Acetone-d_6, 298 K) spectrum of post-sonication polymer **1$_{3C-174}$** before being washed with methanol.

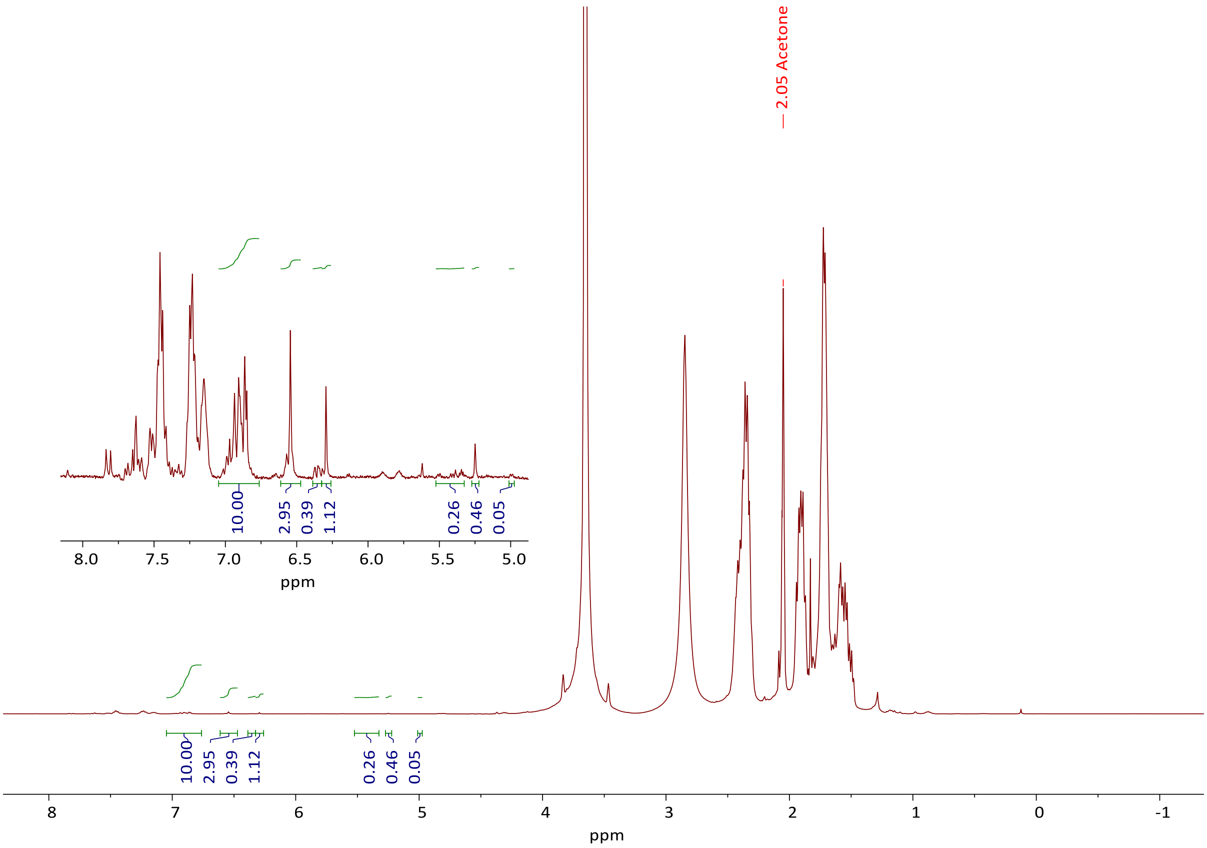

Spectrum S221. ^{1}H NMR (400 MHz, Acetone-d_6, 298 K) spectrum of post-sonication polymer **1**$_3$**c**$_{-174}$ after being washed with methanol.

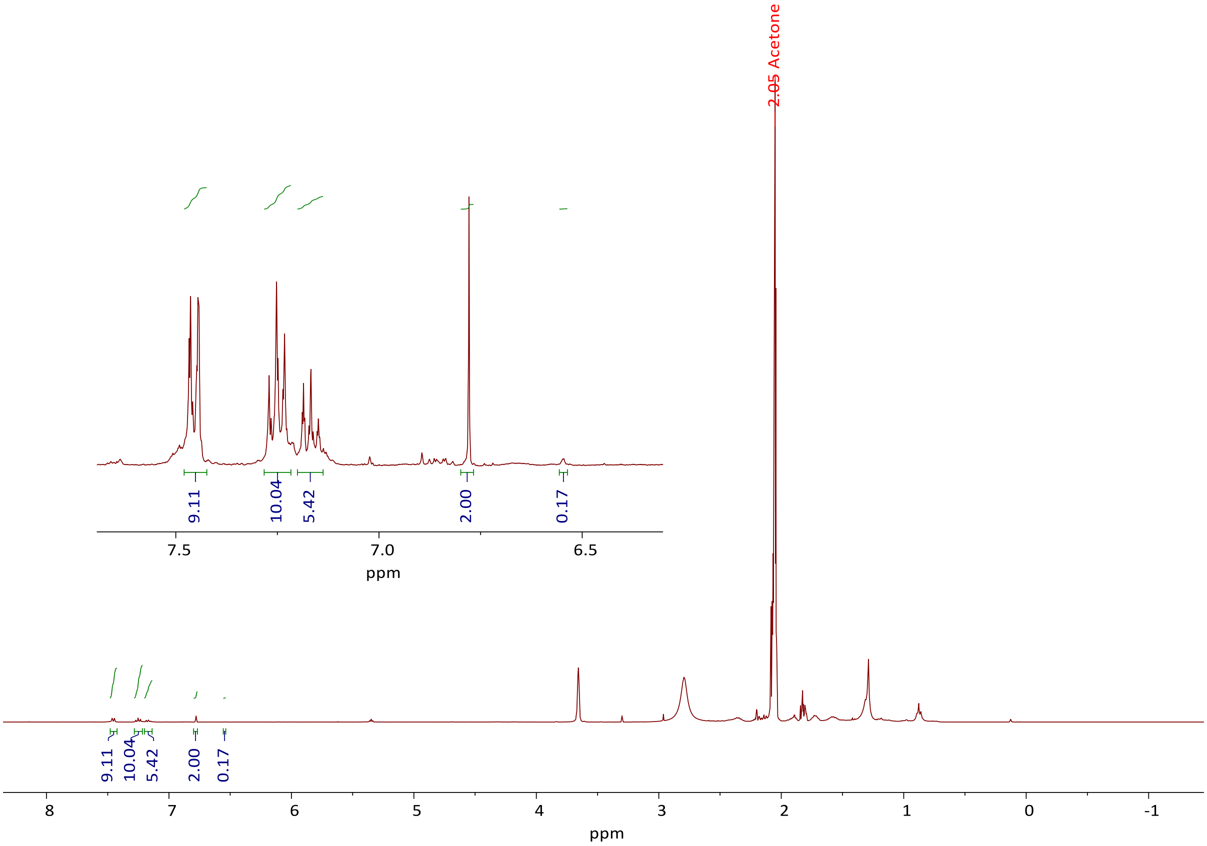

Spectrum S222. ^{1}H NMR (400 MHz, Acetone-d_6, 298 K) spectrum of the concentrated methanol washings from post-sonication polymer **1**$_3$**c**$_{-174}$.

9.3.13 Post-Sonication ^{1}H NMR Spectra of Polymer $1_{5\text{-}60}$

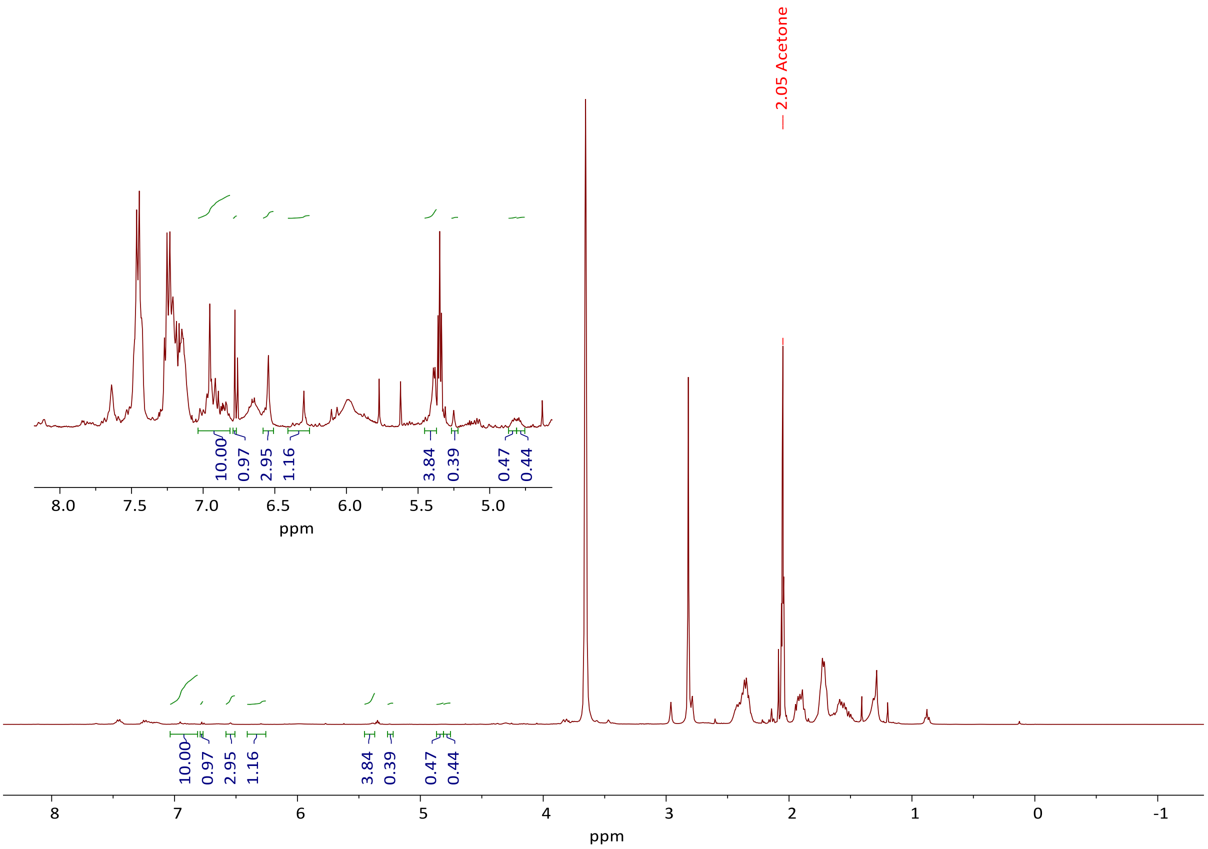

Spectrum S223. ^{1}H NMR (400 MHz, Acetone-d_6, 298 K) spectrum of post-sonication polymer $\mathbf{1}_{5\text{-}60}$ before being washed with methanol.

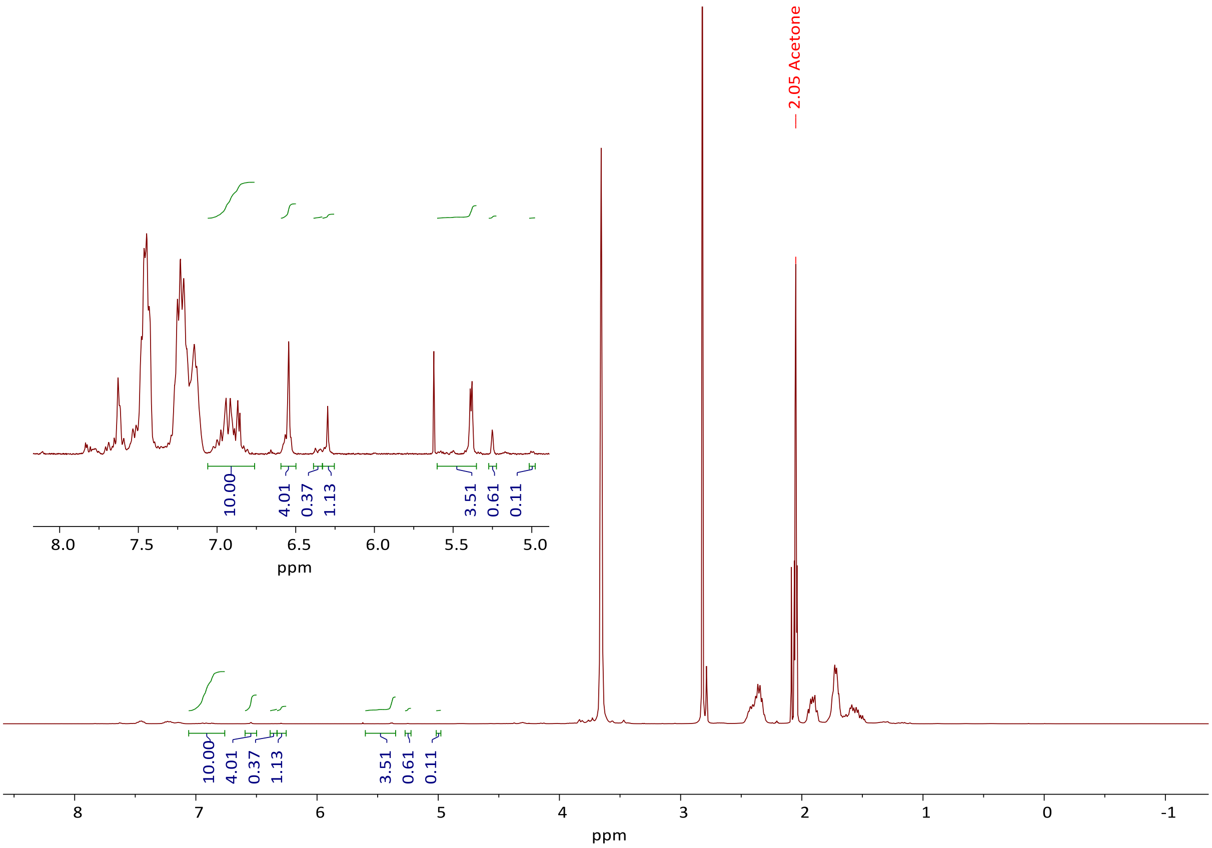

Spectrum S224. ^{1}H NMR (400 MHz, Acetone-d_6, 298 K) spectrum of post-sonication polymer **1**$_{\mathbf{5\text{-}60}}$ after being washed with methanol.

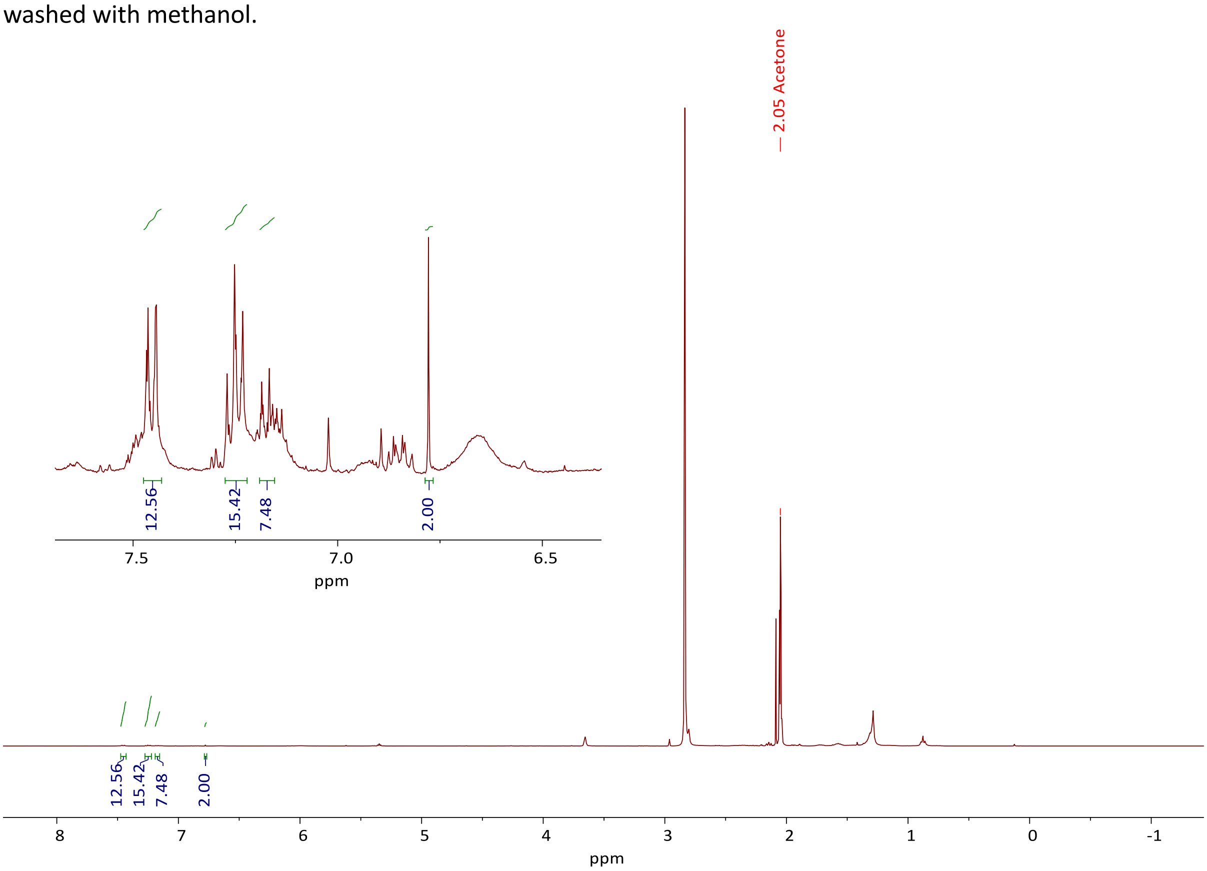

Spectrum S225. ^{1}H NMR (400 MHz, Acetone-d_6, 298 K) spectrum of the concentrated methanol washings from post-sonication polymer **1**$_{\mathbf{5\text{-}60}}$.

9.3.14 Post-Sonication ^{1}H NMR Spectra of Polymer $\mathbf{1_{5\text{-}165}}$

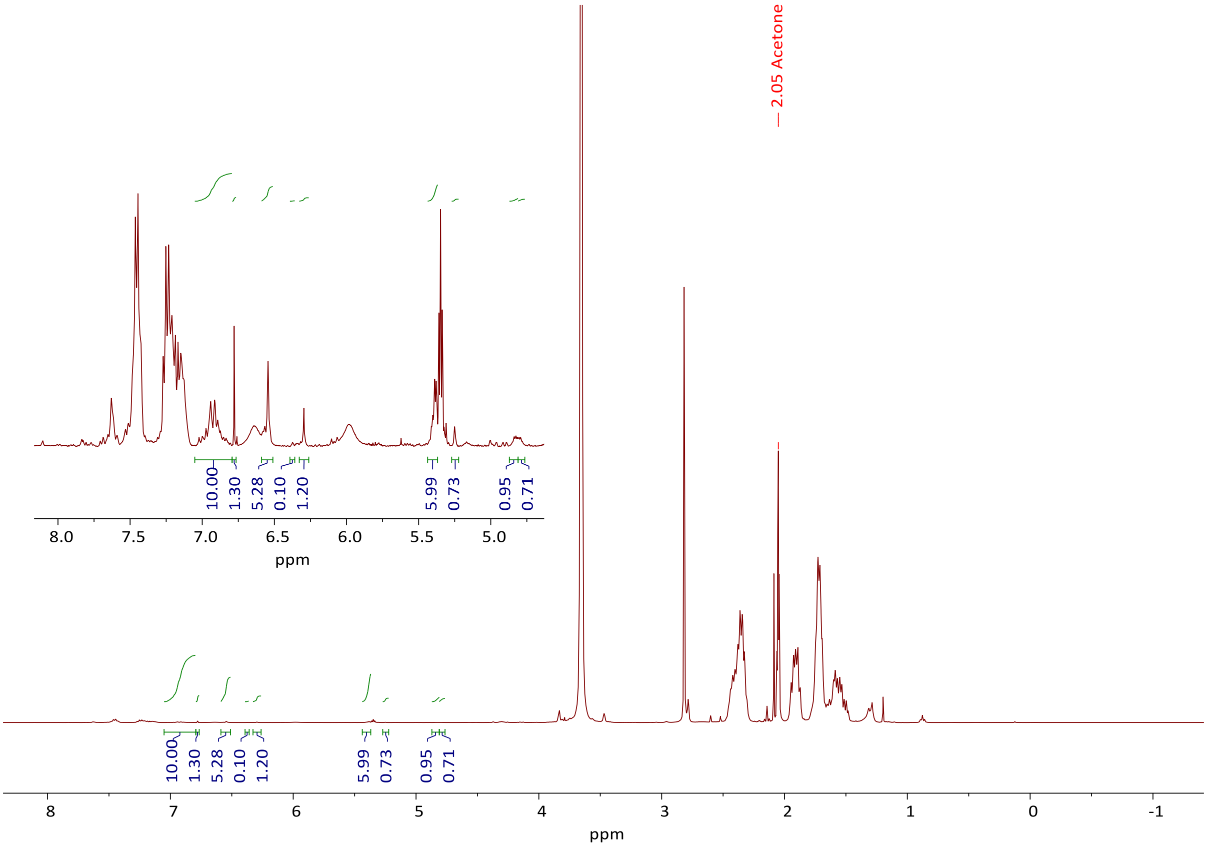

Spectrum S226. ^{1}H NMR (400 MHz, Acetone-d_6, 298 K) spectrum of post-sonication polymer $\mathbf{1_{5\text{-}165}}$ before being washed with methanol.

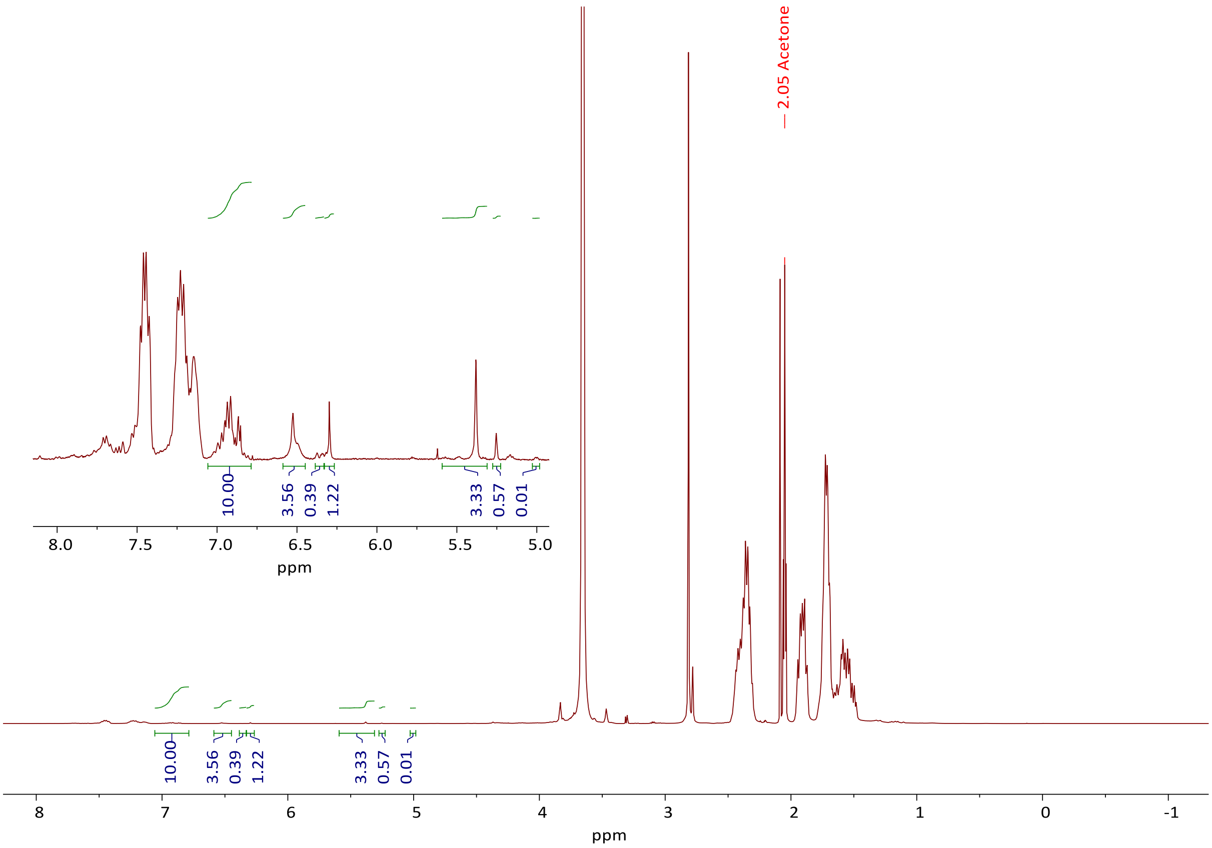

Spectrum S227. ^{1}H NMR (400 MHz, Acetone-d_6, 298 K) spectrum of post-sonication polymer $\mathbf{1}_{\mathbf{5\text{-}165}}$ after being washed with methanol.

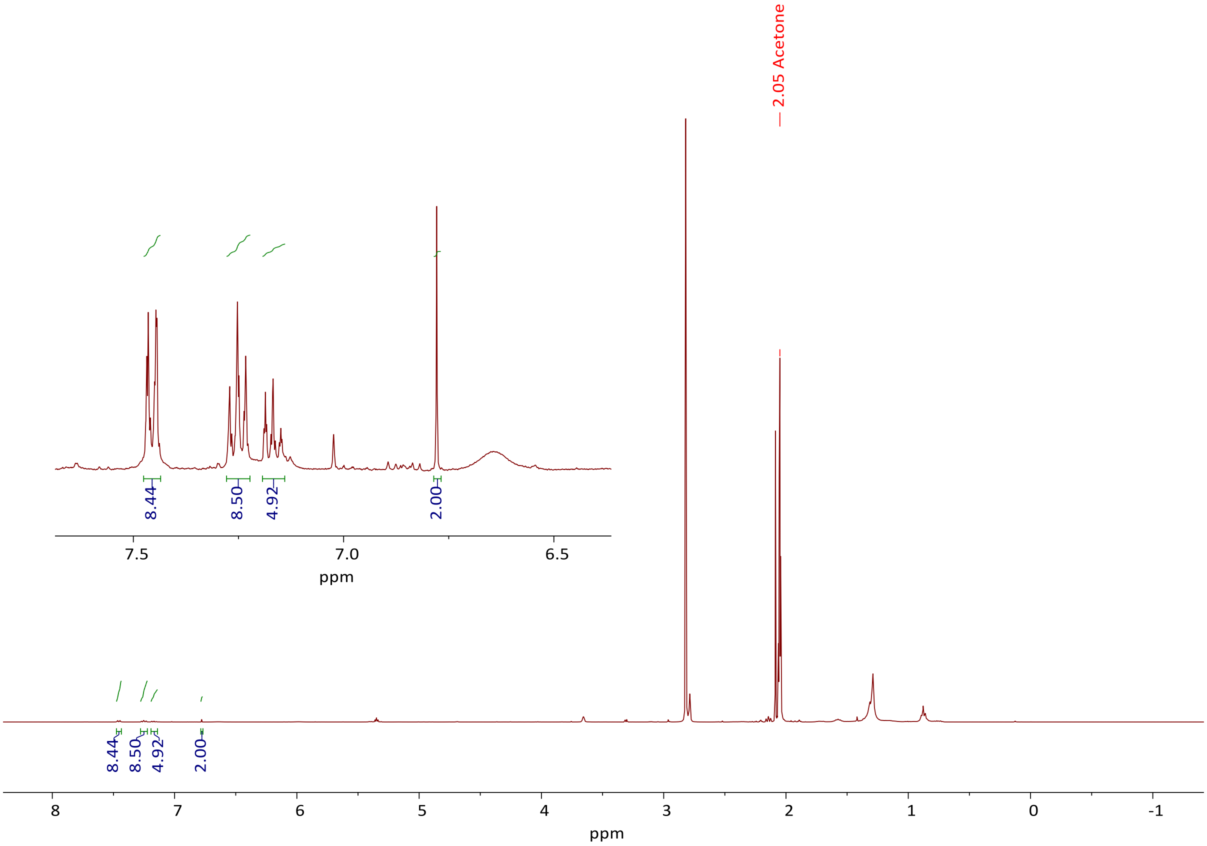

Spectrum S228. ^{1}H NMR (400 MHz, Acetone-d_6, 298 K) spectrum of the concentrated methanol washings from post-sonication polymer $\mathbf{1}_{\mathbf{5\text{-}165}}$.

9.3.15 Post-Sonication ^{1}H NMR Spectra of Polymer $1_{5\text{-}215}$

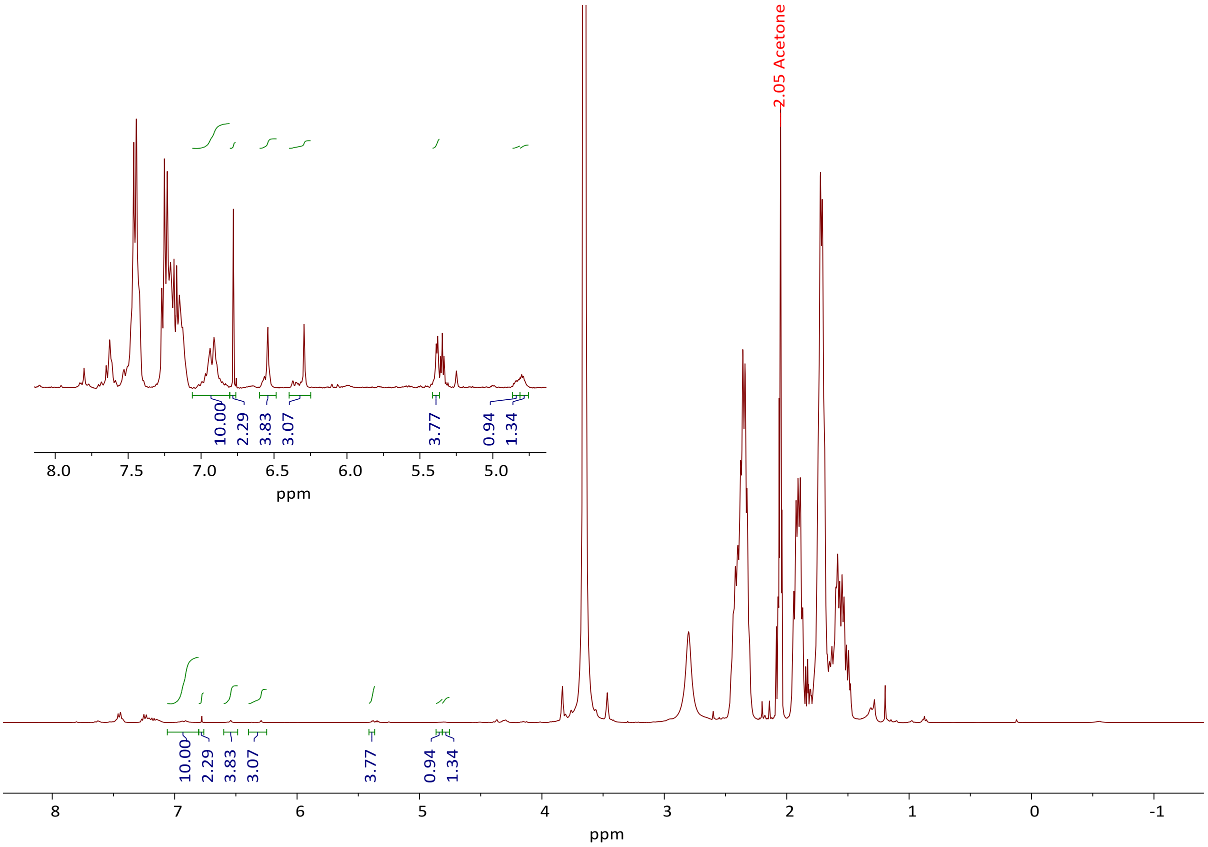

Spectrum S229. ^{1}H NMR (400 MHz, Acetone-d_6, 298 K) spectrum of post-sonication polymer $\mathbf{1_{5\text{-}215}}$ before being washed with methanol.

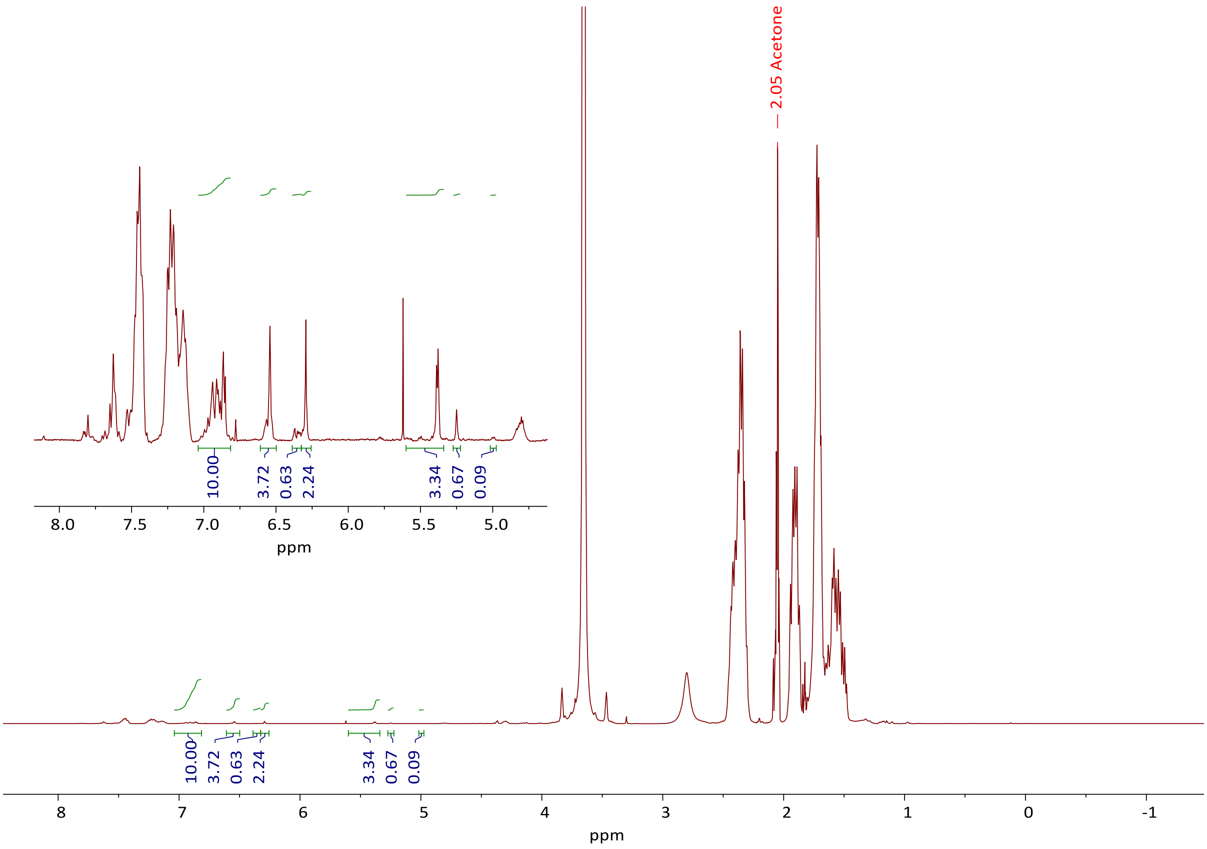

Spectrum S230. ^{1}H NMR (400 MHz, Acetone-d_6, 298 K) spectrum of post-sonication polymer $\mathbf{1}_{\mathbf{5\text{-}215}}$ after being washed with methanol.

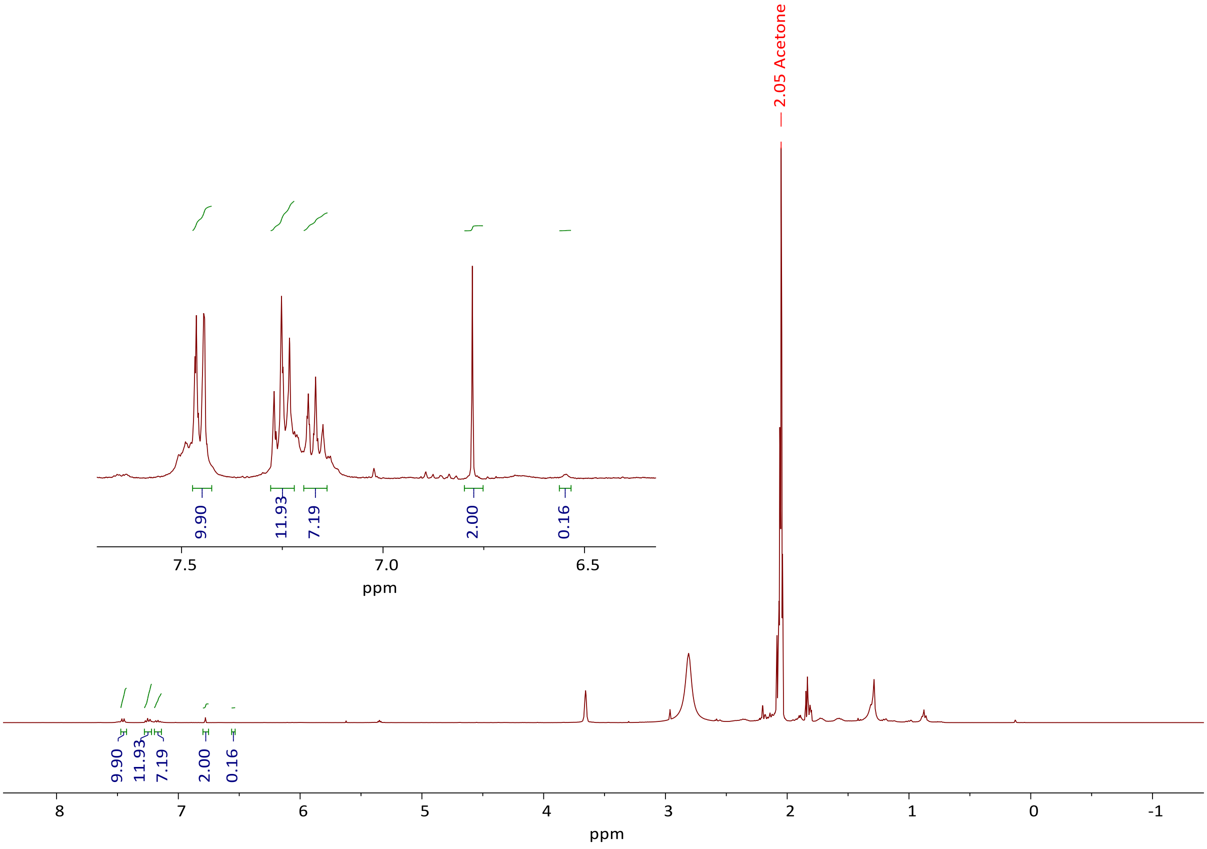

Spectrum S231. ^{1}H NMR (400 MHz, Acetone-d_6, 298 K) spectrum of the concentrated methanol washings from post-sonication polymer $\mathbf{1}_{\mathbf{5\text{-}215}}$.

9.3.16 Post-Sonication ^{1}H NMR Spectra of Polymer $\mathbf{11}_{-127}$

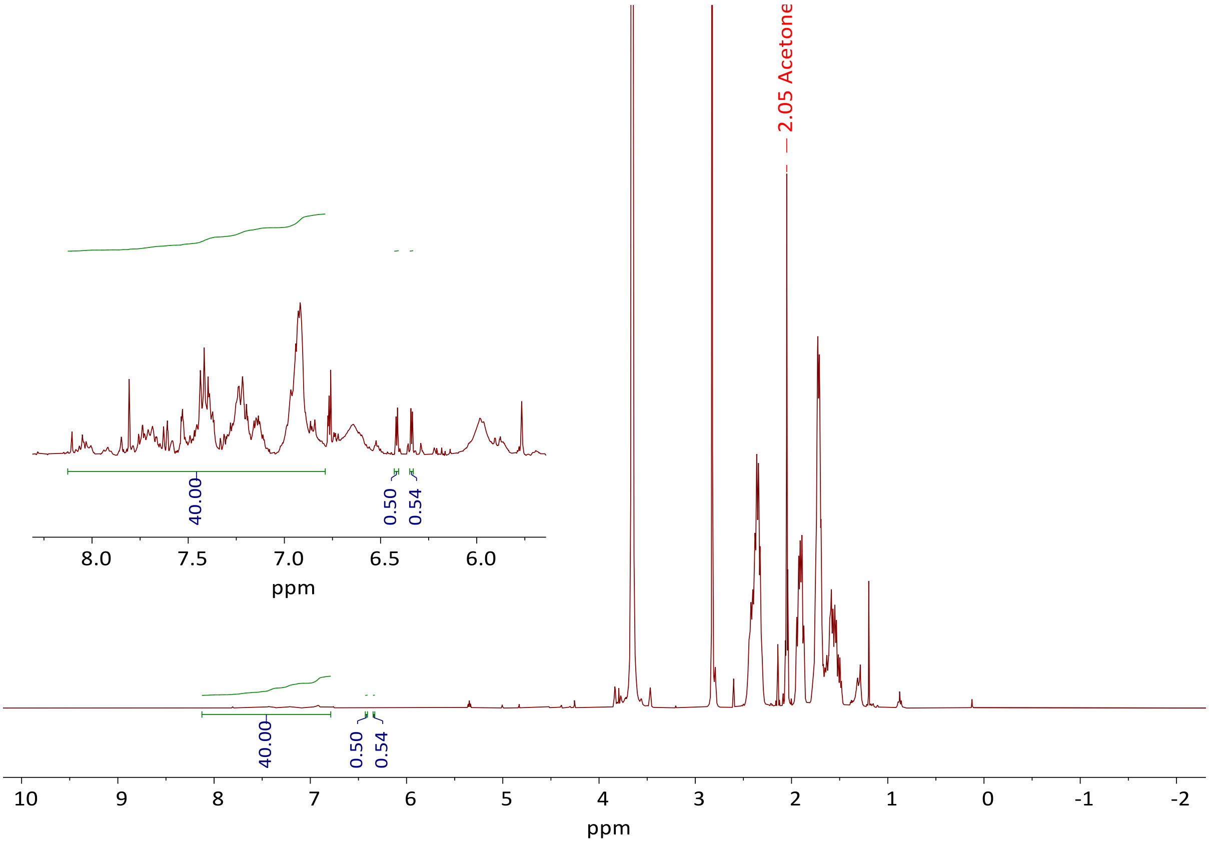

Spectrum S232. ^{1}H NMR (400 MHz, Acetone-d_6, 298 K) spectrum of post-sonication polymer $\mathbf{11}_{-127}$ before being washed with methanol.

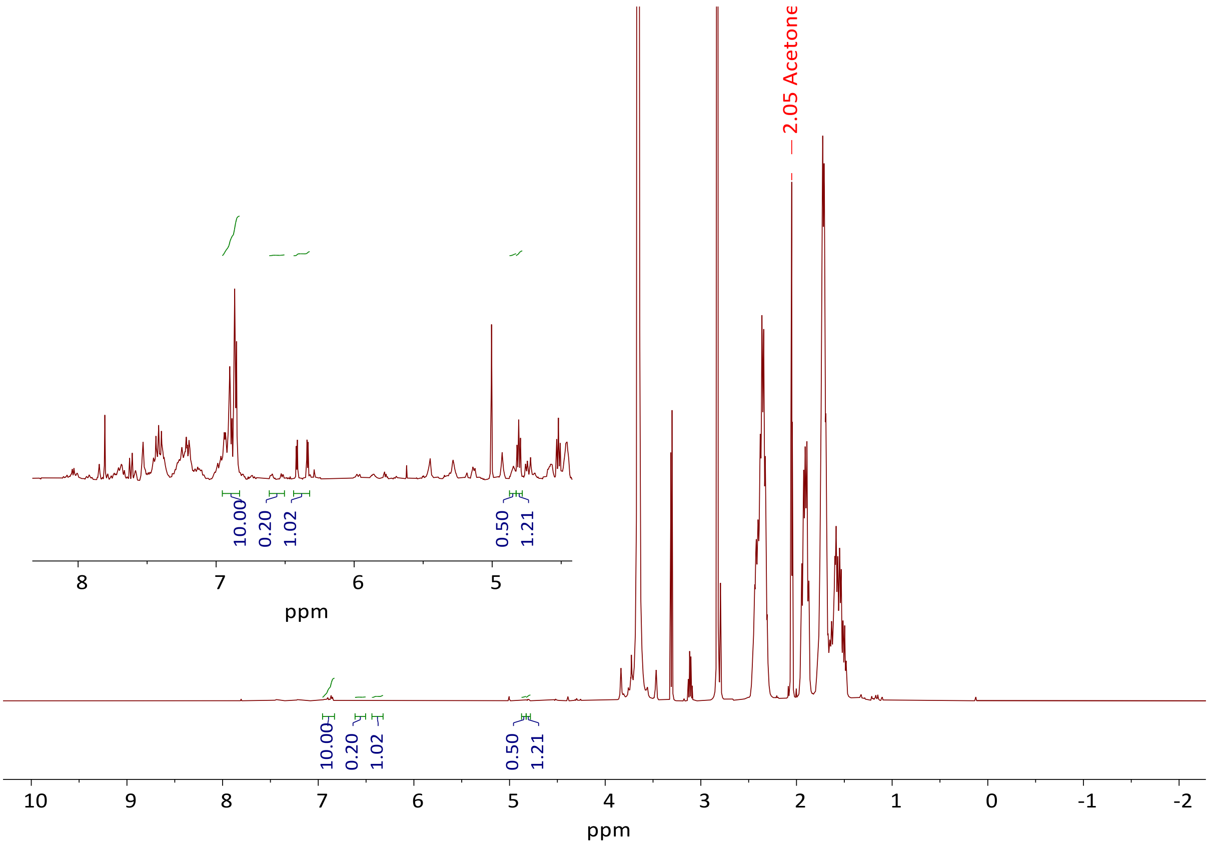

Spectrum S233. ^{1}H NMR (400 MHz, Acetone-d_6, 298 K) spectrum of post-sonication polymer **11**$_{\text{-127}}$ after being washed with methanol.

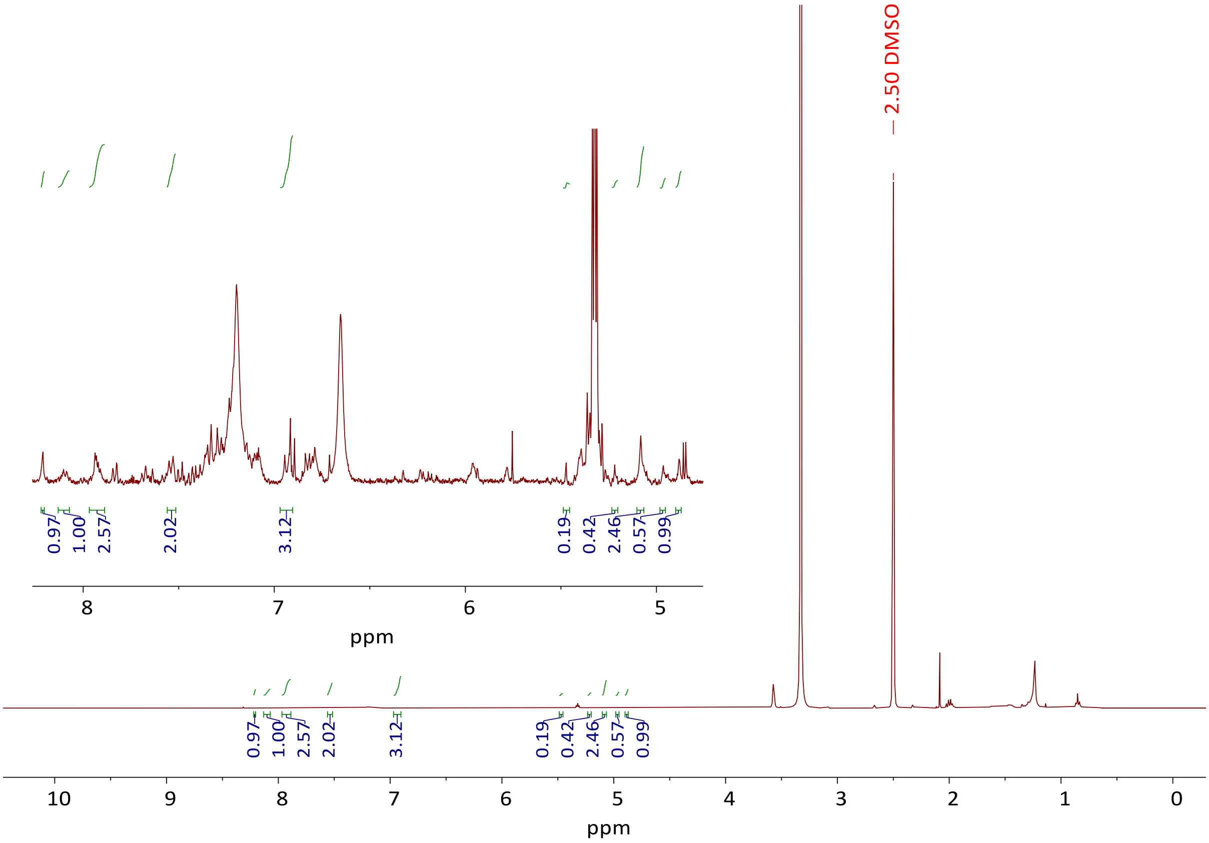

Spectrum S234. ^{1}H NMR (400 MHz, DMSO-d_6, 298 K) spectrum of the concentrated methanol washings from post-sonication polymer **11**$_{\text{-127}}$.

9.3.17 Post-Sonication ^{1}H NMR Spectra of Polymer 13$_{-119}$

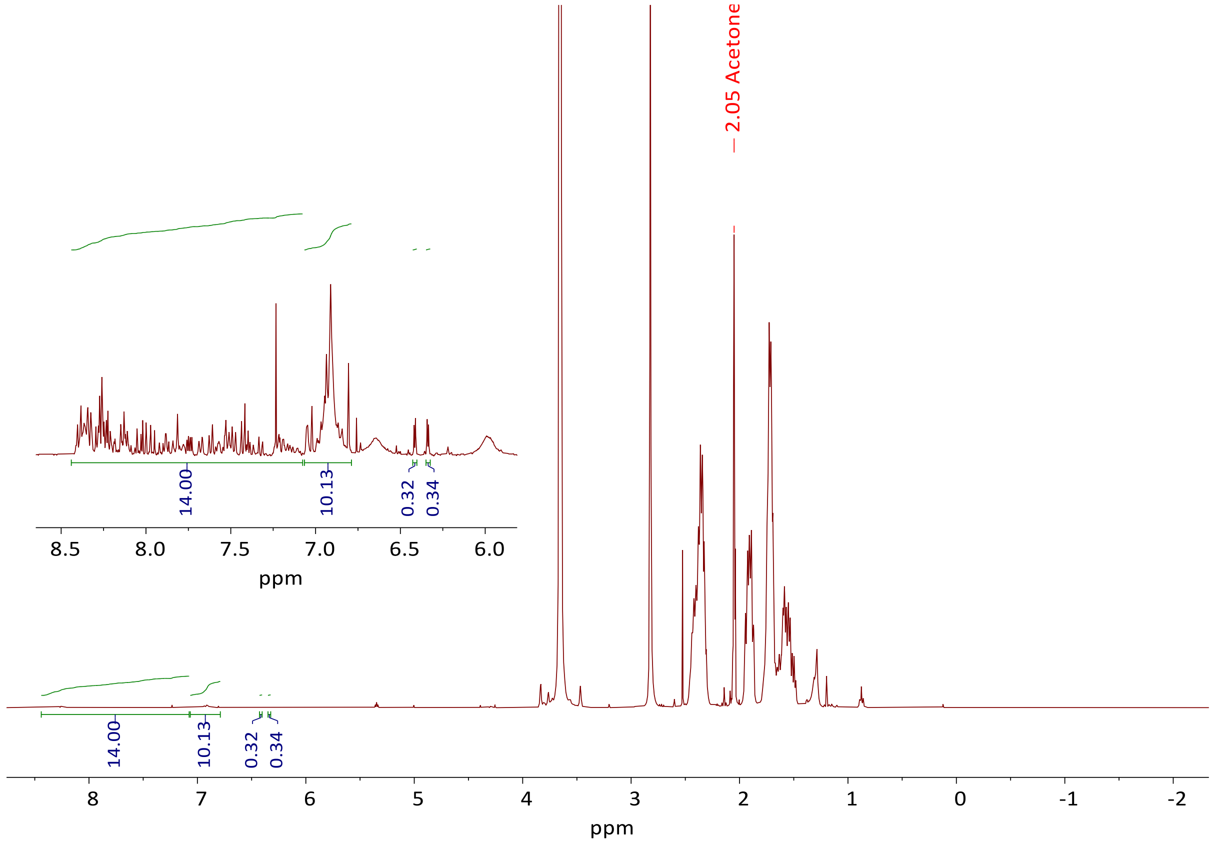

Spectrum S235. ^{1}H NMR (400 MHz, Acetone-d_6, 298 K) spectrum of post-sonication polymer **13**$_{-119}$ before being washed with methanol.

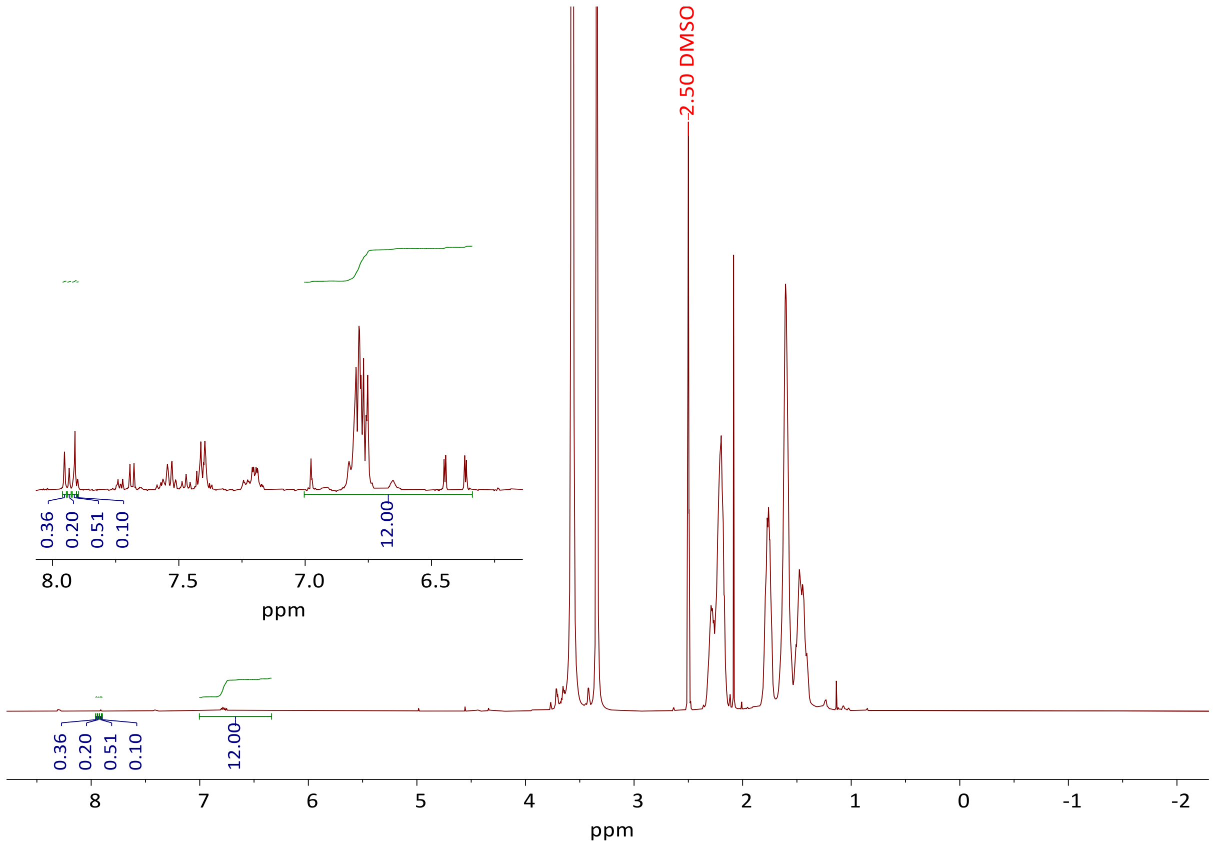

Spectrum S236. ^{1}H NMR (400 MHz, DMSO-d_6, 298 K) spectrum of post-sonication polymer **13**$_{\textbf{-119}}$ after being washed with methanol.

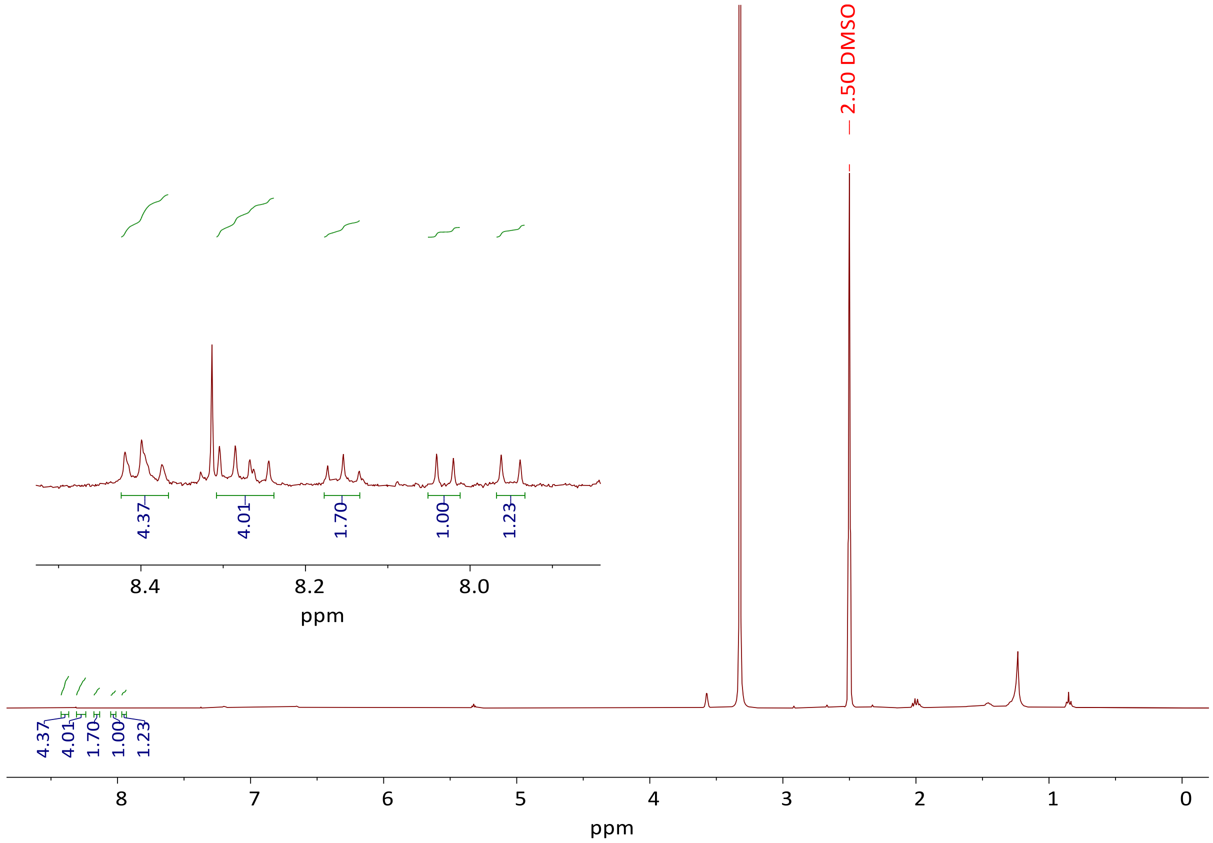

Spectrum S237. ^{1}H NMR (400 MHz, DMSO-d_6, 298 K) spectrum of the concentrated methanol washings from post-sonication polymer **13**$_{\textbf{-119}}$.

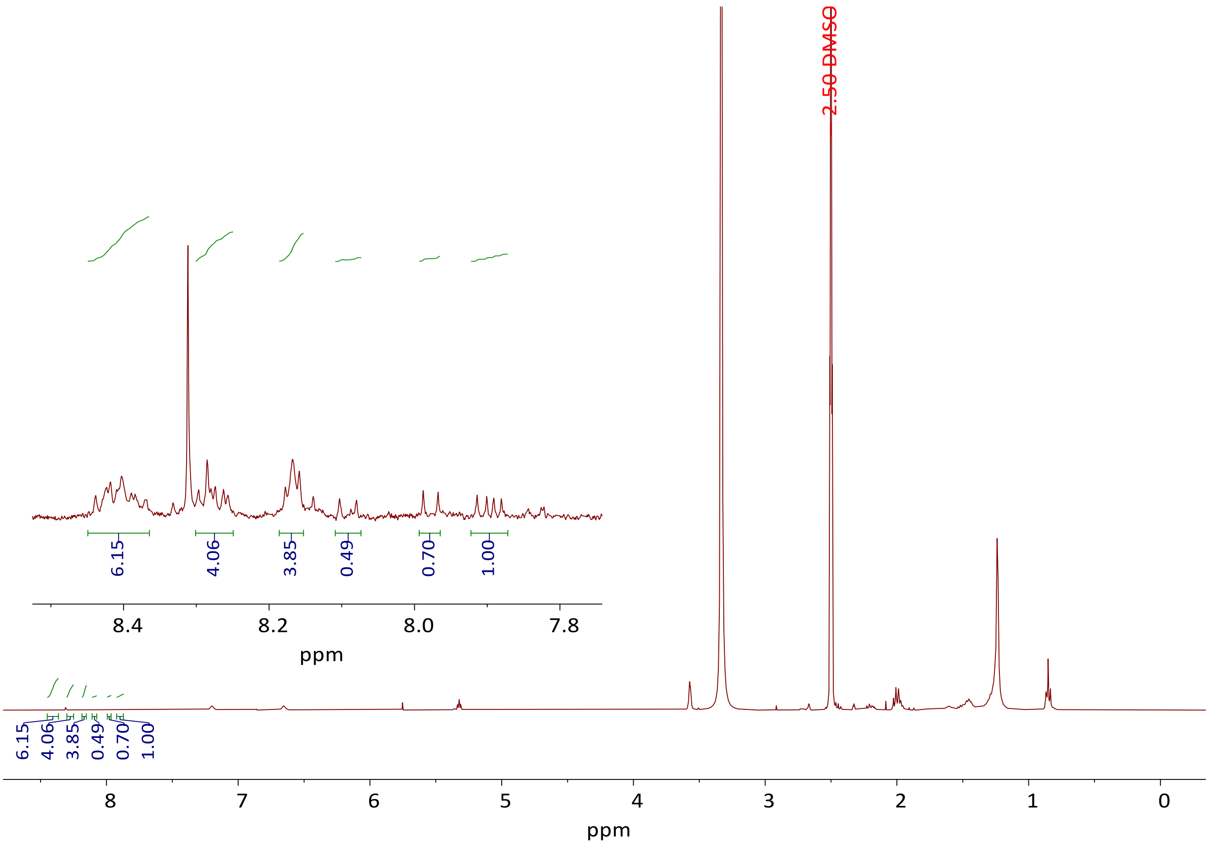

Spectrum S238. ^{1}H NMR (400 MHz, DMSO-d_6, 298 K) spectrum of the concentrated methanol washings from post-sonication polymer **13**$_{\text{-}119}$ after the addition of 1-dodecanethiol.

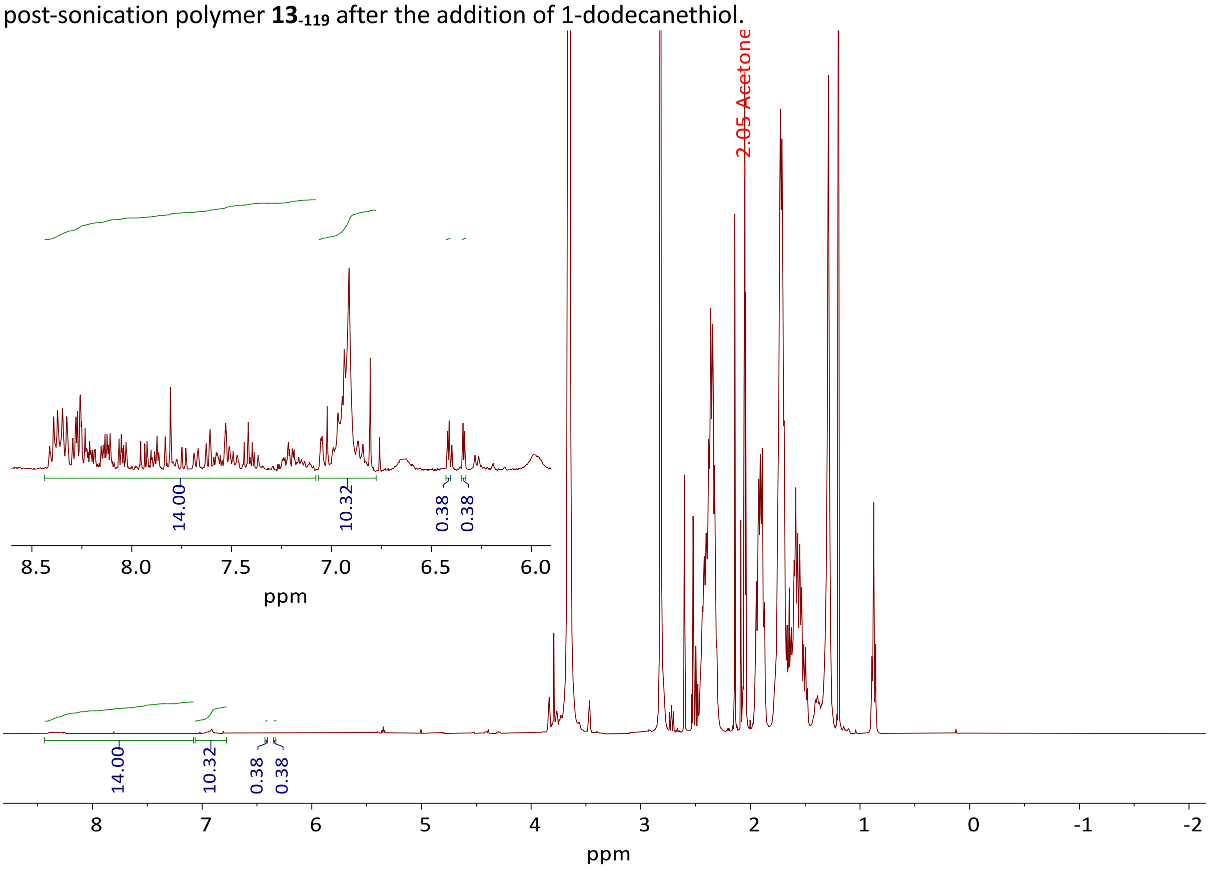

Spectrum S239. ^{1}H NMR (400 MHz, Acetone-d_6, 298 K) spectrum of post-sonication polymer **13**$_{\text{-}119}$ (having been sonicated in the presence of 1-dodecanethiol) before being washed with methanol.

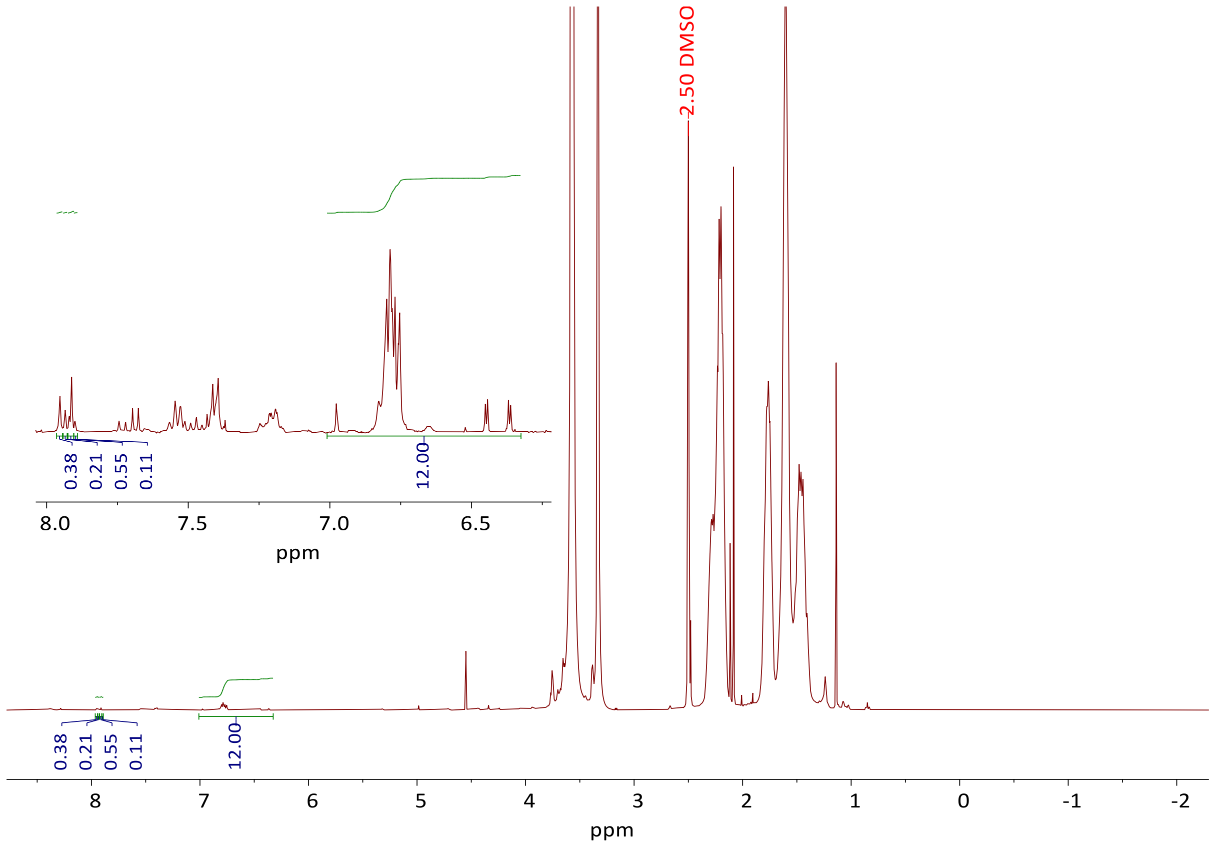

Spectrum S240. ^{1}H NMR (400 MHz, DMSO-d_6, 298 K) spectrum of post-sonication polymer **13-119** (having been sonicated in the presence of 1-dodecanethiol) after being washed with methanol.

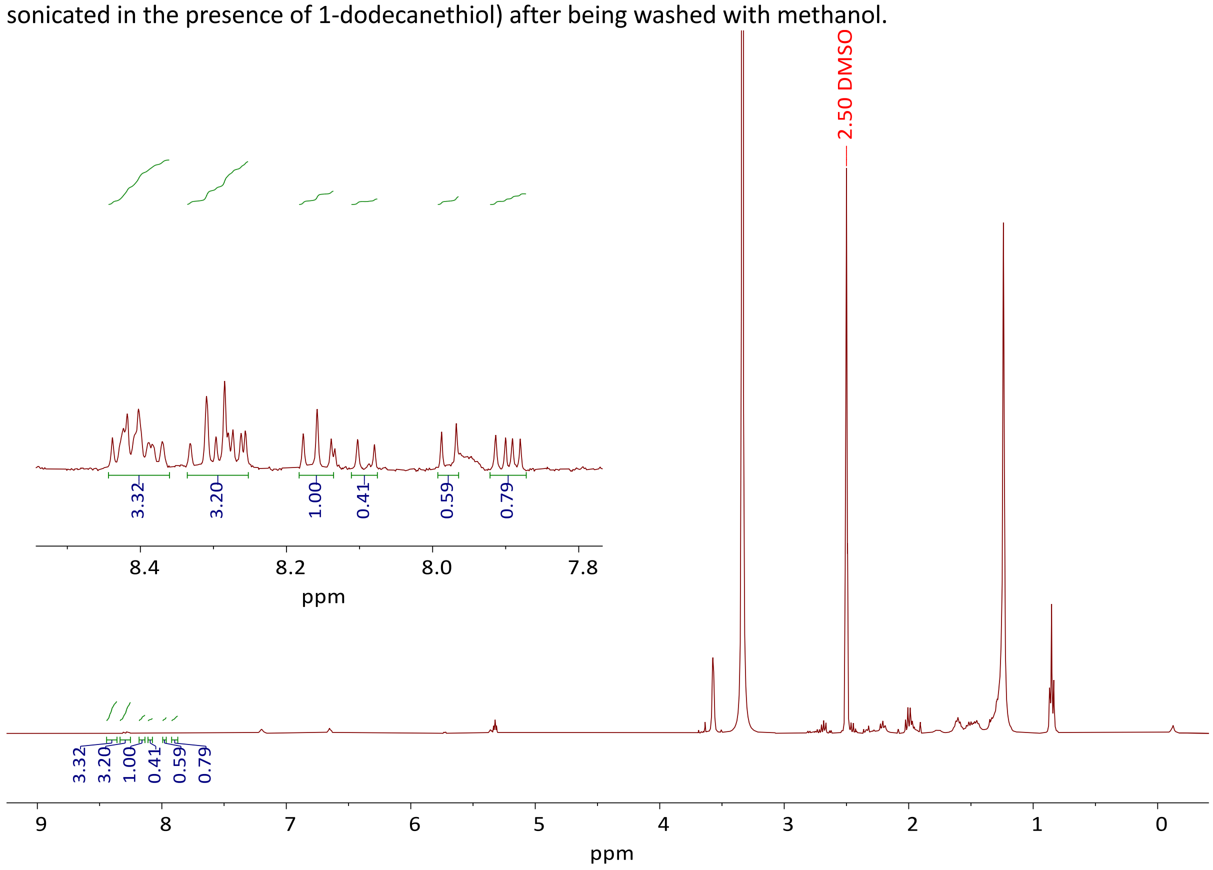

Spectrum S241. ^{1}H NMR (400 MHz, DMSO-d_6, 298 K) spectrum of the concentrated methanol washings from post-sonication polymer **13-119** (having been sonicated in the presence of 1-dodecanethiol).

9.3.18 Post-Sonication ^{1}H NMR Spectra of Polymer S59$_{-77}$

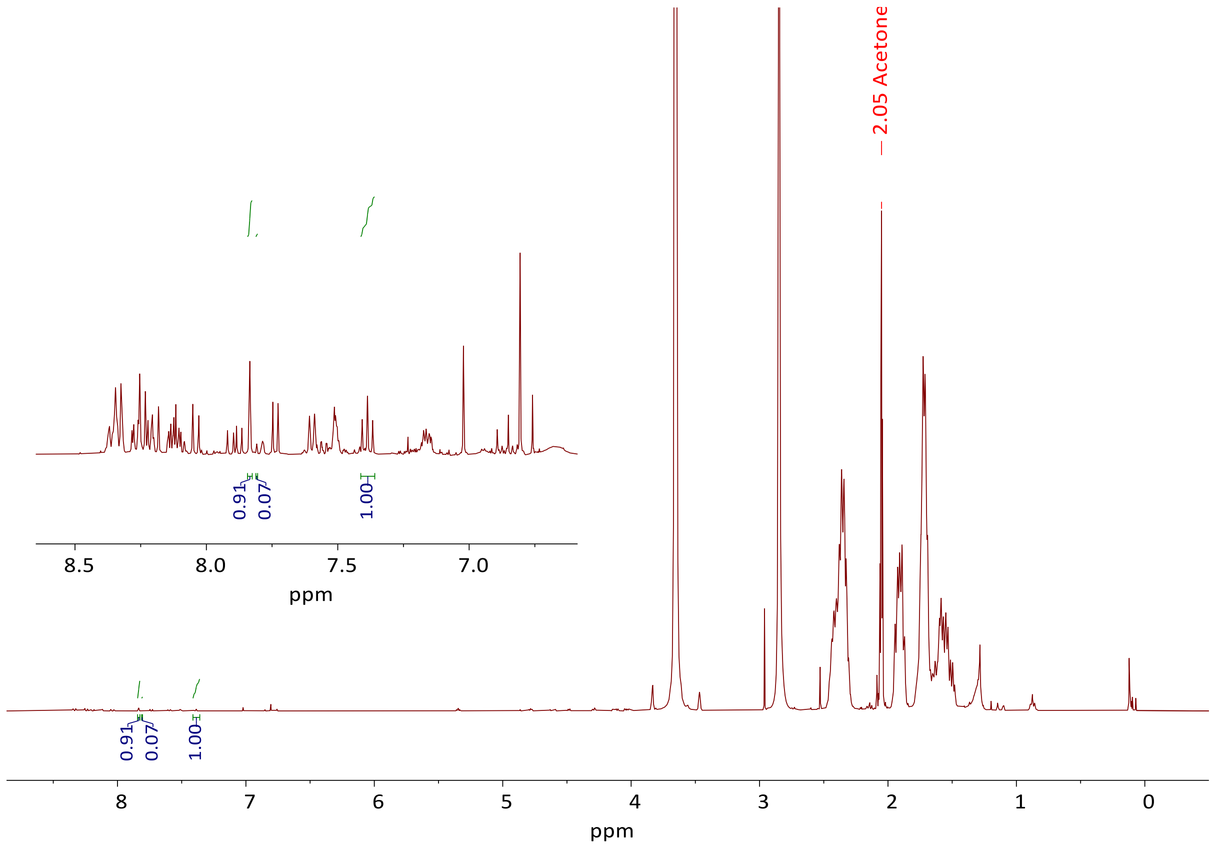

Spectrum S242. ^{1}H NMR (400 MHz, Acetone-d_6, 298 K) spectrum of post-sonication polymer **S59$_{-77}$** before being washed with methanol.

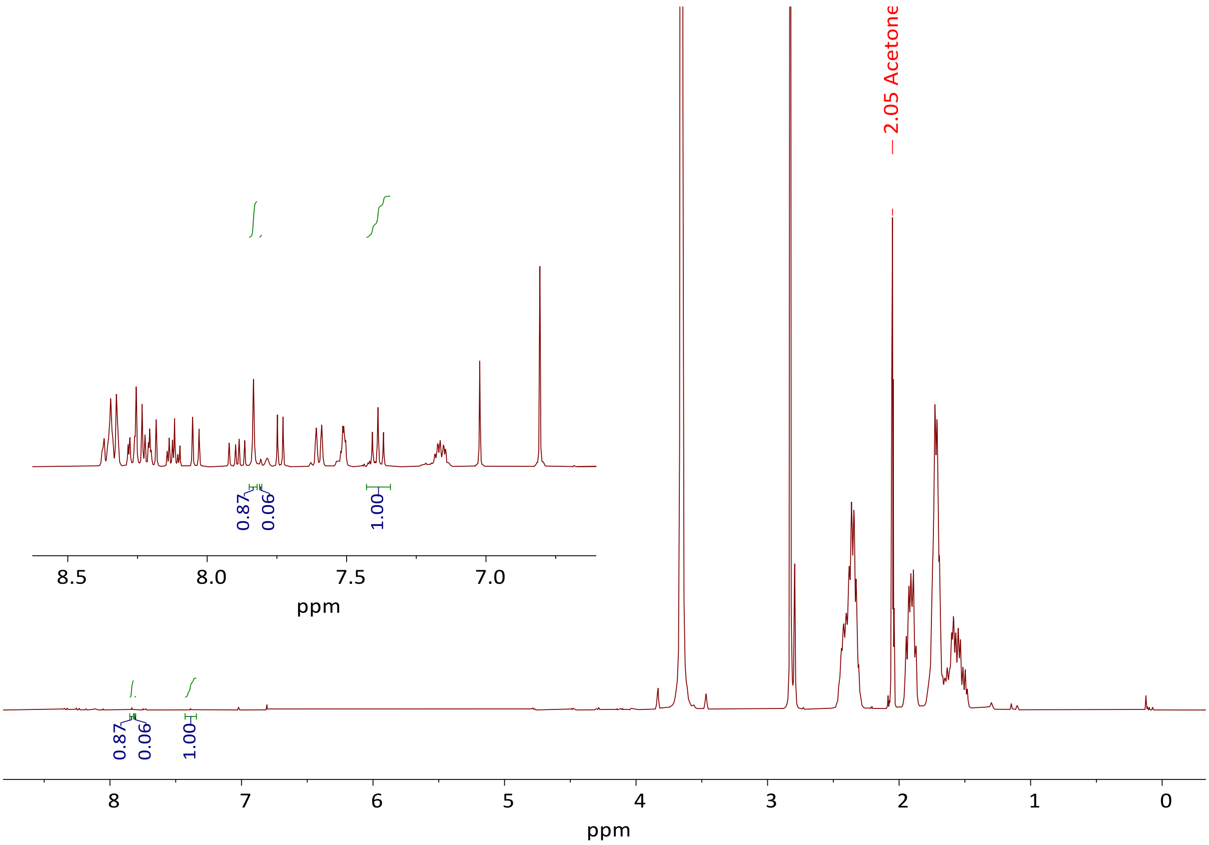

Spectrum S243. ^{1}H NMR (400 MHz, Acetone-d_6, 298 K) spectrum of post-sonication polymer **S59$_{-77}$** after being washed with methanol.

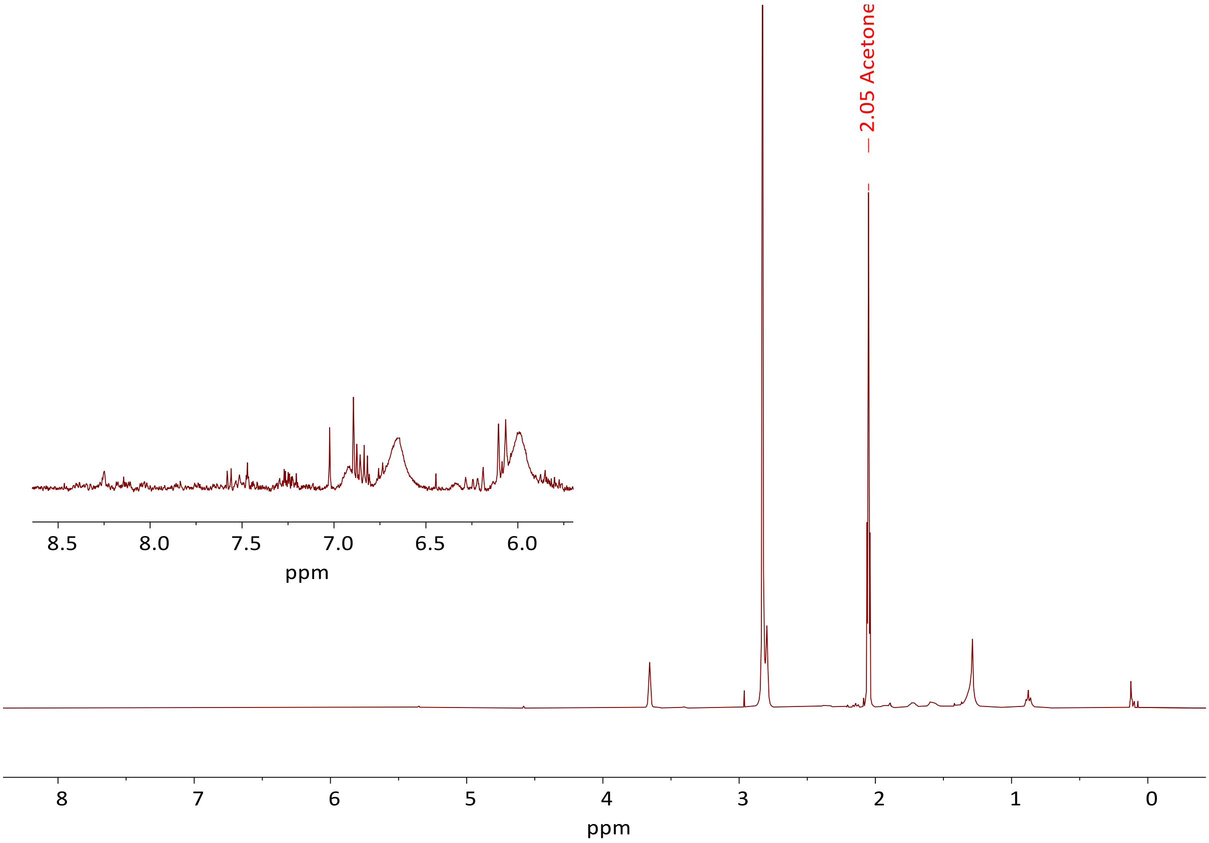

Spectrum S244. ^{1}H NMR (400 MHz, Acetone-d_6, 298 K) spectrum of the concentrated methanol washings from post-sonication polymer **S59$_{-77}$**.

9.3.19 Post-Sonication ^{1}H NMR Spectra of Polymer 14$_{-124}$

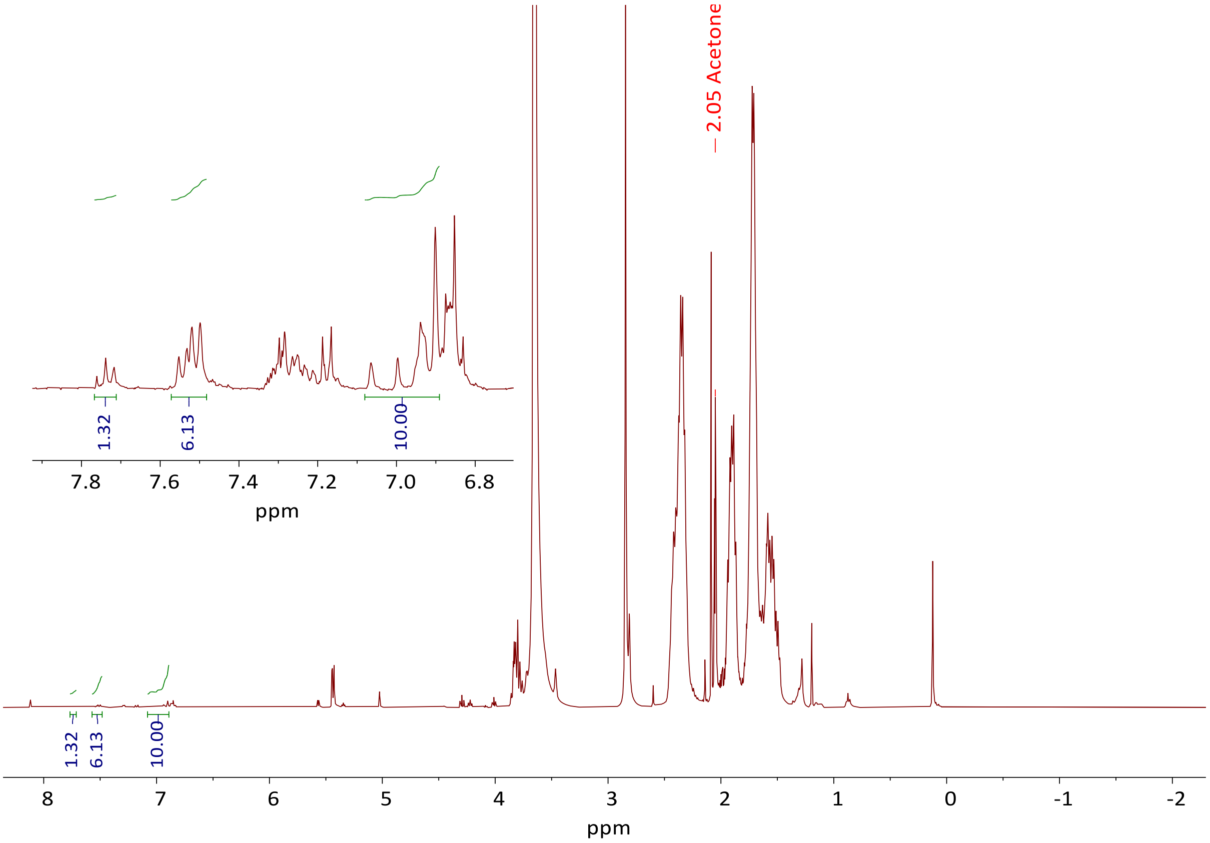

Spectrum S245. ^{1}H NMR (400 MHz, Acetone-d_6, 298 K) spectrum of post-sonication polymer **14$_{-124}$** before being washed with methanol.

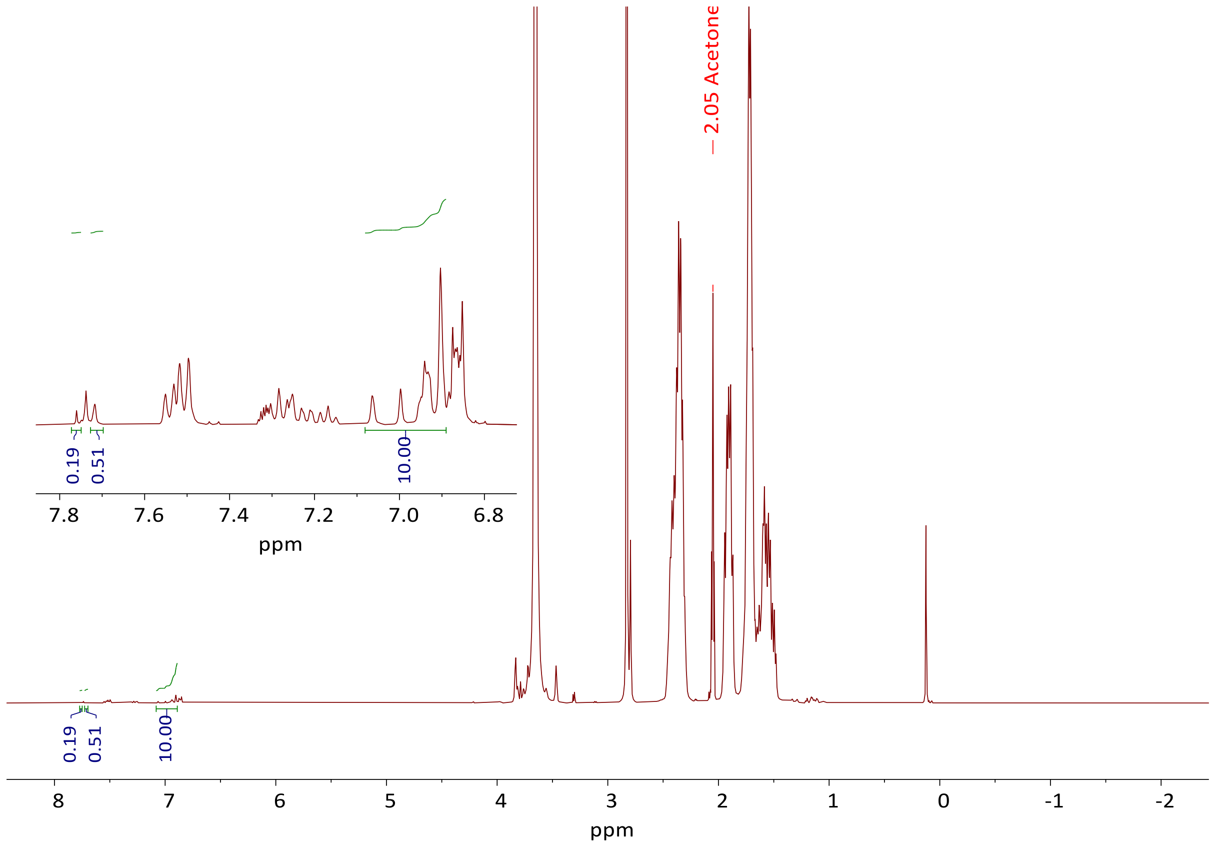

Spectrum S246. ^{1}H NMR (400 MHz, Acetone-d_6, 298 K) spectrum of post-sonication polymer **14**$_{\mathbf{-124}}$ after being washed with methanol.

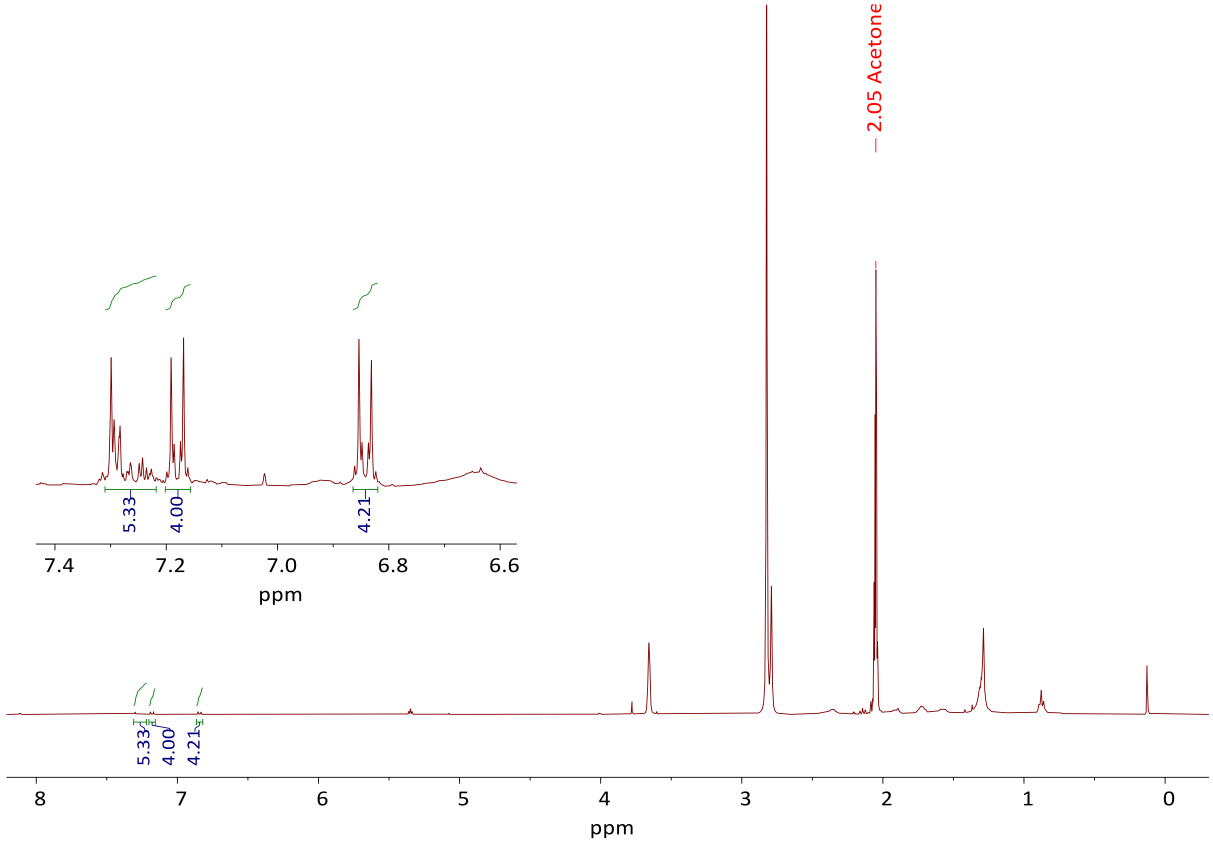

Spectrum S247. ^{1}H NMR (400 MHz, Acetone-d_6, 298 K) spectrum of the concentrated methanol washings from post-sonication polymer **14**$_{\mathbf{-124}}$.

9.3.20 Post-Sonication ^{1}H NMR Spectra of Polymer S60$_{-95}$

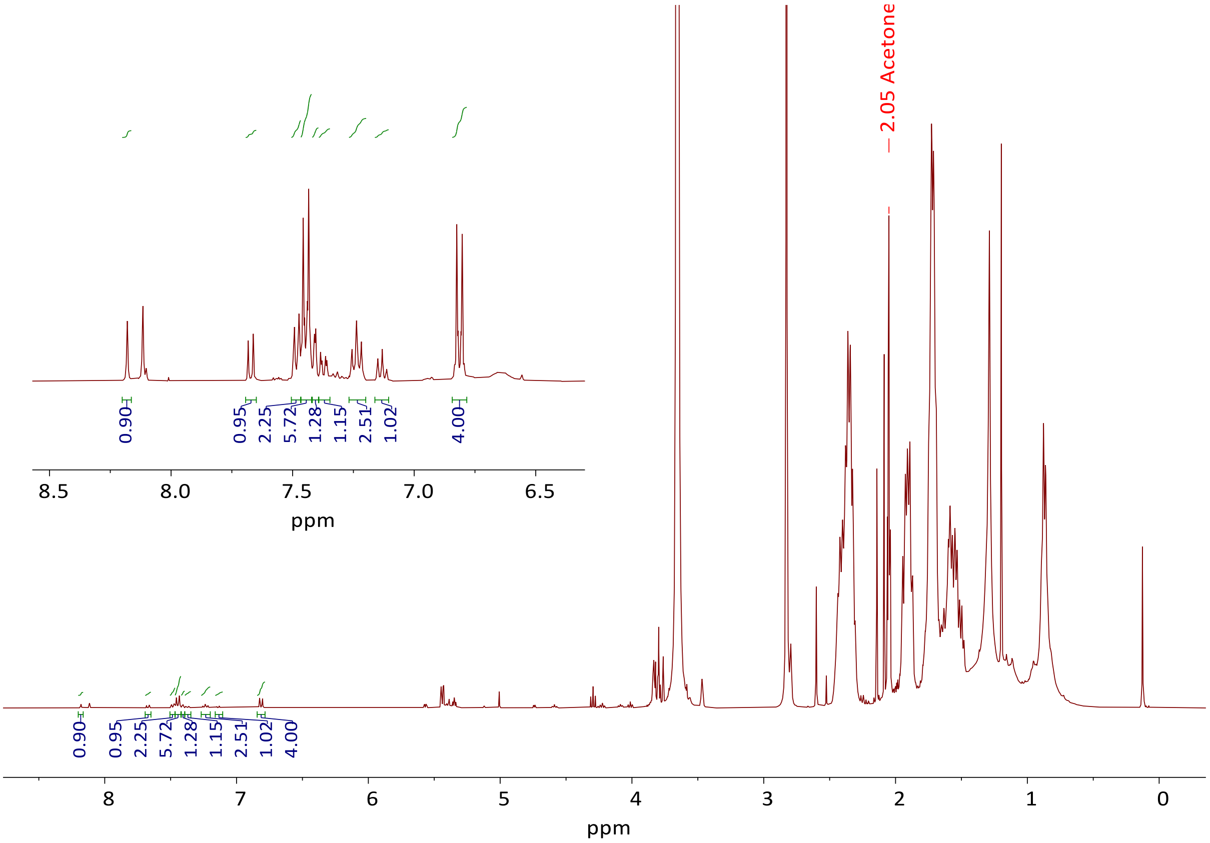

Spectrum S248. ^{1}H NMR (400 MHz, Acetone-d_6, 298 K) spectrum of post-sonication polymer **S60$_{-95}$** before being washed with methanol.

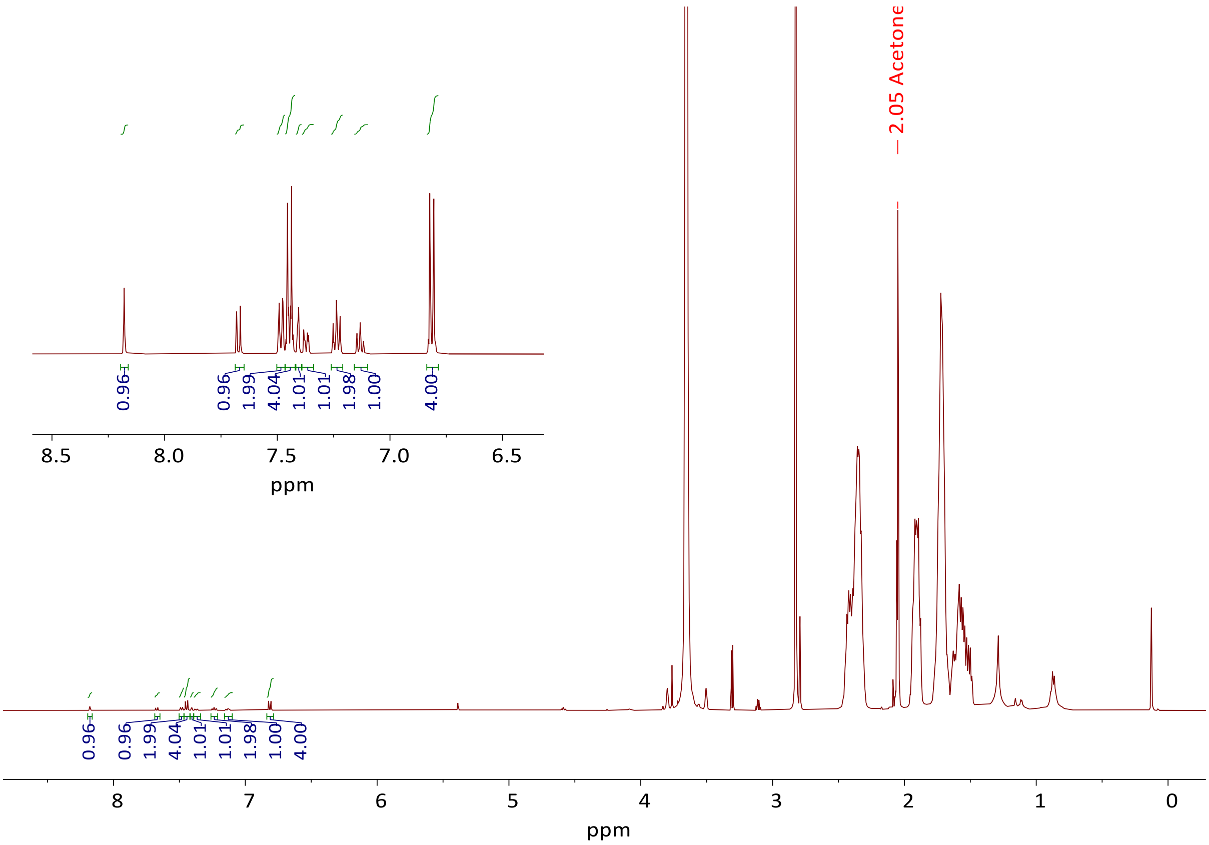

Spectrum S249. ^{1}H NMR (400 MHz, Acetone-d_6, 298 K) spectrum of post-sonication polymer **S60$_{-95}$** after being washed with methanol.

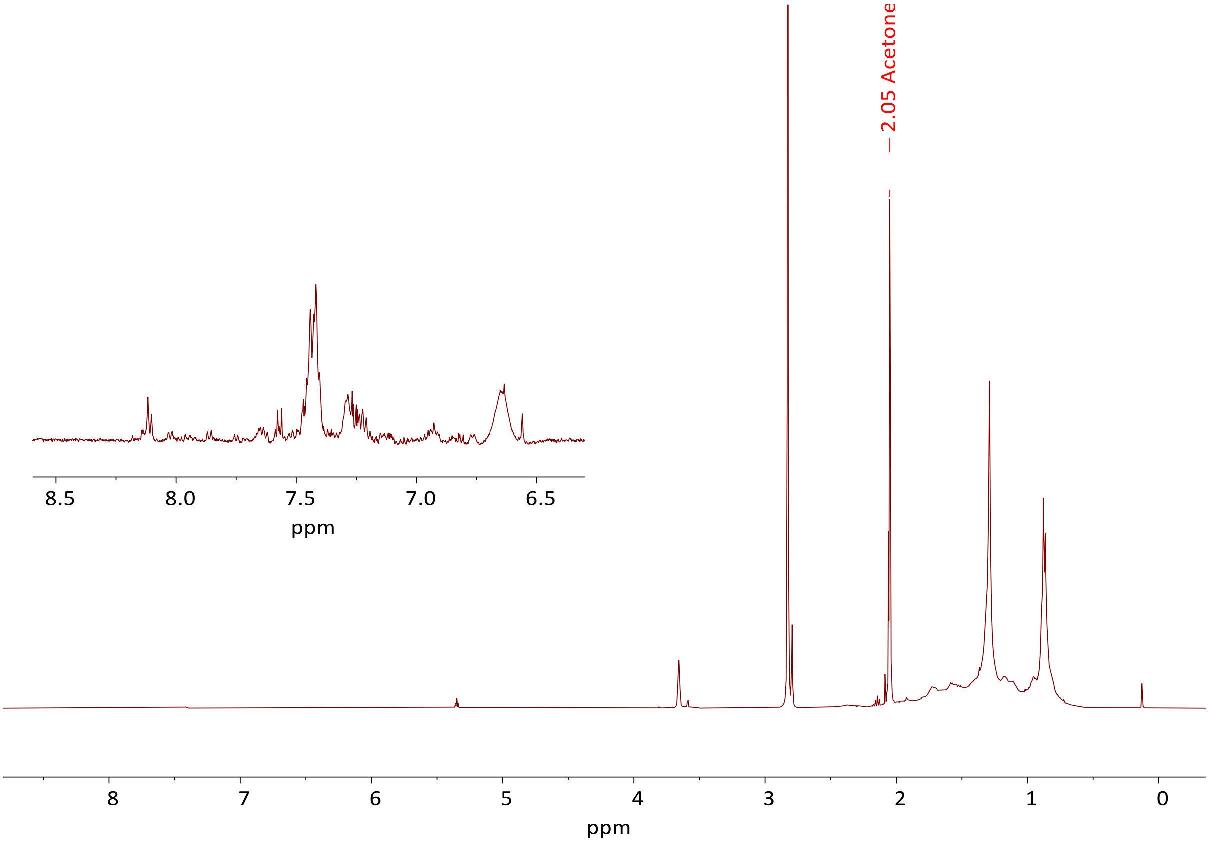

Spectrum S250. ^{1}H NMR (400 MHz, Acetone-d_6, 298 K) spectrum of the concentrated methanol washings from post-sonication polymer **S60$_{-95}$**.

9.4 Post-Solid-State-Activation NMR Spectra

9.4.1 Post-Solid-State-Activation ^{1}H NMR of Control Polymer S55$_{exo\text{-}112}$

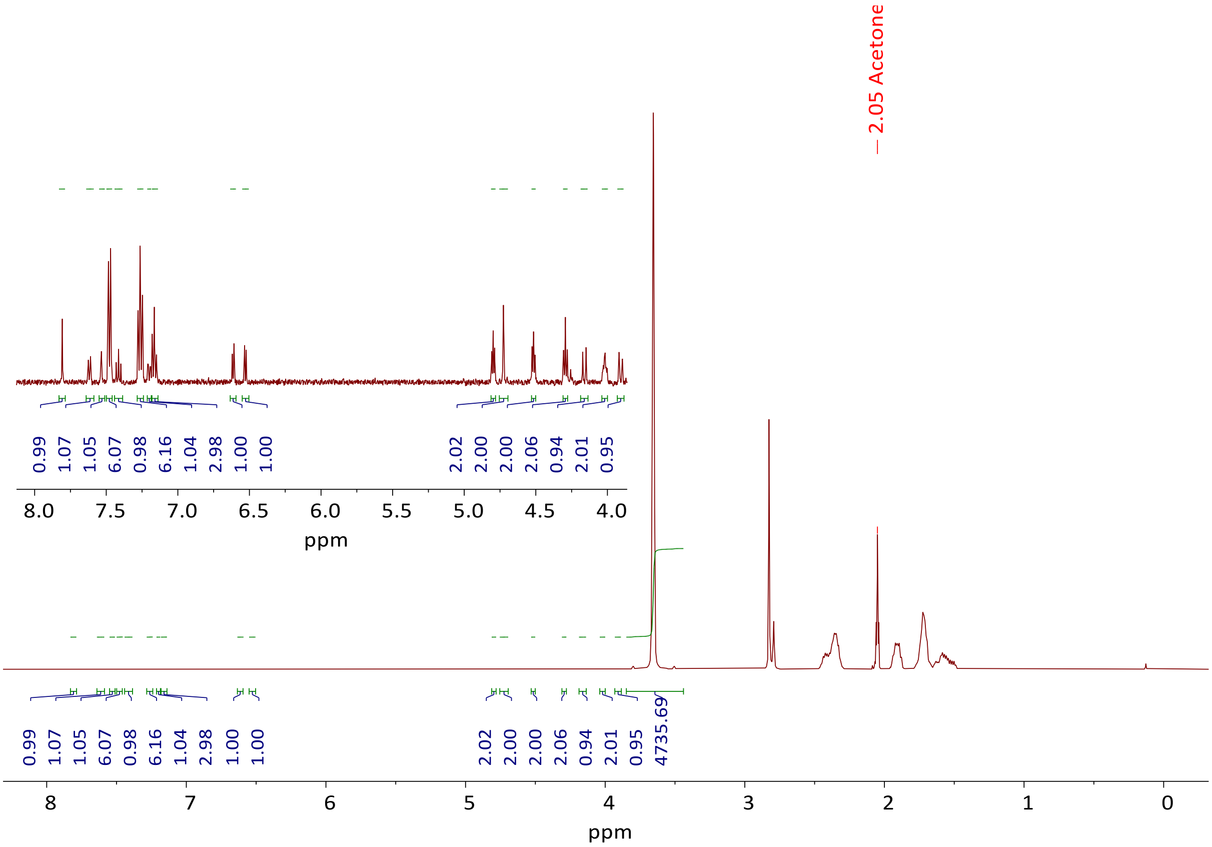

Spectrum S251. ^{1}H NMR (400 MHz, Acetone-d_6, 298 K) spectrum of post-solid-state-activation polymer **S55$_{exo\text{-}112}$** after being washed with methanol.

9.4.2 Post-Solid-State-Activation ^{1}H NMR of Polymer $\mathbf{9}_{trans/exo\text{-}114}$

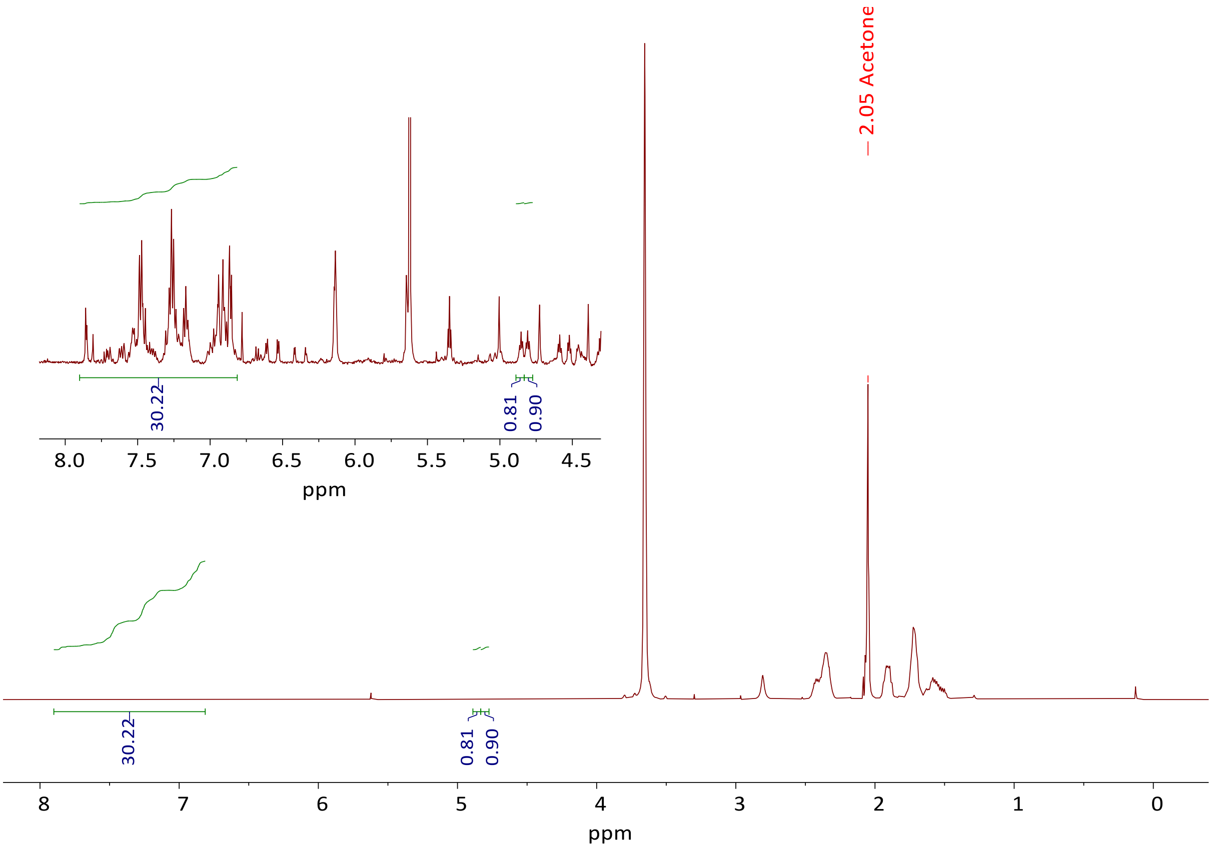

Spectrum S252. ^{1}H NMR (500 MHz, Acetone-d_6, 298 K) spectrum of post-solid-state-activation polymer $\mathbf{9}_{trans/exo\text{-}114}$ before being washed with methanol.

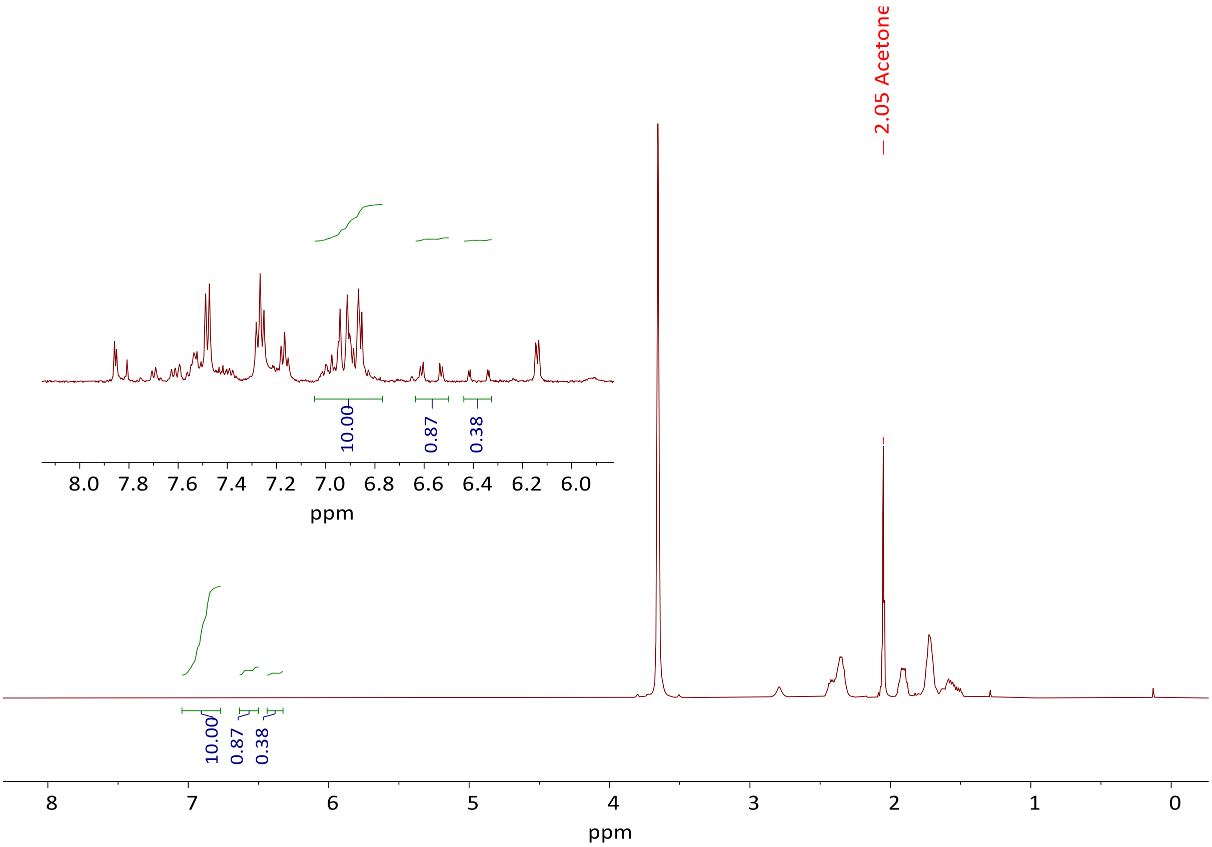

Spectrum S253. ^{1}H NMR (500 MHz, Acetone-d_6, 298 K) spectrum of post-solid-state-activation polymer **9***trans/exo-114* after being washed with methanol.

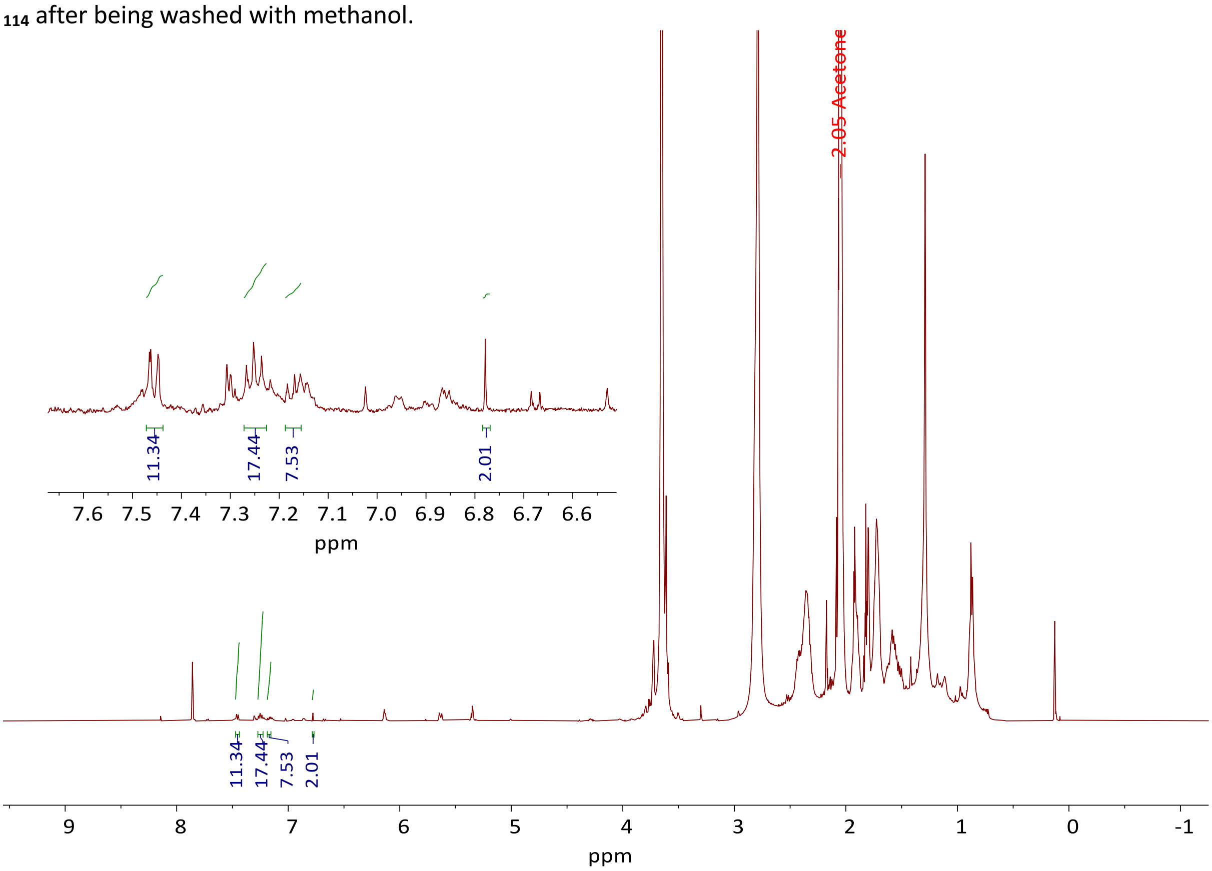

Spectrum S254. ^{1}H NMR (500 MHz, Acetone-d_6, 298 K) spectrum of the concentrated methanol washings from post-solid-state-activation polymer **9***trans/exo-114*.

9.4.3 Post-Solid-State-Activation ^{1}H NMR of 1_3a_{-210}

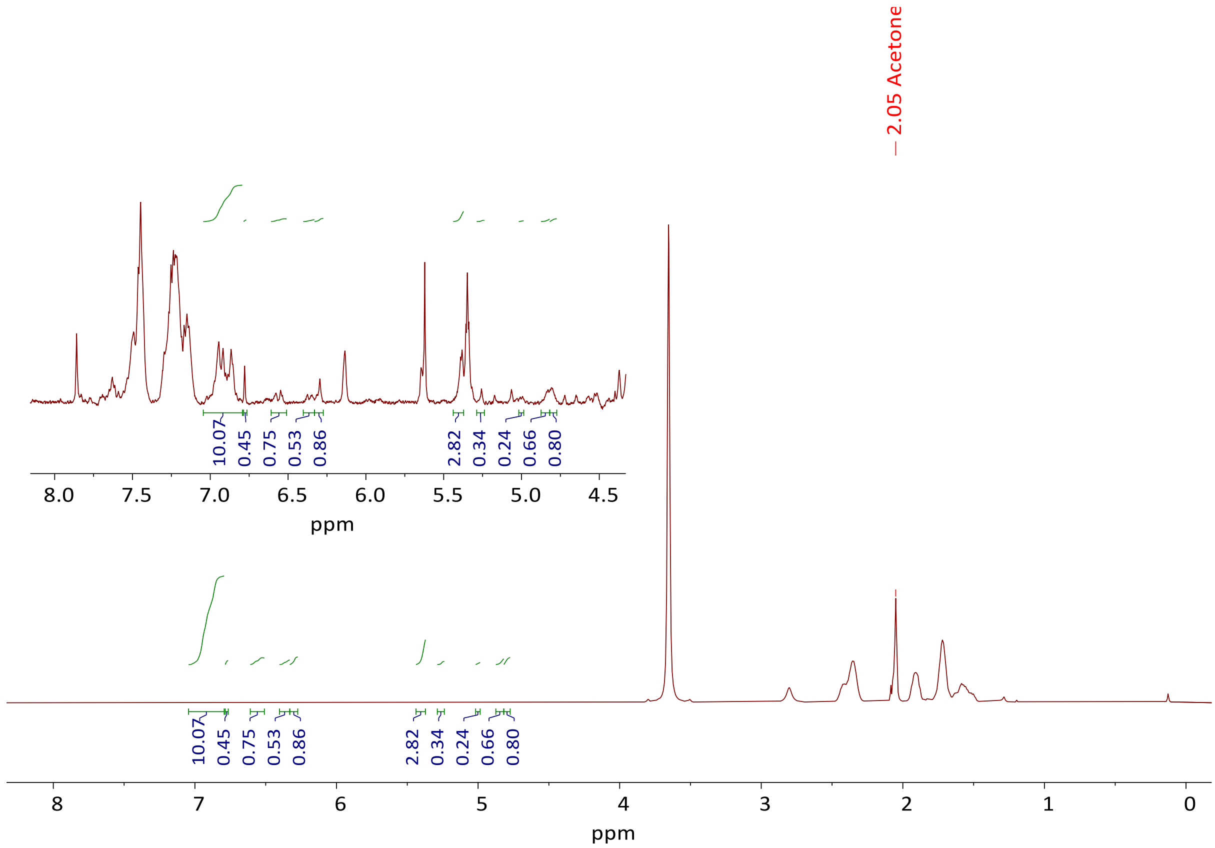

Spectrum S255. ^{1}H NMR (500 MHz, Acetone-d_6, 298 K) spectrum of post-solid-state-activation polymer $\mathbf{1_3a_{-210}}$ before being washed with methanol.

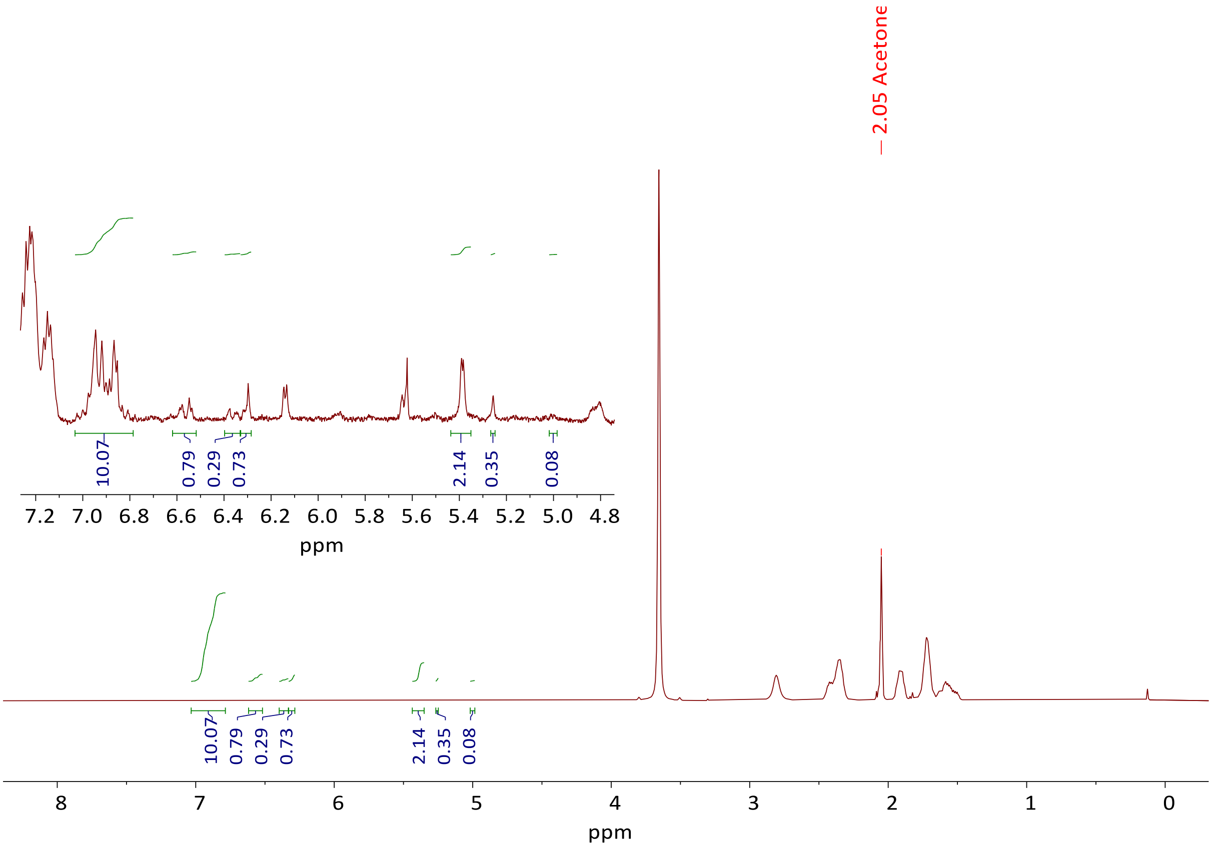

Spectrum S256. ^{1}H NMR (500 MHz, Acetone-d_6, 298 K) spectrum of post-solid-state-activation polymer **1₃a₋₂₁₀** after being washed with methanol.

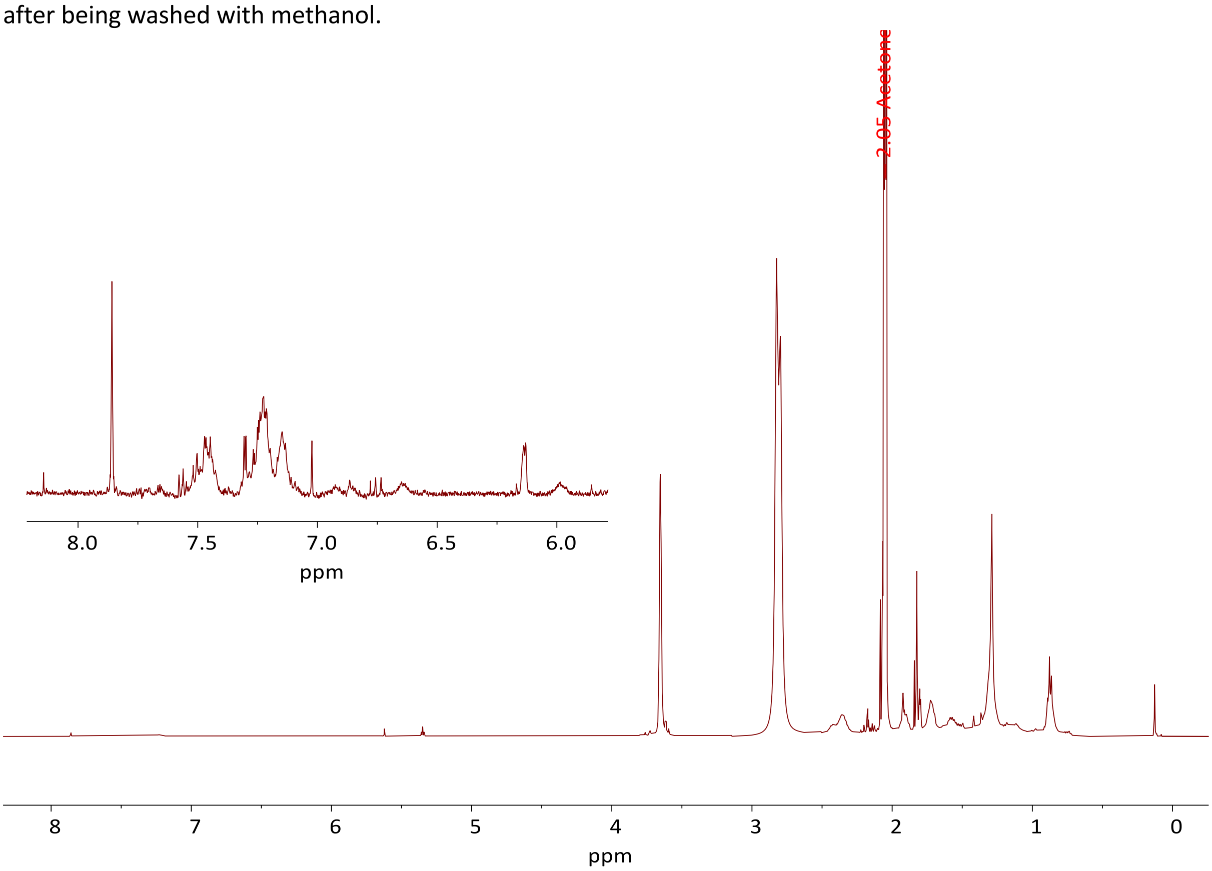

Spectrum S257. ^{1}H NMR (500 MHz, Acetone-d_6, 298 K) spectrum of the concentrated methanol washings from post-solid-state-activation polymer **1_3a_{-210}**.

9.4.4 Post-Solid-State-Activation ^{1}H NMR of Polymer $1_{5\text{-}165}$

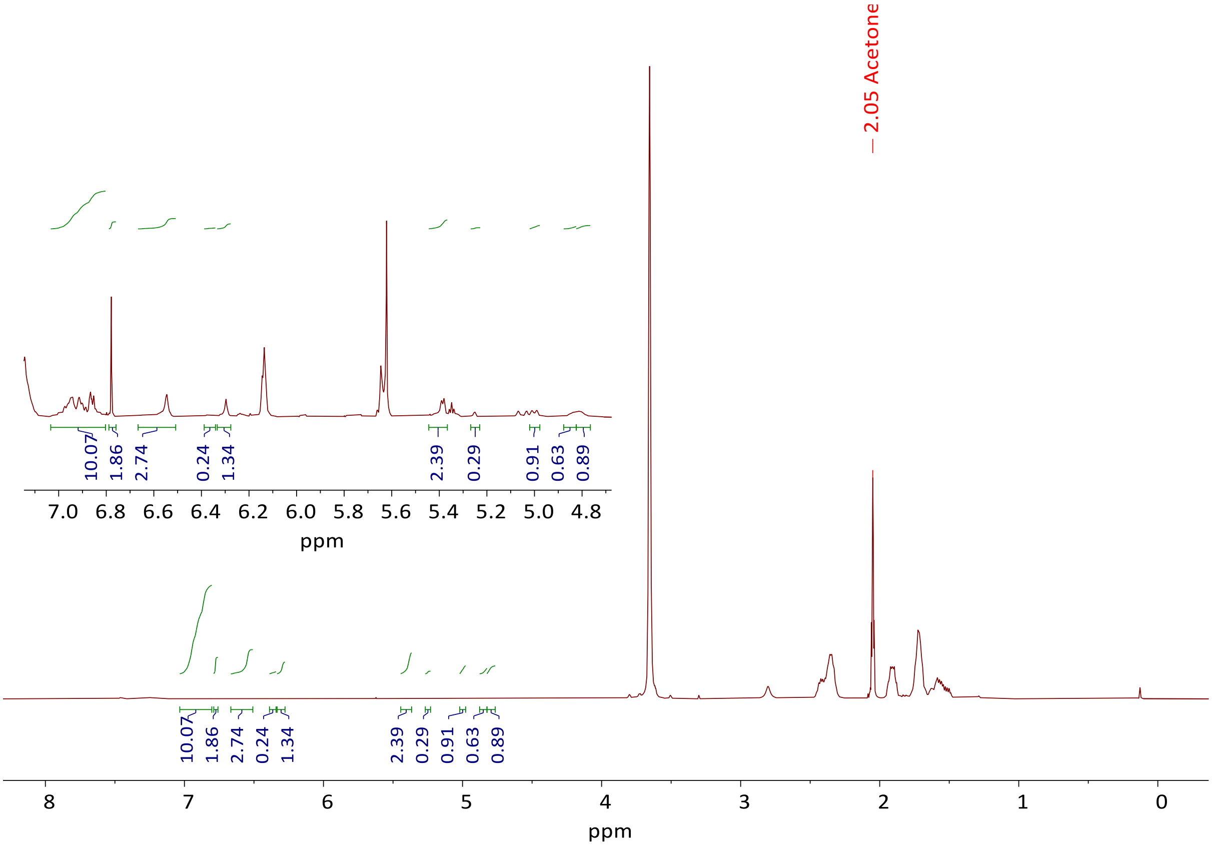

Spectrum S258. ^{1}H NMR (400 MHz, Acetone-d_6, 298 K) spectrum of post-solid-state-activation polymer $\mathbf{1}_{5\text{-}165}$ before being washed with methanol.

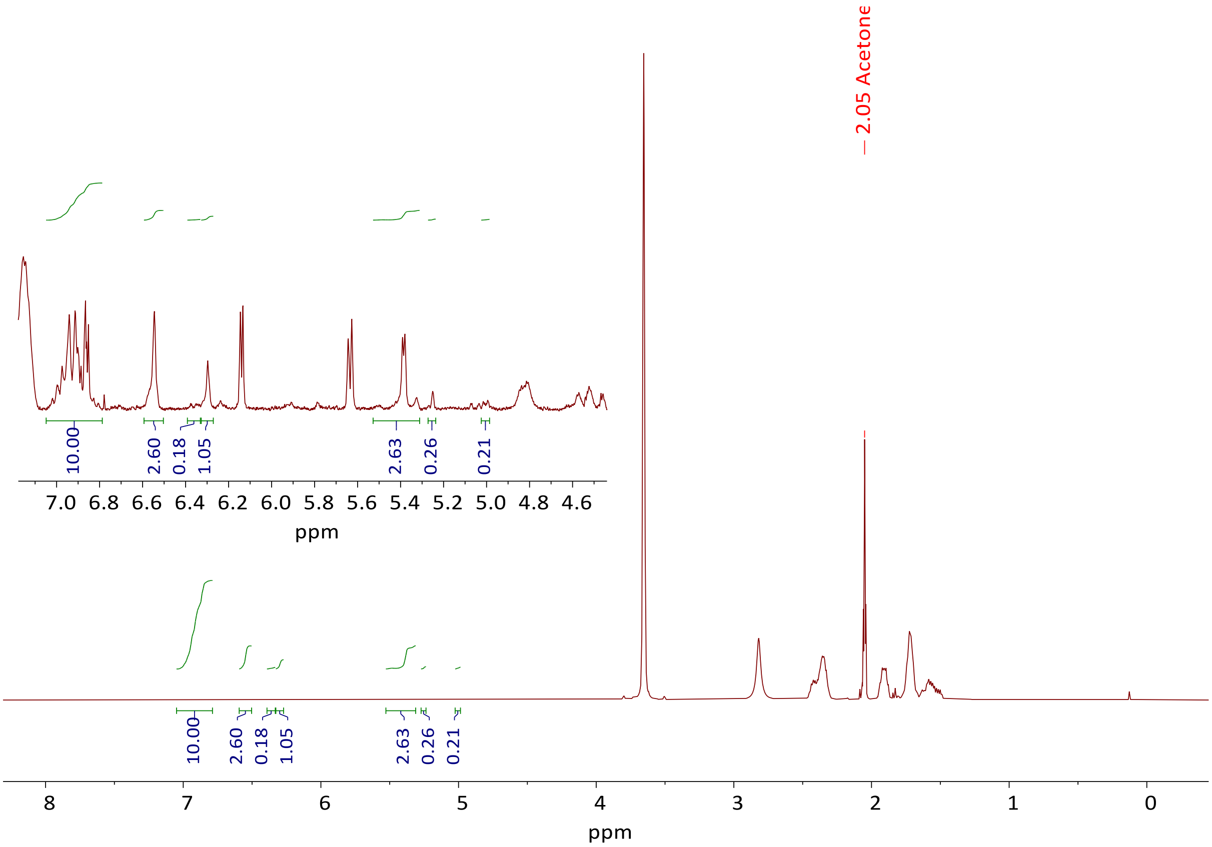

Spectrum S259. ^{1}H NMR (400 MHz, Acetone-d_6, 298 K) spectrum of post-solid-state-activation polymer **1**$_{5\text{-}165}$ after being washed with methanol.

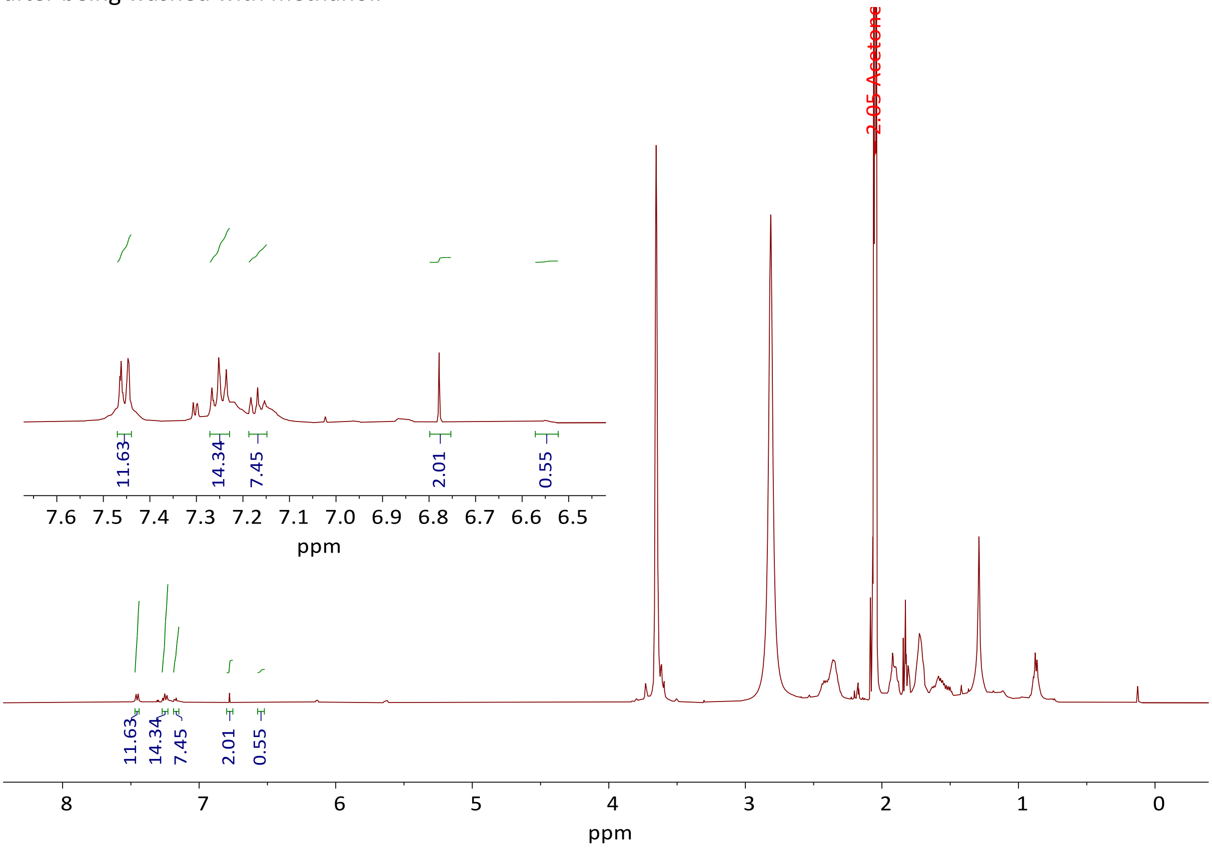

Spectrum S260. ^{1}H NMR (400 MHz, Acetone-d_6, 298 K) spectrum of the concentrated methanol washings from post-solid-state-activation polymer **1**$_{5\text{-}165}$.

9.4.5 Post-Solid-State-Activation ^{1}H NMR of Polymer $\mathbf{1}_{5\text{-}215}$

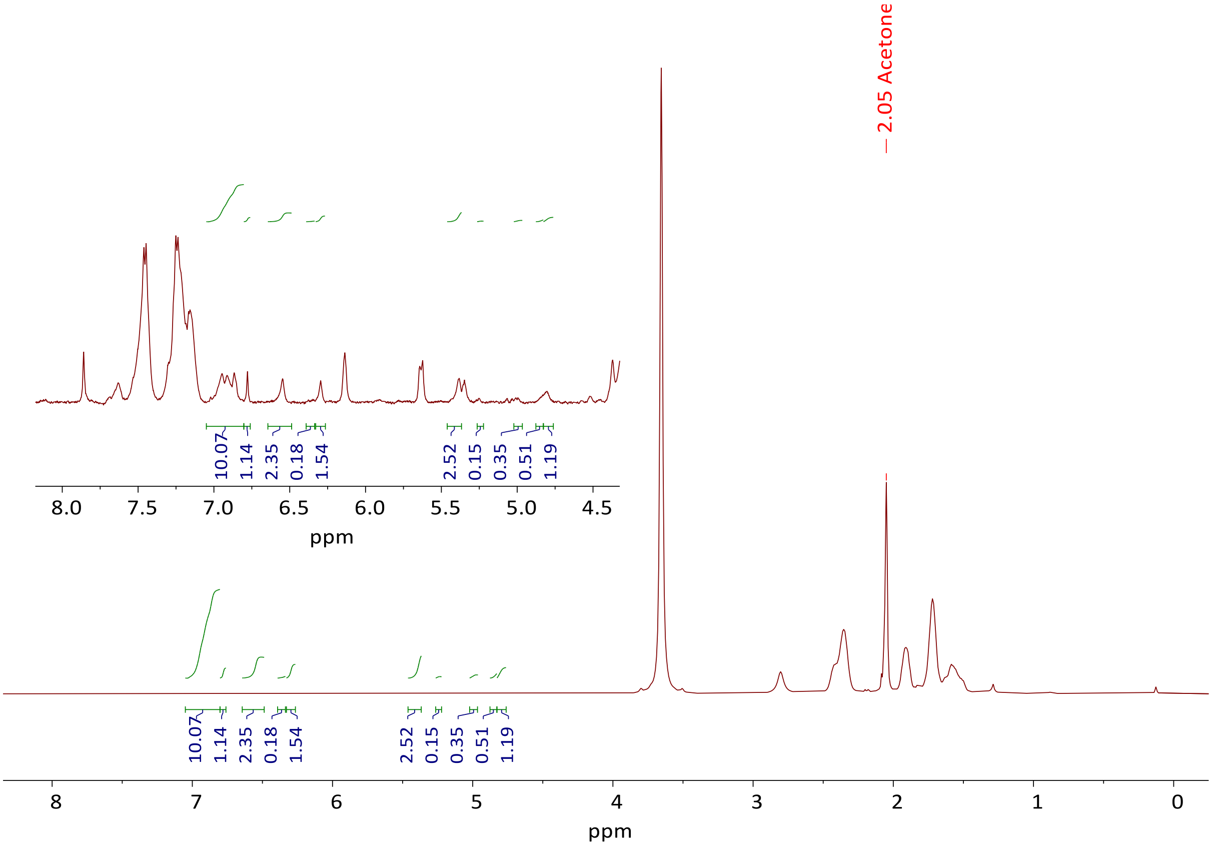

Spectrum S261. ^{1}H NMR (400 MHz, Acetone-d_6, 298 K) spectrum of post-solid-state-activation polymer $\mathbf{1}_{5\text{-}215}$ before being washed with methanol.

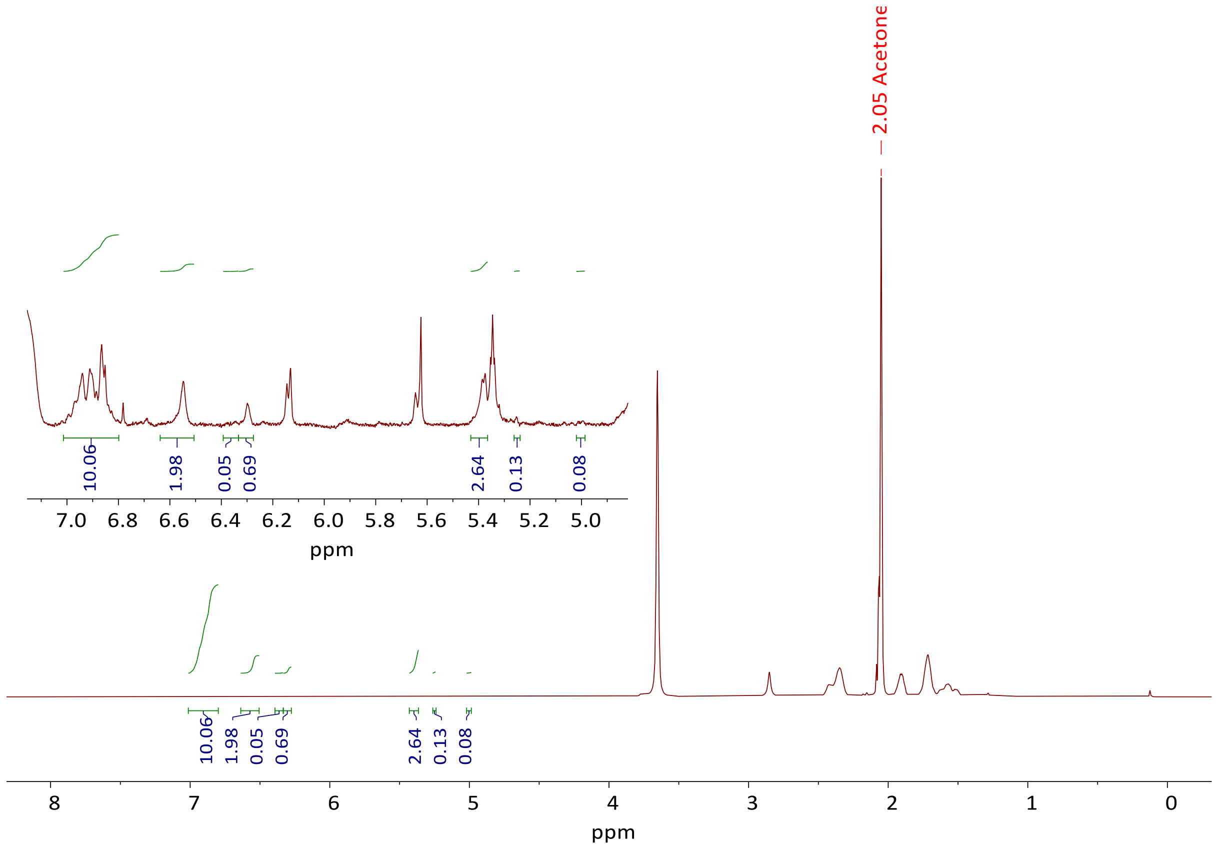

Spectrum S262. ^{1}H NMR (400 MHz, Acetone-d_6, 298 K) spectrum of post-solid-state-activation polymer $\mathbf{1}_{5\text{-}215}$ after being washed with methanol.

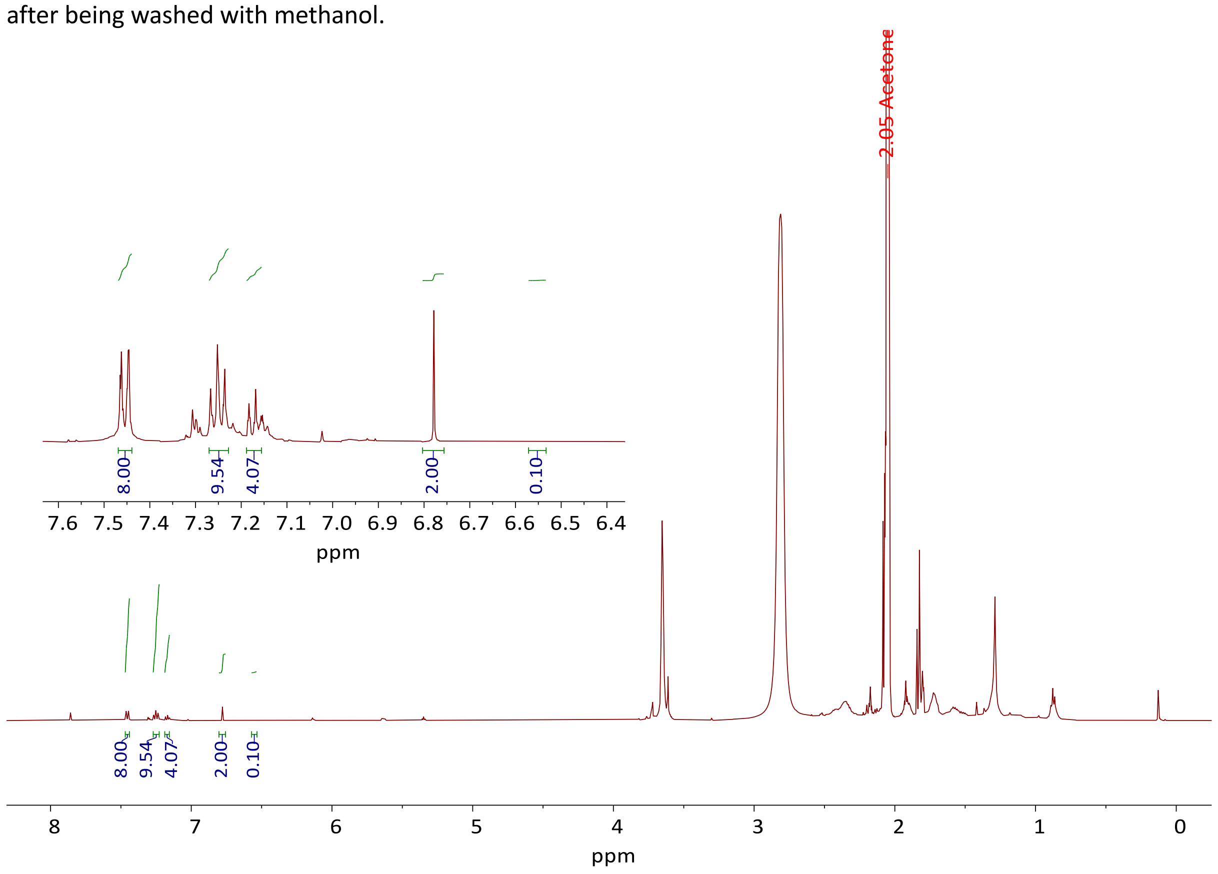

Spectrum S263. ^{1}H NMR (400 MHz, Acetone-d_6, 298 K) spectrum of the concentrated methanol washings from post-solid-state-activation polymer $\mathbf{1}_{5\text{-}215}$.

9.4.6 Post-Solid-State-Activation ^1H NMR of Polymer blend S30

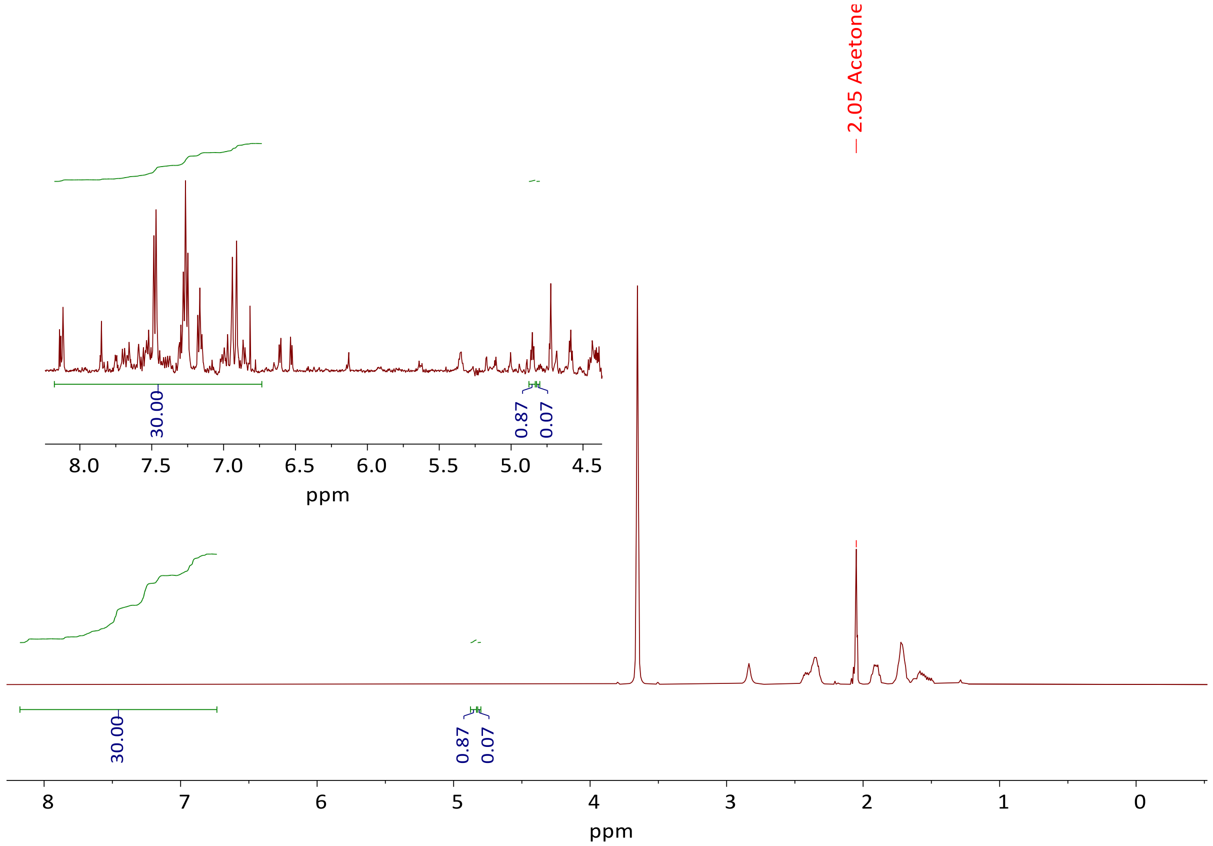

Spectrum S264. ^1H NMR (400 MHz, Acetone-d_6, 298 K) spectrum of post-solid-state-activation polymer **S30** before being washed with methanol.

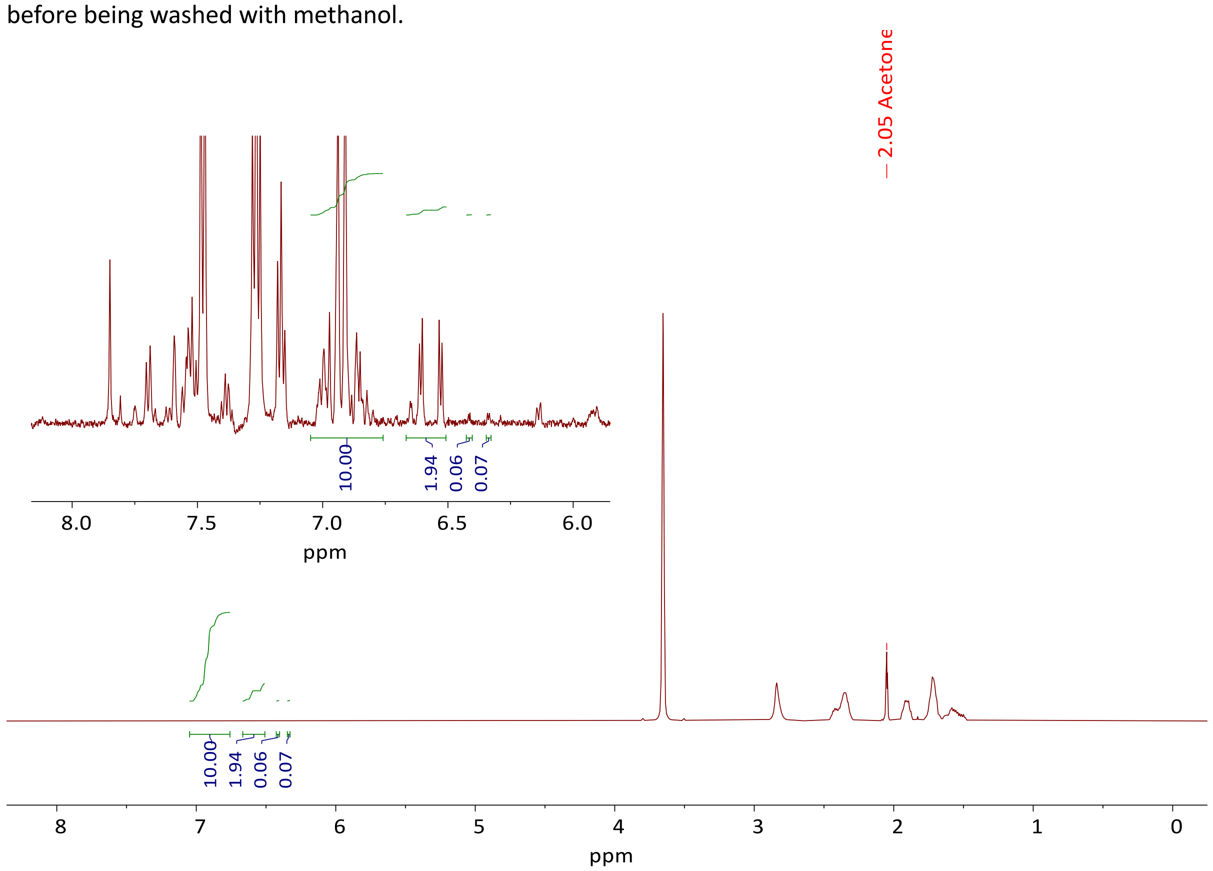

Spectrum S265. ^1H NMR (400 MHz, Acetone-d_6, 298 K) spectrum of post-solid-state-activation polymer **S30** after being washed with methanol.

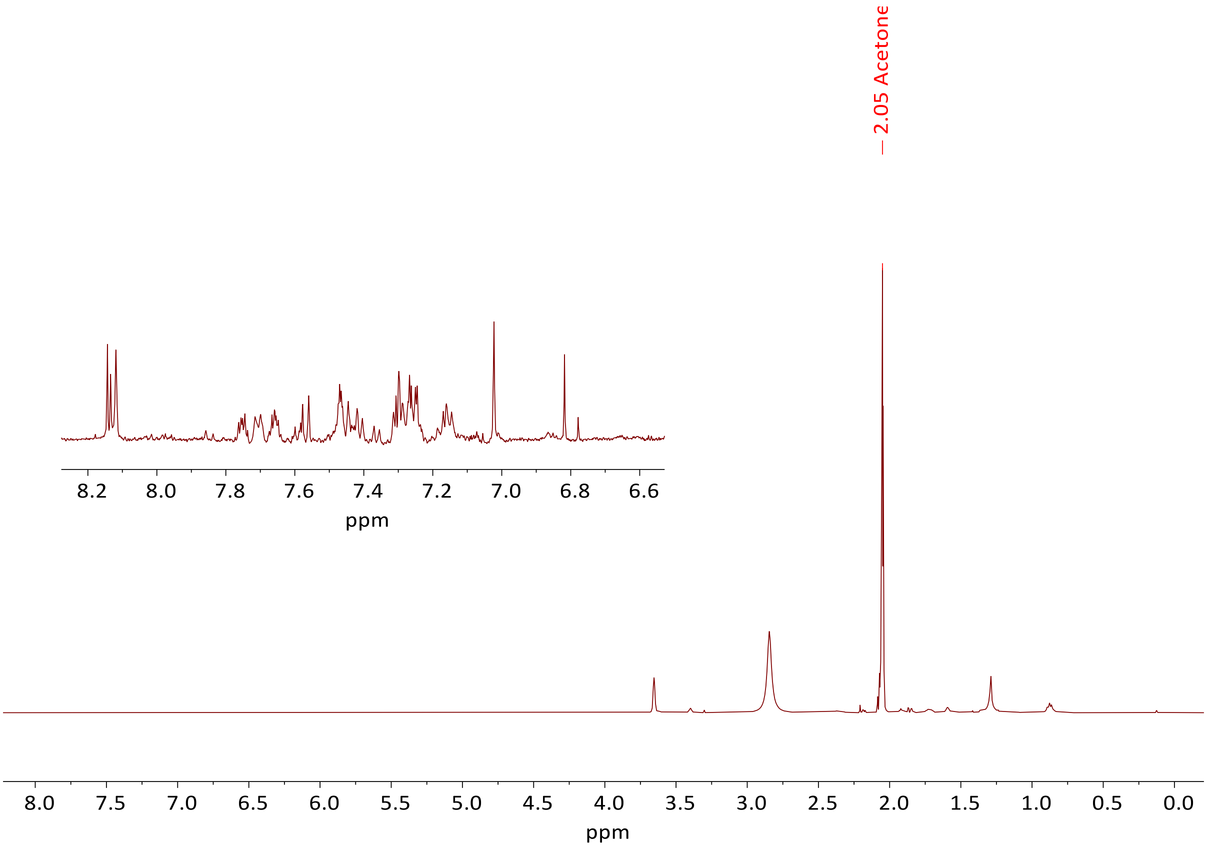

Spectrum S266. ^{1}H NMR (400 MHz, Acetone-d_6, 298 K) spectrum of the concentrated methanol washings from post-solid-state-activation polymer **S30**.

10 Mass Spectrometry Isotopic Patterns

10.1 Isotopic distribution of S15

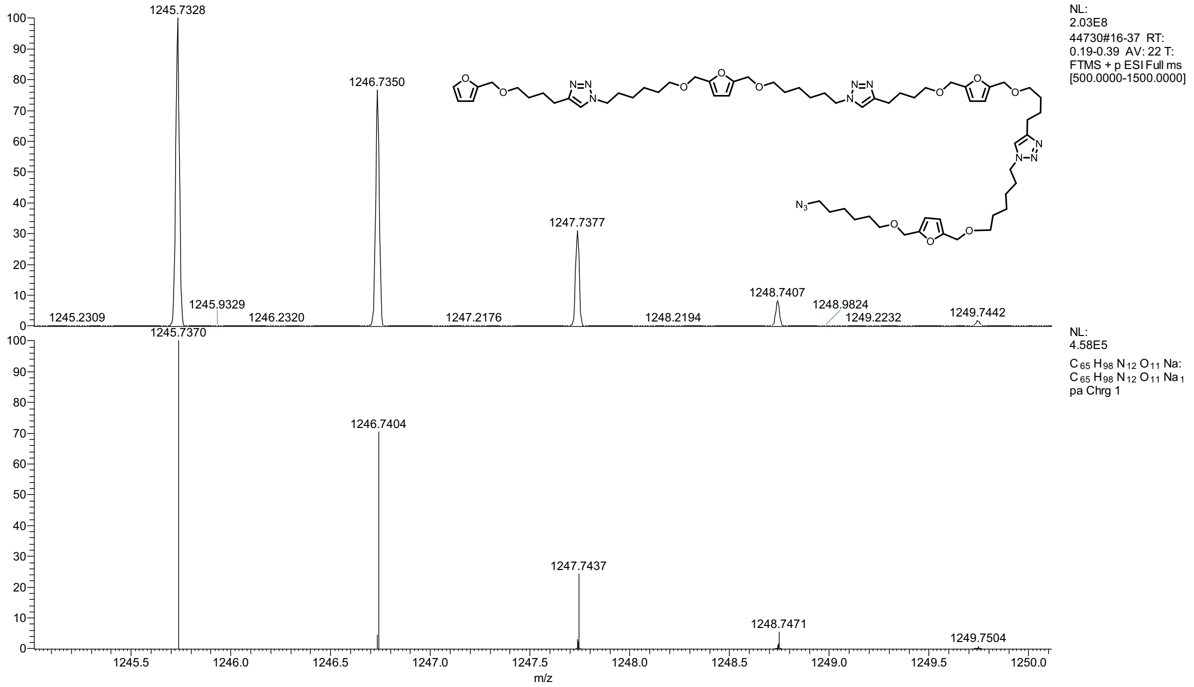

Spectrum S267. Isotopic distribution of **S15**. Top: Measured isotopic distribution for $C_{65}H_{98}N_{12}O_{11}Na$ ($[M+Na]^+$, +ESI). Bottom: Simulated isotopic distribution for $C_{65}H_{98}N_{12}O_{11}Na^+$.

10.2 Isotopic distribution of S16

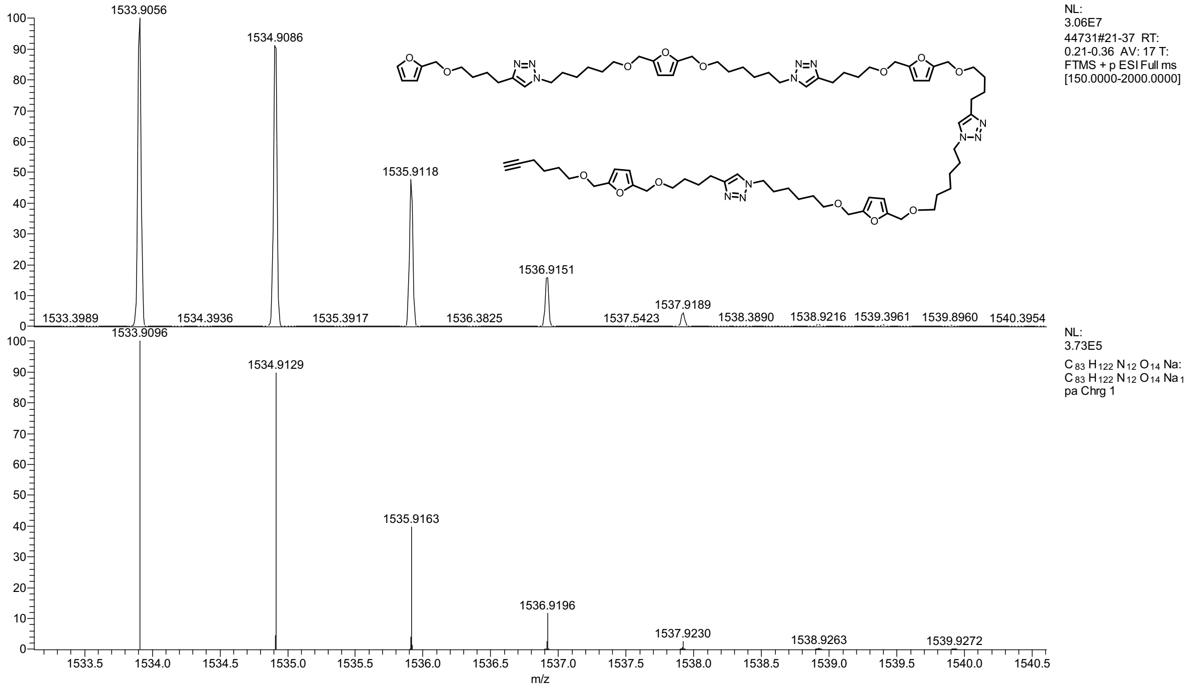

Spectrum S268. Isotopic distribution of **S16**. Top: Measured isotopic distribution for $C_{83}H_{122}N_{12}O_{14}Na$ ($[M+Na]^+$, +ESI). Bottom: Simulated isotopic distribution for $C_{83}H_{122}N_{12}O_{14}Na^+$.

10.3 Isotopic distribution of S17a

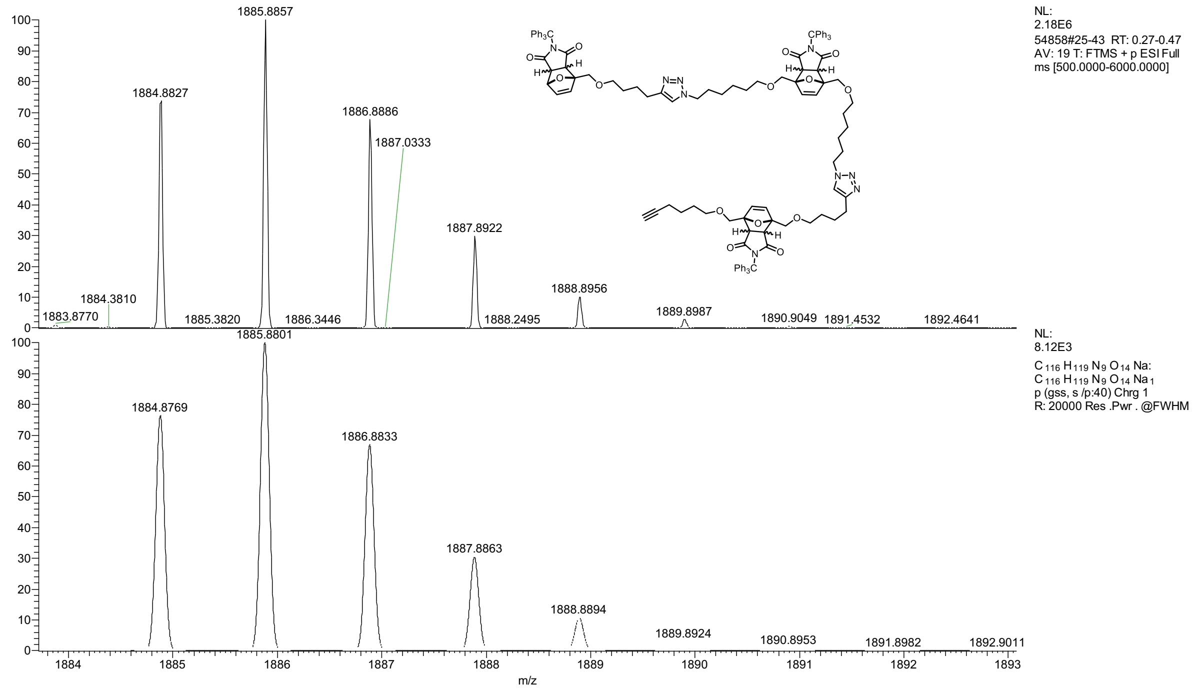

Spectrum S269. Isotopic distribution of **S17a**. Top: Measured isotopic distribution for $C_{116}H_{119}N_9O_{14}Na$ ($[M+Na]^+$, +ESI). Bottom: Simulated isotopic distribution for $C_{116}H_{119}N_9O_{14}Na^+$.

10.4 Isotopic distribution of S17b

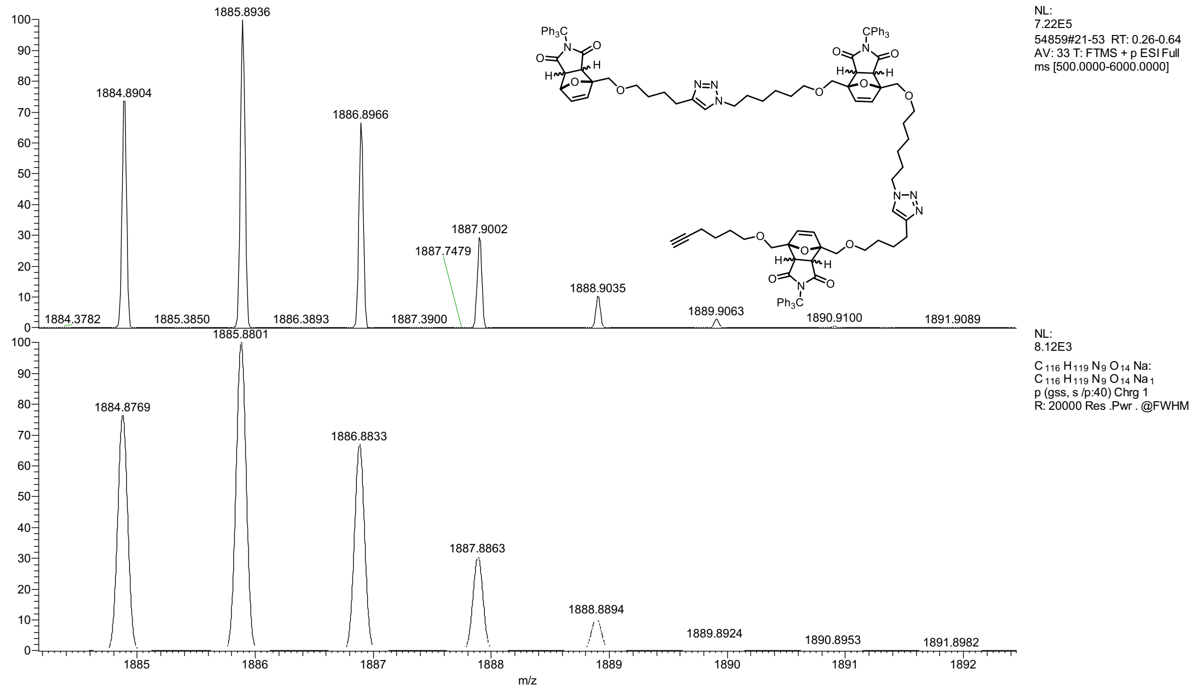

Spectrum S270. Isotopic distribution of **S17b**. Top: Measured isotopic distribution for $C_{116}H_{119}N_9O_{14}Na$ ($[M+Na]^+$, +ESI). Bottom: Simulated isotopic distribution for $C_{116}H_{119}N_9O_{14}Na^+$.

10.5 Isotopic distribution of S17c

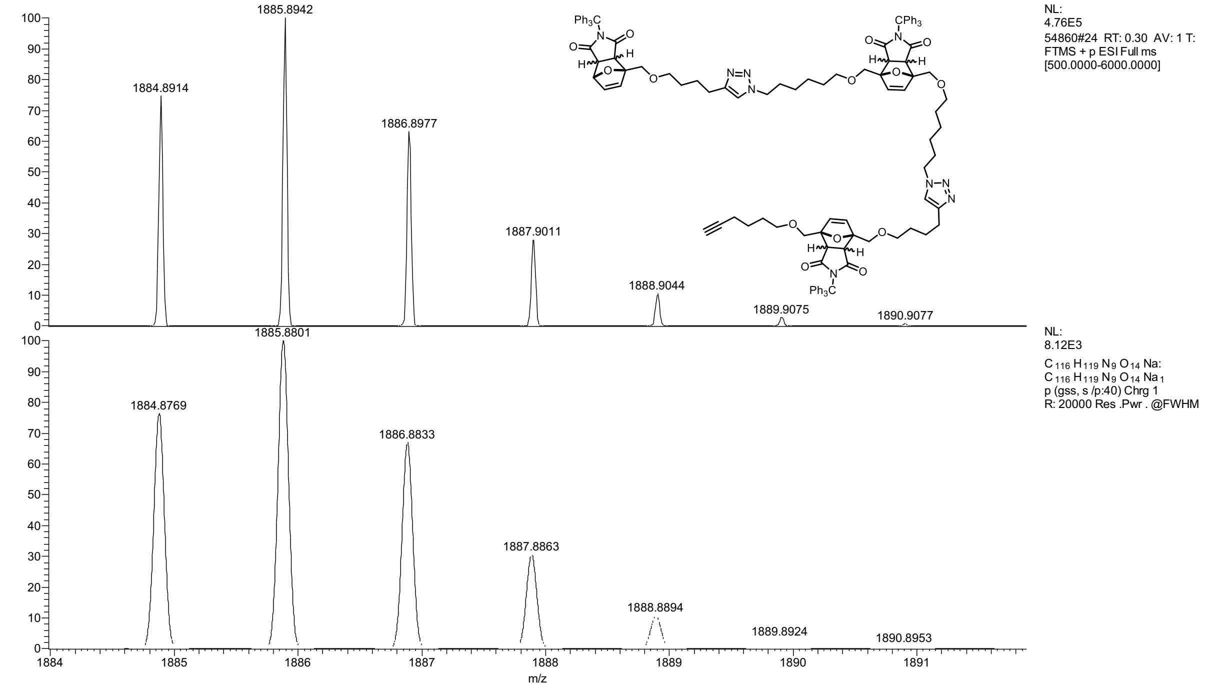

Spectrum S271. Isotopic distribution of **S17c**. Top: Measured isotopic distribution for $C_{116}H_{119}N_9O_{14}Na$ ($[M+Na]^+$, +ESI). Bottom: Simulated isotopic distribution for $C_{116}H_{119}N_9O_{14}Na^+$.

10.6 Isotopic distribution of S18a

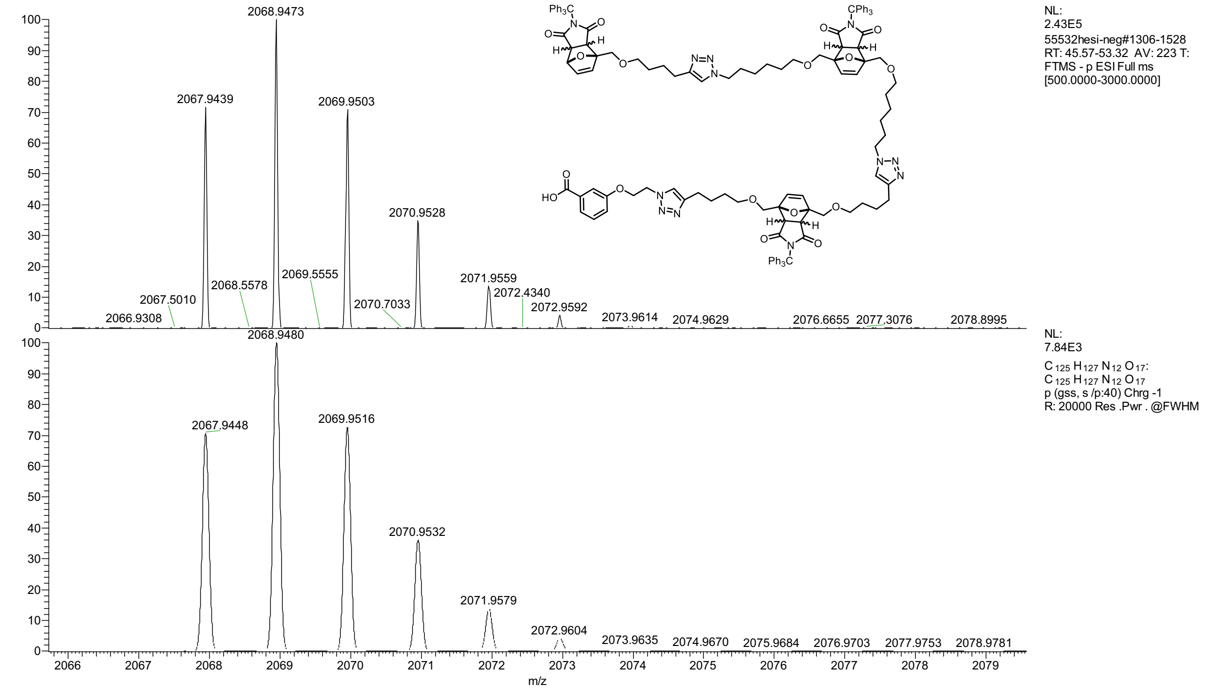

Spectrum S272. Isotopic distribution of **S18a**. Top: Measured isotopic distribution for $C_{125}H_{127}N_{12}O_{17}$ ($[M-H]^-$, -ESI). Bottom: Simulated isotopic distribution for $C_{125}H_{127}N_{12}O_{17}^-$.

10.7 Isotopic distribution of S18b

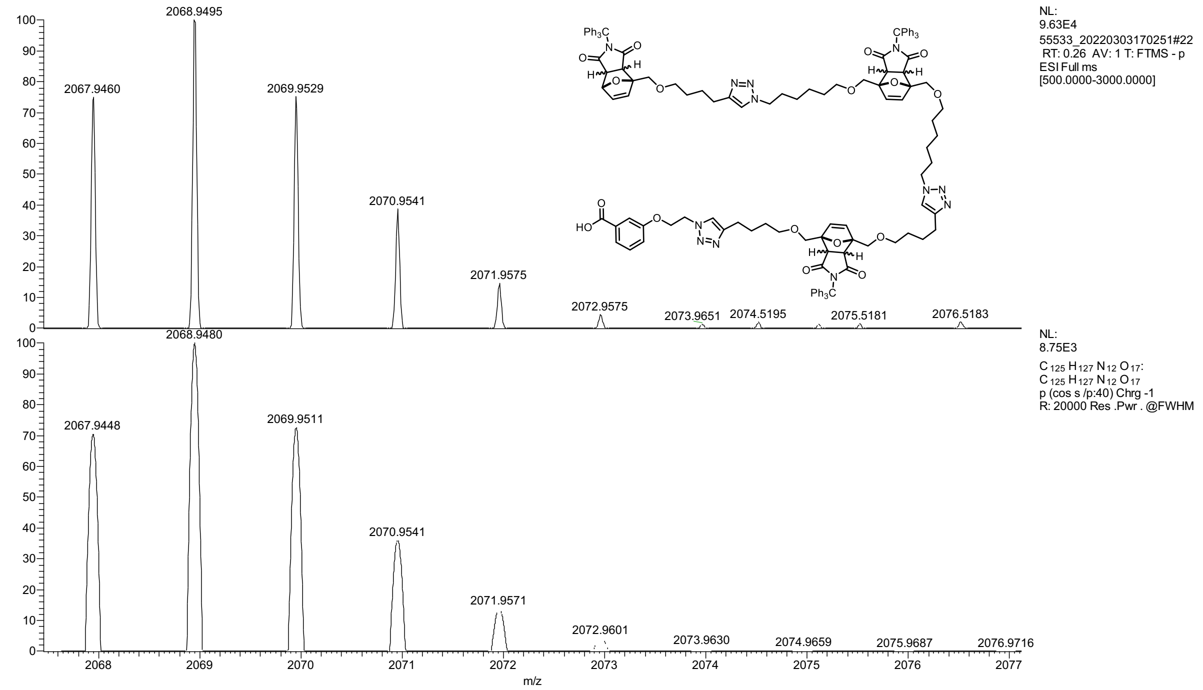

Spectrum S273. Isotopic distribution of **S18b**. Top: Measured isotopic distribution for $C_{125}H_{127}N_{12}O_{17}$ ([M-H]$^-$, -ESI). Bottom: Simulated isotopic distribution for $C_{125}H_{127}N_{12}O_{17}^-$.

10.8 Isotopic distribution of S18c

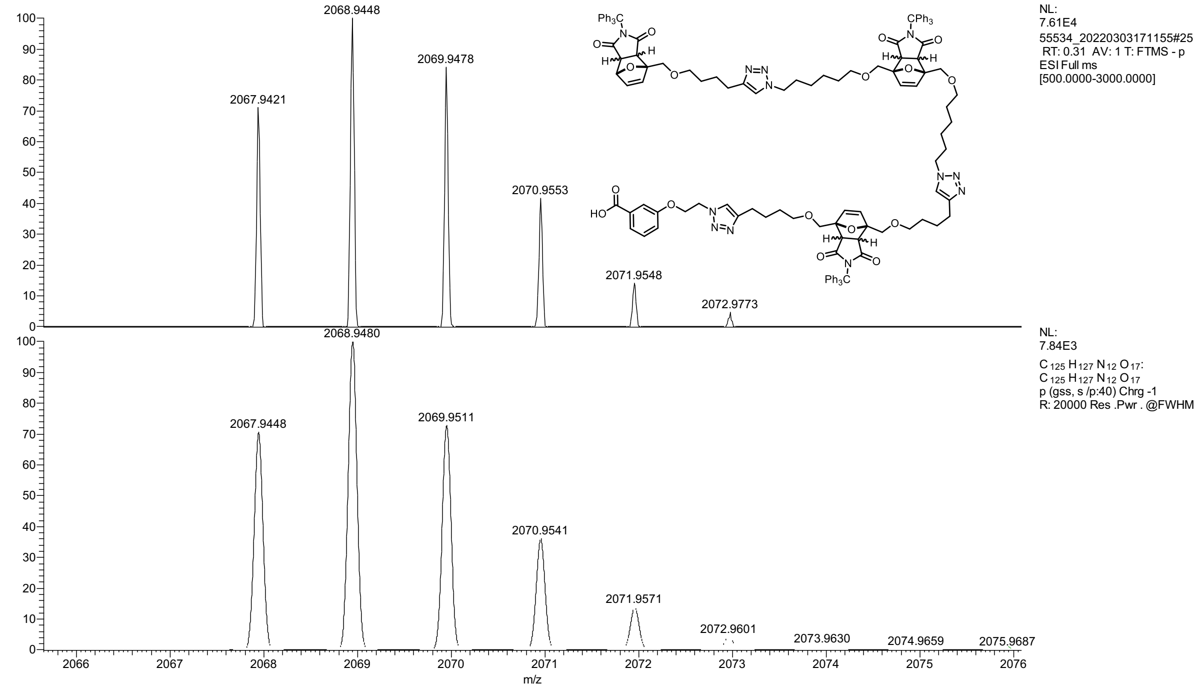

Spectrum S274. Isotopic distribution of **S18c**. Top: Measured isotopic distribution for $C_{125}H_{127}N_{12}O_{17}$ ([M-H]$^-$, -ESI). Bottom: Simulated isotopic distribution for $C_{125}H_{127}N_{12}O_{17}^-$.

10.9 Isotopic distribution of S18d

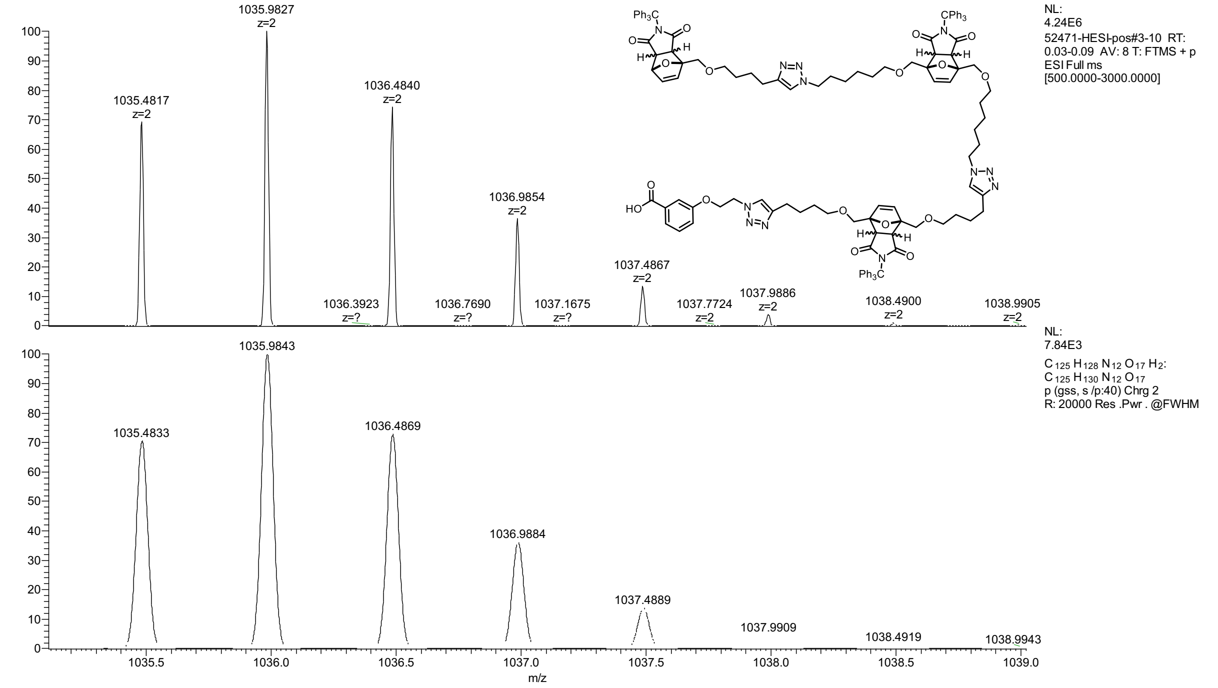

Spectrum S275. Isotopic distribution of **S18d**. Top: Measured isotopic distribution for $C_{125}H_{128}N_{12}O_{17}H_2$ ($[M+2H]^{2+}$, +ESI). Bottom: Simulated isotopic distribution for $C_{125}H_{128}N_{12}O_{17}H_2^{2+}$.

10.10 Isotopic distribution of S19

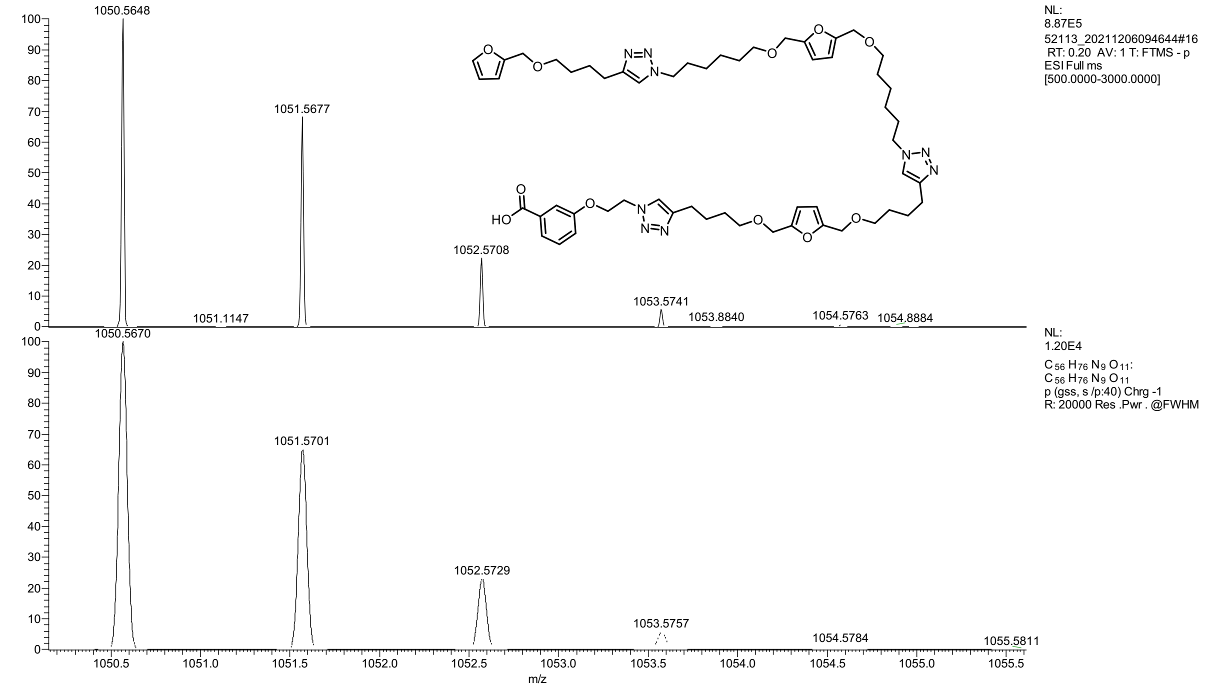

Spectrum S276. Isotopic distribution of **S19**. Top: Measured isotopic distribution for $C_{56}H_{76}N_9O_{11}^-$ ($[M-H]^-$, -ESI). Bottom: Simulated isotopic distribution for $C_{56}H_{76}N_9O_{11}^-$.

10.11 Isotopic distribution of S20

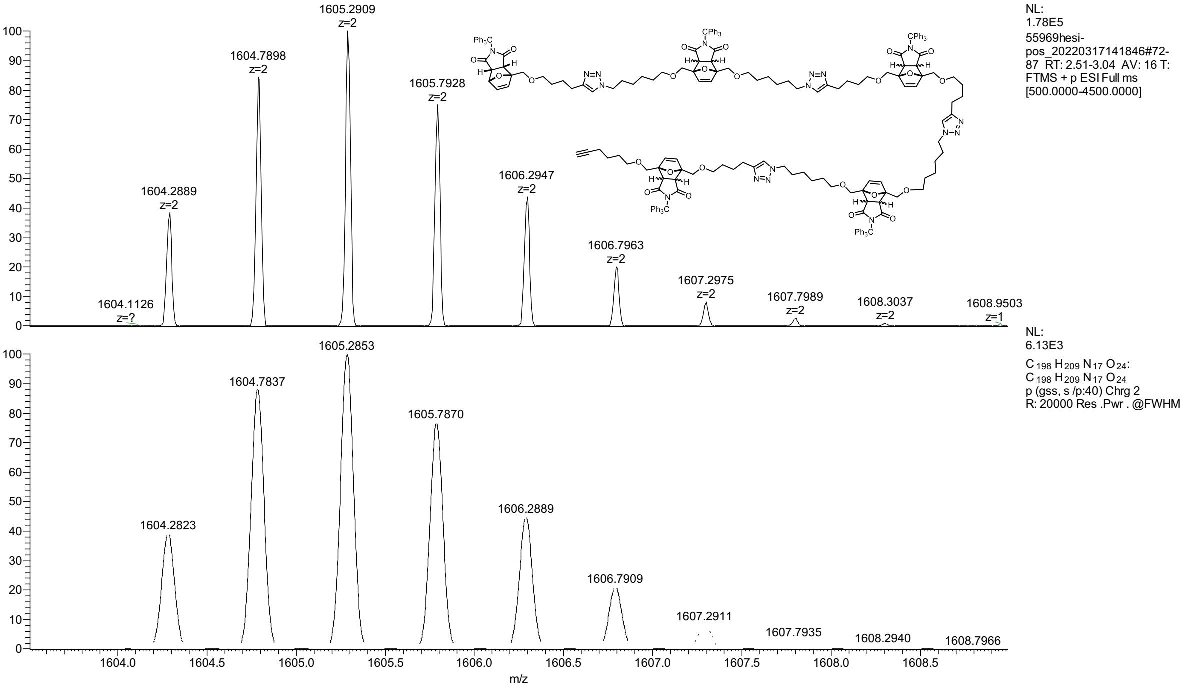

Spectrum S277. Isotopic distribution of **S20**. Top: Measured isotopic distribution for $C_{198}H_{207}N_{17}O_{24}H_2$ ($[M+2H]^{2+}$, +ESI). Bottom: Simulated isotopic distribution for $C_{198}H_{207}N_{17}O_{24}H_2{}^{2+}$.

10.12 Isotopic distribution of S21

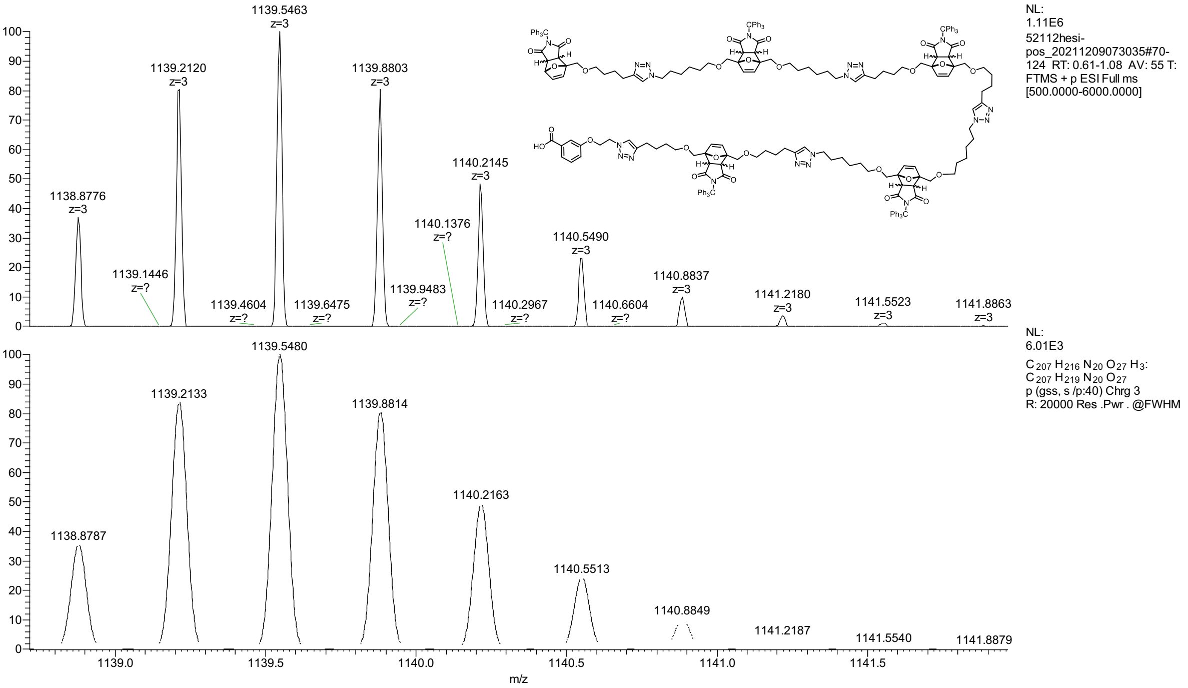

Spectrum S278. Isotopic distribution of **S21**. Top: Measured isotopic distribution for $C_{207}H_{216}N_{20}O_{27}H_3$ ($[M+3H]^{3+}$, +ESI). Bottom: Simulated isotopic distribution for $C_{207}H_{216}N_{20}O_{27}H_3{}^{3+}$.

10.13 Isotopic distribution of S22

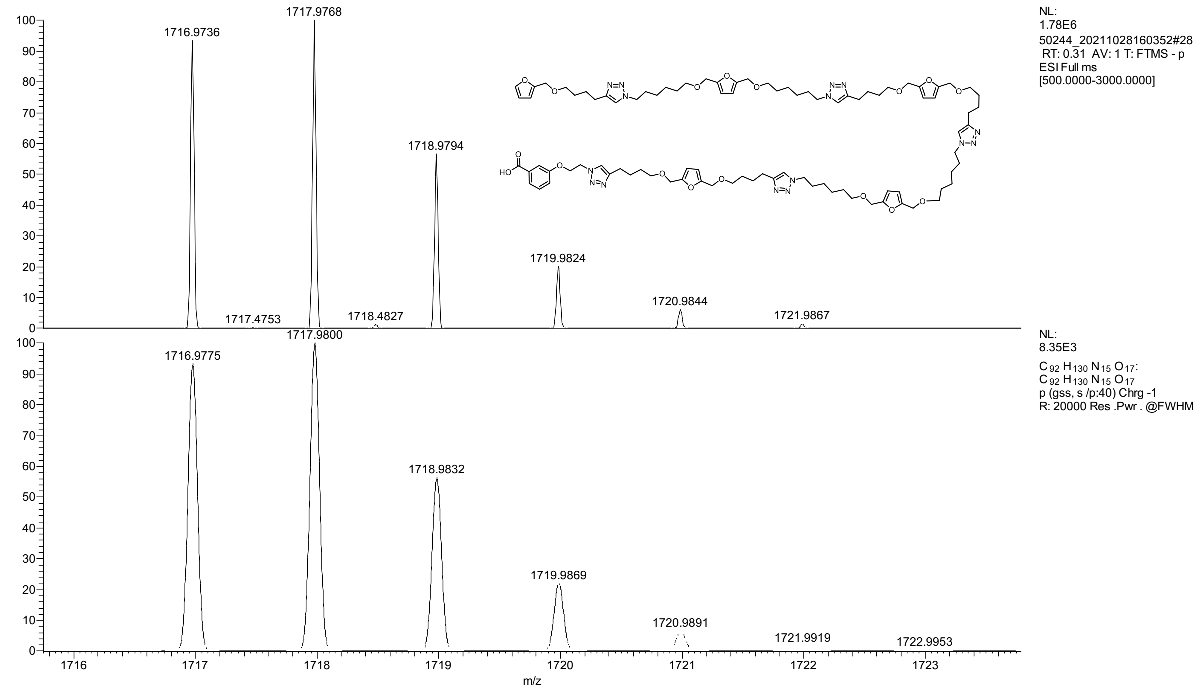

Spectrum S279. Isotopic distribution of **S22**. Top: Measured isotopic distribution for $C_{92}H_{130}N_{15}O_{17}$ ([M-H]$^-$, -ESI). Bottom: Simulated isotopic distribution for $C_{92}H_{130}N_{15}O_{17}^-$.

10.14 Isotopic distribution of 5

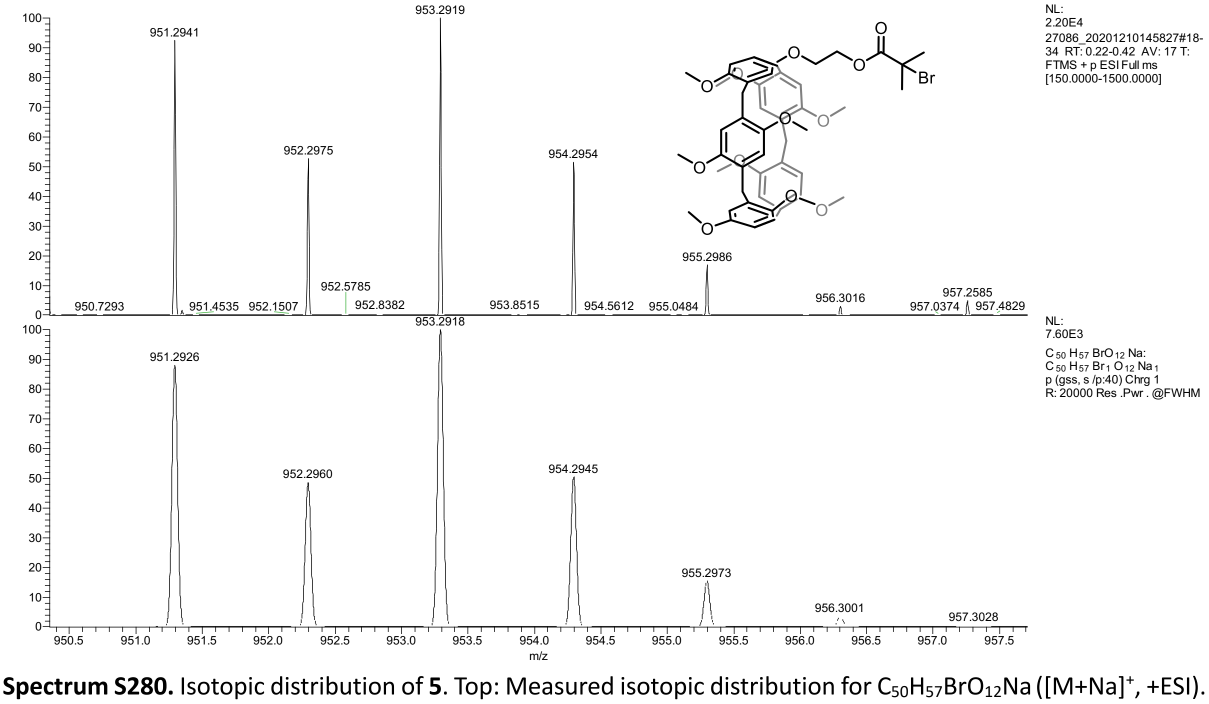

Spectrum S280. Isotopic distribution of **5**. Top: Measured isotopic distribution for $C_{50}H_{57}BrO_{12}Na$ ([M+Na]$^+$, +ESI). Bottom: Simulated isotopic distribution for $C_{50}H_{57}BrO_{12}Na^+$.

10.15 Isotopic distribution of $\mathbf{7}_{\textbf{\textit{trans}}}$

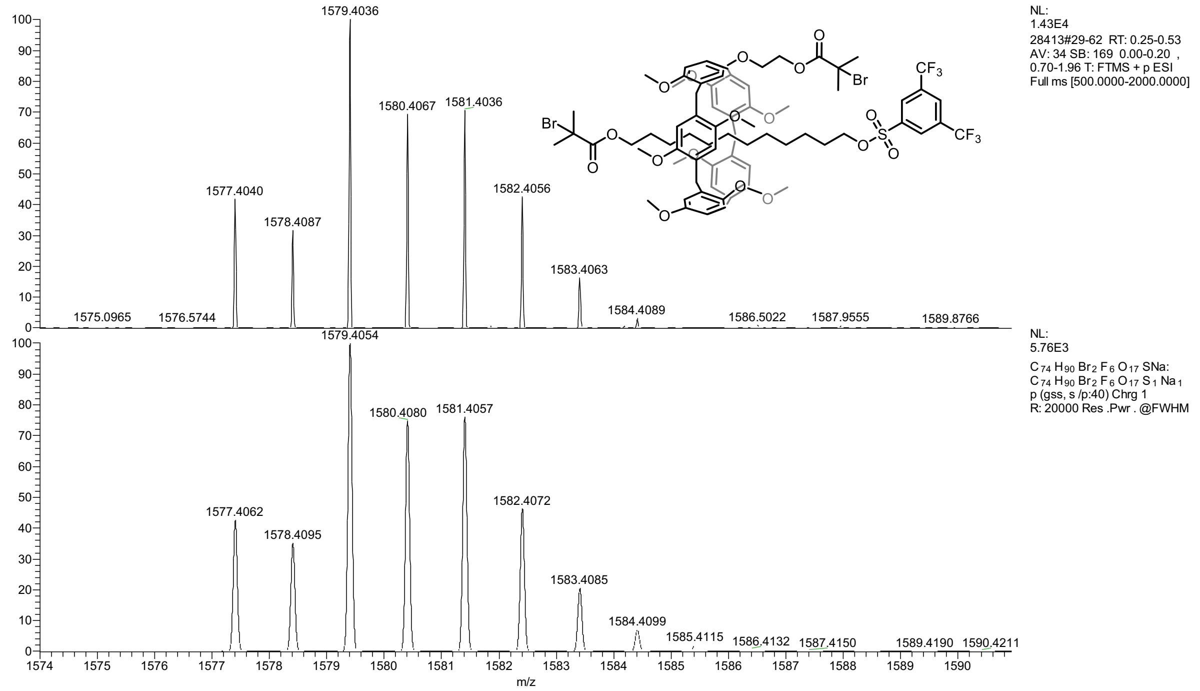

Spectrum S281. Isotopic distribution of $\mathbf{7}_{\textbf{\textit{trans}}}$. Top: Measured isotopic distribution for for $C_{74}H_{90}Br_2F_6O_{17}SNa$ ($[M+Na]^+$, +ESI). Bottom: Simulated isotopic distribution for for $C_{74}H_{90}Br_2F_6O_{17}SNa^+$.

10.16 Isotopic distribution of $\mathbf{7}_{\textbf{\textit{cis}}}$

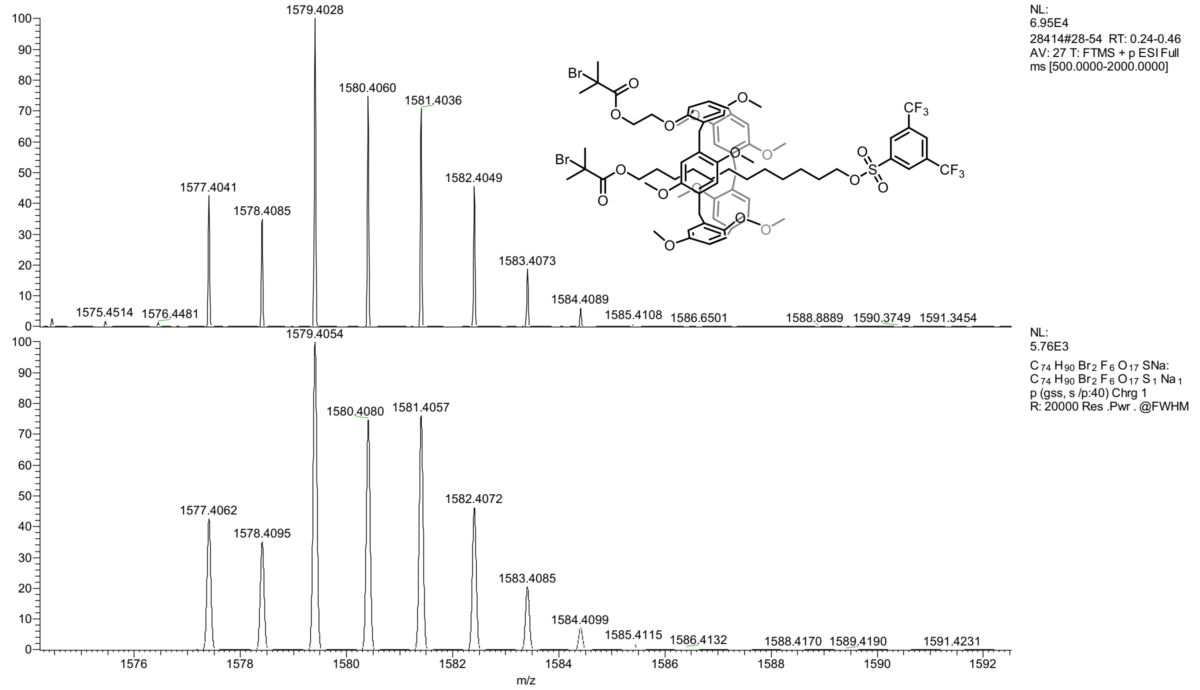

Spectrum S282. Isotopic distribution of $\mathbf{7}_{\textbf{\textit{cis}}}$. Top: Measured isotopic distribution for for $C_{74}H_{90}Br_2F_6O_{17}SNa$ ($[M+Na]^+$, +ESI). Bottom: Simulated isotopic distribution for for $C_{74}H_{90}Br_2F_6O_{17}SNa^+$.

10.17 Isotopic distribution of S25$_{trans}$

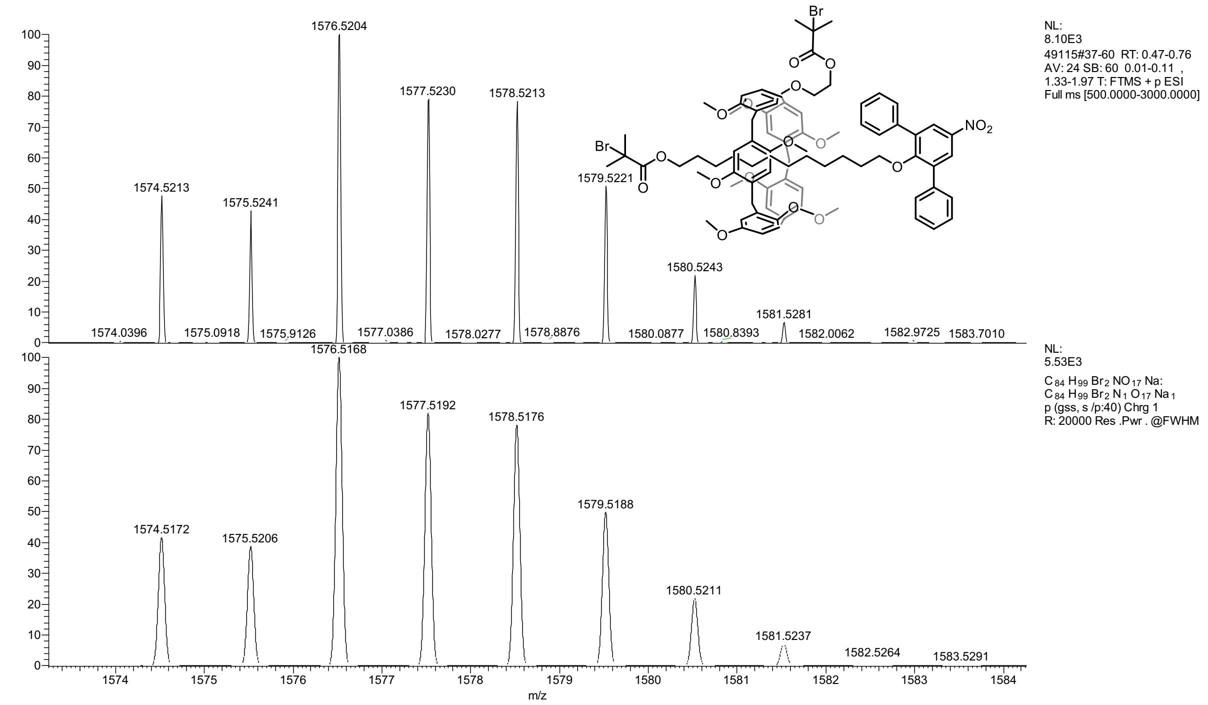

Spectrum S283. Isotopic distribution of **S25$_{trans}$**. Top: Measured isotopic distribution for $C_{84}H_{99}Br_2NO_{17}Na$ ($[M+Na]^+$, +ESI). Bottom: Simulated isotopic distribution for $C_{84}H_{99}Br_2NO_{17}Na^+$.

10.18 Isotopic distribution of S25$_{cis}$

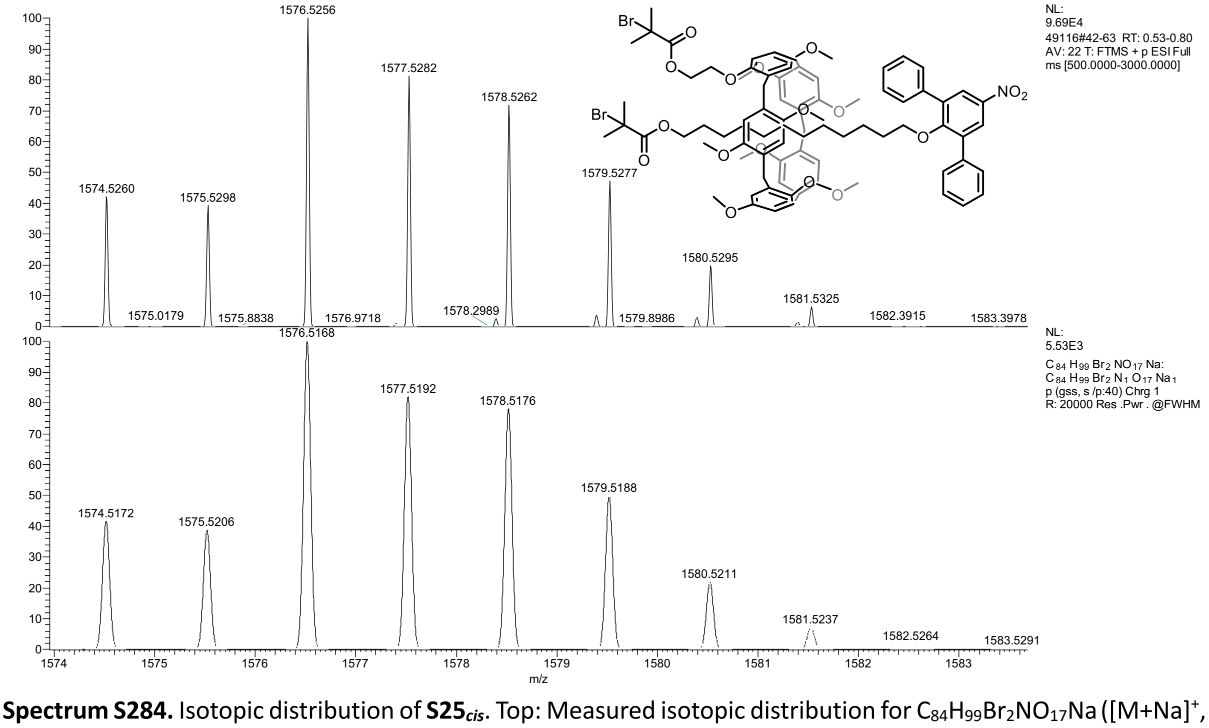

Spectrum S284. Isotopic distribution of **S25$_{cis}$**. Top: Measured isotopic distribution for $C_{84}H_{99}Br_2NO_{17}Na$ ($[M+Na]^+$, +ESI). Bottom: Simulated isotopic distribution for $C_{84}H_{99}Br_2NO_{17}Na^+$.

10.19 Isotopic distribution of S26$_{exo}$

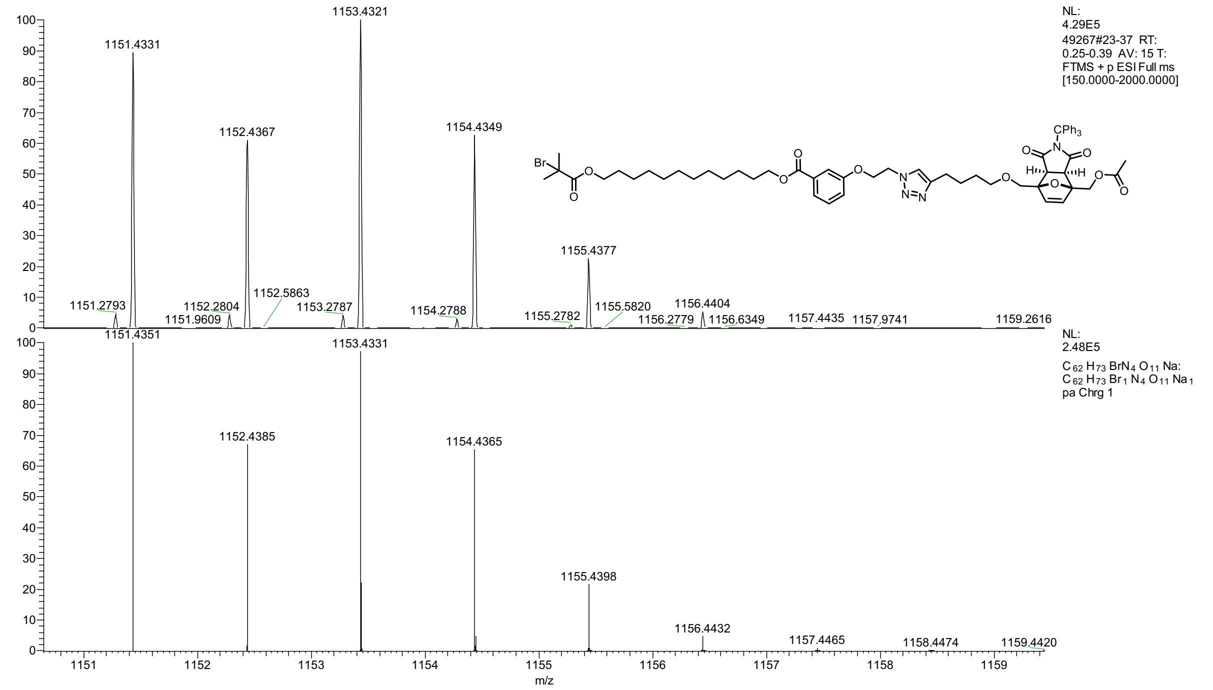

Spectrum S285. Isotopic distribution of **S26$_{exo}$**. Top: Measured isotopic distribution for $C_{62}H_{73}BrN_4O_{11}Na$ ($[M+Na]^+$, +ESI). Bottom: Simulated isotopic distribution for $C_{62}H_{73}BrN_4O_{11}Na^+$.

10.20 Isotopic distribution of S26$_{endo}$

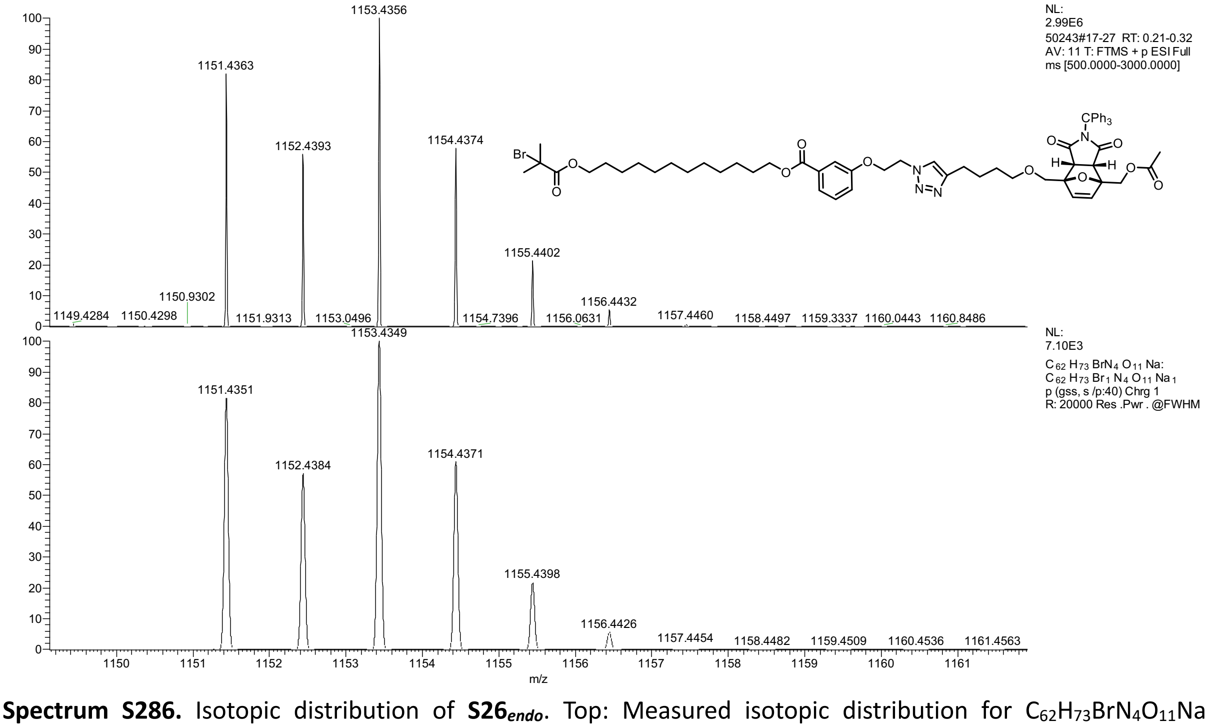

Spectrum S286. Isotopic distribution of **S26$_{endo}$**. Top: Measured isotopic distribution for $C_{62}H_{73}BrN_4O_{11}Na$ ($[M+Na]^+$, +ESI). Bottom: Simulated isotopic distribution for $C_{62}H_{73}BrN_4O_{11}Na^+$.

10.21 Isotopic distribution of S27$_{trans/exo}$

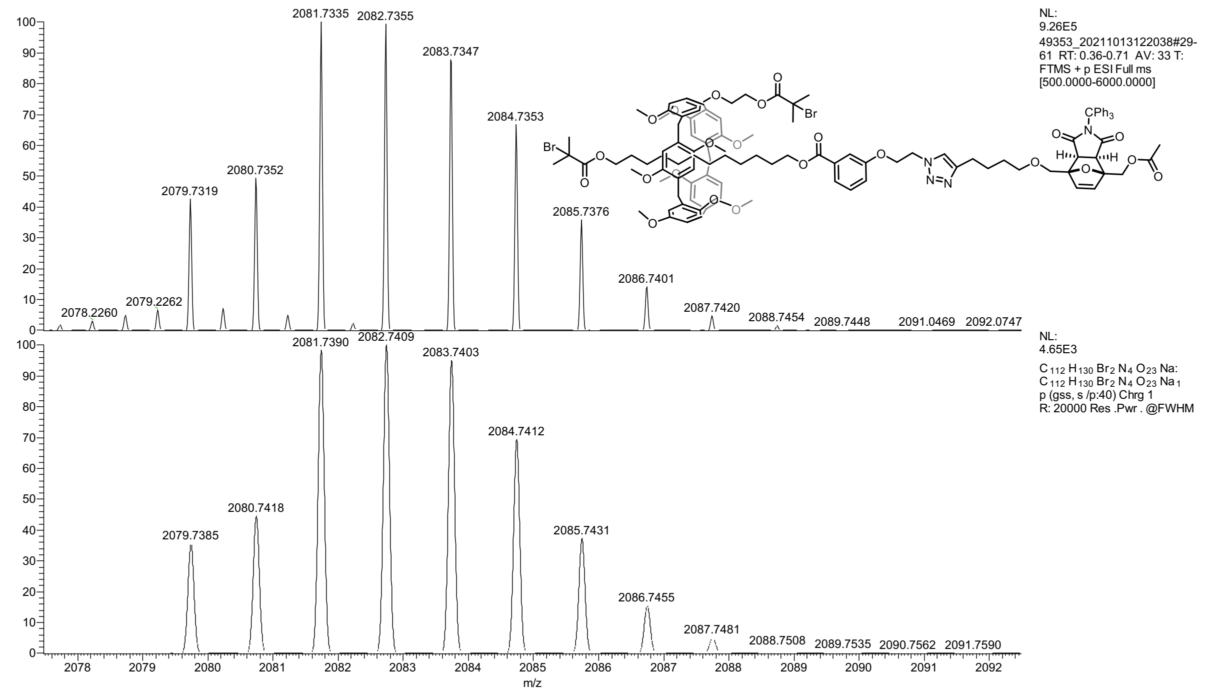

Spectrum S287. Isotopic distribution of **S27*trans/exo***. Top: Measured isotopic distribution for $C_{112}H_{130}Br_2N_4O_{23}Na$ ([M+Na]$^+$, +ESI). Bottom: Simulated isotopic distribution for $C_{112}H_{130}Br_2N_4O_{23}Na^+$.

10.22 Isotopic distribution of S27$_{cis/exo}$

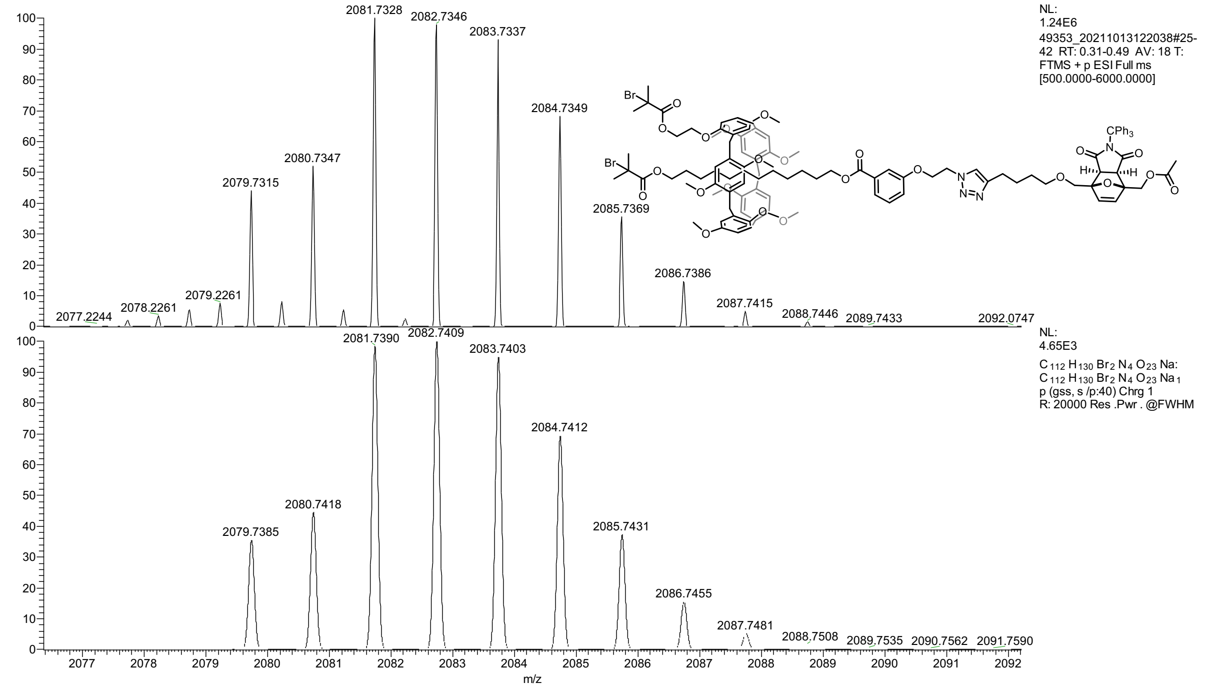

Spectrum S288. Isotopic distribution of **S27*cis/exo***. Top: Measured isotopic distribution for $C_{112}H_{130}Br_2N_4O_{23}Na$ ([M+Na]$^+$, +ESI). Bottom: Simulated isotopic distribution for $C_{112}H_{130}Br_2N_4O_{23}Na^+$.

10.23 Isotopic distribution of S27$_{trans/endo}$

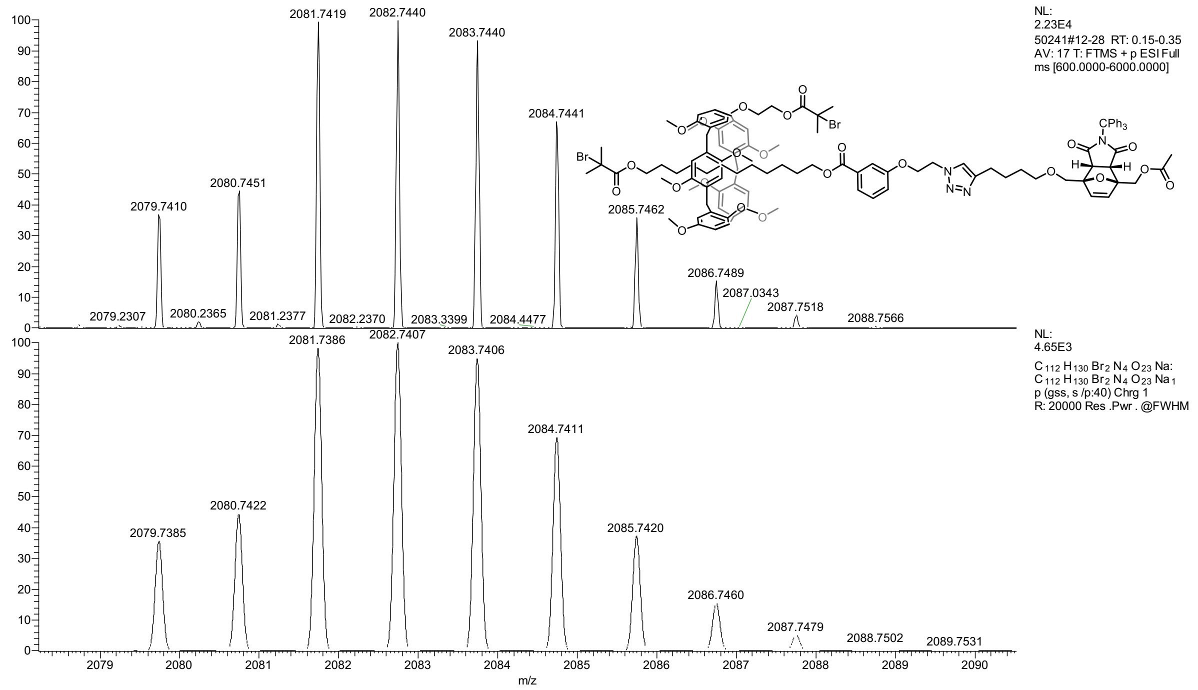

Spectrum S289. Isotopic distribution of **S27$_{trans/endo}$**. Top: Measured isotopic distribution for $C_{112}H_{130}Br_2N_4O_{23}Na$ ($[M+Na]^+$, +ESI). Bottom: Simulated isotopic distribution for $C_{112}H_{130}Br_2N_4O_{23}Na^+$.

10.24 Isotopic distribution of S27$_{cis/endo}$

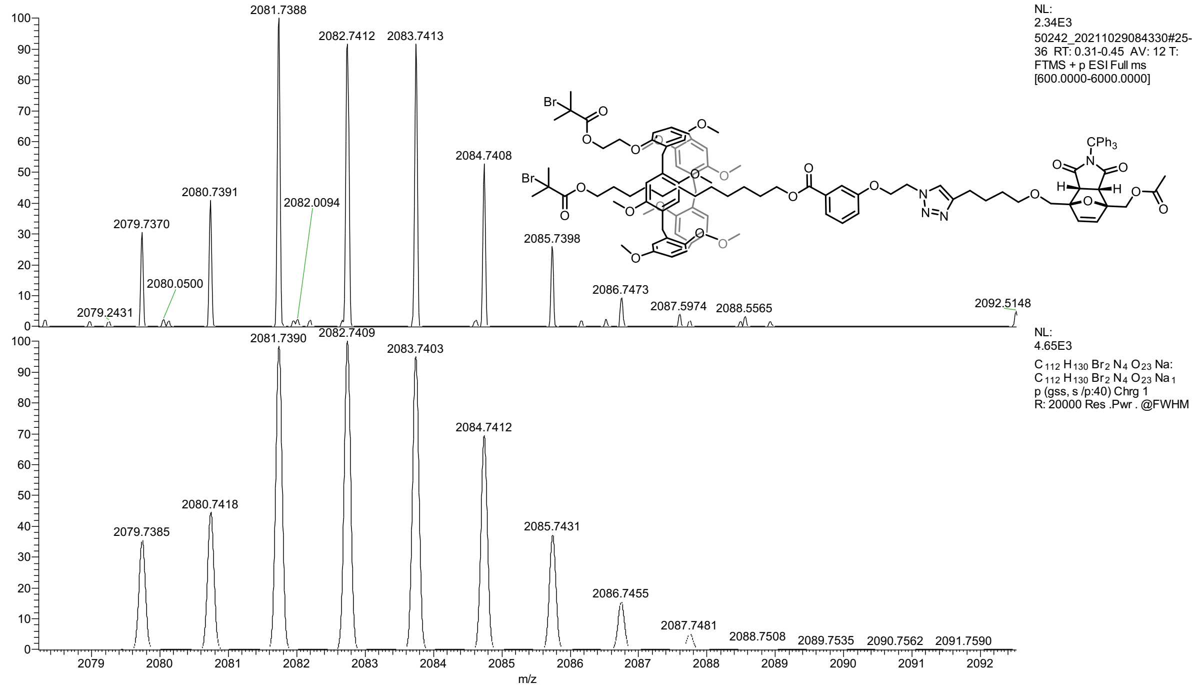

Spectrum S290. Isotopic distribution of **26**. Top: Measured isotopic distribution for $C_{112}H_{130}Br_2N_4O_{23}Na$ ($[M+Na]^+$, +ESI). Bottom: Simulated isotopic distribution for $C_{112}H_{130}Br_2N_4O_{23}Na^+$.

10.25 Isotopic distribution of 8_3a

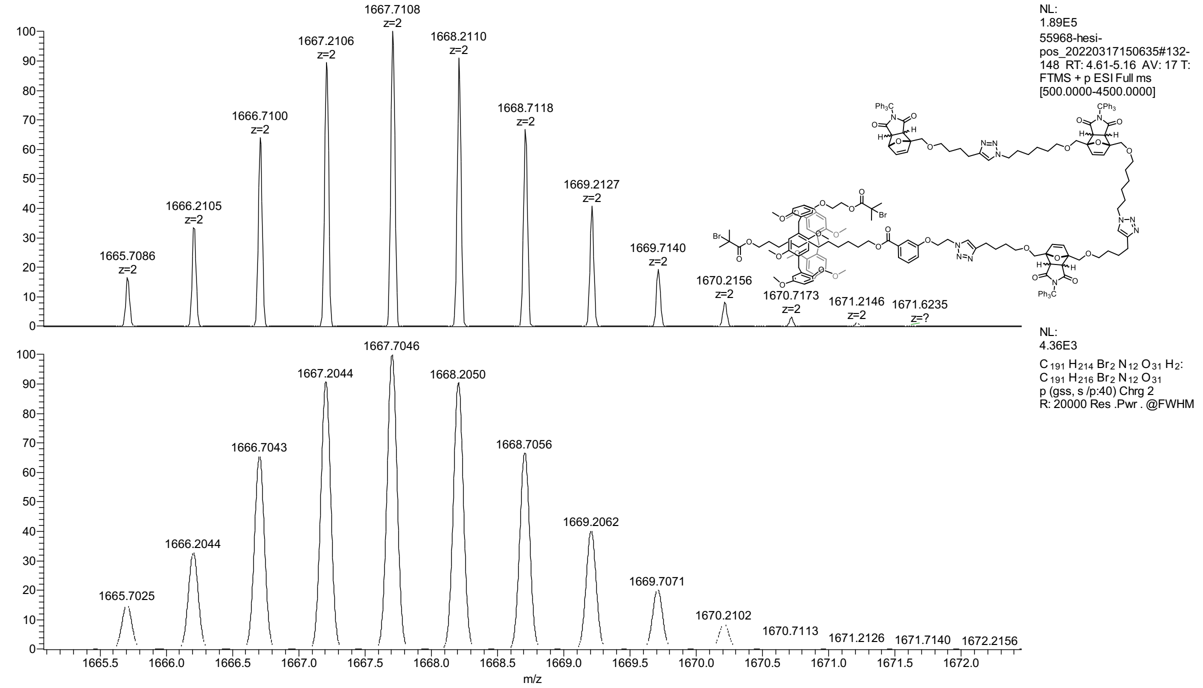

Spectrum S291. Isotopic distribution of **8_3a**. Top: Measured isotopic distribution for $C_{191}H_{214}Br_2N_{12}O_{31}H_2$ ([M+2H]$^{2+}$, +ESI). Bottom: Simulated isotopic distribution for $C_{191}H_{214}Br_2N_{12}O_{31}H_2^{2+}$.

10.26 Isotopic distribution of 8_3b

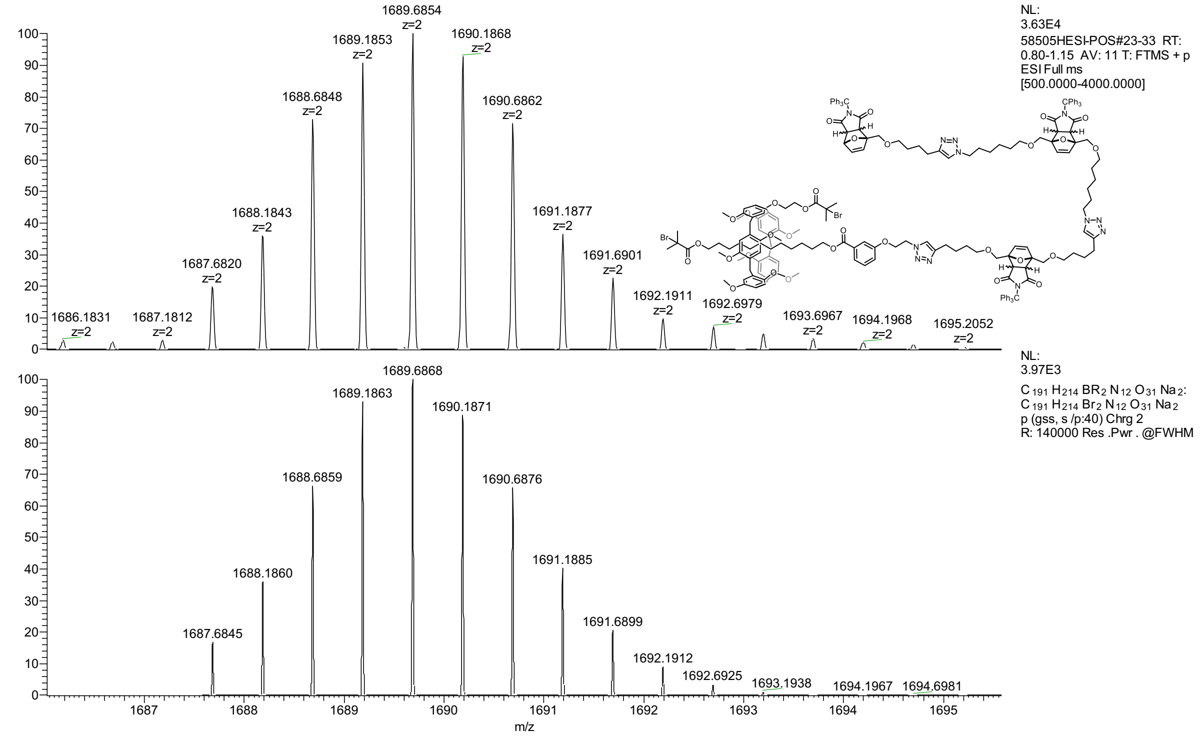

Spectrum S292. Isotopic distribution of **8_3b**. Top: Measured isotopic distribution for $C_{191}H_{214}Br_2N_{12}O_{31}Na_2$ ([M+2Na]$^{2+}$, +ESI). Bottom: Simulated isotopic distribution for $C_{191}H_{214}Br_2N_{12}O_{31}Na_2^{2+}$.

10.27 Isotopic distribution of 8_3c

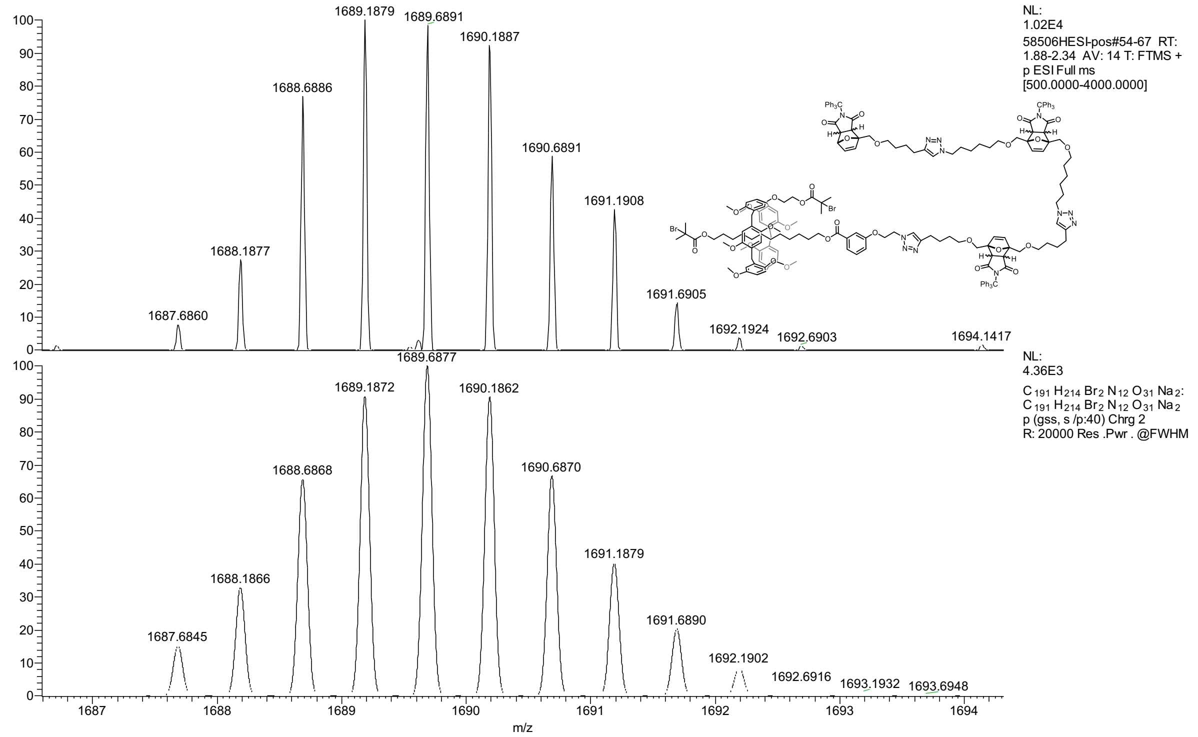

Spectrum S293. Isotopic distribution of **8_3c**. Top: Measured isotopic distribution for $C_{191}H_{214}Br_2N_{12}O_{31}Na_2$ ([M+2Na]$^{2+}$, +ESI). Bottom: Simulated isotopic distribution for $C_{191}H_{214}Br_2N_{12}O_{31}Na_2^{2+}$.

10.28 Isotopic distribution of 8_3d

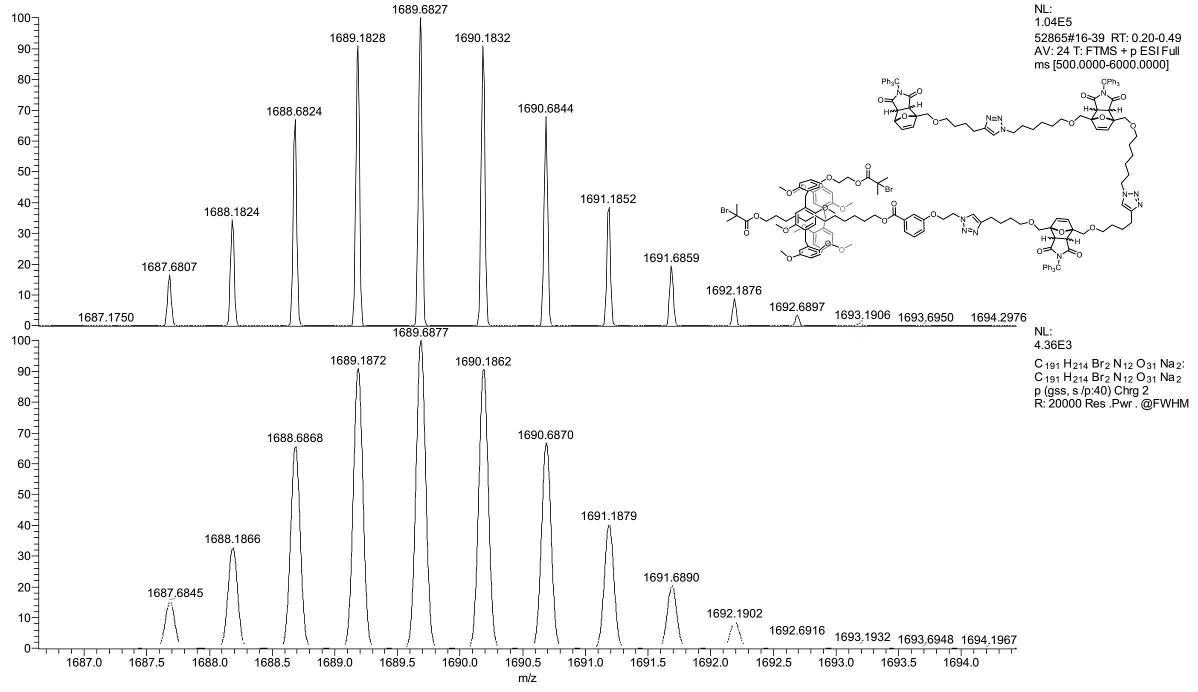

Spectrum S294. Isotopic distribution of **8_3d**. Top: Measured isotopic distribution for $C_{191}H_{214}Br_2N_{12}O_{31}Na_2$ ([M+2Na]$^{2+}$, +ESI). Bottom: Simulated isotopic distribution for $C_{191}H_{214}Br_2N_{12}O_{31}Na_2^{2+}$.

10.29 Isotopic distribution of 8_5

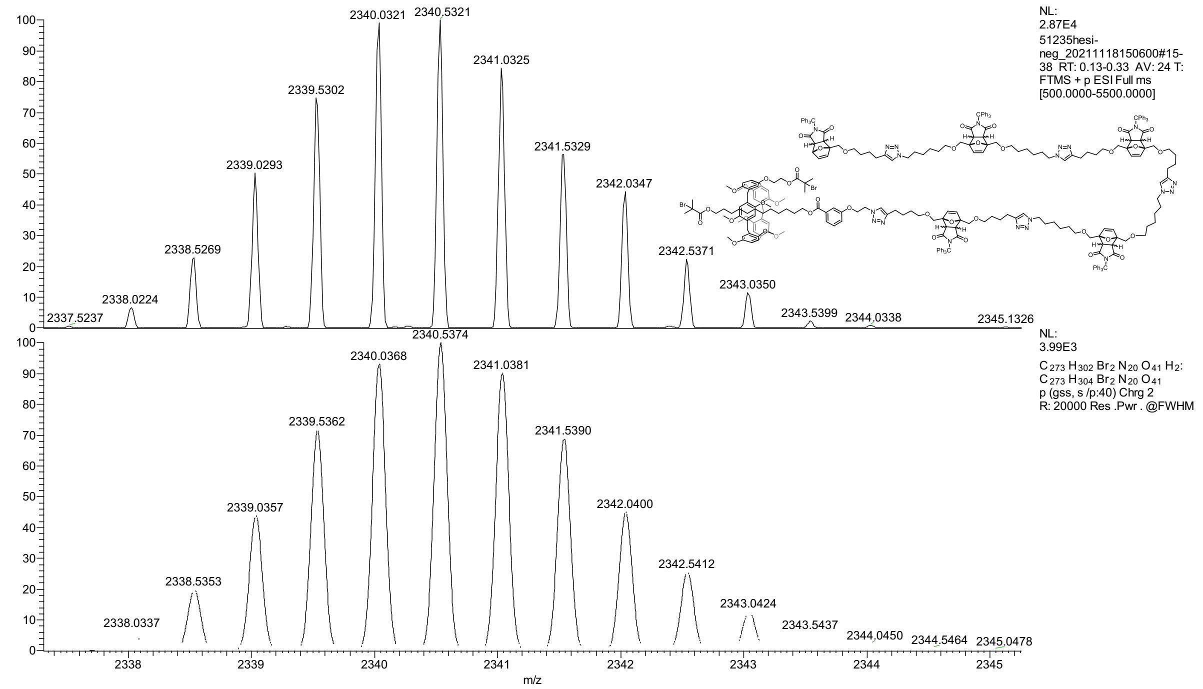

Spectrum S295. Isotopic distribution of **8_5**. Top: Measured isotopic distribution for $C_{273}H_{302}Br_2N_{20}O_{41}H_2$ ($[M+2H]^{2+}$, +ESI). Bottom: Simulated isotopic distribution for $C_{273}H_{302}Br_2N_{20}O_{41}H_2^{2+}$.

10.30 Isotopic Distribution of S33

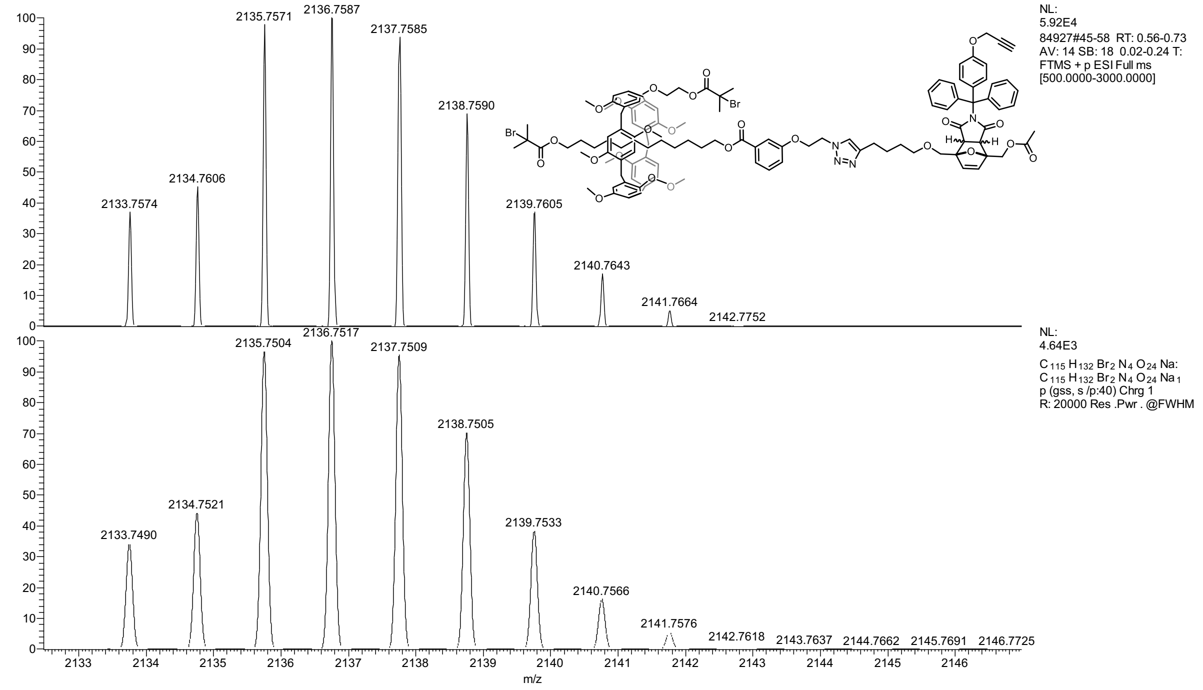

Spectrum S296. Isotopic distribution of **S33**. Top: Measured isotopic distribution for $C_{115}H_{132}Br_2N_4O_{24}Na$ ($[M+Na]^+$, +ESI). Bottom: Simulated isotopic distribution for $C_{115}H_{132}Br_2N_4O_{24}Na^+$.

10.31 Isotopic Distribution of 12

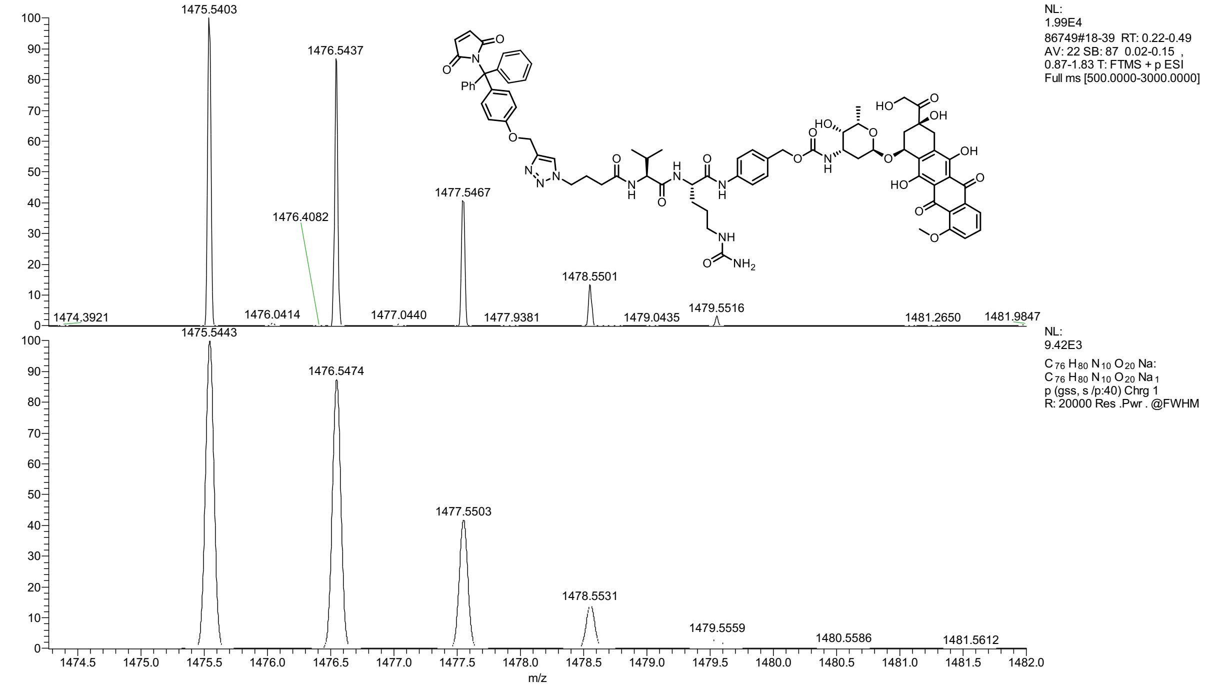

Spectrum S297. Isotopic distribution of **12**. Top: Measured distribution for $C_{76}H_{80}N_{10}O_{20}Na$ ([M+Na]+, +ESI). Bottom: Simulated isotopic distribution for $C_{76}H_{80}N_{10}O_{20}Na^{+}$.

10.32 Isotopic Distribution of S41

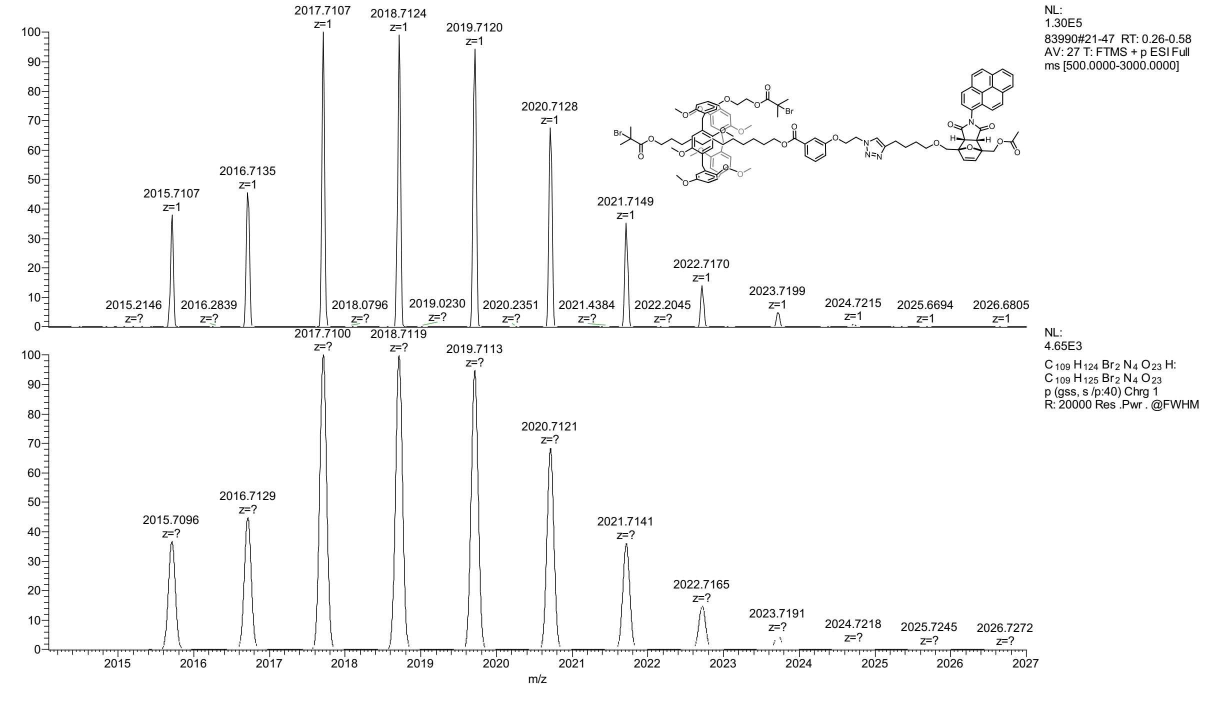

Spectrum S298. Isotopic distribution of **S41**. Top: Measured isotopic distribution for $C_{109}H_{124}Br_2N_4O_{23}H$ ([M+H]$^{+}$, +ESI). Bottom: Simulated isotopic distribution for $C_{109}H_{124}Br_2N_4O_{23}H^{+}$.

10.33 Isotopic Distribution of S42

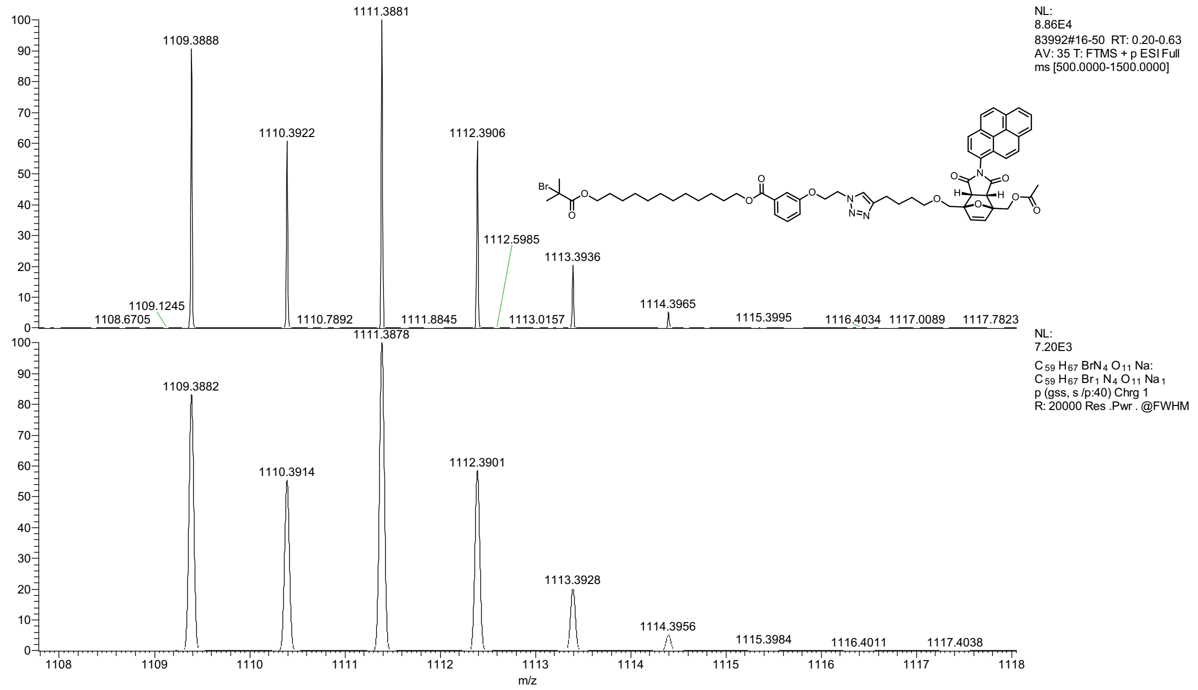

Spectrum S299. Isotopic distribution of **S42**. Top: Measured isotopic distribution for $C_{59}H_{67}BrN_4O_{11}Na$ ($[M+Na]^+$, +ESI). Bottom: Simulated isotopic distribution for $C_{59}H_{67}BrN_4O_{11}Na^+$.

10.34 Isotopic Distribution of S49

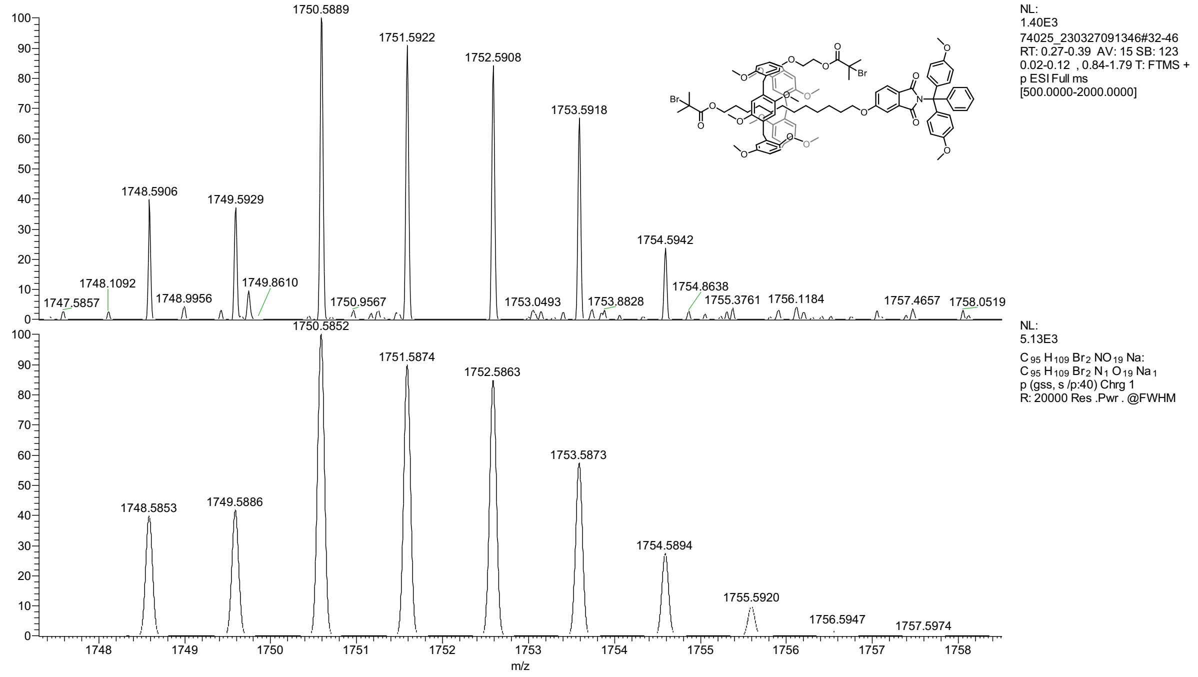

Spectrum S300. Isotopic distribution of **S49**. Top: Measured isotopic distribution for $C_{95}H109Br_2NO_{19}Na$ ($[M+Na]^+$, +ESI). Bottom: Simulated isotopic distribution for $C_{95}H109Br_2NO_{19}Na^+$.

11 Rotaxane Modelling

11.1 General Procedure

The structures of were built in Spartan ’20 and minimized using molecular mechanics (MMFF). CoGEF calculations were performed on Spartan ’20 following Beyer’s method.[6] The distance between the anchor atoms was constrained and increased in increments (2 Å at the start, 1 Å in the middle, and 0.1 Å close to *Emax*). At each step, the energy was minimized by molecular mechanics (MMFF) then DFT (B3LYP/6-31G*) in vacuum. The relative energy of each intermediate was determined by setting the energy of the initial state at 0 kJ/mol. F_{max} values was determined from the slope of the final 40% of the energy/elongation curve (i.e. from E_{max} to 0.6 E_{max} or nearest point).

11.2 Stoppering Ability of the Diels-Alder Adduct

The diameter of the P5Me cavity is ~5 Å while the DA adduct is ~12 Å tall (see CPK models in *Figure S58*, methane is shown for comparison, MMFF). Moreover, the volume of the adduct is ~400 Å^3 (DA adduct without side groups), while the internal cavity of P5Me is ~100-120 Å^3. This factor is particularly striking in the CPK views of the threaded complex which show how the cavity is filled by the alkyl chain of the linker. This was further supported by CoGEF calculations (DFT B3LYP/6-31G*) simulating the elongation of the rotaxane, which confirmed the dissociation of the adduct rather than dethreading (*Figure S59*).

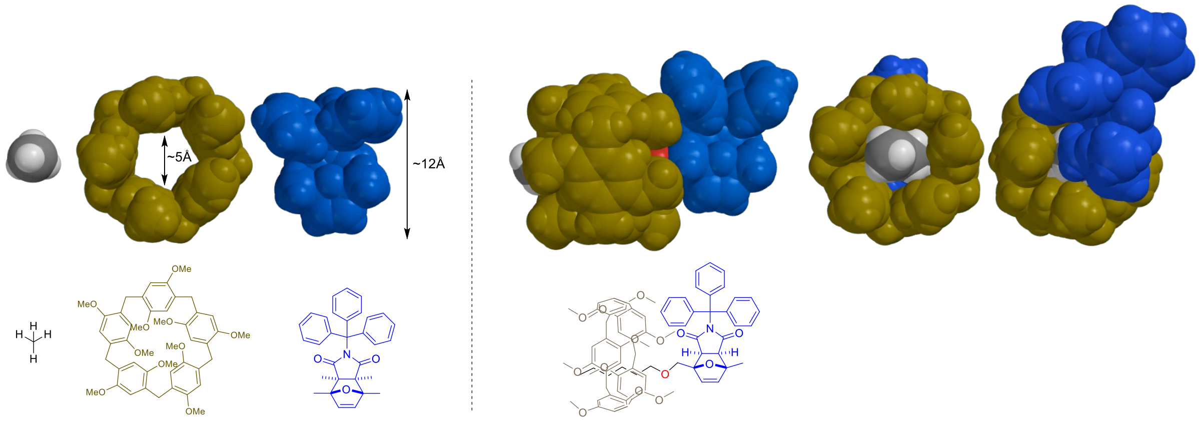

Figure S58. CPK models of the rotaxane components showing their relative size and bulk.

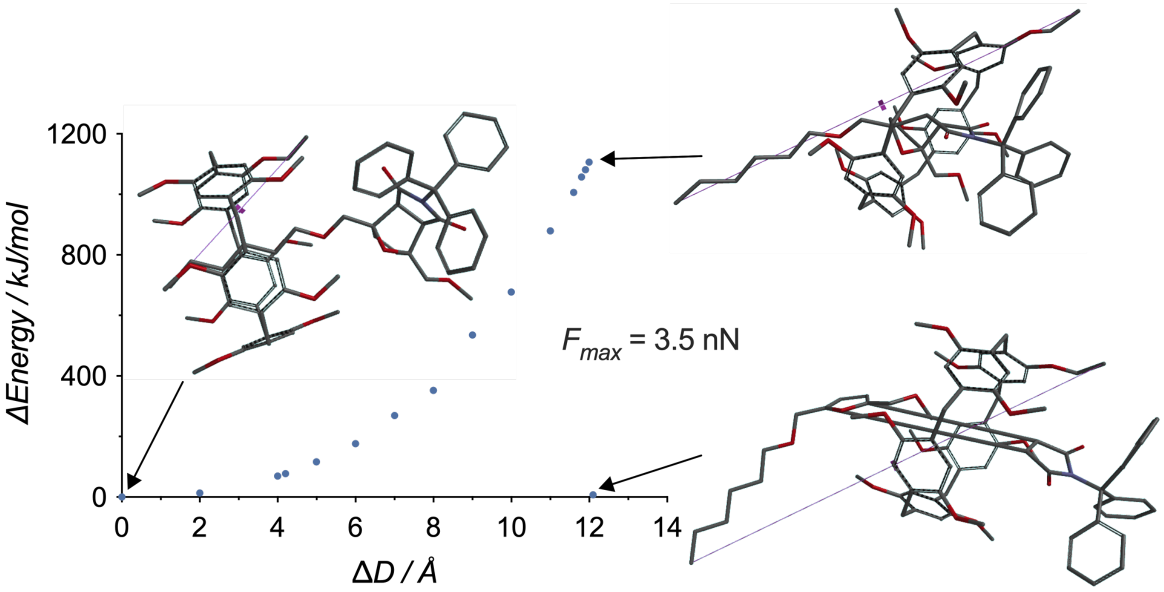

Figure S59. The COGEF profile (DFT, B3LYP/6-31G*) of a model of the *trans-exo* rotaxane, confirms the actuation of the DA adduct by the rotaxane.

12 References

(1) Wang, X. H.; Song, N.; Hou, W.; Wang, C. Y.; Wang, Y.; Tang, J.; Yang, Y. W. Efficient Aggregation-Induced Emission Manipulated by Polymer Host Materials. *Advanced Materials* **31**, 1903962 (2019).

(2) Bang, C. G.; Jensen, J. F.; Cohrt, E. O.; Olsen, L. B.; Siyum, S. G.; Mortensen, K. T.; Skovgaard, T.; Berthelsen, J.; Yang, L.; Givskov, M.; Qvortrup, K.; Nielsen, T. E. A Linker for the Solid-Phase Synthesis of Hydroxamic Acids and Identification of HDAC6 Inhibitors. *ACS Comb. Sci.* **19**, 657–669 (2017).

(3) Sun, H.; Peng, X. Template-Directed Fluorogenic Oligonucleotide Ligation Using "Click" Chemistry: Detection of Single Nucleotide Polymorphism in the Human p53 Tumor Suppressor Gene. *Bioconjugate Chem.* **24**, 1226–1234 (2013).

(4) Li, Changhua; Hu, Jinming; Yin, J.; Liu, S. Click Coupling Fullerene onto Thermoresponsive Water-Soluble Diblock Copolymer and Homopolymer Chains at Defined Positions. *Macromolecules* **42**, 5007–5016 (2009).

(5) Hickenboth, C. R.; Moore, J. S.; White, S. R.; Sottos, N. R.; Baudry, J.; Wilson, S. R. Biasing Reaction Pathways with Mechanical Force. *Nature* **446**, 423–427 (2007).

(6) Beyer, M. The mechanical strength of a covalent bond calculated by density functional theory. *J. Chem. Phys.* **112,** 7307–7312 (2000).
